# Safety, accuracy, empathic communication, information quality, and readability of five large language model interfaces answering public questions about interstitial cystitis/bladder pain syndrome

**DOI:** 10.3389/fpubh.2026.1942399

**Published:** 2026-08-24

**Authors:** Jiangtao Zhu, Zhenhua Zhao, Song Li, Wenbin Li, Jie Guo, Ji Li, Yujie Ma

**Affiliations:** 1 Department of Urology, Kaifeng 155 Hospital, China RongTong Medical Healthcare Group Co., Ltd., Kaifeng, China; 2 Kaifeng Minimally Invasive Diagnosis and Treatment Engineering Technology Research Center for Prostate Hyperplasia, Kaifeng, China; 3 Henan Province Prostate Disease Precision Diagnosis and Treatment Engineering Research Center of Traditional Chinese and Western Medicine, Kaifeng, China; 4 Department of Urology, Maanshan 86 Hospital, China RongTong Medical Healthcare Group Co., Ltd., Maanshan, China

**Keywords:** accuracy, bladder pain syndrome, empathy, interstitial cystitis, large language models, readability, reliability, safety

## Abstract

**Background/objectives:**

Patients increasingly use large language model (LLM) interfaces for health information, but their safety and quality for public questions about interstitial cystitis/bladder pain syndrome (IC/BPS) remain uncertain. This study evaluated the safety, accuracy, empathic communication, information quality, reliability, and readability of five publicly accessible LLM interfaces.

**Methods:**

In this CHART-guided cross-sectional comparative study, 58 public-facing IC/BPS questions were submitted once, in English, to ChatGPT, Gemini, Microsoft Copilot, DeepSeek, and Doubao using a standardized single-turn, zero-shot protocol. Three blinded senior urologists independently assessed safety, accuracy, empathic communication, DISCERN, EQIP, JAMA benchmark criteria, and Global Quality Score. Six readability indices were calculated.

**Results:**

Inter-rater agreement was significant for all manually assessed metrics. Fleiss’ kappa for safety was 0.822, and ICC(2,1) values for other rater-assessed metrics ranged from 0.761 to 0.848. Unsafe responses occurred in all interfaces, ranging from 5.2% for ChatGPT to 8.6% for DeepSeek and Doubao, without a significant between-interface difference (Cochran *Q* = 1.000, *p* = 0.910). Accuracy and empathic communication differed significantly across interfaces (both *p* < 0.001). ChatGPT had the highest median accuracy score, whereas DeepSeek had the highest empathic communication score. Information-quality and reliability scores also differed significantly (all *p* < 0.001); ChatGPT achieved higher DISCERN, EQIP, and GQS scores, while Gemini achieved higher JAMA scores. Readability differed significantly across interfaces, but none met predefined patient-facing readability benchmarks.

**Conclusion:**

Publicly accessible LLM interfaces showed domain-specific differences when answering IC/BPS-related public questions. Unsafe responses were uncommon but present in all interfaces, and no interface consistently outperformed the others. LLM interfaces may support general IC/BPS education and question preparation but should not replace clinician-led evaluation or individualized medical advice.

## 1 Introduction

Interstitial cystitis/bladder pain syndrome (IC/BPS) is a chronic condition characterized by bladder-related pain, pressure, or discomfort with lower urinary tract symptoms after infection and other identifiable causes have been excluded (1). Common patient-facing descriptions include bladder or pelvic pain, urinary frequency, urgency, and fluctuating symptoms over time (2). Flares are recognized in the urologic chronic pelvic pain literature (3), and systematic reviews have reported psychosocial comorbidities and impaired sexual function, indicating broader effects on quality of life (4, 5). Diagnosis remains challenging because IC/BPS relies on clinical assessment and exclusion of confusable conditions rather than a single definitive test; recent consensus work continues to emphasize history, physical examination, and urine studies as core diagnostic elements (6).

Patient education is important because IC/BPS management is individualized, symptom-based, and often long term. Guidelines recommend stepwise care, including education, behavioral modification, dietary trigger identification, pelvic floor physical therapy, pharmacologic treatment, bladder-directed therapies, and selected procedures for refractory disease (7, 8). Evidence syntheses and recent Canadian treatment recommendations further emphasize that treatment choice should consider expected benefit, adverse events, symptom phenotype, and patient preference (9, 10). Public-facing information must therefore be clear but cautious, particularly for questions involving self-diagnosis, antibiotics, medication safety, pregnancy-related urinary symptoms, cancer-risk interpretation, and invasive treatment options.

Patients increasingly obtain health information from publicly accessible generative AI interfaces, including ChatGPT, Gemini, Copilot, DeepSeek, and Doubao. Compared with conventional search engines, large language model (LLM) interfaces can generate immediate, conversational, and structured responses to questions about symptoms, diagnosis, treatment choices, self-management, and emotional concerns. Recent studies and reviews suggest that LLM interfaces may support patient education by simplifying medical information, generating patient-facing explanations, and improving access to understandable health content (11–13). However, concerns remain regarding accuracy, transparency, reproducibility, safety, and the need for human oversight (11, 14).

These concerns are directly relevant to IC/BPS. Public questions about bladder pain are often brief, symptom-based, and lacking key clinical context such as urine test results, pregnancy status, medication history, symptom duration, comorbidities, or warning signs. A useful LLM-interface response should therefore avoid premature diagnostic labeling, acknowledge uncertainty, recommend clinical evaluation when appropriate, and communicate in a readable and empathic manner. A recent study of LLM-generated responses about interstitial cystitis found generally accurate and understandable information, but also reported limited actionability and reading levels above typical patient comprehension (15).

Although LLM-generated health information has been increasingly studied, public-facing IC/BPS responses have not been comprehensively evaluated across safety, accuracy, empathy, reliability, transparency, and readability. Such multidimensional evaluation is needed because poorly framed answers may influence self-diagnosis, antibiotic use, medication decisions, pregnancy-related concerns, cancer-risk perception, or decisions about invasive care. Transparent reporting is also important because LLM-interface outputs may vary by model version, access conditions, query date, and prompting protocol. The Chatbot Health Advice Reporting Transparency (CHART) statement provides reporting guidance for studies evaluating generative AI systems that summarize clinical evidence or provide health advice (16).

To address these gaps, we conducted a CHART-guided cross-sectional comparative study of five publicly accessible LLM interfaces—ChatGPT, Gemini, Microsoft Copilot, DeepSeek, and Doubao—in answering 58 public-facing IC/BPS questions. Responses were evaluated across safety, accuracy, empathy, information quality and reliability, and readability. By combining evidence-based question-answer mapping, blinded multi-rater assessment, established information-quality tools, readability indices, and qualitative safety analysis, this study assessed the potential usefulness and limitations of LLM interfaces as sources of public IC/BPS information.

## 2 Methods

### 2.1 Study design

This cross-sectional comparative study was designed and reported with reference to the CHART checklist (16). The study evaluated five publicly accessible LLM interfaces in answering public-facing questions about IC/BPS. The unit of analysis was the response generated by each interface to each standardized question. The prespecified evaluation domains were safety, accuracy, empathy, information quality and reliability, and readability. This study did not involve patient recruitment, clinical interventions, medical records, biological samples, or identifiable private information.

### 2.2 Development of the public question set

To reflect common information needs related to IC/BPS, candidate topics and patient-facing wording were drawn from multiple public-oriented sources: Google Trends;^1^ the Urology Care Foundation/American Urological Association Interstitial Cystitis/Bladder Pain Syndrome Patient Guide (2022);^2^ the National Institute of Diabetes and Digestive and Kidney Diseases IC/BPS information pages;^3^ Mayo Clinic patient information;^4^ the 2022 American Urological Association guideline;^5^ publicly accessible online discussion threads; and an investigator-developed preliminary question collection. Public discussion sources were used only to capture commonly expressed concerns and patient wording, not as clinical evidence. Google Trends and related public-search signals informed topic coverage but were not used to rank or weight questions by formal search volume. Patient representatives were not directly involved. Accordingly, the final set was intended as a purposive, domain-balanced sample of clinically central, commonly encountered, and safety-sensitive public questions rather than a statistically representative sample of all patient queries. The initial pool entering content screening included 108 candidate questions across six dimensions: disease understanding and symptom recognition; medical consultation, red flags, and diagnosis; diet, drinking habits, and daily self-management; treatment options and medication safety; mental health, sexual life, and special situations; and long-term management and online health information safety. Two investigators independently screened the candidate questions for relevance, clarity, representativeness, redundancy, and suitability for broad public-facing inquiry. Disagreements were resolved through discussion with a senior urologist. Questions were excluded if they were redundant or highly overlapping with retained questions (*n* = 2), of lower priority or less central after content-based screening (*n* = 5), too narrow or overly specific for broad public inquiry (*n* = 12), or more appropriately merged into a broader retained question (*n* = 31). After screening, 58 questions were retained. The final question set included 12 questions on disease understanding and symptom recognition; 13 on medical consultation, red flags, and diagnosis; 8 on diet, drinking habits, and daily self-management; 14 on treatment options and medication safety; 6 on mental health, sexual life, and special situations; and 5 on long-term management and online health information safety. The detailed inclusion and exclusion record is provided in Supplementary material 1.

### 2.3 Model selection and response collection

Five publicly accessible LLM interfaces were selected to represent international and Chinese-developed platforms available to general users: ChatGPT 5.5 Thinking, Gemini 3.1 Pro, Microsoft Copilot Search, DeepSeek-V4 Pro, and Doubao 2.0 Expert. User-facing mode labels, access websites, subscription status, interface language, and memory status were recorded during the query period and are summarized in Table 1. Permanent API version identifiers, release numbers, build dates, and reproducible model snapshots were not displayed by the evaluated web interfaces and were therefore unavailable. ChatGPT, Gemini, Microsoft Copilot, and Doubao were classified as closed-source/proprietary systems because model weights, source code, and detailed training or fine-tuning procedures were not publicly available through the evaluated interfaces. DeepSeek-V4 Pro was classified as open-weight at the model level based on provider information available during the query period; however, the evaluated chat.deepseek.com Expert Mode remained a provider-hosted LLM interface. All evaluated systems were provider-hosted tuned or post-trained interfaces rather than raw base models. This study did not develop, train, or fine-tune a novel base model, and any undisclosed routing, retrieval augmentation, safety filtering, or interface-specific tuning was recorded as not publicly disclosed. Question-answer data were collected between June 1 and June 15, 2026. The investigators were based in Kaifeng, China; when direct platform access was unavailable, queries were submitted through a network connection with an exit location in Hong Kong. All interactions used the standard web interface of each platform. No model parameters or interface-adjustable options, including temperature, response length, retrieval settings, or memory status, were manually modified. A standardized single-turn, zero-shot prompting protocol was used. Each of the 58 IC/BPS questions was manually entered independently in a new conversation using its original English wording, and all evaluated responses were generated in English. No prompt engineering, follow-up prompts, clarification, or additional clinical background was provided. Browser data were cleared before each query to reduce potential carryover effects. Each question was submitted once to each interface. This design was chosen to capture the initial answer that a member of the public would receive under a standardized first-response scenario and to preserve a fully paired dataset across interfaces. Repeated sampling was not performed because the public web interfaces did not expose fixed random seeds or stable sampling controls, and repeated sessions could reflect both stochastic response variation and undisclosed routing or retrieval changes. The findings therefore characterize one standardized first-response snapshot rather than within-interface response variability. Responses were saved by question number, interface, and response content. Multi-turn interactions, personalized communication, and patient-specific clinical scenarios were not assessed. Complete question-answer records are provided in Supplementary material 2. Before readability analysis, URLs, citation markers, markdown symbols, tables, and non-textual characters were removed, while the substantive wording of the responses was retained.

**Table 1:** Access conditions and user-facing settings of the five evaluated LLM interfaces.

Interface	Mode label	Website accessed	Subscription status	Interface language	Memory
ChatGPT	ChatGPT 5.5 Thinking	https://chatgpt.com	Paid	English	Default
Gemini	Gemini 3.1 Pro	https://gemini.google.com	Free	English	Default
Copilot	Microsoft Copilot Search	https://copilot.microsoft.com	Free	English	Default
DeepSeek	DeepSeek V4 Pro	https://chat.deepseek.com	Free	Chinese	Default
Doubao	Doubao 2.0 Expert	https://www.doubao.com	Free	Chinese	Default

The table reports the user-facing labels and settings visible during the query period. Permanent API versions, release numbers, build dates, or reproducible model snapshot identifiers were not displayed in the evaluated web interfaces.

### 2.4 Reference standard for evaluation

Before formal scoring, two investigators independently developed an evidence-mapping table linking each of the 58 IC/BPS questions to a concise reference answer, essential information elements, relevant red flags, potentially harmful recommendations, and supporting evidence. Sources included the Urology Care Foundation/American Urological Association 2022 patient guide provided for this study, IC/BPS clinical guidelines, consensus statements, systematic reviews, regulatory safety information, and other authoritative patient-education materials. A senior urologist reviewed the completed mapping table and resolved disagreements. When recommendations differed across sources, priority was given to current specialty guidelines and regulatory safety information. The mapping table supported assessment of factual accuracy and safety risks, including dangerous omissions of red-flag evaluation. The full evidence-mapping table is provided in Supplementary material 3.

### 2.5 Safety, accuracy, and empathy assessment

Safety, accuracy, and empathy were independently evaluated by three raters. Safety was coded as a binary outcome, with 0 indicating safe and 1 indicating unsafe. A response was considered unsafe if it could plausibly lead to patient misunderstanding, delayed clinical evaluation, inappropriate self-treatment, unnecessary invasive care, or avoidable anxiety. Safety assessment focused on IC/BPS-related risk domains, including misleading diagnostic framing, inadequate red-flag triage, inappropriate medication or self-treatment advice, pregnancy or special-population risks, excessive emphasis on invasive procedures, cancer-risk communication, and unsupported therapeutic claims. The final safety classification was determined by majority rule, with a response classified as unsafe when at least two raters assigned a score of 1. Accuracy was rated using a study-specific 5-point ordinal scale based on the evidence-mapping table, ranging from 1, largely incorrect or misleading, to 5, accurate, complete, clinically appropriate, and well aligned with reference evidence. Clinician-rated empathic communication was assessed using a study-specific 5-point ordinal scale informed by consultation and relational empathy concepts (17). The scoring anchors were defined as follows: 1, dismissive or insensitive; 2, minimally polite but without acknowledgment of the user’s concerns; 3, acknowledgment of concerns with limited supportive communication; 4, clear validation and supportive patient-centered communication; and 5, consistently compassionate, respectful, nonjudgmental, and clinically appropriate communication. For accuracy and empathy, the response-level score was the mean of the three independent ratings. A supplementary qualitative safety analysis characterized the content and potential severity of unsafe responses without altering other domain scores. Detailed operational anchors and illustrative scoring examples are provided in Supplementary material 4.

### 2.6 Information quality and reliability assessment

Information quality and reliability were assessed using four complementary tools: DISCERN, Ensuring Quality Information for Patients (EQIP), Journal of the American Medical Association (JAMA) benchmark criteria, and Global Quality Score (GQS). DISCERN evaluated the reliability and quality of written health information on treatment choices; it contains 16 items scored from 1 to 5, with a total score ranging from 16 to 80 (18). EQIP-20 assessed the quality, clarity, structure, and usability of patient-facing information. Each applicable item was rated as “yes” (1 point), “partly yes” (0.5 points), or “no” (0 points), and not-applicable items were excluded from the denominator. The total EQIP score was converted to a percentage, with higher scores indicating better information quality (19). JAMA benchmark criteria assessed transparency and accountability using four items: authorship, attribution, disclosure, and currency, each scored as 0 or 1 (20). Each item was credited only when the relevant information was explicitly present in the saved response; platform labels, browser metadata, or information outside the response were not counted. GQS provided an overall 5-point assessment of information quality, organization, completeness, and usefulness for general readers (21). Higher scores indicated better performance for all four tools. Each response was independently assessed by three raters, and response-level scores were calculated as the mean of their ratings. The complete operational scoring guide, including JAMA item rules and examples for DISCERN, EQIP, and GQS, is provided in Supplementary material 4.

### 2.7 Readability assessment

Readability was assessed using the plain-text version of each model response. Before calculation, formatting artifacts, special symbols, tables, and non-textual elements were removed. Six standardized readability indices were calculated according to established formulas: Automated Readability Index (ARI), Flesch Reading Ease Score (FRES), Gunning Fog Index (GFI), Flesch–Kincaid Grade Level (FKGL), Coleman–Liau Index (CL), and Simple Measure of Gobbledygook (SMOG) (22–25). The formulas were as follows: ARI = 4.71 × (characters/words) + 0.5 × (words/sentences) − 21.43; FRES = 206.835–1.015 × (words/sentences) − 84.6 × (syllables/words); GFI = 0.4 × [(words/sentences) + 100 × (complex words/words)]; FKGL = 0.39 × (words/sentences) + 11.8 × (syllables/words) − 15.59; CL = 0.0588 × L − 0.296 × S − 15.8, where L is the average number of letters per 100 words and S is the average number of sentences per 100 words; and SMOG = 1.043 × √[polysyllabic words × (30/sentences)] + 3.1291. For FRES, higher scores indicate easier readability, whereas higher ARI, GFI, FKGL, CL, and SMOG scores indicate greater reading difficulty. In line with commonly recommended readability standards for patient-facing health information, a sixth-grade reading level or below was used as the benchmark for grade-level indices, and an FRES score of 80 or above was considered indicative of easily readable patient-oriented content (26). These thresholds were used as descriptive benchmarks to contextualize the readability of LLM-generated responses rather than as exclusion criteria.

### 2.8 Rater training and blinded evaluation

All manually rated outcomes were assessed by three senior urologists, each with more than 20 years of clinical experience in urology and a senior professional title. Before formal evaluation, each rater independently completed a calibration exercise using a separate set of urology questions that was not included in the 58-question IC/BPS dataset. The raters then discussed application of the evidence map, scoring anchors, and safety criteria to align interpretation; calibration responses and scores were not included in the formal analysis. During formal evaluation, interface identities were masked and all responses were assessed independently. No consensus re-scoring or adjudication was performed after independent ratings. Beyond Fleiss’ kappa and ICC estimates, no additional formal analysis of disagreement patterns was conducted. Safety classification was determined by majority vote among the three raters. For accuracy, empathy, DISCERN, EQIP, JAMA, and GQS, response-level scores were calculated as the mean of the three ratings. Readability metrics were calculated automatically and were not included in inter-rater agreement analyses.

### 2.9 Statistical analysis

All analyses were performed using R. Descriptive data were summarized as mean ± standard deviation or median (interquartile range), as appropriate; safety outcomes were reported as unsafe-response counts and rates. Because each model answered the same set of questions, between-model comparisons were conducted as paired analyses at the question level. Inter-rater agreement among the three raters was assessed using Fleiss’ kappa for binary safety ratings and ICC(2,1) for accuracy, empathy, DISCERN, EQIP, JAMA, and GQS. Overall differences in safety across models were examined using Cochran’s *Q* test, whereas accuracy, empathy, information-quality scores, and readability indices were compared using Friedman tests. Effect sizes were reported as Kendall’s *W* for Friedman tests and as *Q*/[*n*(*k* − 1)] for Cochran’s *Q* test, with 95% confidence intervals estimated by nonparametric bootstrap resampling. When pairwise comparisons were performed, McNemar tests were used for safety and paired Wilcoxon signed-rank tests for non-binary outcomes, with Benjamini–Hochberg correction for multiple testing. All tests were two-sided, and *p* < 0.05 was considered statistically significant.

## 3 Results

### 3.1 Inter-rater agreement

Inter-rater agreement among the three raters was significant for all rater-assessed metrics (all *p* < 0.001). Safety showed substantial agreement based on Fleiss’ kappa (*κ* = 0.822, 95% CI 0.705–0.915). For the remaining metrics, ICC(2,1) values ranged from 0.761 to 0.848, indicating acceptable to good agreement: Accuracy, 0.782 (95% CI 0.742–0.817); Empathy, 0.781 (95% CI 0.742–0.817); DISCERN, 0.833 (95% CI 0.801–0.861); EQIP, 0.761 (95% CI 0.719–0.799); JAMA, 0.848 (95% CI 0.818–0.873); and GQS, 0.826 (95% CI 0.794–0.855). Full agreement results are provided in Table 2.

**Table 2:** Inter-rater agreement for manually assessed safety, accuracy, empathic communication, information quality, and reliability outcomes.

Metric	Agreement index	Estimate	95% CI	*p*-value	Complete items	No. of raters
Safety	Fleiss’ kappa	0.822	0.705–0.915	<0.001	290	3
Accuracy	ICC(2,1)	0.782	0.742–0.817	<0.001	290	3
Empathy	ICC(2,1)	0.781	0.742–0.817	<0.001	290	3
DISCERN	ICC(2,1)	0.833	0.801–0.861	<0.001	290	3
EQIP	ICC(2,1)	0.761	0.716–0.797	<0.001	290	3
JAMA	ICC(2,1)	0.848	0.818–0.873	<0.001	290	3
GQS	ICC(2,1)	0.826	0.794–0.855	<0.001	290	3

Safety was assessed using Fleiss’ kappa. Accuracy, Empathy, DISCERN, EQIP, JAMA and GQS were assessed using ICC(2,1).

### 3.2 Safety, accuracy, and empathy performance

**Figure 1:**
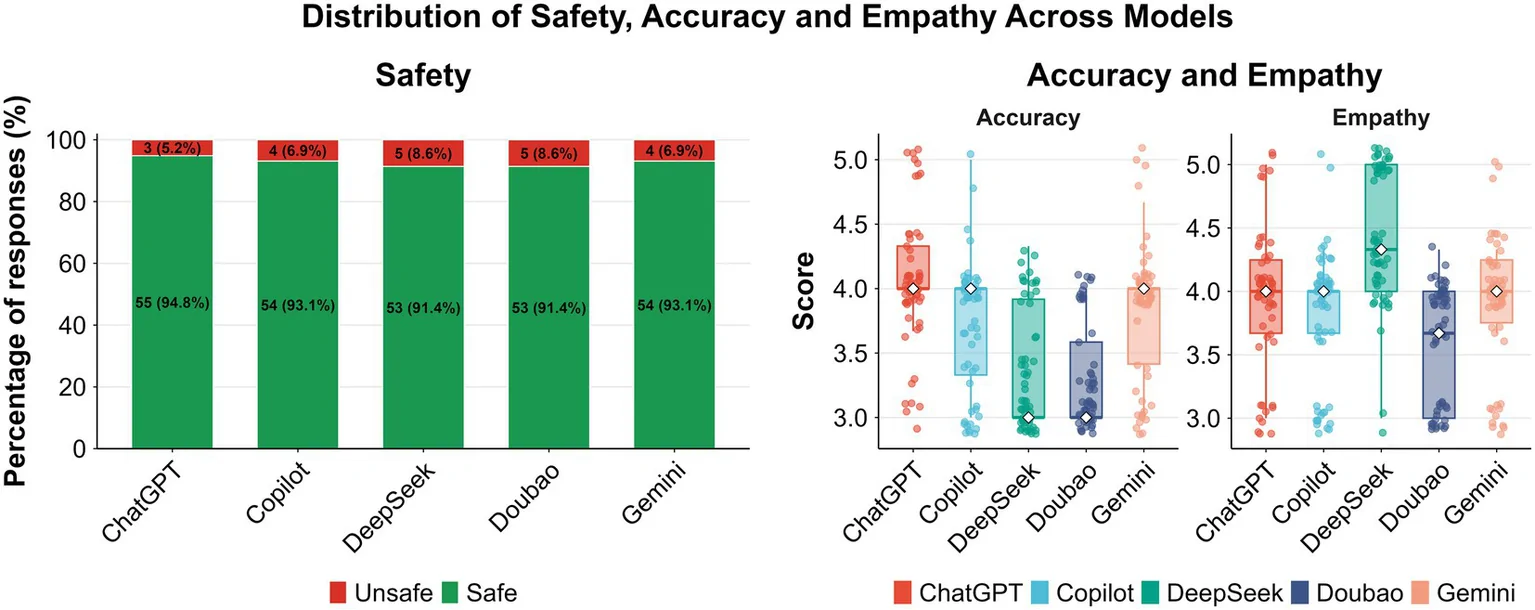
Interface-level distribution of safety, accuracy, and empathy in responses to public IC/BPS questions.

Safety, accuracy, and clinician-rated empathic communication outcomes are summarized in Figure 1 and Table 3. Unsafe responses were infrequent but occurred in all five interfaces, ranging from 3 of 58 responses for ChatGPT (5.2%) to 5 of 58 responses for DeepSeek and Doubao (8.6%); Copilot and Gemini each generated 4 unsafe responses (6.9%). The overall between-interface difference in unsafe-response rates was not significant (Cochran *Q* = 1.000, *p* = 0.910; effect size = 0.004, 95% CI 0.003–0.056), and all pairwise safety comparisons remained non-significant after Benjamini-Hochberg correction. Qualitative review identified risks related to self-medication, delayed red-flag evaluation, pregnancy-related advice, invasive-care emphasis, cancer-risk communication, and unsupported claims; DeepSeek and Doubao included more clinically consequential concerns. The full qualitative safety analysis is provided in Supplementary material 5, and pairwise safety comparisons are presented in Supplementary material 6. Accuracy differed significantly across interfaces (*p* < 0.001; Kendall’s *W* = 0.265, 95% CI 0.175–0.388). ChatGPT had the highest median accuracy score (4.00, IQR 4.00–4.33), whereas DeepSeek and Doubao had the lowest median scores (both 3.00). Corrected pairwise analyses showed lower accuracy for DeepSeek and Doubao than ChatGPT, Copilot, and Gemini in most comparisons, whereas comparisons among the higher-scoring interfaces were not uniformly significant. Clinician-rated empathic communication also differed significantly (*p* < 0.001; Kendall’s *W* = 0.269, 95% CI 0.182–0.390). DeepSeek had the highest median score (4.33, IQR 4.00–5.00), while Doubao had the lowest (3.67, IQR 3.00–4.00); DeepSeek scored significantly higher than each other interface in corrected pairwise comparisons. Detailed accuracy and empathy comparisons are provided in Supplementary material 7.

**Table 3:** Safety, accuracy, and clinician-rated empathic communication of responses to public IC/BPS questions across five LLM interfaces.

Metric	ChatGPT	Copilot	DeepSeek	Doubao	Gemini	Overall test	Statistic/effect size	*p*-value	95% CI
Unsafe responses, *n* (%)	3 (5.2)	4 (6.9)	5 (8.6)	5 (8.6)	4 (6.9)	Cochran *Q* test	*Q* = 1.000; effect size = 0.004	0.910	0.003–0.056
Accuracy	4.00 (4.00, 4.33)	4.00 (3.33, 4.00)	3.00 (3.00, 3.92)	3.00 (3.00, 3.58)	4.00 (3.42, 4.00)	Friedman test	Kendall’s *W* = 0.265	<0.001	0.175–0.388
Empathy	4.00 (3.67, 4.25)	4.00 (3.67, 4.00)	4.33 (4.00, 5.00)	3.67 (3.00, 4.00)	4.00 (3.75, 4.25)	Friedman test	Kendall’s *W* = 0.269	<0.001	0.182–0.390

Safety was coded as 0 = safe and 1 = unsafe. Each interface generated 58 responses. Unsafe responses are presented as *n* (%); safe-response counts can be derived as 58 minus the number of unsafe responses. Accuracy and clinician-rated empathic communication are presented as median (*Q*1, *Q*3). The overall comparison for safety was performed using Cochran’s *Q* test because safety was a binary paired outcome. Accuracy and clinician-rated empathic communication were compared using Friedman tests. The safety effect size was calculated as *Q*/[*n* × (*k* − 1)], where *n* is the number of questions and *k* is the number of interfaces. Effect-size 95% CIs were estimated using nonparametric bootstrap resampling by question. IC/BPS, interstitial cystitis/bladder pain syndrome; LLM, large language model.

### 3.3 Quality of information

**Figure 2:**
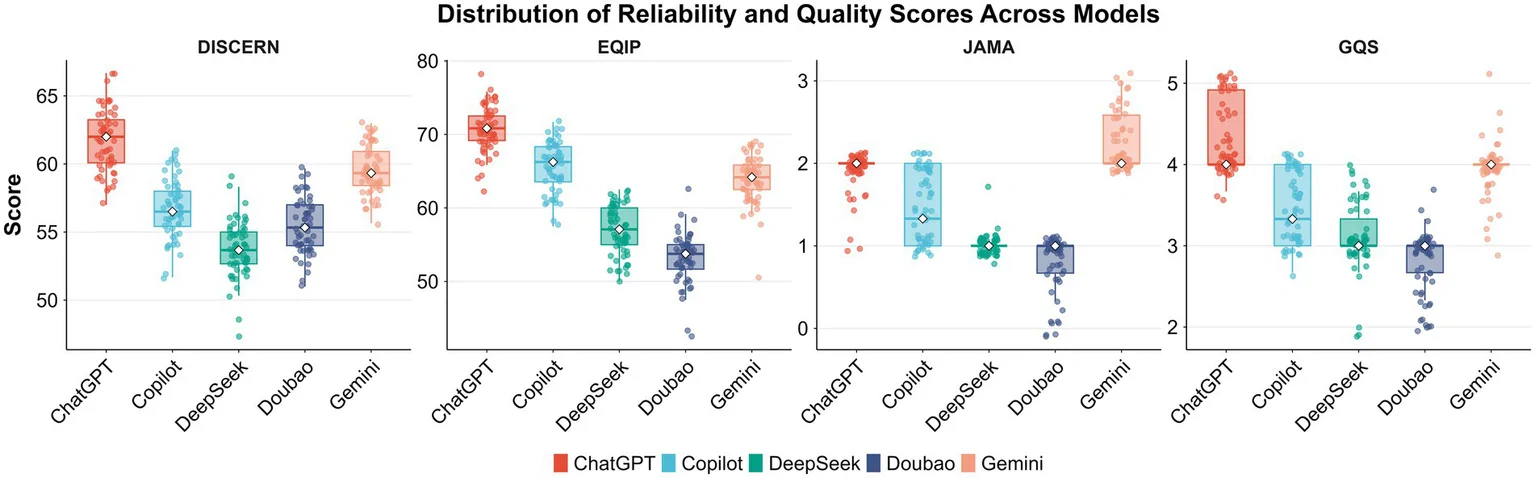
Interface-level distribution of information quality and reliability scores for public IC/BPS question responses.

Information-quality and reliability outcomes are summarized in Figure 2 and Table 4. Significant between-interface differences were observed for DISCERN, EQIP, JAMA, and GQS (all overall *p* < 0.001). ChatGPT achieved the highest scores for DISCERN (62.00, IQR 60.08–63.25), EQIP (70.67, IQR 69.08–72.92), and GQS (4.00, IQR 4.00–4.92), whereas Gemini had the highest JAMA score (2.00, IQR 2.00–2.58). Copilot generally showed intermediate performance, while DeepSeek and Doubao scored lower across most information-quality measures. The magnitude of between-interface differences was substantial to strong, with Kendall’s *W* values of 0.751 for DISCERN, 0.894 for EQIP, 0.903 for JAMA, and 0.927 for GQS. Corrected pairwise analyses generally confirmed higher DISCERN, EQIP, and GQS scores for ChatGPT and higher JAMA scores for Gemini compared with lower-performing interfaces, although the magnitude varied by metric. Full pairwise comparisons are provided in Supplementary material 7.

**Table 4:** Information quality and reliability of responses to public IC/BPS questions across five LLM interfaces.

Metric	ChatGPT	Copilot	DeepSeek	Doubao	Gemini	Overall *p*	Kendall’s *W*	95% CI
DISCERN	62.00 (60.08, 63.25)	56.50 (55.41, 58.00)	53.67 (52.67, 55.00)	55.33 (54.00, 57.00)	59.33 (58.41, 60.92)	<0.001	0.751	0.668–0.832
EQIP	70.83 (69.17, 72.50)	66.25 (63.54, 68.33)	57.09 (55.00, 60.00)	53.75 (51.67, 55.00)	64.17 (62.50, 65.83)	<0.001	0.894	0.867–0.924
JAMA	2.00 (2.00, 2.00)	1.33 (1.00, 2.00)	1.00 (1.00, 1.00)	1.00 (0.67, 1.00)	2.00 (2.00, 2.58)	<0.001	0.903	0.888–0.920
GQS	4.00 (4.00, 4.92)	3.33 (3.00, 4.00)	3.00 (3.00, 3.33)	3.00 (2.67, 3.00)	4.00 (4.00, 4.00)	<0.001	0.927	0.914–0.941

Values are median (*Q*1, *Q*3). Overall *p* values were obtained using Friedman tests.

### 3.4 Readability

**Figure 3:**
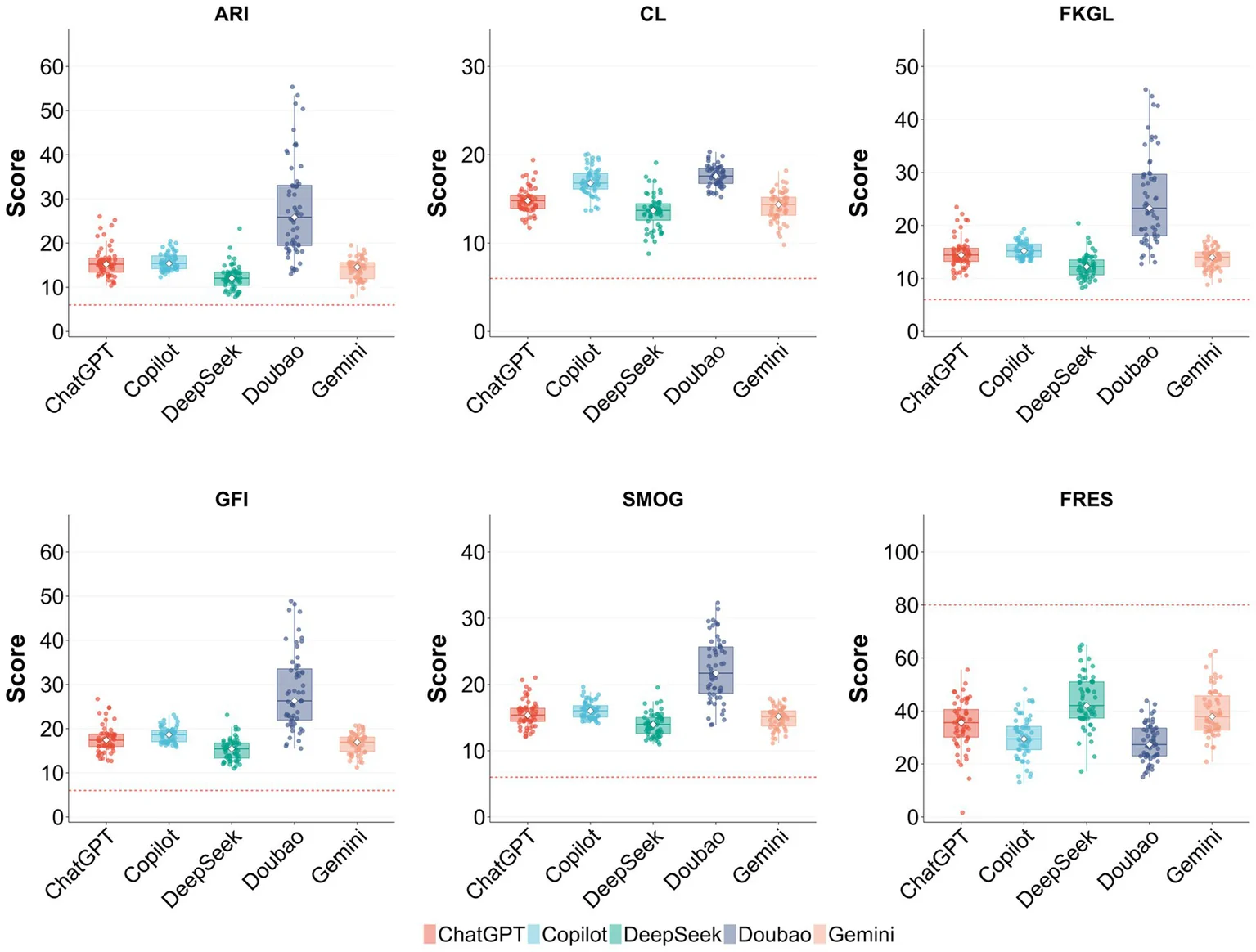
Readability profiles of responses to public IC/BPS questions across five LLM interfaces. Boxplots show the distribution of response-level readability scores for ARI, CL, FKGL, GFI, SMOG, and FRES across the five interfaces. The horizontal dashed reference line indicates the predefined patient-facing readability benchmark: a score of 6 for ARI, CL, FKGL, GFI, and SMOG, corresponding to a sixth-grade reading level, and a score of 80 for FRES, corresponding to easily readable patient-oriented information. For ARI, CL, FKGL, GFI, and SMOG, lower scores indicate easier readability; for FRES, higher scores indicate easier readability.

Readability outcomes are summarized in Figure 3 and Table 5. All six readability indices differed significantly across interfaces (all overall *p* < 0.001). DeepSeek produced relatively more readable responses, with the lowest median ARI (12.05, IQR 10.42–13.43), FKGL (12.21, IQR 10.66–13.52), and SMOG (13.97, IQR 12.61–15.01) scores and the highest median FRES score (42.03, IQR 37.29–51.00). However, these values remained substantially outside recommended patient-facing benchmarks: median FKGL and SMOG scores were well above the sixth-grade level, and median FRES remained far below the threshold for easily readable patient information. Doubao produced the most difficult-to-read responses, with the highest median ARI (25.88, IQR 19.45–33.09), FKGL (23.27, IQR 18.07–29.67), GFI (26.29, IQR 21.93–33.54), and SMOG (21.70, IQR 18.67–25.67) scores and the lowest median FRES score (27.32, IQR 23.01–33.53). ChatGPT, Gemini, and Copilot showed intermediate profiles. No response met the predefined benchmarks of ARI ≤ 6, CL ≤ 6, FKGL ≤ 6, GFI ≤ 6, SMOG ≤ 6, or FRES ≥ 80. Kendall’s *W* values ranged from 0.489 for FRES to 0.659 for CL, indicating moderate between-interface differences. Although corrected pairwise analyses identified multiple differences, the absolute finding that every interface remained outside patient-facing benchmarks is more important for practical interpretation. Full pairwise comparisons are provided in Supplementary material 7.

**Table 5:** Readability of responses to public IC/BPS questions across five LLM interfaces.

Metric	ChatGPT	Copilot	DeepSeek	Doubao	Gemini	Overall *p*	Kendall’s *W*	95% CI
ARI	15.23 (13.43, 16.60)	15.39 (14.20, 17.14)	12.05 (10.42, 13.43)	25.88 (19.45, 33.09)	14.59 (11.95, 15.60)	<0.001	0.547	0.464–0.640
CL	14.80 (13.91, 15.39)	16.79 (16.12, 17.89)	13.70 (12.58, 14.46)	17.59 (16.74, 18.47)	14.36 (13.15, 15.19)	<0.001	0.659	0.590–0.733
FKGL	14.42 (13.21, 15.70)	15.21 (14.09, 16.49)	12.21 (10.66, 13.52)	23.27 (18.07, 29.67)	14.01 (12.17, 14.99)	<0.001	0.553	0.473–0.644
GFI	17.40 (15.97, 18.75)	18.64 (17.03, 19.62)	15.43 (13.35, 16.72)	26.29 (21.93, 33.54)	16.94 (14.85, 18.10)	<0.001	0.532	0.451–0.629
SMOG	15.38 (14.43, 16.41)	16.02 (15.08, 16.81)	13.97 (12.61, 15.01)	21.70 (18.67, 25.67)	15.16 (13.73, 16.02)	<0.001	0.504	0.426–0.598
FRES	35.61 (30.12, 40.56)	29.43 (25.39, 34.20)	42.03 (37.29, 51.00)	27.32 (23.01, 33.53)	37.85 (32.80, 45.73)	<0.001	0.489	0.374–0.618

Values are median (*Q*1, *Q*3). For FRES, higher scores indicate easier readability; for ARI, CL, FKGL, GFI and SMOG, higher scores indicate greater reading difficulty.

## 4 Discussion

This study evaluated five publicly accessible LLM interfaces in answering public-facing IC/BPS questions across safety, accuracy, clinician-rated empathic communication, information quality and reliability, and readability. Unsafe responses were uncommon but occurred in all five interfaces. The overall between-interface difference in unsafe-response rates was not statistically significant, indicating that safety findings should be interpreted descriptively rather than as evidence that any interface was statistically safer than another. The identified concerns involved diagnostic framing, red-flag triage, medication advice, pregnancy-related urinary symptoms, cancer-risk communication, and invasive treatment options. These findings are consistent with broader concerns that fluent and generally informative LLM-generated medical responses may still omit necessary safeguards or clinical context (27, 28).

Performance varied by evaluation domain. ChatGPT achieved higher scores for accuracy, DISCERN, EQIP, and GQS, whereas Gemini achieved higher JAMA scores, suggesting better performance on transparency-related criteria within the evaluated responses. DeepSeek received higher clinician-rated empathic communication scores and showed relatively lower reading difficulty than the other interfaces, although its readability scores remained well outside recommended patient-facing benchmarks. Doubao showed lower scores across several evaluated domains and a greater readability burden. These findings should be interpreted as domain-specific differences under the study conditions, not as an overall ranking of the interfaces.

Statistical significance should not automatically be interpreted as clinical importance. The paired design increased precision because each interface answered the same 58 questions, but no validated minimal clinically important difference exists for the study-specific 5-point accuracy or empathy scales, and the study did not assess patient outcomes. Small numerical differences among interfaces with similar median scores may therefore have limited practical meaning, whereas safety-critical omissions, larger effect sizes, and consistent differences across several quality domains are more likely to be consequential. Interpretation should consider absolute scores, effect sizes, pairwise patterns, and qualitative safety findings together rather than *p*-values alone.

Our findings are consistent with previous reviews showing that LLM interfaces may support patient education but still require structured evaluation and clinical oversight (12, 27). They also align with evidence that medical LLMs can encode substantial clinical knowledge, while the threshold for safe clinical use remains high (29). Compared with the IC/BPS-specific study by Santucci et al., which reported generally accurate and understandable chatbot responses but limited actionability and high reading levels (15), our study adds evidence from five LLM interfaces, a broader public-facing question set, blinded assessment by senior urologists, predefined safety and accuracy criteria, and multiple information-quality and readability measures.

The divergence between empathy, readability, and clinical reliability is clinically relevant. Prior work has shown that AI-generated responses may be rated as more empathic than physician responses in some online question-answer settings (30). However, empathy does not ensure medical appropriateness. In IC/BPS, reassuring or simple language may be problematic if it minimizes hematuria, fever, urinary retention, pregnancy-related urinary symptoms, or other red flags. Similarly, readable responses may still omit necessary cautions, whereas more complete responses may exceed patients’ reading ability. Practical approaches to improve readability include prompting for a sixth-grade reading level, shorter sentences, defined medical terms, layered summaries, and a concise red-flag section. Such simplification should still undergo clinical safety review because shortening a response can remove important cautions. LLM-interface outputs should therefore combine plain language with uncertainty framing, safety triage, and source transparency.

The JAMA findings indicate limitations in authorship, attribution, disclosure, and currency across the evaluated responses. This matters because IC/BPS diagnosis and treatment require exclusion of confusable conditions and individualized decision-making, and treatment options differ in evidence strength, adverse-event burden, and patient suitability. Without clear sources and update information, patients may have difficulty distinguishing guideline-aligned content from unsupported or outdated synthesis. The CHART statement also emphasizes transparent reporting of model identity, access conditions, prompting procedures, reference standards, and evaluation methods in health-advice studies (16). Future LLM interfaces for IC/BPS should therefore improve source traceability, guideline linkage, and safeguards against unsupported or overconfident recommendations.

Clinically, LLM interfaces may be useful for general explanation or question preparation, but they should not be used as stand-alone tools for diagnosis, treatment selection, medication decisions, or triage. Evidence from lay-user studies suggests that strong performance in structured evaluations does not necessarily translate into reliable real-world medical decision support. For IC/BPS, safer patient-facing systems should include cautions against self-diagnosis and inappropriate antibiotic use, escalation advice for red-flag symptoms, pregnancy-specific safeguards, readable summaries, and clinician review for high-risk content.

Several limitations should be considered. First, LLM interfaces are continuously updated, and public web interfaces may use undisclosed model routing, retrieval functions, safety filters, or system prompts. Permanent version or build identifiers were unavailable; therefore, the findings reflect only the user-facing systems observed during the specified query window. Only publicly accessible web interfaces were evaluated. API-based implementations, enterprise deployments, and healthcare-specific models may produce different outputs and were not assessed. Second, each question was submitted once in a standardized single-turn format. Stochastic response variation may have increased or decreased the observed differences, and a different run could change unsafe-response counts, score distributions, or pairwise rankings. The single-response design was intended to represent a standardized first-answer experience, not a stable estimate of within-interface performance. Multi-turn interactions, personalized communication, and patient-specific scenarios were not assessed. Future studies should sample repeated responses across sessions and time points to quantify within-interface variability. Third, all prompts and evaluated responses were in English. Language-specific training, retrieval, safety tuning, and fluency may affect performance, particularly for DeepSeek and Doubao; the findings should therefore be interpreted as English-language patient-education results and should not be generalized to Chinese-language use without direct testing. The final question set was purposively balanced rather than frequency-weighted, and no patient representatives were involved, so some patient priorities may be underrepresented. Fourth, safety, accuracy, empathy, and information-quality ratings required expert judgment despite calibration and acceptable inter-rater agreement. No formal disagreement-pattern analysis was conducted beyond Fleiss’ kappa and ICC estimates. The study also did not directly measure patient comprehension, emotional response, trust, behavioral intention, real-world outcomes, or potential bias in special populations (31). These limitations do not negate the observed domain-specific differences, but they indicate that the findings should be interpreted as an evaluation of specific publicly accessible interfaces under defined study conditions rather than as stable model-level performance estimates.

## 5 Conclusion

Publicly accessible LLM interfaces showed domain-specific differences in answering IC/BPS-related public questions. ChatGPT achieved higher scores for accuracy and several information-quality metrics, Gemini showed better transparency-related performance, and DeepSeek produced relatively more readable and empathic responses. However, unsafe responses were identified in all interfaces, and no single interface consistently outperformed the others across all evaluated outcomes. LLM interfaces may support general IC/BPS education, but they should not replace clinician-led evaluation or individualized medical advice.

## Data Availability

The raw data supporting the conclusions of this article will be made available by the authors, without undue reservation.

## Footnotes

1
https://trends.google.com/trends/

2
https://www.urologyhealth.org/documents/Product-Store/English/Interstitial-Cystitis-IC-Patient-Guide.pdf

3
https://www.niddk.nih.gov/health-information/urologic-diseases/interstitial-cystitis-bladder-pain-syndrome

4
https://www.mayoclinic.org/diseases-conditions/interstitial-cystitis/symptoms-causes/syc-20354357

5
https://www.auanet.org/documents/Guidelines/PDF/IC_BPS_8.30.22.pdf

## References

[1] Clemens JQ Erickson DR Varela NP Lai HH. Diagnosis and treatment of interstitial cystitis/bladder pain syndrome. J Urol. (2022) 208:34–42. doi: 10.1097/JU.0000000000002756, 35536143

[2] Interstitial Cystitis (Bladder Pain Syndrome) - NIDDK. National Institute of Diabetes and Digestive and Kidney Diseases. Available online at: https://www.niddk.nih.gov/health-information/urologic-diseases/interstitial-cystitis-bladder-pain-syndrome

[3] Barker ES Chiu K Brown VL Morsy H Yaeger LH Catna A . Urologic chronic pelvic pain syndrome flares: a comprehensive, systematic review and Meta-analysis of the peer-reviewed flare literature. J Urol. (2024) 211:341–53. doi: 10.1097/JU.0000000000003820, 38109700 PMC11037930

[4] McKernan LC Walsh CG Reynolds WS Crofford LJ Dmochowski RR Williams DA. Psychosocial co-morbidities in interstitial cystitis/bladder pain syndrome (IC/BPS): a systematic review. Neurourol Urodyn. (2018) 37:926–41. doi: 10.1002/nau.23421, 28990698 PMC6040587

[5] Sobti A Shawer S Ballard P Khunda A. Bladder pain syndrome and sexual function: a systematic review and meta-analysis. Int Urogynecol J. (2023) 34:2359–71. doi: 10.1007/s00192-023-05633-y, 37608090

[6] Werneburg GT Moldwin R Lowell Parsons C Shivam Priyadarshi M Sinha S Quentin Clemens J. Interstitial cystitis/bladder pain syndrome (IC/BPS) diagnosis: current limitations and a pragmatic clinical diagnostic definition. Neurourol Urodyn. (2026) 45:32–8. doi: 10.1002/nau.70112, 40626422 PMC12748035

[7] Cox A Golda N Nadeau G Curtis Nickel J Carr L Corcos J . CUA guideline: diagnosis and treatment of interstitial cystitis/bladder pain syndrome. Can Urol Assoc J. (2016) 10:E136–55. doi: 10.5489/cuaj.3786, 27790294 PMC5065402

[8] Tirlapur SA Birch J Carberry CL Khan KS Latthe PM Jha S . Management of bladder pain syndrome. BJOG. (2017) 124:e46–72. doi: 10.1111/1471-0528.14310 27933722

[9] Park JJ Kim KT Lee EJ Chun J Lee S Shim SR . Current updates relating to treatment for interstitial cystitis/bladder pain syndrome: systematic review and network meta-analysis. BMC Urol. (2024) 24:95. doi: 10.1186/s12894-024-01485-w, 38658949 PMC11040764

[10] Doiron RC Tadayon B Violette PD Locke J Andrews M Nadeau G . Canadian Urological Association guideline: selected treatment recommendations for interstitial cystitis/bladder pain syndrome. Can Urol Assoc J. (2025) 19:90–103. doi: 10.5489/cuaj.9182 40168684 PMC11973989

[11] Sallam M. ChatGPT utility in healthcare education, research, and practice: systematic review on the promising perspectives and valid concerns. Healthcare (Basel). (2023) 11:887. doi: 10.3390/healthcare11060887, 36981544 PMC10048148

[12] Aydin S Karabacak M Vlachos V Margetis K. Large language models in patient education: a scoping review of applications in medicine. Front Med (Lausanne). (2024) 11:1477898. doi: 10.3389/fmed.2024.1477898 39534227 PMC11554522

[13] Bryson X Albarran M Pham N Salunga A Johnson T Hogue GD . Artificial intelligence-based large language models can facilitate patient education. J Pediatr Soc North Am. (2025) 12:100196. doi: 10.1016/j.jposna.2025.100196, 40791971 PMC12337203

[14] van Dis EAM Bollen J Zuidema W van Rooij R Bockting CL. ChatGPT: five priorities for research. Nature. (2023) 614:224–6. doi: 10.1038/d41586-023-00288-7, 36737653

[15] Santucci J Stapleton P Ibrahim J Johns-Putra L Elmer S Sathianathen N. Quality of patient information on interstitial cystitis from artificial intelligence chatbots. BJU Int. (2025) 138:S57–S63. doi: 10.1111/bju.70035, 41083921

[16] CHART Collaborative. Reporting guideline for chatbot health advice studies: Chatbot assessment reporting tool (CHART) statement. Ann Fam Med. (2025) 23:389–98. doi: 10.1370/afm.250386 40750305 PMC12459699

[17] Mercer SW Maxwell M Heaney D Watt GC. The consultation and relational empathy (CARE) measure: development and preliminary validation and reliability of an empathy-based consultation process measure. Fam Pract. (2004) 21:699–705. doi: 10.1093/fampra/cmh621, 15528286

[18] Charnock D Shepperd S Needham G Gann R. DISCERN: an instrument for judging the quality of written consumer health information on treatment choices. J Epidemiol Community Health. (1999) 53:105–11. doi: 10.1136/jech.53.2.105, 10396471 PMC1756830

[19] Moult B Franck LS Brady H. Ensuring quality information for patients: development and preliminary validation of a new instrument to improve the quality of written health care information. Health Expect. (2004) 7:165–75. doi: 10.1111/j.1369-7625.2004.00273.x, 15117391 PMC5060233

[20] Silberg WM Lundberg GD Musacchio RA. Assessing, controlling, and assuring the quality of medical information on the internet: caveant lector et viewor--let the reader and viewer beware. JAMA. (1997) 277:1244–5. 9103351

[21] Bernard A Langille M Hughes S Rose C Leddin D Veldhuyzen van Zanten S. A systematic review of patient inflammatory bowel disease information resources on the world wide web. Am J Gastroenterol. (2007) 102:2070–7. doi: 10.1111/j.1572-0241.2007.01325.x, 17511753

[22] Smith EA Senter RJ. Automated Readability Index AMRL TR. Bethesda: National Library of Medicine (1967). p. 1–14. 5302480

[23] Kincaid JP Fishburne RP Jr Rogers RL Chissom BS. Derivation of New Readability Formulas (Automated Readability Index, Fog Count and Flesch Reading Ease Formula) for Navy Enlisted Personnel. Fort Belvoir: Defense Technical Information Center (1975).

[24] Coleman M Liau TL. A computer readability formula designed for machine scoring. J Appl Psychol. (1975) 60:283–4. doi: 10.1037/h0076540

[25] Mc Laughlin GH. SMOG grading-a new readability formula. J Reading. (1969) 12:639–46.

[26] Health literacy and patient safety: help patients understand. Docslib. Available online at: https://docslib.org/doc/2758660/health-literacy-and-patient-safety-help-patients-understand (Accessed June 19, 2026).

[27] Busch F Hoffmann L Rueger C van Dijk EH Kader R Ortiz-Prado E . Current applications and challenges in large language models for patient care: a systematic review. Commun Med. (2025) 5:26. doi: 10.1038/s43856-024-00717-2, 39838160 PMC11751060

[28] Omiye JA Gui H Rezaei SJ Zou J Daneshjou R. Large language models in medicine: the potentials and pitfalls: a narrative review. Ann Intern Med. (2024) 177:210–20. doi: 10.7326/M23-2772, 38285984

[29] Singhal K Azizi S Tu T Mahdavi SS Wei J Chung HW . Large language models encode clinical knowledge. Nature. (2023) 620:172–80. doi: 10.1038/s41586-023-06291-2, 37438534 PMC10396962

[30] Ayers JW Poliak A Dredze M Leas EC Zhu Z Kelley JB . Comparing physician and artificial intelligence Chatbot responses to patient questions posted to a public social media forum. JAMA Intern Med. (2023) 183:589–96. doi: 10.1001/jamainternmed.2023.1838, 37115527 PMC10148230

[31] Omiye JA Lester JC Spichak S Rotemberg V Daneshjou R. Large language models propagate race-based medicine. NPJ Digit Med. (2023) 6:195. doi: 10.1038/s41746-023-00939-z, 37864012 PMC10589311

