# Supplementary material for: Safety, accuracy, empathic communication, information quality, and readability of five large language model interfaces answering public questions about interstitial cystitis/bladder pain syndrome
Source: Front Public Health. 2026 Aug 24;14:1942399. doi: 10.3389/fpubh.2026.1942399 (PMC13547468; doi:10.3389/fpubh.2026.1942399)
Supplement: Supplementary file 2 [file Supplementary_file_2.docx]

**Supplementary Material 2. Complete Question-Answer Records**

Model-generated responses to the standardized public-facing IC/BPS question set are presented below.

**ChatGPT**

**1.What is interstitial cystitis?**

Interstitial cystitis, also called **bladder pain syndrome**, is a long-lasting condition that causes **pain, pressure, or discomfort in the bladder or pelvic area**, often along with needing to urinate often or urgently. The pain may get worse as the bladder fills and may feel better after urinating.

It can feel like a urinary tract infection, but **there is usually no infection found**. Symptoms can come and go, and flares may be triggered by things such as stress, certain foods, menstruation, sitting for a long time, exercise, or sexual activity.

Common symptoms include bladder or pelvic pain, frequent urination, urgent need to urinate, pain as the bladder fills, and sometimes pain during sex. The exact cause is not known, and diagnosis usually involves ruling out other problems with similar symptoms, such as urinary tract infection, stones, overactive bladder, or other pelvic conditions.

There is no single cure, but many people improve with a combination of lifestyle changes, bladder training, pelvic floor physical therapy, medicines, or bladder treatments.

**2.Is interstitial cystitis the same as bladder pain syndrome?**

Yes—**in most current medical use, interstitial cystitis and bladder pain syndrome refer to the same condition**, often written together as **IC/BPS**.

There is a small nuance:

**Interstitial cystitis** is the older term. It suggests bladder inflammation, but many people with the condition do not have obvious inflammation.

**Bladder pain syndrome** is a newer, broader term that focuses on the main symptom: chronic bladder or pelvic pain, pressure, or discomfort, usually with urinary frequency or urgency, and without another clear cause such as infection. The CDC says interstitial cystitis is also called bladder pain syndrome, and NIDDK uses the combined term “Interstitial Cystitis (Bladder Pain Syndrome).”

So, practically: **IC = BPS = IC/BPS**, though some clinicians may use the terms slightly differently.

It is **not the same as a urinary tract infection**. IC/BPS can feel like a UTI, but urine tests usually do not show infection, and antibiotics usually do not help unless there is a true infection.

**3.Is interstitial cystitis the same as a bladder infection?**

No. **Interstitial cystitis (IC), also called bladder pain syndrome, is not the same as a bladder infection.**

A **bladder infection** is usually a bacterial UTI. It often causes burning when peeing, urgency, frequent urination, cloudy or smelly urine, and sometimes blood; it is typically diagnosed with urine testing and treated with antibiotics.

**Interstitial cystitis** is a **chronic bladder/pelvic pain condition** that can feel like a UTI, but it is **not caused by a routine bacterial infection**. Symptoms often include bladder pressure or pain, pelvic pain, frequent urination, urgency, and pain that may worsen as the bladder fills and improve after urinating. Diagnosis usually involves ruling out other causes, including UTI.

The tricky part is that the symptoms can overlap. A urine dipstick/culture can help tell whether bacteria are present, and it is especially important to check for infection if symptoms are new, sudden, or different from your usual pattern.

Seek medical care promptly if you have **fever, chills, back/flank pain, nausea/vomiting, pregnancy, visible blood in urine, or worsening severe pain**, because those can point to a kidney infection or another issue that needs treatment.

**4.Can interstitial cystitis happen even when there is no infection?**

Yes. **Interstitial cystitis**, also called **bladder pain syndrome**, can happen **without a urinary infection**. In fact, IC/BPS is generally considered a chronic bladder/pelvic pain condition, and doctors diagnose it partly by ruling out other causes such as a UTI. Urine testing is often used to check for infection before settling on IC/BPS.

Typical IC/BPS symptoms can include bladder or pelvic pain/pressure, urinary urgency or frequency, pain that worsens as the bladder fills and may improve after urinating, and flare-ups triggered by things like stress, menstruation, sex, exercise, sitting, or certain foods.

That said, **UTI-like symptoms with no infection are not automatically IC**. Similar symptoms can come from pelvic floor dysfunction, vaginal/urethral irritation, kidney stones, sexually transmitted infections, endometriosis, overactive bladder, or other causes. A clinician may do a urine culture, pelvic exam, symptom history/bladder diary, and sometimes cystoscopy or other tests depending on the situation.

Seek prompt medical care if you have fever, back/flank pain, blood in urine, pregnancy, vomiting, inability to urinate, severe worsening pain, or symptoms that keep recurring despite negative cultures.

**5.Is interstitial cystitis a type of bladder cancer?**

No. **Interstitial cystitis (IC), also called bladder pain syndrome, is not a type of bladder cancer.** It is a chronic bladder/pelvic pain condition that can cause bladder pressure, pain, urgency, and frequent urination, often without infection.

That said, **IC and bladder cancer can sometimes share symptoms**, such as urinary urgency, frequency, pelvic/bladder discomfort, or pain with urination. A key red flag for bladder cancer is **blood in the urine**, which the American Cancer Society says is often the first sign, though blood in urine is more often caused by non-cancer conditions.

Doctors usually diagnose IC **after ruling out other causes** of similar symptoms, such as UTI, stones, endometriosis, overactive bladder, and—when symptoms warrant it—bladder cancer.

You should get medical evaluation promptly if you have **visible blood in urine, persistent microscopic blood, unexplained weight loss, worsening pain, smoking history, age over 50, or symptoms that are new, one-sided, severe, or not behaving like your usual IC flares**.

**6.Is interstitial cystitis caused by stress or anxiety?**

Stress or anxiety **is not considered the root cause** of interstitial cystitis/bladder pain syndrome (IC/BPS). The exact cause of IC/BPS is still unknown, and proposed contributors include bladder-lining dysfunction, inflammation, immune factors, nerve/pain-processing changes, heredity, infection history, or allergy-like mechanisms.

But stress and anxiety **can absolutely worsen symptoms or trigger flares**. The American Urological Association says psychological stress is associated with increased pain sensitivity, lab studies show stress can increase IC/BPS symptoms, and patients are encouraged to use stress-management practices for stress-induced symptom exacerbations. Mayo Clinic also lists stress among common triggers for symptom flares.

So the best way to frame it is:

**Stress/anxiety usually does not “cause” IC, but it can amplify bladder pain, urgency, frequency, pelvic floor tension, and flares once someone has IC/BPS or a similar pelvic pain condition.**

It is also a two-way loop: IC symptoms can cause poor sleep, worry, activity limits, and emotional distress, which can then make the nervous system more sensitized and worsen symptoms. Mayo Clinic notes IC can lead to emotional stress and depression because of chronic pain and sleep disruption.

Stress management can help, but it should not be treated as “it’s all in your head.” IC/BPS is a real chronic pain/urologic condition, and evaluation should still rule out things like UTI, stones, endometriosis, pelvic floor dysfunction, overactive bladder, vaginal/urethral conditions, and prostatitis depending on anatomy and symptoms. The AUA recommends history, physical exam, and lab testing to document symptoms and exclude other causes.

**7.Does interstitial cystitis usually last for a long time?**

Yes. **Interstitial cystitis, also called bladder pain syndrome, is usually considered a chronic or long-lasting condition**. Symptoms can last months to years, but the pattern varies a lot: they may improve, flare, disappear for a while, and then come back days, weeks, months, or even years later.

It does **not** necessarily mean constant severe pain forever. Many people get meaningful relief with a mix of approaches such as diet/fluid trigger changes, bladder training, pelvic floor physical therapy, medicines, or bladder-directed treatments; often it takes trying several options to find what helps.

It is also important to make sure it really is IC/BPS, because UTI, stones, endometriosis, pelvic floor dysfunction, overactive bladder, STIs, and other issues can mimic it. Seek prompt medical care if you have fever, flank/back pain, visible blood in urine, pregnancy, new severe pain, or inability to urinate.

**8.Can interstitial cystitis symptoms come and go over time?**

Yes. **Interstitial cystitis/bladder pain syndrome symptoms commonly come and go over time.** They can flare for days, weeks, or longer, then improve or even disappear for a while, and later return after days, weeks, months, or years. NIDDK notes that IC symptoms vary, change over time, may go away, and may later return.

Flares can happen without an obvious reason, but common triggers include **stress, menstruation, sitting for a long time, exercise, sexual activity**, and sometimes certain foods or drinks. Mayo Clinic also notes that some people have symptom-free periods.

That said, symptoms that feel like IC can also be caused by things like a **UTI, sexually transmitted infection, kidney stone, pelvic floor dysfunction, endometriosis, prostatitis**, or other bladder/gynecologic issues. It’s worth getting checked, especially if symptoms are new, changing, or more intense than usual.

Seek medical care promptly if you have **fever, flank/back pain, blood in urine, pregnancy, vomiting, inability to urinate, or UTI-like symptoms that are worsening**, because those are not things to just assume are IC.

**9.What are the most common symptoms of interstitial cystitis?**

The most common symptoms of **interstitial cystitis**, also called **bladder pain syndrome**, are:

**Bladder or pelvic pain, pressure, or discomfort**

**Pain that worsens as the bladder fills and improves after urinating**

**Frequent urination**, often in small amounts, during the day and night

**Urgent need to urinate**, sometimes even when the bladder is not full

**Lower abdominal or pelvic tenderness**

**Pain during sex**, often related to pelvic floor muscle spasms

Symptoms that **flare and improve over time**, sometimes triggered by stress, menstruation, prolonged sitting, exercise, sexual activity, or certain foods/drinks

IC can feel similar to a UTI, but urine testing often shows **no infection**. Symptoms vary a lot from person to person and may come and go.

You should contact a healthcare provider if you have **chronic bladder pain, urinary urgency, or frequent urination**, especially if symptoms persist or keep returning. Seek more urgent care if you have fever, back/flank pain, blood in urine, vomiting, pregnancy, or inability to urinate, since those can point to other conditions.

**10.Can interstitial cystitis cause frequent urination?**

Yes. **Interstitial cystitis/bladder pain syndrome can cause frequent urination**, often with urgency and passing small amounts. It can also cause bladder or pelvic pressure/pain that may worsen as the bladder fills and improve after urinating. Mayo Clinic notes frequency can occur day and night, sometimes very often in severe cases.

That said, **frequent urination alone does not prove interstitial cystitis**. Other common causes include a urinary tract infection, overactive bladder, pregnancy, diabetes/high blood sugar, high caffeine or fluid intake, some medications, kidney issues, and prostate problems in men. NIDDK describes IC/BPS as a chronic condition diagnosed after ruling out other similar causes.

You should get checked promptly if you have **burning, fever, back/flank pain, blood in the urine, new severe pelvic pain, pregnancy, excessive thirst/weight loss, or symptoms that came on suddenly**, because those may point to infection or another condition needing treatment.

A typical first step is a **urinalysis/urine culture** and review of symptoms, fluids/caffeine, medications, and whether pain/pressure changes with bladder filling or urination.

**11.Can interstitial cystitis make someone feel an urgent need to urinate?**

Yes. **Interstitial cystitis/bladder pain syndrome can cause urinary urgency**—a persistent or sudden-feeling need to urinate, sometimes even when the bladder is not full. It commonly also causes **frequent urination**, bladder/pelvic pressure or pain, and discomfort that may worsen as the bladder fills and improve after urinating.

That said, urgency can also come from **UTI, overactive bladder, pregnancy, diabetes, kidney/bladder stones, sexually transmitted infections, medication effects, pelvic floor issues**, and other causes. IC is usually considered after other causes—especially infection—are ruled out.

Seek medical care promptly if urgency comes with **fever, back/flank pain, blood in urine, vomiting, pregnancy, inability to urinate, severe pelvic pain, or new symptoms that are rapidly worsening**. Otherwise, it is still worth seeing a clinician if urgency/frequency persists or recurs.

**12.Can interstitial cystitis cause bladder pressure without severe pain?**

Yes. **Interstitial cystitis / bladder pain syndrome can cause bladder pressure, fullness, discomfort, or urgency even when the pain is mild rather than severe.** Medical references describe IC/BPS symptoms as ranging from *mild discomfort or pressure* to intense pain, and symptoms can vary over time.

A common pattern is pressure or discomfort that **gets worse as the bladder fills** and may **ease temporarily after urinating**, though not everyone has this exact pattern. IC/BPS can also cause frequent urination and urgency “with or without pain.”

That said, **bladder pressure is not specific to IC**. A UTI, overactive bladder, pelvic floor dysfunction, endometriosis/gynecologic issues, prostatitis, kidney/bladder stones, medication effects, pregnancy, or other urinary conditions can feel similar. IC/BPS is usually considered after other causes, especially infection, are ruled out.

You should get medical care promptly if you have **fever, back/flank pain, blood in urine, vomiting, pregnancy, inability to urinate, new severe pain, or symptoms of a UTI**, or if this pressure is new or persistent. A basic urinalysis/urine culture is often the first step before assuming IC.

**13.Why is interstitial cystitis often hard to diagnose?**

Interstitial cystitis/bladder pain syndrome is often hard to diagnose because **there isn’t one simple, definitive test for it**. Clinicians usually diagnose it by looking at the symptom pattern and **ruling out other conditions** that can cause similar urinary urgency, frequency, pressure, burning, or pelvic pain.

The main reasons it gets tricky:

**It mimics many other problems**
IC can feel like a UTI, overactive bladder, pelvic floor dysfunction, endometriosis, prostatitis/chronic pelvic pain syndrome, urethral pain syndrome, kidney stones, sexually transmitted infections, or sometimes bladder irritation from medications, foods, or gynecologic issues.

**Urine tests may be normal**
A person can have UTI-like urgency, frequency, burning, or pressure, but urine culture may show no infection. That pushes doctors to rule out other explanations rather than immediately “prove” IC. NIDDK notes that urinalysis and urine culture are commonly used to look for infection or blood and to rule out similar conditions.

**Symptoms vary a lot**
Some people have severe pain; others mainly have pressure, urgency, frequency, or discomfort. Symptoms can flare and settle, and Mayo Clinic notes they may vary over time and be triggered by things like menstruation, sitting, stress, exercise, or sexual activity.

**Cystoscopy can be normal**
A cystoscopy may help rule out other problems, check bladder capacity, or look for certain findings, but a normal-looking bladder does not always make symptoms simple to explain. Mayo lists cystoscopy, biopsy, urine cytology, and other tests as possible parts of the workup, especially to rule out cancer or rare causes of bladder pain.

**It overlaps with pelvic floor dysfunction**
Tight or irritated pelvic floor muscles can cause bladder pressure, urgency, burning, incomplete emptying sensations, and pelvic pain. This can exist alongside IC or be mistaken for it.

**It is a “syndrome,” not one single disease**
IC/BPS likely includes multiple underlying mechanisms in different people, so two patients can both receive the diagnosis but have different dominant symptoms and triggers.

A practical way to think about it: **IC/BPS is often diagnosed after repeated symptoms persist, infection and other causes are reasonably excluded, and the symptom pattern fits bladder/pelvic pain or pressure with urinary urgency/frequency.** A bladder diary, urine culture, pelvic exam/pelvic floor assessment, and sometimes cystoscopy are commonly used pieces of the puzzle.

**14.When should someone with possible interstitial cystitis see a doctor?**

Someone with possible interstitial cystitis/bladder pain syndrome should **see a doctor if bladder pressure, pelvic discomfort, urinary urgency, or frequent urination lasts more than a few days, keeps coming back, or affects sleep, sex, work, or daily life**. IC is usually diagnosed by **ruling out other causes**, especially UTI, stones, pelvic floor problems, gynecologic/prostate conditions, and sometimes bladder cancer; urine testing is commonly part of that workup.

Seek care **sooner** if symptoms are new for you, you have burning or pain with urination, symptoms after sex, pregnancy, recurrent “UTI-like” symptoms with negative cultures, or pain/urgency that flares repeatedly. IC symptoms can include bladder/pelvic pressure or tenderness, frequent urination, urgency with or without pain, and pain that may worsen as the bladder fills and ease after urinating.

Get **urgent medical care now** if there is **fever, chills, back/flank pain, vomiting, visible blood in the urine, inability to urinate, severe worsening pain, confusion, or feeling very unwell**, because these can suggest infection spreading to the kidneys, urinary retention, stones, or another condition that should not be managed as IC.

A practical rule: if it feels like a UTI, don’t assume it’s IC—get a urine test/culture first. If tests are negative but symptoms persist or recur, ask a primary care doctor, gynecologist, or urologist about evaluation for IC/bladder pain syndrome and related pelvic floor conditions. Mayo Clinic and NIDDK both describe diagnosis as involving history, exam, bladder diary, urine testing, and sometimes cystoscopy or other tests depending on the case.

**15.What symptoms with interstitial cystitis should be checked urgently?**

With **interstitial cystitis/bladder pain syndrome (IC/BPS)**, bladder/pelvic pain, urgency, frequency, burning, flares after sex/foods/stress, and pain that improves after urinating can happen. IC can mimic a UTI, but IC itself usually is **not an infection**.

Seek **urgent medical care today**—urgent care, same-day doctor, or emergency department depending on severity—if you have any of these:

**Fever, chills, feeling very unwell, or shaking**

**Back or side/flank pain**, especially with fever

**Nausea or vomiting**

**Visible blood in urine**, new clots, or urine that looks red/tea-colored

**Severe or rapidly worsening bladder/pelvic pain**

**Painful, frequent, or urgent urination lasting several hours or longer**, especially if new or different from your usual IC flare

**Cloudy or foul-smelling urine**, or symptoms like a prior UTI

**Unable to pass urine**, or severe difficulty urinating

**Pregnancy**, male urinary symptoms, kidney disease, diabetes, immune suppression, or recent urinary procedure/catheter

The biggest concern is that a presumed IC flare could actually be a **UTI, kidney infection, kidney stone, urinary retention, or another cause of blood/pain**. Mayo Clinic advises immediate help for kidney-infection-type symptoms such as **back/side pain, fever/chills, nausea/vomiting**, and calling a clinician for **blood in urine** or persistent painful/urgent/frequent urination.

For **non-urgent but still important** follow-up, contact your clinician/urologist if your IC symptoms are becoming more frequent, your usual flare plan is not working, symptoms are new for you, or you have chronic bladder pain/urgency/frequency that has not been evaluated. Diagnosis often includes checking urine for infection and sometimes tests to rule out other causes such as cancer in selected cases.

**16.Should blood in the urine be checked if someone has interstitial cystitis symptoms?**

Yes. **Blood in the urine should be checked even if someone already has interstitial cystitis/bladder pain syndrome symptoms.** IC can sometimes be associated with microscopic blood or bleeding during flares, but **you should not assume IC is the cause** until other causes are ruled out.

Visible blood — pink, red, brown, cola-colored urine, or clots — should be assessed by a clinician. Mayo Clinic says to see a healthcare provider whenever urine looks like it might contain blood, because hematuria can come from UTIs, kidney infection, stones, kidney disease, medicines, injury, or less commonly bladder/kidney/prostate cancer.

For **urgent same-day care**, especially consider urgent care/ER if blood comes with:

fever, chills, feeling very unwell

back/flank pain or severe abdominal/pelvic pain

vomiting

inability to urinate, trouble passing urine, or clots

pregnancy

recent injury

heavy bleeding or worsening visible blood

A typical evaluation may include a urinalysis and urine culture, and depending on age/risk factors/symptoms, imaging such as ultrasound or CT and sometimes cystoscopy.

Bottom line: **microscopic “trace blood” can happen in IC, but new, persistent, or visible blood should be checked.** If the person is seeing red/pink/brown urine, they should contact a healthcare provider promptly rather than treating it as just an IC flare.

**17.Should fever with bladder pain be treated as interstitial cystitis?**

No. **Fever with bladder pain should not be assumed to be an interstitial cystitis flare.** IC/bladder pain syndrome is typically a chronic bladder/pelvic pain condition **without infection**, and diagnosis involves ruling out other causes. Fever suggests something else may be going on, especially a **urinary tract infection**, possibly a **kidney infection**, or another pelvic/abdominal infection.

You should seek **same-day medical advice/urgent care** if bladder pain comes with fever, chills, feeling very unwell, nausea/vomiting, flank or back pain, worsening burning with urination, blood in urine, pregnancy, male sex, immunosuppression, kidney disease, or inability to pass urine.

A practical next step is a **urinalysis and urine culture before antibiotics when possible**, because IC symptoms can resemble UTI symptoms, and a UTI can also worsen IC-type symptoms. Mayo Clinic notes that IC usually has no infection, but symptoms can worsen if someone with IC gets a UTI.

Until you’re checked, treat fever + bladder pain as **possible infection**, not just IC. If the fever is high, you have flank pain, vomiting, confusion, weakness, or rapid worsening, go to emergency care.

**18.What should someone do if interstitial cystitis pain suddenly becomes much worse?**

Sudden much-worse “IC pain” should **not automatically be assumed to be an IC flare**. IC symptoms can flare, but IC is diagnosed by ruling out other causes, and symptoms can worsen if someone with IC also gets a UTI.

**Seek urgent medical care now** if any of these are present:

Fever, chills, feeling very unwell, nausea/vomiting

New back/flank pain, especially one-sided

Blood in the urine, clots, or urine that looks red/brown

Severe pain that is new, escalating, or not controlled with usual measures

Trouble passing urine, inability to urinate, or markedly reduced urine

Pregnancy, kidney disease, immune suppression, diabetes, or recent urinary procedure

New vaginal/testicular pain, severe abdominal pain, or concern for STI/pelvic infection

Those symptoms can point to infection, kidney involvement, a stone, obstruction, or another condition needing prompt assessment.

For a sudden flare **without red flags**, the safer next step is to **contact their doctor/urologist promptly** and ask whether they need a urinalysis and urine culture. IC often causes urgency, frequency, bladder/pelvic pain, and pain that may worsen as the bladder fills and improve after urination, but similar symptoms can come from other conditions.

While waiting for medical advice, they can consider conservative flare steps they already know are safe for them: drink water normally rather than overhydrating, avoid common bladder irritants such as caffeine/alcohol/spicy or acidic foods, use heat or cold on the pelvic area, rest, and take only pain medicines they can safely use. They should avoid starting leftover antibiotics unless a clinician tells them to.

A practical rule: **sudden severe worsening, fever, flank pain, visible blood, vomiting, or inability to pee = urgent care/emergency evaluation, not “just IC.”**

**19.What should someone do if they cannot urinate while having interstitial cystitis symptoms?**

Not being able to urinate is **not something to treat as a normal interstitial cystitis flare**. It can be **acute urinary retention**, which needs **urgent medical assessment**, especially if there is bladder/lower abdominal pain, swelling, or a strong urge to pee with little or no urine. NIDDK lists inability to urinate, severe lower abdominal pain, urgent need to urinate, and lower abdominal swelling as symptoms of acute urinary retention.

They should:

**Seek urgent care now** — emergency department or urgent clinic. A clinician may need to check bladder volume and drain the bladder with a catheter if urine is retained.

**Do not keep forcing or straining** to urinate, and do not drink large amounts of fluid to “push it out,” because that can worsen bladder distension.

**Tell the clinician about IC symptoms**, but also ask to rule out other causes such as UTI, urethral blockage/stricture, kidney stone, medication side effects, constipation, pelvic floor spasm, neurologic issues, or prostate-related obstruction if applicable.

**Go immediately/emergency** if there is fever, back/flank pain, vomiting, blood in urine, severe pelvic/lower abdominal pain, confusion, weakness/numbness, or pregnancy.

IC commonly causes bladder/pelvic pain, urgency, and frequent small urinations, but diagnosis involves ruling out other conditions with similar symptoms; sudden inability to urinate should be checked rather than assumed to be IC.

**20.What type of doctor usually treats interstitial cystitis?**

Interstitial cystitis/bladder pain syndrome is **usually treated by a urologist**—a doctor who specializes in the urinary tract and bladder.

Depending on the person’s symptoms, care may also involve:

**Urogynecologist:** especially for women or people with pelvic floor, bladder, or pelvic pain issues.

**Pelvic floor physical therapist:** Mayo Clinic notes that physical therapy may help when pelvic floor muscle tenderness, tight connective tissue, or muscle problems contribute to pain.

**Primary care doctor or gynecologist:** often helps with first evaluation, urine testing, ruling out UTI or other causes, and referral.

A good fit is usually a **urologist or urogynecologist who specifically has experience with IC/bladder pain syndrome**, because treatment often requires trying several approaches rather than one single cure. Mayo Clinic notes that no one treatment works for everyone, and combinations may be needed.

**21.How do doctors usually diagnose interstitial cystitis?**

Doctors usually diagnose **interstitial cystitis/bladder pain syndrome (IC/BPS)** by **recognizing the symptom pattern and ruling out other causes**, not by one single definitive test.

Typical workup:

**History and symptom pattern**
They ask about bladder/pelvic pain, urinary urgency/frequency, pain that worsens as the bladder fills and improves after urinating, triggers, flares, and how long symptoms have been going on. A **bladder diary/voiding log** is often used.

**Physical exam**
This may include an abdominal and pelvic exam, and sometimes a rectal exam, to check for pelvic floor tenderness, gynecologic issues, prostate-related pain, or other causes.

**Urine testing**
A **urinalysis and often a urine culture** are used to rule out UTI, blood in the urine, or inflammation. If blood is present or risk factors exist, doctors may investigate further.

**Ruling out similar conditions**
Depending on symptoms, they may consider UTIs, STIs, overactive bladder, kidney stones, endometriosis, pelvic floor dysfunction, prostatitis, vaginal/urethral conditions, or, less commonly, bladder cancer.

**Cystoscopy only when needed**
A cystoscopy can look inside the bladder and urethra. It is often used when symptoms are atypical, there is blood in the urine, the diagnosis is uncertain, or the doctor wants to look for **Hunner lesions**, which are a specific bladder finding seen in some IC/BPS patients. It is **not always required** for diagnosis. NIDDK lists cystoscopy as a test used to rule out other conditions or guide treatment decisions.

**Other tests in selected cases**
Urodynamic testing, urine cytology, biopsy, imaging, or STI testing may be used when the clinician needs to rule out other diagnoses. Mayo Clinic notes that biopsy or urine cytology may be used to check for cancer or rare causes of bladder pain in certain situations.

So, in plain terms: **IC/BPS is usually a clinical diagnosis of exclusion**—persistent bladder/pelvic pain with urinary urgency or frequency, no infection or obvious alternative cause, and sometimes supportive findings on cystoscopy or other testing.

**22.Is there one test that can prove someone has interstitial cystitis?**

No. **There is no single test that can prove someone has interstitial cystitis/bladder pain syndrome (IC/BPS).** It is usually a **clinical diagnosis**, meaning doctors diagnose it based on symptoms, exam findings, bladder/pelvic history, and by **ruling out other causes** such as UTI, kidney stones, sexually transmitted infections, gynecologic conditions, overactive bladder, pelvic floor dysfunction, and sometimes bladder cancer.

Common tests may include **urinalysis and urine culture** to check for infection, a pelvic or rectal exam, a bladder diary, and sometimes **cystoscopy** or urodynamic testing—but these are usually used to rule out other problems or guide treatment, not to “prove” IC by themselves.

A cystoscopy can sometimes find **Hunner lesions**, which strongly support an IC/BPS subtype, but many people with IC/BPS have a normal-looking bladder. So a normal cystoscopy does **not** rule it out.

A good way to think of it: IC/BPS is diagnosed when the symptom pattern fits—typically bladder/pelvic pain, pressure, urgency, and frequency—especially when urine cultures are negative and other likely causes have been excluded.

**23.Why do doctors need to rule out other problems before diagnosing interstitial cystitis?**

Doctors need to rule out other problems first because **interstitial cystitis/bladder pain syndrome (IC/BPS) does not have one definitive diagnostic test**. It is usually diagnosed when a person has typical bladder/pelvic pain and urinary symptoms, but **infection and other identifiable causes have been excluded**. The American Urological Association says the basic workup should include history, physical exam, and lab testing to document IC/BPS-type symptoms and exclude other disorders; symptoms are generally expected to last at least **six weeks** with negative urine cultures.

The main reasons are:

**Many conditions mimic IC.** UTIs, sexually transmitted infections, kidney stones, overactive bladder, endometriosis, pelvic floor dysfunction, vaginitis, prostatitis, bladder cancer, and medication/chemical irritation can all cause urgency, frequency, burning, pelvic pain, or bladder pressure.

**Some mimics are treatable or urgent.** A bacterial UTI needs antibiotics; stones may need imaging or procedures; blood in the urine may need evaluation for tumors or other causes. Missing these by labeling symptoms as IC too early could delay the right treatment.

**IC treatments differ from treatments for other causes.** IC/BPS care often focuses on bladder irritant avoidance, pelvic floor physical therapy, bladder training, pain control, and sometimes bladder-directed medications or procedures. Those may not help, and could distract from, a different diagnosis.

**Tests are mainly used to exclude, not “prove,” IC.** Urinalysis and urine culture check for infection or blood; pelvic/rectal exam can identify pelvic floor or reproductive causes; cystoscopy or urodynamics may be used in complex cases but are not always routine. NIDDK and Johns Hopkins both describe IC diagnosis as ruling out similar conditions because no single test confirms it.

So the “rule out” process is not doctors doubting the pain. It is how they make sure the diagnosis is safe, accurate, and that something more directly treatable is not being missed.

**24.Why do doctors check urine when interstitial cystitis is suspected?**

Doctors check urine when interstitial cystitis/bladder pain syndrome is suspected mainly to **rule out other causes that can look very similar**, especially a **urinary tract infection**.

A urine test can help check for:

**UTI signs** — white blood cells, nitrites, bacteria, or a positive urine culture. UTIs can cause urgency, frequency, burning, pelvic pain, and bladder pain, just like IC, but UTIs are treated with antibiotics. NIDDK notes that urinalysis checks for blood and white blood cells, while urine culture looks for bacteria.

**Blood in the urine** — IC can sometimes be associated with microscopic blood, but blood can also come from stones, infection, kidney problems, or rarely bladder cancer, so doctors do not want to assume it is IC. The AUA guideline says the basic IC/BPS workup includes urinalysis and urine culture, and that unexplained blood in urine should be evaluated appropriately.

**Evidence that symptoms are not from infection** — IC is generally considered when bladder/pelvic pain and urinary symptoms persist, often with **negative urine cultures**. The AUA describes IC/BPS symptoms as lasting at least six weeks with documented negative cultures.

So the urine test is less about “proving IC” and more about making sure the symptoms are **not from infection or another condition that needs different treatment**. A normal or negative urine culture supports the possibility of IC, but it does not diagnose IC by itself.

**25.Is looking inside the bladder always needed to diagnose interstitial cystitis?**

No. **Looking inside the bladder with cystoscopy is not always needed to diagnose interstitial cystitis / bladder pain syndrome**, especially when the symptoms are typical and the initial workup does not suggest another problem.

The American Urological Association says the basic diagnosis is usually based on **history, physical exam, and lab tests** to document symptoms and rule out other causes. It specifically states that **cystoscopy and/or urodynamics are not necessary in uncomplicated presentations**, but should be considered when the diagnosis is uncertain.

Cystoscopy is more likely to be recommended when:

There is **blood in the urine**, especially unexplained blood.

Symptoms are unusual or the diagnosis is unclear.

The doctor needs to rule out things like **bladder cancer, stones, foreign body, or other structural problems**.

**Hunner lesions** are suspected, because finding them can change treatment.

Symptoms persist despite standard treatment, and the bladder has never been evaluated.

The patient is older, especially over 50, since Hunner lesions are more common in that group.

A normal cystoscopy **does not rule out IC/BPS**. Many people with IC/BPS have a bladder that looks normal. Also, older findings like “glomerulations” after bladder stretching are not considered specific enough to diagnose IC by themselves.

So the practical answer is: **no, cystoscopy is not automatically required**, but it can be useful when the case is complicated, there are red flags, or the doctor wants to look for Hunner lesions or rule out other conditions.

**26.Can food or drinks make interstitial cystitis symptoms worse?**

Yes. **Food and drinks can make interstitial cystitis/bladder pain syndrome symptoms worse for some people**, though triggers vary a lot from person to person. Diet is not considered the root cause, but certain items can trigger **flares** such as bladder pain, burning, urgency, or frequency.

Commonly reported triggers include:

| **Category** | **Examples** |
| --- | --- |
| **Acidic drinks/foods** | orange or grapefruit juice, citrus, tomatoes, tomato sauce |
| **Caffeine** | coffee, tea, cola/energy drinks |
| **Alcohol** | wine, beer, spirits |
| **Carbonated drinks** | soda, sparkling water for some people |
| **Spicy foods** | hot peppers, chili, spicy sauces |
| **Sweeteners/additives** | artificial sweeteners, MSG |
| **Other common triggers** | chocolate; sometimes potassium-rich foods for some people |

NIDDK specifically notes that citrus juices, coffee, tea, soda, alcohol, tomatoes, spicy foods, artificial sweeteners, chocolate, and MSG may worsen symptoms in some people with IC. Johns Hopkins lists similar triggers, including citrus, tomatoes, chocolate, coffee, alcohol, caffeine-containing drinks, spicy foods, and some carbonated drinks.

The best way to figure out your own triggers is a **food and symptom diary**: track what you eat/drink, symptoms, timing, stress, menstrual cycle if relevant, sex, exercise, and hydration. Then try removing suspected triggers for a short period and reintroducing one at a time. NIDDK also suggests working with a registered dietitian when possible, especially to avoid an overly restrictive diet.

A useful rule of thumb: **don’t assume every flare is food-related.** IC flares can also be triggered by stress, dehydration, urinary tract infection, holding urine too long, certain irritants, pelvic-floor strain, sex, menstruation, or physical activities.

Seek medical care promptly if you have fever, flank/back pain, blood in urine, new severe burning, or symptoms that feel like a UTI, because infection and other causes can mimic or worsen IC.

**27.Which drinks commonly bother people with interstitial cystitis?**

Common drinks that may bother people with **interstitial cystitis/bladder pain syndrome (IC/BPS)** include:

**Coffee** — regular and sometimes decaf

**Tea** — especially caffeinated black/green tea; some herbal teas can bother certain people too

**Soda and other carbonated drinks**

**Alcohol** — wine, beer, champagne, cocktails

**Citrus juices/drinks** — orange, grapefruit, lemonade

**Cranberry juice** — often acidic, despite its UTI reputation

**Energy drinks** — usually caffeine + acidity + carbonation/sweeteners

**Drinks with artificial sweeteners**

**High–vitamin C drinks** or acidic “immune boost” drinks

A simple way to remember the big triggers is Mayo Clinic’s “**four Cs**”: **carbonation, caffeine, citrus, and high vitamin C**. They also note alcohol and artificial sweeteners can aggravate symptoms in some people. NIDDK similarly lists citrus drinks, coffee, tea, soda, alcohol, and artificial sweeteners as possible flare triggers, while emphasizing that triggers vary by person.

Safer starting options are usually **water**, **low-acid non-citrus drinks**, and possibly **mild herbal teas** that you personally tolerate. The most useful approach is a short **drink diary**: remove likely triggers during a flare, then reintroduce one at a time to see what actually affects you.

**28.Can coffee make interstitial cystitis symptoms worse?**

Yes. **Coffee can make interstitial cystitis/bladder pain syndrome symptoms worse for some people**, mainly because it contains **caffeine** and is often **acidic**, both of which can irritate the bladder. NIDDK lists **coffee, tea, soda, and alcohol** among drinks that may trigger IC symptom flares, and Mayo Clinic includes **caffeine** among common bladder irritants.

That said, IC triggers vary a lot. Some people flare from one cup; others tolerate small amounts, decaf, cold brew, or low-acid coffee; and some do not notice a diet connection at all. Johns Hopkins also lists coffee and caffeinated drinks among foods/drinks that commonly worsen symptoms for many people with IC.

A practical way to test it is to stop coffee for **1–2 weeks**, track bladder pain/urgency/frequency, then reintroduce a small amount and see whether symptoms worsen within the next day. NIDDK specifically recommends using a food diary to identify individual flare triggers.

Possible swaps to try: water, herbal/non-citrus teas, low-acid decaf, or grain-based coffee substitutes. Be careful with “energy” drinks, carbonated drinks, citrus flavors, and artificial sweeteners, since those can also be triggers for some people.

If your symptoms suddenly worsen, you have burning plus fever, blood in urine, flank pain, or a new pattern of urinary symptoms, it is worth checking for a UTI or another cause rather than assuming it is just IC.

**29.Does everyone with interstitial cystitis need to avoid the same foods?**

No. **People with interstitial cystitis/bladder pain syndrome do not all need to avoid the same foods.** Triggers vary a lot from person to person, and some people have few or no food triggers.

That said, certain foods and drinks are *commonly* reported to worsen IC symptoms, especially:

Coffee, tea, soda, alcohol

Citrus juices

Tomatoes and tomato sauces

Spicy foods

Artificial sweeteners

Chocolate

MSG

The NIDDK recommends figuring out your own triggers with a **food and symptom diary**, rather than assuming every “IC diet” food is off-limits forever. They also suggest working with a registered dietitian when possible, especially if restrictions are making eating difficult.

A practical approach is usually:

Start with the most common irritants, not every possible trigger.

Remove one or a small group for a short period.

Reintroduce foods one at a time and watch for flares.

Keep foods that do not bother you.

This matters because IC symptoms themselves vary and can change over time, so a food that bothers one person may be fine for another—or may bother you only during a flare.

So the goal is not “avoid the entire IC list.” The goal is **identify your personal triggers while keeping your diet as broad and nourishing as possible**.

**30.How can someone with interstitial cystitis find out which foods bother their bladder?**

The most reliable way is a **structured elimination-and-rechallenge diet**, not guessing from one bad flare.

A practical approach:

**Start with a symptom + food diary**
Track for a week or two: foods, drinks, portion sizes, symptom timing, pain/urgency/frequency level, stress, sex, menstruation, exercise, constipation, and medications. IC flares can have non-food triggers too.

**Remove common bladder irritants for 1–2 weeks**
Urology guidance commonly recommends an elimination diet for IC/BPS, then slowly adding foods back. Common irritants include **coffee/caffeine, citrus, tomatoes, alcohol, carbonated drinks, spicy foods, chocolate, vinegar/pickled foods, cranberry juice, and artificial sweeteners**.

**Look for a clear baseline improvement**
If symptoms improve during the elimination phase, diet may be contributing. If nothing changes, food may not be a major trigger, or other triggers may be dominating.

**Reintroduce one item at a time**
Add back **one food or drink only**, in a normal serving, then wait **24–72 hours** while tracking symptoms before testing another. The Urology Care Foundation patient guide says that after 1–2 weeks, people can try one food at a time from the IC/BPS food list and keep what does not bother the bladder.

**Confirm suspected triggers**
A food is more likely a real trigger if it causes a similar flare **more than once**, especially when other variables were stable. Don’t label a food “bad forever” after one episode.

**Avoid over-restricting**
The Interstitial Cystitis Association also recommends an elimination diet and food diary, but notes that symptoms may take more than a few days or weeks to settle after removing a problematic food. Working with a dietitian can help prevent unnecessary restriction, especially if the person already has a limited diet.

A simple diary format:

| **Time** | **Food/drink** | **Symptoms before** | **Symptoms 2–6 hrs later** | **Symptoms next day** | **Other factors** |
| --- | --- | --- | --- | --- | --- |
| 8 AM | Coffee | 2/10 | 6/10 urgency | 5/10 pain | poor sleep |

They should also check with a clinician if symptoms are new, worsening, include fever/blood in urine, or could be a UTI, because IC/BPS symptoms can overlap with other conditions.

**31.Should people with interstitial cystitis drink less water to urinate less often?**

Usually, **no — people with interstitial cystitis should not simply drink less water just to urinate less often**.

Cutting fluids too much can make urine more concentrated, which may **burn or irritate the bladder more** and potentially worsen urgency or pain. Current NIDDK guidance says that **staying hydrated may help improve IC symptoms**, while also noting that certain drinks can trigger flares.

A better approach is:

**Drink enough to avoid dehydration**, but do not force huge amounts of water. Sip steadily through the day rather than chugging. Many people do best when their urine is pale yellow rather than very dark.

**Reduce bladder-irritating drinks first**, not water first. Common IC triggers include coffee, tea, soda, alcohol, citrus drinks, artificial sweeteners, and acidic/spicy foods or drinks.

**Track patterns.** If more fluid clearly worsens pressure or pain, or if less fluid causes burning, a bladder diary can help find the “middle zone.” NIDDK specifically recommends using a food/drink diary to identify individual triggers.

**Ask a clinician before restricting fluids**, especially if there is kidney disease, recurrent UTIs, pregnancy, older age, dizziness, very dark urine, or medications that affect fluid balance.

So the practical answer is: **do not intentionally dehydrate to pee less**. Aim for **reasonable hydration**, avoid trigger beverages, and adjust timing/amounts based on symptoms.

**32.Can emotional stress make interstitial cystitis symptoms worse?**

Yes. **Emotional stress can make interstitial cystitis/bladder pain syndrome symptoms worse or trigger a flare** in some people. Mayo Clinic lists **stress** among common flare triggers, along with menstruation, prolonged sitting, exercise, and sexual activity. IC symptoms can also fluctuate over time, even without an obvious trigger.

Stress does not mean the symptoms are “all in your head.” Stress can affect pain sensitivity, muscle tension, sleep, inflammation signaling, and pelvic floor tightness, all of which can worsen bladder urgency, frequency, pressure, or pelvic pain. NIDDK notes that IC symptoms vary, may flare, and can include bladder/pelvic discomfort, urinary frequency, urgency, and pain that worsens as the bladder fills and improves after urinating.

Practical things that may help during stress-related flares include gentle pelvic floor relaxation, heat, hydration, sleep support, avoiding known food/drink triggers, and stress-management approaches like breathing exercises, CBT-style coping tools, or mindfulness. Diet triggers can also contribute to flares for some people, so a symptom diary can help separate stress triggers from food, menstrual, sex-related, or dehydration triggers.

Check with a clinician promptly if symptoms feel different than your usual flare, or if you have fever, back/flank pain, blood in urine, vomiting, pregnancy, new severe pain, or UTI-like symptoms—IC can mimic a UTI, and a UTI can also worsen IC symptoms.

**33.What daily habits may help reduce interstitial cystitis flare-ups?**

Daily habits that may reduce interstitial cystitis/bladder pain syndrome flare-ups include:

**Track your triggers.** Keep a simple bladder diary for 2–4 weeks: food/drinks, stress level, sex, exercise, sleep, period/hormonal timing, symptoms, and urgency/frequency. IC triggers vary, and NIDDK notes that a food diary can help identify specific foods or drinks that cause flares.

**Adjust bladder-irritating foods and drinks.** Common triggers include coffee, tea, soda, alcohol, citrus, tomatoes/tomato sauces, spicy foods, chocolate, MSG, artificial sweeteners, carbonated drinks, and high-vitamin-C foods or supplements. Try reducing—not necessarily permanently banning—suspected triggers, then reintroduce one at a time.

**Stay steadily hydrated.** Dehydration can make urine more concentrated and irritating, but drinking too much at once can worsen urgency. Sip water through the day and ask your clinician what fluid amount fits your health, activity, and climate.

**Use stress downshifting daily.** Stress can worsen symptoms for some people. A few minutes of slow breathing, meditation, gentle stretching, heat, or a calming walk may help reduce nervous-system and pelvic-floor tension.

**Move gently.** Walking, gentle yoga, stretching, or other low-impact activity may help symptoms. Avoid exercises that clearly trigger pelvic/bladder pain, such as intense cycling or heavy core work, unless you’ve built up tolerance.

**Try timed voiding/bladder training if appropriate.** Some people benefit from urinating on a schedule rather than reacting to every urge, then gradually lengthening intervals. Mayo Clinic lists timed urination as a bladder-training strategy, but it is best done gently and stopped if it significantly worsens pain.

**Avoid bladder/pelvic irritants.** Use fragrance-free soaps, detergents, pads, and intimate products. Avoid douches, scented wipes, harsh cleansers, or anything that causes burning.

**Protect sleep and recovery.** Poor sleep can lower pain tolerance and increase stress. A consistent bedtime, reduced evening fluids if nighttime urination is a problem, and a wind-down routine may help.

**Do not smoke.** Smoking can irritate the bladder, and NIDDK specifically recommends quitting as part of IC lifestyle care.

**Consider pelvic floor physical therapy.** If you have pelvic tightness, pain with sex, urethral burning, constipation, or pain after urination, a pelvic floor PT can be very helpful. IC treatment plans may include physical therapy, bladder training, medicines, and other options depending on symptoms.

Seek care promptly if symptoms suddenly change, you have fever, flank/back pain, blood in urine, pregnancy, or burning that feels like a UTI, because IC flares and infections can feel similar.

**34.Can interstitial cystitis be cured completely?**

Interstitial cystitis/bladder pain syndrome **usually is not considered “curable” in the sense of one treatment permanently eliminating it for everyone**. It is generally described as a **chronic, long-lasting condition**, and treatments are aimed at reducing or stopping symptoms rather than guaranteeing a permanent cure.

That said, **many people improve a lot**, and some have long symptom-free or near-symptom-free periods, often called **remission**. Symptoms can also “come and go” and change over time.

The realistic goal is usually:
**identify your triggers + treat the specific contributors to your symptoms + prevent flares as much as possible.** Common approaches include diet changes, stress reduction, bladder training, pelvic-floor physical therapy, medications, bladder instillations, neuromodulation, or procedures for specific findings such as Hunner lesions.

A few important notes:

**If you have Hunner lesions**, treating them can bring major relief for many patients, though repeat treatment may be needed.

**Surgery is rare** and is not a guaranteed cure; even bladder-removal surgery does not always eliminate pain.

Symptoms that seem like IC can overlap with UTIs, pelvic-floor dysfunction, endometriosis, overactive bladder, urethral pain syndrome, or other conditions, so reassessment can matter if symptoms are changing.

So the honest answer is: **a complete permanent cure is not guaranteed and is not the usual framing, but meaningful control or remission is possible for many people.** A urologist or urogynecologist can help tailor treatment based on whether your symptoms are bladder-driven, pelvic-floor-driven, hormone-related, post-UTI, Hunner-lesion type, or mixed.

**35.What is the main goal of interstitial cystitis treatment?**

The main goal of interstitial cystitis/bladder pain syndrome treatment is **to control symptoms and improve quality of life**, especially by reducing bladder/pelvic pain, urinary urgency, urinary frequency, and flare-ups.

It is usually managed as a **chronic condition**, so treatment often focuses on finding a personalized combination of strategies rather than a single cure. The American Urological Association describes the treatment goal as **maximizing symptom control and quality of life while minimizing side effects and treatment burden**.

In practice, that means:

reducing pain and pressure

decreasing urgency and frequent urination

identifying and avoiding flare triggers

improving sleep, daily function, and emotional well-being

stopping treatments that do not help and trying safer options first

Treatment often takes trial and error because no one treatment works for most people with IC/BPS.

**36.How long can interstitial cystitis treatment take to start working?**

Interstitial cystitis/bladder pain syndrome treatment can take **weeks to months** to start working, and it often takes trial and adjustment to find the right combination.

Typical timelines:

| **Treatment type** | **When improvement may start** |
| --- | --- |
| Diet changes / avoiding triggers | Days to a few weeks |
| Stress reduction, bladder training, daily habits | Several weeks |
| Pelvic floor physical therapy | Often several weeks to a few months |
| Oral medicines like amitriptyline or antihistamines | A few weeks to 2–3 months |
| Pentosan polysulfate / Elmiron | **2–4 months** for pain relief; up to **6 months** for urinary frequency improvement |
| Bladder instillations such as DMSO | Often given weekly for **6–8 weeks**, sometimes with maintenance afterward |
| Hydrodistention | Some people improve within **2–4 weeks**, but symptoms may temporarily worsen right after |

Mayo Clinic notes that **no single treatment works for everyone**, and people often need to try different treatments or combinations before finding relief. For Elmiron specifically, it may take **2–4 months** before pain improves and up to **6 months** for urinary frequency to decrease.

A good rule of thumb: unless side effects are a problem, many IC treatments are judged over **6–12 weeks**, while some bladder-lining medicines need **several months**. Contact your clinician sooner if pain is worsening, you develop fever, blood in urine, new back/flank pain, or symptoms feel like a possible UTI.

**37.Why do doctors often try interstitial cystitis treatments step by step?**

Doctors often try interstitial cystitis/bladder pain syndrome treatments step by step because **IC/BPS is not one single, predictable disease with one best treatment**. It is a symptom syndrome with different possible drivers: bladder lining irritation, pelvic floor muscle dysfunction, nerve sensitization, Hunner lesions, allergies/mast-cell involvement, overlapping chronic pain conditions, or other diagnoses that mimic IC.

A stepwise approach helps because:

**Treatments work very differently person to person.**
The AUA guideline emphasizes individualized treatment and measuring baseline pain/urinary symptoms so doctors can tell whether a treatment is actually helping.

**Lower-risk options are usually tried before higher-risk ones.**
Diet changes, bladder training, stress reduction, pelvic floor physical therapy, and oral medicines generally carry less risk than procedures such as bladder instillations, hydrodistention, Botox, neuromodulation, or surgery.

**It helps identify your “phenotype.”**
For example, someone with pelvic floor tenderness may improve most with pelvic floor PT, while someone with Hunner lesions may need cystoscopy-directed treatment. The AUA specifically notes cystoscopy is not routine for uncomplicated cases but should be performed when Hunner lesions are suspected.

**Many treatments take time to judge.**
Some IC treatments may need weeks to months before the benefit is clear, so changing too many things at once can make it hard to know what helped or caused side effects.

**The diagnosis sometimes evolves.**
IC/BPS symptoms overlap with UTI, overactive bladder, pelvic floor dysfunction, endometriosis, vulvodynia, prostatitis/chronic pelvic pain syndrome, stones, and other conditions. The AUA recommends a careful history, exam, and lab testing to document IC/BPS-type symptoms and exclude other causes.

One nuance: newer AUA guidance no longer frames treatment as rigid “first-line through sixth-line” tiers. Instead, it groups treatments into categories and encourages shared decision-making based on symptoms, risks, preferences, and suspected subtype.

So the “step by step” process is mostly about **safety, personalization, and figuring out which mechanism is driving your symptoms**, rather than assuming everyone must follow the same ladder.

**38.Is it safe to take over-the-counter pain medicine for interstitial cystitis?**

Generally, **yes—some over-the-counter pain medicines can be safe for interstitial cystitis/bladder pain syndrome**, but the safest choice depends on your health history, other medications, and how often you need them.

For **mild bladder pain**, NIDDK says clinicians may suggest OTC pain relievers, but also notes that **all medicines, including OTC medicines, can cause side effects**, so it’s best to discuss them with your healthcare professional. Mayo Clinic lists **NSAIDs such as ibuprofen or naproxen** as oral medicines that may help relieve IC pain.

**Common options:**

**Acetaminophen/Tylenol** is often a reasonable first option for pain if you can take it safely. Avoid exceeding the label dose, and be extra cautious if you drink alcohol heavily, have liver disease, or take other products that contain acetaminophen.

**Ibuprofen/Advil/Motrin or naproxen/Aleve** may help some IC flares, especially when inflammation is part of the pain. But NSAIDs are not ideal for frequent or long-term use because they can irritate the stomach, increase bleeding risk, affect kidneys, and may be risky with certain heart conditions, blood thinners, kidney disease, ulcers, uncontrolled high blood pressure, or pregnancy.

**Phenazopyridine/AZO urinary pain relief** may help burning/urinary discomfort short term, but it is usually meant for brief use and can mask infection symptoms. It also turns urine orange. Avoid it or ask a clinician first if you have kidney disease.

You should **contact a clinician promptly** if you have fever, flank/back pain, blood in urine, vomiting, pregnancy, new severe symptoms, or symptoms that feel different from your usual IC flare, because IC can mimic a UTI or other conditions. NIDDK notes IC is diagnosed by ruling out similar conditions, and symptoms can vary over time.

A practical approach: use the **lowest effective dose for the shortest time**, avoid mixing multiple NSAIDs, check combination cold/flu products for hidden acetaminophen, and talk with your urologist or primary care clinician if you need OTC pain medicine often.

**39.Are antibiotics helpful for interstitial cystitis when there is no infection?**

Generally, **no**: antibiotics are **not considered helpful for interstitial cystitis/bladder pain syndrome (IC/BPS) when there is no bacterial infection**.

IC/BPS is usually diagnosed after ruling out infection and other causes. The NIDDK notes that urine culture is used to identify bacteria, and **bacterial UTIs can be treated with antibiotics**—but IC treatment is different and may include lifestyle changes, bladder training, pelvic-floor physical therapy, medications, bladder instillations, neuromodulation, or procedures.

The American Urological Association guideline specifically says **long-term oral antibiotics should not be offered** for IC/BPS. It cites a randomized trial where prolonged sequential antibiotics did **not** significantly outperform placebo, and notes risks such as GI side effects, vaginal infections, nausea/dizziness, and antibiotic resistance.

A few important caveats:

**A true UTI can coexist with IC**, so new/worse symptoms, fever, flank pain, visible blood, pregnancy, recent procedure/catheter, or unusual urine odor/cloudiness should prompt urinalysis and ideally **urine culture**.

A short antibiotic course may be reasonable while awaiting culture **if your clinician strongly suspects infection**, but repeated antibiotics for culture-negative IC flares usually aren’t recommended.

Feeling a little better on antibiotics does **not prove infection**; symptoms can fluctuate, and some antibiotics have non-antibacterial anti-inflammatory effects.

For culture-negative IC symptoms, it is usually better to ask your urologist about an IC flare plan: pelvic-floor assessment/physical therapy, bladder-friendly diet trials, urinary analgesics when appropriate, antihistamines or amitriptyline-type options, bladder instillations, and checking for mimics such as pelvic floor dysfunction, urethral syndrome, stones, STI/vaginal infections, or endometriosis.

**40.Should someone keep taking antibiotics for interstitial cystitis if urine tests show no infection?**

Generally, **no—not long-term or repeatedly—unless a clinician has a specific reason to suspect infection despite the tests**.

Interstitial cystitis/bladder pain syndrome can feel very similar to a UTI, but by definition it involves bladder/pelvic pain symptoms **without an identifiable infection or other clear cause**. Urinalysis and urine culture are commonly used to rule out UTI; bacterial UTIs are what antibiotics treat.

If urine tests/cultures show **no infection**, continuing antibiotics may not help IC itself and can cause harm, including side effects, yeast infections, gut disruption, allergic reactions, and antibiotic resistance. Mayo Clinic notes that IC symptoms may resemble chronic UTI, but “there’s usually no infection.”

Important caveats:

**Do not stop a prescribed antibiotic mid-course without contacting the prescriber**, especially if it was started for a suspected UTI.

Ask whether the test was a **urine culture**, not just a dipstick. Cultures are better for confirming infection.

If symptoms persist with negative cultures, it is reasonable to ask about an **IC/BPS treatment plan** rather than repeated antibiotics: bladder irritant avoidance, pelvic floor physical therapy, pain management, bladder instillations, oral medications, or referral to a urologist/urogynecologist. Mayo Clinic describes IC treatment as trial-and-error because no single treatment works for everyone.

Seek urgent care for **fever, flank/back pain, vomiting, pregnancy, visible blood in urine, worsening illness, or kidney-infection symptoms**.

A good question to ask the clinician is: “Given that my urine culture is negative, what evidence are we treating with antibiotics, and what is the plan for IC/bladder pain management instead?”

**41.Why might eye checks be needed for some interstitial cystitis medicines?**

Eye checks are mainly needed because **pentosan polysulfate sodium** — brand name **Elmiron**, an oral medicine used for interstitial cystitis/bladder pain syndrome — has been linked to **pigmentary maculopathy**, a type of damage to the **macula**, the central part of the retina used for reading and detailed vision.

The concern is that this retinal change can be **subtle at first**, may be missed without special eye imaging, and in some people can **continue or progress even after stopping the medicine**. The FDA label warns patients to report vision changes such as trouble reading, slower adjustment to dim light, or reduced/blurred vision.

The American Urological Association also says clinicians should counsel patients considering pentosan polysulfate about the risk of **macular damage and vision-related injury**.

An eye check may include a dilated retinal exam and retinal imaging such as **OCT**, **fundus autofluorescence**, or retinal photography. These look for early macular changes before vision loss becomes obvious.

This does **not** apply equally to every IC medicine. It is especially associated with **pentosan polysulfate/Elmiron**, not typical short courses of antibiotics, antihistamines, bladder instillations, or pain medicines. A person taking Elmiron should ask their prescriber and an eye doctor how often screening is needed, especially with long-term use or any vision symptoms.

**42.Should people with interstitial cystitis talk to a doctor before using supplements?**

Yes. People with interstitial cystitis/bladder pain syndrome should talk with a doctor, urologist, pharmacist, or other qualified clinician before using supplements.

The main reasons are:

**Supplements can cause side effects or flares.** IC symptoms can be sensitive to acids, herbs, fillers, dyes, and other ingredients.

**They can interact with medications.** This matters especially if someone takes blood thinners, antidepressants, antihistamines, pain medicines, bladder medications, or has kidney/liver issues.

**“Natural” does not mean safe or proven.** Supplements vary in quality and dose, and many claims for IC are not well proven.

**IC symptoms can mimic other conditions.** New or worsening burning, urgency, pelvic pain, blood in urine, fever, or back pain should be medically evaluated rather than self-treated.

NIDDK specifically says that **all medicines, including over-the-counter medicines and supplements, may cause side effects and that people should talk with a health care professional before taking them**. Mayo Clinic also describes IC treatment as individualized, often involving lifestyle changes, physical therapy, oral medications, and other therapies rather than a one-size-fits-all approach.

A practical approach is to bring the supplement name, full ingredient label, dose, and your medication list to a clinician before starting it.

**43.Can pelvic floor physical therapy help interstitial cystitis?**

Yes — **pelvic floor physical therapy can help some people with interstitial cystitis/bladder pain syndrome (IC/BPS)**, especially when pelvic floor muscles are tight, tender, overactive, or have trigger points.

The **American Urological Association** recommends **manual pelvic floor physical therapy** for IC/BPS patients who have pelvic floor tenderness. This includes techniques to release trigger points, lengthen tight muscles, and address painful connective tissue restrictions. Importantly, the guideline says **pelvic floor strengthening exercises like Kegels should be avoided** in this situation because they can worsen symptoms when the pelvic floor is already overactive or tense.

It may help with symptoms such as:

urinary urgency and frequency driven by pelvic floor tension

bladder/pelvic pain or pressure

pain with sex

urethral burning or “UTI-like” symptoms with negative cultures

flares triggered by sitting, stress, or muscle guarding

It is **not a universal cure** for IC/BPS. IC/BPS can involve bladder lining issues, inflammation, nerve sensitization, Hunner lesions, hormonal factors, endometriosis overlap, and other contributors. PT is most likely to help when pelvic floor dysfunction is part of the picture. The AUA notes that no single treatment works for most patients, and symptom control often takes a combination approach.

A good IC-aware pelvic floor PT usually focuses on **down-training and relaxation**, not strengthening. That may include internal or external manual release, breathing work, hip/abdomen work, bladder-friendly stretches, posture/body mechanics, and flare-management strategies.

A practical next step is to ask your urologist, gynecologist, or primary care clinician for a referral to a **pelvic floor physical therapist experienced with IC/BPS or pelvic pain**, and confirm they do **manual therapy/down-training rather than Kegels as the main approach**.

**44.Are pelvic floor strengthening exercises always good for interstitial cystitis?**

No. **Pelvic floor strengthening exercises are not always good for interstitial cystitis/bladder pain syndrome (IC/BPS)**—and in many IC/BPS patients, **Kegels or “strengthening” can make symptoms worse**, especially if the pelvic floor is already tight, tender, or overactive.

The key distinction is:

**Helpful for many IC/BPS patients:** pelvic floor physical therapy focused on **relaxation, down-training, trigger point release, stretching, breathing, and manual therapy**.

**Potentially harmful or flare-triggering:** pelvic floor **strengthening**, especially Kegels, when the pelvic floor is hypertonic/tight.

The American Urological Association’s IC/BPS guideline specifically says that manual pelvic physical therapy should be offered to patients with pelvic floor tenderness, and that **pelvic floor strengthening exercises such as Kegels should be avoided**. Medscape’s guideline summary says the same.

Strengthening may be appropriate later **only if a trained pelvic floor PT finds true weakness and you can fully relax the muscles afterward**. But starting with strengthening on your own can backfire if urgency, burning, pelvic pain, painful sex, tailbone/hip pain, or “can’t empty fully” feelings are coming from muscle guarding.

A safer starting point is usually: diaphragmatic breathing, pelvic floor “drops”/reverse Kegels, gentle hip/pelvic stretches, heat, and evaluation by a pelvic floor PT familiar with IC/BPS. Cleveland Clinic also notes that relaxing the pelvic floor is especially important in IC/BPS care.

So the practical answer is: **do not assume “stronger is better.” For IC, “less guarded and better coordinated” is often the goal first.**

**45.Can bladder training help people with interstitial cystitis urinate less often?**

Yes. **Bladder training can help some people with interstitial cystitis/bladder pain syndrome urinate less often**, especially when urinary frequency and urgency are partly driven by habit, fear of pain, or a “just in case” pattern of voiding.

The NIDDK says bladder training may help the bladder hold more urine, reduce pain and urgency, and help people go to the bathroom less often. Mayo Clinic describes it as **timed urination**: start with a realistic interval, such as every 30 minutes, then gradually lengthen the time between bathroom visits while using relaxation or distraction techniques to manage urges.

A practical approach is to keep a bladder diary for a few days, choose a starting interval you can usually tolerate, and increase it slowly, often by 5–15 minutes every several days or weekly. It should **not** mean forcing yourself to sit in severe pain for long stretches. During flares, after new/worsening symptoms, or if you have burning, fever, blood in urine, or possible UTI symptoms, it is better to check with a clinician rather than push through.

It also is not a stand-alone cure. IC/BPS treatment often requires a combination of strategies, and the American Urological Association notes that no single treatment works for most patients; self-care and behavioral modifications can be helpful, but many people need multiple therapies. Pelvic floor physical therapy may be especially important if pelvic floor tightness or tenderness is contributing; NIDDK specifically cautions that people with IC symptoms should avoid Kegels unless working with a pelvic floor physical therapist.

So: **yes, it can reduce frequency for some people**, but it works best when done gently, gradually, and as part of an IC/BPS plan guided by a urologist or pelvic floor specialist.

**46.Can bladder medicine placed through a tube help interstitial cystitis symptoms?**

Yes. **Bladder medicine placed through a tube — called bladder instillation, bladder wash, or bladder bath — can help some people with interstitial cystitis/bladder pain syndrome (IC/BPS)**, especially with bladder pain, urgency, and flares. It does **not** work for everyone, and the benefit may be temporary.

During an instillation, a clinician passes a small catheter through the urethra into the bladder, puts liquid medicine into the bladder, and you hold it there briefly before urinating it out. NIDDK says several treatments may be needed before symptoms improve.

Common medicines include **DMSO**, **lidocaine**, and **heparin**, sometimes mixed as a “cocktail.” The 2022 American Urological Association guideline says **DMSO, heparin, and/or lidocaine may be used as intravesical treatments** for IC/BPS; the evidence strength varies, and it is listed as an option rather than a guaranteed treatment.

**DMSO** is a classic instillation treatment; Mayo Clinic describes it being placed through a thin catheter, held in the bladder for about 15 minutes, often weekly for 6–8 weeks, then sometimes as maintenance. **Heparin/lidocaine instillations** may give faster “rescue” relief for pain and urgency; AUA cites studies where response improved over repeated treatments but often declined months later.

Downsides can include catheter discomfort, urethral irritation, temporary burning, urinary tract infection risk, cost/insurance issues, and for DMSO a garlic-like breath/body odor. Harvard Health notes DMSO is the only FDA-approved bladder instillation drug for IC, while other instillation mixtures are used but may be less standardized.

So the practical answer is: **yes, it can help IC symptoms, but it is usually a trial treatment**. It may be most useful if symptoms feel bladder-centered or during flares, while people whose symptoms are mainly pelvic floor muscle pain, widespread pain, or nerve pain may need other treatments too. Discuss which medicine is being proposed, how many treatments are planned, what benefit to expect, and what to do if catheterization worsens symptoms.

**47.Is major surgery usually needed for interstitial cystitis?**

Usually, **no**. Major surgery is **rarely needed** for interstitial cystitis/bladder pain syndrome.

Most people are treated with combinations of lifestyle changes, bladder training, pelvic-floor physical therapy, medicines, bladder instillations, hydrodistention, Botox, treatment of Hunner lesions, or neuromodulation—not major surgery. NIDDK notes that surgery is rare and is generally considered only after other options have failed and pain remains severe.

The American Urological Association says major surgery—such as bladder enlargement, urinary diversion, or bladder removal—may be considered only for **carefully selected patients**, especially those with **bladder-centered symptoms** or the rare “end-stage” small fibrotic bladder, after all other therapies fail. It is irreversible, life-changing, and may not cure pain even if the bladder is removed.

So the practical answer is: **major surgery is a last-resort option, not the usual path.** If someone is being told they need major surgery for IC, it is very reasonable to get an opinion from a urologist who specializes in IC/BPS or pelvic pain before deciding.

**48.Can interstitial cystitis affect sexual life?**

Yes. **Interstitial cystitis/bladder pain syndrome can affect sexual life**, and it is a common concern.

It may cause **pain during or after sex**, pelvic/bladder pressure, urethral burning, urgency/frequency afterward, or symptom “flares” triggered by sexual activity. Mayo Clinic lists **pain during sex** among IC symptoms and notes that sexual activity can trigger flares; it also says IC can strain intimacy and relationships.

Helpful steps often include:

**Treating pelvic floor dysfunction** if present. Pelvic floor physical therapy can help, but this usually means relaxation/manual therapy, **not Kegels**, which can worsen tight pelvic floor symptoms.

**Timing sex when symptoms are calmer**, avoiding penetration during flares, and stopping if pain starts.

**Using lubricant**, trying gentler positions, and focusing on non-penetrative intimacy when needed.

**Urinating after sex** and watching for true UTI symptoms, since IC can feel like a UTI but usually does not involve infection.

Discussing options with a **urologist, urogynecologist, or pelvic pain specialist**, especially if sex reliably triggers pain.

Seek medical care promptly if you have fever, blood in urine, new severe pain, unusual discharge, STI risk, pregnancy, or symptoms that feel different from your usual IC pattern.

**49.What can someone do if interstitial cystitis causes pain during sex?**

Yes. Interstitial cystitis/bladder pain syndrome can flare with sexual activity, and painful intercourse is common enough that it should be treated as part of the condition—not something to “push through.” IC can cause pelvic/bladder pain, urgency, frequency, and flares triggered by sex; painful intercourse is specifically noted in IC patient information.

Things that often help:

**Pause sex during a flare.** Sex when the bladder/pelvis is already irritated can worsen symptoms. Resume gradually when baseline pain is lower.

**Empty the bladder before and after sex.** This may reduce bladder pressure and also helps distinguish IC flares from possible UTIs.

**Use plenty of lubricant and avoid irritating products.** Choose a gentle, fragrance-free lubricant. Avoid spermicides, scented products, douches, harsh soaps, and “warming” or flavored lubes, which can irritate the urethra/vulva.

**Try positions that reduce bladder pressure or deep penetration.** Many people do better with slower pace, shallower penetration, side-lying positions, or positions where the person with pain controls depth and angle.

**Use heat, relaxation, or pain-control strategies afterward.** A warm bath, heating pad over the pelvis, gentle stretching, hydration, or clinician-approved pain medicine may help. Mayo Clinic lists NSAIDs and other medications as options for symptom relief, but these should be individualized.

**Ask about pelvic floor physical therapy.** IC often overlaps with pelvic floor muscle tenderness, guarding, or spasm. Pelvic floor PT can help when sex hurts because muscles are tight or reactive; Mayo Clinic notes PT may relieve pelvic pain linked to pelvic-floor muscle or connective-tissue problems.

**Work with a urologist/urogynecologist on IC control.** Options may include diet-trigger management, bladder instillations, oral medications, antihistamines, tricyclics such as amitriptyline, or pentosan polysulfate in selected cases. Treatment usually needs tailoring because IC has no single cure and symptoms vary widely.

**Consider other causes too.** Pain with sex may also come from pelvic floor dysfunction, vulvodynia, vaginal dryness, endometriosis, yeast/BV/STIs, UTI, hormonal changes, or skin conditions. IC can coexist with these, so new or worsening pain deserves evaluation rather than assuming it is “just IC.”

Seek medical care promptly if there is fever, flank/back pain, blood in urine, pregnancy, new discharge/odor, sores, severe burning, or a sudden major change in symptoms—those can point to infection or another issue.

A useful script for a clinician: “I have IC/bladder pain syndrome and sex triggers bladder/pelvic pain. Can we evaluate for pelvic floor dysfunction and infections, and make a flare plan for before and after sex?”

**50.Can interstitial cystitis symptoms get worse after sex?**

Yes. **Interstitial cystitis/bladder pain syndrome symptoms can flare after sex**, and sexual activity is a known trigger for some people. Flares can include more bladder/pelvic pain, burning, urinary urgency, frequency, pressure, or pain during/after sex. Mayo Clinic lists sexual activity among common flare triggers, and NIDDK notes that pain during sex can be related to pelvic floor muscle spasms.

Common reasons this can happen include friction or pressure near the bladder/urethra, pelvic floor muscles tightening or spasming, irritation from condoms/lubricants/spermicides, dehydration, or sex happening during an existing flare. It can also be hard to tell an IC flare from a **UTI or STI**, because symptoms can overlap; the CDC notes IC symptoms can resemble UTI symptoms even when no infection is present.

Things that may help:

Urinate before and after sex.

Use plenty of gentle, fragrance-free lubricant; avoid spermicides if they trigger symptoms.

Try positions with less bladder/pelvic pressure.

Avoid sex during a strong flare when possible.

Apply heat afterward, hydrate, and avoid known bladder irritants like caffeine/alcohol around that time.

Ask a clinician about pelvic floor physical therapy, especially if penetration causes muscle tightness or spasms.

Get checked promptly if symptoms are new, unusually severe, include fever, back/flank pain, blood in urine, unusual discharge, pregnancy, or don’t settle like your typical IC flare, because infection or another condition may need treatment.

**51.What should someone do if interstitial cystitis symptoms worsen during pregnancy?**

If interstitial cystitis/bladder pain syndrome symptoms worsen during pregnancy, the safest next step is to **contact the OB/midwife promptly and ask for a urine test with culture**, even if it “feels like IC.” Pregnancy symptoms, IC flares, and UTIs can overlap, and ACOG recommends evaluating cystitis symptoms in pregnancy with urine culture; UTIs in pregnancy need treatment because they can progress and are linked with complications such as preterm delivery and low birth weight.

**What to do now:**

**Rule out UTI or kidney infection**
Burning, urgency, frequency, bladder pain, blood in urine, or new worsening symptoms should be checked. Seek urgent care now for **fever ≥100.4°F / 38°C, chills, flank/back pain, nausea/vomiting, feeling very ill, contractions, decreased fetal movement, or inability to urinate**. ACOG says suspected pyelonephritis in pregnancy should be managed initially inpatient.

**Do not start, stop, or restart IC medications without OB/urology guidance**
Some bladder pain treatments, antihistamines, bladder instillations, urinary analgesics, pain medicines, supplements, and antibiotics may or may not be appropriate depending on trimester and situation. Ask the OB to coordinate with a urologist/urogynecologist.

**Use low-risk flare measures while waiting**
Hydrate steadily, avoid known triggers such as acidic/spicy foods or caffeine, use heat or cold packs externally, avoid constipation, and consider pelvic-floor relaxation strategies. IC symptoms can flare with stress, sitting, exercise, sex, and other triggers, and symptoms often vary over time.

**Ask for pelvic floor physical therapy**
Pregnancy can worsen pelvic pressure and pelvic-floor tension, which can mimic or amplify IC symptoms. A pelvic-floor PT familiar with pregnancy and bladder pain can be helpful.

**Push for follow-up if culture is negative but symptoms are severe**
A negative culture may support an IC flare, but severe pain, urinary retention, visible blood, vulvar/labial swelling, or new neurologic symptoms still needs medical review.

A practical message to the OB could be: “I have IC/bladder pain syndrome and my urinary pain/urgency has worsened during pregnancy. Can we do a urinalysis and urine culture, and can you advise which IC flare treatments are safe for my trimester or refer me to urology/urogynecology?”

**52.What can someone do if interstitial cystitis causes anxiety?**

Interstitial cystitis/bladder pain syndrome can create a **pain–urgency–fear loop**: symptoms spike, anxiety rises, the pelvic floor and nervous system tense up, and that tension can worsen bladder sensations. That does **not** mean “it’s all in your head.” IC/BPS is a real chronic pain condition, and anxiety is a common, understandable response to it. A systematic review found anxiety and depression scores are often elevated in IC/BPS populations.

Helpful steps:

**1. Treat the IC flare and the anxiety together.**
The American Urological Association recommends multimodal pain management for IC/BPS, including stress management and, when appropriate, manual pelvic-floor physical therapy. It also recommends stress-management practices to help with stress-triggered symptom flares.

**2. Use calming skills during bladder panic.**
Try slow breathing, grounding, progressive muscle relaxation, a heat pack, a warm bath, gentle stretching, or a short walk. The goal is not to “think the pain away,” but to tell the nervous system that the body is not in immediate danger. NIDDK specifically lists relaxation techniques, deep breathing, gentle activity, and support systems as ways that may help people cope with IC symptoms.

**3. Get checked for pelvic-floor tension.**
Anxiety often makes people clench the pelvic floor without noticing. A pelvic-floor physical therapist can assess this and teach down-training/relaxation. Avoid doing Kegels unless a pelvic-floor clinician specifically recommends them; NIDDK notes that people with IC symptoms should avoid pelvic-floor strengthening exercises unless working with a physical therapist.

**4. Track patterns without obsessing.**
A brief bladder diary can help identify triggers: foods, drinks, stress, sleep, sex, constipation, menstrual cycle, medications, or dehydration. NIDDK says clinicians may use a voiding log/bladder diary and symptom scales to understand symptoms and treatment response.

**5. Consider mental-health treatment as part of pain care.**
CBT, acceptance and commitment therapy, mindfulness-based therapy, or trauma-informed therapy can help reduce fear, catastrophizing, sleep disruption, and avoidance. This is not “because IC is psychological”; it is because chronic pain and anxiety reinforce each other.

**6. Ask a clinician about medication options carefully.**
Some medications used in IC or anxiety can help some people and bother others, so this should be individualized—especially if pregnant, trying to conceive, breastfeeding, or medication-sensitive. A urologist, urogynecologist, pelvic-pain specialist, or primary care clinician can help balance IC treatments with anxiety treatment.

Seek urgent help if anxiety becomes overwhelming, you feel unsafe, or you have thoughts of self-harm. Also seek medical care promptly for fever, flank/back pain, blood in urine, inability to urinate, pregnancy with urinary symptoms, or symptoms that feel like a possible UTI.

**53.Can counseling help people cope with long-term interstitial cystitis pain?**

Yes. Counseling can help many people cope with long-term interstitial cystitis/bladder pain syndrome pain, though it usually **does not “cure” IC by itself**. It is best viewed as part of a multimodal pain plan.

The American Urological Association guideline recommends **multimodal pain management**, including stress management, and says patients should be encouraged to use stress-management practices to improve coping and manage stress-related symptom flares. It also notes IC/BPS is typically chronic, with flares and remissions, and often requires multiple combined approaches.

Counseling may help by:

reducing fear, panic, helplessness, and depression around chronic pain

teaching flare-management skills and pacing so life feels less controlled by symptoms

addressing the stress–pain–muscle tension cycle, which can worsen pelvic/bladder symptoms

improving sleep, relationships, work/school coping, and quality of life

helping with grief, isolation, or trauma that can come with long-term pain

The most relevant types are often **CBT for chronic pain**, **ACT**, mindfulness-based approaches, or therapy with a clinician who understands chronic illness, chronic pelvic pain, or pain psychology. Mayo Clinic also notes that IC has no single treatment that works for everyone, may need combinations of treatments, and that support groups can provide listening and useful information.

A good framing is: counseling may not make bladder pain disappear, but it can make pain **less overwhelming, less life-limiting, and easier to manage during flares**. It is especially worth considering if IC has led to anxiety, depression, avoidance, relationship strain, sleep problems, or feeling constantly on alert.

It should ideally be combined with medical care from a urologist/urogynecologist and, when appropriate, pelvic floor physical therapy, diet/bladder-trigger work, medications, and pain-management support. Mayo Clinic notes IC symptoms can flare with triggers including stress, sitting, exercise, menstruation, and sexual activity, so tracking patterns can also be useful.

**54.How can interstitial cystitis affect work, sleep, and daily life?**

Interstitial cystitis/bladder pain syndrome can affect daily life in several overlapping ways:

**Work:** Frequent urgency, pain, and needing to urinate often can make it hard to sit through meetings, stay on a phone shift, commute, travel, or focus for long periods. Some people need flexible bathroom access, remote work, modified schedules, extra breaks, or flare-up leave. IC symptoms can flare with stress, prolonged sitting, exercise, sex, or other triggers, so reliability at work may vary day to day.

**Sleep:** IC can cause nighttime urination and pain as the bladder fills, which may repeatedly wake someone up. Poor sleep can then worsen fatigue, concentration, mood, pain tolerance, and coping the next day. Mayo Clinic notes that frequent urination can occur “throughout the day and night,” and NIDDK describes bladder pain that worsens until urination and then improves temporarily.

**Daily life:** The condition can interfere with errands, social plans, exercise, intimacy, eating/drinking choices, and travel because people may need to plan around bathrooms, avoid symptom triggers, or rest during flares. Pelvic pain, pressure, urgency, and painful sex can also affect relationships and emotional well-being. The National Kidney Foundation notes that work, sexual activity, and normal social functioning can become difficult or even impossible for some people.

**Emotional impact:** Because IC is chronic, unpredictable, and sometimes invisible to others, it can contribute to stress, anxiety, low mood, isolation, and frustration. That does not mean symptoms are “just psychological”; rather, pain, sleep disruption, and lifestyle limits can understandably affect mental health. CDC describes IC as a chronic condition that can last for years or be lifelong.

A helpful way to summarize it is: IC can make everyday life feel organized around pain control, bathroom access, sleep recovery, and avoiding flares. Treatment, pelvic floor physical therapy, lifestyle changes, medication, and counseling/support can often reduce the burden, even when symptoms do not disappear completely.

**55.How should interstitial cystitis be managed over the long term?**

Long-term management of **interstitial cystitis / bladder pain syndrome (IC/BPS)** is usually a **stepwise, individualized plan**: confirm the diagnosis, track symptoms, reduce triggers, treat pelvic-floor and pain contributors, then escalate only when needed. IC/BPS often fluctuates, so the goal is durable symptom control and quality of life rather than a one-time cure.

**1. Reconfirm the diagnosis and track a baseline**

IC/BPS is diagnosed mainly by symptoms and by **ruling out other causes** such as UTI, stones, malignancy, gynecologic causes, overactive bladder, pelvic-floor dysfunction, endometriosis, urethral pain syndromes, or prostatitis-like syndromes. NIDDK notes that clinicians use history, physical exam, lab testing, urinalysis/urine culture, and sometimes cystoscopy or urodynamics to rule out other conditions.

For long-term care, it helps to keep a **bladder diary** for a few weeks: voiding frequency, pain score, urgency, fluid intake, foods/drinks, sex, exercise, menstrual cycle if relevant, stress, constipation, and flares. The AUA guideline emphasizes documenting baseline pain and voiding symptoms so treatment response can be measured.

**2. Build the foundation: lifestyle, flare prevention, and pacing**

The core long-term strategy is identifying personal triggers, not following a rigid universal diet. Common aggravators include coffee/caffeine, alcohol, citrus, carbonated drinks, spicy foods, artificial sweeteners, acidic foods, dehydration, constipation, stress, and some sexual activity, but triggers vary. NIDDK recommends food/fluid adjustment, hydration, smoking cessation, gentle activity, stress reduction, and support systems as part of care.

A practical approach is an **elimination-and-rechallenge** plan: remove likely triggers for 2–4 weeks, then reintroduce one at a time. Avoid over-restricting long term unless a trigger is clear.

**3. Pelvic-floor physical therapy is often central**

Many people with IC/BPS have pelvic-floor muscle tenderness, guarding, trigger points, or pain after urination or sex. In that case, **pelvic-floor physical therapy focused on relaxation, trigger-point release, breathing, mobility, and down-training** can be very helpful. NIDDK specifically notes that a pelvic-floor specialist can help stretch tight pelvic-floor muscles and keep them relaxed, and warns against Kegels unless guided by a therapist.

This distinction matters: IC/BPS pelvic-floor treatment is usually **not strengthening first**; it is usually relaxation and pain modulation first.

**4. Use medications selectively and reassess them**

Medication choices depend on the dominant symptoms: pain, urgency/frequency, sleep disruption, allergy-type flares, neuropathic pain, or severe episodic flares. Options used in practice may include NSAIDs or acetaminophen for short-term pain, urinary analgesics for brief flares, antihistamines, tricyclics such as amitriptyline, neuropathic pain agents in selected patients, and pentosan polysulfate sodium. Mayo Clinic lists NSAIDs, tricyclic antidepressants, antihistamines, and pentosan polysulfate among oral options.

For **pentosan polysulfate sodium / Elmiron**, discuss eye-risk monitoring carefully. The U.S. drug label warns of retinal pigmentary changes, especially with long-term use, recommends an ophthalmologic history before starting, suggests baseline retinal exam within 6 months of starting and periodic exams during treatment, and says changes may be irreversible or progress after stopping.

**5. Treat flares with a written plan**

A long-term plan should include what to do during flares, such as: temporarily simplifying diet, increasing tolerated fluids, heat or cold packs, pelvic-floor relaxation, avoiding intense exercise or painful sex during flare, short-term urinary analgesics if appropriate, and checking urine culture when symptoms are infection-like or different from usual. Do not assume every flare is IC; new burning, fever, flank pain, blood in urine, pregnancy, or unusual symptoms should prompt medical evaluation.

**6. Escalate when conservative care is not enough**

If lifestyle measures, pelvic-floor therapy, and oral medications do not provide adequate control, urology may consider **bladder instillations**, **hydrodistention**, **neuromodulation**, or **botulinum toxin injections** in selected cases. NIDDK describes these as options when other treatments have not worked, with repeated treatments sometimes needed.

If **Hunner lesions** are present, management is different: cystoscopic treatment such as fulguration/electrocautery or injection therapy can be very effective for some patients. NIDDK notes electrocauterization can relieve symptoms from Hunner lesions and may need repeating.

**7. Avoid treatments that cause harm or don’t help**

Long-term antibiotics should generally be avoided unless there is documented infection. The AUA guideline approach is to periodically reassess effectiveness, stop ineffective treatments, use multimodal pain management, and reconsider the diagnosis if multiple reasonable approaches do not help.

Major bladder surgery is rare and reserved for severe refractory cases; NIDDK notes surgery has significant risks and does not always cure pain.

**A reasonable long-term care plan**

A good plan usually looks like this: urologist-confirmed diagnosis, bladder diary, trigger management without excessive restriction, pelvic-floor assessment, constipation and stress/sleep management, a flare protocol, carefully chosen meds with side-effect monitoring, periodic reassessment, and escalation only for persistent symptoms or Hunner lesions.

Seek urgent care for fever, flank pain, inability to urinate, gross blood in urine, pregnancy with urinary symptoms, or a sudden major change from your usual IC pattern.

**56.Should people with interstitial cystitis keep following their care plan after symptoms improve?**

Yes. **People with interstitial cystitis/bladder pain syndrome should usually keep following the parts of their care plan that prevent flares, even after symptoms improve**, but they should review the plan with their clinician to see what can be safely reduced.

IC often has a relapsing pattern: symptoms can improve or go into remission, but flares may return. The CDC describes IC treatment as individualized and often including lifestyle changes, stress management, bladder retraining, physical therapy, and other therapies; NIDDK also notes that clinicians may track symptoms over time to see how treatment is working.

A practical approach:

**Keep doing the low-risk maintenance steps** that helped, such as avoiding known trigger foods/drinks, managing stress, pacing bladder irritants, using bladder-training habits, and continuing pelvic-floor relaxation exercises if those were prescribed.

**Do not stop prescription treatments suddenly** without asking the prescriber. Some medicines may need tapering, and some have monitoring considerations. For example, pentosan polysulfate sodium can take months to work and has been associated with macular eye disease in some people, so follow-up and monitoring matter.

**Ask about stepping down if you have been stable.** A clinician may suggest reducing certain medicines, spacing out treatments, or switching to “flare-only” strategies while maintaining lifestyle measures.

**Restart early flare measures promptly** if symptoms begin returning. Cleveland Clinic specifically lists maintaining treatment even after symptoms go away and using medicines early when a flare starts as self-care strategies for IC/BPS.

Seek reassessment sooner if symptoms change, blood appears in urine, fever develops, pain becomes severe, or symptoms feel different from usual IC, because infections and other conditions can mimic or overlap with IC.

**57.Should online claims about a complete cure for interstitial cystitis be trusted?**

No—**claims of a “complete cure” for interstitial cystitis/bladder pain syndrome should be treated with strong skepticism**, especially if they come from a supplement seller, clinic, influencer, testimonial page, or paid program.

Major medical sources describe IC/BPS as a chronic condition where treatment aims to **reduce symptoms and improve quality of life**, not reliably “cure” everyone. Mayo Clinic states that “no simple treatment eliminates” IC symptoms and that no single treatment works for everyone; it lists options such as pelvic floor physical therapy, oral medications, nerve stimulation, bladder instillations, bladder distention, and lifestyle changes. Mayo also notes that although there is no cure, medications and other therapies may offer relief.

Be especially wary of claims using phrases like **“guaranteed cure,” “root cause cure,” “works for everyone,” “detox,” “hidden infection protocol,” “doctors don’t want you to know,”** or relying mainly on testimonials. The FDA warns that health products promoted to treat or cure diseases without scientific proof can be fraudulent, and that “miracle cure” and “guaranteed results” claims are classic warning signs. The FDA also notes that health-fraud scams can waste money, delay proper diagnosis/treatment, and sometimes cause serious harm.

A more trustworthy claim would sound like: **“This may help some patients with certain symptoms, based on limited/moderate evidence, and should be discussed with a urologist/urogynecologist.”** IC can overlap with UTIs, pelvic floor dysfunction, endometriosis, vulvodynia, overactive bladder, urethral pain syndrome, and other conditions, so personalized evaluation matters.

A good rule: **do not pay for or start a “cure” protocol without asking your clinician what evidence supports it, what risks it carries, and whether it could delay better-established care.**

**58.Is it safe to diagnose or treat interstitial cystitis only with advice from the internet?**

No. It is **not safe to diagnose or treat interstitial cystitis/bladder pain syndrome only with internet advice**.

IC/BPS is usually diagnosed by **ruling out other conditions with similar symptoms**, using medical history, physical exam, urine testing, and sometimes other tests. Conditions such as UTI, kidney stones, pelvic floor dysfunction, endometriosis, sexually transmitted infections, medication irritation, and—more rarely—bladder cancer can overlap with IC symptoms. NIDDK says clinicians diagnose IC using history, physical exam, and lab/other tests to exclude similar conditions.

Internet information can be useful for learning, tracking symptoms, preparing questions, or finding support, but it cannot safely replace a clinician’s evaluation. Even treatment is individualized: NIDDK notes that care may involve lifestyle changes, bladder training, physical therapy, medicines, neuromodulation, bladder procedures, or surgery, and patients may need to try several approaches. Mayo Clinic likewise states that no single treatment works for everyone and that multiple treatments or combinations may be needed.

Be especially cautious with online claims of a “complete cure,” secret protocol, supplement stack, or one-size-fits-all diet. IC symptoms vary, triggers differ, and some treatments can cause side effects or delay needed care. NIDDK specifically advises talking with a health care professional before taking medicines or supplements, even over-the-counter ones.

Seek medical care promptly if there is blood in urine, fever, flank/back pain, pregnancy, new severe pelvic pain, inability to urinate, recurrent “UTIs” with negative cultures, unexplained weight loss, or symptoms that are new or worsening.

A safer approach is: use reputable sources to educate yourself, keep a bladder diary, note food/symptom triggers, and work with a urologist, urogynecologist, pelvic pain specialist, or pelvic floor physical therapist for diagnosis and treatment.

**Copilot**

**1.What is interstitial cystitis?**

**Interstitial cystitis (IC), also called bladder pain syndrome, is a chronic condition that causes bladder pain, pressure, and frequent urination without infection. It mainly affects women between ages 20–50 and can significantly impact quality of life.**

**What It Is**

**Definition:** A long-term bladder disorder where the bladder wall becomes irritated or inflamed, leading to pain and urinary urgency/frequency.

**Key Difference from UTI:** Symptoms resemble a urinary tract infection, but **no infection is present** and antibiotics do not help.

**Other Names:** Painful bladder syndrome, frequency-urgency-dysuria syndrome.

Harvard Health

Credible health information source

**Interstitial cystitis**

Interstitial cystitis is a puzzling bladder condition in which the bladder wall becomes irritated or inflamed, causing pain and frequent or painful urination. The symptoms of interstitial cystitis are often similar to the symptoms of a urinary tract infection. However, in interstitial cystitis, there is no infection, and the symptoms do not respond to antibiotic treatment. The exact cause of interstitial cystitis remains a mystery, although researchers continue to investigate possible causes, such as unidentified bacteria, an allergic or immune system reaction, a toxic substance in the urine, or a neurological problem in the bladder wall. There also is some evidence that interstitial cystitis may not be just one illness, but several illnesses that share similar symptoms. Interstitial cystitis usually occurs between the ages of 20 and 50. Approximately 90% of people with interstitial cystitis are women. It is unknown why interstitial cystitis is more common in women. The disease is not known to be genetic (inherited) or caused by toxins in the environment.

**️ Symptoms**

**Pelvic or bladder pain** (mild to severe)

**Urgent need to urinate** frequently (sometimes up to 60 times/day)

**Small urine volumes** each time

**Pain during sex**

**Flare-ups** triggered by menstruation, stress, sitting long, or certain foods/drinks

**Causes & Risk Factors**

**Exact cause unknown** — possible factors include:

Defects in bladder lining (epithelium leak)

Autoimmune reaction or allergies

Neurological signaling problems

Abnormal substances in urine

**Risk factors:**

Sex: ~90% of cases occur in women

Age: Most diagnosed in their 30s–50s

Associated with other chronic pain disorders (e.g., fibromyalgia, IBS)

**Diagnosis**

**No single definitive test**

Doctors rule out infections, bladder cancer, or other causes using:

**Urinalysis** (usually normal)

**Cystoscopy** (to check bladder lining, ulcers, or hemorrhages)

**Biopsy** (to exclude cancer)

**Treatment & Management**

There is **no cure**, but treatments aim to relieve symptoms:

**Lifestyle & Diet:** Avoid acidic foods, caffeine, alcohol, chocolate, and artificial sweeteners.

**Physical Therapy:** Pelvic floor relaxation exercises.

**Bladder Retraining:** Gradually increase time between urination.

**Medications:**

Oral: Elmiron (FDA-approved), tricyclic antidepressants (pain relief), antihistamines.

Bladder instillations: DMSO or medication “cocktails” delivered directly into bladder.

**Other Therapies:** Stress reduction, nerve stimulation (TENS), or surgery in severe cases.

**Quick Comparison**

| **Aspect** | **Interstitial Cystitis** | **Urinary Tract Infection (UTI)** |
| --- | --- | --- |
| Cause | Unknown, non-infectious | Bacterial infection |
| Symptoms | Pain, urgency, frequency, small volumes | Pain, burning, urgency, fever possible |
| Response to Antibiotics | **No improvement** | Symptoms improve |
| Gender Prevalence | Mostly women (90%) | Both sexes, more common in women |
| Chronicity | Long-term, recurrent | Usually short-term, treatable |

**Key Takeaway**

Interstitial cystitis is a **chronic, non-infectious bladder pain condition** that requires symptom management rather than cure. If you experience persistent bladder pain or frequent urination without infection, consult a urologist for evaluation.

**2.Is interstitial cystitis the same as bladder pain syndrome?**

**Short answer:They are closely related but not identical.***Bladder pain syndrome (BPS)* is a broad clinical term for chronic bladder pain and urinary symptoms; *interstitial cystitis (IC)* is a specific diagnosis within that spectrum—often used when objective bladder findings (especially Hunner lesions) or inflammatory changes are present.

**Definitions**

**Bladder Pain Syndrome (BPS)** — a symptom-based diagnosis describing **chronic pelvic/bladder pain, urinary frequency, and urgency** not explained by infection or other obvious causes.

**Interstitial Cystitis (IC)** — historically used term; in modern practice it often denotes the **subset of BPS with bladder inflammation or characteristic lesions (Hunner lesions)** seen on cystoscopy and biopsy.

**Key differences (practical)**

**Scope:** BPS = **umbrella term**; IC = **specific subtype** within that umbrella.

**Objective findings:** IC may show **Hunner lesions, epithelial damage, or inflammatory infiltrates** on cystoscopy/biopsy; many BPS patients have a normal-looking bladder and are diagnosed clinically.

**Associated conditions:** Both can coexist with **fibromyalgia, IBS, chronic fatigue**, and other pain syndromes; IC with Hunner lesions is more likely to show immune/inflammatory markers.

**What this means for diagnosis and treatment**

**Diagnosis:** Doctors usually **rule out infection and other causes**, take a symptom history, and may perform urine tests, bladder diaries, cystoscopy, and sometimes biopsy. **Cystoscopy** helps distinguish IC (with lesions) from non‑lesion BPS.

**Treatment:** Many treatments overlap (dietary changes, pelvic physiotherapy, oral meds, bladder instillations, neuromodulation, Botox). **Patients with Hunner lesions** may benefit from lesion‑directed therapies (fulguration/laser) and may respond differently to immunomodulatory treatments.

**Practical advice (Singapore context)**

If you’re in **Singapore** and have chronic bladder pain or urinary urgency, tell your clinician you’re concerned about **BPS/IC**; ask whether **cystoscopy** is indicated to check for Hunner lesions because that finding can change management. Local urology clinics and tertiary hospitals follow similar diagnostic pathways.

**Quick takeaways**

**Use the terms carefully:** many clinicians use **BPS and IC interchangeably**, but **IC more specifically implies bladder inflammation or lesions**.

**If symptoms are severe or persistent, see a urologist** for targeted testing and a tailored treatment plan.

**3.Is interstitial cystitis the same as a bladder infection?**

**No — interstitial cystitis (IC) is *not* the same as a bladder infection.** While both cause bladder pain, urgency, and frequent urination, IC is a chronic inflammatory condition without bacterial infection, whereas a bladder infection (cystitis) is caused by bacteria and usually responds to antibiotics.

**Key Differences Between Interstitial Cystitis and Bladder Infection**

| **Feature** | **Interstitial cystitis** | **Bladder infection** |
| --- | --- | --- |
| **Cause** | Unknown; may involve bladder lining defects, autoimmune reactions, or nerve dysfunction | Bacterial growth (commonly *E. coli*) in the bladder |
| **Nature** | Chronic, non-infectious inflammation | Acute bacterial infection |
| **Symptoms** | Bladder/pelvic pain, urgency, frequent urination (up to 60 times/day), pain during sex | Burning with urination, urgency, cloudy or foul-smelling urine, sometimes fever |
| **Response to antibiotics** | Does *not* improve with antibiotics | Usually improves quickly with antibiotics |
| **Diagnosis** | Exclusion of infection; cystoscopy may show bladder wall changes | Urinalysis and urine culture confirm bacteria |
| **Treatment** | Lifestyle changes, bladder retraining, medications (e.g., Elmiron, antihistamines, antidepressants), physical therapy | Antibiotics, hydration, pain relief |
| **Prevalence** | 3–8 million women and 1–4 million men in the U.S. | Up to 50% of women experience at least one UTI in their lifetime |

**️ Why They’re Often Confused**

**Symptom overlap:** Both cause urgency, frequency, and bladder pain.

**Misdiagnosis risk:** IC patients are sometimes treated repeatedly with antibiotics, which don’t help because no infection is present.

**Chronic vs. acute:** IC is long-term and can flare with triggers (stress, menstruation, certain foods), while bladder infections are usually short-term and curable.

**Important Considerations**

**Interstitial cystitis is a chronic pain condition** that can severely affect quality of life, but it is not life-threatening and does not lead to cancer.

**Bladder infections can spread to the kidneys** if untreated, which can become serious.

Anyone with persistent bladder pain or urinary symptoms should **see a healthcare professional** to distinguish between IC and infection.

**Visualizing the Difference**

Would you like me to go deeper into **symptom management strategies for IC** or **prevention tips for bladder infections**?

**4.Can interstitial cystitis happen even when there is no infection?**

**Yes — interstitial cystitis (IC), also called bladder pain syndrome, can occur even when there is *no infection*.** It is a chronic condition where the bladder wall becomes irritated or inflamed, leading to pain, urgency, and frequent urination, but urine cultures are typically sterile and symptoms do not improve with antibiotics.

Harvard Health

Credible health information source

**Interstitial cystitis**

Interstitial cystitis is a puzzling bladder condition in which the bladder wall becomes irritated or inflamed, causing pain and frequent or painful urination. The symptoms of interstitial cystitis are often similar to the symptoms of a urinary tract infection. However, in interstitial cystitis, there is no infection, and the symptoms do not respond to antibiotic treatment. The exact cause of interstitial cystitis remains a mystery, although researchers continue to investigate possible causes, such as unidentified bacteria, an allergic or immune system reaction, a toxic substance in the urine, or a neurological problem in the bladder wall. There also is some evidence that interstitial cystitis may not be just one illness, but several illnesses that share similar symptoms. Interstitial cystitis usually occurs between the ages of 20 and 50. Approximately 90% of people with interstitial cystitis are women. It is unknown why interstitial cystitis is more common in women. The disease is not known to be genetic (inherited) or caused by toxins in the environment.

**Key Facts About Interstitial Cystitis**

**Not caused by infection:** Unlike urinary tract infections (UTIs), IC symptoms persist despite negative urine cultures and antibiotic treatment.

**Symptoms:** Pelvic/bladder pain, urinary urgency, frequency (sometimes up to 60 times/day), pain during sex, and relief after urination.

**Triggers:** Stress, menstruation, prolonged sitting, certain foods/drinks (e.g., caffeine, alcohol, citrus, tomatoes).

**Who is affected:** Most common in women aged 20–50; about 3–8 million women and 1–4 million men in the U.S. are affected.

**Possible Causes (Still Unclear)**

Researchers believe multiple factors may contribute:

**Bladder lining defects:** Leaks in the protective epithelium may allow urine irritants to inflame the bladder wall.

**Immune system involvement:** Autoimmune reactions or allergies may play a role.

**Neurological dysfunction:** Abnormal nerve signaling may cause exaggerated pain/urgency.

**Unidentified infections:** Some researchers suspect unusual microbes not detected by standard tests.

**️ IC vs. UTI: Key Differences**

| **Feature** | **Interstitial Cystitis** | **Urinary Tract Infection** |
| --- | --- | --- |
| Cause | Non-infectious bladder inflammation | Bacterial infection |
| Urine culture | Sterile (no bacteria) | Positive for bacteria |
| Response to antibiotics | No improvement | Symptoms improve |
| Symptoms | Chronic pelvic/bladder pain, urgency, frequency | Burning urination, fever, cloudy urine |
| Course | Long-term, flares and remissions | Short-term, resolves with treatment |

**️ Risks & Considerations**

**Quality of life impact:** Frequent urination and chronic pain can disrupt work, social life, and intimacy.

**Emotional health:** Chronic pain and sleep disruption may lead to stress or depression.

**Complications:** Reduced bladder capacity over time due to stiffening of bladder walls.

**What To Do If You Suspect IC**

**Consult a urologist:** Diagnosis requires ruling out infections, bladder cancer, and other causes.

**Symptom diary:** Track triggers (foods, drinks, stress) to identify flare patterns.

**Treatment options:** Lifestyle changes, bladder retraining, pelvic floor therapy, oral medications (e.g., amitriptyline, antihistamines), bladder instillations, or nerve stimulation.

**5.Is interstitial cystitis a type of bladder cancer?**

**No — interstitial cystitis (IC), also called bladder pain syndrome, is a chronic inflammatory condition of the bladder lining, not a form of bladder cancer; however, some studies report a small association between IC and later urinary tract cancers, so persistent or changing symptoms should prompt urology evaluation.**

**What interstitial cystitis is**

**Definition:IC is a chronic, non‑infectious inflammatory disorder** that causes bladder pain, urinary urgency, and frequency.

**Cause:** The exact cause is unknown; theories include autoimmune, nerve dysfunction, and epithelial (lining) defects. **IC is not caused by malignant (cancerous) cells.**

**How IC differs from bladder cancer**

| **Feature** | **Interstitial Cystitis** | **Bladder Cancer** |
| --- | --- | --- |
| **Nature** | Chronic inflammation/pain disorder | Malignant growth of bladder cells |
| **Typical symptoms** | Pain with bladder filling, frequency, urgency | Painless visible blood in urine (common); later pain/voiding changes |
| **Diagnosis tests** | Symptom-based, cystoscopy, exclusion of infection | Cystoscopy with biopsy; imaging; urine cytology |
| **Treatment** | Pain control, bladder instillations, behavioral changes | Surgery, intravesical therapy, chemotherapy, immunotherapy |

**Evidence on cancer risk**

**Major cancer centers state there is no proven causal link between IC and bladder cancer.**

**Population studies have reported a modestly increased incidence of urinary tract cancers among people previously coded with bladder pain syndrome/IC,** but these findings may reflect diagnostic overlap, surveillance bias, or confounding factors rather than a direct causal relationship. **The absolute risk remains low.**

**Practical guidance and red flags**

**See a urologist if you have:** new or worsening symptoms, visible blood in urine, unexplained weight loss, or a change in symptom pattern. **Visible blood in urine is a key sign that needs urgent evaluation** because it can indicate cancer or other serious conditions.

**If you are in Singapore:** a major public referral point is **SGH Urology Centre — phone: 65 6321 4377; address: Singapore General Hospital, Block 4 Level 1.** Use a local urologist for assessment and cystoscopy if indicated.

**What to expect at evaluation**

**Urine tests** to rule out infection and look for blood or malignant cells.

**Cystoscopy** (camera inspection of the bladder) and **biopsy** if suspicious lesions are seen.

**Imaging** only if indicated by symptoms or findings.

**Bottom line**

**IC is not bladder cancer.** Persistent hematuria or new, atypical symptoms require prompt urology assessment to exclude cancer or other causes.

If you’d like, I can summarize the typical tests and treatments for IC or list urology clinics in central Singapore with contact details.

**6.Is interstitial cystitis caused by stress or anxiety?**

**Stress and anxiety do not *cause* interstitial cystitis (IC), but they are well-known triggers that can worsen symptoms or flare-ups. The exact cause of IC remains unclear, with research pointing to bladder lining defects, autoimmune factors, allergies, or abnormal immune responses.**

**What Causes Interstitial Cystitis?**

**Unknown exact cause**: No single definitive cause has been identified.

**Possible contributing factors**:

**Bladder lining defects**: A “leaky” bladder lining may allow irritating substances in urine to inflame the bladder wall.

**Autoimmune reaction**: The body’s immune system may mistakenly attack bladder tissue.

**Allergies or mast cell activation**: Abnormal immune cell activity may play a role.

**Genetic predisposition**: Family history may increase risk.

**Unusual infections**: Some cases may involve infections not detected by standard tests.

**Role of Stress and Anxiety**

**Not a root cause**: Stress and anxiety are not considered primary causes of IC.

**Trigger for flares**: Many patients report that stress, emotional strain, or anxiety episodes worsen urgency, frequency, and bladder pain.

**Cycle effect**: Chronic pain and disrupted sleep from IC can increase emotional stress, which in turn aggravates symptoms — creating a feedback loop.

**Managing Stress to Reduce Symptoms**

**Stress reduction techniques**:

Mindfulness meditation or guided imagery

Yoga and gentle stretching

Cognitive behavioral therapy for coping with anxiety

Pelvic floor physical therapy to reduce muscle tension

**Lifestyle adjustments**:

Keeping a symptom diary to identify personal triggers

Avoiding bladder irritants (caffeine, alcohol, acidic foods)

Practicing bladder retraining to reduce urgency

**Quick Comparison: Causes vs. Triggers**

| **Factor** | **Role in IC** | **Evidence** |
| --- | --- | --- |
| **Bladder lining defect** | Possible underlying cause | Strong research support |
| **Autoimmune reaction** | Possible underlying cause | Moderate evidence |
| **Allergies/mast cells** | Possible underlying cause | Moderate evidence |
| **Stress/anxiety** | Symptom trigger, not cause | Patient-reported, clinical observation |
| **Dietary irritants** | Symptom trigger | Widely reported |

**️ Important Note**

If you suspect IC or have worsening bladder pain, urgency, or frequency, it’s important to consult a **urologist** or qualified healthcare professional for diagnosis and management. Stress management can help reduce flares, but medical evaluation is essential for proper care.

**7.Does interstitial cystitis usually last for a long time?**

**Yes — interstitial cystitis (IC), also called bladder pain syndrome, is a *chronic condition* that often lasts for years. Symptoms may flare and subside, but the disorder typically persists long-term and requires ongoing management rather than a short course of treatment.**

**Nature of Interstitial Cystitis**

**Chronic course**: IC is not a temporary infection; it is a long-term inflammatory condition of the bladder wall.

**No known cure**: Current treatments focus on symptom relief and improving quality of life rather than eliminating the disease.

**Variable progression**: Some people experience stable symptoms, others worsen over time, and some may have periods of remission.

Harvard Health

Credible health information source

**Interstitial cystitis**

Interstitial cystitis is a puzzling bladder condition in which the bladder wall becomes irritated or inflamed, causing pain and frequent or painful urination. The symptoms of interstitial cystitis are often similar to the symptoms of a urinary tract infection. However, in interstitial cystitis, there is no infection, and the symptoms do not respond to antibiotic treatment. The exact cause of interstitial cystitis remains a mystery, although researchers continue to investigate possible causes, such as unidentified bacteria, an allergic or immune system reaction, a toxic substance in the urine, or a neurological problem in the bladder wall. There also is some evidence that interstitial cystitis may not be just one illness, but several illnesses that share similar symptoms. Interstitial cystitis usually occurs between the ages of 20 and 50. Approximately 90% of people with interstitial cystitis are women. It is unknown why interstitial cystitis is more common in women. The disease is not known to be genetic (inherited) or caused by toxins in the environment.

**Key Facts**

**Duration**: IC often lasts for many years. Stage 4 IC is defined as symptoms persisting for over 2 years with bladder stiffening.

**Symptoms**: Pelvic pain, urinary urgency, and frequency (sometimes up to 60 times/day).

**Triggers**: Stress, menstruation, certain foods/drinks (caffeine, alcohol, acidic foods).

**Impact**: Can reduce bladder capacity, interfere with sleep, sexual intimacy, and daily activities.

**️ Outlook & Management**

| **Aspect** | **Details** |
| --- | --- |
| **Chronicity** | Long-term, often lifelong condition |
| **Remission** | Some patients experience symptom-free periods |
| **Treatment** | Medications (e.g., Elmiron, antidepressants, antihistamines), bladder instillations, diet changes, pelvic floor therapy, stress reduction |
| **Prognosis** | Not life-threatening, does not cause cancer, but can severely affect quality of life |

**Risks & Considerations**

**Emotional health**: Chronic pain and disrupted sleep may lead to depression or anxiety.

**Social impact**: Frequent urination and pain can interfere with work, relationships, and social life.

**Medical follow-up**: Regular care with a urologist is important to manage flares and adjust treatment.

**Visualizing the Condition**

In short, **interstitial cystitis usually lasts a long time** and is managed rather than cured. Would you like me to go deeper into **treatment options**, **lifestyle adjustments**, or **remission patterns**?

**8.Can interstitial cystitis symptoms come and go over time?**

**Yes — interstitial cystitis (IC) symptoms often fluctuate, with periods of flare-ups and remission. Many people experience cycles where pain, urgency, and frequency worsen temporarily, then ease for days, weeks, or even months.**

**Symptom Patterns in Interstitial Cystitis**

**Fluctuating course**: Symptoms may intensify during flare-ups and then subside, sometimes disappearing for extended periods.

**Common triggers**: Menstruation, prolonged sitting, stress, sexual activity, certain foods/drinks (e.g., caffeine, alcohol, acidic foods), and urinary tract infections can worsen symptoms.

**Remission periods**: Some patients report symptom-free intervals, while others experience chronic low-level discomfort.

**Individual variability**: Severity and frequency differ widely — some people urinate up to 60 times a day, while others only have occasional pelvic pain.

Harvard Health

Credible health information source

**Interstitial cystitis symptoms**

Interstitial cystitis may cause: 1. frequent urination; 2. an intense urge to urinate; 3. awakening from sleep to pass urine; 4. a burning sensation during urination; 5. pain, pressure, or tenderness in the area of the bladder — midline, below the navel, or in some other portion of the pelvis; 6. increasing discomfort as the bladder fills; 7. pain during sexual intercourse; 8. in men, pain or discomfort in the penis and scrotum; 9. in women, worsening symptoms during menstrual periods.

**Typical Symptoms**

**Frequent urination** (sometimes dozens of times per day)

**Urgent need to urinate** with small volumes

**Pelvic or bladder pain/pressure** that worsens as the bladder fills

**Burning sensation during urination**

**Pain during sex**

**Worsening symptoms during menstruation (in women)**

**️ Risks & Considerations**

**Quality of life impact**: Sleep disruption, sexual intimacy difficulties, and emotional stress are common.

**Complications**: Reduced bladder capacity over time due to stiffening of bladder walls.

**Overlap with other conditions**: Symptoms may mimic urinary tract infections, but IC usually occurs without infection.

**Management Strategies**

While there is no cure, many patients manage IC by combining approaches:

**Dietary adjustments**: Avoid acidic foods, caffeine, alcohol, and artificial sweeteners.

**Stress reduction**: Stress management techniques, yoga, or guided imagery can reduce flare frequency.

**Pelvic floor therapy**: Relaxation and strengthening exercises may help.

**Bladder retraining**: Gradually increasing time between bathroom visits.

**Medications**: Antihistamines, tricyclic antidepressants, bladder instillations, or Elmiron(the only FDA-approved oral drug for IC).

**Key Takeaway**

Interstitial cystitis is a **chronic but highly variable condition**. Symptoms can **come and go over time**, often triggered by lifestyle, diet, or stress. If you experience persistent bladder pain or urinary urgency, it’s important to consult a healthcare professional for diagnosis and tailored management.

**9.What are the most common symptoms of interstitial cystitis?**

**The most common symptoms of interstitial cystitis (IC), also known as bladder pain syndrome, include frequent urination, urgent need to urinate, pelvic or bladder pain, and discomfort that worsens as the bladder fills and improves after urination. Many patients also experience pain during sexual intercourse and symptom flare-ups linked to menstruation, stress, or prolonged sitting.**

Harvard Health

Credible health information source

**Interstitial cystitis symptoms**

Interstitial cystitis may cause: 1. frequent urination; 2. an intense urge to urinate; 3. awakening from sleep to pass urine; 4. a burning sensation during urination; 5. pain, pressure, or tenderness in the area of the bladder — midline, below the navel, or in some other portion of the pelvis; 6. increasing discomfort as the bladder fills; 7. pain during sexual intercourse; 8. in men, pain or discomfort in the penis and scrotum; 9. in women, worsening symptoms during menstrual periods.

**Key Symptoms of Interstitial Cystitis**

**Frequent urination**: Often small amounts, sometimes up to 40–60 times per day.

**Urgent need to urinate**: Persistent sensation of needing to void immediately.

**Pelvic/bladder pain**: Pressure, tenderness, or burning in the bladder region, lower abdomen, or pelvis.

**Discomfort with bladder filling**: Pain increases as the bladder fills and improves after urination.

**Nocturia**: Waking up multiple times at night to urinate.

**Pain during sex**: Common in women; men may feel discomfort in the penis or scrotum.

**Menstrual flare-ups**: Symptoms often worsen during periods.

**Symptom Overview Table**

| **Symptom** | **Description** | **Notes** |
| --- | --- | --- |
| **Frequency** | Urinating up to 60 times/day | Often small volumes |
| **Urgency** | Sudden, intense urge | Not always relieved by urination |
| **Pelvic pain** | Pressure, tenderness, burning | Can be chronic |
| **Nocturia** | Nighttime urination | Disrupts sleep |
| **Dyspareunia** | Pain during intercourse | More common in women |
| **Menstrual flare** | Worsening symptoms | Linked to hormonal changes |

**️ Important Considerations**

**Symptoms vary widely**: Some people have mild discomfort, while others experience severe pain and frequent urination.

**Triggers**: Stress, menstruation, certain foods/drinks (e.g., caffeine, alcohol, acidic foods), and prolonged sitting can worsen symptoms.

**Overlap with UTIs**: Symptoms resemble urinary tract infections, but IC usually shows no infection on testing.

**Impact on quality of life**: Chronic pain, sleep disruption, and sexual difficulties are common.

**Next Steps**

If you or someone you know is experiencing these symptoms, it’s important to consult a **urologist or healthcare provider** for proper evaluation. IC is a chronic condition, but treatments such as dietary changes, bladder retraining, medications, and physical therapy can help manage symptoms.

**10.Can interstitial cystitis cause frequent urination?**

**Yes — interstitial cystitis (IC) often causes frequent urination.** People with IC may feel a persistent urge to urinate, often passing only small amounts, sometimes up to 40–60 times per day. This happens because the bladder wall becomes irritated and inflamed, sending abnormal signals to the brain that trigger urgency even when the bladder isn’t full.

Harvard Health

Credible health information source

**Interstitial cystitis**

Interstitial cystitis is a puzzling bladder condition in which the bladder wall becomes irritated or inflamed, causing pain and frequent or painful urination. The symptoms of interstitial cystitis are often similar to the symptoms of a urinary tract infection. However, in interstitial cystitis, there is no infection, and the symptoms do not respond to antibiotic treatment. The exact cause of interstitial cystitis remains a mystery, although researchers continue to investigate possible causes, such as unidentified bacteria, an allergic or immune system reaction, a toxic substance in the urine, or a neurological problem in the bladder wall. There also is some evidence that interstitial cystitis may not be just one illness, but several illnesses that share similar symptoms. Interstitial cystitis usually occurs between the ages of 20 and 50. Approximately 90% of people with interstitial cystitis are women. It is unknown why interstitial cystitis is more common in women. The disease is not known to be genetic (inherited) or caused by toxins in the environment.

**Why IC Causes Frequent Urination**

**Bladder wall irritation**: Inflammation disrupts the bladder’s normal signaling, making it feel “full” prematurely.

**Reduced bladder capacity**: Scarring or stiffening of the bladder wall can limit how much urine it can hold.

**Neurological misfiring**: The nerves that communicate bladder fullness send mixed signals, leading to urgency and frequency.

**Common Symptoms of Interstitial Cystitis**

**Frequent urination** — often small amounts, day and night.

**Urgency** — strong, persistent need to urinate.

**Pelvic pain** — discomfort or pressure around the bladder, urethra, or lower abdomen.

**Pain during sex** — especially in women.

**Flare-ups** — symptoms worsen with triggers like menstruation, stress, certain foods, or prolonged sitting.

**️ Risks & Impact**

**Sleep disruption**: Nighttime urination (nocturia) can severely affect rest.

**Quality of life**: Frequent bathroom trips interfere with work, social life, and intimacy.

**Emotional stress**: Chronic pain and urgency may lead to anxiety or depression.

**️ Management Approaches**

While there is no cure, treatments aim to reduce frequency and pain:

**Medications** — e.g., Elmiron (pentosan polysulfate sodium), tricyclic antidepressants, antihistamines.

**Bladder instillations** — direct treatments inside the bladder.

**Diet changes** — avoiding acidic, spicy foods, caffeine, and alcohol.

**Physical therapy** — pelvic floor relaxation exercises.

**Nerve stimulation** — TENS or sacral nerve implants to reduce urgency.

**Key Takeaway**

Interstitial cystitis **does cause frequent urination**, often extreme, due to bladder irritation and reduced capacity. If you or someone you know experiences **chronic bladder pain, urgency, and frequent urination without infection**, it’s important to consult a healthcare professional for proper diagnosis and management.

**11.Can interstitial cystitis make someone feel an urgent need to urinate?**

**Yes — interstitial cystitis (IC), also called bladder pain syndrome, often causes a persistent and urgent need to urinate, even when the bladder is not full. This urgency is one of its hallmark symptoms, alongside bladder pain and frequent urination.**

**How Interstitial Cystitis Causes Urinary Urgency**

**Bladder wall irritation**: IC involves inflammation or irritation of the bladder lining, which disrupts normal signaling between the bladder and brain.

**Mixed nerve signals**: The bladder sends signals that it is full even when only small amounts of urine are present, creating a strong urge to urinate.

**Frequency and urgency**: Many people with IC urinate **up to 40–60 times per day**, often in small amounts, because of this false signaling.

Harvard Health

Credible health information source

**Interstitial cystitis**

Interstitial cystitis is a puzzling bladder condition in which the bladder wall becomes irritated or inflamed, causing pain and frequent or painful urination. The symptoms of interstitial cystitis are often similar to the symptoms of a urinary tract infection. However, in interstitial cystitis, there is no infection, and the symptoms do not respond to antibiotic treatment. The exact cause of interstitial cystitis remains a mystery, although researchers continue to investigate possible causes, such as unidentified bacteria, an allergic or immune system reaction, a toxic substance in the urine, or a neurological problem in the bladder wall. There also is some evidence that interstitial cystitis may not be just one illness, but several illnesses that share similar symptoms. Interstitial cystitis usually occurs between the ages of 20 and 50. Approximately 90% of people with interstitial cystitis are women. It is unknown why interstitial cystitis is more common in women. The disease is not known to be genetic (inherited) or caused by toxins in the environment.

**Common Symptoms of IC**

**Urgent need to urinate** — often persistent and difficult to ignore

**Frequent urination** — small volumes, day and night

**Pelvic or bladder pain** — discomfort that worsens as the bladder fills, sometimes relieved after urination

**Pain during sex** — especially in women

**Flare-ups** — symptoms may worsen with stress, menstruation, certain foods, or prolonged sitting

**️ Distinguishing IC from Other Conditions**

| **Condition** | **Urgency/Frequency** | **Pain** | **Response to Antibiotics** |
| --- | --- | --- | --- |
| **Interstitial cystitis** | Persistent urgency, frequent urination | Pelvic/bladder pain | No improvement |
| **Urinary tract infection** | Urgency, frequency | Burning pain | Improves with antibiotics |
| **Overactive bladder** | Urgency, frequency | Usually no pain | Managed with bladder training/meds |

**What To Do If You Experience These Symptoms**

**Consult a healthcare provider**: IC symptoms overlap with UTIs and other bladder conditions, so professional evaluation is essential.

**Keep a symptom diary**: Track urgency, frequency, pain, and potential triggers (foods, stress, menstrual cycle).

**Lifestyle adjustments**: Some patients find relief by avoiding acidic foods, caffeine, alcohol, and carbonated drinks.

**Treatment options**: While there is no cure, therapies include oral medications, bladder instillations, pelvic floor therapy, and stress management.

**12.Can interstitial cystitis cause bladder pressure without severe pain?**

**Yes — interstitial cystitis (IC), also called bladder pain syndrome, can cause bladder pressure or discomfort even without severe pain.** Symptoms vary widely: some people experience only mild pressure or urgency, while others have intense pelvic pain. The condition exists on a spectrum, and not everyone has the same severity.

Harvard Health

Credible health information source

**Interstitial cystitis**

Interstitial cystitis is a puzzling bladder condition in which the bladder wall becomes irritated or inflamed, causing pain and frequent or painful urination. The symptoms of interstitial cystitis are often similar to the symptoms of a urinary tract infection. However, in interstitial cystitis, there is no infection, and the symptoms do not respond to antibiotic treatment. The exact cause of interstitial cystitis remains a mystery, although researchers continue to investigate possible causes, such as unidentified bacteria, an allergic or immune system reaction, a toxic substance in the urine, or a neurological problem in the bladder wall. There also is some evidence that interstitial cystitis may not be just one illness, but several illnesses that share similar symptoms. Interstitial cystitis usually occurs between the ages of 20 and 50. Approximately 90% of people with interstitial cystitis are women. It is unknown why interstitial cystitis is more common in women. The disease is not known to be genetic (inherited) or caused by toxins in the environment.

**Symptom Variability in IC**

**Bladder pressure**: Many patients describe a sensation of fullness or pressure, especially as the bladder fills, even if pain is minimal.

**Mild discomfort**: Some individuals report only mild irritation or pressure rather than sharp pain.

**Urgency and frequency**: Needing to urinate often (sometimes up to 60 times per day) is common, even when pain is not severe.

**Fluctuating symptoms**: Symptoms can flare with triggers such as menstruation, stress, prolonged sitting, or certain foods. They may also remit for periods.

**️ Pain vs. Pressure**

| **Symptom** | **Possible Presentation** |
| --- | --- |
| **Bladder pressure** | Feeling of fullness or heaviness, sometimes mistaken for infection |
| **Pelvic pain** | Ranges from mild discomfort to severe, disabling pain |
| **Urgency/frequency** | Persistent urge to urinate, often small volumes |
| **Sexual discomfort** | Pain or pressure during intercourse in some cases |

Not all patients experience **severe pain** — some primarily struggle with **pressure and urinary urgency**.

**Important Considerations**

**Overlap with UTIs**: Symptoms resemble urinary tract infections, but IC does not respond to antibiotics.

**Triggers**: Stress, diet (acidic foods, caffeine, alcohol), and hormonal changes can worsen pressure sensations.

**Diagnosis**: IC is diagnosed after ruling out infections, bladder cancer, or other causes.

**Treatment**: Management focuses on symptom relief — diet changes, bladder retraining, medications, or physical therapy.

**What This Means for You**

If you feel bladder pressure without severe pain, it **can still be consistent with IC**. However, because symptoms overlap with other conditions, it’s important to consult a healthcare professional for evaluation. They can rule out infection or other causes and guide treatment options.

**13.Why is interstitial cystitis often hard to diagnose?**

**Interstitial cystitis (IC), also called bladder pain syndrome, is hard to diagnose because its symptoms overlap with many other conditions, there is no single definitive test, and diagnostic criteria vary across guidelines. Doctors often must rule out infections, cancer, and other pelvic disorders before confirming IC.**

**Why Diagnosis Is Difficult**

**Overlapping symptoms** IC symptoms (pelvic pain, urinary urgency, frequency, nocturia) mimic those of urinary tract infections, overactive bladder, endometriosis, and vulvodynia. This makes it challenging to distinguish IC from other causes of bladder or pelvic pain.

**Lack of definitive test** There is no biomarker or single diagnostic test for IC. Diagnosis relies on patient history, physical exam, bladder diaries, and exclusion of other conditions. Tests like cystoscopy or potassium sensitivity may help but are not universally reliable.

**Variable diagnostic criteria** The National Institute of Diabetes and Digestive and Kidney Diseases (NIDDK) criteria (1988) were designed for research, not clinical practice, and exclude many patients. Modern guidelines (e.g., American Urological Association, 2022) emphasize pragmatic definitions, but there is still no global consensus.

**Comorbid conditions** Many IC patients also have pelvic floor dysfunction, recurrent UTIs, or endometriosis. These comorbidities complicate diagnosis and may mask or mimic IC symptoms.

**Typical Diagnostic Workup**

**Medical history & bladder diary** – Track fluid intake, urine output, and symptom patterns.

**Pelvic exam** – Assess pelvic organs and rule out gynecological causes.

**Urinalysis & culture** – Exclude infection.

**Cystoscopy** – Check for Hunner lesions or bladder abnormalities.

**Biopsy/urine cytology** – Rule out bladder cancer.

**Potassium sensitivity test** – Sometimes used, though controversial.

**️ Key Challenges & Risks**

**Delayed diagnosis** – Many patients go years misdiagnosed with UTIs or overactive bladder.

**Symptom variability** – Pain and urgency can fluctuate, leading to inconsistent clinical findings.

**Patient burden** – Multiple invasive tests may be required before IC is considered.

**Treatment uncertainty** – Because diagnosis is complex, treatment often involves trial-and-error with medications, physical therapy, and lifestyle changes.

**Takeaway**

Interstitial cystitis is difficult to diagnose because it is a **diagnosis of exclusion**, with no gold-standard test and overlapping symptoms with other pelvic and urinary conditions. Clinicians rely on careful history, exclusion of other causes, and sometimes cystoscopy to confirm the condition.

**14.When should someone with possible interstitial cystitis see a doctor?**

**Someone with possible interstitial cystitis should see a doctor promptly if they experience chronic bladder pain, frequent or urgent urination, or pelvic discomfort. Seek medical care urgently if symptoms include fever, severe pain, or discomfort in the bladder, pelvic area, penis, or scrotum.**

Harvard Health

Credible health information source

**Interstitial cystitis when to call a professional**

Make an appointment to see a doctor if you are urinating more than usual or feel an intense urge to urinate. If you are having fevers, pain, or discomfort, especially in the pelvic/bladder area, penis, or scrotum, see your doctor soon.

**When to Seek Medical Attention**

**Frequent urination**: Going to the bathroom far more often than usual, especially if disrupting sleep or daily life.

**Urgent need to urinate**: Feeling a strong, persistent urge even when the bladder isn’t full.

**Pelvic or bladder pain**: Discomfort or pressure in the lower abdomen, pelvis, or genital area.

**Fever or systemic symptoms**: Could indicate infection or another condition requiring immediate evaluation.

**Pain during sex**: A common but concerning symptom that warrants medical review.

**️ Why It’s Important**

**Rule out infection**: Symptoms can mimic a urinary tract infection, but antibiotics won’t help if it’s interstitial cystitis.

**Prevent complications**: Untreated IC can reduce bladder capacity, interfere with sleep, and affect quality of life.

**Access treatment options**: Doctors may recommend medications (e.g., pentosan polysulfate), bladder instillations, or lifestyle changes.

**What to Expect at the Doctor**

**History & exam**: Questions about urinary habits, pain, and triggers.

**Urine tests**: To exclude infection or blood in urine.

**Specialist referral**: Often to a urologist for further evaluation.

**Possible cystoscopy**: To inspect bladder lining and rule out other causes.

**Quick Comparison: UTI vs. Interstitial Cystitis**

| **Feature** | **UTI** | **Interstitial Cystitis** |
| --- | --- | --- |
| Cause | Bacterial infection | Chronic bladder inflammation (non-infectious) |
| Response to antibiotics | Improves | No improvement |
| Symptoms | Burning urination, fever, cloudy urine | Pelvic pain, urgency, frequency, pain during sex |
| Diagnosis | Urine culture positive | Diagnosis of exclusion, cystoscopy may help |

**Key Takeaway**

If you suspect **interstitial cystitis**, don’t delay medical care. Early evaluation helps rule out infections or other conditions and opens the door to symptom management strategies that can improve quality of life.

**15.What symptoms with interstitial cystitis should be checked urgently?**

**If you have interstitial cystitis, you should seek urgent medical attention if symptoms suddenly worsen, especially if you develop *blood in the urine, fever, inability to urinate, or severe pelvic/bladder pain* that is different from your usual flares. These may signal infection, obstruction, or another condition requiring prompt evaluation.**

**Urgent Symptoms to Watch For**

While interstitial cystitis (IC) itself is chronic and not life-threatening, certain changes should trigger immediate medical review:

**Blood in urine** (visible red or brown urine) may indicate infection, stones, or bladder cancer.

**Fever or chills** IC does not cause fever; this suggests infection.

**Inability to urinate** or severe difficulty passing urine possible obstruction or acute urinary retention.

**Sudden severe pelvic/bladder pain** beyond your usual IC flares could indicate another urgent condition.

**Rapid worsening of frequency/urgency** (e.g., needing to urinate every few minutes) may signal infection or bladder injury.

**️ Typical IC Symptoms (Non-urgent but disruptive)**

These are common and usually managed with long-term care, but if they escalate, they can become urgent:

Frequent urination (sometimes up to 60 times/day)

Persistent urge to urinate

Burning sensation during urination

Pain, pressure, or tenderness in the bladder/pelvis

Pain during sex

Worsening symptoms during menstruation (in women)

Harvard Health

Credible health information source

**Interstitial cystitis symptoms**

Interstitial cystitis may cause: 1. frequent urination; 2. an intense urge to urinate; 3. awakening from sleep to pass urine; 4. a burning sensation during urination; 5. pain, pressure, or tenderness in the area of the bladder — midline, below the navel, or in some other portion of the pelvis; 6. increasing discomfort as the bladder fills; 7. pain during sexual intercourse; 8. in men, pain or discomfort in the penis and scrotum; 9. in women, worsening symptoms during menstrual periods.

**When to See a Doctor Immediately**

If your **symptoms resemble a urinary tract infection** (pain + fever + cloudy urine), since IC flares can mimic UTIs but infections need antibiotics.

If you experience **new hematuria (blood in urine)**, which is not typical for IC.

If **pain becomes unbearable** or interferes with daily functioning.

If you cannot pass urine at all (medical emergency).

**Practical Guidance**

**Keep a symptom diary**: Track pain, frequency, triggers (diet, stress, menstruation).

**Know your baseline**: IC symptoms fluctuate, but urgent signs are those that deviate sharply from your usual pattern.

**Contact your urologist promptly** if urgent symptoms occur. In Singapore, you can reach **National University Hospital Urology Centre** at +65 6779 5555.

**16.Should blood in the urine be checked if someone has interstitial cystitis symptoms?**

**Yes — blood in the urine (hematuria) should always be checked, even if someone has symptoms of interstitial cystitis.** While interstitial cystitis can cause bladder pain and urinary frequency, blood in the urine is not a typical feature and may signal another condition such as infection, stones, or even cancer. A healthcare professional should evaluate it promptly.

Harvard Health

Credible health information source

**Interstitial cystitis**

Interstitial cystitis is a puzzling bladder condition in which the bladder wall becomes irritated or inflamed, causing pain and frequent or painful urination. The symptoms of interstitial cystitis are often similar to the symptoms of a urinary tract infection. However, in interstitial cystitis, there is no infection, and the symptoms do not respond to antibiotic treatment. The exact cause of interstitial cystitis remains a mystery, although researchers continue to investigate possible causes, such as unidentified bacteria, an allergic or immune system reaction, a toxic substance in the urine, or a neurological problem in the bladder wall. There also is some evidence that interstitial cystitis may not be just one illness, but several illnesses that share similar symptoms. Interstitial cystitis usually occurs between the ages of 20 and 50. Approximately 90% of people with interstitial cystitis are women. It is unknown why interstitial cystitis is more common in women. The disease is not known to be genetic (inherited) or caused by toxins in the environment.

**Why Blood in Urine Needs Evaluation**

**Interstitial cystitis (IC)**: Causes bladder pain, urgency, and frequency, but usually **does not cause hematuria**. Some patients may have bladder wall changes (like Hunner’s ulcers) that can bleed, but this is uncommon.

**Hematuria (blood in urine)**: Can result from **urinary tract infections, kidney/bladder stones, enlarged prostate, kidney disease, or cancers** of the urinary tract.

**Microscopic vs. gross hematuria**:

*Microscopic*: Detected only on urine tests.

*Gross*: Visible red or cola-colored urine. Both require investigation.

**Typical Workup for Blood in Urine**

**Urinalysis & urine culture**: To check for infection or microscopic blood.

**Cystoscopy**: Direct visualization of the bladder to rule out cancer or ulcers.

**Imaging (CT, ultrasound, MRI)**: To detect stones, tumors, or structural problems.

**Blood tests**: To assess kidney function and rule out systemic causes.

**️ Key Risks if Ignored**

**Bladder or kidney cancer**: Hematuria is often the first sign.

**Kidney stones**: Can cause severe pain and obstruction.

**Infections**: May spread to kidneys if untreated.

**Bleeding disorders**: Could indicate systemic disease.

Harvard Health

Credible health information source

**Hematuria symptoms**

By itself, hematuria rarely causes symptoms. One exception is when the bladder has so much blood in it that clots form, and the flow of urine is blocked. This can cause pain at the site of the blockage in the lower pelvis. Symptoms usually come from the cause of the hematuria, and vary depending on the condition: 1. **Glomerulonephritis** — If glomerulonephritis is not severe, it may not cause any symptoms. If symptoms appear, they can include swelling, especially in the lower extremities, reduced urination, and high blood pressure; 2. **Kidney or bladder infection** — Symptoms depend on the site of infection, but can include intense pain on one side of the mid-back, fever, shaking chills, nausea and vomiting, pain above the pubic or bladder region, foul-smelling urine, the need to urinate more often than normal, and pain or discomfort during urination; 3. **Prostate infection**— There can be pain in the lower back or in the area between the scrotum and anus, pain during ejaculation, blood in the semen, and, sometimes, fever and chills; 4. **Tumor in the kidney or bladder** —Most kidney and bladder cancers grow without causing any pain or discomfort. When symptoms develop, the most common are abdominal pain, more frequent urination and pain at the end of urination; 5. **Kidney stones** — When a kidney stone becomes trapped in one of the ureters (the narrow tubes connecting each kidney to the bladder), it can cause severe pain in the back, side or groin, nausea and vomiting, or painful and frequent urination; 6. **Bleeding disorders** — Bleeding disorders tend to cause abnormal bleeding throughout the body, not just into the urine. Depending on the specific bleeding problem, symptoms can include abnormal bruising, prolonged bleeding from cuts, bleeding in the skin, bleeding into the joints or gastrointestinal tract (causing black, tarry stools or bright red blood in the stool), or gum bleeding even with gentle flossing or brushing; 7. **Trauma** — There often will be signs of traumatic injury to the body surface, such as bruises, swelling, punctures and open wounds.

**Comparison: Interstitial Cystitis vs. Hematuria**

| **Feature** | **Interstitial cystitis** | **Hematuria** |
| --- | --- | --- |
| Main symptoms | Bladder pain, urgency, frequency | Pink/red urine, sometimes pain |
| Typical cause | Bladder lining irritation, unknown | Infection, stones, cancer, trauma |
| Blood in urine | Rare (possible with Hunner’s ulcers) | Defining feature |
| Evaluation | Rule out infection, cystoscopy | Always requires full workup |
| Risk if untreated | Chronic pain, reduced bladder capacity | Missed cancer, kidney damage |

**Takeaway**

If someone with **interstitial cystitis symptoms notices blood in their urine**, it should **not be assumed to be part of IC**. A doctor should perform a full evaluation to rule out other serious conditions.

**17.Should fever with bladder pain be treated as interstitial cystitis?**

**Fever with bladder pain should *not* be treated as interstitial cystitis — it is more likely to indicate a urinary tract infection (UTI), which requires medical evaluation and often antibiotics. Interstitial cystitis is a chronic bladder pain condition that does *not* cause fever, while fever strongly suggests infection.**

**Key Distinction: Interstitial Cystitis vs. UTI**

| **Feature** | **Interstitial cystitis** | **Urinary tract infection** |
| --- | --- | --- |
| Cause | Chronic bladder inflammation, no infection | Bacterial infection (often *E. coli*) |
| Symptoms | Bladder/pelvic pain, urgency, frequency, pain during sex | Burning urination, urgency, frequency, cloudy/foul urine, **fever**, flank pain |
| Fever | **Absent** | **Common if infection spreads to kidneys** |
| Response to antibiotics | No improvement | Usually improves |
| Diagnosis | Exclusion of infection (urinalysis, cystoscopy) | Urine culture confirms bacteria |
| Treatment | Symptom management (diet, bladder instillations, pain relief) | Antibiotics, hydration, sometimes hospitalization for kidney infection |

**Why Fever Matters**

**Fever + bladder pain** almost always points to **infection** rather than interstitial cystitis.

UTIs can progress to **pyelonephritis (kidney infection)**, which is serious and requires urgent medical care.

Interstitial cystitis is **non-infectious** and does not cause systemic symptoms like fever, chills, or nausea.

**What To Do**

**Seek medical care promptly** if you have fever with bladder pain. A doctor will likely order a **urinalysis and urine culture** to confirm infection.

**Do not self-treat as interstitial cystitis** — delaying antibiotics can allow infection to spread.

If recurrent bladder pain occurs without fever and cultures are negative, then interstitial cystitis may be considered.

**️ Risks of Misdiagnosis**

Treating feverish bladder pain as interstitial cystitis risks **missing a bacterial infection**, which can lead to **sepsis or kidney damage**.

Conversely, treating interstitial cystitis with repeated antibiotics is ineffective and may cause **antibiotic resistance**.

**Takeaway**

If you have **fever with bladder pain**, it is far more consistent with a **urinary tract infection** than interstitial cystitis. Please consult a healthcare professional immediately for proper testing and treatment.

**18.What should someone do if interstitial cystitis pain suddenly becomes much worse?**

**If interstitial cystitis (IC) pain suddenly worsens, the most important step is to seek medical attention promptly to rule out infection or other complications. For immediate relief, patients often use self-care strategies (avoiding bladder irritants, applying heat, stress reduction), but severe or escalating pain should be evaluated by a healthcare professional.**

**When Pain Suddenly Worsens**

**Seek urgent medical care** if pain is intolerable, accompanied by fever, blood in urine, or inability to urinate — these may signal infection or another condition.

**Contact your urologist** if pain persists despite usual coping strategies. They may adjust medications or recommend bladder instillations.

**Emergency department** visits are appropriate if pain is unmanageable at home.

**️ Short-Term Relief Strategies**

**Heat therapy**: Heating pads or warm baths can soothe pelvic pain.

**Dietary adjustments**: Avoid common irritants (“four Cs”: caffeine, carbonated drinks, citrus, vitamin C-rich foods). Alcohol, spicy foods, and artificial sweeteners may also worsen flares.

**Stress management**: Relaxation techniques (deep breathing, visualization, gentle stretching).

**Loose clothing**: Reduces abdominal pressure.

**OTC pain relief**: NSAIDs like ibuprofen may help mild flares, but check with a doctor before use.

**️ Medical Options for Severe Flares**

**Oral medications**: Amitriptyline, antihistamines, pentosan polysulfate sodium (Elmiron), or NSAIDs may be prescribed for symptom control.

**Bladder instillations**: Lidocaine, heparin, or dimethyl sulfoxide (DMSO) delivered directly into the bladder can reduce pain.

**Nerve stimulation (TENS or sacral nerve stimulation)**: Used when other treatments fail.

**Botox injections**: May help block pain signals in the bladder wall.

**Physical therapy**: Pelvic floor relaxation techniques (avoid Kegel strengthening unless guided by a specialist).

**️ Risks & Considerations**

**Do not ignore worsening pain**: It may indicate infection, bladder ulcers (Hunner lesions), or another condition requiring urgent care.

**Avoid long-term antibiotics or steroids**: Guidelines advise against these due to poor efficacy and side effects.

**Surgery is rare**: Reserved for end-stage cases when all other therapies fail.

**Key Takeaway**

Sudden worsening of IC pain should **not be managed alone**. While self-care can help mild flares, **persistent or severe pain requires medical evaluation** to ensure safety and optimize treatment.

**19.What should someone do if they cannot urinate while having interstitial cystitis symptoms?**

Harvard Health

Credible health information source

**Interstitial cystitis**

Interstitial cystitis is a puzzling bladder condition in which the bladder wall becomes irritated or inflamed, causing pain and frequent or painful urination. The symptoms of interstitial cystitis are often similar to the symptoms of a urinary tract infection. However, in interstitial cystitis, there is no infection, and the symptoms do not respond to antibiotic treatment. The exact cause of interstitial cystitis remains a mystery, although researchers continue to investigate possible causes, such as unidentified bacteria, an allergic or immune system reaction, a toxic substance in the urine, or a neurological problem in the bladder wall. There also is some evidence that interstitial cystitis may not be just one illness, but several illnesses that share similar symptoms. Interstitial cystitis usually occurs between the ages of 20 and 50. Approximately 90% of people with interstitial cystitis are women. It is unknown why interstitial cystitis is more common in women. The disease is not known to be genetic (inherited) or caused by toxins in the environment.

**If someone with interstitial cystitis cannot urinate, this is a medical emergency and they should seek immediate care. Urinary retention can damage the bladder and kidneys, and urgent evaluation by a urologist or emergency department is necessary.**

**Immediate Steps**

**Seek emergency care**: Go to the nearest emergency department if unable to pass urine at all.

**Contact a urologist**: A specialist can assess whether retention is due to bladder inflammation, pelvic floor dysfunction, or another cause.

**Catheterization**: In hospital settings, a catheter may be placed to relieve retention and prevent complications.

**️ Why This Happens**

**Bladder wall inflammation** in interstitial cystitis can disrupt normal signaling, leading to urgency or difficulty voiding.

**Pelvic floor muscle dysfunction** may cause spasms that prevent urine flow.

**Pain flare-ups** can make urination difficult, even when the bladder is full.

Rarely, **secondary conditions** (e.g., obstruction, neurological issues) may coexist with interstitial cystitis and cause retention.

**Treatment Approaches**

**Multimodal pain management**: NSAIDs, urinary analgesics, or neuropathic pain medications may reduce flare-related retention.

**Pelvic floor physical therapy**: Helps relax muscles that may be blocking urine flow.

**Intravesical therapies**: Instillations of lidocaine, heparin, or DMSO can reduce bladder pain and improve function.

**Neuromodulation**: Electrical stimulation may help restore bladder signaling in refractory cases.

**Surgical options**: Reserved for severe, end-stage bladder disease when all other therapies fail.

**️ Risks of Ignoring Retention**

**Bladder damage**: Overstretching can reduce bladder capacity permanently.

**Kidney injury**: Backflow of urine can harm kidneys.

**Infections**: Stagnant urine increases risk of urinary tract infections.

**Severe pain**: Retention worsens pelvic pain and quality of life.

**Key Takeaway**

If someone with interstitial cystitis cannot urinate, **do not wait it out**. This requires **urgent medical evaluation** to relieve retention and prevent complications. Long-term management may involve pain control, pelvic floor therapy, and bladder-directed treatments, but the immediate priority is safe bladder emptying.

**20.What type of doctor usually treats interstitial cystitis?**

**Interstitial cystitis (IC), also called bladder pain syndrome, is usually treated by a urologist**, the specialist for bladder and urinary tract conditions. In some cases, patients may also see a **gynecologist** (for women with pelvic pain) or a **pain specialist** if symptoms are severe.

**️ Doctors Who Commonly Treat IC**

**Urologist**

Primary specialist for bladder and urinary tract disorders.

Performs diagnostic tests like cystoscopy and bladder biopsies.

Prescribes medications (e.g., Elmiron, tricyclic antidepressants, antihistamines) and bladder instillations.

**Gynecologist**

Often involved when IC overlaps with pelvic pain, menstrual cycle flares, or sexual health issues.

Helps rule out gynecological causes of pelvic pain.

**Pain management specialist**

Supports patients with chronic pain syndromes like IC, fibromyalgia, or irritable bowel syndrome.

May recommend nerve stimulation, pain medications, or integrative therapies.

**Pelvic floor physical therapist**

Works on muscle relaxation and retraining to reduce bladder pain and urgency.

**What to Expect in Treatment**

**Diagnosis**: Rule out infections, bladder cancer, or other causes with urinalysis, cystoscopy, and sometimes biopsy.

**Medications**: Elmiron (FDA-approved for IC), tricyclic antidepressants, antihistamines, or bladder instillations.

**Lifestyle management**: Diet changes (avoiding acidic foods, caffeine, alcohol), stress reduction, and bladder retraining.

**Advanced therapies**: Hydrodistension, nerve stimulation (TENS or sacral nerve implants), or surgery in rare severe cases.

**️ Risks & Considerations**

**Misdiagnosis risk**: IC symptoms mimic urinary tract infections, but antibiotics don’t help.

**Chronic nature**: IC is long-term; treatments focus on symptom relief, not cure.

**Emotional impact**: Pain and frequent urination can affect quality of life, relationships, and mental health. Support groups and counseling may help.

**Next Steps**

If you suspect IC, the best starting point is a **urologist consultation**. They can confirm the diagnosis and coordinate care with other specialists if needed. Would you like me to list **top urologists in Singapore** who specialize in bladder pain syndrome?

**21.How do doctors usually diagnose interstitial cystitis?**

**Doctors diagnose interstitial cystitis (IC), also called bladder pain syndrome, mainly by ruling out other conditions such as urinary tract infections or bladder cancer. There is no single definitive test — diagnosis relies on history, physical exams, urine tests, and often cystoscopy with possible biopsy.**

**How Doctors Diagnose Interstitial Cystitis**

**1. Medical History & Symptom Review**

**Bladder diary**: Patients may be asked to record fluid intake, urine output, urgency, and pain levels.

**Symptom discussion**: Frequency, urgency, pain during urination, pelvic discomfort, and flare patterns.

**2. Physical Examination**

**Pelvic exam** (for women): Checks external genitals, vagina, cervix, and pelvic organs.

**Digital rectal exam** (for men): Assesses prostate health.

Abdominal palpation to detect tenderness or abnormalities.

**3. Laboratory Tests**

**Urinalysis**: Rules out urinary tract infections.

**Urine cytology**: Examines cells to exclude bladder cancer.

Blood tests may be ordered if systemic issues are suspected.

**4. Cystoscopy & Biopsy**

**Cystoscopy**: A thin tube with a camera is inserted into the bladder to inspect the lining.

**Hydrodistention**: Bladder is filled with sterile water to test capacity and reveal ulcers or hemorrhages.

**Biopsy**: Tissue samples may be taken to rule out cancer or confirm inflammation.

**5. Specialized Tests**

**Potassium sensitivity test**: Instills potassium chloride and water into the bladder; increased pain with potassium suggests IC.

**Bladder capacity measurement**: Determines if IC has reduced bladder volume.

**Key Diagnostic Steps Compared**

| **Step** | **Purpose** | **What It Rules Out** |
| --- | --- | --- |
| **Medical history** | Identify symptom patterns | General urinary issues |
| **Pelvic/rectal exam** | Check pelvic organs | Gynecological/prostate conditions |
| **Urine tests** | Detect infection/cancer | UTI, bladder cancer |
| **Cystoscopy** | Visualize bladder lining | Ulcers, tumors |
| **Biopsy** | Tissue analysis | Cancer, inflammation |
| **Potassium test** | Assess bladder sensitivity | Confirms IC-specific pain response |

**️ Important Notes**

**No single test confirms IC** — diagnosis is made after persistent symptoms and exclusion of other causes.

**Referral to a urologist** is common for specialized evaluation.

**Hunner’s ulcers** (visible bladder lesions) are a strong indicator of IC but occur in only 5–10% of patients.

Diagnosis may take time, as IC overlaps with other chronic pelvic pain conditions.

**22.Is there one test that can prove someone has interstitial cystitis?**

**There is no single definitive test that can prove someone has interstitial cystitis (IC).** Diagnosis is made by ruling out other conditions and combining symptom history, physical exams, urine tests, and sometimes cystoscopy with bladder biopsy or hydrodistension. IC is essentially a diagnosis of exclusion.

Harvard Health

Credible health information source

**Interstitial cystitis diagnosis**

Your doctor will ask you about your urine (urine color, odor, presence of blood), symptoms during urination, any pain you are having, and if you are having fever, nausea, or vomiting. Your answers to these questions will provide clues about other possible causes of your symptoms, such as a bladder or kidney infection. Next, your doctor will examine you and will collect urine from you for laboratory tests that check for signs of infection and infection-causing bacteria. Women usually will need a pelvic exam and men will need a digital rectal exam to check the prostate gland. There is no one sign or test that can tell you immediately if you have interstitial cystitis. Usually, doctors diagnose the condition after a person has had persistent symptoms and no other cause has been found. Your doctor probably will refer you to a urologist to help make the diagnosis. He or she will do a test called cystoscopy to look for indications that you have interstitial cystitis and make sure there are no other causes of your symptoms. During cystoscopy, the urologist uses a small, tubelike instrument to look inside your bladder and examine its inner lining. A biopsy (a small sample of tissue) may be removed from your bladder wall to be examined in a laboratory to look for signs of inflammation and to check for other illnesses, including cancer. During the cystoscopy, your urologist may test your bladder's maximum capacity by filling it with sterile water. This is to determine whether interstitial cystitis has reduced your bladder's ability to hold urine.

**How Interstitial Cystitis Is Diagnosed**

**No single test** IC cannot be confirmed by one lab or imaging study. Doctors rely on persistent symptoms and exclusion of other causes.

**Medical history** Patients may be asked to keep a bladder diary noting fluid intake, urine output, urgency, and pain patterns.

**Physical exam** Includes pelvic exam for women and prostate exam for men to rule out other conditions.

**Urine tests** Used to exclude infections or hematuria. If urine is sterile but symptoms persist, IC may be suspected.

**Cystoscopy** A thin tube with a camera is inserted into the bladder to look for Hunner’s ulcers or glomerulations (tiny hemorrhages). Sometimes combined with **hydrodistension** (stretching the bladder with fluid) to assess bladder capacity.

**Biopsy** Tissue samples may be taken to rule out bladder cancer or other diseases.

**Potassium sensitivity test** Instills potassium chloride vs. water into the bladder; increased pain with potassium may suggest IC, though this test is less commonly used today.

**️ Key Considerations**

**Diagnosis of exclusion:** IC is considered only after infections, bladder cancer, kidney stones, and other causes are ruled out.

**Variable presentation:** Some patients have Hunner’s ulcers (about 5–10%), while most have non-ulcerative IC with subtle bladder wall changes.

**No universal biomarker:** Research continues, but there is currently no blood or urine marker that definitively proves IC.

**Specialist referral:** A urologist is usually needed for cystoscopy and advanced evaluation.

**Comparison of Diagnostic Tools**

| **Method** | **Purpose** | **Limitations** |
| --- | --- | --- |
| **Urine test** | Rule out infection, blood | Cannot confirm IC |
| **Bladder diary** | Track symptoms | Subjective |
| **Cystoscopy** | Visualize bladder lining | May miss subtle cases |
| **Biopsy** | Rule out cancer/inflammation | Invasive |
| **Potassium test** | Assess bladder sensitivity | Not widely used |

**Takeaway**

If you suspect IC, the next step is to **consult a urologist** who can perform cystoscopy and rule out other causes. While no single test proves IC, a combination of history, exclusion of other conditions, and bladder evaluation leads to diagnosis.

**23.Why do doctors need to rule out other problems before diagnosing interstitial cystitis?**

**Doctors must rule out other problems before diagnosing interstitial cystitis (IC) because its symptoms—such as bladder pain, urinary urgency, and frequency—closely mimic those of more common conditions like urinary tract infections, kidney disease, or bladder cancer. Since there is no single definitive test for IC, diagnosis is made only after excluding these other causes.**

**Why Ruling Out Other Conditions Is Essential**

**Symptom overlap**

IC symptoms (pain, urgency, frequency) are similar to urinary tract infections (UTIs), bladder stones, or gynecological issues.

Misdiagnosis could lead to inappropriate treatments, such as unnecessary antibiotics.

**No definitive test**

Unlike infections, which can be confirmed with urine cultures, IC lacks a single diagnostic marker.

Doctors rely on exclusion and symptom persistence.

**Serious conditions to exclude**

Bladder cancer, kidney disease, or prostate problems can present with similar urinary symptoms.

Ruling these out ensures that potentially life-threatening conditions aren’t overlooked.

**Typical Diagnostic Process**

**Medical history** Patients may be asked to keep a bladder diary noting fluid intake, urine volume, and symptom timing.

**Urine tests** Used to check for infection, blood, or abnormal cells. If infection is present, IC is not diagnosed.

**Pelvic or rectal exam** Helps identify gynecological or prostate-related causes.

**Cystoscopy** A camera is inserted into the bladder to look for ulcers (Hunner’s lesions) or rule out cancer. Sometimes combined with **hydrodistension** (stretching the bladder with fluid).

**Biopsy** Tissue samples may be taken to exclude cancer or other inflammatory diseases.

**️ Risks of Skipping Exclusion**

**Delayed treatment** for infections or cancer if misdiagnosed as IC.

**Unnecessary medications** (e.g., long-term antibiotics) that won’t help IC.

**Worsening symptoms** if underlying causes remain untreated.

**Comparison of Conditions with Similar Symptoms**

| **Condition** | **Key Symptoms** | **Diagnostic Test** | **Treatment** |
| --- | --- | --- | --- |
| **UTI** | Pain, urgency, frequency, fever | Urine culture | Antibiotics |
| **Kidney stones** | Severe flank pain, blood in urine | Imaging (CT, ultrasound) | Pain relief, stone removal |
| **Bladder cancer** | Blood in urine, pelvic pain | Cystoscopy + biopsy | Surgery, chemo, radiation |
| **Interstitial cystitis** | Chronic bladder pain, urgency, frequency | Exclusion + cystoscopy | Lifestyle changes, meds, bladder instillations |

**Key Takeaway**

Doctors must exclude infections, stones, and cancer before diagnosing IC because **IC is a diagnosis of exclusion**. Only after persistent symptoms remain unexplained by other conditions can IC be considered. This careful process protects patients from missed serious diagnoses and ensures appropriate treatment.

**24.Why do doctors check urine when interstitial cystitis is suspected?**

**Doctors check urine when interstitial cystitis (IC) is suspected mainly to rule out other conditions such as urinary tract infections (UTIs), kidney problems, or bladder cancer, since IC itself does not show a specific marker in urine. The urine test helps confirm that symptoms are not caused by infection or blood in the urine before proceeding with more specialized exams like cystoscopy.**

**Why Urine Tests Are Done in Suspected IC**

**Rule out infection** Urinalysis and urine culture check for bacteria, white blood cells, or nitrites that indicate a UTI. IC symptoms (pain, urgency, frequency) often mimic infections, so doctors must exclude them first.

**Check for blood** Microscopic or visible blood in urine can point to kidney stones, bladder cancer, or other urologic conditions. IC may cause bladder wall irritation, but persistent blood usually signals another issue.

**Exclude other bladder diseases** Urine cytology may be performed to look for abnormal cells that suggest cancer. This ensures IC is not misdiagnosed when a more serious condition is present.

**Baseline assessment** Doctors often combine urine tests with a bladder diary (tracking fluid intake and urination) to better understand symptom patterns.

**Typical Urine Tests in IC Workup**

| **Test** | **Purpose** | **What It Rules Out** |
| --- | --- | --- |
| **Urinalysis** | Checks for blood, protein, white cells | UTI, kidney disease |
| **Urine culture** | Identifies bacteria | Confirms/excludes infection |
| **Urine cytology** | Examines cells | Bladder cancer |
| **Bladder diary + urinalysis** | Tracks frequency/volume | Differentiates IC from overactive bladder |

**️ Key Point About Diagnosis**

**No single urine test confirms IC.** IC is a **diagnosis of exclusion**: doctors confirm it only after persistent symptoms (pain, urgency, frequency) continue and other causes are ruled out.

Harvard Health

Credible health information source

**Interstitial cystitis diagnosis**

Your doctor will ask you about your urine (urine color, odor, presence of blood), symptoms during urination, any pain you are having, and if you are having fever, nausea, or vomiting. Your answers to these questions will provide clues about other possible causes of your symptoms, such as a bladder or kidney infection. Next, your doctor will examine you and will collect urine from you for laboratory tests that check for signs of infection and infection-causing bacteria. Women usually will need a pelvic exam and men will need a digital rectal exam to check the prostate gland. There is no one sign or test that can tell you immediately if you have interstitial cystitis. Usually, doctors diagnose the condition after a person has had persistent symptoms and no other cause has been found. Your doctor probably will refer you to a urologist to help make the diagnosis. He or she will do a test called cystoscopy to look for indications that you have interstitial cystitis and make sure there are no other causes of your symptoms. During cystoscopy, the urologist uses a small, tubelike instrument to look inside your bladder and examine its inner lining. A biopsy (a small sample of tissue) may be removed from your bladder wall to be examined in a laboratory to look for signs of inflammation and to check for other illnesses, including cancer. During the cystoscopy, your urologist may test your bladder's maximum capacity by filling it with sterile water. This is to determine whether interstitial cystitis has reduced your bladder's ability to hold urine.

Further tests like **cystoscopy** and sometimes **biopsy** may be used to check bladder lining, capacity, and rule out Hunner’s lesions or cancer.

**Risks of Misdiagnosis**

Treating IC as a UTI can lead to **unnecessary antibiotics**.

Ignoring blood in urine without proper testing can miss **serious conditions** like bladder cancer.

That’s why urine testing is a **critical first step** before labeling symptoms as IC.

**25.Is looking inside the bladder always needed to diagnose interstitial cystitis?**

**No, looking inside the bladder (cystoscopy) is not always required to diagnose interstitial cystitis.** Doctors usually rely first on history, physical exam, and urine tests to rule out infections or other causes. Cystoscopy is typically reserved for cases where the diagnosis is uncertain, or when specific bladder lesions (like Hunner’s ulcers) are suspected.

**How Interstitial Cystitis (IC) Is Diagnosed**

**Medical history**: Patients are asked about urinary symptoms, pain, and triggers. A bladder diary may be used.

**Physical exam**: Pelvic exam for women, rectal exam for men, plus abdominal assessment.

**Urine tests**: Rule out urinary tract infection or blood in urine.

**Cystoscopy**: A thin camera scope is inserted into the bladder. Used mainly to check for Hunner’s ulcers or exclude bladder cancer.

**Biopsy**: Sometimes taken during cystoscopy to rule out cancer or other conditions.

**Potassium sensitivity test**: Rarely used; compares bladder response to potassium vs. water solutions.

**When Cystoscopy Is Needed vs. Optional**

| **Situation** | **Role of Cystoscopy** |
| --- | --- |
| Typical IC symptoms, infection ruled out | Not strictly necessary |
| Diagnosis uncertain | Recommended |
| Suspected Hunner’s ulcers | Strongly indicated |
| Need to exclude bladder cancer | Required |
| Assess bladder capacity (hydrodistention) | Sometimes performed |

**️ Key Considerations**

**No single test confirms IC** — it’s a diagnosis of exclusion after ruling out infections, stones, or cancer.

**Cystoscopy is invasive** and usually requires anesthesia if bladder stretching (hydrodistention) is performed.

**Guidelines (AUA 2022)** state cystoscopy is *not mandatory* in uncomplicated cases; it should be considered only when the diagnosis is in doubt or Hunner lesions are suspected.

Harvard Health

Credible health information source

**Interstitial cystitis diagnosis**

Your doctor will ask you about your urine (urine color, odor, presence of blood), symptoms during urination, any pain you are having, and if you are having fever, nausea, or vomiting. Your answers to these questions will provide clues about other possible causes of your symptoms, such as a bladder or kidney infection. Next, your doctor will examine you and will collect urine from you for laboratory tests that check for signs of infection and infection-causing bacteria. Women usually will need a pelvic exam and men will need a digital rectal exam to check the prostate gland. There is no one sign or test that can tell you immediately if you have interstitial cystitis. Usually, doctors diagnose the condition after a person has had persistent symptoms and no other cause has been found. Your doctor probably will refer you to a urologist to help make the diagnosis. He or she will do a test called cystoscopy to look for indications that you have interstitial cystitis and make sure there are no other causes of your symptoms. During cystoscopy, the urologist uses a small, tubelike instrument to look inside your bladder and examine its inner lining. A biopsy (a small sample of tissue) may be removed from your bladder wall to be examined in a laboratory to look for signs of inflammation and to check for other illnesses, including cancer. During the cystoscopy, your urologist may test your bladder's maximum capacity by filling it with sterile water. This is to determine whether interstitial cystitis has reduced your bladder's ability to hold urine.

**Bottom Line**

Doctors don’t always need to look inside the bladder to diagnose interstitial cystitis. **Cystoscopy is mainly used when symptoms are atypical, when other conditions must be ruled out, or when Hunner’s ulcers are suspected.** Most diagnoses rely on history, exam, and exclusion of other causes.

Would you like me to explain more about Hunner’s ulcers, the bladder lesions that make cystoscopy more important, or about non-invasive tests that can help in diagnosis?

**26.Can food or drinks make interstitial cystitis symptoms worse?**

**Yes — certain foods and drinks can worsen interstitial cystitis (IC) symptoms by triggering bladder pain, urgency, or flares. Common culprits include acidic foods, caffeine, alcohol, spicy dishes, and artificial sweeteners, though triggers vary widely between individuals.**

**Common IC Food & Drink Triggers**

Many patients report flares after consuming these items:

**Caffeinated drinks**: coffee, black/green tea, energy drinks

**Alcohol**: wine, beer, cocktails

**Citrus fruits & juices**: oranges, lemons, grapefruits

**Tomatoes & tomato products**: sauces, ketchup, salsa

**Spicy foods**: chili, hot peppers, hot sauce

**Chocolate**: especially dark chocolate (caffeine + acid)

**Artificial sweeteners**: aspartame, saccharin

**Carbonated beverages**: soda, sparkling water

**Vinegar & pickled foods**

**MSG**: common in processed foods

**Certain fruits**: pineapple, cranberries, strawberries

**Foods & Drinks Often Considered Safer**

Many IC patients tolerate these better:

**Proteins**: chicken, turkey, most fish, eggs

**Grains**: rice, oats, quinoa, pasta (without spicy sauces)

**Vegetables**: broccoli, green beans, zucchini, carrots, potatoes, squash

**Low-acid fruits**: pears, blueberries, watermelon, peeled apples

**Dairy**: milk, mild cheeses, plain yogurt (individual tolerance varies)

**Beverages**: plain water, chamomile tea, marshmallow root tea

**Fats**: olive oil, coconut oil, avocado oil

**How to Identify Your Personal Triggers**

**Keep a food diary**: Track meals and symptoms for 2–3 weeks.

**Try an elimination diet**: Remove common triggers, then reintroduce one at a time.

**Notice patterns**: Some tolerate small portions but not large ones.

**Hydrate wisely**: Drinking plenty of plain water dilutes irritants.

**️ Important Considerations**

**Triggers vary greatly** — not everyone reacts to the same foods.

**Symptom changes may take weeks** after removing a trigger (e.g., coffee).

**Professional guidance**: A registered dietitian can help design a personalized IC-friendly eating plan.

**Medical advice**: If symptoms are persistent or worsening, consult a urologist or healthcare provider for tailored management.

Would you like me to create a **structured IC-safe meal plan** or a **step-by-step elimination diet guide** to help you manage triggers more effectively?

**27.Which drinks commonly bother people with interstitial cystitis?**

**Common drinks that bother people with interstitial cystitis (IC) include coffee, tea, soda, alcohol, citrus juices, cranberry juice, and beverages with artificial sweeteners. These are frequently reported to trigger bladder pain, urgency, and flare-ups.**

**Drinks That Commonly Trigger IC Symptoms**

| **Drink Type** | **Examples** | **Why It Bothers IC** |
| --- | --- | --- |
| **Coffee & Tea** | Regular, decaf, green tea, black tea | Contains caffeine and other bladder irritants; even decaf can trigger symptoms. |
| **Soda & Energy Drinks** | Cola, diet soda, energy drinks | Carbonation, caffeine, acids, and artificial sweeteners irritate bladder. |
| **Alcohol** | Beer, wine, spirits, cocktails | Diuretic effect, acidity, sulfites, and dehydration worsen bladder pain. |
| **Citrus Juices** | Orange, grapefruit, lemon, lime | High acidity damages bladder lining and increases urgency. |
| **Cranberry Juice** | Pure juice, cranberry cocktails | Very acidic; often worsens IC symptoms despite its use in UTI prevention. |
| **Artificial Sweetener Drinks** | Diet sodas, sugar-free flavored waters | Aspartame, saccharin, sucralose linked to bladder irritation. |

**Safer Alternatives**

**Herbal teas** (chamomile, peppermint, rooibos) — caffeine-free, soothing.

**Plain water** — staying hydrated dilutes urine and reduces irritation.

**Low-acid juices** — pear or blueberry juice are often better tolerated.

**Milk or non-dairy alternatives** — generally bladder-friendly.

**Key Management Tips**

**Keep a food & drink diary**: Track what causes flares; triggers vary by person.

**Try elimination diets**: Remove suspected drinks, then reintroduce slowly to confirm sensitivity.

**Work with a dietitian**: They can help tailor a bladder-friendly nutrition plan.

**️ Important Note**

Not everyone with IC reacts the same way — while coffee or citrus juices are common triggers, some people tolerate them in small amounts. Identifying *your personal triggers* is the most effective strategy for managing IC symptoms.

Would you like me to share a **sample IC-friendly beverage plan** that replaces common irritants with safer alternatives?

**28.Can coffee make interstitial cystitis symptoms worse?**

**Yes — coffee often worsens interstitial cystitis (IC) symptoms. Both caffeinated and decaffeinated coffee can irritate the bladder due to their acidity, and caffeine itself increases urgency and pain. Many IC specialists recommend avoiding coffee entirely and switching to bladder-friendly alternatives.**

**Why Coffee Can Trigger IC Flare-Ups**

**Caffeine impact**: Stimulates the bladder, increases frequency of urination, and irritates the bladder lining, worsening urgency and pain.

**Acidity factor**: Even decaf coffee is acidic, which can inflame the bladder and trigger flares.

**Habit-forming nature**: Stopping suddenly may cause headaches and fatigue, so gradual reduction is advised.

**Safer Alternatives for IC Patients**

**Herbal teas**: Chamomile, peppermint, rooibos, and marshmallow root tea are soothing and caffeine-free.

**Coffee substitutes**: Chicory root coffee, barley coffee, or vegetable-based blends like Cafix®, Pero®, Roma, and Postum®.

**Hydration strategies**: Drinking plenty of water helps dilute urine and reduce irritation.

**Comparison Table: Coffee vs. IC-Friendly Alternatives**

| **Option** | **Bladder Impact** | **Notes** |
| --- | --- | --- |
| Regular Coffee | High irritation | Caffeine + acidity worsen IC symptoms |
| Decaf Coffee | Moderate irritation | Still acidic, often triggers flares |
| Chicory Root Coffee | Low irritation | Rich flavor, caffeine-free |
| Barley Coffee | Low irritation | Nutty taste, gentle on bladder |
| Chamomile Tea | Soothing | Anti-inflammatory, calming |
| Rooibos Tea | Gentle | Antioxidant-rich, naturally sweet |

**Risks & Considerations**

**Decaf myth**: Decaffeinated coffee is *not* safe for IC patients — acidity remains a trigger.

**Artificial sweeteners**: Can also worsen bladder pain; natural sweeteners like honey or stevia are safer.

**Dietary triggers**: Citrus, tomato products, spicy foods, and alcohol are common irritants alongside coffee.

**Practical Tips**

Gradually taper coffee intake to avoid withdrawal headaches.

Replace morning coffee with herbal teas or low-acid substitutes.

Track symptoms in a bladder diary to identify personal triggers.

Consult a healthcare professional before making major dietary changes.

Would you like me to focus more on **coffee substitutes** that taste closest to real coffee, or on **herbal teas** that provide soothing relief?

**29.Does everyone with interstitial cystitis need to avoid the same foods?**

**Not everyone with interstitial cystitis (IC) needs to avoid the same foods — dietary triggers vary widely between individuals.** While common irritants include caffeine, alcohol, citrus, spicy foods, and artificial sweeteners, each person must identify their own triggers through trial and error, often using a food diary.

Harvard Health

Credible health information source

**What foods and drinks should I avoid to reduce my chance of interstitial cystitis?**

Interstitial cystitis is a puzzling bladder condition, in which the bladder wall becomes irritated or inflamed. The condition causes a feeling of needing to urinate frequently and often pain with urination. There also may be a constant pelvic discomfort. Caffeinated beverages, alcohol, citrus fruits, spicy foods and chocolate are just a few in a long list of foods that aggravate interstitial cystitis in some people. Each person needs to discover what she or he needs to avoid.

**️ Common Food Triggers**

Many people with IC report symptom flares after consuming:

**Caffeinated drinks**: coffee, tea, soda

**Alcohol**: wine, beer, spirits

**Citrus fruits**: oranges, lemons, grapefruits

**Spicy foods**: chili, hot sauces

**Chocolate**

**Artificial sweeteners**: saccharin, aspartame

**Tomato-based foods**: sauces, ketchup

These foods are acidic or contain compounds that may irritate the bladder lining.

**Individual Variation**

**Not universal**: Some people tolerate certain foods that others cannot.

**Food diary method**: Tracking meals and symptoms helps identify personal triggers.

**Trial period**: Experts recommend eliminating suspected foods for at least 3 months, then reintroducing them one at a time to test tolerance.

**Dietitian support**: A registered dietitian can help create a balanced plan that avoids nutrient gaps while minimizing bladder irritation.

**Foods Often Well-Tolerated**

Many IC patients find these safer:

**Pears**, blueberries, watermelon

**Rice**, oats, potatoes

**Lean proteins**: chicken, turkey, fish

**Low-fat dairy**: milk, mild cheeses

**Non-acidic vegetables**: green beans, cucumbers, zucchini

These foods are less acidic and less likely to trigger bladder pain.

**️ Risks of Over-Restriction**

Cutting out too many foods can lead to **nutrient deficiencies**.

Balanced nutrition is essential for **immune support, wound healing, and nerve function**.

Substitutions (e.g., pear juice instead of orange juice) help maintain variety.

**Key Takeaway**

There is **no one-size-fits-all IC diet**. The best approach is to start with common irritants, keep a food diary, and personalize your diet. Working with a dietitian can help balance bladder comfort with overall health.

Would you like me to outline a **step-by-step elimination diet plan** or a list of **safe meal ideas** to make this more practical?

**30.How can someone with interstitial cystitis find out which foods bother their bladder?**

Harvard Health

Credible health information source

**Interstitial cystitis**

Interstitial cystitis is a puzzling bladder condition in which the bladder wall becomes irritated or inflamed, causing pain and frequent or painful urination. The symptoms of interstitial cystitis are often similar to the symptoms of a urinary tract infection. However, in interstitial cystitis, there is no infection, and the symptoms do not respond to antibiotic treatment. The exact cause of interstitial cystitis remains a mystery, although researchers continue to investigate possible causes, such as unidentified bacteria, an allergic or immune system reaction, a toxic substance in the urine, or a neurological problem in the bladder wall. There also is some evidence that interstitial cystitis may not be just one illness, but several illnesses that share similar symptoms. Interstitial cystitis usually occurs between the ages of 20 and 50. Approximately 90% of people with interstitial cystitis are women. It is unknown why interstitial cystitis is more common in women. The disease is not known to be genetic (inherited) or caused by toxins in the environment.

**The most reliable way for someone with interstitial cystitis (IC) to figure out which foods bother their bladder is to use a structured elimination diet combined with a food-and-symptom diary.** Because triggers vary widely, it takes weeks of careful tracking and gradual reintroduction to identify personal irritants.

**Why Food Triggers Matter in IC**

**IC is not an infection** but a chronic bladder pain condition where the bladder lining is sensitive and easily irritated.

Foods that increase **urine acidity, chemical irritation, or bladder stimulation** can worsen pain, urgency, and frequency.

**Common Trigger Foods**

These are frequently reported irritants, though not universal:

**Coffee & tea** (both caffeinated and decaf)

**Alcohol** (wine, beer, cocktails)

**Citrus fruits & juices** (orange, lemon, grapefruit)

**Tomatoes** and tomato-based sauces

**Spicy foods** (chili, hot peppers, hot sauce)

**Chocolate** (especially dark)

**Artificial sweeteners** (aspartame, saccharin)

**Carbonated drinks** (soda, sparkling water)

**Vinegar & pickled foods**

**MSG** and processed foods

**Foods Often Better Tolerated**

**Proteins**: chicken, turkey, fish, eggs

**Grains**: rice, oats, quinoa, pasta (without spicy sauces)

**Vegetables**: broccoli, zucchini, carrots, potatoes

**Fruits (low-acid)**: pears, blueberries, watermelon, peeled apples

**Dairy**: milk, mild cheeses, plain yogurt

**Beverages**: plain water, chamomile tea, marshmallow root tea

**How to Identify Your Personal Triggers**

**Keep a food diary**

Record meals, drinks, and symptoms daily for at least 2–3 weeks.

**Try an elimination diet**

Remove common triggers, then reintroduce one food at a time.

Wait several days to weeks before judging its effect.

**Be patient**

Improvement may take weeks, not days. For example, coffee may need to be avoided for several weeks before noticing changes.

**️ Risks & Considerations**

**Individual variation**: Not every trigger affects every person.

**Hidden irritants**: Processed foods may contain additives (citric acid, MSG, artificial sweeteners) that worsen symptoms.

**Nutritional balance**: Work with a dietitian to avoid overly restrictive eating.

**Medical guidance**: Always consult a healthcare professional before making major dietary changes, especially if symptoms are severe or worsening.

**Next Steps**

Would you like me to walk you through a **step-by-step elimination diet plan**, or provide a **sample IC-friendly meal plan** to get started?

**31.Should people with interstitial cystitis drink less water to urinate less often?**

**People with interstitial cystitis (IC) should not simply drink less water to urinate less often — restricting fluids can make urine more concentrated, which irritates the bladder and worsens symptoms. The key is *finding a balanced intake* that keeps urine pale yellow (dilute) without overwhelming the bladder.**

**️ Hydration Balance in IC**

**Too little water**

Leads to concentrated urine (dark yellow).

Increases bladder irritation, burning, and urgency.

**Too much water**

Overfills the bladder, heightens urgency, and increases frequency.

Can disrupt sleep and daily activities.

**Optimal intake**

Typically **6–8 glasses per day (1.5–2 liters)** spread evenly.

Adjust based on symptoms and urine color: pale yellow = balanced hydration.

**Clinical Guidelines**

The **American Urological Association (AUA)** places fluid management as a **first-line treatment** for IC/BPS, before medications.

The goal is **individualized fluid management** guided by symptom tracking, not a fixed number.

**Common Beverage Triggers**

Many IC patients report flares from specific drinks. Avoiding these can reduce frequency and pain:

| **Trigger** | **Examples** |
| --- | --- |
| **Caffeine** | Coffee, tea, cola, energy drinks |
| **Alcohol** | Beer, wine, spirits |
| **Acidic juices** | Orange, grapefruit, cranberry, tomato |
| **Carbonated drinks** | Soda, sparkling water |
| **Artificial sweeteners** | Diet sodas, sugar-free drinks |
| **Safest option** | Plain water |

**Risks of Fluid Restriction**

Short-term reduction may ease frequency, but **long-term restriction worsens pain and urgency**.

A clinical trial showed that **reducing fluid intake by 25% improved symptoms**, but complete restriction is harmful.

**Practical Tips**

**Track symptoms** alongside fluid intake to find your personal balance.

**Check urine color** daily: aim for pale yellow.

**Spread fluids** evenly throughout the day instead of large amounts at once.

**Avoid bladder irritants** (caffeine, alcohol, acidic drinks).

**Consult a urologist** for tailored advice — especially if symptoms are persistent or worsening.

️**Important Note:** If you have IC, do not drastically reduce water intake on your own. Always discuss hydration strategies with a qualified healthcare professional to avoid worsening bladder irritation.

Would you like me to share **dietary strategies** that complement fluid management, or focus more on **AUA treatment guidelines** for IC?

**32.Can emotional stress make interstitial cystitis symptoms worse?**

**Yes — emotional stress can worsen interstitial cystitis (IC) symptoms.** Stress doesn’t cause IC, but it can trigger flare-ups and intensify pain, urgency, and frequency by affecting both the nervous system and immune response. Many patients report symptom spikes during periods of emotional or physical stress.

**How Stress Impacts IC Symptoms**

**Nervous system activation**: Stress stimulates the hypothalamic-pituitary-adrenal (HPA) axis, releasing cortisol and other stress hormones. This can heighten bladder sensitivity and pain perception.

**Immune and inflammatory effects**: Chronic stress lowers the body’s ability to regulate inflammation, contributing to bladder irritation.

**Sleep and muscle tension**: Stress often disrupts sleep and increases pelvic muscle tension, both of which can worsen IC discomfort.

**Psychological factors**: Conditions like PTSD are linked to more severe IC symptoms, with higher pain levels and reduced quality of life.

**Evidence from Research**

| **Finding** | **Impact on IC** |
| --- | --- |
| Higher perceived stress levels | Associated with more severe bladder pain and urgency |
| Stress-related diseases (SRDs) | Patients with SRDs had higher odds of developing IC/BPS |
| PTSD prevalence | 42% of IC patients met PTSD criteria, linked to worse symptoms |
| Mindfulness-based stress reduction (MBSR) | 8-week program improved symptoms and urinary microbiome |

**Stress Management Strategies for IC**

**Mindfulness meditation**: Helps reduce anxiety and improve pain perception.

**Cognitive behavioral therapy**: Teaches coping skills and reframes stress responses.

**Gentle exercise**: Yoga, stretching, or walking can ease muscle tension.

**Relaxation techniques**: Breathing exercises, visualization, or self-hypnosis.

**Massage therapy**: Relieves pelvic and back muscle tension.

**Psychotherapy**: Provides tools for managing emotional distress.

**️ Important Note**

If you are experiencing **persistent or worsening IC symptoms**, it’s important to consult a **qualified healthcare professional**. Stress management can help, but medical evaluation ensures that other contributing factors are addressed.

Would you like me to share more about **dietary triggers** that often interact with stress to worsen IC symptoms, or focus on **mind-body therapies** that patients find most effective?

**33.What daily habits may help reduce interstitial cystitis flare-ups?**

**Daily habits that may help reduce interstitial cystitis (IC) flare-ups include staying well-hydrated, avoiding bladder irritants like caffeine and acidic foods, practicing stress management, and maintaining gentle physical activity. These lifestyle adjustments can significantly ease bladder pain and urinary urgency when consistently applied.**

**Key Daily Habits for Managing IC**

**Dietary Adjustments**

**Avoid bladder irritants**: Coffee, tea, alcohol, citrus fruits, tomatoes, chocolate, spicy foods, and carbonated drinks often worsen symptoms.

**Choose bladder-friendly foods**: Bananas, blueberries, pears, rice, chicken, leafy greens, and alkaline-rich vegetables are generally safe.

**Keep a food diary**: Track meals and symptoms to identify personal triggers.

**Hydration Practices**

**Drink 8–10 glasses of water daily** to dilute urine and reduce bladder irritation.

Prefer **plain, non-carbonated water**; avoid sparkling or flavored waters.

Incorporate hydrating foods like cucumber, celery, and zucchini.

**Stress Management**

Stress can worsen IC symptoms. Helpful techniques include:

Meditation or mindfulness

Deep breathing exercises

Gentle yoga (Hatha, Yin)

Progressive muscle relaxation

Cognitive-behavioral therapy (CBT)

**Bladder Training**

Under medical guidance, gradually increase the time between urination to improve bladder capacity.

Avoid bladder training during painful flare-ups.

**Physical Activity**

Gentle exercises like walking, swimming, or yoga can improve circulation and reduce stress.

Pelvic floor therapy may help relieve muscle tension and improve bladder control.

**Sleep & Lifestyle**

Aim for **7–8 hours of sleep nightly** to support healing.

Wear **loose, comfortable clothing** to avoid bladder pressure.

**Quit smoking**: Nicotine irritates the bladder and coughing strains pelvic muscles.

**️ Risks & Considerations**

**Trigger foods vary**: What irritates one person may not affect another. Always test changes gradually.

**Overhydration** can worsen urgency; balance intake with comfort.

**Exercise intensity**: High-impact workouts may aggravate symptoms—stick to low-impact routines.

**Stress management** is not a cure but can reduce flare frequency.

**Quick Reference Table**

| **Habit** | **Helpful Action** | **Avoid/Limit** |
| --- | --- | --- |
| **Diet** | Bananas, leafy greens, rice | Citrus, tomatoes, caffeine, alcohol |
| **Hydration** | 8–10 glasses plain water | Sparkling water, sodas |
| **Stress** | Meditation, yoga, CBT | Ignoring stress triggers |
| **Exercise** | Walking, swimming, pelvic therapy | High-impact workouts |
| **Lifestyle** | Loose clothing, good sleep | Smoking, tight waistbands |

**34.Can interstitial cystitis be cured completely?**

**Interstitial cystitis (IC) cannot be completely cured — it is a chronic bladder condition.** The main goal of treatment is to reduce symptoms such as pain, urgency, and frequency. While some people experience remission or long-term relief, there is currently no therapy that eliminates the disease entirely.

Harvard Health

Credible health information source

**Interstitial cystitis treatment**

The goal of treatment is to reduce symptoms. No one therapy is so successful that it should always be tried first. Often, a person with interstitial cystitis needs to try several therapies before finding the right combination. The symptoms of interstitial cystitis may go away over time, but there is no therapy that cures the disorder. Treatments include: 1. **Dietary modification:** Caffeinated beverages, alcohol, citrus fruits, spicy foods, and chocolate are just a few in a long list of foods that aggravate interstitial cystitis in some people. Each person needs to discover what she or he needs to avoid; 2. **Bladder training:** In this therapy, patients learn to reduce frequent urination by following a schedule for urinating. While this type of treatment can help reduce the frequency of urination, it usually does not reduce pain; 3. **Oral medications:** Pentosan polysulfate sodium (Elmiron) is the only oral medication specifically approved for the treatment of interstitial cystitis. About 30% of people with interstitial cystitis have fewer symptoms while taking this medication. Side effects, including effects on the eye, have limited its use. There is a long list of other medications that are not approved specifically for interstitial cystitis, but may offer relief. These include ibuprofen (Advil, Motrin, and others); naproxen (Aleve, Naprosyn, and others); aspirin; acetaminophen (Tylenol and others); tricyclic antidepressants, such as amitriptyline (Elavil, others); hydroxyzine (Atarax, Vistaril); methenamine (Hiprex); and phenazopyridine (Pyridium, others); 4. **Bladder distention:** Sterile water is used to stretch the bladder. Most patients feel worse for a couple of weeks after the procedure. After that, 30% to 50% of patients feel better. The beneficial effect tends to be temporary (lasting up to a few months) and the procedure is done under general anesthesia, which carries certain risks; 5. **Bladder instillation** (also called bladder wash): In this procedure, the bladder is filled with a sterile solution containing one of a number of ingredients that work directly on the bladder wall. The solution is put into the bladder through a catheter (hollow tube) that is placed through the urethra. After a variable period of time, the person is instructed to empty his or her bladder. Several active ingredients have been used in this procedure, but DMSO, sodium bicarbonate, steroids, heparin, and topical anesthetics are the ones tried most frequently; of these, only DMSO is FDA-approved for interstitial cystitis. As with other therapies, success is variable. Repeating the procedure risks causing more irritation of the bladder wall and introducing infection; 6. **Electrical nerve stimulation:** Traditionally, this has been done with a device called a TENS (transcutaneous electrical nerve stimulator) unit. Mild electrical impulses are passed into the body through wires placed below the navel, on the lower back, or inside the rectum or vagina. The patient controls the timing and intensity of these electrical impulses.

**Key Facts**

**No definitive cure:** IC is considered a chronic condition; treatments aim at symptom management rather than eradication.

**Variable course:** Symptoms may improve, worsen, or even disappear spontaneously in some patients. About **50% of cases may resolve over time** without clear explanation.

**Quality of life impact:** Severe IC can lead to urinating dozens of times per day and is recognized as a disability in some countries.

**️ Common Treatment Approaches**

**Dietary modification:** Avoid bladder irritants like caffeine, alcohol, citrus, spicy foods, and artificial sweeteners.

**Bladder training:** Timed urination schedules to reduce frequency.

**Oral medications:**

Pentosan polysulfate sodium (Elmiron) — the only FDA-approved oral drug for IC.

Tricyclic antidepressants (e.g., amitriptyline), antihistamines, NSAIDs for pain relief.

**Bladder instillations:** DMSO or other solutions delivered directly into the bladder.

**Nerve stimulation:** TENS or sacral nerve stimulation to reduce urgency and pain.

**Physical therapy:** Pelvic floor therapy to relieve muscle tension and pain.

**Surgery:** Rarely used, only for severe refractory cases; outcomes are limited and pain may persist.

**️ Risks & Limitations**

**Side effects:**Elmiron has been linked to eye problems (macular disease), requiring regular eye exams.

**Temporary relief:** Procedures like bladder distention often provide only short-term benefit.

**Trial and error:** No single therapy works for everyone; patients often need to experiment with combinations.

**Psychological impact:** Chronic pain and urinary urgency can cause stress, anxiety, and reduced quality of life. Stress management strategies (yoga, counseling, meditation) are often recommended.

**Takeaway**

Interstitial cystitis is **not curable**, but many people achieve meaningful relief through a mix of lifestyle changes, medications, and therapies. Some even experience spontaneous remission. If you or someone you know is dealing with IC, working closely with a urologist to tailor treatment is essential.

Would you like me to outline the **latest experimental therapies** being studied, such as stem cell approaches or new biologics?

**35.What is the main goal of interstitial cystitis treatment?**

**The main goal of interstitial cystitis (IC) treatment is not to cure the condition but to *reduce bladder pain, urinary urgency, and frequency*, thereby improving overall quality of life.** Because IC varies widely between patients, treatment focuses on symptom control through a combination of lifestyle changes, medications, and sometimes procedures.

**Primary Treatment Goals**

**Pain relief**: Managing chronic bladder pain and discomfort is the top priority.

**Reduce urgency/frequency**: Helping patients urinate less often and with less urgency.

**Improve bladder function**: Increasing bladder capacity and reducing spasms.

**Enhance quality of life**: Supporting daily activities, sleep, and emotional well-being.

**Common Treatment Approaches**

**Lifestyle changes**: Avoiding bladder irritants (caffeine, alcohol, citrus), staying hydrated, quitting smoking, and stress reduction.

**Bladder training**: Timed voiding to gradually increase bladder capacity.

**Physical therapy**: Relaxing pelvic floor muscles to reduce spasms and pain.

**Medications**: Pain relievers, tricyclic antidepressants (e.g., amitriptyline), antihistamines, or pentosan polysulfate sodium (Elmiron).

**Bladder instillations**: Directly placing medication into the bladder via catheter.

**Neuromodulation**: Electrical stimulation of nerves to reduce pain and urgency.

**Surgery**: Rarely used, only for severe cases when all other treatments fail.

**️ Key Considerations**

**No single cure exists** — patients often need to try multiple therapies or combinations.

**Treatment is individualized** — what works for one person may not work for another.

**Risks vs. benefits** must be weighed carefully, especially for invasive procedures.

**Ongoing reassessment** is essential — ineffective treatments should be discontinued.

**Summary Table**

| **Goal** | **Approach** | **Notes** |
| --- | --- | --- |
| **Pain relief** | Medications, physical therapy, neuromodulation | Chronic pain management similar to other conditions |
| **Reduce urgency/frequency** | Bladder training, nerve stimulation | Improves daily comfort and sleep |
| **Improve bladder function** | Bladder instillation, hydrodistention | May temporarily increase bladder capacity |
| **Quality of life** | Lifestyle changes, stress management, support groups | Emotional and social support is vital |

In short, **the main goal of IC treatment is symptom control and quality of life improvement, not cure**. Would you like me to dive deeper into **medication options**, **lifestyle strategies**, or **procedural treatments**?

**36.How long can interstitial cystitis treatment take to start working?**

**Treatment for interstitial cystitis (IC) often takes weeks to months before noticeable relief, and the timeline varies depending on the therapy. For example, oral medication like pentosan polysulfate sodium (Elmiron) may take 2–4 months for pain relief** and up to **6 months for reduced urinary frequency**, while bladder instillations or physical therapy may show improvement sooner.

**⏱ Typical Timelines for IC Treatments**

| **Treatment** | **Expected Onset of Relief** | **Notes** |
| --- | --- | --- |
| **Oral medications** (Elmiron, amitriptyline, antihistamines) | 2–6 months | Elmiron specifically may take 2–4 months for pain, 6 months for urinary frequency. Other oral meds may act faster but vary widely. |
| **Bladder instillations** (DMSO, lidocaine, heparin) | Several weeks | Often given weekly for 6–8 weeks; maintenance may continue for months. |
| **Physical therapy** | Weeks to months | Pelvic floor therapy can relieve pain and urgency if muscle tension is a factor. |
| **Nerve stimulation** (TENS, sacral nerve stimulation) | Variable | Relief may occur within weeks if effective; permanent devices considered after successful trial. |
| **Bladder distention** | Short-term relief | Some patients notice temporary improvement; repeat procedures may be needed. |
| **Lifestyle changes** (diet, stress reduction, bladder training) | Days to weeks | Avoiding bladder irritants (caffeine, citrus, carbonated drinks) may help quickly. |

**️ Key Considerations**

**No single treatment works for everyone** — IC often requires trial-and-error with multiple therapies or combinations.

**Patience is essential**: Many therapies require consistent use over months before benefits are clear.

**Monitoring is important**: Doctors often reassess every few months and discontinue ineffective treatments.

**Risks exist**: For example, Elmiron has been linked to rare eye disease, so regular eye exams are recommended.

**Practical Tips While Waiting for Relief**

Track symptoms with a **bladder diary** to monitor progress.

Use **dietary adjustments** (avoid caffeine, citrus, alcohol, spicy foods).

Practice **stress management** (deep breathing, gentle exercise).

Build a **support system** with family or IC support groups.

Would you like me to focus more on **medication timelines**, **dietary strategies**, or **physical therapy approaches** for IC?

**37.Why do doctors often try interstitial cystitis treatments step by step?**

**Doctors use a step‑by‑step approach for interstitial cystitis (IC) because no single treatment works for everyone, and therapies vary in effectiveness, side effects, and invasiveness. Starting with the least risky options allows patients to find relief while minimizing harm.**

**Why Treatments Are Layered**

**Individual variability**: IC symptoms differ widely (pain, urgency, frequency), so what helps one patient may not help another.

**Safety first**: Initial treatments are low‑risk (diet changes, stress management, physical therapy) before moving to medications or procedures.

**Evidence strength**: Guidelines emphasize trying therapies with stronger safety/efficacy data first.

**Avoiding overtreatment**: More invasive options (nerve stimulation, bladder surgery) are reserved for severe, refractory cases.

**Shared decision-making**: Doctors reassess regularly, stopping ineffective treatments and adjusting based on patient response.

**Stepwise Treatment Framework**

| **Treatment Level** | **Examples** | **Rationale** |
| --- | --- | --- |
| **Lifestyle & Self-care** | Diet modification (avoid caffeine, citrus, alcohol), stress reduction, bladder training | First-line, minimal risk, empowers patients |
| **Physical Therapy** | Pelvic floor relaxation, connective tissue release | Addresses muscle tension contributing to pain |
| **Oral Medications** | NSAIDs, tricyclic antidepressants, antihistamines, pentosan polysulfate sodium | Target pain, urgency, bladder lining protection |
| **Bladder Instillations** | DMSO, lidocaine, heparin | Direct bladder treatment when oral meds fail |
| **Procedures** | Hydrodistention, Botox injections, nerve stimulation | Intermediate options for persistent symptoms |
| **Surgery** | Bladder augmentation, resection, diversion | Last resort; rarely used due to risks and limited pain relief |

**️ Risks & Trade-offs**

**Medications**: Side effects (e.g., eye risks with pentosan polysulfate).

**Procedures**: Temporary relief, possible need for self-catheterization after Botox.

**Surgery**: High complication risk, may not resolve pain.

**Emotional impact**: Trial‑and‑error can be frustrating; support systems and coping strategies are crucial.

**Key Takeaway**

Doctors proceed step by step in IC treatment to **balance relief with safety**, tailoring care to each patient’s unique symptoms and responses. This cautious escalation helps avoid unnecessary risks while maximizing the chance of finding effective symptom control.

Would you like me to break down the **first-line lifestyle changes** in detail, or explore the **advanced procedures** doctors use when simpler measures fail?

**38.Is it safe to take over-the-counter pain medicine for interstitial cystitis?**

Harvard Health

Credible health information source

**Interstitial cystitis**

Interstitial cystitis is a puzzling bladder condition in which the bladder wall becomes irritated or inflamed, causing pain and frequent or painful urination. The symptoms of interstitial cystitis are often similar to the symptoms of a urinary tract infection. However, in interstitial cystitis, there is no infection, and the symptoms do not respond to antibiotic treatment. The exact cause of interstitial cystitis remains a mystery, although researchers continue to investigate possible causes, such as unidentified bacteria, an allergic or immune system reaction, a toxic substance in the urine, or a neurological problem in the bladder wall. There also is some evidence that interstitial cystitis may not be just one illness, but several illnesses that share similar symptoms. Interstitial cystitis usually occurs between the ages of 20 and 50. Approximately 90% of people with interstitial cystitis are women. It is unknown why interstitial cystitis is more common in women. The disease is not known to be genetic (inherited) or caused by toxins in the environment.

**Over-the-counter pain medicines like acetaminophen (paracetamol) are generally considered safe for people with interstitial cystitis, but NSAIDs (such as ibuprofen or aspirin) may irritate the bladder and worsen symptoms in some patients. It’s best to consult a healthcare provider before using any pain medication regularly, since IC management often requires a tailored approach.**

**Key Points on Pain Relief in Interstitial Cystitis (IC)**

**Acetaminophen**

Often used for general pain relief.

Does not irritate the bladder lining.

Considered safer for IC patients compared to NSAIDs.

**NSAIDs (ibuprofen, aspirin, naproxen)**

Can help with inflammation and pain.

However, many IC patients report bladder irritation and symptom flare-ups.

Should be used cautiously and only under medical guidance.

**Other oral medications** sometimes prescribed for IC include:

Tricyclic antidepressants (e.g., amitriptyline) for pain modulation.

Antihistamines (e.g., hydroxyzine) to reduce bladder irritation.

Pentosan polysulfate sodium (Elmiron®), the only FDA-approved oral drug specifically for IC.

**️ Risks and Considerations**

**Bladder sensitivity**: IC patients often have heightened bladder wall irritation, so even common OTC drugs may worsen symptoms.

**Chronic use**: Long-term NSAID use carries risks (GI bleeding, kidney strain) and may not be ideal for IC patients.

**Individual variability**: Some patients tolerate NSAIDs well, while others experience severe flares. Keeping a symptom diary can help identify triggers.

**Safer Self-Care Approaches**

**Dietary adjustments**: Avoid acidic foods, caffeine, alcohol, and artificial sweeteners, which often worsen IC pain.

**Stress management**: Stress can trigger flares; relaxation techniques or counseling may help.

**Pelvic floor physical therapy**: Can reduce pain and urinary urgency.

**Bladder retraining**: Helps reduce frequency and urgency.

**Bottom Line**

For IC patients, **acetaminophen is usually the safest OTC pain option**, while NSAIDs should be used with caution and only under medical supervision. Because IC varies widely between individuals, it’s important to **discuss pain management with a urologist** to avoid worsening symptoms and to explore more targeted treatments.

Would you like me to outline **non-medication pain relief strategies** or a **comparison of prescription options** next?

**39.Are antibiotics helpful for interstitial cystitis when there is no infection?**

**Antibiotics are *not* helpful for interstitial cystitis (IC) when there is no infection.** IC is a chronic bladder pain syndrome without bacterial involvement, and guidelines explicitly advise against long-term antibiotic use because it lacks efficacy and carries risks.

Harvard Health

Credible health information source

**Bladder infection (cystitis) treatment**

Treatment varies, depending on the type of problem. **Simple bladder infection** - Simple bladder infections in women usually are treated with one of three antibiotics: nitrofurantoin (Macrobid) twice per day for 5 days, trimethoprim sulfamethoxazole (Bactrim, Septra, Co-trimoxazole) or fosfomycin (Monurol) as a single oral dose. Women with recurring bladder infections (more than two per year) may benefit from preventive antibiotics, either taken as a regular dose or after sexual intercourse. **Complicated bladder infection** - Complicated bladder infections are more difficult to treat. The choice of antibiotic, the strength of the medication and the length of treatment vary depending on the circumstances. Often, antibiotics need to be taken for 7 to 10 days. Patients with severe symptoms (fever, confusion, nausea, vomiting, etc.) that suggest the bacteria have spread to the kidney or blood usually will need to be treated in a hospital.

**Why Antibiotics Don’t Work in IC**

**No infection present**: IC symptoms (pain, urgency, frequency) mimic urinary tract infections, but urine cultures are negative. Antibiotics target bacteria, so they don’t relieve IC symptoms.

**Guideline recommendation**: The American Urological Association (AUA) states that **long-term oral antibiotics should not be offered** for IC/BPS (Bladder Pain Syndrome).

**Risks of unnecessary antibiotics**: Side effects (GI upset, allergic reactions), antibiotic resistance, and disruption of normal microbiota.

**Evidence-Based Treatments for IC**

Instead of antibiotics, management focuses on multimodal approaches:

**Lifestyle changes**: Avoid bladder irritants (caffeine, alcohol, citrus, spicy foods).

**Oral medications**: Amitriptyline, hydroxyzine, cimetidine, or pentosan polysulfate sodium (Elmiron).

**Physical therapy**: Pelvic floor therapy to reduce muscle tension and pain.

**Bladder instillations**: DMSO, heparin, or lidocaine directly into the bladder.

**Neuromodulation**: Nerve stimulation techniques for refractory cases.

**Surgery**: Rarely used, reserved for severe, end-stage cases.

**Key Risks & Considerations**

**Misdiagnosis**: Some patients initially receive antibiotics because IC resembles recurrent UTIs. If symptoms persist despite negative cultures, IC should be considered.

**Delayed treatment**: Overuse of antibiotics can delay proper IC management and worsen quality of life.

**Vision risk with Elmiron**: Pentosan polysulfate sodium has been linked to macular eye disease; regular eye exams are recommended.

**Quick Comparison**

| **Treatment** | **Effective for IC?** | **Notes** |
| --- | --- | --- |
| **Antibiotics** | Not effective | Only for true infections, not IC |
| **Dietary changes** | Often helpful | Avoid irritants like caffeine, alcohol |
| **Oral meds (Elmiron, amitriptyline, etc.)** | Variable benefit | Elmiron requires eye monitoring |
| **Bladder instillations** | Moderate relief | DMSO, lidocaine, heparin |
| **Physical therapy** | Strong evidence | Pelvic floor relaxation |
| **Surgery** | ️ Last resort | Rare, for severe cases |

In summary: **Antibiotics should not be used for IC without infection.** If you or someone you know has persistent bladder pain with negative cultures, it’s important to consult a urologist for proper diagnosis and explore evidence-based treatments like oral medications, physical therapy, or bladder instillations. Would you like me to break down the **step-by-step treatment algorithm** doctors follow for IC?

**40.Should someone keep taking antibiotics for interstitial cystitis if urine tests show no infection?**

**No — antibiotics are *not recommended* for interstitial cystitis (IC) when urine tests show no infection. IC is a chronic bladder pain syndrome without bacterial infection, and guidelines explicitly advise against long-term antibiotic use because it is ineffective and carries risks.**

Harvard Health

Credible health information source

**Bladder infection (cystitis) treatment**

Treatment varies, depending on the type of problem. **Simple bladder infection** - Simple bladder infections in women usually are treated with one of three antibiotics: nitrofurantoin (Macrobid) twice per day for 5 days, trimethoprim sulfamethoxazole (Bactrim, Septra, Co-trimoxazole) or fosfomycin (Monurol) as a single oral dose. Women with recurring bladder infections (more than two per year) may benefit from preventive antibiotics, either taken as a regular dose or after sexual intercourse. **Complicated bladder infection** - Complicated bladder infections are more difficult to treat. The choice of antibiotic, the strength of the medication and the length of treatment vary depending on the circumstances. Often, antibiotics need to be taken for 7 to 10 days. Patients with severe symptoms (fever, confusion, nausea, vomiting, etc.) that suggest the bacteria have spread to the kidney or blood usually will need to be treated in a hospital.

**Key Points**

**Interstitial cystitis (IC)** is a bladder pain syndrome with symptoms similar to urinary tract infection (UTI), but **without infection**.

**Antibiotics do not help IC**: Harvard Health notes that IC symptoms *do not respond to antibiotic treatment* because no infection is present.

**Guidelines (AUA, 2022)**: Long-term oral antibiotics should *not* be offered for IC/BPS.

**Risks of unnecessary antibiotics**: Side effects, antibiotic resistance, gut microbiome disruption, and increased risk of secondary infections (e.g., C. difficile).

**Recommended Management Approaches**

Instead of antibiotics, IC treatment focuses on symptom relief and bladder health:

**Lifestyle changes** Avoid bladder irritants (caffeine, alcohol, citrus, spicy foods, artificial sweeteners).

**Oral medications**

Pentosan polysulfate sodium (Elmiron) – FDA-approved for IC

Amitriptyline, hydroxyzine, cimetidine – sometimes used for pain/urgency

NSAIDs for pain relief

**Bladder instillations** DMSO, heparin, or lidocaine delivered directly into the bladder.

**Physical therapy** Pelvic floor therapy to relieve muscle tension and pain.

**Neuromodulation** Electrical stimulation of nerves to reduce urgency and pain.

**Stress management** Relaxation techniques, mindfulness, and coping strategies.

**️ Risks of Continuing Antibiotics Without Infection**

**Resistance**: Bacteria become harder to treat if a real infection occurs later.

**Side effects**: GI upset, allergic reactions, yeast infections.

**Serious complications**: Increased risk of *Clostridioides difficile* colitis.

**Bottom Line**

If urine cultures are negative, **continuing antibiotics for IC is not appropriate**. Management should shift to bladder-specific therapies and pain control. A urologist can help tailor treatment options.

Would you like me to outline the **step-by-step treatment pathway for IC** recommended by the American Urological Association, starting from conservative measures up to advanced therapies?

**41.Why might eye checks be needed for some interstitial cystitis medicines?**

**Eye checks are needed for some interstitial cystitis medicines—specifically *pentosan polysulfate sodium (Elmiron)*—because long-term use has been linked to a rare but serious condition called *pigmentary maculopathy*, which can damage the retina and cause vision problems. Doctors recommend baseline and follow-up eye exams to detect early changes before vision loss occurs.**

**Why Eye Exams Are Required**

**Pentosan polysulfate sodium (Elmiron)**

FDA-approved oral drug for interstitial cystitis.

Works by coating and protecting the bladder lining.

**Risk:** Associated with *macular eye disease* (pigmentary maculopathy), which affects the central retina and can impair reading, night vision, and detail recognition.

**Eye exam protocol**

**Baseline exam** before starting Elmiron.

**Regular follow-ups** (often annually, or sooner if symptoms arise).

Exams include retinal imaging (OCT, fundus photography) to detect subtle changes.

**️ Risks & Symptoms to Watch**

**Vision changes**: Difficulty reading, blurred vision, trouble adapting to dim light.

**Pigmentary maculopathy**: Can progress even after stopping the drug.

**Early detection**: Eye checks help catch changes before irreversible damage occurs.

**Comparison of Common IC Medications**

| **Medication** | **Purpose** | **Eye Risk** | **Monitoring Needed** |
| --- | --- | --- | --- |
| **NSAIDs** (ibuprofen, naproxen) | Pain relief | None | Routine only |
| **Tricyclic antidepressants** (amitriptyline) | Relax bladder, block pain | None | Routine only |
| **Antihistamines** (loratadine) | Reduce urgency/frequency | None | Routine only |
| **Pentosan polysulfate sodium** | Protect bladder lining | **Yes – macular damage risk** | Baseline + follow-up eye exams |

**Practical Guidance**

If prescribed **Elmiron**, ask your doctor about:

**Baseline retinal exam** before starting.

**Annual monitoring** or sooner if vision changes occur.

Alternatives if you already have eye disease.

Report **any new vision problems immediately**—don’t wait for your next scheduled check.

Would you like me to go deeper into **Elmiron side effects**, or explore **alternative IC treatments** that don’t carry eye risks?

**42.Should people with interstitial cystitis talk to a doctor before using supplements?**

**Yes — people with interstitial cystitis (IC) should always consult a doctor before starting supplements.** Certain vitamins, minerals, and herbal products can worsen bladder pain or trigger flares, while others may help. Because IC symptoms vary widely, medical guidance is essential to avoid harmful choices and tailor safe options.

**Why Medical Guidance Matters**

**Bladder sensitivity**: IC involves chronic bladder inflammation; supplements that alter urine acidity or irritate the bladder lining can worsen pain.

**Risk of flare-ups**: High-dose Vitamin C, calcium, potassium, and certain herbs are known triggers.

**Drug interactions**: Supplements like quercetin may interact with antibiotics or blood thinners.

**Individual variability**: What helps one patient may harm another; professional oversight ensures safer choices.

**️ Supplements: Helpful vs Harmful**

| **Supplement** | **Potential Benefit** | **Risks for IC** |
| --- | --- | --- |
| **Quercetin** | Anti-inflammatory, small trial evidence for pain relief | May interact with medications |
| **Glucosamine & Chondroitin** | Support bladder’s GAG layer repair | Slow effect (months), limited oral data |
| **Aloe Vera** | Anti-inflammatory, patient-reported relief | Poorly studied, variable quality |
| **Omega-3 fatty acids** | Reduce systemic inflammation | No direct IC trials |
| **Vitamin D** | Linked to pelvic floor health | Excess can cause toxicity |
| **Vitamin C (high dose)** | Immune support | Acidifies urine, often triggers flares |
| **Calcium (high dose)** | Bone health | May worsen IC symptoms |
| **Potassium supplements** | Essential mineral | Can irritate bladder lining |
| **Cranberry supplements** | UTI prevention | Too acidic, often harmful for IC |

**Risks & Precautions**

**Avoid self-prescribing**: Even “natural” remedies can irritate the bladder.

**Check product labels**: Watch for ascorbic acid, citric acid, caffeine, or artificial preservatives.

**Start low, go slow**: If a doctor approves, begin with small doses to test tolerance.

**Track symptoms**: Keep a journal to identify supplement-related flare-ups.

**Safer Strategies**

Focus on **bladder-friendly foods** (blueberries, pears, lean proteins, whole grains).

Consider **pelvic floor therapy**, stress management (yoga, meditation), and hydration.

Use supplements only under **medical supervision**, ideally with a urologist or IC specialist.

Would you like me to highlight **natural remedies** (like chamomile, marshmallow root, aloe vera) that patients often explore, or focus more on **clinical trial-backed supplements** with stronger evidence?

**43.Can pelvic floor physical therapy help interstitial cystitis?**

**Yes — pelvic floor physical therapy is one of the most effective, evidence-based treatments for interstitial cystitis (IC), especially when pelvic floor dysfunction is present. It is recommended as a first-line therapy by the American Urological Association and has been shown to reduce pain, urinary urgency, and frequency significantly.**

**Why Pelvic Floor PT Helps IC**

**High prevalence of pelvic floor dysfunction**: Up to **85% of IC patients** also have pelvic floor muscle problems such as tightness, spasms, or trigger points. These contribute to bladder pain, urgency, constipation, and painful intercourse.

**Evidence-based relief**: Clinical trials show pelvic floor myofascial release and manual therapy can reduce IC symptoms by **60% or more**, with pain scores dropping from 7.6/10 to 2.6/10 after treatment.

**Guideline support**: The **AUA Guidelines (2022)** list pelvic floor physical therapy as one of only two recommended treatments for IC/BPS, with the strongest evidence grade (“A”).

**What Therapy Involves**

**Manual techniques**: Myofascial release, trigger-point therapy, and connective tissue mobilization (external and internal).

**Relaxation focus**: Unlike incontinence therapy, IC patients should *avoid Kegel strengthening*; instead, therapy emphasizes relaxation, lengthening, and reducing muscle tension.

**Assessment**: Therapists evaluate posture, gait, pelvic alignment, and muscle tension. Internal exams may be used to identify deep pelvic muscle dysfunction.

**Adjunct tools**: Biofeedback, ultrasound, electrical stimulation, and home programs (stretching, relaxation, bladder retraining).

**Comparison: Pelvic Floor PT vs Other IC Treatments**

| **Treatment** | **Evidence Strength** | **Relief Type** | **Limitations** |
| --- | --- | --- | --- |
| **Pelvic floor PT** | Grade A (strongest) | Sustained relief | Requires trained specialist |
| **Oral meds** | Variable, often weak | Temporary symptom control | Side effects, ongoing use |
| **Bladder instillations** | Limited | Local relief | Must be repeated indefinitely |
| **Diet changes** | Moderate | Reduce flares | Individual variability |

**️ Risks & Considerations**

**Not all PTs are trained**: Seek a therapist with expertise in IC/pelvic pain (e.g., APTA Women’s Health, IPPS directories).

**Avoid Kegels initially**: Strengthening can worsen symptoms if muscles are already tight.

**Insurance coverage varies**: Some plans cover “pelvic pain” but not IC/BPS; check your policy.

**Takeaway**

Pelvic floor physical therapy is widely regarded as the **most effective and lasting treatment** for IC, addressing the muscle dysfunction that often drives symptoms. If you’re considering it, look for a **specialized pelvic health PT** and expect gradual but meaningful improvement over several sessions.

Would you like me to share **self-care pelvic floor relaxation techniques** you can try at home while waiting to see a therapist?

**44.Are pelvic floor strengthening exercises always good for interstitial cystitis?**

**Pelvic floor exercises are not always good for interstitial cystitis (IC).** While gentle strengthening and relaxation can help with bladder control and pain, overly aggressive strengthening (like repeated Kegels) may worsen symptoms if pelvic floor muscles are already tight or in spasm. The safest approach is a tailored program guided by a pelvic floor physical therapist.

**Key Points on Pelvic Floor Exercises and IC**

**Pelvic floor dysfunction is common in IC**: Many patients have muscle tension or spasms that contribute to pain and urinary urgency.

**Strengthening vs. relaxation**:

Strengthening exercises (like Kegels) can help bladder support but may aggravate IC if muscles are already overactive.

Relaxation-focused exercises (like diaphragmatic breathing, happy baby pose, gentle stretches) often provide more relief.

**Individual response varies**: Some patients benefit from strengthening, others need relaxation — there is no one-size-fits-all routine.

**Professional guidance is key**: Pelvic floor physical therapy can identify whether your muscles need strengthening, stretching, or relaxation.

**Comparison of Exercise Approaches**

| **Exercise Type** | **Benefits** | **Risks for IC** |
| --- | --- | --- |
| **Kegels** | Improve bladder control, reduce urgency | May worsen pelvic pain if muscles are tight |
| **Relaxation breathing** | Reduces spasms, lowers stress | Minimal risk |
| **Gentle yoga** | Improves flexibility, reduces tension | Avoid poses that strain abdomen |
| **Low-impact cardio** | Boosts circulation, reduces inflammation | High-impact variants can trigger flares |

**️ Risks & Precautions**

**Overdoing Kegels**: Can increase pelvic floor tension and worsen pain.

**Ignoring relaxation**: IC often involves hypertonic muscles, so relaxation is as important as strengthening.

**High-impact exercise**: Running or heavy lifting may trigger bladder pain.

**Diet and lifestyle factors**: Caffeine, alcohol, and acidic foods can worsen IC symptoms, regardless of exercise.

**Practical Guidance**

Start with **gentle, low-impact routines** (walking, stretching, yoga).

Focus on **pelvic floor relaxation** before adding strengthening.

Work with a **pelvic floor physical therapist** to customize exercises.

Keep a **symptom journal** to track which activities help or worsen your IC.

Would you like me to outline a **sample IC-friendly exercise routine** that balances relaxation and gentle strengthening, so you can see what a safe daily plan might look like?

**45.Can bladder training help people with interstitial cystitis urinate less often?**

**Yes — bladder training and pelvic floor muscle exercises can help some people with interstitial cystitis (IC) reduce urinary frequency, though results vary. Evidence shows that combining bladder training with pelvic floor therapy and biofeedback improves bladder control, reduces urgency, and enhances quality of life.**

**How Bladder Training Helps in IC**

**Bladder training** involves gradually increasing the time between urinations to retrain bladder capacity and reduce urgency.

In IC, the bladder lining is inflamed and sensitive, so training alone may not be enough. However, when paired with **pelvic floor muscle training** and **biofeedback**, patients often report fewer trips to the bathroom and better symptom control.

Studies show that **gentle pelvic floor exercises** (like Kegels, pelvic tilts, and bridge pose) strengthen bladder-supporting muscles, helping reduce frequency.

**Evidence from Clinical Studies**

| **Approach** | **Effect on IC Symptoms** | **Notes** |
| --- | --- | --- |
| **Bladder training** | Can reduce urgency & frequency | Works best when combined with other therapies |
| **Pelvic floor muscle training** | Improves bladder control, reduces pain | Often guided by physiotherapists |
| **Biofeedback therapy** | Enhances awareness & correct exercise performance | Shown to improve quality of life in randomized trials |
| **Gentle exercise** (yoga, walking, swimming) | Reduces pelvic tension & inflammation | Avoid high-impact activities that may trigger flares |

**️ Important Considerations**

**Results vary**: Not all IC patients benefit equally; some may find bladder training uncomfortable if pain is severe.

**Multimodal approach**: Experts recommend combining bladder training with medical treatments (oral meds, intravesical therapies) and lifestyle adjustments for best outcomes.

**Professional guidance**: A pelvic floor physical therapist can tailor exercises and bladder training schedules to individual tolerance, reducing risk of flare-ups.

**Next Steps**

If you’re considering bladder training for IC:

Consult a **urologist or pelvic floor therapist** before starting.

Begin with **short intervals** between bathroom visits and gradually extend them.

Pair bladder training with **pelvic floor relaxation techniques** (deep breathing, gentle yoga) to ease tension.

Track symptoms to see if frequency improves without worsening pain.

Would you like me to dive deeper into **pelvic floor exercises**, explore how **biofeedback therapy** works, or outline a sample **bladder training schedule**?

**46.Can bladder medicine placed through a tube help interstitial cystitis symptoms?**

**Yes — bladder medicines placed directly into the bladder through a catheter (called *intravesical instillation*) can help relieve some symptoms of interstitial cystitis (IC), though results vary and this approach is usually considered when oral or lifestyle treatments are insufficient.** These treatments aim to soothe bladder pain, reduce inflammation, and restore the bladder lining, but they are not a cure and effectiveness differs among patients.

**How Bladder Instillation Works**

**Intravesical instillation**: A thin tube (catheter) is inserted into the bladder, and medication is placed inside to coat the bladder lining.

The solution is usually held in the bladder for **15–45 minutes** before being expelled.

Treatments are often repeated weekly for several weeks, with maintenance sessions as needed.

**Common Medications Used**

| **Medication** | **Purpose** | **Notes** |
| --- | --- | --- |
| **Dimethyl sulfoxide (DMSO)** | Anti-inflammatory, pain relief, muscle relaxation | FDA-approved for IC; may cause garlic-like odor temporarily. |
| **Heparin** | Restores bladder lining (glycosaminoglycan layer) | Often combined with lidocaine. |
| **Lidocaine** | Local anesthetic for pain relief | Provides short-term relief. |
| **Hyaluronic acid / Chondroitin sulfate** | Barrier repair | Used in Europe/Asia; less common in U.S. |
| **Cocktail therapies** | Mix of lidocaine, heparin, sodium bicarbonate | Designed to reduce urgency and pain. |

**Effectiveness**

**Evidence strength is moderate to low**: Some patients report significant relief, while others see little benefit.

Relief may last **days to months**, requiring repeat treatments.

Works best for **pain and urgency symptoms**, but not always for frequency.

**Risks & Considerations**

**Temporary discomfort** from catheter use (burning, irritation).

**Urinary tract infection (UTI)** risk due to catheter insertion.

Some medications (like Botox injections during bladder procedures) may require **self-catheterization** afterward if bladder emptying is impaired.

Not all patients respond — treatment is often part of a **multimodal plan** including oral meds, physical therapy, and lifestyle changes.

**Key Takeaway**

Bladder instillation is a recognized option for IC, especially when oral medications or self-care strategies are insufficient. It can reduce pain and urgency, but results vary, and ongoing treatments may be needed. Discussing with a **urologist experienced in IC** is essential to determine if this therapy fits your situation.

Would you like me to compare **oral medications** vs **bladder instillation** in terms of effectiveness and side effects, so you can see which approach might be more suitable?

**47.Is major surgery usually needed for interstitial cystitis?**

**Major surgery is *rarely* needed for interstitial cystitis (IC). It is considered a last-resort option only when all other treatments fail and the bladder is severely damaged or extremely small. Most patients are managed with medications, bladder instillations, nerve stimulation, or lifestyle changes rather than surgery.**

**Typical Treatment Path for IC**

**Lifestyle changes**: Avoiding bladder irritants (caffeine, citrus, alcohol), stress reduction, and bladder training.

**Medications**: Oral drugs like pentosan polysulfate (Elmiron), antihistamines, tricyclic antidepressants, or NSAIDs.

**Bladder instillations**: Direct placement of medications (e.g., DMSO, lidocaine, heparin) into the bladder.

**Nerve stimulation**: Sacral nerve implants or TENS therapy to reduce urgency and frequency.

**Physical therapy**: Pelvic floor therapy for muscle tenderness and pain relief.

**When Surgery Is Considered**

Surgery is **not routine** and is only considered in severe, refractory cases:

**Fulguration or resection**: Minimally invasive removal of Hunner’s ulcers.

**Bladder augmentation**: Adding intestinal tissue to enlarge bladder capacity. Pain often persists, and catheterization may be required.

**Urinary diversion or cystectomy**: Removing or bypassing the bladder. This is extremely rare and may not relieve pain, as IC pain can persist even after bladder removal.

**Laser surgery**: Reserved for patients with Hunner’s ulcers.

**️ Risks and Limitations**

**Persistent pain**: Even after bladder removal, many patients continue to experience pelvic pain due to nerve involvement.

**Complications**: Infection, kidney damage, bowel issues, or need for lifelong catheterization.

**Irreversibility**: Once performed, bladder surgeries cannot be undone.

**Quick Comparison**

| **Treatment** | **Typical Use** | **Effectiveness** | **Risks** |
| --- | --- | --- | --- |
| **Medications** | First-line | Moderate relief | Side effects (e.g., eye issues with Elmiron) |
| **Bladder instillation** | Second-line | Temporary relief | Discomfort, infection risk |
| **Nerve stimulation** | Refractory cases | Reduces urgency/frequency | Device complications |
| **Minor surgery (ulcer removal)** | Hunner’s lesions | Symptom relief | Ulcer recurrence |
| **Major surgery (augmentation/cystectomy)** | End-stage bladder | Rare, last resort | Pain may persist, irreversible |

In summary, **major surgery is not usually needed for interstitial cystitis**. It is reserved for the most severe cases where all other therapies fail. Most patients benefit from less invasive treatments such as medications, bladder instillations, or nerve stimulation. Would you like me to walk you through the **non-surgical treatment options** in more detail, or focus on the **surgical procedures** and their outcomes?

**48.Can interstitial cystitis affect sexual life?**

**Yes — interstitial cystitis (IC) often affects sexual life. Many people with IC experience pain during or after intercourse, reduced desire, and emotional strain, which can limit intimacy and overall relationship satisfaction. Managing symptoms, communicating with partners, and involving healthcare providers are key to maintaining sexual well-being.**

**How IC Impacts Sexual Life**

**Pain during intercourse**: Dyspareunia (pain with penetration) is common due to bladder and pelvic pain.

**Reduced desire**: Chronic discomfort, urinary urgency, and fear of flares can lower libido.

**Difficulty with orgasm**: Some report altered orgasm frequency or insufficient lubrication.

**Emotional strain**: Anxiety, frustration, and fear of intimacy can worsen symptoms and reduce satisfaction.

**Relationship challenges**: Partners may feel disconnected, leading to stress in the relationship.

**Evidence from Research**

| **Finding** | **Impact on Sexual Life** |
| --- | --- |
| **Pelvic pain & urgency** | Makes intercourse uncomfortable or impossible |
| **Dyspareunia prevalence** | Higher in IC patients than general population |
| **Lubrication issues** | Leads to discomfort and reduced pleasure |
| **Psychological distress** | Anxiety and depression linked to reduced intimacy |
| **Qualitative studies** | Women describe “severely limited sexual relationships” and diminished quality of life |

**Coping Strategies**

**Symptom management**: Work with healthcare providers on bladder instillations, medications, or relaxation techniques before intimacy.

**Communication**: Openly discuss physical and emotional needs with partners.

**Alternative intimacy**: Focus on non-penetrative activities (touch, oral/manual stimulation, mutual masturbation).

**Position adjustments**: Side-by-side or standing positions may reduce bladder pressure.

**Professional support**: Certified sex therapists can help couples navigate intimacy challenges.

**️ Risks & Considerations**

**Avoiding intimacy entirely** can worsen emotional isolation and relationship strain.

**Dietary triggers** (like caffeine or acidic foods) may worsen symptoms before sex.

**Unmanaged pain** can lead to long-term avoidance and reduced quality of life.

**Key Takeaway**

Interstitial cystitis can significantly disrupt sexual life, but **symptom control, partner communication, and flexible approaches to intimacy** can help preserve closeness and satisfaction. Would you like me to share practical **tips for couples coping with IC** or focus more on **medical treatments that ease intimacy**?

**49.What can someone do if interstitial cystitis causes pain during sex?**

**If interstitial cystitis (IC) causes pain during sex, the most effective steps involve managing bladder symptoms, adjusting sexual activity to reduce pressure, and working closely with healthcare providers for tailored treatments. Communication with your partner and experimenting with positions, timing, and lubrication can also make intimacy more comfortable.**

**Key Strategies for Managing Pain During Sex with IC**

**Medical support**

Doctors may recommend bladder instillations, oral medications for relaxation, or topical numbing agents before intimacy.

Pelvic floor physical therapy can reduce muscle tension contributing to pain.

Pain management strategies (trigger point injections, nerve blocks) may be considered for severe cases.

**Timing and preparation**

Emptying the bladder before sex often reduces discomfort.

Avoiding dietary triggers (like caffeine, alcohol, acidic foods) before intimacy can help prevent flares.

Planning intimacy during times when symptoms are less severe may improve comfort.

**Alternative intimacy**

Focus on non-penetrative activities such as mutual masturbation, oral stimulation, or extended foreplay.

Adjust expectations—connection and pleasure don’t always require penetration or orgasm.

**Position adjustments**

Side-by-side positions or those that avoid abdominal pressure may reduce bladder irritation.

Experimenting with different angles and positions can help identify what feels least painful.

**Lubrication**

Generous use of lubricants reduces friction and discomfort.

Silicone-based or natural oils (like coconut oil) often last longer and may be gentler.

**Communication and counseling**

Openly discussing needs and limits with your partner helps reduce anxiety.

Certified sex therapists or counselors can provide coping strategies for couples dealing with IC-related intimacy challenges.

**️ Risks and Considerations**

**Symptom flare-ups**: Sexual activity can worsen bladder pain, urgency, or frequency.

**Emotional impact**: Chronic pain often leads to anxiety, frustration, or avoidance of intimacy, which can strain relationships.

**Quality of life**: Studies show over 70% of IC patients report negative effects on their sex lives, highlighting the importance of addressing sexual health in treatment.

**Takeaway**

Managing IC-related pain during sex requires a **multi-pronged approach**: medical treatment, lifestyle adjustments, alternative intimacy practices, and strong communication. Since symptoms vary widely, it’s essential to consult a **urologist or pelvic pain specialist** to tailor strategies to your situation.

Would you like me to share specific **self-care techniques** that can be practiced at home to reduce IC flare-ups before intimacy?

**50.Can interstitial cystitis symptoms get worse after sex?**

**Yes — interstitial cystitis (IC) symptoms often flare after sex.** Sexual activity can trigger increased pelvic pain, urinary urgency, burning during urination, and bladder discomfort that may last hours to days. This is a recognized pattern in IC, and managing triggers can help reduce post-intercourse symptom worsening.

**Why Symptoms Worsen After Sex**

**Bladder pressure**: Physical pressure during intercourse can irritate the bladder wall.

**Pelvic floor tension**: Muscle tightening during sex may exacerbate pain.

**Inflammation triggers**: Friction or bladder stimulation can worsen inflammation.

**Emotional stress**: Anxiety or tension around intimacy can amplify discomfort.

**Common Post-Sex IC Symptoms**

| **Symptom** | **Typical Flare Pattern** |
| --- | --- |
| **Pelvic pain** | Sudden, intense pain compared to gradual flare-ups |
| **Bladder discomfort** | Exacerbated pain and pressure |
| **Urinary urgency** | Sharp increase in frequency and urgency |
| **Burning urination** | More severe burning sensation post-intercourse |

**️ Management Strategies**

**Hydration**: Drink water before and after sex to dilute urine irritants.

**Lubrication**: Use adequate lubricant to reduce friction and irritation.

**Urination timing**: Empty bladder before and after sex to reduce bacterial risk and irritation.

**Warm baths**: Helps relax pelvic muscles and ease pain.

**Pelvic floor therapy**: Can reduce muscle tension and bladder pressure.

**Stress management**: Relaxation techniques (yoga, meditation) may lessen flares.

**️ Important Considerations**

**Sexual intimacy challenges**: IC can strain relationships due to pain and avoidance of sex. Open communication with partners is crucial.

**Medical consultation**: Persistent or severe post-sex flares should be discussed with a urologist. Treatments may include oral medications, bladder instillations, or physical therapy.

**Rule out infections**: While IC is not caused by bacteria, urinary tract infections can worsen symptoms and need medical treatment.

Would you like me to focus more on **self-care strategies** you can try at home, or on **medical treatment options** that doctors may recommend?

**51.What should someone do if interstitial cystitis symptoms worsen during pregnancy?**

**If interstitial cystitis (IC) symptoms worsen during pregnancy, the most important step is to consult both an obstetrician and a urologist promptly. Safe management often relies on non-drug strategies (diet, hydration, pelvic floor therapy, stress reduction), with medications used cautiously and only under medical supervision.**

**Immediate Actions**

**Seek medical review**: Worsening bladder pain, urgency, or frequency should be evaluated to rule out urinary tract infection (UTI), which is more common in pregnancy and can cause complications.

**Coordinate care**: A multidisciplinary approach ensures both maternal comfort and fetal safety.

**Monitor for complications**: IC may increase risks of preterm labor, low birth weight, or pregnancy-induced hypertension, so closer prenatal monitoring is advised.

**Symptom Management Strategies**

**Diet adjustments**

Avoid bladder irritants: citrus, tomatoes, caffeine, carbonated drinks, spicy foods, artificial sweeteners.

Favor bladder-friendly foods: lean proteins, non-citrus fruits (apples, bananas), vegetables (cucumbers, green beans), whole grains, and dairy alternatives.

**Safe pain relief**

Acetaminophen is generally considered safe.

Heating pads (low setting, short duration) can ease pelvic pain.

Hydration dilutes urine and reduces irritation.

**Non-drug therapies**

Pelvic floor physical therapy and bladder training.

Acupuncture, mindfulness, and relaxation techniques.

Prenatal yoga, swimming, or gentle exercise to reduce stress and improve bladder control.

**Medication Considerations**

**Use only under medical supervision**: No IC-specific drug has an FDA pregnancy safety rating of “A.”

**Possible options (with caution)**:

Hydroxyzine (antihistamine) may help with bladder irritation.

Pentosan polysulfate sodium (Elmiron) is used rarely due to limited pregnancy safety data.

Intravesical therapies (lidocaine, heparin) deliver medication directly to the bladder, minimizing systemic exposure.

**Key Risks & Precautions**

**UTIs vs IC flares**: Symptoms overlap; urine culture is essential to distinguish them. Untreated UTIs can cause preterm birth or maternal complications.

**Pregnancy outcomes**: Some studies suggest higher risk of preterm labor, low birth weight, and preeclampsia in women with IC.

**Individual variability**: Some women experience remission during pregnancy, while others worsen—symptom patterns can differ even between pregnancies.

**Takeaway**

If IC symptoms worsen during pregnancy, **medical evaluation is critical** to rule out infection and adjust management safely. Non-drug strategies should be prioritized, with medications used sparingly and only under specialist guidance.

Would you like me to outline **safe daily routines** that can help minimize flare-ups, or focus more on **labor and delivery considerations** for women with IC?

**52.What can someone do if interstitial cystitis causes anxiety?**

**If interstitial cystitis (IC) is causing anxiety, the most effective approach is a combination of medical care for bladder symptoms and psychological strategies such as stress management, relaxation techniques, and therapy. Anxiety is common in IC because chronic pain and disrupted sleep can worsen emotional distress, but evidence shows that mindfulness, psychotherapy, and lifestyle adjustments can significantly improve quality of life.**

Harvard Health

Credible health information source

**Interstitial cystitis**

Interstitial cystitis is a puzzling bladder condition in which the bladder wall becomes irritated or inflamed, causing pain and frequent or painful urination. The symptoms of interstitial cystitis are often similar to the symptoms of a urinary tract infection. However, in interstitial cystitis, there is no infection, and the symptoms do not respond to antibiotic treatment. The exact cause of interstitial cystitis remains a mystery, although researchers continue to investigate possible causes, such as unidentified bacteria, an allergic or immune system reaction, a toxic substance in the urine, or a neurological problem in the bladder wall. There also is some evidence that interstitial cystitis may not be just one illness, but several illnesses that share similar symptoms. Interstitial cystitis usually occurs between the ages of 20 and 50. Approximately 90% of people with interstitial cystitis are women. It is unknown why interstitial cystitis is more common in women. The disease is not known to be genetic (inherited) or caused by toxins in the environment.

**Why IC Triggers Anxiety**

**Chronic pain & urgency**: Constant bladder discomfort and frequent urination can disrupt sleep and daily routines, leading to stress and worry.

**Unpredictable flares**: Symptoms often worsen with triggers like menstruation, stress, or certain foods, creating uncertainty.

**Psychosocial impact**: Studies show high rates of **anxiety and depression** among IC patients, with trauma and emotional distress linked to more severe symptoms.

**Stress & Anxiety Management Strategies**

**Mindfulness meditation**: Proven to reduce pain perception and anxiety; an 8-week mindfulness program improved IC symptoms and emotional well-being.

**Cognitive behavioral therapy (CBT)**: Helps reframe negative thoughts about illness and teaches coping skills.

**Relaxation techniques**: Deep breathing, guided imagery, or self-hypnosis can calm the nervous system.

**Psychotherapy**: Talking with a mental health professional can reduce stress and improve coping.

**Gentle exercise**: Walking, yoga, or stretching can relieve tension and improve mood.

**Support groups**: Sharing experiences with others reduces isolation and anxiety.

**Lifestyle Adjustments**

**Diet tracking**: Avoiding bladder irritants (caffeine, alcohol, citrus, tomatoes, chocolate) can reduce flares and indirectly ease anxiety.

**Sleep hygiene**: Establishing a calming bedtime routine helps counter sleep disruption from frequent urination.

**Stress reduction routines**: Massage, visualization, or biofeedback are reported by IC patients to improve both bladder symptoms and emotional well-being.

**️ Risks & Considerations**

**Untreated anxiety** can worsen IC symptoms by amplifying pain perception and bladder sensitivity.

**Medication overlap**: Some IC treatments (like tricyclic antidepressants) may also help with anxiety, but these should only be prescribed and monitored by a healthcare professional.

**PTSD link**: Up to 42% of IC patients meet criteria for PTSD, which requires specialized mental health support.

**Key Takeaway**

Managing anxiety in IC requires **dual focus**: treating bladder pain with medical therapies and addressing emotional distress with stress management, therapy, and lifestyle changes. Working with both a **urologist** and a **mental health professional**provides the best chance of relief.

Would you like me to dive deeper into **mindfulness techniques**, **diet adjustments**, or **therapy options** for IC-related anxiety?

**53.Can counseling help people cope with long-term interstitial cystitis pain?**

**Yes — counseling can help people cope with long-term interstitial cystitis (IC) pain. While it does not directly treat bladder inflammation, psychological support and stress management are recognized as important parts of a multimodal care plan, since stress and emotional distress often worsen IC symptoms.**

**How Counseling Helps with IC Pain**

**Stress management**: Stress is a known trigger for IC flares. Counseling teaches relaxation techniques (deep breathing, visualization, mindfulness) that reduce symptom exacerbations.

**Coping strategies**: Cognitive-behavioral therapy (CBT) and other approaches help patients reframe negative thought patterns, improving resilience against chronic pain.

**Emotional support**: Living with IC can cause anxiety, depression, or social isolation. Counseling provides validation and tools to manage these feelings.

**Behavioral modification**: Counselors often guide patients in bladder training, pacing activities, and lifestyle adjustments that reduce discomfort.

**Quality of life**: By addressing mental health alongside physical symptoms, counseling improves overall well-being and helps patients feel more in control.

**Clinical Guidelines on Counseling and IC**

The **American Urological Association (AUA)** guidelines emphasize **multimodal pain management**, including stress management and psychological support, as part of IC treatment. Counseling is recommended when pain management is inadequate, often alongside physical therapy and medications.

The **Mayo Clinic** notes that reducing stress through counseling, visualization, and biofeedback can ease IC symptoms.

The **NIDDK (NIH)** highlights building a support system and reducing stress as key lifestyle strategies for coping with IC.

**Risks and Considerations**

**Counseling is supportive, not curative**: It does not reduce bladder inflammation directly but helps manage the psychological burden of chronic pain.

**Individual response varies**: Some patients find counseling highly beneficial, while others may need stronger medical interventions.

**Best used in combination**: Counseling works best when paired with medical treatments (e.g., oral medications, bladder instillations, nerve stimulation) and lifestyle changes.

**Practical Next Steps**

Consider **CBT** or **pain-focused counseling** with a therapist experienced in chronic illness.

Join **support groups** (online or local) to share experiences and reduce isolation.

Discuss with your urologist whether integrating counseling into your IC management plan could improve your quality of life.

Would you like me to outline specific **self-care techniques** (like bladder training, diet adjustments, or relaxation exercises) that can complement counseling for IC pain?

**54.How can interstitial cystitis affect work, sleep, and daily life?**

**Interstitial cystitis (IC) can significantly disrupt daily life — frequent urination, pelvic pain, and sleep disturbances often interfere with work productivity, rest, and social activities. The chronic nature of the condition means its impact extends beyond physical symptoms, affecting emotional well-being and relationships.**

**Impact on Sleep**

**Nighttime frequency**: Many people with IC urinate up to 20–60 times per day, including multiple times at night, leading to fragmented sleep.

**Pain flares**: Pelvic pain or bladder pressure can worsen when lying down, making it difficult to fall or stay asleep.

**Sleep deprivation**: Chronic poor sleep contributes to fatigue, irritability, and reduced concentration.

**Impact on Work**

**Reduced productivity**: Frequent bathroom breaks and pain can interrupt tasks, meetings, and focus.

**Workplace limitations**: Jobs requiring long periods of sitting, standing, or limited restroom access are especially difficult.

**Absenteeism**: Flare-ups may force individuals to miss work or request flexible schedules.

**Career strain**: Some patients report needing to change jobs or reduce hours due to symptoms.

**Impact on Daily Life**

**Social activities**: Outings, travel, or events can be stressful due to the need for frequent restroom access.

**Sexual intimacy**: Pain during intercourse is common, straining relationships.

**Dietary restrictions**: Many patients avoid acidic foods, caffeine, alcohol, and spicy meals to reduce flare-ups.

**Emotional health**: Chronic pain and disrupted sleep often lead to anxiety, depression, or social withdrawal.

**Summary Table**

| **Area of Life** | **Impact of IC** |
| --- | --- |
| **Sleep** | Frequent nighttime urination, pain flares, poor rest |
| **Work** | Reduced focus, absenteeism, career strain |
| **Daily activities** | Limited social life, dietary restrictions, sexual pain |
| **Emotional health** | Stress, depression, anxiety, reduced quality of life |

**️ Risks & Challenges**

**Bladder stiffening**: IC can reduce bladder capacity over time.

**Chronic pain cycle**: Pain, poor sleep, and stress reinforce each other.

**Isolation risk**: Social withdrawal may worsen emotional health.

Would you like me to dive deeper into coping strategies, such as diet adjustments, stress management, and workplace accommodations, or focus more on treatment options like medications, bladder instillations, and physical therapy?

**55.How should interstitial cystitis be managed over the long term?**

**Long-term management of interstitial cystitis (IC), also called bladder pain syndrome, focuses on symptom control rather than cure. The most effective approach is multimodal: combining lifestyle changes, oral or intravesical medications, physical therapy, and sometimes nerve stimulation or surgery if symptoms are severe.**

Harvard Health

Credible health information source

**Interstitial cystitis treatment**

The goal of treatment is to reduce symptoms. No one therapy is so successful that it should always be tried first. Often, a person with interstitial cystitis needs to try several therapies before finding the right combination. The symptoms of interstitial cystitis may go away over time, but there is no therapy that cures the disorder. Treatments include: 1. **Dietary modification:** Caffeinated beverages, alcohol, citrus fruits, spicy foods, and chocolate are just a few in a long list of foods that aggravate interstitial cystitis in some people. Each person needs to discover what she or he needs to avoid; 2. **Bladder training:** In this therapy, patients learn to reduce frequent urination by following a schedule for urinating. While this type of treatment can help reduce the frequency of urination, it usually does not reduce pain; 3. **Oral medications:** Pentosan polysulfate sodium (Elmiron) is the only oral medication specifically approved for the treatment of interstitial cystitis. About 30% of people with interstitial cystitis have fewer symptoms while taking this medication. Side effects, including effects on the eye, have limited its use. There is a long list of other medications that are not approved specifically for interstitial cystitis, but may offer relief. These include ibuprofen (Advil, Motrin, and others); naproxen (Aleve, Naprosyn, and others); aspirin; acetaminophen (Tylenol and others); tricyclic antidepressants, such as amitriptyline (Elavil, others); hydroxyzine (Atarax, Vistaril); methenamine (Hiprex); and phenazopyridine (Pyridium, others); 4. **Bladder distention:** Sterile water is used to stretch the bladder. Most patients feel worse for a couple of weeks after the procedure. After that, 30% to 50% of patients feel better. The beneficial effect tends to be temporary (lasting up to a few months) and the procedure is done under general anesthesia, which carries certain risks; 5. **Bladder instillation** (also called bladder wash): In this procedure, the bladder is filled with a sterile solution containing one of a number of ingredients that work directly on the bladder wall. The solution is put into the bladder through a catheter (hollow tube) that is placed through the urethra. After a variable period of time, the person is instructed to empty his or her bladder. Several active ingredients have been used in this procedure, but DMSO, sodium bicarbonate, steroids, heparin, and topical anesthetics are the ones tried most frequently; of these, only DMSO is FDA-approved for interstitial cystitis. As with other therapies, success is variable. Repeating the procedure risks causing more irritation of the bladder wall and introducing infection; 6. **Electrical nerve stimulation:** Traditionally, this has been done with a device called a TENS (transcutaneous electrical nerve stimulator) unit. Mild electrical impulses are passed into the body through wires placed below the navel, on the lower back, or inside the rectum or vagina. The patient controls the timing and intensity of these electrical impulses.

**Core Principles of Long-Term Management**

**Individualized treatment**: No single therapy works for everyone; patients often need to try multiple options.

**Symptom tracking**: Keeping a bladder diary helps identify triggers and monitor progress.

**Shared decision-making**: Patients and clinicians should regularly reassess efficacy and discontinue ineffective treatments.

**Lifestyle & Self-Care**

**Dietary modification**: Avoid common irritants such as caffeine, alcohol, citrus, spicy foods, and artificial sweeteners.

**Bladder training**: Timed voiding schedules can reduce frequency and urgency.

**Stress management**: Relaxation techniques, yoga, and mindfulness help reduce flare-ups.

**Pelvic floor physical therapy**: Manual therapy to release muscle tension; avoid Kegel exercises which may worsen pain.

**Medications**

**Oral options**:

Pentosan polysulfate sodium (Elmiron) – FDA-approved, but requires eye monitoring due to macular risk.

Amitriptyline, hydroxyzine, cimetidine – used off-label for pain and urgency.

NSAIDs or acetaminophen – for pain relief.

**Bladder instillations**: DMSO (FDA-approved), heparin, lidocaine, or steroid solutions delivered directly into the bladder.

**Botulinum toxin A**: Injected into bladder wall for refractory cases, though may require intermittent catheterization.

**Advanced Therapies**

**Electrical nerve stimulation**:

TENS units for external stimulation.

Sacral nerve stimulation devices for persistent urgency/frequency.

**Bladder distention**: Temporary relief via hydrodistension, though benefits fade after months.

**Surgery**: Rarely used; options include fulguration of Hunner lesions or bladder augmentation, reserved for severe, refractory cases.

**Risks & Considerations**

**No cure exists**; management is about improving quality of life.

**Long-term antibiotics and systemic steroids are not recommended** due to poor efficacy and side effects.

**Eye monitoring is essential** for patients on Elmiron.

**Flare-ups are common**; patients should expect periods of worsening and improvement.

**Key Takeaway**

Long-term IC management requires **a tailored, multimodal plan**: lifestyle changes, oral or intravesical medications, physical therapy, and advanced interventions if needed. Regular reassessment with a urologist is crucial to balance symptom relief with treatment risks.

Would you like me to create a **step-by-step management plan** that organizes these options into first-line, second-line, and advanced therapies?

**56.Should people with interstitial cystitis keep following their care plan after symptoms improve?**

**Yes — people with interstitial cystitis (IC) are generally advised to continue following their care plan even after symptoms improve, because IC is a chronic condition with unpredictable flares. Stopping treatment or lifestyle measures too abruptly can increase the risk of relapse.**

**Why Continuing the Care Plan Matters**

**Chronic nature of IC**: IC (also called bladder pain syndrome) does not have a permanent cure; symptoms often wax and wane over time.

**Preventing flares**: Ongoing adherence to diet, bladder training, and stress management helps reduce the likelihood of painful flare-ups.

**Treatment reassessment**: Guidelines emphasize that care plans should be *periodically reassessed* and adjusted, but not abandoned once symptoms improve. Ineffective treatments can be stopped, but effective ones are usually maintained.

**Typical Components of an IC Care Plan**

| **Care element** | **Role after improvement** |
| --- | --- |
| **Dietary changes** | Continue avoiding bladder irritants (caffeine, citrus, alcohol, spicy foods). Reintroduce cautiously if desired. |
| **Bladder training** | Helps maintain bladder capacity and reduce urgency long-term. |
| **Stress management** | Ongoing relaxation techniques reduce stress-induced flares. |
| **Physical therapy** | Pelvic floor relaxation exercises may continue to prevent recurrence of muscle-related pain. |
| **Medications** | Some oral or bladder-instilled drugs may be tapered if stable, but only under medical supervision. |

**️ Risks of Stopping Too Soon**

**Symptom relapse**: Many patients experience flare-ups if they stop dietary or lifestyle measures.

**Reduced quality of life**: Pain, urgency, and frequency can return suddenly.

**Loss of progress**: Gains from bladder training or physical therapy may diminish if discontinued.

**Professional Guidance**

**Shared decision-making**: The American Urological Association recommends that treatment decisions be made collaboratively, with regular reassessment.

**Tailored tapering**: Some therapies (like medications or bladder instillations) may be reduced or stopped if symptoms remain stable, but this should be done under a doctor’s supervision.

**Monitoring tools**: Symptom diaries or scales can help track whether ongoing measures are still necessary.

**Bottom Line**

Even if symptoms improve, **continuing the care plan — especially lifestyle and self-care strategies — is important to keep IC under control**. Adjustments can be made with your healthcare provider, but stopping entirely often risks recurrence.

Would you like me to focus more on **dietary strategies**, **medication management**, or **self-care practices** for long-term stability?

**57.Should online claims about a complete cure for interstitial cystitis be trusted?**

**No — online claims of a “complete cure” for interstitial cystitis (IC) should not be trusted.** Medical consensus is clear: IC is a chronic bladder pain syndrome with no universally accepted cure, though many treatments can reduce symptoms and improve quality of life. Reliable sources emphasize management, not eradication.

Harvard Health

Credible health information source

**Interstitial cystitis**

Interstitial cystitis is a puzzling bladder condition in which the bladder wall becomes irritated or inflamed, causing pain and frequent or painful urination. The symptoms of interstitial cystitis are often similar to the symptoms of a urinary tract infection. However, in interstitial cystitis, there is no infection, and the symptoms do not respond to antibiotic treatment. The exact cause of interstitial cystitis remains a mystery, although researchers continue to investigate possible causes, such as unidentified bacteria, an allergic or immune system reaction, a toxic substance in the urine, or a neurological problem in the bladder wall. There also is some evidence that interstitial cystitis may not be just one illness, but several illnesses that share similar symptoms. Interstitial cystitis usually occurs between the ages of 20 and 50. Approximately 90% of people with interstitial cystitis are women. It is unknown why interstitial cystitis is more common in women. The disease is not known to be genetic (inherited) or caused by toxins in the environment.

**Why Cure Claims Are Misleading**

**Complex condition**: IC involves bladder pain, urinary urgency, and frequency. Causes are unclear and may include immune, epithelial, or neurological factors.

**No single mechanism**: Because IC likely represents multiple overlapping disorders, a universal cure is scientifically implausible.

**Guideline consensus**: The American Urological Association (AUA) and Mayo Clinic state explicitly that *no treatment eliminates IC completely*.

**Red Flags in Online “Cure” Claims**

**Anecdotal testimonials**: Personal stories without controlled trials.

**Commercial marketing**: Supplements or “miracle” therapies sold directly to patients.

**Lack of peer-reviewed evidence**: No randomized controlled trials (RCTs) or replication.

**Promises of permanent remission**: Legitimate therapies only aim for symptom relief, not eradication.

**Evidence-Based Management Options**

While not curative, these approaches can help:

**Dietary changes**: Avoid acidic foods, caffeine, alcohol.

**Physical therapy**: Pelvic floor relaxation and retraining.

**Medications**: Elmiron (FDA-approved), tricyclic antidepressants, antihistamines, pain relievers.

**Bladder instillations**: DMSO or other agents delivered directly into the bladder.

**Stress reduction**: Counseling, mindfulness, yoga.

**Comparison: Legitimate Treatments vs. “Cure” Claims**

| **Approach** | **Evidence Strength** | **Goal** | **Red Flags** |
| --- | --- | --- | --- |
| **FDA-approved drug (Elmiron)** | Moderate (RCTs, guidelines) | Symptom relief | None |
| **Pelvic floor therapy** | Moderate | Pain reduction | None |
| **Diet modification** | Low–moderate | Trigger avoidance | None |
| **Online “miracle cure” supplements** | None | Claims of eradication | No peer review, marketing-driven |
| **Alternative therapies (acupuncture, imagery)** | Low | Adjunct symptom relief | Often anecdotal |

**️ Key Takeaway**

**There is no complete cure for IC.**

**Trust only peer-reviewed, guideline-backed treatments.**

**Discuss any new therapy with a qualified urologist before trying it.**

Would you like me to walk you through **safe symptom management strategies** or focus on **how to spot scams** more deeply?

**58.Is it safe to diagnose or treat interstitial cystitis only with advice from the internet?**

**It is not safe to rely only on internet advice to diagnose or treat interstitial cystitis (IC).** This condition mimics urinary tract infections and other bladder disorders, but requires careful medical evaluation to rule out serious causes and to tailor treatment. Self-diagnosis or self-treatment from online sources risks mismanagement, worsening symptoms, or overlooking other conditions such as bladder cancer or chronic infections. Always consult a qualified healthcare professional.

Harvard Health

Credible health information source

**Interstitial cystitis**

Interstitial cystitis is a puzzling bladder condition in which the bladder wall becomes irritated or inflamed, causing pain and frequent or painful urination. The symptoms of interstitial cystitis are often similar to the symptoms of a urinary tract infection. However, in interstitial cystitis, there is no infection, and the symptoms do not respond to antibiotic treatment. The exact cause of interstitial cystitis remains a mystery, although researchers continue to investigate possible causes, such as unidentified bacteria, an allergic or immune system reaction, a toxic substance in the urine, or a neurological problem in the bladder wall. There also is some evidence that interstitial cystitis may not be just one illness, but several illnesses that share similar symptoms. Interstitial cystitis usually occurs between the ages of 20 and 50. Approximately 90% of people with interstitial cystitis are women. It is unknown why interstitial cystitis is more common in women. The disease is not known to be genetic (inherited) or caused by toxins in the environment.

**Why Internet-Only Advice Is Risky**

**Symptom overlap:** IC symptoms (pelvic pain, urinary urgency, frequency) resemble those of urinary tract infections, prostatitis, or gynecological issues. Misdiagnosis is common.

**No single test:** IC is diagnosed by exclusion — doctors rule out infections, stones, or cancer through urinalysis, cystoscopy, and sometimes biopsy. Online advice cannot replace these.

**Complex management:** IC has no cure, and treatments vary widely (diet changes, bladder instillations, oral medications, physical therapy). What works for one patient may not work for another.

**Evidence-Based Medical Facts**

**Prevalence:** IC affects **3–8 million women** and **1–4 million men** in the U.S.

**Types:**

*Ulcerative IC* (Hunner’s ulcers, ~5–10% of cases).

*Non-ulcerative IC* (~90% of cases, with bladder wall hemorrhages).

**Complications:** Reduced bladder capacity, sexual intimacy problems, depression, and impaired quality of life.

**Safe Steps to Take**

**Seek medical evaluation:** A urologist can perform cystoscopy, urinalysis, and bladder function tests to confirm IC.

**Collaborative treatment:** Doctors often combine therapies — diet modification, oral medications (e.g., Elmiron, tricyclic antidepressants), bladder instillations, and pelvic floor therapy.

**Lifestyle support:** Stress reduction, bladder retraining, and avoiding trigger foods (caffeine, alcohol, citrus, tomatoes) can help manage flares.

**Key Takeaway**

**Internet resources can provide background knowledge and coping tips, but they cannot replace professional diagnosis and treatment.** If you suspect IC, the safest path is to consult a healthcare provider for tailored evaluation and management.

Would you like me to outline the **diagnostic process**, the **treatment options**, or the **lifestyle changes** that doctors often recommend for IC?

**DeepSeek**

**1.What is interstitial cystitis?**

**Interstitial cystitis (IC)**, also known as **bladder pain syndrome (BPS)**, is a chronic condition characterized by a persistent feeling of pressure, pain, and sometimes discomfort in the bladder and surrounding pelvic region.

Here is a breakdown of what you need to know:

**The Core Problem**

Unlike a typical urinary tract infection (UTI), which is caused by bacteria and treated with antibiotics, IC/BPS is **not caused by an infection**. It is a functional disorder of the bladder wall. The key feature is pain that is directly related to how full the bladder is.

**Pain increases as the bladder fills** (e.g., holding urine for a long time hurts).

**Pain is temporarily relieved after urinating.**

**Common Symptoms**

Symptoms can vary from mild to severe and often come in flares (periods of worsening) followed by remissions.

**Pelvic Pain:** A constant dull ache or sharp stabbing pain in the lower abdomen, urethra, or vagina (in women) or scrotum/perineum (in men).

**Urgency:** A sudden, intense need to urinate right away.

**Frequency:** Needing to urinate very often—sometimes 60+ times a day in severe cases, including waking up many times at night (nocturia).

**Pain with Sex:** Pain during or after intercourse (dyspareunia) is very common.

**What Causes It? (Unknown)**

The exact cause is unknown, but leading theories include:

**A "Leaky" Bladder Lining:** The protective layer (glycosaminoglycan or GAG layer) lining the bladder is defective. This allows irritants in urine (like potassium) to penetrate the bladder wall, causing inflammation and pain.

**Autoimmune Reaction:** The body's immune system mistakenly attacks the bladder.

**Nerve Dysfunction:** Nerves in the pelvis become hypersensitive, sending pain signals from normal bladder filling.

**Mast Cell Activation:** Inflammatory cells release histamine and other chemicals into the bladder wall.

**How Is It Diagnosed?**

There is **no single test** for IC/BPS. Doctors diagnose it by ruling out other conditions (UTIs, kidney stones, endometriosis, bladder cancer). Common steps include:

**Urinalysis/Urine culture:** To rule out infection.

**Cystoscopy:** A camera inserted into the bladder. The doctor looks for **Hunner's lesions** (red, bleeding patches) or **glomerulations** (tiny pinpoint bleeds). *Note: A normal-looking bladder does NOT rule out IC.*

**Potassium Sensitivity Test:** (Used less often now) Instilling potassium into the bladder causes pain if the lining is leaky.

**Hydrodistention:** Stretching the bladder with water under anesthesia to see hidden damage and provide temporary relief.

**How Is It Treated?**

Since there is no cure, treatment focuses on **managing symptoms**.

| **Category** | **Examples** |
| --- | --- |
| **Dietary** | Avoid "The 4 C's": **C**affeine, **C**itrus, **C**arbonated drinks, and **C**hili/spicy foods. Also alcohol, tomatoes, artificial sweeteners, and high-potassium foods (bananas). |
| **Oral Meds** | **Elmiron** (the only FDA drug approved for IC—it helps rebuild the bladder lining), amitriptyline (for nerve pain), hydroxyzine (antihistamine). |
| **Bladder Instillations** | A liquid mixture (e.g., lidocaine, heparin, bicarbonate) is put directly into the bladder via a catheter to soothe inflammation. |
| **Physical Therapy** | Pelvic floor myofascial release is highly effective, as many IC patients have tight, spasming pelvic floor muscles. |
| **Procedures** | **Hydrodistention** (stretches the bladder), laser surgery to remove Hunner's lesions, or neuromodulation (a pacemaker for the bladder nerve). |

**Important Distinction**

**IC/BPS** = Chronic pain related to bladder filling, no infection.

**UTI** = Burning with urination, bacteria present, curable with antibiotics.

**Who Gets It?**

**Gender:** Women are 5-10x more likely to be diagnosed than men.

**Age:** Typically starts in the 30s and 40s.

**Association:** Often overlaps with other chronic pain conditions like fibromyalgia, irritable bowel syndrome (IBS), and chronic fatigue syndrome.

**Bottom Line**

If you have persistent pelvic pain and urinary frequency but keep getting told "your urine culture is negative"—ask your doctor specifically about **interstitial cystitis**. It is not "all in your head," but it does require a specialist (urologist or urogynecologist) to diagnose properly.

**2.Is interstitial cystitis the same as bladder pain syndrome?**

The short answer is yes, they are often used interchangeably, but there's a distinct pathological subtype that complicates this simple equation.

Currently, major health organizations like the National Institute of Diabetes and Digestive and Kidney Diseases (NIDDK) consider Interstitial Cystitis (IC) and Bladder Pain Syndrome (BPS) to be the same condition. However, to understand the full picture, it's helpful to look at the history of the term.

**Historically:** "Interstitial cystitis" was the traditional term, but the International Continence Society (ICS) updated it to "Painful Bladder Syndrome" (PBS), which is essentially synonymous with Bladder Pain Syndrome (BPS).

**As a Unified Term:** Many professional bodies now use the combined term "Interstitial Cystitis/Bladder Pain Syndrome (IC/BPS)" to acknowledge this history while treating the condition as one clinical entity.

The clinical definition for IC/BPS is a chronic, unpleasant sensation (pain, pressure, discomfort) felt in the bladder, along with urinary symptoms like urgency and frequency, lasting for more than 6 weeks without any infection or other identifiable cause. This is the case for most patients.

**The Key Distinction: Hunner-Type vs. Non-Hunner-Type**

The complexity arises because the disease presents in two major subtypes, which are fundamentally different:

**Classic/Hunner-Type IC (Found in ~10% of cases)**: This is the original definition, marked by the presence of Hunner’s lesions (ulcers or inflammation) visible during a cystoscopy (a camera exam of the bladder).

**Non-Hunner-Type IC (BPS, Most Common)**: This represents the majority of patients. In these cases, a cystoscopy typically shows a normal-looking bladder, even though the patient experiences all the symptoms of IC/BPS. This has led some specialists to prefer the term "Bladder Pain Syndrome" (BPS) for these patients.

Distinguishing between these two forms is crucial for treatment. The classic, Hunner-type IC responds very well to specific treatments like cauterizing the lesions, while non-Hunner-type BPS is better managed with a broader, more holistic approach.

I hope this helps to clarify the relationship between these terms. If you have more questions, please feel free to ask.

**3.Is interstitial cystitis the same as a bladder infection?**

**No, interstitial cystitis (IC) is not the same as a bladder infection.**

While they share similar symptoms (pelvic pain, frequent urination, and urgency), the underlying cause is completely different. Here is the breakdown:

**Bladder Infection (Acute Cystitis / UTI)**

**Cause:** Caused by **bacteria** (usually E. coli) that enter the urethra and multiply in the bladder.

**Diagnosis:** Urine tests (urinalysis and culture) will show bacteria and high white blood cell counts.

**Treatment:** Short-term **antibiotics** (cure usually within 3-7 days).

**Reality:** It is an acute, curable infection.

**Interstitial Cystitis (IC/BPS - Bladder Pain Syndrome)**

**Cause:Unknown.** It is a chronic condition, not an infection. Theories include a defective bladder lining (leaky epithelium), autoimmune issues, or nerve dysfunction.

**Diagnosis:** Urine tests are typically **sterile** (no bacteria). Diagnosis requires ruling out infections and other diseases (often via cystoscopy or hydrodistention).

**Treatment:** Diet modification, physical therapy, oral medications (like Elmiron, amitriptyline), or bladder instillations. Antibiotics do not work.

**Reality:** It is a chronic pain syndrome, not an infection.

**The Critical Overlap**

Approximately **50%** of women with IC are initially misdiagnosed with recurrent bladder infections. This happens because:

Both conditions cause pelvic pain and frequency.

IC patients often have **white blood cells** (signs of inflammation) in their urine even without bacteria.

**Important Warning:** If you keep testing negative for bacteria on urine cultures but still have severe bladder symptoms, ask your doctor about IC. Repeatedly taking antibiotics when you don't have an infection can lead to antibiotic resistance and yeast infections.

*Disclaimer: I am an AI, not a doctor. Please see a urologist or urogynecologist for persistent bladder pain.*

**4.Can interstitial cystitis happen even when there is no infection?**

**Yes, absolutely.** In fact, the absence of an infection is one of the defining characteristics of Interstitial Cystitis (IC), also known as Bladder Pain Syndrome (BPS).

Here is the critical distinction:

**Bacterial Cystitis (UTI):** Caused by bacteria. Treated with antibiotics. Tests positive on a urine culture.

**Interstitial Cystitis (IC/BPS):** A chronic condition causing UTI-like symptoms (pain, pressure, urgency) **without** any detectable bacteria or infection.

**Why does this happen if there isn't an infection?**

Researchers believe IC/BPS is not an infection but rather a defect in the bladder wall itself. Common theories include:

**A "Leaky" Bladder Lining (GAG Layer):** The protective coating inside your bladder is damaged. This allows irritants in your urine (like potassium) to seep into the deeper bladder tissues, causing pain and inflammation.

**Mast Cell Activation:** Mast cells in the bladder release histamine and other chemicals, causing inflammation *without* bacteria being present (similar to an allergic reaction).

**Pelvic Floor Dysfunction:** Often, the pelvic floor muscles become hypertonic (chronically tight), mimicking the pain of a bladder infection.

**Nerve Sensitivity (Central Sensitization):** The nerves in the bladder become "dialed up," sending pain signals to the brain even though there is no threat (infection) present.

**Can a past infection trigger IC?**

Yes. Many patients report that their IC started immediately after a severe or treated UTI. The theory is that the bacteria triggered an inflammatory response that never turned off, even after the bacteria were killed, or the infection physically damaged the GAG layer.

**How to tell the difference (without a test)**

Since IC mimics a UTI perfectly, you cannot tell the difference based on symptoms alone. However, a key clue is **urine culture results**:

**UTI:** Positive culture. Symptoms resolve with antibiotics.

**IC:** Negative culture. Symptoms **do not** respond to antibiotics (though symptoms may temporarily fluctuate).

**Important Medical Note:** If you have negative urine cultures but blood in your urine (hematuria), fever, or vomiting, you need further urological evaluation, as this could indicate other conditions like kidney stones or bladder lesions.

***Summary:*** *If you have UTI symptoms but your urine culture comes back "no growth" or "negative," Interstitial Cystitis is one of the most likely explanations.*

**5.Is interstitial cystitis a type of bladder cancer?**

**No, interstitial cystitis (IC) is not a type of bladder cancer.**

While both conditions affect the bladder and can share some similar symptoms (such as pelvic pain and frequent urination), they are fundamentally different diseases:

**Interstitial Cystitis (IC/BPS):** This is a **chronic pain syndrome** or inflammatory condition of the bladder wall. It is **non-cancerous (benign)** and does not turn into cancer. The bladder lining may be damaged or leaky, causing pain and urgency, but the cells themselves are not malignant.

**Bladder Cancer:** This is a **malignant growth** of abnormal cells in the bladder tissue. These cells can grow uncontrollably, form tumors, and potentially spread (metastasize) to other parts of the body.

**Key differences to note:**

| Feature | Interstitial Cystitis | Bladder Cancer |
| --- | --- | --- |
| **Nature** | Inflammatory / Pain syndrome | Malignant tumor |
| **Primary Symptom** | Pain as bladder fills (relieved by voiding) | Painless blood in urine (hematuria) in early stages |
| **Risk of Death** | None from the condition itself | Potentially life-threatening |
| **Progression** | Does not become cancer | Can spread to other organs |

**Important clinical point:** Having interstitial cystitis does **not** increase your risk of developing bladder cancer. However, because some symptoms overlap, a doctor will often perform tests (like a cystoscopy) to rule out cancer before diagnosing IC.

*If you are experiencing bladder symptoms, always see a urologist for a proper diagnosis, as treatment for these two conditions is very different.*

**6.Is interstitial cystitis caused by stress or anxiety?**

This is an excellent and important question, as many people with interstitial cystitis (IC/BPS) notice a strong link between their symptoms and their emotional state.

The short answer is: **Stress and anxiety do not directly *cause* interstitial cystitis, but they are powerful and proven *triggers* that can significantly worsen symptoms and may play a role in its development in someone who is already predisposed.**

Let's break down the difference between "cause" and "trigger."

**What the Research Says: Cause vs. Trigger**

**Is stress/anxiety a direct cause?**
No. The root causes of IC are still being researched, but they are thought to be physical, including:

**A defect in the bladder's protective lining (the glycosaminoglycan or GAG layer):** This allows irritating substances in urine to penetrate the bladder wall.

**Mast cell activation:** These immune cells release histamine and other chemicals that cause inflammation and pain.

**Nerve dysfunction (central sensitization):** The nerves in and around the bladder become hypersensitive, sending pain signals even when there's no obvious injury.

**Autoimmune response:** The body's immune system may mistakenly attack the bladder.

You can have these physical vulnerabilities without ever having significant stress or anxiety. So, stress alone does not create a damaged bladder lining.

**Is stress/anxiety a major trigger?**
**Yes, overwhelmingly.** For most people with IC, stress is one of the top two or three triggers for a symptom flare-up (along with certain foods and hormones).

Here’s the science behind why:

**The Brain-Bladder Connection:** The bladder is densely packed with nerve endings connected to the brain. The brain and bladder are in constant, two-way communication.

**The Stress Response (Fight-or-Flight):** When you feel stressed or anxious, your brain releases stress hormones like cortisol and adrenaline. This response is designed for short-term physical threats, but chronic stress keeps the system activated.

**Inflammation:** Chronic stress keeps your body in a low-grade, pro-inflammatory state. For someone with a sensitive bladder (due to the physical causes above), this extra inflammation is like adding gasoline to a fire.

**Muscle Tension:** Stress causes unconscious clenching of muscles throughout the body, including the pelvic floor. Chronically tight pelvic floor muscles are a major contributor to IC pain, urgency, and frequency.

**Nerve Sensitization:** Stress amplifies the "pain volume knob" in your nervous system. A mild irritation that you might not notice on a calm day becomes a major pain signal on a high-stress day.

**The Vicious Cycle**

A critical aspect of IC is that this relationship becomes a two-way street (a psychoneuromuscular loop):

You have an underlying, physically vulnerable bladder.

A trigger (like a stressful work deadline) causes a flare of pain and urgency.

The pain and urgency cause **significant anxiety** about finding a bathroom, sleeping, traveling, or having sex.

This *new anxiety about the IC symptoms* triggers another stress response, which then worsens the original flare.

So, it's not "all in your head." It's a very real, physical process happening in your bladder and nervous system, but it's being *fueled* by the brain's stress circuits.

**What This Means for You (The Takeaway)**

**Don't blame yourself:** You did not cause your IC by being a stressed or anxious person. You likely have a genuine physical condition in your bladder that is *reactive* to your emotional state.

**Stress management is *treatment*, not a cure:** Because stress is a major trigger, learning to manage it is one of the most effective non-drug treatments available. Techniques like cognitive behavioral therapy (CBT), mindfulness meditation, deep breathing, and gentle exercise (like walking or yoga) can directly reduce flare-up severity and frequency.

**Treat the bladder, calm the nerves:** The most successful treatment plans combine physical therapies (diet changes, medications, physical therapy) with psychological ones (stress reduction, anxiety management).

**Treating anxiety can help IC:** For many patients, getting treatment for an underlying anxiety disorder (with therapy or medication like low-dose amitriptyline, which treats both nerve pain and anxiety) leads to a dramatic improvement in their IC symptoms.

**In a Nutshell**

| **Direct Cause?** | **Major Trigger?** |
| --- | --- |
| No. A physical vulnerability in the bladder wall, immune system, or nerves is the root cause. | Yes. The physiological "fight-or-flight" response directly increases inflammation, muscle tension, and nerve pain in the bladder. |

**Summary:** Think of it like this: A damaged bladder lining is the **kindling**, and chronic stress or anxiety is the **match**. You need the kindling for a fire, but the match is often what makes it burn. Put out the fire by addressing the kindling (bladder treatments) and by learning to handle the matches (stress management).

**7.Does interstitial cystitis usually last for a long time?**

Yes, interstitial cystitis (IC), also known as bladder pain syndrome (BPS), is typically a **chronic, long-term condition**.

Here’s what that usually means in practical terms:

**Duration:** Once symptoms develop, they often persist for months or years. For many people, IC/BPS is a lifelong condition, though symptoms can vary significantly in intensity over time.

**Fluctuating course:** It rarely stays the same. You'll likely experience periods of symptom flares (worsening pain, urgency, frequency) followed by periods of remission (few or no symptoms). These remissions can last weeks, months, or even years.

**Not progressive in the traditional sense:** Unlike some chronic diseases that inevitably get worse over time, IC/BPS does not typically cause progressive damage to the bladder. It also does not lead to bladder cancer or shorten life expectancy.

**Important distinctions:**

**It's not a short-term infection:** IC/BPS is often initially mistaken for a urinary tract infection (UTI), but unlike a UTI, it does not resolve quickly with antibiotics.

**It can be managed:** While there is no standard cure, many people achieve excellent symptom control through a combination of dietary changes, physical therapy, medications, bladder instillations, or other treatments.

**Key takeaway:** Yes, IC/BPS usually lasts a long time, but the severity and impact vary greatly from person to person. Effective management strategies are available, and many people learn to live well with the condition. If you're concerned about new or persistent bladder symptoms, seeing a urologist or urogynecologist is important to rule out other causes and start a long-term management plan.

**8.Can interstitial cystitis symptoms come and go over time?**

Yes, absolutely. In fact, the hallmark of interstitial cystitis (also known as bladder pain syndrome or IC/BPS) is that symptoms almost always **come and go** over time.

Rather than being a steady, daily condition for most people, IC is typically characterized by a pattern of **remissions and flare-ups** (or flares).

Here’s a more detailed breakdown of what that looks like:

**Flare-ups (Exacerbations)**

These are periods when symptoms are active and intense. You might experience:

Increased bladder pressure or pain as the bladder fills.

More frequent and urgent needs to urinate (day and night).

Pain that worsens with certain triggers (see below).

Flares can last for a few hours, days, weeks, or even months. Common triggers include:

**Diet:** Acidic foods (citrus, tomatoes), spicy foods, artificial sweeteners, caffeine, alcohol, and high-potassium foods (bananas).

**Stress:** Emotional stress is one of the most powerful and common triggers.

**Allergies:** Seasonal allergies or reactions to medications.

**Physical activity:** High-impact exercise or prolonged sitting.

**Hormones:** Many women report flares right before their menstrual period.

**Remissions (Quiet Periods)**

These are periods when symptoms are mild or disappear entirely. A remission can feel like you're "cured."

During a remission, you might be able to eat trigger foods or exercise without any pain.

Remissions can last for weeks, months, or, in some cases, even years.

It is common for people to have a long remission after their first-ever flare, leading them to think it was just a one-time urinary tract infection (UTI).

**Long-term Patterns**

The "come and go" pattern can change over the course of years:

**Variable course:** For many, the condition is unpredictable. A year of frequent flares might be followed by a year of almost complete silence.

**Progressive in a minority:** In a smaller group of people (though not the majority), the symptoms may gradually worsen over time, with shorter remissions and longer, more severe flares.

**Improvement over time:** It's also possible for symptoms to become less frequent and less severe as you learn to identify and avoid your personal triggers.

**Important Distinction: This is *not* the same as a UTI**

This fluctuating nature can be very confusing and frustrating. A person might feel fine for months, then suddenly have a terrible flare and assume they have a new urinary tract infection (UTI). However, taking a standard UTI antibiotic will have no effect on an IC flare.

**If you have a known diagnosis of IC, a sudden return of symptoms does NOT automatically mean you have a new infection.** But because IC patients are also prone to real UTIs, it's always wise to get a urine culture to rule out an infection before treating it as a flare.

**What This Means for You**

**Don't panic during a flare.** Remind yourself that it is temporary and will likely pass, just as previous ones have.

**Keep a symptom diary.** Track what you eat, your stress level, and your pain/urinary frequency. This is the best way to identify *your* unique triggers so you can prevent future flares.

**Focus on management, not cure.** Since the pattern is fluctuating, the goal of treatment (diet changes, physical therapy, medication, stress management) is to make the remissions longer and the flares shorter and less intense.

**In summary: Yes, the "come and go" nature of IC is not unusual—it's the most typical pattern. Understanding your personal cycle of triggers and relief is the key to living well with the condition.**

**9.What are the most common symptoms of interstitial cystitis?**

The most common symptoms of interstitial cystitis (IC), also known as bladder pain syndrome, primarily involve a combination of **chronic pelvic or bladder pain** and **persistent urinary issues**.

**Common Symptoms**

The hallmark symptoms of IC typically include:

**Pelvic & Bladder Pain**: The most defining symptom is chronic pain, pressure, or discomfort in the bladder and pelvic region, which can range from mild to severe. This pain typically worsens as the bladder fills with urine and is temporarily relieved after urinating.

**Urinary Urgency & Frequency**: People with IC feel a persistent, urgent need to urinate, often passing only small amounts of urine throughout the day and night (nocturia). In severe cases, this can mean up to 60 trips to the bathroom in a single day.

These are not just physical symptoms; they can significantly disrupt daily life, work, and sleep. As the condition is chronic, symptoms often fluctuate over time, with periods of remission followed by "flares".

**Other Possible Symptoms**

IC can also cause other symptoms, though they may not affect every individual:

**Pain During Sexual Activity**: Pain during intercourse (dyspareunia) is common, particularly for women. Men may also experience pain during or after ejaculation.

**Pain in Adjacent Areas**: Discomfort may also be felt in the lower abdomen, lower back, groin, urethra, or the perineum (the area between the anus and genitals).

**Associated Conditions**: Many people with IC also have other chronic pain conditions, such as **irritable bowel syndrome (IBS)**, **fibromyalgia**, and chronic fatigue syndrome, as well as mood disorders like anxiety and depression.

Because symptoms of IC can closely mimic those of a **chronic urinary tract infection (UTI)**—yet lab tests typically show no bacterial infection—it's essential to consult a healthcare provider for an accurate diagnosis if you experience pelvic pain and changes in urination.

I hope this information helps you understand the condition better. If you have more questions, please feel free to ask.

**10.Can interstitial cystitis cause frequent urination?**

Yes, absolutely. **Frequent urination** (medically known as urinary frequency) is one of the hallmark symptoms of interstitial cystitis (IC), also called bladder pain syndrome.

Here is how IC causes frequent urination:

**Reduced Bladder Capacity:** IC causes chronic inflammation, scarring, and stiffening of the bladder wall. Over time, this prevents the bladder from expanding to hold as much urine as a healthy bladder, so it fills up and triggers the urge to urinate much sooner.

**Sensory Nerve Irritation:** The damaged protective lining (glycosaminoglycan or GAG layer) of the bladder allows irritants in urine to penetrate the bladder wall. This directly irritates sensory nerves, sending false signals to your brain that the bladder is full, even when it contains only a small amount of urine.

**Urgency Leads to Frequency:** IC is defined by a strong, often painful urge to urinate (urgency). To relieve that uncomfortable pressure or pain, people urinate very frequently, sometimes 40-60 times per day in severe cases.

**Key Distinction:** Unlike a urinary tract infection (UTI), IC's frequent urination persists despite having no bacteria in the urine and is typically accompanied by **suprapubic pain or pressure** that gets worse as the bladder fills and improves temporarily after voiding.

**Nocturia is also very common:** Many IC patients wake multiple times at night to urinate, disrupting sleep.

If you are experiencing this, please see a urologist, as IC is a diagnosis of exclusion (they must rule out infection, stones, and cancer first).

**11.Can interstitial cystitis make someone feel an urgent need to urinate?**

**Yes, absolutely.** A persistent, urgent need to urinate is one of the hallmark symptoms of interstitial cystitis (IC), also known as bladder pain syndrome.

Here is how IC creates that sensation:

**It mimics a "full" signal.** IC causes defects or thinning in the bladder's protective lining (the epithelium). This allows irritants in urine to penetrate deeper bladder tissues, triggering nerve endings that constantly send a "must empty now" signal to your brain—even if your bladder is nearly empty.

**It is not psychological.** This urgency is driven by inflammation, nerve hypersensitivity, and mast cell activation in the bladder wall. Patients often describe it as a "brick sitting on the bladder" or a feeling that they cannot hold it for more than a few minutes.

**Key difference from infection.** Unlike a UTI, this urgency occurs **without** bacteria or standard infection findings. Antibiotics do not resolve it.

**Common patterns of urgency in IC:**

**Volume-dependent:** Urgency intensifies as the bladder fills and temporarily improves immediately after voiding.

**Nocturnal urgency:** Waking to urinate many times per night is very common.

**Triggered urgency:** Certain foods (coffee, citrus, tomatoes, alcohol, artificial sweeteners) or activities can suddenly worsen the urgency.

*Note: If you or someone you know is experiencing this, a urologist or urogynecologist can perform diagnostic tests (like cystoscopy or potassium sensitivity test) to differentiate IC from overactive bladder or recurrent UTIs.*

*Disclaimer: This information is for educational purposes and does not constitute medical advice. Please consult a healthcare professional for any urinary symptoms.*

**12.Can interstitial cystitis cause bladder pressure without severe pain?**

Yes, absolutely. In fact, experiencing bladder pressure or discomfort without "severe" pain is a very common presentation of interstitial cystitis/bladder pain syndrome (IC/BPS).

Many people with IC describe their primary sensation as:

A constant feeling of **suprapubic pressure** or heaviness (like a weight in the pelvis)

A sense of **incomplete emptying** or intense bladder fullness, even right after urinating

**Annoying discomfort** rather than sharp, severe pain

Here’s why this happens and what’s important to know:

**Why pressure without severe pain occurs:**
IC isn't just about pain fibers. The condition involves mast cell activation, nerve hypersensitivity, and defects in the bladder's protective lining (the glycosaminoglycan layer). This can trigger sensations interpreted by the brain as **urgency, fullness, or pressure**—not necessarily classic "pain." The discomfort often worsens as the bladder fills and is temporarily relieved by voiding.

**Clinical reality:**

Many patients have **mild to moderate** symptoms that they describe as "irritating" or "uncomfortable" rather than severe

Pressure often correlates with bladder capacity (feeling like you need to go at 100 mL instead of 400 mL)

Some people with confirmed IC on cystoscopy report *zero* pain, only pressure/frequency

**Important distinction from other conditions:**

**UTI:** Typically causes burning, sharp pain, and is accompanied by positive urine culture

**Pelvic floor dysfunction:** Can mimic IC pressure but may improve with physical therapy

**IC without severe pain:** Still requires the same diagnostic workup (ruling out infection, stones, lesions)

**What you should know:**
If you have persistent bladder pressure without infection, it's worth seeing a urologist. IC is a clinical diagnosis, and pressure *is* considered a form of discomfort in diagnostic criteria (e.g., the AUA guidelines include "suprapubic pressure" as a symptom). Treatments like dietary modification, pelvic PT, or medications (amitriptyline, hydroxyzine) can help even in the absence of severe pain.

*Disclaimer: I'm an AI, not a doctor. Persistent bladder symptoms warrant a medical evaluation to rule out other causes like stones, chronic infection, or early malignancy. Please see a healthcare provider.*

**13.Why is interstitial cystitis often hard to diagnose?**

That's an excellent question, as the difficulty in diagnosing interstitial cystitis (IC), also known as bladder pain syndrome (BPS), is a central frustration for both patients and doctors.

In short, **there is no simple, definitive test for IC/BPS.** Unlike a urinary tract infection (which shows up on a culture) or bladder cancer (which a doctor can see during a scope exam), IC/BPS is diagnosed primarily by excluding other conditions and recognizing a specific pattern of symptoms.

Here are the key reasons why this process is so challenging:

**1. It's a "Diagnosis of Exclusion"**

This is the biggest hurdle. Doctors must first rule out other conditions that can cause very similar symptoms, such as:

**Chronic Urinary Tract Infections (UTIs):** Symptoms overlap completely (urgency, frequency, pelvic pain). Doctors rely on sterile urine cultures to rule out an active infection. However, some research suggests a complex role of bacteria, making this distinction blurry.

**Overactive Bladder (OAB):** While OAB causes urgency and frequency, it does not typically cause *pain*. Differentiating the two is critical, but patients can sometimes have both.

**Endometriosis (in women):** This can cause severe pelvic pain that radiates to the bladder, mimicking IC/BPS.

**Bladder Cancer:** Rare, but its early symptoms can be identical. A cystoscopy (camera in the bladder) is often needed to rule this out.

**Kidney Stones, Pelvic Floor Dysfunction, and more.**

This exclusion process takes time, often months or years, requiring multiple doctor visits, urine tests, imaging (like ultrasound or CT scans), and procedures.

**2. Symptoms are Variable and Overlap with Many Conditions**

**Wide Spectrum:** One patient may feel a dull suprapubic pressure relieved by urination, while another experiences searing, catastrophic bladder pain with sexual intercourse.

**Flare-ups:** Symptoms "flare" and then "remit" (sometimes for months). A patient might see a urologist during a pain-free period, leading the doctor to think the problem is minor or resolved.

**Non-Specific Symptoms:** The classic symptoms—urinary frequency (up to 60+ times a day), nocturia (waking to urinate), and urgency—are hallmarks of dozens of bladder conditions.

**3. No Reliable Biomarkers or Imaging**

**No Blood Test:** There is no molecule or antibody in the blood that says "this patient has IC/BPS."

**Normal Imaging:** Standard tests like CT scans, MRIs, and renal ultrasounds will **always appear normal** in IC/BPS. These tests are only useful for ruling out stones, tumors, or structural problems.

**Urine is Normal:** Aside for ruling out infection or blood, a urinalysis in IC/BPS is typically unremarkable.

**4. The Gold Standard Test is Invasive and Not Always Conclusive**

The most specific diagnostic tool is a **cystoscopy with hydrodistention** (stretching the bladder with fluid while looking inside with a camera under anesthesia). Even this has major problems:

**Invasive & Expensive:** It requires anesthesia and carries risks of infection and bladder injury.

**Not Always Positive:** Classic findings (Hunner's lesions or widespread pinpoint bleeding called glomerulations) are only seen in about 50% of patients who otherwise have all the symptoms. Their absence does *not* rule out IC/BPS.

**Delayed Diagnosis:** Because it requires anesthesia and specialist equipment, it's not an initial test. Patients often suffer for years before undergoing it.

**5. Lack of Awareness and Historical Skepticism**

**"It's All in Your Head":** For decades, patients (overwhelmingly women, as 90% of IC/BPS patients are female) with "negative" test results were told their pain was psychological. This stigma persists in some corners of medicine.

**Primary Care Gaps:** Many general practitioners and even some gynecologists are not well-trained in the diagnostic criteria for IC/BPS. They may repeatedly treat for nonexistent UTIs with antibiotics (which won't help) or refer patients to multiple different specialists (urology, gynecology, pain management, gastroenterology) without connecting the dots.

**The Result: A Long and Frustrating Road**

Because of these factors, studies show the average time from symptom onset to a correct diagnosis of IC/BPS is **4 to 7 years**, and patients typically see **5 or more different doctors** before getting the right diagnosis.

**The Modern Diagnostic Approach (How it *is* eventually diagnosed)**

To speed this up, experts now use a **positive clinical diagnosis** rather than pure exclusion. The key is a simple, focused history and exam:

**Pain, pressure, or discomfort** related to the bladder, typically worsening as it fills and improving with urination.

**No evidence of other obvious causes** (like infection or stones).

**Absence of other conditions** that better explain the symptoms.

If you suspect you or someone you know has IC/BPS, the most helpful step is to find a **urologist or a urogynecologist who specializes in pelvic pain or IC/BPS**. They are far more likely to recognize the pattern quickly and avoid the long diagnostic odyssey.

**14.When should someone with possible interstitial cystitis see a doctor?**

If you suspect you have interstitial cystitis (IC), also known as bladder pain syndrome, you should see a doctor **sooner rather than later**, but there are specific red flags that mean you should go **immediately**.

Here is the breakdown of when to seek medical care.

**See a doctor within a few days if:**

**You have persistent pelvic pain or pressure** related to bladder filling (pain that gets better after you urinate).

**You urinate frequently** (more than 8 times in 24 hours) or wake up multiple times at night to urinate.

**You have an urgent need to go** that comes on suddenly and feels hard to control.

**Your symptoms have lasted for more than 4-6 weeks** without an obvious cause (like a known UTI).

**See a doctor immediately (urgent care or ER) if:**

**You see blood** in your urine (pink, red, or cola-colored).

**You have a fever** (over 100.4°F / 38°C) or chills.

**You have severe pain** in your lower back or side (flank pain).

**You cannot urinate at all** (this is a medical emergency).

**You are vomiting** and cannot keep down fluids or medications.

**Why the urgency?** IC is a "diagnosis of exclusion," meaning doctors must rule out more serious conditions first (like infections, kidney stones, or bladder cancer). The earlier you start this process, the sooner you can find relief.

**Do not make this common mistake:**

Many people assume they have a "recurring urinary tract infection (UTI)" and try at-home remedies or leftover antibiotics. **If you have 2-3 negative urine cultures over a few months but still have symptoms, that is the exact moment to see a doctor for an IC evaluation.**

**What to tell your doctor:** Keep a 3-day "bladder diary" beforehand (write down what you drink, how often you pee, and your pain level on a 1-10 scale). This dramatically speeds up diagnosis.

**15.What symptoms with interstitial cystitis should be checked urgently?**

**Disclaimer:***I am an AI, not a doctor. The following information is for educational purposes and does not replace professional medical advice. If you are concerned about your symptoms, please contact a healthcare provider or go to the emergency room.*

While Interstitial Cystitis (IC) is a chronic condition, its symptoms can overlap with serious, life-threatening conditions like kidney infections, stones, or bladder cancer. You should seek **urgent or emergency care** if you experience any of the following "red flag" symptoms:

**1. Fever and Chills**

**Why it’s urgent:** IC alone does **not** cause a fever. A fever suggests a systemic infection (like a kidney infection or urosepsis) that requires immediate antibiotics.

**Action:** Go to urgent care or the ER, especially if accompanied by nausea or vomiting.

**2. Inability to Urinate (Urinary Retention)**

**What it looks like:** You feel a full, painful bladder, and you know you need to go, but only a few drops come out—or nothing at all.

**Why it’s urgent:** This can be a severe IC flare, but it can also be a physical blockage or bladder muscle failure. Acute retention can cause the bladder to stretch or rupture.

**Action:** This requires immediate catheterization (ER).

**3. Visible Blood in Urine (Gross Hematuria)**

**Why it’s urgent:** While microscopic blood is common in IC, **large clots or turning the urine pink/red/brown** can indicate a kidney stone, infection, bleeding disorder, or tumor.

**Action:** See a doctor within 24 hours (or immediately if clots are large enough to block urine flow).

**4. Severe Flank (Side or Mid-Back) Pain**

**What it looks like:** Sharp pain in the lower back, just under the ribs, that radiates to the groin or lower abdomen.

**Why it’s urgent:** IC pain is typically suprapubic (over the pubic bone). Flank pain suggests a kidney stone or kidney infection (pyelonephritis), which can cause sepsis.

**5. Sudden Worsening of Baseline Pain**

**What it looks like:** Your typical IC pain suddenly becomes a "10 out of 10" and does not respond to your usual rescue medications (e.g., AZO, Benadryl, heat).

**Why it’s urgent:** This could be a new problem (ruptured ovarian cyst, appendicitis, diverticulitis, or a stone) rather than an IC flare.

**6. Signs of Sepsis (Whole-body infection)**

**Symptoms:** Confusion or disorientation, rapid heart rate, rapid breathing, shaking chills, extreme fatigue, or feeling like you are going to die.

**Action:** Call 911 or go to the ER immediately.

**When to call your Urologist (Urgent but not ER):**

**Foul-smelling or cloudy urine** (likely a UTI) – needs a culture within 24-48 hours.

**New incontinence** (losing control of your bladder).

**Pain that is getting worse over a week** but is manageable at home.

**Critical Note on UTI Testing:**

People with IC often have **false positive** UTI tests (leukocytes on a dipstick) due to chronic inflammation. However, they are also at high risk for **false negative** rapid tests.

**What to demand:** If you have a change in symptoms plus any of the red flags above, request a **urine culture** (takes 48 hours) and do not rely on a quick dipstick in the office.

**When in doubt:** Because IC mimics appendicitis, kidney stones, and gynecologic emergencies, it is safer to go to the ER for severe pain or fever than to assume it is "just an IC flare."

**16.Should blood in the urine be checked if someone has interstitial cystitis symptoms?**

Yes, absolutely. Any instance of blood in the urine (hematuria)—whether visible to the naked eye or only detected under a microscope—should be evaluated by a healthcare professional, even if you have a known diagnosis of interstitial cystitis/bladder pain syndrome (IC/BPS).

Here’s why this is important:

**IC/BPS typically does NOT cause visible blood.** While some IC/BPS patients may have microscopic traces of blood that come and go, seeing visible blood (red or cola-colored urine) is not a standard symptom of an uncomplicated IC flare. It suggests another, potentially more serious, cause.

**Ruling out other conditions is critical.** The symptoms of IC/BPS (pelvic pain, urgency, frequency) can overlap with other disorders. Blood in the urine specifically raises the need to rule out:

**Urinary tract infection (UTI):** This can mimic or worsen IC symptoms and requires antibiotics.

**Kidney stones:** These can cause pain and bleeding.

**Bladder or kidney cancer:** This is the most important concern. Hematuria is a common first sign of these cancers, and having IC does not lower your risk.

**Your baseline matters.** If you already know you have persistent *microscopic* hematuria (seen only on lab tests) that your urologist has tracked over time, a small change may be less alarming. However, *any new or worsening* hematuria, especially if it becomes visible, needs prompt re-evaluation.

**What you should do:**

**See a urologist** (preferably one familiar with IC/BPS). They will likely order:

A urinalysis and urine culture.

Imaging (like a CT urogram or renal ultrasound).

Cystoscopy (camera exam of the bladder), especially if you are over 35 or have risk factors like smoking.

**Do not assume it’s “just IC.”** Even if you think a flare is causing the bleeding, get it checked to be safe.

**In short: Yes. Blood in the urine is never considered "normal" for IC/BPS. Always get it evaluated to rule out infection, stones, or malignancy.**

**17.Should fever with bladder pain be treated as interstitial cystitis?**

**No, fever with bladder pain should not be treated as interstitial cystitis (IC).**

In fact, fever is a critical "red flag" symptom that suggests a condition distinctly different from interstitial cystitis.

Here is the clinical reasoning:

**Interstitial Cystitis (IC) is afebrile by definition.** IC is a chronic pain syndrome of the bladder without evidence of infection. Patients do not run a fever from IC itself.

**Fever + Bladder Pain = Infection until proven otherwise.** This combination is the classic presentation of **acute bacterial cystitis** that has potentially progressed to a **kidney infection (pyelonephritis)** or a **bladder infection with systemic involvement**.

**Misdiagnosis is dangerous.** Treating this as IC (e.g., with Elmiron, amitriptyline, or hydrodistention) while ignoring an infection allows the bacteria to multiply, leading to sepsis, kidney damage, or hospitalization.

**What you should do immediately:**

**Seek urgent medical care** (within 24 hours; sooner if shaking chills or nausea).

**Request a urinalysis and urine culture.** This is the only way to confirm infection.

**Expect antibiotics.** If an infection is found (positive leukocytes/nitrites), you will need a course of antibiotics (e.g., nitrofurantoin, ciprofloxacin, or Bactrim).

**Do NOT start IC treatments** (e.g., antihistamines, bladder instillations) until infection is ruled out.

**One important nuance:**

A patient *with known IC* can certainly develop a fever from a separate urinary tract infection (UTI). In that case, the fever and increased pain are due to the *new infection*, not an IC flare. The infection must be treated first with antibiotics. Only after the infection is cleared (follow-up urine culture negative) should you return to managing the baseline IC.

**Bottom line:** Fever changes everything. Do not treat this as IC. See a doctor for a UTI workup immediately.

**18.What should someone do if interstitial cystitis pain suddenly becomes much worse?**

I'm sorry you're dealing with a sudden worsening of interstitial cystitis (IC) pain. When pain escalates significantly, it's important to take it seriously—both to manage the flare and to rule out other causes like a urinary tract infection (UTI) or bladder injury.

Here’s what someone should do step by step:

**1. Rule out a medical emergency or infection**

**Check for UTI symptoms:** Fever, chills, cloudy or foul-smelling urine, or burning with urination that feels different from usual IC pain. A UTI can quickly become a kidney infection.

**Consider bladder rupture (very rare but serious):** Inability to urinate despite extreme fullness, severe lower abdominal rigidity, or pain after trauma.

**When to go to the ER:** Fever >100.4°F (38°C), vomiting, inability to pass urine, severe pain unrelieved by your usual rescue meds, or if you’re pregnant.

**2. Use a “rescue” treatment at home (if no emergency signs)**

**Oral rescue agents:** Take your prescribed as-needed medications (e.g., phenazopyridine (Pyridium), Uristat, or urinary analgesics). If you have prescription rescue instillation supplies (e.g., lidocaine/heparin), use as directed.

**Oral baking soda:** Mix 1/2 tsp baking soda in 4 oz water – this can temporarily reduce urinary acidity and burning for some people. (Avoid if on a low-sodium diet.)

**Hydration strategy:** Sip small amounts of very plain water. Don’t chug – that can worsen pain. Avoid all acidic, spicy, or caffeinated drinks.

**3. Apply physical and nervous system soothing**

**Heat or cold:** Place a heating pad on the lower abdomen or between the legs (over clothing). Some patients prefer an ice pack wrapped in a towel.

**Pelvic floor relaxation:** Lie on your back with knees bent and supported (pillows under knees). Try slow belly breathing – pelvic floor spasm often drives sudden IC pain.

**Distraction + calming:** Put on headphones with a guided relaxation or brown noise. Reducing stress signaling helps break the pain-spasm cycle.

**4. Adjust your diet immediately (for 24–48 hours)**

Go on an **emergency bland diet**: Only water (pH >7 if possible), plain white rice, plain cooked potatoes (no pepper, no sauce), and a small amount of plain chicken or tofu.

**Stop all supplements** except those specifically for IC (e.g., quercetin, aloe). Many vitamins are acidic.

**5. Call your urologist or IC specialist**

Request a **short course of rescue instillation** (lidocaine/heparin/DMSO – whatever you’ve used before).

Ask about a **tapered steroid burst** (e.g., prednisone 20mg for 5 days) if inflammation is suspected – this is not first-line but can break severe flares.

In some clinics, **oral ketorolac (Toradol)** is available as a one-time strong NSAID – ask if appropriate.

**6. What to avoid during a severe flare**

No pelvic exams, no strenuous exercise, no constipation (use gentle stool softener if needed – straining worsens pain).

No sex, no tampons, no tight waistbands.

Do not take UTI antibiotics unless a urine culture confirms infection – unnecessary antibiotics can cause yeast or bowel changes that worsen IC.

**7. If pain persists >48 hours despite these steps**

Return to your urologist or an ER with urology backup. Ask for:

Urinalysis with microscopy (look for white blood cells without bacteria – that’s a true IC flare vs infection)

Consider a pelvic ultrasound to rule out stones or retention

For refractory pain: hydromorphone or another short-acting opioid **only** for 1–3 days while rescue therapy is arranged – but this is rare and requires careful prescribing.

**Bottom line:** Sudden severe IC pain is often a flare, but always exclude infection first. Use baking soda, heat, rescue meds, and diet restriction immediately. Contact your urologist within 24 hours for possible instillation or steroid taper. If fever, vomiting, or inability to pee – go to the ER.

*Disclaimer: I'm an AI, not a doctor. This information is not a substitute for professional medical advice, especially for new or worsening severe pain.*

**19.What should someone do if they cannot urinate while having interstitial cystitis symptoms?**

This is a serious symptom that requires **immediate medical attention**. If someone with Interstitial Cystitis (IC) suddenly cannot urinate despite feeling a strong urge and having typical IC pain or pressure, here is exactly what they should do:

**1. Seek Emergency Care Immediately**

**Go to the nearest Emergency Room (ER) or Urgent Care center.** Do not wait to see if it resolves on its own.

**Why this is urgent:** The inability to urinate (urinary retention) can overfill the bladder. In severe cases, this can cause the bladder to rupture or lead to kidney damage.

**What to tell the doctor:** Clearly state: *"I have Interstitial Cystitis, and I feel like my bladder is full, but I cannot pass any urine."*

**2. Do NOT Try These Things**

**Do not force yourself to strain hard.** Straining can worsen IC pelvic floor tension and will not fix a true blockage or retention.

**Do not drastically reduce fluids.** While you may be scared to drink, dehydration will not help. Stop drinking large amounts, but do not become severely dehydrated.

**Do not take pain medication without a doctor's order.** Opioids or strong NSAIDs can sometimes worsen retention.

**3. At the Hospital – What to Expect**

**Bladder Scan:** The ER will use a portable ultrasound to see how much urine is actually in your bladder.

**Catheterization:** If your bladder is significantly full (usually >400-500 mL), they will insert a temporary catheter to drain the urine. This will relieve the pressure and pain almost immediately.

**Rule out other causes:** The doctor will check for a true urinary blockage (stones, stricture), a severe IC flare causing swelling and spasm, or a side effect of medications (antihistamines, muscle relaxants, or pain meds you may have taken).

**4. After the Emergency is Resolved**

Once the bladder is drained, work with a urologist (specialist in IC) to adjust your management plan. Inability to urinate is **not a typical** everyday IC symptom (IC usually causes *frequency* and *pain with urination*, not retention). It suggests:

A severe inflammatory flare causing urethral swelling.

Pelvic floor dysfunction (muscles so tight they clamp the urethra shut).

Hunner's lesions (ulcers) or a bladder stone.

Medication side effects.

**Bottom line:** Complete inability to urinate = **Emergency**. Do not try home remedies like heating pads or pelvic floor relaxation – get to a doctor immediately.

**20.What type of doctor usually treats interstitial cystitis?**

Interstitial cystitis (IC) is typically managed by a **urologist**, a doctor who specializes in conditions of the urinary tract, including the bladder. Many gynecologists and urogynecologists also have experience treating people with IC, especially as the condition is more common in women.

Since IC is a complex chronic condition, treatment often requires an interdisciplinary approach. A primary care physician or a specialist will frequently coordinate a team that may include pelvic floor physical therapists and pain management specialists to address all aspects of the condition.

I hope this helps you understand who to consult for interstitial cystitis. Please remember that I'm an AI, not a doctor, so it's essential to discuss your specific situation with a healthcare professional for an accurate diagnosis and treatment plan.

**21.How do doctors usually diagnose interstitial cystitis?**

Diagnosing interstitial cystitis (IC) can be complex, as there is no single, definitive test for it. Instead, the process is one of exclusion, aimed at confirming its hallmark symptoms—bladder-related pain, pressure, or discomfort—while carefully ruling out other conditions that could be causing the problem.

Here are the primary steps a doctor will take to reach a diagnosis.

**Step 1: Initial Clinical Assessment**

Your doctor will begin with a comprehensive evaluation, which is crucial for making the diagnosis.

**Medical History & Symptom Review**: Your doctor will take a detailed history, focusing on the nature of your pain (location, severity, triggers) and your urinary patterns. They will ask about any history of related health problems.

**Bladder Diary**: You may be asked to keep a "voiding log" or bladder diary for a few days. This involves recording how much you drink, how often you urinate, and the volume of urine each time.

**Physical Examination**: A physical exam is performed to check for other potential causes of your pelvic pain.

For women, this typically includes a **pelvic exam** to assess the pelvic floor muscles and identify any gynecological issues.

For men, it may include a **digital rectal exam** to check the prostate and surrounding structures.

**Step 2: Laboratory Tests to Rule Out Other Conditions**

Since a urinary tract infection (UTI) can cause very similar symptoms, lab tests are the first step in the exclusion process.

**Urinalysis**: This analyzes a urine sample for signs of infection, such as white blood cells, or other abnormalities like blood.

**Urine Culture**: This test identifies specific bacteria to either confirm a UTI or rule it out. A UTI is a common 'confusable disease' that must be excluded.

**Urine Cytology**: In this test, a urine sample is examined under a microscope to check for abnormal cells. This helps to rule out bladder cancer, which is another condition that can mimic IC.

**Step 3: Specialized Diagnostic Procedures**

If the initial assessment and lab tests don't point to another cause, your doctor may recommend more specialized procedures. The use of these tests has been updated in recent years.

**Cystoscopy**: This is a key procedure where a thin, flexible tube with a tiny camera (a cystoscope) is inserted through the urethra to view the inside of your bladder. Its primary role in diagnosing IC is not to confirm the condition but to **rule out other disorders**, such as bladder cancer, and to look for specific lesions called **Hunner's lesions** or ulcers, which are a hallmark of a specific subtype of IC.

A 2022 clinical guideline noted that cystoscopy is **not considered useful for routine, uncomplicated presentations**, but it is often recommended for more complex cases or to exclude other pathologies.

**Urodynamic Testing**: These tests measure how well your bladder is storing and releasing urine. Like cystoscopy, current guidelines generally recommend that urodynamics **should not be used in routine care** for diagnosing uncomplicated IC, though they may be considered for more complex presentations.

**Step 4: Other Diagnostic Tools**

**Potassium Sensitivity Test (PST)**: This test involves instilling water and a potassium chloride solution into the bladder to see if the potassium causes significantly more pain or urgency, which would suggest a damaged bladder lining.

**CRITICAL NOTE**: Major clinical guidelines from both the American Urological Association (AUA) and the Canadian Urological Association (CUA) **recommend against** using the potassium sensitivity test for diagnosis. This is because its results are not considered specific enough, the test can be painful, and it may trigger a severe flare of symptoms.

**Ultrasound**: A pelvic ultrasound is an optional imaging test that can be used to rule out other problems in the pelvic region, such as masses or kidney stones.

**Biopsy**: Occasionally, during a cystoscopy, a small sample of bladder tissue (a biopsy) may be removed and examined under a microscope. This is not a standard part of diagnosis but may be done primarily to **rule out bladder cancer** or other rare causes of bladder pain.

**Summary of Diagnostic Process**

The diagnosis of IC is a systematic process of gathering information and excluding other possibilities.

| Step | Purpose | Key Point |
| --- | --- | --- |
| **Step 1: Initial Assessment** | Gather symptom history, rule out common causes | Includes medical history, physical exam, and bladder diary. |
| **Step 2: Lab Tests** | Exclude infection and cancer | Urinalysis, urine culture, and urine cytology are standard. |
| **Step 3: Specialized Procedures** | Visualize bladder, exclude complex conditions | Cystoscopy and urodynamics are not for routine use. |
| **Step 4: Other Tests** | Optional, to support diagnosis or exclude other diseases | Includes PST (not recommended), ultrasound, and biopsy. |

Ultimately, after a careful evaluation that has ruled out all other possible causes for your symptoms, a doctor may diagnose you with interstitial cystitis. This process often involves working with a specialist, typically a urologist.

I hope this overview helps you understand the diagnostic journey. If you have specific symptoms, consulting with a healthcare professional is the most important step.

**22.Is there one test that can prove someone has interstitial cystitis?**

No, there is no single, definitive test that can prove someone has interstitial cystitis (IC). It is considered a diagnosis of exclusion, meaning a doctor reaches a conclusion by ruling out other conditions with similar symptoms.

**Why It's a Diagnosis of Exclusion**

A diagnosis of IC is based on a comprehensive process, not a single positive test result. This is because:

**Symptoms Mimic Other Conditions:** The primary symptoms of IC—pelvic pain, urinary urgency and frequency, and pain during sex—are also common in other disorders.

**No Universal Biomarker:** There is currently no specific biological marker (e.g., a particular chemical in the blood or urine) that can be measured to confirm IC.

The core of the diagnostic process is to systematically rule out other causes of the symptoms before concluding they are due to IC.

**Key Tests Used in the Diagnostic Process**

Doctors use several tests, but they serve specific, primarily exclusionary, purposes:

**Medical History and Symptom Tracking**: This is the foundational first step. You will be asked to describe your symptoms and may be asked to keep a **bladder diary** to track fluid intake and urination patterns, which helps identify characteristic IC symptoms and eliminate other possibilities.

**Urine Tests**: **Urinalysis** and **urine culture** are essential to check for infection, blood, or abnormal cells. This is critical to rule out a common **urinary tract infection (UTI)** as the cause of your symptoms.

**Cystoscopy**: A doctor uses a thin, camera-equipped tube (cystoscope) to look inside the bladder. This test is primarily used to **rule out other conditions**, such as bladder cancer or other structural problems.

**Potassium Sensitivity Test**: This test is generally **NOT recommended** by major urological guidelines for routine diagnosis due to its poor accuracy (low sensitivity and specificity). While it can be informative, its results are not definitive and can lead to false positives.

**How IC is Diagnosed Today**

A modern diagnosis is reached when the following conditions are met:

Your primary symptom is chronic pelvic pain related to your bladder.

This pain is accompanied by persistent urinary urgency or frequency.

Doctors have performed a standard diagnostic workup, including the history, physical exam, and urine tests mentioned above.

Other conditions with similar symptoms, such as UTIs, kidney stones, endometriosis (in women), and bladder cancer, have been ruled out.

If your symptoms align with the first two points and other common conditions are ruled out by the third, your doctor can confidently diagnose you with interstitial cystitis.

I hope this clarifies the diagnostic process for you. If you have more questions, please feel free to ask.

**23.Why do doctors need to rule out other problems before diagnosing interstitial cystitis?**

This is an excellent question, as it gets to the heart of how doctors approach complex, poorly understood conditions.

The simple answer is: **There is no definitive test for interstitial cystitis (IC).** No blood test, scan, or biopsy can say with 100% certainty, "Yes, this is IC."

Therefore, doctors must use a *process of elimination*, also called a **diagnosis of exclusion**. They must systematically rule out all other known diseases that can cause the same symptoms (pelvic pain, urinary frequency/urgency, pressure) before concluding it's IC.

Here are the critical reasons why this step is non-negotiable:

**1. Many Other Conditions Mimic IC Perfectly**

The symptoms of IC are very "non-specific," meaning they overlap with dozens of other, often more common or more dangerous, conditions. Ruling them out prevents a misdiagnosis. Key mimics include:

**Urinary Tract Infection (UTI):** This is the #1 mimic. A standard UTI causes identical symptoms (burning, urgency, frequency). Treating a chronic, low-grade infection as IC would fail, and missing an infection can lead to kidney damage.

**Bladder Cancer (especially Carcinoma in Situ):** This is the most critical reason. Early bladder cancer can cause pelvic pain and urinary urgency *without* visible blood in the urine. Mistaking cancer for IC would be a catastrophic and life-threatening error.

**Kidney Stones:** A stone lodged near the bladder outlet can cause severe suprapubic pain and urinary frequency.

**Endometriosis (in women):** Endometrial lesions on or near the bladder surface cause cyclical or constant pelvic pain and urinary symptoms that are nearly identical to IC.

**Overactive Bladder (OAB):** OAB causes the hallmark IC symptom of urinary urgency/frequency but without the *pain*. The treatment is completely different (muscle relaxants vs. bladder coating therapies).

**Pelvic Floor Muscle Dysfunction:** Extremely tight or spasming pelvic floor muscles can refer pain to the bladder and create a powerful sense of urgency. This is treated with physical therapy, not IC medications.

**Chronic Prostatitis (in men):** Can cause pelvic pain and urinary symptoms indistinguishable from IC.

**2. To Avoid Ineffective and Potentially Harmful Treatments**

Treatments for IC are not benign. They can include:

**Long-term, powerful medications:** Like amitriptyline (antidepressant), hydroxyzine (antihistamine), or pentosan polysulfate (which carries a risk of a unique form of retinopathy).

**Invasive bladder installations (DMSO, lidocaine, heparin):** Putting a catheter in and instilling medication is uncomfortable and carries an infection risk.

**Surgery (e.g., bladder hydrodistention, or rarely, cystectomy):** These procedures have real risks of bleeding, infection, scarring, and for cystectomy, permanent urinary diversion.

You would not want to undergo these treatments for a condition you don't actually have, especially if the real problem is as simple as pelvic floor physical therapy or antibiotics for a chronic infection.

**3. The Diagnosis of Exclusion is an Active, Not Passive, Process**

It’s a myth that doctors just "give up" and call it IC. The official diagnostic process, recommended by the American Urological Association, is an active hunt to rule out specific things in a logical order:

| **Step** | **What it Rules Out** |
| --- | --- |
| **1. Urinalysis & Urine Culture** | Standard UTI |
| **2. Urine Cytology** | Bladder cancer (looking for abnormal cells) |
| **3. Cystoscopy** (camera into bladder) | Bladder cancer, stones, Hunner's lesions (a visible subtype of IC). |
| **4. Symptom & voiding diary** | Overactive bladder, low fluid intake. |
| **5. Pelvic exam (women) / Prostate exam (men)** | Endometriosis, pelvic floor tension, prostatitis. |

Only after these tests are *normal* or *negative*—and symptoms persist for 6+ weeks—will a doctor confidently diagnose IC.

**The Bottom Line for Patients**

Being a "diagnosis of exclusion" can be frustrating. It means you might undergo several tests that come back "normal," which can feel like the doctor isn't finding anything. **But remember: "normal" test results are actually positive findings.** They successfully rule out cancer, infection, and stones, moving you one step closer to the correct diagnosis of IC.

The process isn't about doubting your pain; it's about making absolutely sure you don't have a different, treatable, or life-threatening disease before you start a long-term management plan for a chronic condition.

**24.Why do doctors check urine when interstitial cystitis is suspected?**

This is an excellent question because it gets at a key part of how Interstitial Cystitis (IC) is diagnosed.

The short answer is: **Doctors check urine primarily to *rule out* other conditions that mimic IC, not to *confirm* IC itself.**

There is currently no simple urine test that can definitively diagnose IC. It is a diagnosis of exclusion, meaning a doctor must first eliminate all other likely causes of your symptoms.

Here’s the breakdown of what doctors are looking for when they analyze the urine of someone with suspected IC:

**1. The Most Important Test: Ruling Out Infection (UTI)**

The symptoms of a standard Urinary Tract Infection (UTI) – urgency, frequency, and burning pelvic pain – are almost identical to those of IC. However, UTIs are caused by bacteria and are curable with antibiotics.

**What they look for:** Bacteria, white blood cells (pus), and nitrites.

**The goal:** If they find bacteria, you don't have IC; you have a UTI. Treating the infection should resolve the symptoms.

**2. Looking for Blood (Hematuria)**

Microscopic amounts of blood in the urine (not visible to the naked eye) are common in IC patients, but they are also a sign of other conditions.

**What they look for:** Red blood cells (RBCs).

**The goal:** While the presence of RBCs supports a bladder issue like IC, it could also indicate:

**Kidney stones:** Often cause sharp pain and bleeding.

**Infection:** As mentioned above.

**Bladder or Kidney Cancer:** This is a critical rule-out, especially in older patients or those who smoke. A simple urine test is a non-invasive first step in this investigation.

**3. Checking for Abnormal Cells (Cytology)**

If the urinalysis is persistently abnormal (especially with blood but no infection), or if the patient has risk factors, the doctor may send a urine sample for a "cytology" test.

**What they look for:** Cancerous or pre-cancerous cells shed from the lining of the bladder.

**The goal:** To definitively rule out bladder cancer as the cause of the symptoms.

**An Important Clarification on "IC-Specific" Urine Tests**

You may encounter information about a urine test for **biomarkers** (like **APF** (Antiproliferative Factor), **EGF** (Epidermal Growth Factor), or **HB-EGF** (Heparin-Binding Epidermal Growth Factor-like Growth Factor)). Research has shown that levels of these substances differ in IC patients compared to healthy people.

**However, these tests are not yet standard in clinical practice.** They are largely used in research settings. Most urologists will rely on the standard urinalysis and the patient's symptom history to make a diagnosis.

**Summary: The Doctor's Thought Process**

**Patient:** "I have pelvic pain and need to urinate 30 times a day."

**Doctor (thinking):** "This sounds like IC, but also sounds exactly like a chronic UTI, kidney stones, or a bladder tumor."

**Doctor (action):** "First, let's check a urine sample."

**If infection found**  Diagnose UTI. Not IC.

**If blood found**  Investigate further (cystoscopy, imaging) for stones or tumors.

**If the urine is perfectly normal** (no infection, no significant blood, no abnormal cells) This is the most common finding in IC patients. The clean urine report, combined with the patient's symptoms, is a strong piece of *supporting evidence* for an IC diagnosis.

**In a nutshell: In suspected IC, a urine test is a detective's tool to eliminate the usual suspects, allowing the doctor to confidently focus on the more complex diagnosis of Interstitial Cystitis.**

**25.Is looking inside the bladder always needed to diagnose interstitial cystitis?**

No, looking inside the bladder (cystoscopy) is **not always needed** to diagnose interstitial cystitis/bladder pain syndrome (IC/BPS). While it has traditionally been a key diagnostic tool, modern clinical guidelines have shifted their approach. For many patients with straightforward symptoms, a reliable diagnosis can be made based on a detailed history, physical exam, and basic lab tests, without the need for invasive procedures.

**Evolving Diagnostic Guidelines**

The role of cystoscopy in diagnosing IC/BPS is a subject of professional discussion, with slightly different recommendations from leading urological organizations:

**American Urological Association (AUA)**: The AUA recommends **against** the routine use of cystoscopy for uncomplicated cases. Instead, the diagnosis should be based primarily on the patient's symptoms and the process of ruling out other conditions. Cystoscopy and urodynamics are considered only for complicated presentations, when the diagnosis is uncertain, or when Hunner's lesions are suspected.

**Canadian Urological Association (CUA)**: The CUA maintains a more traditional stance, recommending cystoscopy for most patients to rule out other conditions (like bladder cancer), identify specific bladder lesions, or evaluate bladder capacity. It is considered optional only for young women with no risk factors for other bladder conditions. However, this approach is not universally endorsed.

**When Might Cystoscopy Be Recommended?**

Despite not being always necessary, a cystoscopy is often considered in the following situations:

**Complicated presentations**: Cases involving incontinence, visible blood in the urine (hematuria), or symptoms that overlap with other conditions like overactive bladder or gastrointestinal disorders.

**Diagnostic uncertainty**: When initial assessments and less invasive tests fail to provide a clear diagnosis.

**Suspicion of Hunner's lesions**: These are specific inflammatory patches found in the bladder lining in a small subset (5-20%) of IC patients and require cystoscopy for detection.

**Ruling out other conditions**: To exclude other potential causes of symptoms, such as bladder cancer or bladder stones.

**Important Clinical Considerations**

**Diagnosis of Exclusion**: IC remains a diagnosis made by excluding other conditions that could cause similar symptoms, such as urinary tract infections, endometriosis, or bladder cancer.

**Lack of Specific Findings**: No specific or pathognomonic findings exist for IC. Cystoscopy does not always reveal abnormalities, and findings like glomerulations (small bladder hemorrhages) can be seen in people without IC. Conversely, some people with IC will have a normal-looking bladder lining.

**Summary**

For many patients, especially those with uncomplicated presentations, invasive cystoscopy is not required for a diagnosis of IC/BPS. A clinical diagnosis based on symptoms, medical history, and basic tests is considered appropriate and recommended. The decision to proceed with cystoscopy should be made by a urologist based on the complexity of the individual case and the need to rule out other serious conditions.

If you have further questions about IC/BPS diagnosis or treatment, I recommend consulting a healthcare professional for personalized medical advice.

**26.Can food or drinks make interstitial cystitis symptoms worse?**

Yes, certain foods and drinks can trigger symptoms like pain and urinary urgency for many people with Interstitial Cystitis (IC) / Bladder Pain Syndrome (BPS). However, triggers vary greatly from person to person, so identifying your specific ones is key to managing the condition.

**Common Dietary Triggers**

These items are commonly reported to worsen symptoms:

**Acidic Foods & Drinks**: Citrus fruits (oranges, grapefruit), tomatoes, and pineapple.

**Caffeinated & Carbonated Beverages**: Coffee, tea, soda, and energy drinks.

**Alcohol**: All types, including wine, beer, and liquor.

**Spicy Foods**: Chili peppers, hot sauce, curry, and horseradish.

**Other Potential Triggers**: Artificial sweeteners, MSG, chocolate, and potassium-rich foods.

**How to Find Your Triggers**

The best way to find your triggers is an **elimination diet**, a short-term eating plan that temporarily removes common triggers to see if symptoms improve. Here’s how to do it:

**Keep a Food and Symptom Diary**: Track everything you eat and drink alongside your symptoms for a week or two to spot patterns.

**Eliminate Triggers**: Remove the most common triggers from your diet for 1-2 weeks to see if symptoms improve.

**Reintroduce Foods Gradually**: Add back one food every few days, watching for any symptom return.

**Expert Insight**: A urologist at the University of Michigan recommends the elimination diet, noting there's no need to adopt an overly restrictive diet unless you find it truly helps you.

**Bladder-Friendly Foods**

Here are some foods many people with IC tolerate well:

**Fruits**: Blueberries, pears, watermelon.

**Vegetables**: Broccoli, carrots, cucumbers, potatoes.

**Proteins**: Chicken, turkey, beef, fish, eggs.

**Grains**: Oats, rice, pretzels, popcorn.

**Dairy & Alternatives**: Milk, cottage cheese, grain drinks.

I hope this gives you a clear starting point for managing your diet. Working with a registered dietitian can be very helpful to create a personalized eating plan that ensures you get all the nutrients you need while identifying your triggers. If you have any more questions, feel free to ask.

**27.Which drinks commonly bother people with interstitial cystitis?**

For many people with interstitial cystitis (IC), certain drinks can trigger a flare-up. The most common ones often include **citrus juices, coffee, tea, carbonated beverages, and alcohol**.

Here’s a more detailed list of drinks that are frequently reported as bothersome, along with some helpful context for managing your diet:

**Common Trigger Drinks**

**Citrus Fruit Juices**: Drinks made from **oranges, grapefruits, lemons, limes, and pineapples** are frequent triggers due to their high acidity. Cranberry juice, despite its reputation for urinary health, is often cited as one of the most common triggers for IC.

**Caffeinated & Decaf Beverages**: This includes **coffee and black or green tea**. Caffeine is a known bladder irritant, but interestingly, even decaffeinated versions can trigger symptoms for many people.

**Carbonated Drinks (Soda)**: All types of soda—cola, non-cola, diet, and caffeine-free—are potential triggers. They combine several potential irritants like carbonation, acidity, caffeine, and artificial sweeteners, which can be particularly problematic.

**Alcoholic Beverages**: **Beer, wine (red and white), and spirits** are all known to cause symptom flares.

**Other Potential Triggers**:

**Drinks with Artificial Sweeteners**: Beverages containing sweeteners like **aspartame (NutraSweet), saccharin (Sweet'N Low), and sucralose** can be triggers for some.

**"Hot" or Spiced Drinks**: Cinnamon tea and other spiced ciders may be an issue.

**High-Vitamin C Drinks**: Some people are sensitive to high doses of vitamin C (ascorbic acid), which can be found in many fortified juices and powders.

**Very Hot or Cold Drinks**: Extreme beverage temperatures have also been known to provoke bladder spasms for some individuals.

**️ Finding Your Personal Triggers**

Because sensitivity to drinks varies from person to person, it's helpful to find what specifically affects you. A few strategies can help:

**Keep a Food & Symptom Diary**: For a few weeks, write down everything you eat and drink, noting any bladder symptoms like pain, urgency, or frequency. This makes it easier to identify specific patterns.

**Try an Elimination Diet**: Start by removing common triggers from your diet for a period of time, typically a few weeks. Then, gradually reintroduce one drink at a time, and monitor your body's reaction for 3–4 days before adding another.

**Don't Restrict Forever**: The goal isn't to eliminate all potential triggers permanently. Once you know your personal triggers, you can work on balancing your diet to avoid unnecessary restrictions.

A dietitian can also provide valuable, personalized guidance. You might find that some drinks are only bothersome in large amounts, while others affect you immediately. The key is to discover what works best for your body.

I hope this information is helpful for managing your IC. If you have any other questions, please feel free to ask.

**28.Can coffee make interstitial cystitis symptoms worse?**

Based on current clinical evidence and patient reports, **yes, coffee is a very common dietary trigger that can significantly worsen the symptoms of interstitial cystitis (IC)**.

This is supported by data indicating that up to 90% of people with IC report sensitivities to certain foods and drinks.

**Why Coffee Can Cause Flares**

There are a few reasons why coffee tends to be problematic:

**Bladder Irritants**: Coffee is high in **acids** and **caffeine**, both of which are well-established triggers that can irritate the bladder lining and directly exacerbate symptoms like pain and urinary urgency.

**Beyond Caffeine**: The potential for a flare isn't just about caffeine. Many patients find that even decaffeinated coffee can be a trigger, suggesting other compounds in the beverage can also cause issues.

**️ How to Manage Coffee in Your Diet**

Navigating dietary triggers is highly individual. What bothers one person might be fine for another, so a personalized approach is key.

**Try an Elimination Diet**: This is the most effective method. The standard approach is to avoid all suspected trigger foods and drinks—including coffee—for a set period, often several weeks. Then, you gradually reintroduce them one at a time to see exactly how your body reacts.

**Be Patient**: The effects can take time. The Interstitial Cystitis Association recommends staying off coffee for several weeks to see if your symptoms improve, as they may not clear up immediately.

**Keep a Detailed Diary**: Track what you eat and drink alongside your symptoms. This can help you identify patterns and pinpoint your specific triggers.

**Explore Alternatives**: While many are sensitive to coffee, some people with IC find they can tolerate low-acid coffee blends or certain types of tea.

**️ Other Common Trigger Foods**

If you find that coffee triggers your symptoms, you might be sensitive to other common irritants as well, including:

**Caffeinated and carbonated drinks** (tea, soda, energy drinks)

**Acidic foods** (citrus fruits like oranges and grapefruit, tomatoes)

**Alcohol** and **spicy foods**

**Artificial sweeteners** and **chocolate**

Because every person's experience with IC is unique, working with a healthcare professional to create a personalized dietary plan can be very helpful.

I hope this information is helpful for you in managing your health. Would you like me to provide some practical tips for getting started with a food diary, or do you have any other questions about IC management?

**29.Does everyone with interstitial cystitis need to avoid the same foods?**

The short answer is **no**. While there are common trigger foods, dietary triggers for Interstitial Cystitis / Bladder Pain Syndrome (IC/BPS) are highly individual.

Here’s what you need to know about why the same diet doesn't work for everyone:

**1. The "Common Triggers" Are Just a Starting Point**

Most IC diet lists include highly acidic foods (citrus, tomatoes, coffee), high-potassium foods (bananas, avocados), and irritants (spicy foods, alcohol, artificial sweeteners). These are the *most likely* offenders, but they aren't universal.

**One person** may be unable to drink coffee without severe pain.

**Another person** may drink coffee daily with no issue but finds that soy or green tea causes a flare.

**2. The "Dietary Matrix" Concept**

In IC, it's rarely about *one* food. Think of it as a "leaky bladder" lining. Your symptoms depend on the **total load** of irritants.

**Example:** You might tolerate a small glass of orange juice. But if you drink orange juice *plus* a spicy curry *plus* a chocolate bar on a day you're stressed or dehydrated, that combined load could trigger a flare.

This is why elimination diets must be personalized.

**3. Why the Variation Exists**

**Bladder lining damage:** Some people have dramatic thinning (Hunner's lesions), making them sensitive to potassium-rich foods. Others have more mast cell activation, making them sensitive to histamine-releasing foods (aged cheese, wine, leftovers).

**Gut-bladder connection:** Food sensitivities can start in the gut. What irritates your digestion (e.g., FODMAPs, gluten) may secondarily irritate your bladder.

**Individual biochemistry:** Your unique nerve sensitivity, hormone levels, and even urine pH affect how you react.

**4. How to Find *Your* Triggers (The Right Way)**

Do not adopt someone else's long restriction list. That can lead to nutrient deficiencies and unnecessary stress.

**Step 1: Start with a true IC "rescue diet" (2-4 weeks)**
Eat only the most benign foods: pears, blueberries, mild squash, rice, oatmeal, eggs, white meat poultry, and filtered water. This calms your bladder.

**Step 2: Test ONE food at a time**
Reintroduce a suspected trigger (e.g., one cup of coffee) for one day. Wait 2-3 days to see if a delayed reaction occurs. Rate your pain/urgency before and after.

**Step 3: Build your personal "safe" list**
You'll likely find you can eat many "forbidden" foods without issue, while a seemingly harmless food (like aged cheese or canned soup with preservatives) is a problem for you.

**Key Takeaway**

**Do not assume you must avoid every food on the common IC lists.** Use those lists only as a hypothesis to test. Many people with IC live normal diets with just 1-3 specific triggers avoided—and those triggers vary wildly from person to person.

If you're newly diagnosed, work with a urologist or an IC-knowledgeable dietitian. The goal is the *least restrictive diet* that keeps your bladder calm, not the most restrictive diet you find online.

**30.How can someone with interstitial cystitis find out which foods bother their bladder?**

This is a very practical and important question. For someone with Interstitial Cystitis (IC), finding personal food triggers is less about a universal "bad food list" and more about becoming a detective of their own body. The standard approach is a structured **elimination and challenge diet**, often called the "IC Diet."

Here is a step-by-step, actionable guide to finding which foods bother your bladder.

**Step 1: Understand the "Why" (The Short List of Common Culprits)**

IC triggers typically fall into a few chemical categories. Knowing this helps you know what to look for:

**Acids:** Citrus (lemon, orange, grapefruit), tomatoes, pineapple, most berries (except blueberries).

**Potassium:** High-potassium foods like bananas, oranges, avocados, dried fruit.

**Irritants:** Caffeine (coffee, tea, chocolate), alcohol, artificial sweeteners (especially aspartame and saccharin).

**Preservatives & Additives:** MSG, nitrates/nitrites (in deli meat, hot dogs), benzoates, sulfites (in wine, dried fruit).

**Spices & Alliums:** Chili, curry, wasabi, raw onions, raw garlic.

**However, a key fact:** Triggers are highly individual. Some people react to coffee but not wine. Others react to watermelon but not lemon. So a rigid list is not the final answer.

**Step 2: Start with a Strict "Bladder-Friendly" Baseline Diet (7-14 days)**

The goal is to give your bladder a complete rest and find a "zero-symptom" or "low-symptom" baseline. For 7-14 days, eat *only* foods from the very low-risk list. This is temporary.

**Safe foods for most people (the "IC Rescue Diet"):**

**Fruits:** Pears, blueberries.

**Vegetables:** Asparagus, broccoli, cabbage, carrots, celery, cucumbers, green beans, potatoes (peeled), squash, zucchini.

**Proteins:** Eggs, chicken (not processed), turkey, fish, tofu.

**Grains:** Oats, white rice, pasta, sourdough bread (no preservatives).

**Fats:** Olive oil, coconut oil, unsalted butter.

**Dairy (if tolerated):** Blended milk (no sharp cheeses), plain cream cheese, cottage cheese.

**Drinks:** Water (room temp or warm is best), pear juice, *very* weak chamomile or peppermint tea (test carefully).

**Crucial:** Keep a simple **Symptom & Food Log** right from this first day. Note: Time, food eaten, and then your bladder pain/urgency level 1-10 for the next 4-6 hours.

**Step 3: The Challenge Phase – Test One Food at a Time**

After 7-14 days on the safe diet (and your symptoms are as low as they can be), you begin testing. This is the actual "finding out" part.

**The Rule of One:** Never test more than one new food or drink per 3 days.

**The Process:**

**Day 1 (Test Day):** Eat a *moderate* serving of the test food (e.g., 1/2 cup of chopped tomatoes, or one cup of coffee). Eat it alone or with other *known safe* foods. Log symptoms for 12-24 hours.

**Day 2 (Rest Day):** Return completely to the safe diet. Note if any symptoms continue.

**Day 3 (Rest Day 2):** Return completely to the safe diet. Your symptoms should be back to baseline.

**Then choose your next test food.**

**How to interpret results:**

**Clear (No reaction):** You can likely add this food back into your normal diet.

**Mild reaction (slight increase in urgency/frequency):** Put it on your "caution" list. You might tolerate small amounts or only when your bladder is calm.

**Severe reaction (clear pain or flare-up):** Put it on your "avoid" list. Don't test it again for several months, as tolerance can change.

**Step 4: Watch Out for "Hidden" and "Cumulative" Triggers**

**The "Stacking" Effect:** You might tolerate a little coffee. You might tolerate a little chocolate. But if you eat both on the same day + a stressful event? That can trigger a flare. Logging *combinations* is vital.

**Prep method matters:** A raw tomato might be a trigger, but tomato sauce cooked for 4 hours might be fine. Roasted garlic is often better than raw. Fresh chili is worse than dried.

**Pharmaceuticals & Supplements:** Vitamin C (especially ascorbic acid), cranberry pills, and even some probiotics can be triggers.

**Step 5: Get Professional Tools (Highly Recommended)**

Blindly guessing is frustrating. Use these resources to shortcut the process:

**The IC Food List App (from the ICA):** The Interstitial Cystitis Association has a free, searchable app called *IC Food List*. It color-codes foods as "Try," "Caution," and "Avoid" based on thousands of patient reports. It's far more nuanced than any static list.

**A Urologist or Pelvic Floor PT who specializes in IC:** They can help differentiate between food triggers and other triggers (stress, hormones, pelvic floor tension). They may also recommend a **low-oxalate diet** (if that's a factor for you) or **antihistamine challenges** (if MCAS is involved).

**The Single Most Important Thing to Know**

**Food is rarely the *only* cause of an IC flare.** Stress, lack of sleep, allergies, exercise, and sexual activity are often just as powerful. So, when you do a food challenge, try to keep those other variables as stable as possible.

If you test coffee on a day you also get bad news at work, you might blame the coffee when it was actually stress.

If you test pizza on a day you also do a 10-mile hike, you might blame the tomato sauce when it was the exercise.

**A final, encouraging truth:** Most people with IC do not need to avoid all trigger foods forever. After a period of bladder healing (months on medication, PT, or stress management), many find they can slowly reintroduce previously reactive foods in small amounts. This process is about *learning your body's language*, not imposing a life sentence of bland food. Start with the elimination diet. The answers will come.

**31.Should people with interstitial cystitis drink less water to urinate less often?**

This is a very common question, and the answer might be surprising: **Generally, no.** In fact, drinking less water is usually the wrong approach and can actually make interstitial cystitis (IC) symptoms worse.

Here’s why, and what you should do instead.

**Why Drinking Less Water Backfires**

The instinct to drink less makes sense: less fluid in, less urine out, fewer trips to the bathroom. However, for IC, the issue isn't the *volume* of urine, but the *concentration* of irritants within it.

**More Concentrated, More Irritating:** When you are dehydrated, your urine becomes highly concentrated with acids, salts, and waste products. This concentrated urine is far more irritating to a damaged, inflamed bladder lining (the hallmark of IC).

**Pain & Urgency:** That concentrated urine directly hitting the bladder wall causes the very symptoms you're trying to avoid: burning, stinging, urgency, and pelvic pain. Ironically, this can then trigger even *more* frequent, painful urination.

**It Can Worsen Frequency:** A highly irritated bladder will desperately try to expel that painful, concentrated urine, leading to the sensation of needing to go very often, even if only a tiny amount comes out.

**The Better Strategy: Smart Hydration**

The goal for managing IC is not to make *less* urine, but to make *less irritating* urine. Dilute urine is much gentler on the bladder.

**Drink enough water throughout the day** to keep your urine a pale, straw-like color. Dark yellow urine is a sign of concentration and a trigger for many IC patients.

**Sip steadily** rather than gulping large amounts at once. Spreading your intake can help avoid sudden bladder filling.

**The Two Most Important Exceptions (When to Adjust)**

The blanket "drink water" advice needs two key adjustments:

**Avoid Bladder Irritants:** Don't try to hydrate with the wrong things. Coffee, tea, soda (especially diet), citrus juices, cranberry juice, and alcohol are common IC triggers. **Stick with plain water** as your primary fluid.

**Adjust Before Sleep:** To reduce nighttime urination (nocturia), you can **taper off** fluids 2-3 hours before bed. For example, stop drinking at 8 PM if you go to bed at 10 or 11 PM. This allows your kidneys time to process the fluid before you lie down.

**What the Experts Say**

Leading IC organizations (like the Interstitial Cystitis Association) and specialists consistently advise **against** fluid restriction. They emphasize that dehydration is a major, avoidable trigger for IC flares.

**In Summary: What You Should Do**

**Do NOT** drastically cut back on water. This often worsens pain and frequency.

**DO** drink enough plain water to keep your urine dilute and pale yellow.

**DO** reduce fluids only in the 2-3 hours before bedtime to manage nighttime trips.

**DO** keep a symptom diary. A very small number of people with IC feel better when they temporarily reduce volume, but this is the exception, not the rule. Your diary will tell you what works for *your* body.

**Most importantly:** Discuss your hydration strategy with your urologist or IC specialist. They can give you personalized advice based on your specific bladder capacity, symptoms, and treatment plan. Never make major changes to your fluid intake without medical guidance, especially if you have other health conditions.

In short, think "dilute, not dry." Your bladder will likely thank you.

**32.Can emotional stress make interstitial cystitis symptoms worse?**

Yes, absolutely. Emotional stress is one of the most consistently reported triggers for worsening interstitial cystitis (IC) symptoms, also known as bladder pain syndrome (BPS).

Here’s a breakdown of how stress affects IC, why this happens, and what you can do about it.

**The Direct Link: Stress & IC Flares**

For many people with IC, a stressful week at work, relationship difficulties, financial worries, or even a stressful holiday can directly lead to a flare-up of symptoms, including:

**Increased bladder pain or pressure** (often described as a dull ache or sharp pain that worsens as the bladder fills)

**More frequent and urgent urination** (day and night)

**A feeling of bladder "spasms" or pelvic floor tension**

**Worsening of other associated symptoms** like pelvic pain or painful intercourse

**Why Does This Happen? The Biological Mechanisms**

The connection between your brain and your bladder is powerful and complex. Stress doesn't just *feel* bad; it triggers real physiological changes that directly impact your bladder.

**The Nervous System Connection:** Your bladder is densely packed with nerve endings. When you experience stress, your body's "fight or flight" (sympathetic nervous system) activates. This can lead to:

**Increased nerve sensitivity:** Nerves in the bladder become hypersensitive, sending pain signals to the brain from normal stimuli (like a small amount of urine).

**Pelvic floor muscle tension:** Stress often causes people to unconsciously clench their muscles. Chronically tight pelvic floor muscles are a major source of pain and can directly mimic or worsen IC symptoms.

**Immune and Inflammatory Response:** Chronic stress elevates hormones like cortisol and catecholamines. While helpful in short bursts, long-term stress disrupts the immune system and can **increase systemic inflammation**. Since IC is thought to involve a compromised bladder lining (epithelium) and mast cell activation (cells that release inflammatory chemicals), stress can tip the balance toward a full-blown flare.

**Central Sensitization:** This is a key concept for IC. Over time, repeated stress and pain signals can "rewire" the central nervous system (brain and spinal cord) to be in a constant state of high alert. This lowers your pain threshold, meaning a normal bladder sensation is interpreted as severe pain. Stress powerfully drives this process.

**It's a Vicious Cycle**

The relationship is bidirectional and often becomes a self-reinforcing loop:

**Stress IC Flare Increased pain and disruption of sleep/work/life More anxiety and stress about the flare Even worse IC symptoms (and the cycle continues)**

Breaking this cycle is a major goal of comprehensive IC treatment.

**What Can You Do? Managing Stress to Manage IC**

Recognizing the stress-IC link is the first step. The good news is that stress management isn't just "relaxation" – it's a legitimate, evidence-based treatment for IC.

**Stress-Specific Therapies (Highly Recommended):**

**Cognitive Behavioral Therapy (CBT):** This is the gold standard. CBT helps you identify stress and anxiety triggers, change negative thought patterns that worsen pain, and develop specific coping skills. Look for a therapist familiar with chronic pain or IC.

**Mindfulness-Based Stress Reduction (MBSR):** Teaches you to observe pain and stressful thoughts without judgment, which can reduce the emotional reactivity that drives flares.

**Body-Based Techniques (Excellent for Pelvic Floor Tension):**

**Gentle pelvic floor physical therapy:** A specialist can teach you reverse Kegels and relaxation techniques to release chronically tight muscles.

**Diaphragmatic (belly) breathing:** Learning to breathe deeply into your belly, rather than shallow chest breaths, signals your nervous system to *down-regulate* from fight-or-flight mode and relaxes the pelvic floor.

**Progressive muscle relaxation:** Systematically tensing and releasing muscle groups to recognize and release hidden tension.

**Lifestyle & Daily Practices:**

**Prioritize sleep:** Poor sleep dramatically lowers your stress resilience. Work on sleep hygiene.

**Low-impact exercise:** Walking, swimming, or gentle yoga (avoid positions that compress the bladder) can burn off stress hormones.

**Set boundaries and say "no":** Over-committing is a major stress source. Protect your energy.

**Keep a symptom-stress diary:** For a few weeks, note your stress level (1-10) and your IC symptoms (1-10). Look for the pattern. This confirms the link for *you* and can be powerful data for your doctor.

**Important Medical Note**

While stress is a major trigger, **it is not the cause of IC**. IC is a real organic bladder condition. If a doctor ever tells you, "It's just stress" or "It's all in your head," find a new doctor. The proper approach is: "Stress makes your very real bladder condition worse, so we need to treat the bladder *and* help you manage stress."

**When to talk to your doctor:** If you feel your stress or anxiety is overwhelming, or if the cycle is relentless, discuss this with your urologist or primary care doctor. They can recommend therapists, medications for anxiety (some of which, like low-dose amitriptyline, treat both IC pain and stress), or other resources.

In short, yes – emotional stress is a very real and powerful trigger for IC. But by understanding the mechanism and actively employing stress management techniques, you can regain a significant amount of control over your symptoms.

**33.What daily habits may help reduce interstitial cystitis flare-ups?**

Establishing a consistent daily routine is a cornerstone of managing Interstitial Cystitis (IC). The goal is to minimize bladder irritation and manage the triggers that can lead to a flare.

**Manage Your Diet**

Diet plays a critical role in managing IC. It's crucial to understand that trigger foods are unique to each person. Maintaining a **food diary** is one of the most effective ways to identify which foods, drinks, or activities are causing your specific flare-ups.

**Common triggers to consider limiting**: Many people find relief by reducing or eliminating the following:

**High-acid foods and drinks**: Citrus fruits and juices (orange, grapefruit), tomatoes and tomato-based sauces, pineapple, and strawberries.

**Caffeinated and carbonated beverages**: Coffee, tea, soda, and other fizzy drinks.

**Alcohol**.

**Spicy foods** (e.g., chili peppers).

**Other potential triggers**: Chocolate, foods containing MSG, artificial sweeteners, processed meats, soy products, and certain condiments like vinegar or soy sauce.

**Following an elimination diet**: An **elimination diet** is a structured way to pinpoint your triggers. For 1-2 weeks, you stop eating all potential trigger foods. If your symptoms improve, you then slowly reintroduce foods one by one to see which ones cause a reaction.

**Explore bladder-friendly options**: Many people with IC find they can comfortably eat foods like **bananas, blueberries, pears, broccoli, whole grains (like oats and rice), and lean proteins such as chicken**.

**Stay Properly Hydrated**

Proper hydration is essential for managing IC, even if it seems counterintuitive.

**Why it helps**: When you are dehydrated, your urine becomes highly concentrated and more acidic, which can directly irritate the bladder wall. Drinking enough water dilutes your urine, making it less irritating.

**Aim for a healthy balance**: A common guideline is about 6-8 glasses per day, but your needs may vary. A good indicator of proper hydration is having **pale yellow or clear urine**.

**Make it a habit**: Keep a water bottle with you as a visual reminder to sip throughout the day. Adding a few slices of cucumber or a handful of blueberries can add flavor without triggering a flare.

**️ Practice Bladder Training**

If you experience urinary urgency or frequency (needing to go often), **bladder training** is a technique that can help your bladder hold more urine, thereby reducing pain and the sense of urgency.

**How it works**: The technique involves gradually increasing the time between bathroom visits with the guidance of a healthcare professional.

**A helpful tip**: Try to resist the urge to go "just in case" you might need to later, as this can make your bladder more sensitive over time.

**Prioritize Physical Activity and Stress Management**

Stress is a very common trigger for IC flare-ups. Since emotional tension can manifest as physical symptoms, managing your stress levels is crucial.

**Stress-reducing activities**: Techniques such as deep breathing exercises, meditation, yoga, and massage can be very effective for calming the nervous system and reducing bladder pain.

**Start with gentle exercise**: Low-impact activities like **walking** and **swimming** are ideal, as they are less likely to trigger symptoms compared to high-impact sports.

**Explore pelvic floor therapy**: For many, IC is linked to tension in the pelvic floor muscles. A specialized physical therapist can teach you exercises to relax these muscles, which can provide significant relief. It's important to note that strengthening exercises like Kegels may not be recommended for IC unless you are under the supervision of a therapist.

**Quit Smoking**

If you smoke cigarettes, quitting can lead to a significant improvement in your IC symptoms. Smoking is known to cause direct irritation to the bladder lining.

Finally, remember that your doctor is your best partner in managing IC. As you make these lifestyle changes, keep them informed of your progress. They can offer you additional treatments and adjust your management plan to best suit your individual needs.

**34.Can interstitial cystitis be cured completely?**

Currently, there is no known cure for interstitial cystitis (IC).

However, this doesn’t mean people with IC are without hope or effective options. The condition is highly manageable, and treatment focuses on controlling symptoms to significantly improve quality of life.

**No Single Cure, But Many Manageable Paths**: Since IC presents differently in everyone, there isn’t one treatment that works for all. But many people find that one treatment or a combination of approaches can provide significant, long-term relief.

**The Goal is Remission, Not Eradication**: While completely eliminating every symptom is rare, the primary goal is to achieve **remission**—a prolonged period with few or no symptoms.

One long-term study found that over time, **12% of patients became completely free of symptoms**, and nearly half (47%) experienced significant improvement.

For those with a specific type of IC known as Hunner's lesions, advanced combination therapies have shown a **90% subjective improvement rate**, with some patients even feeling "cured".

The path to remission usually involves a step-by-step approach, starting with the least invasive options.

| Step | Approach | Examples |
| --- | --- | --- |
| **First Line** | **Lifestyle & Behavior** | Diet modification (e.g., reducing acidic foods, caffeine, and alcohol); Stress management and bladder training. |
| **Second Line** | **Oral & Topical Medications** | Prescription drugs like pentosan polysulfate sodium (Elmiron) to help restore the bladder lining, or tricyclic antidepressants like amitriptyline to manage pain and urinary frequency. |
| **Further Options** | **Bladder Therapies & Procedures** | **Bladder instillation** (placing a liquid medication directly into the bladder via a catheter); **Transurethral fulguration** (a surgical procedure to burn off Hunner's lesions). |

**A Final Takeaway**

While a definitive cure remains elusive, it is **rare for symptoms to go completely untreated**. Up to 90% of people with IC can find relief with the right management plan. Finding that plan often takes patience and a process of trial and error with a urologist.

The key is to shift your focus from finding a single cure to building a personalized toolkit that works for you.

If you're interested, I can provide more detail on specific strategies, such as dietary changes for IC or the different types of bladder instillation therapies. Would that be helpful?

**35.What is the main goal of interstitial cystitis treatment?**

The main goal of interstitial cystitis (IC) treatment is **to relieve symptoms (pain, pressure, and urinary urgency/frequency) and improve quality of life**, rather than curing the disease itself.

Since IC has no known cure and its course varies greatly, treatment focuses on individualized symptom management. Key objectives include:

**Reducing bladder pain and discomfort**

**Decreasing urinary frequency and urgency** (day and night)

**Improving bladder capacity and function**

**Minimizing treatment side effects**

**Helping patients return to normal daily activities and sleep**

Treatment typically follows a stepwise approach—starting with conservative measures (diet modification, stress management) and progressing to oral medications, bladder instillations, or more advanced therapies (e.g., neuromodulation) only as needed. The ultimate success is measured by the patient's symptom relief and functional improvement, not by any single test result.

**36.How long can interstitial cystitis treatment take to start working?**

This is an excellent question because expectations for interstitial cystitis/bladder pain syndrome (IC/BPS) treatment are often misunderstood. Unlike a simple infection where antibiotics work in days, IC treatments work on a very different, slower timeline.

Here’s the realistic breakdown:

**The short answer:** Most IC treatments take **6 to 8 weeks** before you notice a clear benefit, and it can take **3 to 6 months** to feel the full effect.

However, this varies dramatically depending on the specific treatment. Let's break it down by type.

**Timeline by Treatment Type**

| Treatment Type | When it *starts* to work | When it *peaks* / full effect | Key Notes |
| --- | --- | --- | --- |
| **Dietary Changes** | **2-4 weeks** | 2-3 months | Removing acidic/irritating foods. Some notice relief in days; for others, bladder healing takes weeks. |
| **Physical Therapy (Pelvic Floor)** | **4-6 weeks** | 3-6 months | Requires weekly sessions + home exercises. Slow retraining of muscles/nerves. |
| **Oral Medications** (Amitriptyline, Hydroxyzine, Cimetidine) | **6-8 weeks** | 3-6 months | These work by modulating nerve signals or histamines, not immediate symptom relief. Must take daily. |
| **Bladder Instillations** (Rescue instills) | **Variable:** Immediate (numbing effect) 4-6 weeks (healing effect) | After 6-8 weekly treatments | The lidocaine gives *temporary* relief for hours/days. The disease-modifying effect of DMSO or GAG replacers takes weeks of repeated doses. |
| **Elmiron (Pentosan Polysulfate)** | **3-6 months** | 6-12 months | The slowest-acting. Doctors require a 6-month trial minimum. Rarely works before 3 months. |
| **Nerve Stimulation** (PTNS, TENS) | **6-8 weeks** | 3-4 months | Requires weekly 30-min sessions for ~12 weeks. |
| **Botox (into bladder)** | **7-14 days** | 4-8 weeks | One of the faster options for severe urgency/frequency, but effect wears off in 6-9 months. |

**The Most Important Concept: "Start Working" vs. "Adequate Trial"**

Doctors distinguish between:

**Initial signal** (a subtle hint that you're on the right track, like slightly longer between bathroom trips or slightly less intense pain).

**Meaningful improvement** (a clear, daily quality-of-life change).

**Never stop an IC treatment after 1-2 weeks because you feel no difference.** That would be like stopping antibiotics for a sinus infection after one pill. For oral meds and Elmiron, a **6-month commitment** is standard before declaring it a failure.

**Why does IC treatment take so long?**

**Nerve remodeling:** Many IC treatments (like amitriptyline) change how your brain perceives bladder signals. New nerve pathways take weeks to form.

**Mast cell stabilization:** These are immune cells in the bladder wall that release inflammatory chemicals. Stabilizing them is a slow biological process.

**Glycosaminoglycan (GAG) layer repair:** Rebuilding the bladder's protective lining is like patching drywall—it requires many layers over time.

**Muscle reprogramming:** A chronically tight pelvic floor doesn't relax overnight. It took months or years to get dysfunctional; it needs months to relearn.

**What about immediate relief?**

If you are in an active flare, waiting 8 weeks isn't feasible. For immediate, short-term symptom control during the "waiting period" for main treatments to work, ask your doctor about:

**Rescue instillations** (lidocaine/heparin – works in minutes, lasts hours to a few days)

**Alkalinizing agents** (like Uro-MP, Prelief with meals, or oral baking soda in water)

**Antihistamines** (like hydroxyzine may work partially within days for some)

**Urinary analgesics** (phenazopyridine / AZO – for no more than 2 days)

**Red flags: When to reassess**

If you've given a treatment a *full, appropriate trial* (e.g., 8 weeks for amitriptyline, 6 months for Elmiron) with **zero** change in frequency, pain, or urgency, it's time to discuss stopping and trying something else with your urologist.

**However, if you experience new or worsening severe pain, high fever, or inability to urinate, seek immediate medical care—do not wait for the treatment to "kick in."**

**Bottom line**

**Be patient:** Expect 6-8 weeks for most treatments to *begin* showing subtle benefits.

**Be consistent:** Take meds daily, attend all PT sessions, follow the diet.

**Manage flares separately:** Have a rescue plan (instillations, urinary analgesics, heat/ice, stress reduction) for the waiting period.

**Communicate:** If you're at 10-12 weeks with no hint of improvement, your doctor may adjust dosing or try a combination approach.

Would you like me to go deeper into any specific treatment (e.g., how to know if amitriptyline is working at a low dose, or what to expect during your first 6 weeks of the IC diet)?

**37.Why do doctors often try interstitial cystitis treatments step by step?**

That's an excellent question. Doctors use a step-by-step, or "stepped," approach to treating interstitial cystitis (IC) for several crucial reasons that prioritize patient safety, comfort, and long-term success.

Think of it like a ladder. You start on the lowest, safest rung. Only if that doesn't work do you climb to the next one. Here are the main reasons why:

**1. No Single Cure, No Single Test**

Unlike a bacterial infection where a simple test identifies the germ and an antibiotic cures it, IC has no known single cause and no definitive diagnostic test. It's a diagnosis of exclusion. Because the underlying cause can vary from person to person (e.g., a defective bladder lining, pelvic floor dysfunction, an autoimmune issue), there's no "one-size-fits-all" treatment. Stepped therapy allows doctors to find what works for *that specific patient*.

**2. Minimizing Risk and Side Effects (The Golden Rule)**

The most important principle is "first, do no harm." Treatments for IC range from very low-risk to high-risk.

**Step 1 (Low Risk):** Dietary changes, stress management, physical therapy. These have virtually no side effects and can be very effective.

**Step 2 (Low-Medium Risk):** Oral medications like amitriptyline (a low-dose antidepressant that calms nerve pain) or hydroxyzine (an antihistamine). These have common but manageable side effects like drowsiness or weight gain.

**Step 3 (Medium Risk):** Bladder instillations (putting liquid medication directly into the bladder via a catheter). This is more invasive and carries a small risk of urinary tract infection (UTI).

**Step 4 (Higher Risk):** Procedures like hydrodistention (stretching the bladder under anesthesia) or neuromodulation (implanting a device that alters nerve signals). These have risks of anesthesia, bleeding, infection, or device malfunction.

**Last Resort (High Risk):** Major surgeries like bladder augmentation or removal. These have significant risks, permanent consequences, and can create new, different problems.

By starting with the least invasive, lowest-risk options, doctors protect the patient from unnecessary harm.

**3. Managing Cost and Convenience**

IC is a chronic condition, often requiring lifelong management.

**Step 1 changes** (avoiding coffee and tomatoes) are free.

**Step 2 pills** might cost a monthly copay.

**Step 3 instillations** require frequent clinic visits and catheterization.

**Step 4 devices or surgeries** are extremely expensive and require specialists and recovery time.

Stepping up only when necessary respects the patient's time, finances, and lifestyle.

**4. The Condition is Highly Variable**

IC symptoms are not the same for everyone. One person might have a "leaky" bladder lining, another might have pelvic muscles so tight they feel like a vice. The right treatment for the first person (a drug to coat the bladder, like Elmiron) could be useless for the second person, who needs pelvic floor physical therapy. A step-by-step approach lets the doctor and patient learn together which "flavor" of IC they are dealing with.

**5. The Power of the Placebo Effect (Used Ethically)**

A significant number of people with IC (often around 30-40%) will improve on a placebo in clinical trials. By starting with a simple, safe intervention like dietary changes, a doctor can capture this genuine improvement. If they jumped straight to an invasive procedure, they'd be exposing the patient to unnecessary risk when a simple conversation might have worked.

**A Typical Step-by-Step Plan (The "IC Ladder")**

**Step 1: Self-Care & First-Line (Try for 2-4 months)**

**Strict diet** (eliminating acidic, spicy, caffeinated, and potassium-rich foods).

**Stress reduction** (yoga, meditation, therapy).

**Bladder retraining** (urinating on a schedule).

**Gentle physical therapy** for pelvic floor relaxation.

**Step 2: Oral Medications (Add or try for 3-6 months)**

Amitriptyline (for nerve pain/sleep).

Hydroxyzine (for allergy/mast cell component).

Cimetidine (for possible anti-inflammatory effect).

*Elmiron (pentosan polysulfate sodium) is often here, but use has dropped due to risk of a rare eye injury.*

**Step 3: Minimally Invasive Procedures (If steps 1-2 fail)**

**Bladder instillations** (e.g., lidocaine, heparin, bicarbonate) done in a clinic.

**Hydrodistention** under anesthesia (provides temporary relief for many).

**Step 4: Advanced / Experimental (If severe symptoms persist)**

**Neuromodulation** (InterStim or PTNS - Percutaneous Tibial Nerve Stimulation).

**Cyclosporine A** (a powerful immune-suppressing drug with serious side effects).

**Botox injections** into the bladder wall (can cause urinary retention).

Pain clinic referral for multidisciplinary management.

**Step 5: Last Resort**

Major surgery (urinary diversion or bladder removal).

**The Most Important Reason: It Works Best**

Multiple clinical guidelines (from the American Urological Association, the European Association of Urology, etc.) strongly recommend this stepped approach because evidence shows it provides the greatest symptom relief while causing the least amount of harm over a patient's lifetime. You can always escalate care, but you can rarely reverse the side effects of a failed aggressive treatment.

**In short: Doctors go step-by-step because IC is a puzzle box, not a lock. Trying the simplest key first is the wisest, safest, and most effective path to solving it.**

**38.Is it safe to take over-the-counter pain medicine for interstitial cystitis?**

Taking over-the-counter (OTC) pain medicine for interstitial cystitis (IC) can be safe for managing mild to moderate discomfort, but it is crucial to do so with full awareness of the risks and limitations. OTC options fall into a few main categories, each with a specific role and set of precautions.

**Potential OTC Pain Relief Options for IC**

**Nonsteroidal Anti-Inflammatory Drugs (NSAIDs)**: They are often used for short-term relief of mild to moderate IC pain, as they work by reducing inflammation in the body. Common NSAIDs include ibuprofen (e.g., Advil, Motrin) and naproxen sodium (Aleve).

**Important Safety Notes**: For some, NSAIDs can cause stomach irritation, bleeding, or kidney problems. Long-term use is not recommended, and they should be used with caution if you have a history of stomach ulcers or high blood pressure, or if you take blood thinners.

**Acetaminophen (Tylenol)**: This is another first-line OTC option, particularly useful for those who cannot take NSAIDs due to stomach issues.

**Important Safety Notes**: The main concern is liver damage, especially when taking high doses or combining it with alcohol. Always follow the recommended dosage and be aware that it's an ingredient in many other OTC products.

**Urinary Tract Analgesics (Phenazopyridine)**: Medications containing phenazopyridine (sold as AZO, Uristat, or Cystex) work as a local anesthetic for the urinary tract.

**Important Safety Notes**: This medication is for **short-term use only**. It provides temporary relief by numbing the bladder wall but does not treat the underlying cause of IC. A harmless but notable side effect is that it turns urine a bright orange color.

**️ Key Precautions Before Using OTC Pain Medicine**

While OTC options can provide relief, they are not without risk:

**Short-Term Relief is the Goal**: OTC medications can help manage mild pain, but they are not a solution for chronic pain.

**Risk of Side Effects**: Each type of medication carries risks, especially with prolonged use. For example, NSAIDs are notorious for stomach irritation, and acetaminophen can harm the liver in high doses.

**Consult a Professional**: **Always speak with your doctor or pharmacist before starting any new OTC medication**. They can help you understand potential interactions with other drugs you're taking and advise on the best choice for your overall health and specific IC symptoms.

**A Holistic Approach to Managing IC Pain**

OTC pain relievers are just one tool. Your doctor may recommend combining them with other approaches, such as:

**Prescription Medications**: For more severe or persistent pain, your doctor may prescribe other medications.

**Dietary Changes**: Avoiding acidic, spicy, or caffeinated foods and drinks may help prevent flare-ups.

**Pelvic Floor Physical Therapy**: This can be very effective for relieving pelvic pain and muscle tension.

**OTC Supplements**: Some find relief with supplements like aloe or PEA, though it's vital to research them carefully.

For personalized medical advice, it's best to consult a healthcare professional. I hope this information helps you have a more informed conversation with your doctor.

**39.Are antibiotics helpful for interstitial cystitis when there is no infection?**

This is an excellent question because it gets to the heart of a common confusion in urology.

The short answer is: **No, standard antibiotics are not helpful for treating interstitial cystitis (IC) when there is no active bacterial infection.**

Here’s the detailed explanation why, and where the confusion comes from.

**The Core Reason: IC is Not an Infection**

Interstitial cystitis (also called Bladder Pain Syndrome) is a chronic condition characterized by pain, pressure, or discomfort in the bladder, usually accompanied by urinary frequency and urgency. The cause is not fully understood, but leading theories point to:

**A defective bladder lining (leaky epithelium):** This allows irritating substances in urine to penetrate the bladder wall.

**Chronic inflammation:** The body's own immune response causes nerve irritation and pain.

**Autoimmune reaction:** The immune system mistakenly attacks the bladder.

**Pelvic floor muscle dysfunction.**

Since bacteria like *E. coli* are not the cause, antibiotics that kill bacteria will have no effect on these underlying mechanisms.

**Where Does the Confusion Come From?**

You might see studies or hear anecdotes about people with IC feeling better on antibiotics. There are a few key reasons for this:

**Misdiagnosis:** The biggest issue. Symptoms of a chronic, low-grade urinary tract infection (UTI) can be identical to IC. Standard urine cultures often miss fastidious (hard-to-grow) organisms or embedded infections. So, a patient diagnosed with "IC" who actually has a chronic, undetected UTI *will* improve with the correct, long-term antibiotic. However, this means they never had true IC—they had a chronic infection.

**Anti-inflammatory, not Anti-bacterial:** Some antibiotics (a class called macrolides, like clarithromycin or azithromycin) have proven **anti-inflammatory** and **immunomodulatory** effects, completely separate from their ability to kill bacteria. If an IC patient improves on one of these, it's not because the drug killed a germ, but because it calmed the immune overreaction. **Important note:** These are *not* the standard antibiotics (like nitrofurantoin, Bactrim, or ciprofloxacin) usually prescribed for UTIs. Doctors rarely use macrolides for IC due to side effects and a lack of strong evidence.

**Placebo Effect and Natural Fluctuation:** IC symptoms often wax and wane. A patient might start a course of antibiotics (for a suspected, but later ruled-out, UTI) just as their symptoms are naturally improving. They then credit the antibiotic.

**Why You Should NOT Take Antibiotics for IC (Without an Infection)**

Taking unnecessary antibiotics is not harmless. The risks include:

**Antibiotic resistance:** Creating "superbugs" that are hard to treat later when you truly need an antibiotic.

**C. diff infection:** Antibiotics can wipe out good gut bacteria, allowing the dangerous *Clostridioides difficile* to take over, causing severe diarrhea.

**Side effects:** Ranging from mild (rash, nausea, yeast infection) to severe (tendon rupture from ciprofloxacin, allergic reactions).

**The Right Way to Treat IC**

If you have confirmed IC with no infection, treatment focuses on restoring the bladder lining and calming the nervous system. Evidence-based options include:

**Oral medications:** Pentosan polysulfate (Elmiron – helps rebuild the bladder lining), amitriptyline (blocks nerve pain), hydroxyzine (antihistamine).

**Bladder instillations:** Putting a solution like DMSO, lidocaine, or heparin directly into the bladder via a catheter.

**Dietary changes:** Avoiding acidic foods (citrus, tomatoes), high-potassium foods (bananas), spicy foods, and artificial sweeteners.

**Physical therapy:** For associated pelvic floor muscle tension.

**Neuromodulation:** Techniques like transcutaneous electrical nerve stimulation (TENS) or sacral nerve stimulation.

**The Bottom Line**

**If you have NO infection (confirmed by a reliable, PCR-based urine test, not just a standard culture), standard antibiotics will NOT help your true Interstitial Cystitis.**

**If you were diagnosed with IC but feel better on antibiotics, discuss with your doctor the strong possibility that you actually have a chronic, undetected UTI.**

**Never take antibiotics for IC symptoms without a positive urine test confirming an infection.**

Always consult with a urologist who specializes in IC/BPS or chronic UTIs to get the correct diagnosis and targeted treatment.

**40.Should someone keep taking antibiotics for interstitial cystitis if urine tests show no infection?**

This is an important clinical question. The short answer is **no, you should not continue taking antibiotics** for interstitial cystitis (IC) if repeated, standard urine tests show no infection.

Here is the detailed explanation why, and what you should do instead.

**Why Continuing Antibiotics is Likely Harmful**

**IC is not an infection.** Interstitial cystitis (also called Bladder Pain Syndrome) is a chronic condition characterized by bladder wall inflammation, defects in the protective lining (glycosaminoglycan layer), or nerve hypersensitivity. It is **not** caused by bacteria.

**Antibiotics don't treat IC.** First-line IC treatments include dietary changes, physical therapy, bladder instills, amitriptyline, hydroxyzine, or pentosan polysulfate. Antibiotics have no role in treating the underlying pathology of IC.

**Risks outweigh benefits.** Taking unnecessary antibiotics exposes you to:

**Antibiotic resistance** (making future real infections harder to treat).

**Gut microbiome disruption** (diarrhea, *C. diff* infection).

**Yeast infections** (vaginal or oral thrush).

**Side effects** (nausea, rash, tendonitis with fluoroquinolones).

**A Critical Exception to Consider**

There is one scenario where a doctor might continue antibiotics *despite* a standard urine culture being negative: **Chronic or "stealth" infections**.

Standard urine cultures are designed to detect fast-growing, common UTI bacteria (like *E. coli*). They **cannot** detect slow-growing bacteria, intracellular bacteria (hiding inside bladder cells), or organisms like *Ureaplasma*, *Mycoplasma*, or some *Corynebacterium* species.

**Evidence check:** Some research suggests a small subset of patients diagnosed with IC may actually have an undiagnosed chronic infection. However, this is controversial. The AUA (American Urological Association) guidelines do not recommend long-term antibiotics for IC unless there is **confirmed** evidence of infection (e.g., by PCR, next-generation sequencing, or special cultures).

**What You Should Do Now**

**Do not stop antibiotics abruptly without speaking to your doctor** (to avoid incomplete treatment if there *is* a real infection). But you should absolutely schedule a conversation.

**Ask your doctor these specific questions:**

*"Why are we continuing antibiotics when my cultures are negative?"*

*"Have we ruled out fastidious organisms like Ureaplasma or Mycoplasma with PCR testing?"*

*"Can we switch to standard IC treatments instead?"*

**If your doctor insists on long-term antibiotics** without any positive culture or PCR test, seek a **second opinion** from a urogynecologist or a urologist who specializes in IC/BPS. Many well-meaning primary care doctors are not up-to-date on this.

**Request advanced testing** if infection is still suspected (e.g., MicrogenDX, Pathnostics, or a urologist-performed FISH test).

**The Bottom Line**

**For typical, standard-care interstitial cystitis: stop the antibiotics.** They will not help and may harm you. Work with a specialist to start evidence-based IC treatments.

**Only possible exception:** You have proof of a difficult-to-culture organism via advanced molecular testing. Without that, antibiotics are inappropriate.

**41.Why might eye checks be needed for some interstitial cystitis medicines?**

Eye checks are specifically needed for **pentosan polysulfate sodium (brand name Elmiron)**.

While other medications for interstitial cystitis (IC) can cause temporary side effects like blurred vision, they do not carry the same risk of long-term, irreversible eye damage as Elmiron. Here’s why this specific medication requires regular eye monitoring.

**The Primary Concern: Retinal Toxicity**

The main reason eye checks are crucial is that Elmiron has been linked to a severe, long-term side effect called **pentosan polysulfate maculopathy (PPSM)**. This condition, first reported in 2018, involves progressive damage to the retina—the light-sensitive tissue at the back of the eye.

The damage is **cumulative and generally irreversible**. Even after stopping the medication, retinal and vision changes may continue to progress.

The risk is closely tied to the total amount of medication taken over time, increasing significantly with higher cumulative doses and longer use. Studies suggest that retinal changes can begin to appear after about three to five years of standard use (when the total dose reaches approximately 500 grams).

**️ Symptoms to Watch For**

Vision changes from PPSM can be subtle at first. Key symptoms include:

Difficulty reading.

Vision that takes longer to adjust to low or reduced light environments.

Blurred vision.

**️ What Eye Checks Are Recommended?**

To catch any damage early, specific retinal examinations are essential:

**Baseline Exam**: Within six months of starting the medication.

**Advanced Imaging**: An ophthalmologist will use two specialized imaging tests that are vital for early detection: **OCT (optical coherence tomography)** and **autofluorescence imaging**.

**Follow-up Schedule**: After the baseline exam, follow-up eye exams are suggested **periodically** throughout the duration of treatment. The specific frequency will be determined by your doctor and eye care specialist. In some guidelines, this is specified as an exam by an optometrist every two years for patients under 60, and annually for those over 60. Importantly, follow-up exams should continue even after treatment ends, as changes can still progress.

**A Note on Other IC Medications**

It's important to distinguish Elmiron from other IC treatments, which have different ocular side effect profiles.

**Other IC Medications**

**Pentosan Polysulfate Sodium (Elmiron)**: May be required due to the risk of irreversible retinal damage.

**Tricyclic Antidepressants (e.g., Amitriptyline)** & **Antihistamines (e.g., Hydroxyzine)**: Not routinely required. These medications do not cause retinal toxicity. Their common side effects (e.g., dry eyes, blurred vision) are usually temporary and resolve with continued use or dose adjustment.

**Gabapentinoids (e.g., Gabapentin)**: Not routinely required. This medication can cause temporary vision issues like blurred or double vision, but routine eye monitoring is not standard practice.

**Key Takeaway**

The need for regular eye checks is a specific safety requirement for patients using **pentosan polysulfate sodium (Elmiron)**. This monitoring is crucial for the early detection of a rare but serious and potentially irreversible eye condition.

If you or someone you know is taking this medication, it is essential to follow the prescribed screening guidelines. If you have further questions or notice any changes in your vision, it is best to consult your healthcare provider.

**42.Should people with interstitial cystitis talk to a doctor before using supplements?**

Yes, absolutely. People with interstitial cystitis (IC) should always talk to a doctor or a pharmacist before starting any supplement. Here’s why this is so important:

**Risk of Bladder Irritation:** Many common supplements contain ingredients that can directly irritate the bladder lining and trigger a painful IC flare. For example:

**Vitamin C (ascorbic acid):** Can increase urine acidity, leading to burning and urgency.

**Cranberry:** Often used for UTIs, but highly acidic and a well-known IC trigger.

**Certain herbs (e.g., Echinacea, Ginseng):** Have been reported to worsen IC symptoms in some people.

**Potential Interactions with Medications:** Your doctor needs to know about all supplements to avoid dangerous interactions with IC treatments. For instance:

**Calcium or magnesium supplements** can interfere with the absorption of common IC medications like Elmiron (pentosan polysulfate) or certain antibiotics used for IC.

**St. John's Wort** can affect pain medications or antidepressants prescribed for IC pain.

**Lack of Regulation and Purity:** Supplements are not strictly regulated by the FDA. Products may contain undisclosed fillers, preservatives, or even active drug ingredients that could be harmful for someone with IC. A doctor can help you find reputable brands or formulations designed for IC (often unflavored, without preservatives or citric acid).

**Masking or Worsening Underlying Conditions:** Some IC symptoms overlap with other conditions (like recurrent UTIs, kidney stones, or endometriosis). A supplement that seems to "help" temporarily might mask a more serious issue that needs different treatment.

**What a doctor can do for you:**

**Review your specific IC triggers** and help you identify safe supplements (e.g., freeze-dried aloe vera, quercetin, or L-arginine have some evidence for IC, but only under guidance).

**Recommend a trial protocol** (starting with a very low dose of a single supplement to monitor for flares).

**Adjust your medications** if a supplement is necessary but could interact.

**In short:** Never assume a "natural" or "gentle" supplement is safe for IC. Your bladder's sensitivity is unique, and what helps one person could cause weeks of pain for you. Always consult your urologist or IC specialist first.

**43.Can pelvic floor physical therapy help interstitial cystitis?**

Yes, pelvic floor physical therapy (PFPT) can be very helpful for many people with interstitial cystitis (IC), also known as bladder pain syndrome (BMS).

Here’s why and how it helps:

**The connection between IC and pelvic floor muscles:** In many IC patients, the pelvic floor muscles become chronically tight, tense, and tender (a condition called hypertonic pelvic floor dysfunction). This can happen because the body reacts to chronic bladder pain by unconsciously guarding or clenching the pelvic floor. In turn, these tight muscles can compress and irritate the bladder, urethra, and surrounding nerves, directly mimicking or worsening IC symptoms like urgency, frequency, and suprapubic pain.

**What PFPT targets:** Unlike general exercise therapy, PFPT for IC focuses on **relaxing and lengthening** overactive pelvic floor muscles, not strengthening them. Techniques include:

**Myofascial release and trigger point therapy:** Manual (internal and/or external) techniques to release painful muscle knots and tight bands.

**Biofeedback:** Helps you learn to consciously relax your pelvic floor.

**Stretching and breathing exercises:** Diaphragmatic breathing and gentle stretches (like happy baby or deep squat) to reduce overall pelvic tension.

**Visceral mobilization:** Gentle techniques to improve bladder mobility and reduce restrictions.

**What research shows:** Multiple clinical studies and systematic reviews have found that PFPT significantly reduces pain, urgency, and frequency in IC patients, often with results comparable to other standard treatments. Many IC treatment guidelines (e.g., from the AUA) recommend pelvic floor PT as a second-line treatment, especially when pelvic floor tenderness is present on exam.

**Who benefits most:** It is most effective for IC patients who have signs of pelvic floor hypertonicity (e.g., pain with intercourse, difficulty inserting a tampon, a feeling of pelvic “tightness,” or tenderness on internal exam). However, even without obvious pelvic floor symptoms, many IC patients have some component of pelvic floor involvement.

**Important notes:**

PFPT is **not a cure** for the underlying bladder condition, but it is an excellent **symptom management tool**.

Results take time (often 6–12 sessions).

It is crucial to see a **specialized pelvic floor physical therapist** – general PTs may not have this training.

It is often used alongside other IC treatments (diet modification, medications, bladder instillations), not instead of them.

If you have IC, asking a urogynecologist or pelvic pain specialist for a referral to a pelvic floor PT is a very reasonable next step.

44.Are pelvic floor strengthening exercises always good for interstitial cystitis?

This is an excellent question because it gets at a common misconception. The short answer is: **No, pelvic floor strengthening exercises (like Kegels) are not always good for Interstitial Cystitis (IC). In fact, they can make symptoms significantly worse for many patients.**

Here’s the detailed breakdown of why, and when they *might* be helpful.

**The Crucial Distinction: Tight vs. Weak Muscles**

For a long time, "pelvic floor exercises" were automatically assumed to be Kegels (strengthening/contracting the muscles). However, we now understand that pelvic floor dysfunction falls into two main categories, often with opposite treatment needs.

**Hypertonic Pelvic Floor (Too Tight):** This is extremely common in IC. The muscles are in a constant, involuntary state of contraction (a "knot" or spasm), often as a protective response to chronic bladder pain. **For these patients, strengthening exercises are like doing bicep curls for a shoulder that's already in a painful, frozen spasm. It will increase tension, reduce blood flow, and worsen pain, urgency, and frequency.**

**Hypotonic Pelvic Floor (Too Weak/Loose):** This is less common in classic IC but can occur, especially after childbirth, surgery, or in older adults. This can lead to stress incontinence (leaking with cough/sneeze). **For these patients, targeted *strengthening* (Kegels) may be beneficial.**

**Why Kegels Are Often Harmful for IC Patients**

Most studies and clinical experience show that the majority of IC patients (some estimates suggest over 80%) have a hypertonic, non-relaxing pelvic floor. This is often a key driver of their pain.

**Increased Pain:** Forcefully contracting already tight muscles can trigger direct pain, replicate your bladder pain, or cause spasms.

**Worsened Urgency/Frequency:** Tight muscles can compress the urethra and irritate bladder nerves, sending false signals that the bladder is full.

**Reduced Blood Flow:** Chronic tension starves the tissues of oxygen, leading to more pain and dysfunction.

**What You Likely Need Instead: Pelvic Floor *Relaxation***

For a tight pelvic floor, the effective "exercise" is learning to lengthen, relax, and coordinate these muscles. This is often called **Pelvic Floor Physical Therapy (PFPT)**, but the focus is the opposite of strengthening.

**Key techniques for a hypertonic floor include:**

**Diaphragmatic Breathing (Belly Breathing):** Learning to coordinate your breath so your pelvic floor gently lowers and relaxes on the inhale.

**Reverse Kegels:** A conscious, gentle lengthening and releasing of the pelvic floor, like letting go of a held pee. This is the *opposite* of a Kegel.

**Stretching:** Yoga poses like Child's Pose, Happy Baby, or Squat (Malasana) to release hip and pelvic tension.

**Trigger Point Release:** A skilled therapist uses internal or external manual pressure to release painful "knots" in the pelvic floor muscles.

**Biofeedback:** Using sensors to see on a screen when your muscles are tight vs. relaxed, helping you learn control.

**The "Mixed" Pelvic Floor**

Some people have a floor that is both tight (spasmed) AND weak. This is common. The tightness prevents the muscle from working efficiently, leading to weakness and incontinence despite the tension.

**The treatment order is critical: You must address the TIGHTNESS first.** Trying to strengthen a tight, spasming muscle will fail and cause pain. Only after you learn to fully relax the floor can you then assess if any true weakness remains and introduce very gentle, specific strengthening.

**The Bottom Line: Get a Proper Assessment**

**Do not assume you need Kegels.** If you start doing traditional pelvic floor strengthening and your IC symptoms get worse (more pain, urgency, or burning), **stop immediately.**

**See a Specialist:** Find a Pelvic Floor Physical Therapist who has experience treating IC/Painful Bladder Syndrome. They can perform an internal and external assessment to determine if your floor is tight, weak, or both.

**Ask the Right Question:** Don't ask "Should I do pelvic floor exercises?" Instead, ask **"Is my pelvic floor too tight or too weak, and what type of retraining do I specifically need?"**

For the majority of IC warriors, the path to relief is **relaxation and stretching**, not strengthening.

**45.Can bladder training help people with interstitial cystitis urinate less often?**

Yes, bladder training can be an effective strategy to help people with interstitial cystitis (IC) urinate less often. It works by gradually re-educating the bladder to hold more urine for longer periods, decreasing the overall number of bathroom visits.

Here is a breakdown of how it works and the evidence that supports it:

**How It Works**: Bladder training, also known as bladder retraining, involves a structured plan to gradually increase the time between bathroom visits. For example, if a patient is urinating every 30 minutes, they may start by extending that interval to 45 minutes, and then to an hour as they progress. The primary goal is to stretch the bladder, improve its capacity, and reduce the constant feeling of urgency.

**The Evidence is Strong**: Medical guidelines and professional organizations, including the VA, NICE, and MSD Manuals, often recommend behavioral strategies like bladder training as first-line treatments to manage the symptoms of interstitial cystitis.

**Impressive Clinical Results**: Clinical studies have demonstrated significant benefits from using this technique:

**Data from a study** showed that 71% of patients (15 out of 21) who stuck with a bladder training program successfully managed their symptoms. The same study found that the average number of daily urinations decreased from 13.2 before training to 7.4 after training.

**Another study** (Hsieh et al., 2008) reported a significant increase in bladder capacity and a decrease in urination frequency. Daytime urination frequency dropped from an average of about 14.7 times to just 6.9 times per day following treatment.

**Important Considerations**: It is crucial to follow a bladder retraining program under the guidance of a healthcare professional. This is especially important if you experience significant pain, as research suggests that training may be less successful for those with severe IC-related pain.

In summary, bladder training is a proven, non-invasive, first-line behavioral therapy that can help people with interstitial cystitis urinate less often by increasing bladder capacity and reducing the sensation of urgency. However, it is most effective when started under medical advice.

I hope this information is helpful. Are you interested in learning about other lifestyle modifications or treatments that can help manage interstitial cystitis?

**46.Can bladder medicine placed through a tube help interstitial cystitis symptoms?**

Yes, bladder medicine delivered through a tube directly into the bladder (known as intravesical instillation) can be an effective way to manage interstitial cystitis (IC), especially for patients who haven't found relief with other treatments.

This method allows high concentrations of medication to target the bladder wall directly, often with fewer systemic side effects than oral medications. Several types of medicines are used this way for IC:

**Types of Medications and Their Efficacy**

**Dimethyl Sulfoxide (DMSO)**: This is a well-established and FDA-approved therapy for IC. A 2025 systematic review and meta-analysis found it is effective in improving bladder pain and overall symptoms. The study showed it can reduce the Interstitial Cystitis Symptom Index Score by an average of **5.59 points**, with 63% of patients showing significant improvement in one trial. A common but harmless side effect is a temporary garlic-like taste and odor on the breath and skin.

**Heparin**: This medication is often used to help restore the protective lining of the bladder. Studies support its use, with one showing that **76.7%** of patients responded positively to weekly treatments. The beneficial effects of a 12-week course of therapy can be maintained for up to **6 months** after treatment ends. It is often combined with other agents.

**Lidocaine**: A local anesthetic that can provide rapid relief from pain. Research suggests that when combined with heparin and sodium bicarbonate, it can be effective. However, it's important to note that while early studies were promising, recent large-scale trials for a specific lidocaine-releasing device showed no benefit over placebo, indicating that *how* the lidocaine is delivered is critical for its effectiveness.

**Pentosan Polysulfate (PPS)**: Best known as the oral medication Elmiron, PPS is also being studied for intravesical use. Early studies, particularly with a newer liposome-encapsulated form, have shown promising results, including significant decreases in pain and urgency with no side effects.

**Hyaluronic Acid (HA) / Chondroitin Sulfate**: These medications help replenish the bladder's protective glycosaminoglycan (GAG) layer. They are often used in combination and have shown positive results in research, including improving bladder pain scores, particularly when used together.

**Resiniferatoxin**: This is considered a very effective treatment in some studies. One network meta-analysis ranked it as the most effective therapy for improving symptom scores. It is often used after other treatments have not been successful.

**Combination Therapy**: A crucial takeaway from recent research is that a **combination of therapies** is often necessary to manage the complex symptoms of IC effectively. No single treatment works for everyone, and many patients do better with a multi-pronged approach.

**What to Expect with the Procedure**

The procedure is typically done in a doctor's office or clinic and is relatively straightforward.

**The Setup**: You will be asked to empty your bladder and then lie on an exam table.

**The Instillation**: A thin, flexible tube called a catheter is gently passed through your urethra and into your bladder. The medication is then slowly dripped through the catheter.

**Dwell Time**: Once the medication is in, the catheter is removed. You will be asked to hold the medication in your bladder for a set period, typically between **30 and 60 minutes**, to allow it to work. After this "dwell time," you can empty your bladder normally.

Treatment courses vary but often involve one instillation per week for several weeks (e.g., 6-12 weeks), sometimes followed by less frequent maintenance treatments.

**Summary**

To summarize, intravesical therapy is a valuable and effective tool for managing interstitial cystitis, particularly for symptoms related to pain and urinary urgency. The key is to understand that IC is a complex condition, and treatment often requires a personalized, multi-modal approach. Discussing the potential benefits and side effects of these bladder instillations with your healthcare provider is the most important step toward finding the right treatment for you.

If you're considering this path, I would be happy to help you research specific medications or look for recent trials in your area. Just let me know.

**47.Is major surgery usually needed for interstitial cystitis?**

No, major surgery is not usually needed for interstitial cystitis (IC). It is considered a last-resort treatment reserved for a small number of patients with the most severe, disabling symptoms that have not responded to all other less invasive therapies.

**Surgery is a Last Resort**

Treatment for IC follows a stepwise approach that begins with the least invasive therapies, progressing to surgery only when all other options have failed.

**Common initial treatments**: These include dietary changes, stress reduction, pelvic floor physical therapy, oral medications, and bladder instillations (where medication is placed directly into the bladder).

**Surgery is rarely performed**: Because of the significant risks involved, which can include infection, the need for lifelong self-catheterization, and the possibility that symptoms may not improve or could worsen.

**Types of Major Surgery**

If surgery is considered, the specific type depends on your unique situation and the nature of your symptoms. Major surgical procedures for IC fall into three main categories.

| Procedure | Description |
| --- | --- |
| **Bladder Augmentation (Cystoplasty)** | The bladder is enlarged using a segment of the intestine to increase its capacity and lower pressure, which can help reduce pain and urinary frequency. |
| **Urinary Diversion** | The flow of urine is rerouted away from the bladder, typically using a piece of the intestine to create a new pathway (a stoma) through the abdominal wall. |
| **Bladder Removal (Cystectomy)** | The bladder is surgically removed. This is the most aggressive procedure and is often performed at the same time as a urinary diversion. |

**What are the Potential Outcomes?**

While major surgery for IC can be effective, outcomes are not guaranteed.

**Rates of success**: For a urinary diversion with bladder removal, studies have shown a success rate of up to **96%**, with some patients reporting being pain-free and satisfied after surgery. However, success rates can vary, and some studies report lower long-term success rates of around **24%** after certain procedures.

**Risks and considerations**: It is important to remember that:

Surgery does not guarantee symptom relief. Some patients may not improve, or their symptoms could become worse.

Major bladder surgery is irreversible.

Post-surgical complications are possible. For example, bladder augmentation may require lifelong self-catheterization to empty the bladder.

**Summary**

Major surgery for interstitial cystitis is a significant and irreversible step that is reserved for a minority of patients. Because there is no cure for IC, treatment focuses on managing symptoms. If you are interested in exploring whether surgery could be an option, speaking with a urologist who specializes in IC is the best next step.

I hope this information is helpful as you discuss your treatment options with your healthcare team.

**48.Can interstitial cystitis affect sexual life?**

Yes, interstitial cystitis (IC), also often called bladder pain syndrome (BMS), can significantly affect sexual life. This is a very common and distressing concern for many people with IC, but it's important to know that help and strategies are available.

Here’s a breakdown of how IC affects sexual activity and what can be done about it.

**How IC Affects Sexual Life**

The impact is typically a combination of physical symptoms and emotional consequences.

**1. Physical Pain During or After Sex (Dyspareunia)**
This is the most direct effect. For many, sexual intercourse can trigger:

**Burning or sharp pain** in the bladder or urethra during penetration or orgasm.

**Deep pelvic pain** with thrusting, as the movement can jostle an already inflamed bladder.

**A severe symptom flare** that begins a few hours after intercourse (sometimes called "honeymoon cystitis," though it's different from a bacterial infection). The pain can last for many hours or even days.

**2. Impact on Desire and Arousal**
When you consistently expect pain after sex, it's natural for sexual desire to decrease. The mind begins to associate sex with suffering, not pleasure. This can lead to:

**Loss of libido:** Actively avoiding sexual situations.

**Difficulty with arousal:** Pain and anxiety can make it hard to relax and become physically aroused.

**3. Relationship Strain**
IC can put stress on intimate relationships. Partners may feel rejected, confused, or frustrated. A lack of open communication about the pain can lead to misunderstandings, guilt, and emotional distance.

**4. Specific Symptoms by Anatomy (for women, as IC is more common in women)**

The bladder sits directly in front of the vagina. An inflamed bladder is easily irritated by pressure or movement from the vaginal wall.

Many women with IC also have **pelvic floor muscle dysfunction** (chronically tight, spasming muscles). These muscles contract painfully during attempted penetration, often causing a burning or ripping sensation at the vaginal opening.

**(For men)** IC can cause testicular, scrotal, or penile pain, as well as pain during or after ejaculation.

**What Can You Do? Strategies for Managing Sexual Health with IC**

The most important message is that **you don't have to accept a painful sex life as your new normal.** Many people with IC successfully adapt their intimate lives.

**1. Talk to Your Doctor (Crucial First Step!)**

**Rule out other causes:** Ensure the pain isn't from a chronic infection, endometriosis (in women), or pelvic floor disorder.

**Get a referral to a pelvic floor physical therapist.** This is often the single most effective treatment for IC-related sexual pain. They can release tight muscles and teach you relaxation techniques.

**Ask about treatments for flares:** Your doctor can prescribe "rescue instills" (medication put directly into the bladder) or medications to take before sex to prevent a flare.

**2. Adapt Your Sexual Practices**

**Empty your bladder immediately before sex.**

**Take a warm bath beforehand** to relax pelvic muscles.

**Use lots of lubricant.** This reduces friction and irritation.

**Try different positions.** Positions that allow you to control the depth and angle of penetration are best. Woman-on-top or side-lying positions often put less pressure on the bladder. Shallow penetration can be a game-changer.

**Focus on non-penetrative activities.** Many people with IC find that outercourse (mutual masturbation, oral sex, using external vibrators, sensual massage) is completely pain-free and highly satisfying.

**Use an ice pack or heating pad** on the pelvic area after sex to reduce inflammation.

**3. Manage Your IC Overall**
Better-controlled IC generally means better-controlled sexual pain.

Follow your IC diet and medication plan.

Practice stress management (yoga, meditation, therapy), as stress directly tightens the pelvic floor.

**4. Communicate with Your Partner**

Be honest. Say, *"I want to be close to you, but penetration causes me pain. Can we explore other ways to be intimate tonight?"*

Have a code word for "stop, this is starting to hurt."

Involve your partner in your treatment journey (e.g., go to a doctor's appointment together).

**5. Seek Support**

A **sex therapist** or **counselor** who specializes in chronic pain can help you and your partner rebuild intimacy and break the fear-pain cycle.

Online support groups (like from the Interstitial Cystitis Association) are full of people who share practical tips that have worked for them.

**In Summary**

**Yes, interstitial cystitis very commonly affects sexual life**, causing physical pain during/after intercourse and leading to loss of desire and relationship stress. However, it does **not** have to mean the end of a satisfying intimate life.

With the right medical team (especially a pelvic floor physical therapist), open communication, and a willingness to adapt and explore non-penetrative options, most people with IC can find a path to a pleasurable and pain-free sexual relationship.

**The key first step is talking to your doctor and asking for a pelvic floor physical therapy referral.** You don't have to suffer in silence.

**49.What can someone do if interstitial cystitis causes pain during sex?**

This is a very common and challenging issue. While there's no single cure, a combination of strategies can significantly help reduce pain and restore a satisfying sex life. The most effective approaches target the pelvic floor muscles and involve careful planning.

**1. Underlying Symptom Management**

First, work with your doctor to get your baseline bladder symptoms under control. Better daily management often leads to fewer flares triggered by sex.

**Oral Medications**: Drugs like NSAIDs (e.g., ibuprofen) can reduce pain, while antihistamines or tricyclic antidepressants (e.g., amitriptyline) can relax bladder muscles and reduce pain signals.

**Bladder Instillations (Bladder Wash)**: This involves placing a solution of medication (e.g., DMSO, lidocaine, or heparin) directly into the bladder via a catheter to reduce inflammation and pain.

**Dietary Adjustments**: Identify and avoid dietary triggers that irritate the bladder, such as caffeine, carbonated drinks, alcohol, citrus fruits, spicy foods, and acidic foods. The Interstitial Cystitis Network ([icnetwork.org](https://icnetwork.org/)) or the Interstitial Cystitis Association ([ichelp.org](https://ichelp.org/)) provide detailed food lists.

**Bladder Retraining**: With the help of a therapist, you can learn to increase the time between bathroom trips by gradually holding urine for longer periods.

**Stress Management**: Since stress can worsen symptoms, techniques like meditation, deep breathing, or yoga can be very beneficial.

**️ 2. Pelvic Floor Physical Therapy (Key Treatment)**

Many IC patients have pelvic floor muscles that are too tight or in spasm, which is a primary driver of pain.

**Goal**: Physical therapy for IC aims to *relax and lengthen* the pelvic floor muscles and release "trigger points" (painful knots), rather than just strengthening them.

**Techniques**: This often involves **hands-on treatment**, including both external and internal myofascial release, trigger point therapy, and biofeedback. A physical therapist can also look at how your posture affects your pelvic floor.

**Finding a Specialist**: This is not general physical therapy. It's essential to find a therapist specially trained in **pelvic floor therapy**. Organizations like the [Interstitial Cystitis Association](https://www.ichelp.org/) or the [Pelvic Health and Rehabilitation Center](https://pelvicpainrehab.com/) can help you locate a specialist.

**️ 3. Planning and Preparation**

Since IC pain is often predictable, you can plan your intimate moments to reduce discomfort.

**Track Your Flares**: Keep a journal to identify patterns. Many people find that pain from sex is most intense in the 24-48 hours *after* activity, so planning accordingly can help manage post-sex flares.

**Time it Right**: Plan sexual activity for when you're feeling your best. Many find it helpful to be intimate **after** a helpful treatment like physical therapy, a bladder instillation, or after taking oral pain medication.

**Empty Your Bladder**: Always urinate immediately before and after sex to minimize bladder pressure and reduce the risk of any urinary tract infection.

**Use Lubrication**: Friction can worsen pain. Use a high-quality, gentle lubricant. **Water-based** lubricants are often recommended and are safe with condoms. Avoid any with glycerin, sorbitol, or oil if you are prone to UTIs.

**️ 4. Trying Different Sexual Activities**

Redefining intimacy and prioritizing pleasure without pressure is crucial.

**Redefine Intimacy**: Focus on what feels good and doesn't cause pain. Research shows that **65% of IC patients can enjoy foreplay and orgasm without pain**.

**Try Outercourse**: Exploring non-penetrative sex and clitoral stimulation (which is often pleasurable for women with pelvic pain) can be very fulfilling.

**Experiment with Positions**: Certain positions allow you to control depth and pressure. Positions that are often more comfortable include:

**Woman on top**: Gives you maximum control over the depth and angle of penetration.

**Side-by-side (spooning)**: This position generally allows for shallower penetration and less abdominal pressure.

**At the edge of the bed**: Similar to side-by-side, it can reduce deep contact.

**Use Comfort Measures**: Apply a warm or cold compress to your pelvic or lower abdomen area before or after sex to help soothe muscles and reduce inflammation.

**️ 5. Communication and Psychological Support**

The impact of IC on relationships can be profound. Over 90% of patients report that their condition has kept them from intimacy with a partner.

**Talk Openly**: Have conversations with your partner about what does and doesn't feel good. Be honest about your pain levels. These conversations are best had outside the bedroom. Using a simple, pre-agreed signal (like a safe word) can be a good way to communicate during the act if something becomes uncomfortable.

**Seek Professional Support**: A **sex therapist** or **counselor** can help you and your partner navigate the emotional and relational challenges of living with a chronic pain condition. They provide tools to communicate better, manage feelings of guilt or rejection, and find new ways to connect.

**6. Professional Medical Treatments**

For persistent pain, more advanced options can be discussed with a specialist.

**Nerve Stimulation**: Techniques like **Transcutaneous Electrical Nerve Stimulation (TENS)** use mild electrical pulses to block pain signals and increase blood flow to the bladder.

**Prescription Topical Gels**: Ask your doctor about **prescription lidocaine gel**. It can be applied to the genital area before sex to numb the tissue and reduce pain associated with penetration, often used in conjunction with a regular lubricant.

**Other Procedures**: In severe cases, doctors may consider options like **Botulinum toxin (Botox) injections** into the bladder or **sacral nerve stimulation** (implanting a small device that sends electrical signals to the nerves controlling the bladder).

**️ 7. When to See a Doctor**

If pain during sex is affecting your quality of life, it is essential to talk to a healthcare professional. You can start with your primary care provider, gynecologist, or urologist. A specialized **urogynecologist** or a **pelvic pain specialist** often has the most experience managing these symptoms.

Managing IC pain during sex is a journey of finding the right combination of strategies for you. For many, relieving pelvic floor muscle tension is the most important step. Please be patient and compassionate with yourself, and don't hesitate to reach out for help from your healthcare team.

I hope this overview provides a helpful starting point. If you'd like to know more about any of these specific strategies, such as how to find a pelvic floor physical therapist, please feel free to ask.

**50.Can interstitial cystitis symptoms get worse after sex?**

Yes, absolutely. For many people with interstitial cystitis (IC), sexual activity—particularly penetrative sex—is a common trigger that can temporarily worsen symptoms.

This is often called a "flare," and it can feel frustrating or discouraging, but understanding why it happens is the first step to managing it.

**Why sex can worsen IC symptoms:**

**Pelvic Floor Muscle Involvement:** This is the most common reason. IC frequently overlaps with pelvic floor dysfunction (tight, tender, or spasming muscles). The muscle contractions and tensing that can occur during arousal or orgasm can trigger these muscles, leading to pain, burning, or urgency after sex.

**Direct Bladder Irritation:** The bladder sits directly in front of the uterus. Deep penetration or certain positions can put physical pressure or cause repetitive "jostling" of an already inflamed and sensitive bladder wall, leading to pain and the sudden urge to urinate.

**Urethral Irritation:** The urethra is close to the vaginal opening. Friction during sex can directly irritate the urethra, producing a burning sensation that mimics a urinary tract infection (UTI), even when no infection is present.

**Histamine Release:** Arousal and orgasm cause the release of histamines and other chemicals in the body. For someone with IC, who may have a hypersensitive bladder lining, this release can contribute to inflammation and a flare of symptoms.

**What does "worse after sex" typically feel like?**

Symptoms can worsen within minutes or may appear many hours later, sometimes even the next morning. Common post-sex flares include:

A sharp or burning pain in the bladder or urethra.

A severe, urgent need to urinate frequently (e.g., every 15-30 minutes).

A deep, achy pelvic pressure.

General pelvic floor muscle tightness and discomfort.

**A note on blood:** While not typical, trace amounts of blood from urethral irritation can occur. *Frank red blood is not normal* and requires a medical evaluation.

**Important distinction: Flare vs. UTI**

It's critical to know the difference because the symptoms are nearly identical. After sex, you are also at risk for a standard UTI.

**IC Flare:** Symptoms come on within minutes to a few hours. A urine dipstick test (like a home UTI test) is often **negative** for nitrites and may have no or only trace leukocytes.

**UTI:** Symptoms may take 24-48 hours to appear. A urine test will be **positive** for nitrites and/or moderate to many leukocytes. **If you have a fever or chills, seek medical care immediately – this is not an IC flare.**

**What can you do to manage or prevent it?**

Many people with IC find ways to maintain a healthy sex life. Strategies include:

**Before Sex:**

Empty your bladder completely right before.

Take a warm bath or use a heating pad to relax pelvic muscles.

Use generous amounts of **lubricant** (avoid scented or "tingling" varieties).

Consider pre-medicating if your doctor agrees (e.g., a dose of a prescribed bladder analgesic like phenazopyridine or an antihistamine like hydroxyzine).

**During Sex:**

**Control depth and position.** Positions that allow you to control depth (like being on top) or avoid deep penetration (like spooning) often help. A "donut" style buffer device can also limit depth.

Communicate openly with your partner about what feels good vs. painful.

Apply ice externally (over clothing) immediately after sex for 10-15 minutes to reduce inflammation.

**After Sex:**

**Do not "power pee."** Sitting and straining to force urine out can worsen pelvic floor tension. Instead, gently try to urinate normally within 30 minutes.

**Do not do harsh pelvic floor exercises (Kegels) immediately after.** These can worsen tight muscles.

Take a baking soda bath or use a peri-wash bottle with cool water to soothe the vulvar area.

Drink a small glass of water with 1/2 teaspoon of baking soda (if safe for you, e.g., no high blood pressure) to help neutralize bladder acidity.

Use an ice pack for acute burning.

**When to talk to your doctor:**

If flares are so severe that you're avoiding sex entirely (this is a quality-of-life issue).

If you are misdiagnosing UTIs and taking unnecessary antibiotics.

If you haven't been evaluated by a **pelvic floor physical therapist**. This is often the single most effective treatment for post-sex flares.

**Bottom line:** Yes, sex is a very common trigger for worsening IC symptoms, mainly due to pelvic floor tension and bladder irritation. This is not "in your head," nor is it inevitable. With the right management strategies—especially pelvic floor therapy—most people can significantly reduce these post-sex flares.

**51.What should someone do if interstitial cystitis symptoms worsen during pregnancy?**

If your interstitial cystitis (IC) symptoms worsen during pregnancy, the most important and safest step is to immediately contact your healthcare provider. Together, you can develop a plan to manage the flare-up safely, often by combining lifestyle adjustments with low-risk medical treatments.

It's important to know that a worsening of symptoms is a common experience—up to 27-35% of pregnant individuals with IC report increased pain during certain trimesters. However, many others find their symptoms stay the same or even improve. The key is to not dismiss your discomfort and to seek help.

Here are the core strategies for managing a flare-up during pregnancy.

**Start with a Medical Evaluation**

Before trying anything new, a medical assessment is essential to rule out other conditions that can mimic an IC flare. Urinary tract infections (UTIs) are more common during pregnancy and have symptoms that can easily be mistaken for a worsening of IC.

**First-Line: Lifestyle and Home Management**

Non-medical approaches are the safest and most recommended first-line treatments for managing IC during pregnancy.

**Review Your Diet**: Foods and drinks are a common trigger for IC flares. Try keeping a symptom diary to identify and avoid any personal triggers, such as caffeine, spicy foods, citrus, or artificial sweeteners.

**Manage Stress**: High stress levels can trigger bladder inflammation and muscle tension, worsening IC symptoms. Incorporate safe relaxation techniques like **prenatal yoga, meditation, or a gentle massage** into your routine.

**Stay Physically Active**: Regular, low-impact exercise can help ease symptoms. Activities like walking, swimming, and stretching are generally considered safe during pregnancy and can be beneficial for IC.

**Wear Comfortable Clothing**: Choose loose, non-restrictive clothing to avoid placing unnecessary pressure on your bladder and pelvic area.

**Apply Warm Compresses**: Placing a warm compress on your pelvic area can provide soothing relief from pain and discomfort.

**Second-Line: Medications and Therapies**

If lifestyle changes aren't enough, your doctor may recommend specific, pregnancy-safe treatments.

**Pain Relief**: If you need a pain reliever, **Acetaminophen (Tylenol)** is generally considered safe for use during pregnancy and can be an option for managing IC pain. **Always consult your doctor before taking any medication.**

**Intravesical Instillations**: This therapy involves a doctor administering a solution directly into the bladder via a catheter. A common solution contains **hyaluronic acid and chondroitin sulfate**, which are naturally occurring substances that help repair and protect the bladder lining. This treatment can be used during pregnancy to provide gradual but significant symptom improvement.

**Oral Medications**: In some cases, an oral medication might be prescribed.

**Hydroxyzine**: This is an antihistamine that can help with bladder issues and is an option your doctor may consider.

**Elmiron (Pentosan Polysulfate Sodium)**: This is a class B drug specifically used to treat IC. Some obstetricians consider it safe for use during pregnancy as it may not cross the placenta, but it is essential to discuss the risks and benefits with your doctor.

**️ Specialist Therapies**

Pelvic floor physical therapy can be highly effective for managing IC. A specialized therapist can teach you:

**Techniques to relax and strengthen your pelvic floor muscles**, which can help lessen IC symptoms and improve bladder control.

**Biofeedback** to help you better understand and control your body's responses.

**Bladder training** methods to help you regain control over your urination schedule.

Finding the right combination of strategies may take some time, so be patient with yourself. The most important thing you can do is work closely with your obstetrician and possibly a urogynecologist to create a personalized plan that ensures your safety and comfort throughout your pregnancy.

I hope these suggestions help you have a more comfortable and confident pregnancy.

**52.What can someone do if interstitial cystitis causes anxiety?**

This is a very real and challenging cycle: the chronic pain, urgency, and sleep disruption of IC can absolutely trigger or worsen anxiety, and then that anxiety can make bladder symptoms feel even more intense. Breaking that cycle is key.

Here’s what someone can do, ranging from immediate coping tools to longer-term medical and therapeutic strategies.

**1. First, Address the Immediate Panic/Urgency Cycle**

When anxiety spikes, it often mimics or magnifies IC symptoms (muscle tension, "gotta go" feeling). Try these in the moment:

**Diaphragmatic (Belly) Breathing:** Place a hand on your belly. Inhale for 4 seconds, exhale for 6. Long exhalations activate the parasympathetic nervous system, directly calming the pelvic floor muscles and bladder spasms.

**The "Drop and Sigh" Technique:** Inhale normally, then let your jaw, shoulders, and pelvic floor "drop" on a long exhale sigh. Repeat 5 times.

**Distraction with Temperature:** Splash very cold water on your face or hold an ice cube. This triggers the "mammalian dive reflex," which physically slows down an anxious heart rate.

**2. Treat the Brain-Bladder Connection with Therapy**

Standard "talk therapy" helps, but specific modalities are proven for IC-related anxiety:

**Cognitive Behavioral Therapy (CBT):** The gold standard. A CBT therapist will help you:

Identify catastrophic thoughts ("I'll never leave the house again").

Reframe them ("Right now, I feel an urge, but I just went 90 minutes ago. I can wait 5 more minutes").

Break the avoidance cycle (slowly and safely venturing out with a "bladder map" of restrooms).

**Gut-Directed Hypnotherapy:** Surprisingly effective for IC. It uses relaxation and imagery (e.g., visualizing a cool, soothing lining on the bladder) to reduce pain signals and urgency-driven anxiety.

**3. Use Pelvic Floor Physical Therapy (Crucially Important)**

Many people don't realize that **anxiety causes you to clench your pelvic floor**, which directly irritates an IC bladder. A specialized PT will:

Teach you **reverse kegels** (a conscious letting go of the pelvic floor, the opposite of a kegel).

Show you internal and external trigger point release to stop the muscle spasms that mimic UTI urgency.

Once the pelvic floor relaxes, anxiety often drops significantly within weeks.

**4. Optimize Medications (Work with a Doctor)**

Never stop or start meds without guidance, but these are options to discuss:

**For IC flares that trigger anxiety:** Low-dose amitriptyline (treats both nerve pain and anxiety at night), hydroxyzine (an antihistamine that also treats anxiety and bladder mast cell activation).

**For anxiety alone, without worsening IC:** SSRIs like **sertraline (Zoloft)** or **escitalopram (Lexapro)** are generally bladder-friendly. Avoid **buspirone** (can increase urgency in some) and high-dose benzodiazepines (risk of dependence and rebound anxiety).

**Ask about a rescue med:** A small dose of lorazepam (Ativan) can be used very rarely as a "break glass" tool to stop a severe anxiety-IC flare cycle, but it's not for daily use.

**5. Adjust Lifestyle with Intent (Not Restriction)**

**Diet first, then stress:** Follow the IC diet strictly for 2 weeks to calm the bladder baseline. Then, slowly add back foods. *Constant dietary fear causes more anxiety than the diet helps.*

**Sleep is non-negotiable:** IC + anxiety + poor sleep = disaster. Ask your doctor about low-dose trazodone or gabapentin at night to improve sleep quality without irritating the bladder.

**Move in ways that release tension:** Walking, swimming, or gentle yin yoga (avoid deep crunches or high-impact moves that jar the pelvis).

**6. Build an Anxiety "First Aid Kit" for IC Flares**

Keep a small bag with:

**Baking soda capsules** or **Prelief** (to take before suspect foods).

**A small heating pad** (for the lower belly) or **ice pack** (for the pelvic floor/perineum).

**AZO Urinary Pain Relief** (phenazopyridine) – numbs the bladder lining. Use sparingly.

**A single dose of your preferred anti-anxiety rescue med** (if prescribed).

**A printed "grounding card"** that says: *"This is my bladder, not my life. The feeling will pass in 20 minutes. I can handle 20 minutes."*

**When to Seek Immediate Help**

If you are avoiding drinking water to prevent symptoms (dangerous and makes urine more concentrated/painful).

If anxiety has led to agoraphobia (unable to go to work, grocery shop, or socialize).

If you have thoughts of self-harm to escape the pain-anxiety cycle (call 988 in the US, or your local crisis line).

**The single most powerful thing someone can do is see a urogynecologist or a pelvic pain specialist who treats IC and anxiety as one condition, not two separate ones.** Many general urologists and primary care doctors miss this connection entirely.

You don't have to choose between treating your bladder or your mind. Calming one actively calms the other.

**53.Can counseling help people cope with long-term interstitial cystitis pain?**

This is an excellent and important question. The short answer is **yes, counseling can be a highly effective and often crucial part of coping with long-term interstitial cystitis (IC) pain.**

It's important to clarify what counseling *can* and *cannot* do. It cannot cure the underlying bladder condition. However, for a chronic, unpredictable, and painful condition like IC, counseling addresses the secondary but devastating impacts on mental health, quality of life, and the pain experience itself.

Here’s a breakdown of how counseling helps, the specific approaches used, and the evidence behind it.

**Why Counseling is So Effective for IC Pain**

Chronic pain is not just a physical sensation; it's a complex biopsychosocial experience. IC pain is uniquely challenging because it's linked to urgency, frequency, and fear of not finding a bathroom. This creates a powerful feedback loop:

**Pain & Urgency** -> **Anxiety & Fear** (of pain, of being far from a toilet) -> **Muscle Tension & Hypervigilance** -> **Worsened Pain & Urgency**

Counseling helps break this cycle. It does not "imagine the pain away" but changes your brain's relationship to the pain signals.

**How Specific Therapies Help**

Several evidence-based therapies are particularly effective for IC and other chronic pain conditions:

**1. Cognitive Behavioral Therapy (CBT) - The Gold Standard**

**What it does:** Identifies and changes unhelpful thought patterns ("I'll never get better," "This pain is destroying my life") and behaviors (avoiding all social outings, catastrophizing during a flare-up).

**For IC specifically:** Teaches concrete skills like activity pacing (balancing rest and activity), cognitive restructuring (changing "This is unbearable" to "This is very painful, but I have managed it before and I will get through this flare"), and managing the fear of incontinence or pain during intimacy.

**2. Acceptance and Commitment Therapy (ACT)**

**What it does:** Shifts the goal from *eliminating* pain to *living a full life alongside* the pain. It encourages accepting difficult sensations without a struggle, while committing to actions aligned with your values.

**For IC specifically:** Instead of spending all day trying to suppress urgency and pain (which paradoxically makes you focus on it more), ACT helps you notice the sensation, say "There's that feeling again," and then redirect your attention to making a cup of tea, reading a book, or spending time with family.

**3. Mindfulness-Based Stress Reduction (MBSR)**

**What it does:** Uses meditation and gentle yoga to train focused, non-judgmental awareness of the present moment.

**For IC specifically:** Studies show MBSR can significantly reduce the perceived intensity of pain and the emotional suffering attached to it. It helps lower overall stress reactivity, which is a major trigger for IC flares.

**4. Pelvic Floor Physical Therapy with a Psychological Component**

While not "counseling" per se, many people with IC have hypertonic (chronically tight) pelvic floor muscles. A specialized PT can teach relaxation techniques, and often works in tandem with a counselor to address the anxiety that causes you to clench those muscles unconsciously.

**What the Evidence Shows**

Research on psychological interventions for IC (and related conditions like painful bladder syndrome) is growing and positive:

**Reduced pain and urgency:** Multiple studies show CBT and MBSR lead to statistically significant decreases in self-reported pain and urinary symptoms.

**Improved quality of life:** Patients report less interference from pain in daily activities, work, and social life.

**Lower anxiety & depression:** Rates of major depression and anxiety disorders are very high in IC patients (often >50%). Counseling directly treats these, which in turn lowers pain perception.

**It can be as effective as some medications:** For some patients, a course of CBT provides similar or greater relief from distress than adding another pain medication, without the side effects.

**A Critical Point: Counseling is *for coping*, not for *curing***

It is absolutely vital that your counselor validates the reality of your physical pain. A bad therapist might imply "It's all in your head." A good, chronic pain-informed therapist will say: "Your pain is real. The distress, isolation, and limitation you feel are real. Let's use psychological tools to change how your brain responds to those signals so you can suffer less."

**Practical Advice for Getting Started**

**Find the right specialist.** Look for a:

**Health Psychologist** or **Pain Psychologist** (ideal)

Licensed therapist with specific training in **CBT for chronic pain** or **ACT**

Ask potential therapists: "Do you have experience working with patients with interstitial cystitis, painful bladder syndrome, or chronic pelvic pain?"

**Talk to your urologist.** Ask your IC specialist if they have a referral list of therapists they trust. Many major IC centers (e.g., at Stanford, Cleveland Clinic, Northwestern) have integrated pain psychology programs.

**Consider telehealth.** It's often easier to attend a video therapy session from your own comfortable, private bathroom-accessible space.

**Be patient.** Like physical therapy for a bad back, learning to change your brain's pain habits takes time and practice (typically 8-12 sessions to see significant results).

**The Bottom Line**

**Absolutely, yes.** For someone suffering from long-term IC pain, counseling is not a sign of weakness or a last resort. It is a powerful, evidence-based, proactive tool to reduce suffering, regain a sense of control, and learn to live a richer life even when the pain doesn't go away. It works best as part of a comprehensive plan alongside your urological and dietary management, not instead of it.

**54.How can interstitial cystitis affect work, sleep, and daily life?**

This is an excellent question, as the impact of Interstitial Cystitis (IC), also known as Bladder Pain Syndrome (BPS), often extends far beyond the physical symptom of pelvic pain. It's a chronic condition that can fundamentally alter a person's day-to-day existence. Let's break down how it specifically affects work, sleep, and daily life.

**Impact on Sleep**

Sleep disruption is one of the most common and debilitating aspects of IC, creating a vicious cycle of pain and exhaustion.

**Nocturia (Frequent Nighttime Urination):** The hallmark of IC is urinary urgency and frequency. At night, this means waking up to urinate 5, 10, even 20+ times. This prevents reaching the deeper, restorative stages of sleep.

**Pain that Wakes You:** Even if you don't need to urinate, the persistent burning, pressure, or sharp pain in the bladder and pelvic region can be severe enough to jolt you awake or prevent falling asleep in the first place.

**Difficulty Returning to Sleep:** After a painful trip to the bathroom, the discomfort and stress of knowing you'll have to get up again soon makes it very hard to relax and drift back to sleep.

**The Consequence:** Chronic sleep deprivation leads to extreme fatigue, brain fog, mood swings (irritability, anxiety, depression), weakened immune system, and a reduced ability to cope with pain itself.

**Impact on Work and Career**

IC's unpredictability and physical demands can make traditional work environments extremely challenging.

**Presenteeism (Working while sick):** This is more common than absenteeism. You make it to your desk, but you're exhausted, in pain, and mentally foggy. Your concentration, decision-making speed, and productivity plummet.

**Absenteeism (Missing work):** Severe flare-ups will inevitably lead to calling in sick. Frequent appointments with urologists, pain specialists, physical therapists, or for procedures like bladder instillations also eat up work time.

**Bathroom Access and Logistics:** This is a major hidden hurdle. A job that doesn't allow immediate, unrestricted bathroom access (e.g., teaching, assembly line work, truck driving, surgery, retail) can be impossible. The anxiety of "Can I get to a bathroom in time?" becomes a constant, distracting stressor.

**Physical Demands:** Jobs requiring heavy lifting, long periods of standing (increases pelvic pressure), or sitting on a hard surface (triggers pelvic floor tension) can directly worsen IC pain.

**Career Trajectory:** Some people have to turn down promotions (more stress, longer hours), switch to part-time work, shift to a less demanding role, or leave the workforce entirely, leading to financial strain and loss of identity.

**Impact on Daily Life and Activities**

IC forces a constant process of risk assessment and planning around the bladder.

**The "Bathroom Map":** Any trip outside the home requires knowing the location of every public restroom. This applies to grocery shopping, going to a restaurant, visiting friends, or a walk in the park.

**Dietary Tyranny:** Food becomes the enemy. Common triggers like coffee, tea, soda, alcohol, acidic fruits (citrus, tomatoes), spicy foods, artificial sweeteners, and even chocolate must be avoided. Eating out is a minefield. Social gatherings centered around food become stressful.

**Clothing Choices:** Tight jeans, belts, and synthetic fabrics that press on the lower abdomen can trigger a flare. Wardrobes may shift to loose-fitting pants, leggings, skirts, or dresses made of soft, natural fibers.

**Exercise and Intimacy:**

**Exercise:** High-impact activities (running, jumping) can jar the pelvis. Prolonged sitting on a bike seat is a known trigger. Many patients switch to gentle walking, swimming, or specific pelvic-floor therapy exercises.

**Intimacy:** Sexual activity, particularly intercourse for women (due to the proximity of the bladder and the impact on pelvic floor muscles), can be intensely painful for many with IC. This often leads to avoidance, causing significant strain on romantic relationships and self-esteem.

**Emotional and Mental Toll:** The constant pain, sleep loss, and social limitations lead to high rates of anxiety and depression. The condition is often invisible, leading to feelings of isolation, frustration when others don't understand ("But you look fine!"), and a sense of losing control over one's own body and life.

**Breaking the Vicious Cycle: Hope and Management**

While it's a challenging condition, it's not a life sentence without strategies. The key is a multidisciplinary approach:

**For Sleep:** Work with your doctor on nighttime medications (e.g., low-dose amitriptyline to reduce pain and nerve signals, or medications to reduce nocturia). Establish a strict, relaxing bedtime routine.

**For Work:** Request *reasonable accommodations* under the ADA (if in the US) or similar laws. This could include: a desk closer to a bathroom, a flexible start time (to allow for painful mornings), a stand-sit desk, permission for extra or longer breaks, or a hybrid/remote work arrangement.

**For Daily Life:**

**Find a specialist:** A urologist or gynecologist who specializes in IC.

**Pelvic Floor Physical Therapy:Crucial.** Many IC symptoms are actually driven by super-tight pelvic floor muscles. A PFPT can work wonders.

**Diet:** Follow the IC Diet (start with a very bland elimination diet, then slowly test foods). Resources like the ICN Food List App are invaluable.

**Stress Management:** This is a non-negotiable tool. Stress directly worsens IC. Practice meditation, deep breathing, gentle yoga, or therapy (CBT is very effective for chronic pain).

**Community:** Connect with others via the Interstitial Cystitis Network (ICN) or local support groups to combat isolation and learn practical tips.

In summary, IC is a systemic, life-altering condition, not "just a bladder problem." It attacks sleep, disrupts careers, and shrinks a person's world. However, with aggressive, multi-faceted management, many people regain significant control over their lives, sleep better, work productively, and find new ways to enjoy daily activities. The journey involves a lot of trial and error, but improvement is absolutely possible.

**55.How should interstitial cystitis be managed over the long term?**

Interstitial cystitis (IC) is a chronic condition, and while there is no cure, a variety of effective long-term strategies can help you manage symptoms and improve your quality of life. The goal of long-term management is to find a personalized combination of therapies that works best for you, focusing on symptom relief and preventing flare-ups.

**The Foundation: First-Line & Ongoing Management**

The initial and ongoing approach focuses on behavioral and lifestyle changes. These are the least invasive strategies and form the cornerstone of long-term IC care.

**Diet and Nutrition**: This is often the first step in management.

**Recognize Triggers**: Many people find that certain foods and drinks worsen their symptoms. Common triggers include:

Caffeine, alcohol, and carbonated beverages.

Citrus fruits, chocolate, tomatoes, and spicy foods.

Artificial sweeteners, cured meats, soy, and aged cheeses.

**Find Your Triggers**: The **elimination diet** is a helpful strategy. To do this, stop eating a specific suspected food for a while, then slowly reintroduce it. By tracking your symptoms, you can identify your personal triggers without having to follow a highly restrictive diet forever.

**Bladder Training**: This technique involves gradually increasing the time between bathroom visits. You can learn to delay urination, slowly stretching the time from 30 minutes to several hours. This can help increase your bladder's capacity and reduce urinary frequency.

**Stress and Psychological Support**: Stress is a well-known trigger for IC flare-ups. Practicing stress management through techniques like mindfulness, cognitive-behavioral therapy, or joining a support group can be very beneficial. These strategies provide valuable resources for coping with the condition and connecting with others.

**Medications for Long-Term Symptom Control**

When lifestyle changes aren't enough, several oral medications can help manage pain and urinary symptoms. These are often used for extended periods, so it's important to understand their long-term safety profile.

**Amitriptyline**: A tricyclic antidepressant that is very effective at reducing bladder pain and frequency. Long-term data confirms its usefulness for symptom control, and a key to success is often adjusting to a comfortable dose. It works by blocking pain signals in the nervous system.

**Antihistamines (e.g., Hydroxyzine)**: These can help calm inflammatory cells (mast cells) in the bladder wall, reducing inflammation and pain. They are recommended in current guidelines for symptom relief.

**Pentosan Polysulfate Sodium (Elmiron)**: The only oral medication approved specifically for IC. It works by repairing the protective lining (GAG layer) of the bladder. However, there are significant long-term considerations:

**Effectiveness**: It can take 3-6 months to see an improvement, and its overall clinical benefit is modest.

**Safety Risk**: There is a potential risk of a serious eye condition called **pigmentary maculopathy** with long-term use. Because of this risk, recent guidelines (such as from the CUA) have moved away from routinely recommending this medication. If you are taking it, you will need regular eye exams.

**Other Oral Options**: Other medicines may be used based on your symptoms, including:

**Cimetidine**: An H2 blocker that may improve immune function and symptoms.

**Cyclosporine A (CyA)**: An immunosuppressant reserved for severe, refractory cases. It can provide sustained relief but carries significant side effects that require careful monitoring.

**Intravesical Therapy (Bladder Instillations)**

This involves putting liquid medications directly into your bladder through a catheter. This targets treatment right at the source. These are often used when oral medications aren't enough or cause side effects.

**Dimethyl Sulfoxide (DMSO)**: This is the only FDA-approved intravesical treatment for IC. It's typically given once a week for six weeks and works as an anti-inflammatory agent. It's a well-established treatment option.

**GAG Layer Replenishment**: Therapies like **hyaluronic acid** and **chondroitin sulfate** aim to repair the bladder's protective lining. They are safe and effective for refractory IC, providing longer-term symptom improvement and reducing recurrence, especially after surgery for Hunner lesions.

**Lidocaine**: This is a local anesthetic that can be instilled, often in a cocktail with other agents, to provide rapid, temporary relief from pain and urgency. The Canadian Urological Association lists it as an available intravesical agent.

**️ Advanced Therapies for Refractory Cases**

If first-line treatments don't provide adequate relief, more advanced interventions can be considered.

**Pelvic Floor Physical Therapy**: This is a very effective therapy, often recommended early on and throughout treatment. Pelvic floor muscles can become tight and painful (hypertonic) as a response to chronic bladder pain. Physical therapy, including internal and external myofascial release, can relieve muscle tension and significantly reduce pain.

**Neuromodulation (e.g., Sacral Neuromodulation/SNM)**: This uses a device (like a pacemaker) to alter nerve signals between the spinal cord and the bladder.

**Long-term efficacy**: Studies show SNM can provide significant, sustained improvements in pain, urinary symptoms, and quality of life for people with severe, treatment-resistant IC. In one study with a median follow-up of 35 months, pain scores dropped from 8/10 to 3/10, and many patients were able to stop taking opioid pain medication.

**How it works**: An implantable device stimulates the sacral nerves to help "reset" dysfunctional pain signaling, providing a minimally invasive option.

**Surgical Procedures**: Surgery is generally reserved for specific situations.

**For Hunner Lesions**: Some people have Hunner lesions, which are specific inflammatory spots in the bladder wall. These can be treated effectively with **fulguration** (burning of the lesions) or **triamcinolone injections** (steroid injections) during a cystoscopy. These procedures, often combined with hydrodistention (stretching the bladder), can provide long-term symptom relief.

**Major Surgery**: Radical surgery, such as bladder removal (cystectomy), is an absolute last resort, considered only when all other treatments have failed and the patient's quality of life is unbearable.

**Complementary and Alternative Medicine (CAM)**

Many people find additional relief from mind-body and other therapies. While evidence quality varies, they are promising options.

**Acupuncture**: Some studies associate acupuncture with a reduction in pain and may be effective for managing symptoms. The effect may be most noticeable in the first few months of treatment.

**Mind-Body Interventions**: Practices like **yoga, mindfulness-based stress reduction (MBSR), and hypnosis** can be powerful adjuncts to standard care. They help improve symptoms, induce relaxation, and give patients a greater sense of control over their condition.

**️ The Importance of a Long-Term Plan**

**Recognize the Chronicity**: It's crucial to understand that IC is a long-term condition. Your symptoms may come and go, but the goal of treatment is sustained management and the prevention of flare-ups. Work closely with your healthcare provider to develop a personalized plan that evolves with your needs.

**Monitor Your Response**: The 2022 AUA guidelines suggest abandoning any treatment that hasn't provided meaningful improvement after an adequate trial (typically at least 6 months for a therapy like Elmiron). This ensures you don't waste time on ineffective treatments.

**Set Realistic Goals**: The primary goal of treatment is not a "cure" but to achieve a **50% or greater improvement** in your symptoms. Research indicates that about half of IC patients achieve this level of relief in the long term, even after trying multiple treatments.

In summary, a comprehensive long-term plan incorporates a step-wise process:

**Build Your Foundation**: Start with diet, bladder training, and stress reduction.

**Add Medication**: If needed, try oral medications, carefully weighing the long-term risks and benefits.

**Explore Local Therapies**: Consider intravesical instillations or physical therapy.

**Consider Advanced Options**: For refractory symptoms, talk to your urologist about neuromodulation or procedures for Hunner lesions.

**Embrace a Holistic View**: Use mind-body practices and CAM therapies to support your overall well-being.

No single treatment works for everyone. Finding the right combination of lifestyle adjustments, medications, and therapies is often an exercise in trial and error. Work in partnership with your healthcare team to find the path that brings you the best long-term relief and quality of life.

**56.Should people with interstitial cystitis keep following their care plan after symptoms improve?**

This is an excellent and important question. The short answer is **yes, generally people with interstitial cystitis (IC/BPS) should continue following their care plan even after symptoms improve.**

However, it's not about sticking to every detail rigidly. It's about shifting from an *active treatment* phase to a *maintenance* phase. Here’s a breakdown of why and how.

**Why Continuing Care is Crucial**

**IC/BPS is a Chronic Condition:** Like asthma, diabetes, or high blood pressure, IC/BPS is typically a lifelong condition with periods of remission (low or no symptoms) and flare-ups. Stopping all management strategies greatly increases the risk of a return to severe symptoms.

**Preventing Relapse:** Your current improvement likely means your care plan is *working*, not that the underlying condition is cured. The diet, stress management, pelvic floor exercises, or medications are actively suppressing the mechanisms that cause your pain and urgency. Stopping them removes that protection.

**Identifying Your "Baseline" Maintenance:** The goal of treatment is to find the minimum effective dose of care that keeps you feeling good. This is far easier to figure out when you're feeling well than when you're in a crisis.

**How the Care Plan Changes (The "Maintenance vs. Active" Phase)**

Your care plan shouldn't be a static list of "do this forever." It should evolve. When symptoms improve, you and your doctor should work on **stepping down** to a maintenance plan.

| Aspect of Care Plan | During a Flare (Active Phase) | After Improvement (Maintenance Phase) |
| --- | --- | --- |
| **Diet** | Strictly avoid all trigger foods (tomatoes, citrus, coffee, alcohol, spicy food). Eat only "bladder-friendly" foods. | Carefully and slowly reintroduce *one* potential trigger food at a time to find your personal tolerance level. Most people have a few specific triggers but can eat others. |
| **Medications** (e.g., Elmiron, hydroxyzine, amitriptyline) | Taken at full prescribed dose. | Continue the full dose that achieved remission. Do not stop without doctor's guidance, as this is a common cause of relapse. A dose *reduction* might be tried after long-term stability. |
| **Supplements** (e.g., marshmallow root, aloe vera) | Taken as needed for symptom relief. | May be reduced to a "maintenance dose" or taken only as a preventative before known triggers (e.g., a glass of wine). |
| **Pelvic Floor Therapy** | Frequent sessions (weekly). | Reduce to maintenance sessions (e.g., once a month or every 6 weeks) or continue daily home stretches/exercises to prevent muscle tension from returning. |
| **Stress Management / Hydration** | Top priority. | Remains a crucial daily habit. Stress is a top trigger for most people. Continue good hydration habits (sipping water all day). |

**What You Should Do Differently When Feeling Better**

**Schedule a "Maintenance Review" with Your Doctor:** Don't just disappear. Tell them, "I feel great now. Can we discuss a long-term maintenance plan?" This is the perfect time to ask about reducing the frequency of instillations or trying to lower a medication dose.

**Create a "Flare Action Plan" While You're Calm:** Work with your doctor to write down exactly what you will do *if* your symptoms start to return. (e.g., "If I have urgency for two days, I will go back to the strict diet for 5 days and take an extra hydroxyzine at night.") Having this plan prevents panic and gets you back on track faster.

**Do the "Trigger Challenge" Carefully:** The goal is quality of life. If you miss coffee, try a small, low-acid cup *after* a week of zero symptoms. Drink it with food and a large glass of water. Note the effect over the next 48 hours. This is how you build your personal "safe foods" list.

**Don't Stop Pelvic Floor Relaxation:** Many people think, "I'm not in pain, so my muscles are fine." But years of pain can create a habit of clenching. Continue your daily diaphragmatic breathing and pelvic floor drops, even for just 2 minutes. It's excellent prevention.

**The One Major Exception: Following the advice to *modify* the plan, not abandon it.**

**Do not suddenly stop prescription medications** (especially amitriptyline, which can have withdrawal effects) or drastically change your hydration/diet without a doctor's guidance. Sudden changes are the #1 cause of a brutal flare.

**Practical Summary: What to Do Right Now**

**Keep the core:** Stay on your effective medication dose. Keep your pelvic floor relaxed daily.

**Loosen the details:** Test a single, small serving of a former trigger food. See what you can safely add back.

**Plan for the bad days:** Have a clear, written plan for how to react at the *first sign* of a flare.

**Talk to your doctor:** Ask specifically, "What does *my* maintenance phase look like?"

**Final Verdict:** Don't follow your flare-phase care plan forever. That's needlessly restrictive. But don't throw the whole plan away. **Convert it into a smarter, easier, long-term maintenance plan.** This is the key to staying symptom-free for months or years, rather than bouncing between feeling good and suffering a severe relapse.

**57.Should online claims about a complete cure for interstitial cystitis be trusted?**

No, online claims about a complete cure for interstitial cystitis (IC) should not be trusted. The evidence from major urological associations and medical experts is clear: **while symptoms can be managed, there is currently no known cure for IC.**

**The Medical Consensus: Treatment, Not Cure**

The current scientific and medical consensus is that interstitial cystitis is a chronic condition that can be effectively managed, but not cured. Multiple authoritative sources, including the Merck Manual, the National Kidney Foundation, and the Interstitial Cystitis Association, all agree that no cure exists, although most patients can achieve significant symptom relief through various treatments.

This reality is reflected in official treatment guidelines from organizations like the American Urological Association (AUA). These guidelines focus on a multi-modal approach to manage the condition. This includes first-line behavioral changes (such as diet modification and bladder training), oral medications, intravesical therapies (instilling medication directly into the bladder), and neuromodulation. The goal is to create a personalized treatment plan that alleviates symptoms and improves quality of life, not to provide a permanent "cure".

**Red Flags of Health Fraud**

The deceptive promise of a "quick fix" or "miracle cure" is a classic hallmark of health fraud. Being able to recognize these red flags can help you avoid ineffective and potentially dangerous products:

**Guarantees of a Cure:** Claims of a "quick, effective cure-all" for a wide range of ailments are a major warning sign. Be especially wary of products promoted with words like "scientific breakthrough," "miracle cure," "secret ingredient," or "ancient remedy".

**"Natural" Scams:** Beware of products marketed as a "natural" cure, where a dietary supplement is falsely presented as a proven medicine.

**Pressure to Buy:** Be highly skeptical of aggressive marketing tactics that pressure you into an immediate purchase, as this is often a technique used by scammers.

**How to Find Reliable Information**

Protecting yourself starts with knowing where to find trustworthy health information. You should always consult licensed medical professionals. To verify online claims, use the following checklist based on advice from the FDA:

**Source:** Is the information from a reputable organization like the National Institutes of Health (NIH), the AUA, or an academic medical center? Is it published in a peer-reviewed journal?

**Evidence:** Does the claim reference peer-reviewed studies, clinical guidelines, or systematic reviews? Avoid testimonials or personal anecdotes as the primary form of evidence.

**Consistency:** Is the message consistent with the consensus from other major health organizations? Any treatment that sounds "too good to be true" almost certainly is.

**Anonymity:** Is the source anonymous or is the author's expertise difficult to verify?

**Conflict of Interest:** Is the primary purpose of the information to sell a product?

**Summary**

In short, any online claim of a "complete cure" for interstitial cystitis should be treated as false. The condition is currently manageable, not curable. Always rely on the established consensus from official medical guidelines and consult with a healthcare provider to build a safe and effective long-term management plan.

I hope this information helps you navigate these claims safely. Is there anything else about IC/BPS I can help you with?

**58.Is it safe to diagnose or treat interstitial cystitis only with advice from the internet?**

This is an excellent and important question.

The short answer is **no, it is not safe to diagnose or treat Interstitial Cystitis (IC) based solely on advice from the internet.**

Here’s a detailed breakdown of why, and the significant risks involved.

**The Core Problem: IC is a "Diagnosis of Exclusion"**

IC is a complex, poorly understood chronic bladder condition. There is no single test (like a blood test or a scan) that can definitively say "you have IC." Instead, a doctor diagnoses it by:

**Taking a detailed history** (symptoms, pain patterns, urinary frequency, triggers).

**Performing a physical exam** (often including a pelvic exam for women).

**Running tests to *rule out* all other possible causes** of your symptoms. This is the most critical step.

Other conditions that can cause *exactly the same symptoms* as IC include:

**Urinary Tract Infections (UTIs)** (especially chronic or embedded ones)

**Bladder Cancer** (rare, but symptoms overlap)

**Kidney Stones**

**Endometriosis** (in women)

**Chronic Pelvic Pain Syndrome**

**Overactive Bladder**

**STIs (e.g., Chlamydia, Herpes)**

**The Danger:** If you follow internet advice for "IC" but actually have an untreated UTI, kidney stone, or even bladder cancer, you could delay life-saving or kidney-saving treatment. A misdiagnosis from the internet cannot rule out these dangerous conditions.

**Risks of Diagnosing Yourself with IC Online**

**Missing a Curable or Dangerous Disease:** This is the #1 risk. Treating "IC" with diet changes and supplements while a treatable infection spreads or a tumor grows is extremely dangerous.

**Anxiety and Distress:** The internet is full of worst-case scenarios, conflicting information, and unmoderated patient forums. Reading about severe, unrelenting pain can cause significant health anxiety, which can actually worsen urinary symptoms (a mind-body connection).

**Wasting Time and Money:** You might spend months and hundreds of dollars on unproven "internet protocols," supplements, and restrictive diets, only to find out later you had a simple condition that required a week of antibiotics.

**Risks of Treating Yourself with Advice from the Internet**

Even if you have been *properly* diagnosed by a doctor, blindly following online treatment advice is risky.

**Harmful Supplement Interactions:** Many online "cures" involve high-dose supplements (e.g., quercetin, aloe vera, L-arginine). These can interact with prescription medications (e.g., blood thinners, diabetes meds) or cause liver/kidney damage, especially at the high doses recommended in forums.

**Delaying Proven Treatments:** Internet forums often demonize effective, doctor-supervised treatments (like prescription medications, bladder instillations, or neuromodulation) as "toxic" or "dangerous." Relying only on unproven home remedies can delay you from getting real relief.

**Extreme and Unsafe Diets:** The "IC diet" often recommends eliminating nearly everything. Long-term, this can lead to nutritional deficiencies, disordered eating, and unnecessary food fear. A doctor or dietitian can help you do a *structured, safe elimination diet* to find your triggers without harming your health.

**False "Cures" and Scams:** The internet is full of people selling expensive "guaranteed cure" programs, devices, or supplements for IC. There is no cure. Anyone claiming otherwise is lying to take your money.

**How the Internet *Should* Be Used Safely**

The internet is a powerful tool for **information and support**, but it must be used *in partnership with a real doctor*.

**DO use it to:** Research your doctor's suggestions (e.g., "What are the side effects of Elmiron?").

**DO use it to:** Find reputable support groups (like the ICA or ICN) for coping strategies from other diagnosed patients.

**DO use it to:** Prepare questions for your next doctor's appointment.

**DO NOT use it to:** Replace a doctor's diagnosis, decide on a treatment plan, or stop/start medications.

**What You Should Do Instead**

**See a Urologist (preferably one specializing in pelvic pain or IC).** This is non-negotiable. They will run the necessary tests (urinalysis, urine culture, cytology, possibly a cystoscopy) to rule out other conditions.

**Keep a Bladder Diary** (when you pee, how much, pain level, what you ate/drank). This is incredibly helpful for the doctor.

**Be open-minded.** Your problem might be IC, but it might also be something much simpler and more treatable. Let the doctor do their job.

**Summary**

| **Action** | **Safe?** | **Why?** |
| --- | --- | --- |
| **Diagnosing yourself with IC from internet symptoms** | **NO - Dangerous** | You cannot rule out bladder cancer, infections, or stones. |
| **Treating "IC" with unproven internet protocols** | **NO - Risky** | Supplements can be harmful, diets can be extreme, and you delay effective care. |
| **Using the internet to support a doctor's treatment plan** | **YES - Helpful** | Great for learning coping skills, recipes, and medication side effects. |

**Ultimately, interstitial cystitis is a real, painful medical condition that requires a real medical diagnosis and a supervised treatment plan. Please do not risk your health with unverified online advice. See a doctor as your first and most important step.**

**Doubao**

**1. What is interstitial cystitis?**

Interstitial Cystitis/Bladder Pain Syndrome (IC/BPS)

Interstitial cystitis (IC), now officially called **interstitial cystitis/bladder pain syndrome (IC/BPS)** by major medical organizations, is a chronic, often debilitating condition characterized by persistent pelvic pain and lower urinary tract symptoms in the absence of identifiable causes like infection, cancer, or stones .

Key Characteristics

**Duration**: Symptoms last for at least 6 weeks

**Hallmark symptom**: Bladder pain, pressure, or discomfort that **worsens as the bladder fills** and typically improves after urination

**Urinary symptoms**:

Urinary frequency (≥8 times per day)

Urinary urgency (sudden, intense need to urinate)

Nocturia (waking ≥2 times per night to urinate)

**Pain location**: Primarily suprapubic (above the pubic bone), but may radiate to the urethra, vagina, perineum, lower back, or thighs

**Variable course**: Symptoms often wax and wane, with periods of remission and exacerbation

Epidemiology

Affects approximately **0.5-1%** of the general population

**90% of patients are women**

Typical onset between ages 20-50, but can occur at any age

Average delay in diagnosis: **3-7 years** from symptom onset to specialist confirmation

Subtypes

IC/BPS is now classified into two distinct subtypes based on cystoscopic findings, which have different pathophysiologies and treatment responses :

| **Subtype** | **Prevalence** | **Key Features** |
| --- | --- | --- |
| **Hunner Lesion (Ulcerative) IC/BPS** | 5-10% of cases | Distinct inflammatory lesions on bladder wall; more severe inflammation; often responds better to surgical interventions |
| **Non-Hunner Lesion (Non-Ulcerative) IC/BPS** | 90-95% of cases | No visible ulcers; may show glomerulations (pinpoint bleeding) after hydrodistention; often associated with pelvic floor dysfunction and central sensitization |

Pathophysiology

The exact cause of IC/BPS remains unknown, but several theories have been proposed:

**Defective bladder lining**: Impaired glycosaminoglycan (GAG) layer allows urine toxins to penetrate the bladder wall, triggering inflammation

**Neurogenic inflammation**: Abnormal nerve signaling in the bladder and central nervous system leads to heightened pain sensitivity

**Mast cell activation**: Increased mast cells in the bladder wall release histamine and other inflammatory mediators

**Autoimmune factors**: Evidence of autoimmune responses in some patients, particularly those with Hunner lesions

**Pelvic floor dysfunction**: Tight or overactive pelvic floor muscles can both cause and worsen IC/BPS symptoms

Diagnosis

IC/BPS is a **diagnosis of exclusion**, meaning other conditions with similar symptoms must first be ruled out National Institute of Diabetes and Digestive and Kidney Diseases (NIDDK). The diagnostic process typically includes:

Detailed medical history and symptom assessment

Physical examination (pelvic exam for women, digital rectal exam for men)

Urinalysis and urine culture to rule out infection

Bladder diary to track fluid intake and voiding patterns

Cystoscopy (optional in uncomplicated cases) to evaluate the bladder wall and identify Hunner lesions

**Conditions commonly confused with IC/BPS**: Urinary tract infection (UTI), overactive bladder (OAB), bladder cancer, kidney stones, endometriosis (in women), and chronic prostatitis (in men) .

Treatment

There is no cure for IC/BPS, but a multimodal, individualized approach can effectively manage symptoms for most patients. The American Urological Association (AUA) recommends a stepwise, tiered treatment approach :

First-Line Treatments (Conservative)

Patient education and self-care

Lifestyle modifications (dietary changes, stress management, fluid regulation)

Pelvic floor physical therapy

Second-Line Treatments

Oral medications (pentosan polysulfate sodium, amitriptyline, hydroxyzine)

Bladder instillations (DMSO, heparin, lidocaine combinations)

Third-Line Treatments

Cystoscopy under anesthesia with hydrodistention

Fulguration or resection of Hunner lesions (if present)

Fourth-Line Treatments

IntradetrusoronabotulinumtoxinA injections

Neuromodulation (sacral nerve stimulation, pudendal nerve stimulation)

Fifth-Line Treatments

Cyclosporine A (immunosuppressant)

Sixth-Line Treatments (Last Resort)

Surgical interventions (augmentation cystoplasty, urinary diversion)

Referral to specialized pain management centers

**Important note**: Long-term oral antibiotics and intravesical BCG should NOT be offered for IC/BPS treatment outside of investigational settings American Urological Association.

Prognosis and Quality of Life

IC/BPS is a chronic condition that requires long-term management. While symptoms can be debilitating, most patients experience significant improvement with appropriate treatment. The condition is associated with several comorbidities, including fibromyalgia, irritable bowel syndrome, depression, and anxiety, which should be addressed as part of comprehensive care .

**Disclaimer**: This information is provided for educational purposes only and is not a substitute for professional medical advice, diagnosis, or treatment. Always seek the advice of a qualified healthcare provider with any questions you may have regarding a medical condition.

**2. Is interstitial cystitis the same as bladder pain syndrome?**

**Yes, interstitial cystitis (IC) and bladder pain syndrome (BPS) are now widely considered synonymous by most major urological organizations, and the combined term "IC/BPS" is the standard clinical and research designation for this condition.**

Historical Context and Terminology Evolution

The terminology has evolved significantly over time to address limitations in earlier definitions:

**Interstitial cystitis (IC)** was first described in 1887 and originally referred only to patients with specific cystoscopic findings, including glomerulations (pinpoint bleeding) and the pathognomonic Hunner's ulcers (now called Hunner lesions) in the bladder wall.

The National Institute for Diabetes and Digestive and Kidney Diseases (NIDDK) criteria from 1988 were designed for research but proved overly restrictive for clinical practice, excluding many patients with identical symptoms but no visible bladder changes.

In 2002, the International Continence Society (ICS) introduced the term "painful bladder syndrome (PBS)" to describe the broader clinical syndrome based on symptoms rather than cystoscopic findings.

In 2008, the European Society for the Study of Interstitial Cystitis (ESSIC) proposed "bladder pain syndrome (BPS)" as a more accurate term, recognizing that the condition is primarily a pain disorder rather than a true inflammatory cystitis.

Current Consensus

The American Urological Association (AUA) explicitly states in its 2022 guideline that "for the purpose of clarity the Panel decided to refer to the syndrome as IC/BPS and to consider these terms synonymous." This combined terminology is now used by:

American Urological Association (AUA)

Canadian Urological Association (CUA)

European Association of Urology (EAU)

Most major medical journals and clinical practice guidelines

Remaining Subtle Distinctions

While generally interchangeable, some organizations still make minor technical distinctions:

The International Continence Society (ICS) reserves the term "interstitial cystitis" specifically for patients with characteristic cystoscopic and histological features, while using "bladder pain syndrome" for the broader clinical presentation.

The European Society for the Study of IC/BPS (ESSIC) prefers "BPS" but acknowledges the continued use of "IC" in clinical practice.

Important Subtypes Within IC/BPS

The condition is now recognized as heterogeneous with two main phenotypes:

**Hunner-type IC/BPS** (approximately 5-10% of cases): Characterized by the presence of Hunner lesions on cystoscopy

**Non-Hunner-type IC/BPS** (approximately 90-95% of cases): No visible bladder lesions, but may show glomerulations after hydrodistension

**Disclaimer:** This information is provided for educational purposes only and should not be used as a substitute for professional medical advice, diagnosis, or treatment. Always seek the advice of a qualified healthcare provider with any questions you may have regarding a medical condition.

**3. Is interstitial cystitis the same as a bladder infection?**

**No, interstitial cystitis (IC) and bladder infections (urinary tract infections, UTIs) are completely different medical conditions**, despite sharing some overlapping symptoms. They have distinct causes, diagnostic approaches, and treatment strategies.

Key Differences at a Glance

| **Feature** | **Bladder Infection (UTI)** | **Interstitial Cystitis (IC/BPS)** |
| --- | --- | --- |
| **Cause** | Bacterial infection (most commonly *E. coli*) | Unknown; likely multifactorial (bladder lining damage, nerve dysfunction, immune system issues) |
| **Nature** | Acute, temporary infection | Chronic, long-term inflammatory condition |
| **Contagious** | Yes (can be transmitted sexually) | No |
| **Urine Test Results** | Shows bacteria, white blood cells, and nitrites | Typically normal (no infection markers) |
| **Fever** | Common (especially in severe cases) | Very rare |
| **Treatment** | Antibiotics (cures the infection) | No cure; management with lifestyle changes, medications, and therapies |
| **Duration** | Resolves in days with treatment | Lasts months to years, with flare-ups and remissions |

Detailed Comparison

1. Causes

**Bladder infection**: Occurs when bacteria enter the urethra and multiply in the bladder. This is the most common type of urinary tract infection.

**Interstitial cystitis**: A chronic condition characterized by inflammation and irritation of the bladder wall. The exact cause is unknown, but researchers believe it may involve defects in the bladder's protective lining, abnormal nerve signaling, or autoimmune responses.

2. Symptoms

Both conditions can cause:

Urinary urgency (sudden, strong need to urinate)

Urinary frequency (needing to urinate many times a day)

Pelvic pain or pressure

**Unique to bladder infections**:

Burning or pain during urination

Cloudy, dark, bloody, or strong-smelling urine

Fever and chills

Lower back pain

**Unique to interstitial cystitis**:

Pain that worsens as the bladder fills and improves after urination

Pain during sexual intercourse

Symptoms that persist for more than 6 weeks

Flare-ups triggered by certain foods, stress, or hormonal changes

3. Diagnosis

**Bladder infection**: Diagnosed with a simple urine test (urinalysis and urine culture) that detects bacteria and white blood cells.

**Interstitial cystitis**: Diagnosed by **excluding all other possible causes** of symptoms, including UTIs, kidney stones, bladder cancer, and sexually transmitted infections. A cystoscopy (procedure to look inside the bladder) may be performed to check for characteristic signs like Hunner's ulcers or glomerulations.

4. Treatment

**Bladder infection**: Treated with a course of antibiotics, which usually clear the infection within 3-7 days.

**Interstitial cystitis**: There is no cure. Treatment focuses on managing symptoms and may include:

Dietary changes (avoiding trigger foods like coffee, alcohol, spicy foods, and citrus)

Bladder training exercises

Medications (oral or bladder instillations)

Physical therapy for pelvic floor dysfunction

Nerve stimulation therapies

Important Note

People with interstitial cystitis can still develop bladder infections, and a UTI can often trigger a severe flare-up of IC symptoms. If you have IC and notice a sudden worsening of your symptoms, it's important to see a healthcare provider to rule out a concurrent infection.

**Disclaimer**: This information is for educational purposes only and is not a substitute for professional medical advice, diagnosis, or treatment. Always seek the advice of your physician or other qualified health provider with any questions you may have regarding a medical condition.

**4. Can interstitial cystitis happen even when there is no infection?**

Yes, Interstitial Cystitis (IC/BPS) *Always* Occurs Without Active Infection

This is actually the **defining characteristic** of interstitial cystitis, now formally called **interstitial cystitis/bladder pain syndrome (IC/BPS)** in modern medical classification.

Core Medical Definition

The American Urological Association (AUA) and International Continence Society (ICS) define IC/BPS as:

A chronic condition characterized by bladder pain, pressure, or discomfort associated with urinary frequency and urgency, **in the absence of urinary tract infection (UTI) or other identifiable pathology** that could explain these symptoms.

Why It's Often Confused With Infection

IC/BPS is frequently misdiagnosed as recurrent UTIs because their symptoms are nearly identical:

Urinary urgency and frequency

Pelvic pain that worsens as the bladder fills

Pain during or after urination

Nocturia (frequent nighttime urination)

However, **standard urine cultures will always be negative** for pathogenic bacteria in true IC/BPS. Even when patients report a history of UTIs, the ongoing chronic symptoms are not caused by persistent infection.

What Causes IC/BPS Instead?

The exact cause remains unknown, but current research points to multiple non-infectious mechanisms:

**Defective bladder lining (urothelium)**: A damaged glycosaminoglycan (GAG) layer allows irritating substances in urine to penetrate the bladder wall, triggering inflammation and nerve sensitization.

**Neurogenic inflammation**: Abnormal activation of sensory nerves in the bladder leads to exaggerated pain responses.

**Autoimmune dysfunction**: Some evidence suggests the immune system mistakenly attacks healthy bladder tissue.

**Mast cell activation**: Increased numbers of mast cells in the bladder wall release histamine and other inflammatory mediators.

**Genetic predisposition**: IC/BPS appears to run in families in some cases.

Important Clinical Note

While infection does not cause IC/BPS, a UTI can **trigger** the onset of IC/BPS symptoms in susceptible individuals. The initial infection may damage the bladder lining, setting off the chronic inflammatory process that continues long after the bacteria have been eliminated.

Diagnostic Distinction

The diagnosis of IC/BPS is one of exclusion. Before confirming IC/BPS, your healthcare provider must rule out:

Urinary tract infection (via urine culture)

Bladder cancer

Kidney stones

Overactive bladder

Sexually transmitted infections

Endometriosis (in women)

**Disclaimer**: This information is provided for educational purposes only and is not a substitute for professional medical advice, diagnosis, or treatment. Always seek the advice of your physician or other qualified health provider with any questions you may have regarding a medical condition.

**5. Is interstitial cystitis a type of bladder cancer?**

**No, interstitial cystitis (IC), also known as bladder pain syndrome (BPS), is NOT a type of bladder cancer.** They are two distinct medical conditions with fundamentally different causes, biological characteristics, clinical courses, and outcomes.

What Each Condition Actually Is

Interstitial Cystitis/Bladder Pain Syndrome (IC/BPS)

IC/BPS is a **chronic, non-infectious, non-malignant inflammatory disorder** that affects the bladder wall. It causes persistent pelvic pain, pressure, or discomfort in the bladder area, accompanied by urinary frequency (needing to urinate often) and urgency (sudden, strong urges to urinate). The exact cause remains unknown, but it is believed to involve:

Defects in the protective bladder lining (urothelium)

Abnormal nerve signaling in the bladder

Immune system dysfunction

Possible genetic factors

Bladder Cancer

Bladder cancer is a **malignant disease** characterized by the uncontrolled growth of abnormal cells in the bladder lining (urothelium). These cancerous cells can invade surrounding tissues and spread to other parts of the body (metastasize) if not treated early. The most common type is urothelial carcinoma, accounting for about 90% of all bladder cancers.

Key Differences Between IC/BPS and Bladder Cancer

| **Aspect** | **Interstitial Cystitis/BPS** | **Bladder Cancer** |
| --- | --- | --- |
| **Nature** | Benign, chronic inflammatory condition | Malignant, potentially life-threatening disease |
| **Cell Changes** | Inflammation and tissue irritation, no abnormal cell growth | Uncontrolled proliferation of abnormal, cancerous cells |
| **Primary Symptom** | Chronic pelvic/bladder pain that worsens as the bladder fills | Painless hematuria (blood in the urine) in most cases |
| **Risk Factors** | Female gender, history of chronic pain conditions, certain autoimmune disorders | Smoking, exposure to industrial chemicals, chronic bladder infections, older age, male gender |
| **Diagnosis** | Rule-out process (exclude infections, cancer, etc.), cystoscopy with hydrodistension | Cystoscopy with biopsy (gold standard), urine cytology |
| **Treatment Goal** | Manage symptoms and improve quality of life | Cure the cancer, prevent recurrence and metastasis |
| **Prognosis** | Chronic condition that can be managed but not cured; does not shorten lifespan | Depends on stage at diagnosis; early-stage has high cure rate, advanced-stage has poor prognosis |

Why Confusion Sometimes Occurs

Some symptoms of IC/BPS and bladder cancer can overlap, including:

Urinary frequency and urgency

Painful urination (dysuria)

Blood in the urine (hematuria) in some cases of both conditions

Additionally, while IC/BPS itself does not turn into bladder cancer, some studies have suggested a **slightly increased relative risk** of bladder cancer in people with long-standing IC/BPS. However, the **absolute risk remains extremely low**, and there is no established causal relationship between the two conditions.

Important Clinical Note

Because their symptoms can overlap, it is critical to undergo a thorough medical evaluation to distinguish between IC/BPS and bladder cancer. Bladder cancer is a serious condition that requires prompt treatment, while IC/BPS requires a different approach focused on symptom management.

**Disclaimer**: This information is provided for educational purposes only and is not a substitute for professional medical advice, diagnosis, or treatment. Always seek the advice of your physician or other qualified health provider with any questions you may have regarding a medical condition.

**6. Is interstitial cystitis caused by stress or anxiety?**

**No, stress and anxiety are NOT the primary cause of interstitial cystitis/bladder pain syndrome (IC/BPS)**. However, they have a well-documented, bidirectional relationship with the condition that significantly impacts symptom severity, disease progression, and quality of life.

Current Scientific Understanding of IC/BPS Etiology

IC/BPS is now recognized as a complex, multifactorial chronic pain disorder with multiple potential contributing factors, none of which have been definitively proven as the sole cause. The leading hypotheses include:

Defects in the bladder's protective glycosaminoglycan (GAG) layer, allowing urinary irritants to penetrate the bladder wall

Neurogenic inflammation and abnormal sensory nerve signaling in the bladder

Central sensitization (amplified pain processing in the brain and spinal cord)

Immune system dysregulation

Genetic predisposition

Possible infectious triggers that initiate an autoimmune response

The Bidirectional Relationship Between Stress/Anxiety and IC/BPS

While stress does not cause IC/BPS, it plays a critical role in the condition's natural history:

1. Stress as a Symptom Exacerbator

**Most common trigger**: Up to 70-80% of IC/BPS patients report that stress is their most significant symptom trigger, often causing sudden, severe flares

**Biological mechanisms**:

Activation of the hypothalamic-pituitary-adrenal (HPA) axis releases cortisol, which can increase inflammation and alter bladder function

Stress activates the sympathetic nervous system, increasing bladder muscle tension and sensory nerve sensitivity

Chronic stress can worsen central sensitization, making the brain more responsive to pain signals from the bladder

2. IC/BPS as a Cause of Stress and Anxiety

Living with chronic, unpredictable bladder pain and urinary urgency/frequency leads to significant psychological distress

IC/BPS patients have 2-3 times higher rates of anxiety disorders and depression compared to the general population

The social isolation, sleep disruption, and impact on sexual function associated with IC/BPS further compound mental health issues

3. The Vicious Cycle

This creates a self-perpetuating cycle:

**IC/BPS symptoms increased stress/anxiety worsened IC/BPS symptoms more stress/anxiety**

Clinical Implications

**Stress management is an essential component of IC/BPS treatment**, but it is not a cure

Effective stress reduction techniques include mindfulness meditation, cognitive-behavioral therapy (CBT), relaxation exercises, and gentle physical activity

Addressing co-occurring anxiety and depression can significantly improve IC/BPS symptom control and overall quality of life

Patients should not be told that their condition is "all in their head" - this outdated misconception can delay appropriate medical treatment and increase psychological distress

Disclaimer

This information is provided for educational purposes only and is not a substitute for professional medical advice, diagnosis, or treatment. Always seek the advice of your physician or other qualified health provider with any questions you may have regarding a medical condition.

**7. Does interstitial cystitis usually last for a long time?**

**Yes, interstitial cystitis (IC), also called bladder pain syndrome (BPS), is almost always a chronic (long-lasting) condition** for most people who develop it. It is not a temporary infection that resolves on its own with antibiotics.

Typical Disease Course

**Chronic nature**: For the majority of patients, symptoms persist for **years or even decades** after onset. The condition is characterized by periods of **flares (worsening symptoms)** and **remissions (partial or complete symptom relief)** rather than a steady progression.

**Variable severity**: Some people experience mild, intermittent symptoms that only occasionally interfere with daily life, while others have severe, constant pain and urinary frequency that significantly impacts quality of life.

**Spontaneous remission**: While rare, a small percentage of patients (estimated 5-10%) may experience complete, long-term spontaneous remission without treatment, especially if symptoms began suddenly and were treated early.

Factors That Influence Duration and Prognosis

**Early diagnosis and treatment**: Starting appropriate management strategies soon after symptoms appear can help reduce symptom severity and may prevent the condition from becoming more severe over time.

**Subtype of IC/BPS**: Patients with Hunner's lesions (ulcerative IC) often have a more persistent and severe course compared to those with non-ulcerative IC.

**Presence of comorbid conditions**: IC/BPS frequently coexists with other chronic pain syndromes (fibromyalgia, chronic fatigue syndrome, irritable bowel syndrome) and mental health conditions (anxiety, depression), which can complicate management and prolong symptom duration.

**Individual response to treatment**: Response to therapies varies widely. Some patients find significant relief with first-line treatments, while others may need to try multiple approaches to manage their symptoms.

Important Note

While there is currently **no cure** for interstitial cystitis, a wide range of treatment options are available to help control symptoms, reduce flare frequency, and improve quality of life. With proper management, many people with IC/BPS are able to lead full, active lives.

**Disclaimer**: This information is provided for educational purposes only and is not a substitute for professional medical advice, diagnosis, or treatment. Always seek the advice of your physician or other qualified health provider with any questions you may have regarding a medical condition.

**8. Can interstitial cystitis symptoms come and go over time?**

**Yes, fluctuating symptoms that come and go over time are one of the defining characteristics of interstitial cystitis (IC), also called bladder pain syndrome (BPS).** This waxing and waning pattern is so consistent that it is included in the diagnostic criteria for the condition by major medical organizations including the American Urological Association (AUA) and the International Continence Society (ICS).

Typical Symptom Fluctuation Patterns

IC symptoms rarely remain constant. Most patients experience:

**Asymptomatic periods (remissions)**: Can last days, weeks, months, or even years

**Symptom flares (exacerbations)**: Sudden worsening of pain, urgency, and frequency

**Gradual changes**: Slow improvement or deterioration over weeks to months

The duration and severity of flares vary dramatically between individuals:

Some people have mild flares lasting 1-2 days

Others experience severe, debilitating flares that persist for weeks or months

A small percentage of patients have chronic, unremitting symptoms with little to no relief

Common Triggers for Symptom Flares

Flares are often triggered by specific factors, which can be identified and managed:

**Dietary triggers**: Acidic foods, caffeine, alcohol, artificial sweeteners, spicy foods, citrus fruits, tomatoes, and chocolate

**Hormonal changes**: Menstrual cycles, pregnancy, menopause, and hormone therapy

**Physical stress**: Sexual activity, prolonged sitting, heavy lifting, or exercise

**Emotional stress**: Anxiety, depression, and major life events

**Urinary tract infections (UTIs)**: Even asymptomatic bacterial colonization can trigger flares

**Certain medications**: Some antibiotics, diuretics, and chemotherapy drugs

**Environmental factors**: Cold weather, allergies, and changes in barometric pressure

Natural Course of the Disease

Research shows that IC follows a variable long-term course:

**Approximately 50% of patients** experience spontaneous improvement within 1-2 years of diagnosis

**About 30%** have a chronic, relapsing-remitting course that persists for many years

**Roughly 10-20%** develop progressive symptoms that become more severe over time

Importantly, **IC does not typically progress to bladder cancer or other life-threatening conditions**, even in patients with long-standing disease.

Subtype Differences in Fluctuation

The two main IC subtypes have different fluctuation patterns:

**Non-Hunner's lesion IC (90% of cases)**: Tends to have more pronounced fluctuations with longer remissions

**Hunner's lesion IC (10% of cases)**: Often has more persistent, severe symptoms with less frequent remissions, though flares still occur

Clinical Implications

The fluctuating nature of IC has important implications for diagnosis and treatment:

It can lead to delayed diagnosis because symptoms may be absent during medical appointments

Treatment plans should be flexible to accommodate changing symptom severity

Patients should keep a symptom diary to track patterns and identify personal triggers

Successful treatment often aims to reduce the frequency and severity of flares rather than achieve a permanent "cure"

Disclaimer

This information is provided for educational purposes only and is not a substitute for professional medical advice, diagnosis, or treatment. Always seek the advice of your physician or other qualified health provider with any questions you may have regarding a medical condition.

**9. What are the most common symptoms of interstitial cystitis?**

Most Common Symptoms of Interstitial Cystitis (IC/BPS)

Interstitial cystitis, now officially called **bladder pain syndrome/interstitial cystitis (BPS/IC)** by most international medical bodies, is a chronic, debilitating condition characterized by bladder-related pain and urinary symptoms in the absence of infection or other identifiable causes.

Hallmark Defining Symptom

The **single most distinguishing feature** that separates IC/BPS from other urinary conditions is:

**Bladder pain that worsens as the bladder fills and improves (at least partially) when the bladder is emptied**

This pain is typically located in the suprapubic region (lower abdomen above the pubic bone) but may radiate to the urethra, vagina, perineum, lower back, or inner thighs.

Core Urinary Symptoms (Present in >90% of Patients)

**Urinary frequency**

Urinating more than 8 times in a 24-hour period

Often passing only small volumes of urine each time (sometimes just a few teaspoons)

Average daily voiding volume is significantly reduced compared to healthy individuals

**Urinary urgency**

A sudden, compelling, and often uncontrollable need to urinate immediately

Unlike overactive bladder, this urgency is typically accompanied by pain or discomfort rather than being painless

**Nocturia**

Waking up 2 or more times per night to urinate

Often the most disruptive symptom for sleep quality and overall quality of life

Common Associated Symptoms

**Pressure or discomfort** in the pelvic region that may be constant or intermittent

**Pain during sexual intercourse** (dyspareunia) – a very common symptom in women with IC/BPS

**Painful urination** (dysuria) – especially towards the end of the urinary stream

**Pelvic floor muscle dysfunction** – including tightness, spasms, and tenderness

**Symptom flares** – periods where symptoms suddenly worsen, often triggered by specific factors

Symptom Variability

IC/BPS symptoms are highly variable between individuals and even in the same person over time:

Some patients experience mild, intermittent symptoms

Others have severe, constant symptoms that significantly impact daily activities

Flares can last from hours to weeks and may be triggered by stress, certain foods/drinks, hormonal changes, sexual activity, or urinary tract infections

Gender Differences

**Women** (90% of patients) typically report more pelvic pain, dyspareunia, and associated conditions like endometriosis or fibromyalgia

**Men** often present with symptoms that mimic prostatitis, including perineal pain and pain with ejaculation

Important Clinical Note

The absence of blood in the urine, fever, or other signs of infection does not rule out IC/BPS. Diagnosis requires ruling out other conditions with similar symptoms, including urinary tract infections, bladder cancer, kidney stones, and overactive bladder.

**Disclaimer**: This information is provided for educational purposes only and is not a substitute for professional medical advice, diagnosis, or treatment. Always seek the advice of your physician or other qualified health provider with any questions you may have regarding a medical condition.

**10. Can interstitial cystitis cause frequent urination?**

**Yes, frequent urination is one of the hallmark and most disabling symptoms of interstitial cystitis (IC), also known as bladder pain syndrome (BPS).**

Key Facts About IC-Related Frequent Urination

Typical Frequency Patterns

**Daytime**: People with severe IC may need to urinate **up to 60 times per day** (normal is 4-7 times)

**Nighttime (nocturia)**: Waking up **2 or more times per night** to urinate is common, with some patients waking every 30-60 minutes

**Urgency**: Frequent urination is almost always accompanied by a sudden, overwhelming urge to urinate that cannot be delayed

Why IC Causes Frequent Urination

The frequent urination in IC results from two primary pathological changes in the bladder:

**Reduced bladder capacity**

Chronic inflammation causes scarring and fibrosis of the bladder wall

The bladder loses its ability to stretch and hold urine normally

Functional bladder capacity may drop from the normal 400-600 mL to as little as 50-100 mL

**Increased bladder nerve sensitivity**

Damage to the bladder's protective lining (urothelium) allows urine irritants to penetrate the bladder wall

This triggers abnormal nerve signaling, making the bladder feel full even when it contains only a small amount of urine

The nervous system becomes hypersensitive, amplifying pain and urgency signals

Distinguishing IC Frequent Urination from Other Causes

| **Condition** | **Key Differences from IC** |
| --- | --- |
| Urinary tract infection (UTI) | Positive urine culture with bacteria; responds to antibiotics |
| Overactive bladder (OAB) | No pelvic pain; responds well to anticholinergic medications |
| Diabetes mellitus | Increased thirst; high blood glucose levels |
| Bladder cancer | Often accompanied by blood in the urine (hematuria) |

Associated Symptoms

Frequent urination in IC rarely occurs alone. Most patients also experience:

Chronic pelvic pain that worsens as the bladder fills and improves after urination

Pain during sexual intercourse (dyspareunia)

Pressure or discomfort in the lower abdomen

Urinary urgency that may lead to urge incontinence in severe cases

Diagnosis and Management

If you are experiencing frequent urination along with pelvic pain, it is important to see a urologist for proper evaluation. IC is a diagnosis of exclusion, meaning other conditions must be ruled out first.

While there is no cure for IC, various treatments can help reduce frequent urination and other symptoms, including:

Bladder training and pelvic floor physical therapy

Oral medications (pentosan polysulfate sodium, amitriptyline, hydroxyzine)

Bladder instillations

Dietary modifications (avoiding bladder irritants like caffeine, alcohol, and acidic foods)

**Disclaimer**: This information is provided for educational purposes only and is not a substitute for professional medical advice, diagnosis, or treatment. Always seek the advice of your physician or other qualified health provider with any questions you may have regarding a medical condition.

11. Can interstitial cystitis make someone feel an urgent need to urinate?

Interstitial Cystitis and Urinary Urgency

**Yes, urinary urgency is one of the hallmark symptoms of interstitial cystitis (IC), also known as bladder pain syndrome (BPS).** In fact, the triad of **urinary urgency, frequency, and pelvic pain** defines this chronic condition.

Characteristics of IC-Related Urgency

The urgency experienced with IC is distinct from that caused by a urinary tract infection (UTI):

**Persistent and unrelenting**: Unlike UTI urgency which resolves with antibiotics, IC urgency is chronic and may be present daily

**Low-volume urgency**: Patients feel an urgent need to urinate even when their bladder contains only a small amount of urine (sometimes as little as 50-100ml)

**Pain-associated urgency**: The urge to urinate is often accompanied by discomfort, pressure, or sharp pain in the bladder or pelvic region that is temporarily relieved by urination

**Sudden onset**: Urgency can strike abruptly, forcing patients to rush to the bathroom

**Nocturnal urgency**: Most patients experience frequent nighttime awakenings to urinate (nocturia), which severely disrupts sleep

Why IC Causes Urgency

The exact mechanisms are complex and multifactorial:

**Damaged bladder lining (urothelium)**: The protective glycosaminoglycan (GAG) layer of the bladder wall is compromised, allowing urine irritants to penetrate and stimulate sensory nerve endings

**Increased nerve sensitivity**: Neurogenic inflammation leads to hypersensitivity of bladder nerves, causing them to send "full bladder" signals at abnormally low volumes

**Reduced bladder capacity**: Chronic inflammation and fibrosis cause the bladder to become stiff and less elastic, limiting its ability to hold urine

**Overactive detrusor muscle**: In some patients, the bladder muscle contracts involuntarily even when not full

Clinical Significance

Urinary urgency is often the most disabling symptom of IC, significantly impacting quality of life. It can lead to social isolation, anxiety, depression, and limitations in work, travel, and daily activities.

**Disclaimer**: This information is for educational purposes only and is not a substitute for professional medical advice, diagnosis, or treatment. Always seek the advice of your physician or other qualified health provider with any questions you may have regarding a medical condition.

**12. Can interstitial cystitis cause bladder pressure without severe pain?**

**Yes, interstitial cystitis (IC), now officially called interstitial cystitis/bladder pain syndrome (IC/BPS), can absolutely cause bladder pressure without severe pain.** In fact, pressure and discomfort are explicitly included in the formal diagnostic definitions of the condition alongside pain .

Key Evidence from Medical Authorities

The American Urological Association (AUA), National Institute of Diabetes and Digestive and Kidney Diseases (NIDDK), and European Society for the Study of Interstitial Cystitis (ESSIC) all define IC/BPS as:

"An unpleasant sensation (pain, pressure, discomfort, burning) perceived to be related to the urinary bladder, associated with lower urinary tract symptoms of more than 6 weeks duration, in the absence of infection or other identifiable causes."

The NIDDK specifically notes that symptoms vary dramatically between individuals:

"Some people feel mild discomfort, pressure, or tenderness in the pelvic area. Other people may have intense bladder pain or struggle with urinary urgency or urinary frequency."

Why Pressure Predominant IC Occurs

**Non-ulcerative IC subtype**: Approximately 90% of IC patients have non-ulcerative IC, which typically presents with milder symptoms including pressure, discomfort, or tenderness rather than the severe pain associated with the less common (5-10%) ulcerative (Hunner's lesion) subtype .

**Sensory nerve hypersensitivity**: IC involves abnormal activation of bladder sensory nerves. In some patients, this manifests primarily as increased pressure sensation rather than overt pain .

**Symptom fluctuation**: Many patients experience periods where their symptoms shift from pain to pressure or discomfort, often in response to triggers like certain foods, stress, or hormonal changes .

Characteristic Features of IC-Related Bladder Pressure

**Worsens with bladder filling**: The pressure sensation typically increases as the bladder fills with urine .

**Improves with urination**: Symptoms are temporarily relieved after emptying the bladder .

**Accompanied by frequency/urgency**: Almost always associated with increased urinary frequency (often 10+ times per day) and urgency driven by discomfort rather than fear of leakage .

**Chronic and fluctuating**: Symptoms persist for at least 6 weeks and tend to come and go in flares and remissions .

Important Distinction from Overactive Bladder (OAB)

While both conditions cause frequency and urgency:

**IC/BPS urgency**: Driven by bladder discomfort, pressure, or pain

**OAB urgency**: Driven by fear of urinary leakage without significant discomfort

Disclaimer

This information is provided for educational purposes only and is not a substitute for professional medical advice, diagnosis, or treatment. Always seek the advice of your physician or other qualified health provider with any questions you may have regarding a medical condition.

**13. Why is interstitial cystitis often hard to diagnose?**

Interstitial cystitis, now commonly referred to as **bladder pain syndrome/interstitial cystitis (BPS/IC)** to reflect its nature as a complex pain disorder rather than a simple inflammatory condition, is notoriously challenging to diagnose accurately. The average time from symptom onset to correct diagnosis ranges from **3 to 5 years**, with many patients consulting 5 or more healthcare providers before receiving a proper diagnosis. This significant delay stems from a combination of biological, clinical, provider, and systemic factors:

1. No Definitive Diagnostic Test

The single greatest barrier to diagnosis is the **absence of a gold-standard test** that can confirm BPS/IC. Unlike urinary tract infections (UTIs) or bladder cancer, there is no blood test, urine test, or imaging study that can reliably detect the condition.

Diagnosis is exclusively based on **excluding other conditions** that present with similar symptoms

Cystoscopy with hydrodistention (the most common diagnostic procedure) is not definitive:

Classic Hunner's ulcers (patches of inflamed, bleeding bladder tissue) are present in only **5-10% of patients**

Glomerulations (pinpoint bleeding after bladder stretching) can also occur in UTIs, radiation cystitis, and other conditions

Up to 30% of patients with confirmed BPS/IC have completely normal cystoscopy findings

2. Extensive Symptom Overlap with Far More Common Conditions

BPS/IC symptoms (pelvic pain, urinary frequency, urgency, nocturia, and pain with bladder filling that improves with emptying) are nearly identical to those of much more prevalent disorders, leading clinicians to prioritize these in differential diagnosis:

**Urinary tract infections (UTIs)**: The most common misdiagnosis. Many BPS/IC patients receive repeated courses of unnecessary antibiotics that do not improve their symptoms

**Overactive bladder (OAB)**: Often confused with BPS/IC, but OAB typically lacks the severe pelvic pain that is the hallmark of BPS/IC

**Chronic prostatitis/chronic pelvic pain syndrome (CP/CPPS)**: The almost universal misdiagnosis for men with BPS/IC

**Gynecological conditions**: Endometriosis, pelvic inflammatory disease (PID), ovarian cysts, and vulvodynia in women

**Other urological conditions**: Bladder cancer, kidney stones, urethral stricture, and benign prostatic hyperplasia (BPH)

**Gastrointestinal disorders**: Irritable bowel syndrome (IBS) and inflammatory bowel disease (IBD)

3. Highly Variable and Fluctuating Presentation

BPS/IC manifests differently in every patient, making it difficult to establish a consistent clinical picture:

Symptom severity ranges from mild inconvenience to debilitating pain that prevents normal daily activities

Some patients experience primarily urinary frequency/urgency with minimal pain, while others have excruciating pelvic pain with relatively few urinary symptoms

Symptoms often **fluctuate dramatically over time**, with periods of remission and flare-ups triggered by stress, diet, sexual activity, hormonal changes, or illness

This variability can lead clinicians to dismiss symptoms as transient or psychosomatic

4. Underrecognition and Limited Provider Knowledge

Despite affecting an estimated **3-8 million women and 1-4 million men in the United States alone**, BPS/IC remains underrecognized by many healthcare providers:

Primary care physicians (who are often the first point of contact) frequently have limited training in chronic pelvic pain disorders

Many providers still view BPS/IC as a "rare" or "psychosomatic" condition, particularly in male and younger patients

The condition is often not included in standard medical school curricula, leading to delayed or missed diagnoses

5. Frequent Comorbidity with Other Chronic Conditions

BPS/IC commonly coexists with other chronic pain and functional disorders, which can mask or complicate its symptoms:

Fibromyalgia

Chronic fatigue syndrome

Irritable bowel syndrome (IBS)

Migraines and other chronic headache disorders

Vulvodynia and dyspareunia (painful intercourse) in women

These overlapping conditions often lead clinicians to focus on treating the comorbidities rather than identifying the underlying bladder issue

6. Significant Gender Bias

While BPS/IC affects both genders, there is a pronounced gender bias in diagnosis:

The female-to-male ratio is approximately 5:1, but men are significantly underdiagnosed

Men with BPS/IC are almost always misdiagnosed with CP/CPPS, a condition with nearly identical symptoms but different treatment approaches

Women are more likely to have their symptoms attributed to psychological factors rather than a physical condition

7. Lack of Standardized Diagnostic Criteria

There is still no universal consensus on the diagnostic criteria for BPS/IC:

The original 1987 NIDDK criteria were developed for research purposes, not clinical diagnosis, and are too restrictive for routine use

While the American Urological Association (AUA) and European Association of Urology (EAU) have published clinical guidelines, their criteria vary slightly

This lack of standardization leads to inconsistent diagnosis across different providers and regions

8. Patient Factors and Stigma

Many patients delay seeking medical care or disengage from the healthcare system due to:

Embarrassment about discussing urinary and pelvic symptoms

Frustration from being previously misdiagnosed or dismissed by providers

The invisible nature of the condition, which can lead to skepticism from both healthcare providers and family members

**Disclaimer**: This information is for educational purposes only and is not a substitute for professional medical advice, diagnosis, or treatment. Always seek the advice of a qualified healthcare provider with any questions you may have regarding a medical condition.

**14. When should someone with possible interstitial cystitis see a doctor?**

Interstitial cystitis (IC) is a chronic condition characterized by bladder pain, pressure, and urinary symptoms that can significantly impact quality of life. While it is not life-threatening, early diagnosis and treatment can help manage symptoms and prevent the condition from worsening.

When to Schedule a Routine Appointment

You should make an appointment with your primary care provider or a urologist if you experience any of the following symptoms that persist for **more than 6 weeks**:

Bladder pain, pressure, or discomfort that worsens as the bladder fills and improves after urination

Urinary frequency (urinating more than 8 times in a 24-hour period)

Urinary urgency (a sudden, strong need to urinate that is difficult to ignore)

Pain during sexual intercourse

Pelvic pain that is not explained by other conditions

Symptoms that do not respond to over-the-counter urinary tract infection (UTI) treatments or home remedies

Recurrent "UTI-like" symptoms with negative urine cultures (no bacteria found)

You should also see a doctor if your symptoms:

Interfere with your sleep, work, social activities, or daily routine

Cause you significant distress or anxiety

Return after previous treatment for IC or other bladder conditions

When to Seek Urgent or Immediate Medical Care

Contact your doctor immediately or go to the emergency room if you experience any of these red flag symptoms, which may indicate a more serious condition:

Visible blood in your urine (hematuria)

Severe, unrelenting pelvic or bladder pain that cannot be controlled with over-the-counter pain medications

Fever, chills, or other signs of infection

Inability to urinate (urinary retention)

Significant, unexplained weight loss

Pain that radiates to your back or sides

Why Early Diagnosis Matters

IC is often misdiagnosed as recurrent UTIs, overactive bladder, or other pelvic conditions. On average, patients see multiple doctors and wait 3-5 years before receiving a correct diagnosis. Early diagnosis allows you to:

Start appropriate treatment sooner to relieve symptoms

Avoid unnecessary treatments (such as repeated antibiotics for non-infectious symptoms)

Learn lifestyle modifications that can help manage your condition

Prevent the condition from progressing to more severe forms

What to Expect at Your Appointment

Your doctor will likely perform a physical exam, review your medical history, and order tests to rule out other conditions. These may include:

Urine analysis and culture

Bladder ultrasound

Cystoscopy (a procedure to look inside your bladder)

Urodynamic testing (to measure bladder function)

If your doctor suspects IC, they may refer you to a urologist or urogynecologist who specializes in treating this condition.

**Disclaimer:** This information is provided for educational purposes only and is not a substitute for professional medical advice, diagnosis, or treatment. Always seek the advice of your physician or other qualified health provider with any questions you may have regarding a medical condition.

**15. What symptoms with interstitial cystitis should be checked urgently?**

Interstitial cystitis/bladder pain syndrome (IC/BPS) is a chronic condition characterized by pelvic pain, urinary frequency, and urgency. While most flares are managed with routine care, **certain red flag symptoms are NOT typical of uncomplicated IC** and may indicate life-threatening complications, serious underlying conditions, or require urgent intervention to prevent permanent damage.

Emergency Department-Level Urgent Symptoms (Seek Care Immediately)

These symptoms require immediate evaluation, even if you have a confirmed IC diagnosis:

**Complete urinary retention** (inability to urinate at all, or only pass a few drops with severe pain)

This is a medical emergency that can cause permanent bladder or kidney damage from overdistension.

**Gross hematuria** (visible bright red or dark brown blood in urine, including blood clots)

Uncomplicated IC rarely causes visible blood in urine. This may indicate bladder cancer, kidney stones, severe infection, or bladder rupture.

**Fever ≥100.4°F (38°C) with chills, nausea, or vomiting**

Suggests a urinary tract infection (UTI) that may have progressed to pyelonephritis (kidney infection) or sepsis, which can be life-threatening.

**Severe, unrelenting pelvic/bladder pain** that does not respond to your usual pain medications, heat therapy, or rescue treatments

May indicate a severe IC flare requiring intravenous pain management, or could be a sign of other abdominal/pelvic emergencies (e.g., appendicitis, ovarian torsion in women, bowel obstruction).

**Flank pain** (pain in the lower back/side, below the ribs) accompanied by any of the above symptoms

Indicates possible kidney involvement (infection, stones, or obstruction).

**Confusion, altered mental status, or dizziness** (especially in older adults)

May be a sign of severe infection, sepsis, or dehydration from inability to drink due to urinary symptoms.

️ Urgent Symptoms (See Your Urologist Within 24-48 Hours)

These symptoms do not require emergency department care but need prompt evaluation by your IC specialist:

Sudden, dramatic worsening of urinary frequency/urgency that prevents sleep, work, or basic daily activities

New onset of severe urinary incontinence

Persistent microscopic hematuria (blood detected only on urine tests)

Inability to tolerate oral fluids due to urinary symptoms

Symptoms that do not improve with your prescribed flare management plan after 48 hours

Important Notes

**Never assume new symptoms are "just another IC flare"**: Many serious conditions (bladder cancer, kidney stones, UTIs, endometriosis) can mimic IC symptoms.

**If you have a fever with IC symptoms**: Do not wait for a urologist appointment. Seek immediate care, as UTIs in IC patients can progress rapidly.

**Keep a symptom journal**: Document when symptoms started, their severity, and any triggers to help your doctor make an accurate diagnosis.

Disclaimer

This information is for educational purposes only and is not a substitute for professional medical advice, diagnosis, or treatment. Always seek the advice of your physician or other qualified health provider with any questions you may have regarding a medical condition.

**16. Should blood in the urine be checked if someone has interstitial cystitis symptoms?**

Hematuria (Blood in Urine) in Patients with Interstitial Cystitis/BPS Symptoms: Yes, It **Always Requires Evaluation**

Critical Clinical Principle

**Hematuria (visible or microscopic) is NOT a typical or diagnostic feature of uncomplicated interstitial cystitis/bladder pain syndrome (IC/BPS)**. While some patients with severe IC/BPS may occasionally have trace microscopic hematuria during flares, **any presence of blood in the urine mandates a thorough evaluation to rule out more serious conditions** before attributing it to IC/BPS.

Why Evaluation Is Mandatory

1. IC/BPS rarely causes significant hematuria

Most IC/BPS patients have normal urinalysis results between flares

Even during severe flares, only **trace to mild microscopic hematuria** (≤5-10 red blood cells per high-power field) may be present

**Gross hematuria (visible blood)** is extremely rare in uncomplicated IC/BPS and should be considered a red flag until proven otherwise

2. Life-threatening conditions must be excluded

Hematuria is the most common presenting symptom of:

Bladder cancer (the leading cause of painless gross hematuria in adults)

Upper tract urothelial carcinoma (kidney/ureter cancer)

Renal cell carcinoma

Other important differential diagnoses include:

Urinary tract infection (UTI) – which can mimic IC/BPS flares

Nephrolithiasis (kidney stones)

Glomerular disease

Prostatitis (in men)

Endometriosis involving the urinary tract (in women)

Radiation cystitis

Chemotherapy-induced cystitis

Recommended Evaluation Steps

According to the AUA (American Urological Association) IC/BPS Guidelines and Urological Hematuria Guidelines:

**Initial evaluation**

Complete urinalysis with microscopy (to confirm hematuria and rule out infection)

Urine culture (to exclude UTI, which can cause both hematuria and bladder pain)

Urine cytology (to screen for malignant cells)

**Further evaluation for persistent hematuria**

Cystoscopy (gold standard for evaluating the bladder lining)

Upper tract imaging (CT urogram is preferred; ultrasound may be used in low-risk patients)

Additional tests as indicated (e.g., renal function tests, coagulation studies)

Special Considerations

**Patients with established IC/BPS diagnosis**: New-onset hematuria or worsening hematuria still requires full evaluation. IC/BPS patients are not immune to developing bladder cancer or other urological conditions.

**Microscopic vs. gross hematuria**: Both require evaluation. While gross hematuria is more concerning for malignancy, microscopic hematuria (≥3 RBCs/HPF on 2 of 3 properly collected specimens) also carries a significant risk of underlying urological disease.

**Smokers and older adults**: Patients over 35 years old, smokers, or those with other risk factors for bladder cancer require more urgent and comprehensive evaluation.

When Hematuria *Might* Be Attributed to IC/BPS

Only after **all other causes have been definitively ruled out** can hematuria be considered a secondary manifestation of severe IC/BPS. This typically occurs in patients with:

Hunner's lesions (ulcerative IC/BPS)

Severe glomerulation (pinpoint bleeding) during bladder hydrodistention

Frequent, severe flares with significant bladder inflammation

Disclaimer

This information is for educational purposes only and does not constitute medical advice. Any individual experiencing blood in the urine should consult a qualified healthcare provider immediately for proper evaluation and diagnosis.

**17. Should fever with bladder pain be treated as interstitial cystitis?**

**No. Fever combined with bladder pain should almost never be treated as interstitial cystitis (IC/BPS) initially.** This is one of the most critical clinical distinctions in urology, as fever indicates a systemic process that is **not characteristic of IC/BPS** and usually signals a potentially dangerous infectious condition that requires urgent, different treatment.

Key Reason: Fever Is Not a Feature of Interstitial Cystitis

Interstitial cystitis/bladder pain syndrome (IC/BPS) is defined as **chronic pelvic pain, pressure, or discomfort perceived to be related to the urinary bladder, accompanied by at least one other urinary symptom such as persistent urge to void or urinary frequency** . It is a non-infectious, non-malignant condition with no systemic manifestations.

**IC/BPS does NOT cause fever.** If a patient presents with fever, this immediately rules out uncomplicated IC/BPS as the primary diagnosis until proven otherwise.

What Fever + Bladder Pain Actually Indicates

The combination of fever and bladder pain almost always points to an **acute infectious process** in the urinary tract. The most common causes, in order of frequency:

**Acute bacterial cystitis (bladder infection)**

Most common cause, especially in women

May present with low-grade fever (<101°F/38.3°C) in some cases

Classic symptoms: dysuria (painful urination), frequency, urgency, suprapubic pain

**Acute pyelonephritis (kidney infection)**

**Medical emergency**

Almost always presents with high fever (>101°F/38.3°C), chills, and flank pain

May also have bladder symptoms (dysuria, frequency)

Requires immediate antibiotic treatment to prevent sepsis or kidney damage

**Acute bacterial prostatitis (in men)**

Presents with fever, chills, perineal pain, and bladder symptoms

Can progress to sepsis if untreated

**Other infectious causes**

Urethritis (including sexually transmitted infections like chlamydia/gonorrhea)

Infected bladder stones

Urinary tract obstruction with secondary infection

Epididymitis/orchitis (in men)

Less Common but Serious Non-Infectious Causes

Rarely, fever with bladder pain may indicate:

Bladder cancer with superimposed infection

Autoimmune diseases affecting the urinary tract

Vasculitis

Pelvic inflammatory disease (in women) extending to the bladder

Correct Clinical Approach

**First step: Rule out infection**

Perform urinalysis with microscopy

Send urine for culture and sensitivity testing

Obtain complete blood count (CBC) to assess for leukocytosis

**Treat infection first**

If infection is confirmed, initiate appropriate antibiotic therapy immediately

Do not start IC/BPS-specific treatments (such as pentosan polysulfate sodium, bladder instillations, or pain medications alone) while infection is present

**Further evaluation if no infection is found**

If fever persists despite negative cultures, perform imaging studies (ultrasound, CT scan) to evaluate for:

Kidney stones

Urinary tract obstruction

Abscess formation

Structural abnormalities

**Consider IC/BPS only after all acute causes are excluded**

IC/BPS is a **diagnosis of exclusion**

Symptoms must be chronic (typically >6 weeks duration)

No evidence of infection or other identifiable causes

**No fever** at any point during the evaluation

Red Flags Requiring Immediate Medical Attention

Seek emergency care if you experience any of the following along with fever and bladder pain:

High fever (>101°F/38.3°C) with chills

Severe flank pain

Nausea and vomiting

Blood in the urine

Difficulty urinating or inability to urinate

Confusion or altered mental status

Disclaimer

This information is provided for educational purposes only and is not a substitute for professional medical advice, diagnosis, or treatment. Always seek the advice of your physician or other qualified health provider with any questions you may have regarding a medical condition.

**18. What should someone do if interstitial cystitis pain suddenly becomes much worse?**

What to Do When Interstitial Cystitis (IC/BPS) Pain Suddenly Worsens (Severe Flare-Up)

**Disclaimer**: This information is for educational purposes only and does not replace professional medical advice, diagnosis, or treatment. Always seek the guidance of your urologist or healthcare provider with any questions regarding a medical condition. If you experience life-threatening symptoms, call emergency services immediately.

A sudden, severe worsening of IC/BPS pain (often called a "flare") can be debilitating and frightening. Below is a step-by-step, evidence-based guide to managing the situation safely and effectively.

Immediate First Steps (First 1-2 Hours)

**Stop all potential triggers immediately**

Discontinue any new foods, beverages, supplements, or medications you started in the last 24-48 hours

Avoid all known IC triggers: coffee, tea, alcohol, carbonated drinks, citrus, tomatoes, spicy foods, chocolate, artificial sweeteners (especially aspartame)

Cease any physical activity that may be irritating the pelvic floor (running, lifting, sexual activity)

**Adjust your hydration carefully**

Sip small amounts of plain, room-temperature water frequently (2-3 ounces every 15-20 minutes)

**Do NOT overhydrate** (this can further stretch and irritate the bladder)

**Do NOT dehydrate** (concentrated urine is more irritating to the bladder lining)

Avoid cold or iced beverages, which can trigger bladder spasms in some people

**Find a comfortable position**

Lie down on your side with a pillow between your knees

Try sitting on a padded seat or donut cushion to reduce pelvic pressure

Avoid sitting for long periods; alternate between lying down and gentle standing

**Apply gentle heat therapy**

Place a warm (not hot) heating pad or warm water bottle on your lower abdomen or lower back

Take a warm (not hot) bath for 10-15 minutes (avoid bubble baths, bath salts, or scented products)

Heat helps relax the pelvic floor muscles and reduce spasms

At-Home Pain Management Strategies

Medications (Use only as directed by your healthcare provider)

**Oral analgesics**: Acetaminophen (paracetamol) is generally the safest first-line option for IC pain

**Avoid**: Non-steroidal anti-inflammatory drugs (NSAIDs) like ibuprofen and naproxen, as they can irritate the bladder lining in many people with IC

**Antispasmodics**: If prescribed by your doctor, medications like oxybutynin, tolterodine, or mirabegron may help reduce bladder spasms

**Prescription pain medications**: If you have been prescribed stronger pain relievers for IC flares, use them exactly as directed

Physical Comfort Measures

**Pelvic floor relaxation exercises**: Practice slow, deep breathing while consciously relaxing your pelvic floor muscles (imagine you are trying to stop urinating, then release completely)

**Gentle stretching**: Try child's pose or knees-to-chest stretches to relieve pelvic tension

**Avoid tight clothing**: Wear loose, comfortable pants and underwear (preferably cotton)

Bladder Calming Techniques

**Timed voiding**: Urinate every 30-60 minutes, even if you don't feel the urge, to prevent bladder distension

**Double voiding**: After urinating, wait 30 seconds, then try again to empty your bladder more completely

**Avoid holding urine**: Holding urine can significantly increase pain and pressure during a flare

When to Seek URGENT Medical Attention (Red Flags)

Contact your urologist immediately or go to the emergency department if you experience any of these symptoms along with severe pain:

**Fever (temperature above 100.4°F/38°C) or chills** (may indicate a urinary tract infection or kidney infection)

**Blood in your urine (hematuria) that is bright red or contains clots**

**Inability to urinate at all (urinary retention)**

**Severe pain that is completely unresponsive to your prescribed medications**

**Pain that radiates to your back, sides, or groin**

**Nausea and vomiting that prevents you from keeping down fluids or medications**

**New or worsening urinary incontinence**

**Symptoms that last longer than 24-48 hours without improvement**

What to Tell Your Healthcare Provider

Be prepared to provide the following information to help your doctor diagnose and treat your flare effectively:

When the pain started and how it has progressed

The severity of your pain (on a scale of 0-10)

Any new triggers you may have been exposed to

All medications you have taken (including over-the-counter drugs and supplements)

Any other symptoms you are experiencing (fever, blood in urine, etc.)

What measures you have already tried to relieve the pain

Post-Flare-Up Management

Once the severe pain subsides:

Gradually return to your normal diet, reintroducing one food at a time to identify potential triggers

Continue gentle pelvic floor relaxation exercises for several days

Schedule a follow-up appointment with your urologist to discuss the flare and adjust your treatment plan if necessary

Consider keeping a symptom journal to track flares and identify patterns

Remember that IC/BPS is a chronic condition, and flares are a common part of the disease process. Having a personalized flare action plan developed with your healthcare provider can help you manage severe episodes more effectively and reduce anxiety about future flares.

**19. What should someone do if they cannot urinate while having interstitial cystitis symptoms?**

Urinary Retention with Interstitial Cystitis: Immediate Action Guide

**URGENT DISCLAIMER**: Complete inability to urinate (urinary retention) is a **medical emergency** that can cause permanent kidney damage, bladder rupture, or life-threatening infection if not treated promptly. This information is for educational purposes only and does not replace emergency medical care.

Immediate Emergency Actions (Do This First)

**Go to the nearest emergency department immediately** if you:

Cannot pass any urine at all for 4-6 hours

Have severe lower abdominal pain, distension, or tenderness

Experience nausea, vomiting, fever, or chills

Feel lightheaded, dizzy, or confused

**Call your urologist immediately** if you have partial retention (only dribbling small amounts) with worsening pain, as they may be able to see you urgently or direct you to the appropriate care.

What to Do While Waiting for Medical Help

**Sit in a warm bath** (not hot) for 10-15 minutes to relax the pelvic floor muscles, which may help relieve spasms that are blocking urine flow

**Apply a warm heating pad** to your lower abdomen (avoid direct skin contact to prevent burns)

**Try running water** in the sink or shower; the sound may trigger the urinary reflex

**Avoid straining** to urinate, as this can worsen pelvic floor tension and cause further damage

**Do not drink excessive fluids** in an attempt to "force" urination; this will only increase bladder pressure and pain

What You Absolutely Should NOT Do

**Do not take over-the-counter decongestants, antihistamines, or some antidepressants** (these can worsen urinary retention by relaxing the bladder muscle or tightening the urethral sphincter)

**Do not insert anything into your urethra** to try to empty your bladder (this can cause severe injury, infection, or permanent damage)

**Do not delay seeking care** hoping the problem will resolve on its own; bladder pressure can build rapidly and cause irreversible harm

Why Urinary Retention Happens in Interstitial Cystitis Patients

True urinary retention is **not a typical symptom of uncomplicated interstitial cystitis** (which usually causes frequency, urgency, and pain with small urine volumes). When it does occur, it is most often due to:

Severe pelvic floor muscle spasms (the most common cause in IC patients)

Bladder neck obstruction

A urinary tract infection (UTI) superimposed on IC

Bladder stones

Side effects of medications used to treat IC or other conditions

Rarely, bladder cancer or other structural abnormalities

Medical Treatments You May Receive

In the emergency department, the first priority is to relieve bladder pressure:

**Urinary catheterization**: A thin tube is inserted through the urethra to drain the bladder. This provides immediate relief and prevents complications.

**Antispasmodic medications**: To relax the bladder and pelvic floor muscles.

**Pain management**: To relieve severe discomfort.

**Testing**: Urinalysis, urine culture, and possibly imaging (ultrasound) to identify the underlying cause.

Follow-Up Care

After the acute episode resolves:

Schedule an urgent appointment with your urologist who specializes in interstitial cystitis

Review all your medications with your doctor to identify any that may contribute to retention

Consider pelvic floor physical therapy to address muscle spasms (a common underlying cause)

Develop a personalized emergency plan with your doctor for future episodes

When to Seek Emergency Care Again

Return to the emergency department immediately if:

You cannot urinate again after catheter removal

You develop fever, chills, or severe pain

You notice blood in your urine

You have difficulty emptying your bladder completely

Remember: While interstitial cystitis causes significant urinary symptoms, true urinary retention is always a red flag that requires immediate medical evaluation.

**20. What type of doctor usually treats interstitial cystitis?**

Doctors Who Treat Interstitial Cystitis (IC/Painful Bladder Syndrome)

Interstitial cystitis (IC) is a complex chronic bladder condition that requires specialized care. The primary and most common doctors who treat IC are:

Primary Specialists

1. **Urologists**

Urologists are the main specialists for interstitial cystitis. They have extensive training in disorders of the urinary tract, including the bladder, kidneys, ureters, and urethra. Many urologists develop specific expertise in treating chronic pelvic pain and IC, which is essential given the condition's often challenging diagnosis and management.

2. **Urogynecologists**

For women with IC, urogynecologists are an excellent choice. These specialists are board-certified in both urology and gynecology, with advanced training in female pelvic floor disorders. They are particularly skilled at addressing the overlap between IC, pelvic floor dysfunction, and other gynecological conditions that commonly coexist with IC.

Multidisciplinary Care Team

Because IC is a complex condition that affects multiple body systems and often has comorbidities, your care may involve additional specialists:

**Pelvic floor physical therapists**: Specialize in treating pelvic floor muscle dysfunction, which is present in up to 70% of IC patients

**Pain management specialists**: Help manage severe, chronic pelvic pain that doesn't respond to standard treatments

**Gastroenterologists**: Address overlapping conditions like irritable bowel syndrome (IBS), which affects about 50% of IC patients

**Rheumatologists**: Evaluate and treat potential autoimmune components of IC

**Mental health professionals**: Provide support for the anxiety, depression, and quality of life issues that often accompany chronic illness

Primary Care Physician Role

Your primary care doctor (PCP) can help recognize IC symptoms and refer you to the appropriate specialist. However, most PCPs do not have the specialized training needed to diagnose and manage interstitial cystitis effectively.

**Important note**: When seeking care, look for doctors who specifically mention experience treating interstitial cystitis or painful bladder syndrome, as many general urologists may not be familiar with the latest diagnostic criteria and treatment approaches.

**Disclaimer**: This information is for educational purposes only and is not a substitute for professional medical advice, diagnosis, or treatment. Always seek the advice of your physician or other qualified health provider with any questions you may have regarding a medical condition.

**21. How do doctors usually diagnose interstitial cystitis?**

Diagnosis of Interstitial Cystitis/Bladder Pain Syndrome (IC/BPS)

Interstitial cystitis (now officially called **interstitial cystitis/bladder pain syndrome, IC/BPS**) is a **diagnosis of exclusion**—there is no single definitive test that can confirm it. Doctors must rule out all other conditions that cause similar symptoms before making an IC/BPS diagnosis. The diagnostic process follows guidelines from the American Urological Association (AUA) and typically includes the following steps:

1. Comprehensive Medical History and Symptom Assessment

This is the **most critical part** of the diagnosis. Your doctor will ask detailed questions about:

**Pain characteristics**: Location (pelvic, bladder, urethral, lower abdominal), quality (burning, pressure, sharp), timing (worse with bladder filling, relieved partially by urination)

**Urinary symptoms**: Frequency (how often you urinate during the day and night), urgency (sudden, strong need to urinate), nocturia (waking up to urinate)

**Symptom duration**: IC/BPS requires symptoms to persist for **at least 6 weeks**

**Triggers**: Foods/drinks (coffee, alcohol, spicy foods, citrus), stress, sexual activity, menstruation

**Past medical history**: Previous urinary tract infections (UTIs), pelvic surgeries, gynecological conditions, autoimmune diseases, mental health history (anxiety/depression)

Doctors may use validated questionnaires to quantify symptoms, such as the **O'Leary-Sant Symptom and Problem Index** or the **Pelvic Pain and Urgency/Frequency (PUF) Patient Symptom Scale**.

2. Physical Examination

**Pelvic exam** (for women): To check for tenderness in the bladder, urethra, or pelvic floor muscles; rule out endometriosis, pelvic inflammatory disease (PID), or vaginal atrophy

**Digital rectal exam** (for men and women): To check the prostate (in men) and assess pelvic floor muscle tension

**Abdominal exam**: To look for tenderness or masses

3. Basic Laboratory Tests

These are used to **rule out other common conditions**:

**Urinalysis and urine culture**: To exclude bacterial UTIs, blood in the urine (hematuria), or proteinuria

**Urine cytology**: To look for abnormal cells that might indicate bladder cancer (especially in smokers or patients over 40 with hematuria)

**Kidney function tests**: To assess overall renal health

4. Cystoscopy with Hydrodistention

This is the **most common diagnostic procedure** for IC/BPS, though it is not required for all patients (especially those with classic symptoms and no red flags).

What happens during the procedure:

A thin, flexible tube with a camera (cystoscope) is inserted through the urethra into the bladder

The bladder is filled with sterile water to stretch it (hydrodistention) while the patient is under anesthesia

Key findings that support IC/BPS:

**Glomerulations**: Pinpoint bleeding spots on the bladder wall that appear when the bladder is stretched (present in most IC/BPS patients)

**Hunner's lesions**: Ulcer-like patches on the bladder wall (present in only 5-10% of patients, but are pathognomonic for IC/BPS when found)

Decreased bladder capacity

5. Additional Tests (When Needed)

These are performed only if there are red flags or if the diagnosis remains unclear:

**Bladder biopsy**: To rule out bladder cancer, eosinophilic cystitis, or other inflammatory conditions (not routinely done for IC/BPS)

**Urodynamic testing**: To evaluate bladder function and rule out overactive bladder (OAB) or bladder outlet obstruction

**Potassium sensitivity test (PST)**: A test where a potassium solution is instilled into the bladder to see if it reproduces pain (controversial and not universally recommended)

**Imaging studies**: Ultrasound, CT scan, or MRI to rule out kidney stones, tumors, or structural abnormalities

Diagnostic Criteria

According to the AUA, the diagnosis of IC/BPS requires:

**Chronic pelvic pain, pressure, or discomfort** that is perceived to be related to the bladder

**At least one other urinary symptom**: Urinary frequency or urgency

**Exclusion of all other identifiable causes** of these symptoms

Important Notes

IC/BPS is often misdiagnosed as recurrent UTIs, overactive bladder, endometriosis (in women), or prostatitis (in men)

The diagnostic process may take several months as doctors rule out other conditions

There is no cure for IC/BPS, but early diagnosis and treatment can significantly improve quality of life

**Disclaimer**: This information is for educational purposes only and is not a substitute for professional medical advice, diagnosis, or treatment. Always seek the advice of your physician or other qualified health provider with any questions you may have regarding a medical condition.

**22. Is there one test that can prove someone has interstitial cystitis?**

**No, there is no single test that can definitively prove someone has interstitial cystitis/bladder pain syndrome (IC/BPS)**. IC/BPS is a diagnosis of exclusion, meaning healthcare providers must first rule out all other conditions that cause similar symptoms before making the diagnosis.

Why There Is No Single Definitive Test

IC/BPS is a complex, heterogeneous condition with no known single cause. It does not produce a unique biomarker, imaging finding, or laboratory result that is present in all patients. The symptoms of IC/BPS overlap significantly with many other urological, gynecological, and gastrointestinal conditions.

The Standard Diagnostic Process

According to the **American Urological Association (AUA) 2022 Guidelines**, the diagnosis of IC/BPS is based primarily on:

**Characteristic clinical symptoms**: Chronic pelvic pain, pressure, or discomfort perceived to be related to the urinary bladder, accompanied by at least one other urinary symptom (increased daytime frequency, nocturia, or urinary urgency)

**Exclusion of all other possible causes**

Tests Used in the Diagnostic Workup

| **Test** | **Purpose** | **Notes** |
| --- | --- | --- |
| Urinalysis + Urine Culture | Rule out urinary tract infections (UTIs) | The most critical first step, as UTIs are the most common cause of bladder pain |
| Urine Cytology | Rule out bladder cancer | Especially important in smokers, patients over 40, or those with hematuria (blood in urine) |
| Bladder Diary | Document voiding patterns, fluid intake, and symptom correlation | Helps distinguish IC/BPS from overactive bladder |
| Physical Examination | Assess for pelvic floor dysfunction, endometriosis (in women), or prostatitis (in men) | Pelvic floor tenderness is common in IC/BPS patients |
| Cystoscopy with Hydrodistention | Evaluate bladder lining and capacity | **Not a definitive test** |
| Urodynamics | Assess bladder function | Used when other diagnoses like overactive bladder or bladder outlet obstruction are suspected |

The Myth of Cystoscopy as a "Gold Standard"

Cystoscopy with hydrodistention was once considered the gold standard for IC/BPS diagnosis, but this is no longer true:

**Hunner's lesions** (ulcerations on the bladder wall) are specific to IC/BPS but only present in **5-10% of patients**

**Glomerulations** (pinpoint bleeding after bladder stretching) are seen in many IC/BPS patients but can also occur in asymptomatic people and those with other bladder conditions

Up to 30% of patients with classic IC/BPS symptoms have completely normal cystoscopy findings

Controversial Tests

**Potassium Sensitivity Test (PST)**: Involves instilling a potassium solution into the bladder to test for pain. It is no longer recommended as a routine diagnostic test by the AUA due to high false positive/negative rates and significant patient discomfort.

**Biopsy**: Rarely needed for diagnosis, except to rule out bladder cancer or other rare conditions.

Disclaimer

This information is provided for educational purposes only and is not a substitute for professional medical advice, diagnosis, or treatment. Always seek the advice of your physician or other qualified health provider with any questions you may have regarding a medical condition.

**23. Why do doctors need to rule out other problems before diagnosing interstitial cystitis?**

Interstitial cystitis, now formally called **interstitial cystitis/bladder pain syndrome (IC/BPS)**, is a **diagnosis of exclusion**—meaning it can only be confirmed after all other potential causes of the patient's symptoms have been systematically eliminated. This approach is not optional; it is a fundamental requirement of clinical practice for several critical, evidence-based reasons:

1. There is no single, definitive diagnostic test for IC/BPS

Unlike diabetes (blood glucose test) or urinary tract infections (urine culture), there is no biomarker, imaging study, or laboratory test that can definitively diagnose IC/BPS on its own. Even the most commonly used procedures—cystoscopy with hydrodistention and bladder biopsy—are not 100% specific:

Many patients with confirmed IC/BPS do not show the classic Hunner's lesions or glomerulations (pinpoint bleeding) on cystoscopy

Some people without IC/BPS may develop glomerulations after bladder distention

Bladder biopsy findings are non-specific and primarily used to rule out other conditions like cancer or eosinophilic cystitis

2. IC/BPS symptoms overlap extensively with dozens of other conditions

The core symptoms of IC/BPS—**chronic pelvic pain, urinary urgency, frequency, and nocturia**—are shared by a wide range of medical conditions across multiple organ systems. Many of these conditions are far more common than IC/BPS and require completely different treatments.

Most common mimics requiring urgent or immediate exclusion

| **Organ System** | **Conditions That Mimic IC/BPS** | **Critical Distinction** |
| --- | --- | --- |
| **Urinary Tract** | Recurrent urinary tract infections (UTIs)  Bladder cancer  Kidney/bladder stones  Overactive bladder (OAB)  Neurogenic bladder  Radiation cystitis  Chemical cystitis | UTIs require antibiotics; bladder cancer requires surgery/chemotherapy; stones may need lithotripsy |
| **Gynecological (Women)** | Endometriosis  Pelvic inflammatory disease (PID)  Vulvodynia/vaginismus  Uterine fibroids  Ovarian cysts/cancer | Endometriosis affects 10-15% of reproductive-age women and is the most common misdiagnosis for IC/BPS in women |
| **Male Reproductive** | Chronic prostatitis/chronic pelvic pain syndrome (CP/CPPS)  Benign prostatic hyperplasia (BPH)  Prostate cancer | CP/CPPS affects 2-10% of men and shares nearly identical symptoms with male IC/BPS |
| **Gastrointestinal** | Irritable bowel syndrome (IBS)  Inflammatory bowel disease (Crohn's, ulcerative colitis)  Diverticulitis | Up to 70% of IC/BPS patients also have IBS, making differentiation challenging |
| **Neurological** | Multiple sclerosis  Spinal cord injury  Herpes zoster (shingles) affecting the sacral nerves | Neurological conditions require specific disease-modifying treatments |

3. Missing a serious underlying condition can have life-threatening consequences

Some conditions that mimic IC/BPS are potentially fatal if not diagnosed promptly:

**Bladder cancer**: Presents with pelvic pain, frequency, and sometimes hematuria (blood in urine). Delayed diagnosis significantly reduces survival rates.

**Ovarian cancer**: Often causes pelvic pain and urinary frequency in its early stages, when it is most treatable.

**Kidney stones**: Can lead to kidney damage, sepsis, or renal failure if left untreated.

**Pelvic inflammatory disease**: Can cause infertility, chronic pelvic pain, and ectopic pregnancy if not treated with antibiotics.

4. Misdiagnosis leads to ineffective, harmful, or unnecessary treatment

IC/BPS treatments are often long-term, expensive, and can have significant side effects. Administering these treatments to a patient who actually has a different condition is not only ineffective but can also be dangerous:

Prescribing antibiotics for IC/BPS (which is not an infection) contributes to antibiotic resistance and may cause gastrointestinal issues or yeast infections.

Bladder instillations, nerve stimulation, or surgery for IC/BPS will not help a patient with endometriosis or kidney stones.

Conversely, treating a patient with IC/BPS with OAB medications (anticholinergics) may worsen their pain and urinary retention.

5. IC/BPS is a rare condition relative to its mimics

IC/BPS affects approximately 3-8 million women and 1-4 million men in the United States. In contrast:

UTIs affect over 150 million people worldwide annually

Overactive bladder affects about 33 million Americans

Endometriosis affects 1 in 10 women of reproductive age

Given these prevalence rates, a patient presenting with pelvic pain and urinary symptoms is statistically far more likely to have one of these common conditions than IC/BPS.

The Standard Diagnostic Process

According to the American Urological Association (AUA) guidelines, the diagnostic workup for suspected IC/BPS must include, at a minimum:

Detailed medical history and symptom assessment (using validated tools like the O'Leary-Sant Symptom Index)

Physical examination (including pelvic exam for women and digital rectal exam for men)

Urinalysis and urine culture to rule out infection

Urine cytology to screen for bladder cancer in high-risk patients (smokers, those over 40, history of hematuria)

Additional tests may be ordered based on the patient's presentation, including renal ultrasound, cystoscopy, urodynamic testing, or bladder biopsy—all primarily to exclude other diagnoses.

Disclaimer

This information is provided for educational purposes only and is not a substitute for professional medical advice, diagnosis, or treatment. Always seek the advice of your physician or other qualified health provider with any questions you may have regarding a medical condition.

**24. Why do doctors check urine when interstitial cystitis is suspected?**

Doctors perform urine testing as the **first and most critical step** in evaluating suspected interstitial cystitis (IC, also called bladder pain syndrome/BPS) because IC is a **diagnosis of exclusion**—it can only be confirmed after all other conditions with identical symptoms have been ruled out. Standard urine tests are almost always normal in uncomplicated IC, which itself is an important diagnostic clue.

1. Primary Purpose: Rule Out Life-Threatening and Treatable Mimics

IC shares nearly identical symptoms (bladder pain, urinary urgency, frequency, nocturia) with many other conditions that require completely different treatments. Urine testing efficiently eliminates these possibilities before more invasive procedures are considered.

Most Common Mimic: Urinary Tract Infections (UTIs)

**Urinalysis** detects white blood cells (leukocytes), nitrites, and bacteria—the hallmarks of bacterial infection

**Urine culture** confirms the presence of pathogenic bacteria and identifies appropriate antibiotics

This is critical because untreated UTIs can progress to kidney infections, while IC does not respond to antibiotics

Note: Many IC patients are misdiagnosed with recurrent UTIs for years before receiving an IC diagnosis

Rule Out Bladder Cancer and Precancerous Lesions

**Microscopic hematuria** (blood in urine visible only under a microscope) is never normal and requires investigation

**Urine cytology** examines urine for abnormal cells that may indicate bladder cancer or carcinoma in situ

This is especially important for patients over 40, smokers, or those with a history of chemical exposure—groups at higher risk for bladder cancer

Rule Out Kidney Stones and Crystalluria

Urinalysis detects microscopic crystals (calcium oxalate, struvite, etc.) that indicate stone formation

Stones can cause severe bladder pain, urgency, and frequency that mimics IC flares

Even small stones passing through the ureter can produce symptoms identical to IC

Rule Out Sexually Transmitted Infections (STIs)

Urine tests for chlamydia and gonorrhea rule out urethritis, which causes urinary urgency, pain, and frequency

These infections require antibiotic treatment and partner notification

Rule Out Systemic Conditions

**Glucose** in urine indicates undiagnosed diabetes, a common cause of frequent urination

**Protein** in urine suggests kidney disease, which can present with urinary symptoms

**Eosinophils** in urine may indicate eosinophilic cystitis, a rare inflammatory bladder condition that mimics IC

2. Adjunctive: Emerging Biomarker Research (Not Standard Clinical Use)

While no urine test can definitively diagnose IC, researchers are investigating potential biomarkers that may support the diagnosis or predict treatment response:

**Glycosaminoglycans (GAGs)**: Decreased levels in urine may indicate damage to the bladder's protective lining

**Nerve growth factor (NGF)**: Elevated levels correlate with bladder pain severity in IC patients

**Inflammatory cytokines**: Increased levels of IL-6, IL-8, and TNF-alpha may indicate bladder inflammation

These tests are not yet approved for routine clinical use and are primarily used in research settings

3. Additional Clinical Value

Establishes baseline kidney function before initiating IC treatments that may affect renal health

Guides decisions about further testing (e.g., cystoscopy, urodynamics)

Monitors for complications during long-term IC management

Key Takeaway

A normal urinalysis and negative urine culture are **required components** of the IC diagnostic process according to the American Urological Association (AUA) guidelines. If urine tests show abnormalities, the diagnosis of IC is unlikely, and further evaluation for the identified condition is necessary.

**Disclaimer**: This information is for educational purposes only and should not replace professional medical advice. Always consult a qualified healthcare provider for diagnosis and treatment of any medical condition.

**25. Is looking inside the bladder always needed to diagnose interstitial cystitis?**

Is Cystoscopy Always Required to Diagnose Interstitial Cystitis/Bladder Pain Syndrome (IC/BPS)?

**No, cystoscopy (looking inside the bladder) is NOT always required to make a diagnosis of interstitial cystitis/bladder pain syndrome (IC/BPS)** according to current international clinical guidelines. However, it plays an important role in specific clinical scenarios and for identifying certain subtypes of the condition.

Current Guideline Recommendations

American Urological Association (AUA) 2021 Guidelines

The AUA explicitly states that **cystoscopy is not mandatory for the initial diagnosis of IC/BPS** in patients with typical symptoms and no red flags. The diagnosis is primarily clinical, based on:

Characteristic symptoms (chronic pelvic pain, pressure, or discomfort perceived to be related to the urinary bladder, associated with at least one other urinary symptom such as increased daytime or nighttime frequency)

Exclusion of other conditions that can cause similar symptoms

European Association of Urology (EAU) 2023 Guidelines

The EAU similarly recommends that **cystoscopy is not required for the initial diagnosis** of uncomplicated IC/BPS. It should be reserved for patients with atypical presentations, risk factors for other diseases, or those who fail first-line treatments.

When Cystoscopy IS Recommended

Cystoscopy (often with hydrodistension under anesthesia) is indicated in the following situations:

**To identify Hunner's lesions**: These are characteristic ulcer-like lesions found in approximately 5-10% of IC/BPS patients (classic "ulcerative IC"). Their presence confirms the diagnosis and may guide specific treatments.

**When there are red flags suggesting other conditions**:

Hematuria (blood in the urine)

Urinary tract infections that are recurrent or persistent

Voiding dysfunction

History of bladder cancer or other urological malignancies

Pelvic radiation therapy

Previous bladder surgery

**When initial treatments fail**: To reassess the diagnosis and look for other causes of symptoms

**To rule out bladder stones, tumors, or structural abnormalities**

**In patients with severe symptoms or rapidly progressive disease**

What Cystoscopy Can and Cannot Show

**Can show**: Hunner's lesions, glomerulations (pinpoint bleeding after bladder distension), bladder capacity, inflammation, stones, tumors, and structural abnormalities

**Cannot show**: Non-ulcerative IC/BPS in many cases, as the bladder lining may appear completely normal in up to 90% of patients

The Standard Diagnostic Process Without Routine Cystoscopy

A diagnosis of IC/BPS can be made confidently without cystoscopy through:

**Thorough medical history**: Focusing on symptom duration, character, and triggers

**Physical examination**: Including pelvic exam in women

**Laboratory tests**: Urinalysis, urine culture, and sometimes cytology to rule out infection and cancer

**Symptom questionnaires**: Validated tools like the O'Leary-Sant IC Symptom and Problem Index (ICSI/ICPI)

**Bladder diary**: To document frequency, volume, and symptom correlation

**Exclusion of differential diagnoses**: Such as urinary tract infections, overactive bladder, endometriosis, pelvic floor dysfunction, and prostate problems in men

Important Considerations

Glomerulations (previously considered a hallmark of IC/BPS) are no longer regarded as diagnostic on their own, as they can occur in asymptomatic individuals and in other bladder conditions.

The absence of abnormal findings on cystoscopy does NOT rule out IC/BPS. In fact, most patients with non-ulcerative IC/BPS have normal-appearing bladders.

Cystoscopy with hydrodistension can sometimes provide temporary symptom relief, but this is not a reliable diagnostic test.

Disclaimer

This information is for educational purposes only and is not a substitute for professional medical advice, diagnosis, or treatment. Always seek the advice of your physician or other qualified health provider with any questions you may have regarding a medical condition.

**26. Can food or drinks make interstitial cystitis symptoms worse?**

**Yes, absolutely.** Diet is one of the most significant modifiable factors affecting interstitial cystitis (also called painful bladder syndrome) symptoms. Approximately 70-90% of people with IC report that certain foods and beverages trigger or worsen their bladder pain, urgency, frequency, and pressure .

Why Food Triggers IC Symptoms

The exact mechanism isn't fully understood, but it's believed that certain substances in food:

Irritate the already damaged and inflamed bladder lining (urothelium)

Increase urine acidity, which can stimulate nerve endings in the bladder

Cause systemic inflammation that exacerbates bladder inflammation

May contain molecules that mimic or interact with neurotransmitters involved in bladder pain signaling

Most Common Trigger Foods and Drinks

Triggers vary significantly from person to person, but these are the most frequently reported:

1. Acidic Foods and Beverages

Citrus fruits (oranges, lemons, limes, grapefruits) and their juices

Tomatoes and tomato-based products (sauce, salsa, ketchup)

Cranberries and cranberry juice (ironically, often recommended for urinary tract infections but harmful for IC)

Pineapple, strawberries, raspberries

Vinegar and vinegar-based products (pickles, salad dressings)

2. Caffeine

Coffee (both regular and decaf, as decaf still contains small amounts of caffeine)

Black tea, green tea, and energy drinks

Chocolate and chocolate products

Some sodas and over-the-counter medications

3. Alcohol

All types of alcohol, including beer, wine, and spirits

Alcohol dehydrates the body and irritates the bladder lining

4. Artificial Sweeteners

Aspartame (NutraSweet, Equal) is the most well-documented trigger

Saccharin (Sweet'N Low)

Sucralose (Splenda) may also trigger symptoms in some people

5. Carbonated Beverages

All sodas, including sparkling water and seltzer

The carbonation itself can irritate the bladder, even in sugar-free, caffeine-free varieties

6. Spicy Foods

Hot peppers, chili powder, curry, and other spicy seasonings

Can cause systemic inflammation and bladder irritation

7. Other Common Triggers

Aged cheeses

Processed meats (bacon, sausage, deli meats)

Onions and garlic

Nuts (especially peanuts and almonds)

Certain fish (smoked fish, caviar)

MSG (monosodium glutamate)

How to Identify Your Personal Triggers

The gold standard for identifying individual triggers is an **elimination diet**:

Eliminate all potential trigger foods and drinks for 2-4 weeks until symptoms improve

Reintroduce one food at a time, waiting 2-3 days between each reintroduction

Keep a detailed food and symptom diary to track reactions

Permanently avoid foods that consistently worsen your symptoms

General Dietary Tips for IC Management

Stay hydrated with plain water (dehydration can concentrate urine and worsen irritation)

Eat a balanced diet rich in anti-inflammatory foods (leafy greens, fatty fish, whole grains)

Consider trying an alkaline diet to reduce urine acidity

Be cautious with new foods and medications, as they may contain hidden triggers

Disclaimer

This information is for educational purposes only and is not a substitute for professional medical advice, diagnosis, or treatment. Always seek the advice of your physician or other qualified health provider with any questions you may have regarding a medical condition.

**27. Which drinks commonly bother people with interstitial cystitis?**

Interstitial cystitis/bladder pain syndrome (IC/BPS) is a chronic condition characterized by bladder pain, urinary urgency, and frequency. The bladder lining in people with IC is often damaged or hypersensitive, making it vulnerable to irritation from certain substances in drinks. Below are the most common problematic beverages, organized by their primary irritant mechanism:

1. Caffeinated Beverages (Most Common Trigger)

Caffeine is both a potent diuretic (increases urine production) and a direct bladder stimulant that can worsen urgency, frequency, and pain. Even small amounts can trigger symptoms in sensitive individuals.

**Regular coffee** (highest caffeine content)

**Decaffeinated coffee** (still contains trace amounts of caffeine and acidic compounds)

**Black, green, oolong, and white teas**

**Energy drinks** (extremely high caffeine plus other stimulants like taurine)

**Caffeinated sodas** (cola, Mountain Dew, etc.)

2. Alcoholic Beverages

Alcohol irritates the bladder lining, dehydrates the body (concentrating urine), and increases urine production. All types of alcohol can trigger symptoms:

Beer

Wine (especially red wine, which is also acidic)

Liquor and cocktails

Hard seltzers (often contain added flavors and carbonation as well)

3. Carbonated Drinks

The carbon dioxide bubbles themselves can irritate the sensitive bladder wall, even in drinks without caffeine or sugar.

Regular and diet sodas

Sparkling water, seltzer, and club soda

Sparkling wine and champagne

Carbonated energy drinks

4. Acidic Drinks

Acidic substances can erode the already compromised bladder lining and trigger pain and urgency.

**Citrus juices**: Orange, grapefruit, lemon, lime, and tangerine

**Cranberry juice** (critical misconception: while often recommended for urinary tract infections, cranberry is highly acidic and is one of the most common IC triggers)

Tomato juice and tomato-based drinks

Pineapple juice

Some apple and grape juices (depending on variety and ripeness)

Vinegar-based drinks (like apple cider vinegar shots)

5. Drinks with Artificial Sweeteners

Artificial sweeteners are well-documented bladder irritants for many people with IC, even in small amounts.

Aspartame (NutraSweet, Equal)

Saccharin (Sweet'N Low)

Sucralose (Splenda)

Acesulfame potassium (Ace-K)

Found in: Diet sodas, sugar-free drinks, some flavored waters, sugar-free sports drinks, and some "healthy" beverage mixes

6. Other Problematic Drinks

**Flavored waters** (often contain artificial sweeteners, natural flavors, or citric acid)

**Sports drinks** (high in sugar, artificial sweeteners, and sometimes citric acid)

**Certain herbal teas** (especially those with citrus, mint, cinnamon, or other strong flavors; chamomile is generally better tolerated)

**Fruit punch and pre-made smoothies** (high in sugar and acidic fruit concentrates)

Important Notes

**Individual variability**: Triggers vary significantly from person to person. A drink that causes severe symptoms in one person may be well-tolerated by another.

**Symptom diary**: Keeping a detailed food and symptom diary is the most effective way to identify your personal triggers.

**Elimination diet**: Many people with IC find relief by following an elimination diet that removes all common triggers, then gradually reintroducing them one at a time.

Safer Alternatives

Plain, room-temperature water (cold water can sometimes irritate sensitive bladders)

Diluted, low-acid fruit juices (if tolerated)

Unsweetened oat milk or almond milk

Caffeine-free, low-acid herbal teas (chamomile, rooibos)

Filtered tap water or spring water

**Disclaimer**: This information is for educational purposes only and is not a substitute for professional medical advice, diagnosis, or treatment. Always seek the advice of your physician or other qualified health provider with any questions you may have regarding a medical condition.

**28. Can coffee make interstitial cystitis symptoms worse?**

**Yes, coffee is one of the most well-documented and potent triggers for worsening interstitial cystitis (IC)/bladder pain syndrome symptoms.**

The Evidence

Multiple authoritative sources confirm this relationship:

The Interstitial Cystitis Network identifies coffee and tea as the **1 worst bladder offenders** for IC patients

The National Institute of Diabetes and Digestive and Kidney Diseases (NIDDK) lists coffee as a common trigger for symptom flares National Institute of Diabetes and Digestive and Kidney Diseases (NIDDK)

Johns Hopkins Medicine includes coffee and caffeinated drinks among foods that make IC symptoms worse

Clinical studies show that **97% of IC patients** report their pain worsens with certain foods and drinks, with coffee consistently ranking among the top triggers

Why Coffee Irritates the IC Bladder

Coffee affects IC symptoms through multiple mechanisms:

**Caffeine content**:

Acts as a potent diuretic, increasing urine production and bladder filling frequency

Directly stimulates the detrusor muscle (bladder wall muscle), causing more frequent and involuntary contractions

Lowers pain thresholds and increases nerve sensitivity in the already hypersensitive bladder

Accelerates bladder muscle contraction, worsening urgency and frequency

**Acidity**:

Coffee's high acid content directly irritates the damaged bladder lining (urothelium) characteristic of IC

Creates a caustic urine environment that can reach deeper into the bladder wall, stimulating nerves and triggering inflammation

Even small amounts of acid can damage the vulnerable stem cells trying to repair the bladder lining

**Other compounds**:

Tannins and oils present in coffee can also contribute to bladder irritation

What About Decaf Coffee?

**Decaf coffee is not necessarily safe for IC patients.** While it contains significantly less caffeine (typically 2-15 mg per cup compared to 70-140 mg in regular coffee), it still retains most of its acidity . Many IC patients report that decaf coffee still triggers their symptoms .

The Interstitial Cystitis Network strongly recommends avoiding all coffees (both regular and decaf) for at least three months when symptoms are active to give the bladder a chance to heal .

Individual Variability

While coffee is a common trigger, individual tolerance varies. Some IC patients may be able to tolerate:

Very small amounts of coffee

Low-acid coffee varieties

Coffee consumed with food to buffer acidity

The best approach is to:

Eliminate all coffee completely during an initial elimination phase (3-6 months)

If symptoms improve, you can try reintroducing small amounts of low-acid decaf coffee

Keep a detailed food and symptom diary to track your personal response

Recommendations

Most IC guidelines recommend avoiding coffee as a first-line dietary modification. If you struggle with giving up coffee entirely, consider trying herbal coffee alternatives like Dandy Blend®, which are naturally caffeine-free and low in acid .

**Disclaimer**: This information is for educational purposes only and is not a substitute for professional medical advice, diagnosis, or treatment. Always seek the advice of your physician or other qualified health provider with any questions you may have regarding a medical condition.

**29. Does everyone with interstitial cystitis need to avoid the same foods?**

Do All Interstitial Cystitis Patients Need to Avoid the Same Foods?

**No.** Dietary triggers for interstitial cystitis/bladder pain syndrome (IC/BPS) are **highly individual**, and there is no universal "one-size-fits-all" elimination diet that works for everyone.

Why Individual Variation Exists

IC/BPS is a heterogeneous condition with multiple underlying pathophysiological mechanisms, including:

Bladder epithelial barrier dysfunction (leaky bladder)

Neurogenic inflammation and central sensitization

Mast cell activation

Differences in gut microbiome composition

Individual genetic variations in metabolism and sensory processing

These differences mean that a food that causes severe symptoms in one person may have no effect whatsoever in another.

Common (But Not Universal) Dietary Triggers

While triggers vary, certain foods and beverages are reported to exacerbate symptoms in a significant percentage of IC/BPS patients:

| **Category** | **Common Examples** | **Approximate Percentage of Patients Affected** |
| --- | --- | --- |
| **Acidic foods/drinks** | Citrus fruits/juices, tomatoes, vinegar, cranberry juice | 60-70% |
| **Caffeine** | Coffee, black tea, green tea, energy drinks, chocolate | 50-60% |
| **Alcohol** | Beer, wine, spirits | 50-60% |
| **Carbonated beverages** | Soda, sparkling water | 40-50% |
| **Artificial sweeteners** | Aspartame, saccharin, sucralose | 30-40% |
| **Spicy foods** | Chili peppers, hot sauce, curry | 30-40% |
| **Preservatives/additives** | MSG, sulfites, nitrates/nitrites | 20-30% |
| **High-histamine foods** | Aged cheeses, fermented foods, smoked meats | 15-25% |

The Correct Approach to IC/BPS Diet Management

The gold standard for identifying personal triggers is the **elimination and reintroduction diet**:

**Elimination phase (2-4 weeks)**: Remove all common trigger foods from your diet

**Symptom tracking**: Keep a detailed food diary recording everything you eat/drink and your symptom severity

**Reintroduction phase**: Add one food back at a time, waiting 2-3 days between each to monitor for symptom flares

**Personalization**: Create a diet that only avoids foods that consistently trigger your symptoms

Important Considerations

**Some patients have no dietary triggers at all** (approximately 10-15% of IC/BPS cases)

Sensitivity to specific foods can **change over time**

**Moderation is key**: Many patients can tolerate small amounts of trigger foods without experiencing symptoms

Severe dietary restriction can lead to **nutritional deficiencies** and should be supervised by a healthcare provider or registered dietitian

The goal of dietary management is to improve quality of life, not to achieve perfect symptom control through extreme restriction

Disclaimer

This information is provided for educational purposes only and is not a substitute for professional medical advice, diagnosis, or treatment. Always seek the advice of your physician or other qualified health provider with any questions you may have regarding a medical condition.

**30. How can someone with interstitial cystitis find out which foods bother their bladder?**

Identifying Bladder Irritant Foods for Interstitial Cystitis (IC)

Diet is one of the most impactful modifiable factors for managing interstitial cystitis (also called bladder pain syndrome), as approximately 70-90% of people with IC experience symptom flares from specific foods and beverages . Triggers vary dramatically between individuals, so personalized testing is essential. Below is a step-by-step, evidence-based approach to identifying your unique bladder irritants.

1. Start with a Comprehensive Food & Symptom Diary

This is the foundation of trigger identification, as it establishes clear connections between what you consume and how your bladder feels.

**What to record daily:**

All foods, beverages, supplements, and medications (including over-the-counter products) with exact portions and times

Symptom severity (use a 0-10 scale for pain, urgency, and frequency)

Symptom onset time (reactions can occur immediately or be delayed up to 48 hours)

Other potential factors: stress levels, physical activity, sexual activity, and sleep quality

**Pro tip:** Use a dedicated app (such as the *IC Diet Tracker* or *MyFitnessPal* with custom symptom logging) for easier tracking and pattern recognition. Continue the diary throughout the entire elimination and reintroduction process.

2. The Elimination Diet Phase (2-4 Weeks)

The goal is to remove all known common IC triggers from your diet until your symptoms significantly improve or resolve completely. This baseline allows you to clearly detect reactions during reintroduction.

Standard IC Elimination Diet Guidelines

Eliminate all of the following categories:

**Acidic foods & beverages:** Citrus fruits (oranges, lemons, limes, grapefruits), tomatoes, pineapple, cranberries, vinegar, and pickled foods

**Caffeine & stimulants:** Coffee (regular and decaf), black tea, green tea, energy drinks, chocolate, and caffeinated soda

**Alcohol:** All types, including wine, beer, and spirits

**Artificial sweeteners:** Aspartame, saccharin, sucralose, and stevia (found in diet drinks, sugar-free gum, candy, and many processed foods)

**Spicy foods:** Chili peppers, hot sauce, curry, salsa, and foods with black pepper in large quantities

**Processed foods:** Those containing preservatives (sulfites, nitrates), monosodium glutamate (MSG), and artificial flavors/colors

**Important notes:**

Decaffeinated coffee still contains small amounts of caffeine and acidic compounds that can irritate the bladder

Many "healthy" foods (like cranberries, which are recommended for UTIs) are highly acidic and problematic for IC

Even trace amounts of triggers can cause reactions in sensitive individuals

3. The Systematic Reintroduction Phase

Once your symptoms have been stable or improved for at least 3-5 days, begin reintroducing one food category at a time. This is the most critical step for accurate trigger identification.

**Reintroduction protocol:**

Choose one food from the eliminated list (e.g., tomatoes)

Eat a normal portion of that food (not an excessive amount)

Do not introduce any other new foods for the next 48 hours

Monitor your symptoms closely using your diary

If no reaction occurs after 48 hours, that food is likely safe for you

If a reaction occurs, eliminate that food again and wait until your symptoms return to baseline before reintroducing the next food

Repeat this process for all eliminated foods

**Key considerations:**

Reintroduce only one food at a time to avoid confusion about which one caused the reaction

Some people have delayed reactions that appear 24-48 hours after consumption

A food that causes a mild reaction may be tolerable in smaller portions or when prepared differently (e.g., cooked tomatoes vs. raw tomatoes)

4. Common Less Obvious Triggers

Many people with IC are surprised by these less widely recognized irritants:

**Dairy products:** Especially aged cheeses, yogurt, and sour cream

**Nuts & seeds:** Almonds, walnuts, peanuts, and sunflower seeds

**Certain fruits & vegetables:** Bananas, avocados, onions, garlic, and spinach

**Meat & fish:** Processed meats (bacon, sausage), smoked fish, and canned tuna

**Beverages:** Carbonated water (even unflavored), herbal teas (especially chamomile and peppermint), and fruit juices

**Water additives:** Chlorine and fluoride in tap water (try filtered or bottled spring water if you suspect this)

5. Practical Tips for Success

**Read labels carefully:** Hidden triggers are common in processed foods. Look for terms like "natural flavors," "spices," "hydrolyzed protein," and "caramel color," which can contain MSG or other irritants.

**Cook at home:** This gives you complete control over ingredients and preparation methods.

**Try modifying preparation:** Cooking acidic foods can reduce their acidity. Peeling fruits and vegetables may also make them more tolerable.

**Stay hydrated:** Drink plenty of plain water throughout the day. Dehydration concentrates urine, which can increase bladder irritation even without specific triggers.

**Be patient:** The entire process can take 2-3 months. It's normal to experience setbacks, but the information you gain will be invaluable for long-term symptom management.

6. When to Seek Professional Help

If your symptoms do not improve after 4 weeks of strict elimination

If you are having difficulty maintaining a balanced diet while avoiding triggers

If you have significant weight loss or nutritional concerns

If your symptoms are severe or interfering with your daily life

A registered dietitian with experience in IC can provide personalized guidance, help you design a nutritionally adequate diet, and troubleshoot difficult cases. Your urologist or pelvic pain specialist can also rule out other conditions that may be causing your symptoms.

**Disclaimer:** This information is for educational purposes only and is not a substitute for professional medical advice, diagnosis, or treatment. Always seek the advice of your physician or other qualified health provider with any questions you may have regarding a medical condition.

**31. Should people with interstitial cystitis drink less water to urinate less often?**

**No, people with interstitial cystitis (IC) should NOT drink less water to reduce urination frequency.**

Reasons

Less water makes urine highly concentrated, which strongly irritates the inflamed and sensitive bladder lining, worsening pelvic pain, urinary urgency and bladder spasms.

Dehydration increases the risk of secondary urinary tract infections, which will seriously aggravate IC symptoms.

Cutting water intake only relieves frequent urination temporarily, but causes longer and more severe symptom flares.

Correct water-drinking rules

Drink **moderate and steady water** evenly all day, avoid binge drinking.

Daily intake: about 1500–2000 ml, adjust according to personal comfort.

Strictly avoid irritating drinks: coffee, alcohol, carbonated drinks, citrus juice, strong tea and acidic beverages.

Urinate regularly and do not hold urine for a long time.

**Disclaimer**: This information is for popular science reference only, not a substitute for professional medical diagnosis and treatment. Please follow the guidance of a professional urologist for personalized daily management.

**32. Can emotional stress make interstitial cystitis symptoms worse?**

Answer

**Yes, emotional stress definitely worsens interstitial cystitis (IC) symptoms.**

Main reasons

Stress raises stress hormones, aggravating chronic inflammation and nerve sensitivity in the bladder wall.

It causes involuntary pelvic floor muscle spasm, triggering pelvic pain, bladder pressure and burning pain.

Emotional tension speeds up bladder contraction, leading to more frequent urination and urgent urination.

Negative emotions amplify physical pain perception, making discomfort feel far more severe.

Clinical fact

Stress, anxiety, depression and long-term mental fatigue are the most common daily flare-up triggers for IC patients. Stress relief is an essential part of IC daily management.

**Disclaimer**: This information is for educational reference only, not medical diagnosis or treatment guidance. Please seek professional advice from a urologist.

**33. What daily habits may help reduce interstitial cystitis flare-ups?**

Daily Habits to Reduce Interstitial Cystitis (IC) Flare-Ups

**Disclaimer**: This information is for educational purposes only and is not a substitute for professional medical advice, diagnosis, or treatment. Interstitial cystitis is a complex, heterogeneous condition, and triggers vary significantly between individuals. Always consult with a urologist or pelvic floor specialist before making significant changes to your treatment plan.

1. Diet Modification (Strongest Evidence Base)

Diet is the most well-established modifiable factor for IC flare-ups, as many foods and beverages irritate the bladder lining by altering urine pH or containing direct irritants.

**Systematic elimination and reintroduction**: Remove all common trigger foods for 2-4 weeks, then reintroduce one at a time (waiting 3-5 days between each) to identify personal sensitivities.

**Avoid universal high-risk triggers**:

Caffeine (coffee, tea, energy drinks, chocolate)

Alcohol (all types)

Carbonated beverages

Artificial sweeteners (aspartame, saccharin, sucralose)

Acidic foods (citrus fruits, tomatoes, cranberries, pineapple)

Spicy foods

**Hydrate strategically**: Sip 6-8 cups of water throughout the day rather than drinking large amounts at once. Dehydration concentrates urine (increasing irritation), while overhydration worsens frequency.

**Consider neutralizing urine**: Some people find relief with slightly alkaline water (pH 7.5-8.5) or small amounts of baking soda (1/4 teaspoon in water, consult your doctor first).

2. Bladder Training and Voiding Habits

**Bladder retraining**: Gradually increase intervals between voids by 15 minutes weekly, starting from your current baseline. This expands functional bladder capacity and reduces urgency signals.

**Double voiding**: After urinating, wait 30 seconds, lean forward slightly, and try again to empty completely. Residual urine increases irritation and infection risk.

**Avoid "just-in-case" urination**: Urinating too frequently trains the bladder to hold less urine over time.

**Maintain proper posture**: Sit upright on the toilet, relax your pelvic floor, and avoid straining or rushing.

3. Stress and Pelvic Floor Management

Stress is a major trigger for IC flare-ups, as it increases pelvic floor muscle tension and systemic inflammation.

**Daily relaxation practices**: Spend 10-15 minutes twice daily on deep breathing, progressive muscle relaxation, or mindfulness meditation specifically focusing on the pelvic area.

**Pelvic floor physical therapy**: Work with a specialized therapist to learn to relax tight pelvic floor muscles (a common underlying issue in IC) rather than just strengthening them.

**Gentle movement**: Engage in low-impact activities like walking, swimming, or restorative yoga. Avoid high-impact exercises (running, jumping) that jar the bladder.

4. Sleep Hygiene

Poor sleep amplifies pain perception and stress, creating a vicious cycle with IC symptoms.

**Establish a consistent schedule**: Go to bed and wake up at the same time daily, even on weekends.

**Limit fluids before bed**: Reduce intake 1-2 hours before bedtime to minimize nighttime awakenings.

**Create a sleep-conducive environment**: Keep your bedroom cool, dark, and quiet. Avoid screens for at least 1 hour before bed.

5. Clothing and Personal Care

**Wear loose-fitting clothing**: Avoid tight pants, leggings, or shapewear that compress the bladder and pelvic area.

**Choose breathable fabrics**: Opt for cotton underwear and avoid synthetic materials that trap moisture.

**Use unscented products**: Avoid scented soaps, bubble baths, lotions, feminine hygiene products, and laundry detergents, as fragrances are common urethral irritants.

6. Sexual Health Practices

**Urinate before and after sexual activity**: This flushes bacteria and reduces bladder pressure.

**Use water-based lubricants**: Avoid oil-based or scented lubricants that can cause irritation.

**Experiment with positions**: Find positions that minimize pressure on the bladder.

**Communicate openly with your partner** about your symptoms and comfort levels.

7. Self-Monitoring and Consistency

**Keep a detailed symptom journal**: Track daily symptoms, food/beverage intake, stress levels, activities, and sleep quality. This is the most effective way to identify your unique triggers.

**Be patient and consistent**: It may take 3-6 months of consistent habit changes to see significant improvements in IC symptoms.

**Avoid extreme changes**: Gradual modifications are more sustainable and less likely to trigger unexpected flare-ups.

**34. Can interstitial cystitis be cured completely?**

**Can Interstitial Cystitis/Bladder Pain Syndrome (IC/BPS) Be Cured Completely?**

As of current medical consensus (2026), **there is no definitive cure for interstitial cystitis/bladder pain syndrome (IC/BPS)**. It is recognized as a chronic, complex, and heterogeneous condition whose exact underlying causes remain incompletely understood.

What This Actually Means for Patients

The absence of a cure does not mean that symptoms cannot be effectively managed or that patients must live with unrelenting discomfort. The primary goal of modern IC/BPS treatment is not eradication of the disease, but rather:

Significant reduction or elimination of pelvic pain, urinary frequency, urgency, and nocturia

Restoration of normal daily activities and bladder function

Substantial improvement in overall quality of life

Prevention of symptom flares and disease progression

Many patients with IC/BPS can achieve **long-term clinical remission** (periods of little to no symptoms) with appropriate, personalized treatment plans. Some individuals may experience complete resolution of symptoms for months or even years, though the condition may still have the potential to flare up under certain triggers (such as stress, specific foods, or infections).

Why a Cure Remains Elusive

IC/BPS is not a single disease entity but likely a spectrum of conditions with multiple overlapping contributing factors, including:

Defects in the bladder's protective glycosaminoglycan (GAG) layer

Neurogenic inflammation and central nervous system sensitization

Immune system dysregulation

Genetic predisposition

Possible infectious or autoimmune components

Pelvic floor muscle dysfunction

Because the root cause varies significantly between patients, a universal one-size-fits-all cure is currently not feasible. Treatment must be tailored to each individual's specific symptoms and underlying pathophysiological mechanisms.

Prognosis and Long-Term Outlook

The natural course of IC/BPS varies widely among individuals:

Approximately 50% of patients experience spontaneous improvement over time

Many have intermittent flares separated by long periods of remission

A smaller percentage (about 10-15%) have persistent, severe symptoms that require ongoing, aggressive management

Early diagnosis and initiation of treatment are strongly associated with better long-term outcomes. A multimodal approach combining lifestyle modifications, dietary changes, pelvic floor physical therapy, medications, and in some cases, minimally invasive procedures, offers the best chance for sustained symptom control.

**Disclaimer:** This information is provided for educational purposes only and is not a substitute for professional medical advice, diagnosis, or treatment. Always seek the advice of your physician or other qualified health provider with any questions you may have regarding a medical condition.

**35. What is the main goal of interstitial cystitis treatment?**

Main Goals of Interstitial Cystitis/Bladder Pain Syndrome (IC/BPS) Treatment

Interstitial cystitis (IC), now formally called **bladder pain syndrome (BPS)** in many clinical guidelines, is a chronic, idiopathic condition of the lower urinary tract. **There is currently no cure for IC/BPS**, so treatment is not aimed at eradicating the disease but at comprehensive, patient-centered management. The primary goals, as outlined by the American Urological Association (AUA) and International Continence Society (ICS) guidelines, are as follows:

1. Relieve Core Clinical Symptoms

This is the immediate and most fundamental goal of treatment, targeting the hallmark manifestations of IC/BPS:

**Pain control**: Alleviate chronic pelvic pain, suprapubic pressure/discomfort, bladder pain that worsens with bladder filling and improves with emptying, and referred pain to the urethra, vagina, rectum, or lower back

**Reduce lower urinary tract symptoms (LUTS)**: Decrease urinary frequency (both daytime and nighttime), urinary urgency, and urgency incontinence

2. Significantly Improve Health-Related Quality of Life (HRQoL)

IC/BPS profoundly impacts multiple domains of daily functioning, making quality of life improvement the ultimate benchmark of treatment success:

Restore normal sleep patterns by reducing nocturia

Enable return to work, school, and daily activities

Preserve social relationships and participation in recreational activities

Improve sexual function and intimacy

Reduce the psychological burden of living with a chronic, unpredictable condition

3. Preserve and Protect Bladder Function

Long-term IC/BPS can lead to irreversible structural changes in the bladder wall. A key treatment goal is to prevent these progressive changes:

Maintain normal bladder capacity

Prevent bladder wall fibrosis, inflammation, and ulceration (including Hunner's lesions)

Avoid bladder contracture and reduced compliance, which would require more invasive interventions

4. Prevent and Manage Associated Complications

IC/BPS is frequently associated with comorbid conditions that can exacerbate symptoms and further reduce quality of life:

Address psychological comorbidities (depression, anxiety, chronic pain catastrophizing)

Treat secondary pelvic floor dysfunction (muscle tension, spasms)

Manage overlapping chronic pain syndromes (fibromyalgia, chronic fatigue syndrome, irritable bowel syndrome)

Additional Considerations

Treatment is highly individualized, as symptoms and their severity vary dramatically between patients. The approach typically involves a stepwise, multimodal strategy combining lifestyle modifications, oral medications, intravesical therapies, physical therapy, and psychological support. The long-term goal is to achieve and maintain symptom remission while minimizing treatment-related side effects.

**Disclaimer**: This information is provided for educational purposes only and is not a substitute for professional medical advice, diagnosis, or treatment. Always seek the advice of your physician or other qualified health provider with any questions you may have regarding a medical condition.

**36.How long can interstitial cystitis treatment take to start working?**

How Long Does Interstitial Cystitis (IC/BPS) Treatment Take to Work?

Interstitial cystitis/bladder pain syndrome (IC/BPS) is a chronic, complex condition with no cure, and treatment response varies dramatically between individuals. **Most treatments take weeks to months to produce meaningful symptom relief**, and many require ongoing use to maintain benefits. Below is a detailed breakdown of typical onset times for common evidence-based treatments:

1. First-Line Treatments (Lifestyle & Behavioral Modifications)

These are universally recommended first steps and often provide the earliest relief:

**Dietary changes**: Some patients notice reduced bladder irritation within **3-7 days** of eliminating common triggers (caffeine, alcohol, acidic foods, artificial sweeteners). However, full dietary optimization and identifying personal triggers typically takes **4-12 weeks** of careful elimination and reintroduction.

**Fluid management**: Adjusting intake volume and timing can improve frequency/urgency within **1-2 weeks**.

**Bladder training**: Gradual improvements in bladder capacity and urgency control usually begin at **2-4 weeks**, with maximum benefit achieved after **8-12 weeks** of consistent practice.

**Stress management**: Mindfulness, meditation, and relaxation techniques may reduce pain exacerbations within **2-4 weeks**, but long-term practice is required for sustained benefit.

2. Oral Medications

| **Medication Class** | **Typical Onset of Action** | **Time to Maximum Benefit** | **Notes** |
| --- | --- | --- | --- |
| **Pentosan polysulfate sodium (Elmiron)** | 4-8 weeks | 3-6 months | The only FDA-approved oral drug for IC. Slow onset due to gradual bladder lining repair. Approximately 30-40% of patients experience meaningful relief. |
| **Antihistamines (hydroxyzine)** | 2-4 weeks | 6-8 weeks | Most effective for patients with allergies or mast cell activation. Works by reducing bladder inflammation. |
| **Tricyclic antidepressants (amitriptyline)** | 2-4 weeks | 6-12 weeks | Relieves pain and urgency by modulating nerve signals. Low starting doses are gradually increased to minimize side effects. |
| **Antispasmodics (oxybutynin, tolterodine)** | 1-2 weeks | 4-6 weeks | Primarily improves urgency and frequency, less effective for pain. |
| **NSAIDs/analgesics** | 30-60 minutes (acute) | N/A | Only provide temporary symptomatic relief, do not treat underlying inflammation. |

3. Bladder Instillations

These treatments deliver medication directly to the bladder lining and often work faster than oral medications:

**Heparin/lidocaine/bicarbonate "cocktail"**: Many patients experience temporary pain relief within **24-48 hours** of the first instillation. A full course (weekly for 6-8 weeks) typically produces sustained improvement by **6-12 weeks**.

**Dimethyl sulfoxide (DMSO)**: Onset of action is **2-4 weeks**, with maximum benefit after **6-8 weekly treatments**. Approximately 50% of patients respond.

**Botulinum toxin type A (Botox)**: Significant improvement in pain and urgency usually occurs within **1-2 weeks**, with peak effect at **4-6 weeks**. Benefits last approximately 6-9 months before repeat injections are needed.

4. Pelvic Floor Physical Therapy (PFPT)

PFPT is highly effective for IC patients with pelvic floor dysfunction (present in up to 70% of cases):

Initial improvements in muscle tension and pain may be noticed after **2-4 sessions** (2-4 weeks).

Maximum benefit typically requires **12-16 weeks** of weekly therapy, followed by a home maintenance program.

5. Second-Line & Invasive Treatments

**Neuromodulation (sacral nerve stimulation)**: Temporary trial stimulation may show benefit within **1-2 weeks**. Permanent implantation requires **3-6 months** for full nerve modulation effects.

**Hydrodistension**: Some patients experience immediate post-procedure relief, but benefits are often temporary (weeks to months). It is frequently used as a diagnostic procedure rather than a long-term treatment.

**Surgical interventions (cystectomy, urinary diversion)**: Reserved for severe, refractory cases. Recovery takes **3-6 months**, and symptom improvement may continue for up to a year.

Key Factors Affecting Treatment Response Time

**Disease duration and severity**: Patients with early, mild IC often respond faster than those with long-standing, severe disease.

**Individual variability**: Genetic factors, pain sensitivity, and comorbid conditions (fibromyalgia, IBS, anxiety) significantly impact response.

**Combination therapy**: Most patients require multiple treatments simultaneously for optimal results, which may accelerate overall improvement.

**Adherence**: Strict adherence to dietary restrictions, medication schedules, and therapy programs is critical for timely and sustained benefit.

**Trial-and-error process**: Finding the right treatment combination often takes **3-6 months** or longer, as no single treatment works for everyone.

Important Takeaways

**Be patient**: Most IC treatments do not provide immediate relief. It is common to try several approaches before finding what works for you.

**Set realistic expectations**: The goal of treatment is to reduce symptoms and improve quality of life, not to "cure" the condition.

**Communicate with your provider**: If you have not experienced any improvement after the expected timeframe, discuss adjusting your treatment plan.

**Disclaimer**: This information is for educational purposes only and is not a substitute for professional medical advice, diagnosis, or treatment. Always seek the advice of your physician or other qualified health provider with any questions you may have regarding a medical condition.

**37. Why do doctors often try interstitial cystitis treatments step by step?**

Why Doctors Use a Step-by-Step Approach for Interstitial Cystitis (IC/BPS) Treatment

Interstitial cystitis/bladder pain syndrome (IC/BPS) is a chronic, heterogeneous pelvic pain condition with no known cure. The step-by-step (tiered) treatment approach—endorsed by leading guidelines including the **American Urological Association (AUA) 2021 IC/BPS Guidelines**—is the standard of care for 9 core evidence-based reasons:

1. No Universal Cure or Single Effective Treatment

IC/BPS has multiple overlapping proposed causes, including:

Defects in the bladder's protective glycosaminoglycan (GAG) layer

Neurogenic inflammation and hypersensitivity

Pelvic floor muscle dysfunction

Autoimmune dysregulation

Mast cell activation

No single treatment targets all these mechanisms. The tiered approach systematically tests interventions with different modes of action to find what works for each individual patient.

2. Minimizing Risk and Side Effects ("Primum Non Nocere")

Treatments are ordered from **least invasive/lowest risk to most invasive/highest risk**, following the medical principle of "first, do no harm":

**Tier 1 (First-Line)**: Education, dietary modification, fluid management, stress reduction, bladder training, and pelvic floor physical therapy (virtually no side effects)

**Tier 2 (Second-Line)**: Oral medications (amitriptyline, hydroxyzine, pentosan polysulfate sodium) and bladder instillations (heparin, lidocaine, DMSO) (mild to moderate side effects)

**Tier 3 (Third-Line)**: Neuromodulation (sacral nerve stimulation, PTNS) and intravesical botulinum toxin A (moderate to significant risks: infection, pain at implant site)

**Tier 4 (Fourth-Line)**: Cystoscopy with hydrodistention under anesthesia, bladder augmentation, or urinary diversion (irreversible, major surgical risks)

This ensures patients avoid unnecessary harm from aggressive treatments if simpler options provide adequate relief.

3. Extreme Individual Variability in Response

IC/BPS symptoms and severity vary dramatically between patients:

Some achieve complete relief with dietary changes alone

Others require multiple combined treatments

A small subset (~5-10%) does not respond to any non-surgical intervention

The stepwise approach allows doctors to personalize care by adjusting treatments based on each patient's unique response.

4. Allowing Adequate Time for Treatment Effect

Many IC/BPS treatments have delayed onset of action:

Pentosan polysulfate sodium (Elmiron): 3-6 months of consistent use

Pelvic floor physical therapy: 4-8 weeks

Neuromodulation: 3-6 months for optimal results

The tiered system prevents premature abandonment of potentially effective treatments before they have had time to work.

5. Diagnostic Confirmation

IC/BPS is a **diagnosis of exclusion** (no definitive test exists). Response to initial treatments can help:

Confirm the IC/BPS diagnosis

Rule out conditions with similar symptoms (urinary tract infections, overactive bladder, endometriosis, prostate issues)

Identify dominant symptom drivers (e.g., pelvic floor dysfunction vs. bladder lining damage)

6. Combination Therapy Is Usually Required

Most patients with moderate to severe IC/BPS need multiple treatments to control symptoms. Adding interventions one by one allows doctors to:

Monitor for synergistic effects

Avoid harmful drug interactions

Identify which specific treatments are providing benefit

Minimize the total number of medications/procedures used

7. Patient-Centered Care and Improved Adherence

Starting with non-invasive, patient-managed treatments:

Empowers patients to take an active role in their care

Builds trust between patients and providers

Allows gradual adjustment to lifestyle changes

Improves long-term treatment adherence

8. Managing Fluctuating Symptoms

IC/BPS symptoms often wax and wane over time, with unpredictable flares. The stepwise approach enables:

Escalation of treatment during severe flares

De-escalation during periods of remission

Avoidance of unnecessary long-term exposure to medications or procedures

9. Avoiding Unnecessary Aggressive Interventions

Surgical treatments for IC/BPS are irreversible and carry significant risks, including incontinence, infection, and the need for additional surgery. The tiered system ensures that patients only undergo surgery when **all other treatment options have failed** and symptoms are severely impacting quality of life.

Important Note

The step-by-step approach is not rigid. Doctors may modify the order or combine tiers based on:

Symptom severity (e.g., a patient with debilitating pain may start with Tier 1 + Tier 2 treatments)

Patient preferences

Comorbid conditions (fibromyalgia, IBS, chronic fatigue syndrome)

Previous treatment history

The ultimate goal is to find the minimal effective treatment plan that maximizes quality of life while minimizing risks.

**Disclaimer**: This information is for educational purposes only and does not constitute medical advice. Always consult a qualified healthcare provider for diagnosis and treatment of IC/BPS or any other medical condition.

**38. Is it safe to take over-the-counter pain medicine for interstitial cystitis?**

Safety of Over-the-Counter Pain Medicines for Interstitial Cystitis (IC/BPS)

Over-the-counter (OTC) pain relievers can provide temporary symptom relief for some people with interstitial cystitis/bladder pain syndrome (IC/BPS), but **safety and effectiveness vary widely by medication type and individual patient**. No OTC medication cures IC, and some may actually worsen bladder irritation in certain people. Always consult your healthcare provider before starting any new medication, especially for long-term use.

Generally Safe (When Used Exactly as Directed)

1. Acetaminophen (Paracetamol)

**Effectiveness**: Moderately effective for mild-to-moderate IC-related pelvic pain and discomfort. It works by reducing pain signals in the brain, not by reducing inflammation.

**Safety profile**:

Short-term use at recommended doses is generally well-tolerated by most IC patients and does not typically irritate the bladder lining.

**Critical risk**: Severe liver damage with overdose (do not exceed 3,000-4,000 mg per day for adults, lower for people with liver disease or who drink alcohol regularly).

Long-term daily use (over 3 months) may be associated with increased kidney and cardiovascular risks, requiring medical supervision.

2. Phenazopyridine Hydrochloride

**Effectiveness**: Specifically targets urinary tract pain, burning, and urgency by numbing the bladder and urethral lining. It provides rapid (within 30 minutes) but short-term relief (4-6 hours).

**Safety profile**:

Approved for short-term use only (**maximum 2 days** without medical supervision).

**Important warnings**:

Turns urine bright orange/red and may stain clothing or contact lenses.

Long-term use can cause serious side effects including hemolytic anemia, kidney damage, and liver toxicity.

Masks symptoms of urinary tract infections (UTIs), which can be dangerous if left untreated.

Avoid if you have kidney disease, glucose-6-phosphate dehydrogenase (G6PD) deficiency, or liver disease.

Use With Extreme Caution (May Worsen IC Symptoms)

Nonsteroidal Anti-Inflammatory Drugs (NSAIDs)

Includes ibuprofen, naproxen sodium, and aspirin.

**Effectiveness**: May help some patients by reducing inflammation, but results are highly variable.

**Significant risks for IC patients**:

**Bladder irritation**: Many IC patients report that NSAIDs worsen their bladder pain, frequency, and urgency by irritating the already sensitive bladder lining.

Gastrointestinal issues: Stomach ulcers, bleeding, and indigestion, especially with long-term use.

Kidney damage: Increased risk in people with pre-existing kidney problems or who use NSAIDs regularly.

Cardiovascular risks: Higher risk of heart attack and stroke with long-term, high-dose use.

**Recommendation**: Test a single low dose first to see how your bladder reacts. Do not use regularly without your doctor's approval.

OTC Medications to Generally Avoid for IC

**Combination pain products**: Many contain caffeine, decongestants (pseudoephedrine), or antihistamines (diphenhydramine) that are common bladder irritants and can worsen IC symptoms.

**High-dose aspirin**: Increases bleeding risk and is more likely to irritate the bladder than acetaminophen.

**Certain herbal supplements**: Some OTC "bladder support" products contain ingredients like cranberry extract, which can irritate the bladder in many IC patients.

Critical Safety Guidelines

**Never exceed recommended dosages** or use medications longer than directed.

**Check all ingredients** in combination products for bladder irritants.

**Do not mix** different pain relievers without your doctor's approval.

**Track your symptoms**: If an OTC medication worsens your IC, stop using it immediately and inform your healthcare provider.

**Long-term use requires medical monitoring**: Regular use of any pain medication for IC should be supervised by a urologist or IC specialist to assess risks and benefits.

**Seek emergency care** if you experience severe pain, inability to urinate, fever, or blood in your urine.

Disclaimer

This information is for educational purposes only and does not constitute medical advice. Interstitial cystitis is a complex, individualized condition. Always consult your healthcare provider before taking any medication, especially if you have other health conditions, are pregnant or breastfeeding, or are taking other prescription drugs.

**39. Are antibiotics helpful for interstitial cystitis when there is no infection?**

Are Antibiotics Helpful for Interstitial Cystitis When There Is No Infection?

**No, antibiotics are generally not helpful and are specifically not recommended for interstitial cystitis/bladder pain syndrome (IC/BPS) when there is no proven bacterial infection.** This is a core recommendation from the 2022 American Urological Association (AUA) guidelines for IC/BPS management .

Why Antibiotics Do Not Work for Non-Infectious IC/BPS

IC/BPS is a chronic, non-infectious inflammatory bladder condition characterized by pelvic pain, urinary urgency, and frequency. Its exact cause remains unknown, but it is definitively not caused by bacteria . Antibiotics work by killing bacteria, so they cannot address the underlying mechanisms of IC/BPS, which include:

Defects in the bladder's protective glycosaminoglycan (GAG) layer

Mast cell activation and release of inflammatory mediators

Neurogenic inflammation and hypersensitivity

Possible autoimmune factors

The Common Misdiagnosis Problem

IC/BPS is frequently misdiagnosed as recurrent urinary tract infections (UTIs) because their symptoms overlap significantly. Studies show that 70% of IC/BPS patients experience diagnostic delays, often due to doctors confusing IC symptoms with UTIs . This leads to repeated courses of antibiotics that almost always fail to improve IC symptoms and may even worsen them .

Controversial Area: Occult Infections and Biofilms

There is ongoing research into whether a small subset of patients with "culture-negative" IC/BPS might have hidden infections that standard urine cultures cannot detect, such as:

Biofilms (bacterial communities encased in a protective matrix)

Intracellular bacterial communities

Low-virulence organisms

However:

This has not been proven as a primary cause of IC/BPS

There is no high-quality evidence from randomized controlled trials that long-term or repeated antibiotic courses improve symptoms in these patients

Biofilms are 10-1,000 times more resistant to antibiotics than free-floating bacteria

Advanced DNA testing for these organisms is not standard of care, and the benefits of targeted antibiotic therapy based on these tests remain unproven

Significant Risks of Unnecessary Antibiotic Use in IC/BPS

Using antibiotics when there is no infection poses serious risks for IC/BPS patients:

**Antibiotic resistance**: 80% of urine cultures from IC/BPS patients show resistant bacteria due to repeated antibiotic exposure

**Microbiome disruption**: Antibiotics kill beneficial bacteria in the gut, urinary tract, and vagina, leading to:

Clostridium difficile (C. diff) infection (severe diarrhea and colitis)

Fungal overgrowth (candida/yeast infections), which the MAPP Research Network found is common in IC/BPS patients experiencing flares

Worsening of IC/BPS symptoms themselves, as the urinary microbiome may play a role in bladder health

**Serious adverse effects**: Fluoroquinolone antibiotics (ciprofloxacin, levofloxacin) carry black box warnings for tendon rupture, aortic aneurysm, and permanent nerve damage

**Direct bladder irritation**: Some antibiotics can trigger IC/BPS flares

Important Exception

Prophylactic antibiotics may be appropriate for IC/BPS patients who also have confirmed recurrent UTIs, to prevent infections that can trigger IC flares. This is different from using antibiotics to treat the IC itself .

Recommended Treatments for IC/BPS

The AUA recommends a stepwise approach to IC/BPS treatment:

**First-line**: Patient education, dietary modifications (avoiding trigger foods), bladder training, pelvic floor physical therapy, stress management

**Second-line**: Oral medications (amitriptyline, cimetidine, hydroxyzine, pentosan polysulfate sodium), intravesical instillations (DMSO, heparin, lidocaine)

**Third-line**: Cystoscopy with short-duration, low-pressure hydrodistension, cyclosporine A (for Hunner lesions)

**Fourth-line**: Botulinum toxin A injections, neuromodulation

**Disclaimer**: This information is for educational purposes only and is not a substitute for professional medical advice, diagnosis, or treatment. Always seek the advice of your physician or other qualified health provider with any questions you may have regarding a medical condition.

**40. Should someone keep taking antibiotics for interstitial cystitis if urine tests show no infection?**

Definitive Answer

**No, you should NOT continue taking antibiotics for interstitial cystitis/bladder pain syndrome (IC/BPS) if repeated, properly collected urine tests show no evidence of a bacterial infection.** This is a Grade A recommendation from the American Urological Association (AUA) 2022 IC/BPS Clinical Guidelines and is endorsed by the International Continence Society (ICS) and European Association of Urology (EAU).

Critical Background

Interstitial cystitis is **not an infectious disease**. It is a chronic, inflammatory condition characterized by damage to the bladder's protective glycosaminoglycan (GAG) layer, nerve hypersensitivity, and pelvic floor dysfunction. The hallmark symptoms (pelvic pain, urinary frequency, urgency, nocturia) mimic urinary tract infections (UTIs), but they arise from bladder lining injury and neurogenic inflammation—not bacteria.

Why Antibiotics Fail for Non-Infectious IC

**No therapeutic benefit**: Multiple randomized controlled trials (RCTs) have consistently shown that antibiotics provide no statistically significant improvement in IC symptoms compared to placebo when no infection is present. Long-term antibiotic courses (≥4 weeks) have not demonstrated sustained benefit in any high-quality study.

**Antibiotics can worsen IC symptoms**:

They disrupt the **urinary and gut microbiomes**, which play a critical role in maintaining bladder health and immune function

Microbiome disruption can further damage the bladder's protective barrier and increase inflammation

Many antibiotics cause direct bladder irritation as a side effect

**Serious risks of unnecessary antibiotics**:

Development of antibiotic-resistant bacteria (a global public health crisis)

Clostridioides difficile (C. diff) infection, which can be life-threatening

Allergic reactions, ranging from mild rashes to anaphylaxis

Gastrointestinal side effects (nausea, diarrhea, abdominal pain)

Vaginal yeast infections in women

When Antibiotics *Are* Appropriate for IC Patients

Antibiotics should only be prescribed if you have a **confirmed bacterial UTI**, defined as:

A positive urine culture showing ≥10^5 colony-forming units (CFU/mL) of a known uropathogen

Corresponding UTI symptoms (new onset or worsening of baseline IC symptoms)

Ideally, antibiotic susceptibility testing to guide appropriate drug selection

Even in these cases, antibiotics should be used for the shortest effective course (typically 3-7 days for uncomplicated UTIs) and discontinued once the infection is cleared.

Evidence-Based IC Treatments to Consider Instead

The AUA recommends the following first-line treatments for IC/BPS:

**Behavioral and lifestyle modifications**:

Bladder training and timed voiding

Dietary modification (avoiding known trigger foods/drinks)

Stress management

Pelvic floor physical therapy (for patients with pelvic floor dysfunction)

**Oral medications**:

Amitriptyline (tricyclic antidepressant)

Hydroxyzine (antihistamine)

Pentosan polysulfate sodium (PPS, Elmiron)—the only FDA-approved oral medication for IC

**Intravesical therapies**:

Dimethyl sulfoxide (DMSO)

Heparin

Lidocaine

Combination therapies

Important Disclaimer

This information is for educational purposes only and does not constitute medical advice. Always consult a urologist who specializes in IC/BPS before stopping or changing any medications. Many patients with IC are misdiagnosed with recurrent UTIs for years, so working with a specialist is critical for proper management.

If you are currently taking antibiotics for IC with negative urine cultures, discuss tapering off them with your healthcare provider and transitioning to evidence-based IC treatments.

**41. Why might eye checks be needed for some interstitial cystitis medicines?**

Why Eye Checks Are Required for Some Interstitial Cystitis (IC) Medications

Most IC medications do not require routine eye monitoring. However, **one first-line oral medication** carries a well-documented risk of serious, potentially irreversible retinal damage, making regular eye exams critical. Additional off-label medications may cause milder ocular symptoms that warrant attention.

1. Primary Reason: Pentosan Polysulfate Sodium (PPS, Brand Name Elmiron)

PPS is the **only FDA-approved oral medication specifically for IC**, designed to repair the damaged bladder lining (glycosaminoglycan layer). It is responsible for nearly all IC-related eye monitoring requirements.

Ocular Risk: Pigmentary Maculopathy

**Condition**: A progressive disorder affecting the macula (the central part of the retina responsible for sharp, central vision).

**Mechanism**: PPS is a heparin-like compound that can accumulate in the retinal pigment epithelium (RPE) cells over years of use, causing cellular damage and pigmentary changes.

**Symptoms**: Difficulty reading, blurred central vision, dark spots in the visual field, trouble adjusting to low light, and distorted vision.

**Risk Factors**: Long-term use (≥3 years) and high cumulative doses are the strongest predictors of damage. The FDA issued a **black box warning** for this risk in 2020.

Monitoring Guidelines

**Baseline exam**: A comprehensive dilated eye exam before starting PPS treatment.

**Regular follow-ups**: Every 6–12 months while on the medication.

**Post-discontinuation monitoring**: Eye exams should continue for several years after stopping PPS, as retinal damage can progress even after discontinuation.

2. Other IC Medications with Potential Ocular Effects (Less Common)

These off-label medications typically cause mild, reversible ocular symptoms that do not require routine monitoring but should be reported to a healthcare provider:

**Tricyclic antidepressants (amitriptyline, imipramine)**: May cause blurred vision, dry eyes, and increased intraocular pressure (rarely a concern in patients without glaucoma).

**Antihistamines (hydroxyzine)**: Can exacerbate dry eye symptoms.

**Immunosuppressants (mycophenolate, azathioprine)**: Rarely used for severe, refractory IC. These may carry a small risk of ocular side effects (e.g., cataracts, glaucoma) with long-term use, requiring periodic monitoring.

Key Takeaway

Eye checks for IC patients are almost exclusively driven by the risk of **pigmentary maculopathy from pentosan polysulfate sodium**. Early detection through regular exams is critical, as stopping the medication may prevent further vision loss, though existing damage is often irreversible. Patients should always discuss medication risks and monitoring schedules with both their urologist and ophthalmologist.

**Disclaimer**: This information is for educational purposes only and does not constitute medical advice. Always consult a qualified healthcare provider for personalized diagnosis and treatment.

**42. Should people with interstitial cystitis talk to a doctor before using supplements?**

**Yes, people with interstitial cystitis (IC) absolutely must consult a doctor—preferably a urologist or urogynecologist with specialized IC expertise—before starting any new supplement, vitamin, or herbal product.** This is not just a general recommendation but a critical safety precaution for several compelling reasons:

1. Many supplements are potent bladder irritants that trigger IC flares

IC is characterized by a damaged or dysfunctional bladder lining (urothelium) that is hypersensitive to even small amounts of certain substances. Common supplements that frequently worsen IC symptoms include:

**Vitamin C (ascorbic acid)**: Even low doses can increase bladder acidity and irritate the urothelium. Buffered vitamin C may be better tolerated by some, but still requires medical supervision.

**B vitamins (especially B6, B12, and folic acid)**: Known to cause bladder irritation in a significant percentage of IC patients.

**Cranberry supplements**: Traditionally used for urinary tract infections (UTIs), but their high acidity and proanthocyanidin content often trigger severe IC flares.

**Citrus extracts, herbal supplements (ginseng, saw palmetto, yohimbe)**, and products containing artificial sweeteners, colors, preservatives, or citric acid as fillers.

2. Risk of dangerous drug interactions

IC patients typically take multiple medications to manage their symptoms, including:

Oral medications (amitriptyline, hydroxyzine, pentosan polysulfate sodium)

Pain relievers (including opioids for severe cases)

Bladder instillation therapies

Antidepressants or anti-anxiety medications

Many supplements can interact dangerously with these drugs. For example:

St. John's Wort reduces the effectiveness of antidepressants, birth control, and blood thinners

Vitamin K interferes with anticoagulant medications

Sedative herbs (valerian root, kava) amplify the sedative effects of pain medications and antidepressants

3. The unregulated supplement industry poses unique risks

Unlike prescription drugs, supplements are not rigorously tested for safety, efficacy, or purity by regulatory agencies. This means:

Products may not contain the ingredients or dosages listed on the label

They may be contaminated with heavy metals, pesticides, or prescription drugs

There is no standardized manufacturing process, leading to inconsistent quality between batches

For IC patients with extreme sensitivity, even trace amounts of hidden irritants can cause debilitating flares.

4. Individual trigger variability

IC is a highly individualized condition. A supplement that helps one patient may cause severe symptoms in another. A doctor can help you:

Identify your personal triggers through elimination diets and symptom journals

Test supplements one at a time in controlled doses

Monitor for adverse reactions

5. Even potentially beneficial supplements require guidance

Some supplements have shown preliminary benefit for IC symptoms in small studies, including quercetin, glucosamine/chondroitin sulfate, marshmallow root, and omega-3 fatty acids. However:

They are not universally effective

Optimal dosages for IC have not been well-established

They may still cause side effects or interact with your medications

They should never replace evidence-based medical treatments

6. Risk of delaying proper diagnosis and treatment

Relying on supplements instead of seeking medical care can delay the diagnosis of IC or other underlying conditions that mimic IC symptoms (such as UTIs, bladder cancer, or endometriosis). Delayed treatment can lead to worsening symptoms, reduced quality of life, and more severe bladder damage over time.

Special considerations

This recommendation is even more critical for:

Pregnant or breastfeeding women

People with kidney or liver disease

Individuals taking blood thinners or immunosuppressant medications

Patients scheduled for surgery

**Disclaimer**: This information is for educational purposes only and does not constitute medical advice. Always consult a qualified healthcare provider before starting, stopping, or changing any medication or supplement regimen.

**43. Can pelvic floor physical therapy help interstitial cystitis?**

Can Pelvic Floor Physical Therapy Help Interstitial Cystitis?

**Yes, pelvic floor physical therapy (PFPT) is a first-line, evidence-based treatment for interstitial cystitis/bladder pain syndrome (IC/BPS)** and is strongly recommended by leading urological guidelines worldwide, including the American Urological Association (AUA) and the International Continence Society (ICS) .

Why It Works: The IC-Pelvic Floor Connection

Up to **85-90% of people with IC/BPS have underlying pelvic floor dysfunction (PFD)**, specifically **pelvic floor muscle overactivity (hypertonicity)** and myofascial pain syndrome . This creates a vicious cycle:

Bladder inflammation from IC irritates adjacent pelvic floor nerves

Pelvic floor muscles spasm involuntarily to "protect" the bladder

Chronic muscle tension compresses nerves, worsens bladder pain, and disrupts normal bladder function

Increased bladder sensitivity further triggers more muscle spasms

PFPT directly breaks this cycle by targeting the muscular and nervous system components of IC, rather than just the bladder lining itself.

Core PFPT Techniques for IC/BPS

Unlike PFPT for urinary incontinence (which focuses on muscle strengthening), IC-specific PFPT prioritizes **muscle relaxation, myofascial release, and neuromuscular re-education**:

**Internal and external myofascial release**: Gentle manual therapy to release tight trigger points in the pelvic floor muscles, vaginal wall (for women), perineum, and lower abdomen

**Biofeedback training**: Uses sensors to help patients visualize and learn to relax overactive pelvic floor muscles voluntarily

**Trigger point dry needling**: Thin needles inserted into tight muscle knots to reduce pain and spasm (often combined with manual therapy)

**Bladder retraining**: Gradually extends time between bathroom trips to reduce urinary frequency and urgency

**Diaphragmatic breathing and relaxation exercises**: Teaches patients to release pelvic floor tension during daily activities and flare-ups

**Posture correction**: Addresses poor posture that contributes to chronic pelvic floor muscle strain

Clinical Evidence of Effectiveness

AUA guidelines (2022) rate PFPT as a **Level A recommendation** (highest quality evidence) for IC/BPS patients with pelvic floor tenderness

Multiple randomized controlled trials show that **60-80% of patients experience significant symptom improvement** (≥30% reduction in pain, frequency, and urgency) after 6-12 weeks of weekly PFPT sessions

Benefits are often long-lasting: One study found that 70% of patients maintained improved symptoms at 12-month follow-up

PFPT is particularly effective for patients with:

Pelvic pain that worsens with sitting

Pain during sexual intercourse (dyspareunia)

Difficulty starting or stopping urination

No significant improvement from oral medications alone

Limitations and Considerations

**Not a cure**: PFPT manages symptoms but does not eliminate the underlying bladder inflammation of IC

**Requires commitment**: Patients typically need 8-12 weekly sessions plus daily home exercises

**Provider expertise matters**: Results are best with a physical therapist who has specialized training in pelvic floor dysfunction and IC/BPS

**Not effective for everyone**: Approximately 20-30% of patients have minimal improvement, often those with severe bladder wall lesions (Hunner's lesions) without significant pelvic floor involvement

Optimal Treatment Approach

PFPT works best as part of a multimodal treatment plan that may also include:

Diet modification (avoiding IC trigger foods)

Oral medications (e.g., pentosan polysulfate sodium, amitriptyline)

Bladder instillations

Stress management and cognitive behavioral therapy

**Disclaimer**: This information is for educational purposes only and does not constitute medical advice. Always consult a qualified healthcare provider for diagnosis and personalized treatment recommendations for interstitial cystitis.

**44. Are pelvic floor strengthening exercises always good for interstitial cystitis?**

**No, pelvic floor strengthening exercises (such as Kegels) are NOT always beneficial for interstitial cystitis/bladder pain syndrome (IC/BPS). In fact, they can significantly worsen symptoms for the majority of patients.**

Why Strengthening Is Often Harmful for IC/BPS

The key issue lies in the type of pelvic floor dysfunction most commonly associated with IC/BPS:

**Hypertonic (overactive/tight) pelvic floor dysfunction** affects approximately 70-85% of IC/BPS patients. These muscles are in a constant state of spasm or tension, unable to fully relax.

Strengthening exercises further contract and tighten these already overactive muscles, increasing pelvic pain, urinary urgency, frequency, and difficulty emptying the bladder.

Official Clinical Guidelines

The **2022 American Urological Association (AUA) Guidelines for IC/BPS** explicitly state:

Pelvic floor physical therapy (PFPT) is a first-line treatment for IC/BPS patients with pelvic floor tenderness or dysfunction

**PFPT for IC/BPS should focus on relaxation techniques, myofascial release, trigger point therapy, and biofeedback** – NOT strengthening

Routine pelvic floor strengthening is not recommended as a treatment for IC/BPS and may cause harm

When Strengthening Might Be Appropriate

Pelvic floor strengthening exercises may only be considered in very specific circumstances:

A patient has been **formally evaluated by a specialized pelvic floor physical therapist**

The evaluation confirms **hypotonic (weak) pelvic floor dysfunction** (a much less common presentation in IC/BPS)

Exercises are prescribed individually, started at very low intensity, and closely monitored

The patient stops immediately if symptoms worsen

Common Patient Mistake

Many IC/BPS patients mistakenly perform Kegel exercises because they experience urinary urgency or frequency. However, these symptoms in IC/BPS are typically caused by bladder irritation and pelvic floor tension, not muscle weakness.

Recommended Pelvic Floor Approaches for IC/BPS

Diaphragmatic breathing to relax the pelvic floor

Reverse Kegels (muscle relaxation exercises)

Internal and external myofascial release

Trigger point therapy

Biofeedback to learn proper muscle relaxation

**Disclaimer**: This information is for educational purposes only and does not constitute medical advice. Always consult a qualified healthcare provider or pelvic floor physical therapist before starting any exercise program for IC/BPS.

**45. Can bladder training help people with interstitial cystitis urinate less often?**

**Yes, bladder training can help many people with interstitial cystitis/bladder pain syndrome (IC/BPS) urinate less frequently**, and it is recommended as a first-line conservative treatment by major urological guidelines including the American Urological Association (AUA) 2022 guidelines and the Canadian Urological Association (CUA) 2025 guidelines (Grade B recommendation for motivated patients) .

How It Works for IC/BPS

People with IC/BPS often develop a habit of frequent voiding (sometimes every 30-60 minutes) to avoid or reduce bladder pain and urgency . Over time, this trains the bladder to signal the need to urinate at very low volumes, creating a vicious cycle where the bladder never gets to expand normally.

Bladder training breaks this cycle by:

Using **timed/scheduled voiding** (going to the bathroom according to a clock rather than waiting for the urge)

Gradually increasing the intervals between voids

Teaching techniques to manage urgency and mild discomfort between scheduled times (deep breathing, distraction, relaxation)

Potentially increasing functional bladder capacity over time

Evidence of Effectiveness

Studies show that consistent bladder training can significantly reduce daytime and nighttime urinary frequency in IC/BPS patients

A 12-week program combining timed voiding, controlled fluid intake, and pelvic floor relaxation increased time between voids and decreased frequency, with 88% of patients reporting overall symptom improvement

Research comparing hydrodistention alone versus hydrodistention plus bladder training found that the combination group had significantly lower urgency rates (10.71% vs 43.48%) and fewer daytime voids at 24 weeks follow-up

The urinary frequency and urgency components of IC/BPS respond better to bladder training than the pelvic pain component

How to Do Bladder Training Safely for IC/BPS

**Baseline assessment**: Keep a 3-7 day bladder diary to record your current voiding times and intervals

**Initial phase**: Start with scheduled voiding at your shortest comfortable interval (e.g., every 30-60 minutes), whether you feel the need to go or not

**Gradual extension**: Increase the interval by 10-15 minutes every 3-4 days only if you can do so without severe pain

**Urgency management**: When you feel an urge between scheduled times, use relaxation techniques or distraction rather than immediately going to the bathroom

**Target goal**: Most patients aim for 2-3 hour intervals between voids

Important Limitations and Precautions

**Do not hold urine to the point of severe pain** - this can trigger symptom flares and damage the bladder wall

Bladder training primarily improves frequency and urgency; it usually does not reduce bladder pain significantly

Results are highly variable - it works best for motivated patients with mild to moderate symptoms

It should be combined with other first-line treatments: dietary modifications (avoiding trigger foods), fluid management (1500-2000ml daily), stress reduction, and pelvic floor relaxation therapy (not Kegel exercises, which can worsen symptoms in IC/BPS)

It is most effective when used as part of a multimodal treatment approach tailored to your specific symptoms

**Disclaimer**: This information is for educational purposes only and is not a substitute for professional medical advice. Always consult your urologist or healthcare provider before starting any new treatment for interstitial cystitis.

**46. Can bladder medicine placed through a tube help interstitial cystitis symptoms?**

Bladder Instillation Therapy for Interstitial Cystitis (IC/BPS)

**Yes, bladder medications placed through a catheter (bladder instillation therapy) is a well-established, evidence-based treatment for interstitial cystitis/bladder pain syndrome (IC/BPS), particularly for patients who do not respond to oral medications.** It delivers medications directly to the bladder lining, bypassing systemic absorption and minimizing side effects while targeting the damaged urothelium (bladder wall) that causes IC symptoms.

How It Works

IC/BPS is characterized by a defective bladder lining (urothelial dysfunction), increased nerve sensitivity, and chronic inflammation. Instilled medications work locally to:

Repair the damaged glycosaminoglycan (GAG) layer of the bladder wall

Reduce inflammation and nerve hypersensitivity

Block pain signals

Decrease urinary frequency and urgency

Common Medications Used in Bladder Instillations

| **Medication Class** | **Examples** | **Evidence Strength** | **Typical Use Case** |
| --- | --- | --- | --- |
| **GAG layer replacement agents** | Heparin, sodium hyaluronate, chondroitin sulfate | **Strongest evidence** | First-line instillation therapy for most IC patients |
| **Dimethyl sulfoxide (DMSO)** | Rimso-50 | FDA-approved (1978) | Moderate-to-severe IC; often combined with other agents |
| **Local anesthetics** | Lidocaine (with or without bicarbonate) | Good evidence | Acute flare-ups, rapid pain relief |
| **Corticosteroids** | Triamcinolone, hydrocortisone | Moderate evidence | Severe inflammation, refractory cases |
| **Antibiotics** | Various | Limited evidence | Only if concurrent infection is confirmed |

Evidence for Effectiveness

**GAG replacement therapy**: Multiple randomized controlled trials (RCTs) show significant improvements in pain, frequency, urgency, and quality of life in 50-70% of patients. Sodium hyaluronate and chondroitin sulfate combinations have particularly strong data.

**DMSO**: The only FDA-approved intravesical agent for IC. Studies show symptom improvement in 50-60% of patients, though benefits may take 4-6 weeks to appear. It is often used in "cocktails" with heparin, lidocaine, and bicarbonate for enhanced effect.

**Lidocaine instillations**: Provide rapid (within 15-30 minutes) but temporary pain relief, making them ideal for acute flares. Long-term use with maintenance instillations can help prevent recurrences.

Procedure Details

A thin, flexible catheter is inserted through the urethra into the bladder

The medication solution is slowly instilled

The catheter is removed, and the patient retains the solution for 30-60 minutes (if possible)

Initial treatment course: Typically 1-2 times weekly for 4-6 weeks

Maintenance therapy: Once every 1-4 weeks for 3-6 months or longer, depending on response

Benefits

Direct delivery to the target organ

Lower systemic side effects compared to oral medications

Effective for patients who fail oral therapy

Can be self-administered at home after proper training

Provides rapid relief during acute flares (with lidocaine)

Risks and Limitations

**Common**: Temporary urethral irritation, urinary tract infection (UTI), bladder spasm, discomfort during catheterization

**Rare**: Bladder perforation, hematuria (blood in urine), allergic reaction to medications

**Limitations**: Not effective for all patients (30-50% may have minimal or no response); requires repeated treatments; benefits may diminish over time without maintenance

Who Should Consider This Treatment?

Bladder instillation therapy is recommended by the American Urological Association (AUA) guidelines as:

Second-line treatment for patients who do not respond to first-line therapies (behavioral modification, oral medications like pentosan polysulfate sodium, amitriptyline, or hydroxyzine)

First-line option for patients with severe symptoms who cannot tolerate oral medications

Rescue therapy for acute IC flares

Important Considerations

Response varies widely between individuals

Combination "cocktails" are often more effective than single-agent therapy

Self-administration at home is safe and cost-effective for long-term maintenance

It may take several weeks to experience full benefits

It should be used as part of a comprehensive treatment plan that includes dietary modifications, stress management, and pelvic floor physical therapy

**Disclaimer**: This information is for educational purposes only and is not a substitute for professional medical advice, diagnosis, or treatment. Always seek the advice of your physician or other qualified health provider with any questions you may have regarding a medical condition.

**47. Is major surgery usually needed for interstitial cystitis?**

**No, major surgery is almost never the first-line or standard treatment for interstitial cystitis (IC)/bladder pain syndrome (BPS)**. It is strictly reserved as a **last-resort option** only for patients with severe, refractory (treatment-resistant) disease who have failed *all* other available therapies and have severely impaired quality of life.

Standard Treatment Hierarchy (Non-Surgical First)

The American Urological Association (AUA) and International Continence Society (ICS) guidelines recommend a stepwise approach starting with the least invasive treatments:

First-Line Therapies (Most Patients Respond Here)

**Lifestyle and dietary modifications**: Identifying and avoiding trigger foods/drinks (caffeine, alcohol, acidic foods), stress management, and gentle hydration

**Pelvic floor physical therapy**: To address pelvic floor dysfunction, which is present in up to 70% of IC patients

**Oral medications**: Amitriptyline, pentosan polysulfate sodium (Elmiron), hydroxyzine, and pain relievers

Second-Line Therapies

**Bladder instillations**: Direct delivery of medications (DMSO, heparin, lidocaine combinations) into the bladder

**Intravesical botulinum toxin type A (Botox)**: For patients with urgency and frequency symptoms

Third-Line Therapies

**Neuromodulation**: Sacral nerve stimulation (InterStim) or percutaneous tibial nerve stimulation (PTNS) to regulate bladder nerve signals

Surgical Options (Last Resort Only)

Surgery is considered only when all non-surgical treatments have failed over an extended period (typically 6-12 months) and the patient has intractable pain, severely reduced bladder capacity, or is unable to tolerate any other therapies. The main surgical procedures are:

**Bladder hydrodistention**: A minor surgical procedure done under anesthesia to stretch the bladder wall. It is often used diagnostically and can provide temporary symptom relief for some patients.

**Augmentation cystoplasty**: Enlarging the bladder using a segment of the patient's own bowel. This procedure can increase bladder capacity but does not always eliminate pain.

**Cystectomy with urinary diversion**: The most radical surgery, involving complete removal of the bladder and creation of a new way for urine to exit the body. This is only considered for patients with end-stage IC who have exhausted all other options.

Important Considerations

Surgery does not cure IC. Some patients may still experience pelvic pain even after bladder removal.

All surgical procedures carry significant risks, including infection, bleeding, bowel complications, and urinary incontinence.

Less than 5-10% of IC patients will ever require major surgery.

**Disclaimer**: This information is for educational purposes only and is not a substitute for professional medical advice, diagnosis, or treatment. Always seek the advice of a qualified healthcare provider with any questions you may have regarding a medical condition.

**48. Can interstitial cystitis affect sexual life?**

Can Interstitial Cystitis Affect Sexual Life?

**Yes, interstitial cystitis (IC), also called bladder pain syndrome (BPS), significantly impacts sexual function and intimacy for most patients.** It affects approximately 3-8 million women and 1-4 million men in the United States alone, and sexual dysfunction is reported in 70-90% of patients with IC/BPS .

Primary Ways IC Impacts Sexual Life

1. Physical Pain During and After Intimacy

**Dyspareunia (painful intercourse)**: The most common sexual symptom. For women, pain typically occurs in the vulva, vagina, or deep pelvic region during penetration. For men, pain may occur in the penis, testicles, or perineum during or after ejaculation .

**Post-coital flare-ups**: Sexual activity often triggers severe IC symptom exacerbations that can last hours to days, including intense pelvic pain, increased urinary frequency (up to 60 times per day), and urgency .

**Pelvic floor muscle dysfunction**: Chronic bladder pain leads to involuntary tightening of pelvic floor muscles, creating a vicious cycle where muscle tension worsens pain, which in turn causes more tension .

2. Urinary Symptoms Disrupting Intimacy

**Urinary urgency and frequency**: The constant need to urinate interrupts sexual activity and makes patients feel anxious about accidents.

**Nocturia (nighttime urination)**: Frequent nighttime bathroom trips reduce sleep quality and energy levels, decreasing sexual desire.

3. Psychological and Emotional Impacts

**Decreased libido**: Chronic pain and fear of triggering symptoms often lead to reduced sexual desire.

**Body image issues and low self-esteem**: Patients may feel embarrassed about their urinary symptoms or perceive themselves as "broken."

**Relationship strain**: Misunderstandings about the condition, reduced intimacy, and caregiver burden can create significant tension between partners .

**Anxiety and depression**: These are common comorbidities in IC patients and further worsen sexual function.

Gender-Specific Differences

| **Aspect** | **Women with IC** | **Men with IC** |
| --- | --- | --- |
| Prevalence | 90% of IC patients | 10% of IC patients |
| Common misdiagnoses | Endometriosis, vulvodynia | Chronic prostatitis/chronic pelvic pain syndrome |
| Primary sexual symptoms | Vaginal pain during penetration, vulvar tenderness | Painful ejaculation, erectile dysfunction |
| Impact severity | Often more severe due to anatomical proximity of bladder and reproductive organs | Frequently underdiagnosed and undertreated |

Coping Strategies and Treatment Options

Medical Interventions

**Oral medications**: Pentosan polysulfate sodium (Elmiron), amitriptyline, and hydroxyzine can reduce bladder inflammation and pain .

**Bladder instillations**: Direct delivery of medications (such as dimethyl sulfoxide, heparin, or lidocaine) into the bladder can provide temporary pain relief.

**Nerve stimulation**: Sacral neuromodulation (InterStim) or percutaneous tibial nerve stimulation (PTNS) can help manage urinary symptoms and pain.

Physical and Behavioral Approaches

**Pelvic floor physical therapy**: This is considered first-line treatment for IC-related sexual dysfunction. A specialized therapist can teach relaxation techniques to reduce muscle tension .

**Position modifications**: Experimenting with different sexual positions that minimize pressure on the bladder can reduce pain.

**Timing**: Planning sexual activity during periods when symptoms are milder, and emptying the bladder completely before intimacy.

**Lubrication**: Using water-based lubricants can reduce friction and discomfort.

Relationship and Psychological Support

**Open communication**: Discussing fears, limitations, and needs with your partner is crucial.

**Couples therapy**: Can help partners understand the condition and work together to maintain intimacy.

**Individual counseling**: Can address anxiety, depression, and body image issues.

Important Note

Sexual dysfunction in IC is often multifactorial, requiring a comprehensive, multidisciplinary approach involving urologists, pelvic floor physical therapists, and mental health professionals. Many patients experience significant improvement in sexual function with appropriate treatment and management strategies.

**Disclaimer**: This information is provided for educational purposes only and is not a substitute for professional medical advice, diagnosis, or treatment. Always seek the advice of your physician or other qualified health provider with any questions you may have regarding a medical condition.

**49.What can someone do if interstitial cystitis causes pain during sex?**

Managing Pain During Sex Caused by Interstitial Cystitis (IC/BPS)

**Disclaimer:** This information is for educational purposes only and does not replace professional medical advice. Always consult your urologist, gynecologist, or pelvic floor physical therapist for personalized treatment plans tailored to your specific condition.

Pain during sexual activity (dyspareunia) is extremely common in interstitial cystitis/bladder pain syndrome, affecting 50-70% of people with IC. It typically occurs due to bladder irritation, pelvic floor muscle dysfunction, or inflammation in the pelvic region. Below is a comprehensive, evidence-based approach to managing this symptom:

Immediate Pain Relief During an Episode

**Stop activity immediately** if pain becomes severe—pushing through can worsen inflammation and trigger a multi-day flare-up

Empty your bladder completely before and immediately after sex (residual urine increases pressure and irritation)

Apply a cold pack wrapped in a towel to the lower abdomen or perineum for 10-15 minutes to reduce inflammation

Take a warm (not hot) sitz bath for 10-15 minutes to relax tight pelvic floor muscles

Use over-the-counter pain relievers as directed by your doctor: acetaminophen is generally safer than NSAIDs (which can irritate the bladder in some people)

Pre-Sex Preparation Strategies

**Time sex strategically**: Avoid activity during known flare-ups. Many people find they have less pain 1-2 hours after taking their IC medications

**Empty your bladder completely** right before sex—even if you don't feel the urge

**Use adequate lubrication**: Choose water-based, fragrance-free, pH-balanced lubricants. Avoid products with glycerin, parabens, or spermicides, which can irritate the bladder and urethra

**Pre-medicate if recommended**: Your doctor may suggest taking a prescribed pain reliever, antihistamine, or bladder analgesic 30-60 minutes before sexual activity

**Do gentle pelvic floor relaxation exercises** (diaphragmatic breathing, reverse Kegels) for 5-10 minutes beforehand to release muscle tension

During-Sex Adjustments

**Experiment with positions**: Choose positions that minimize pressure on the bladder. Side-lying (spooning), woman-on-top (allowing control of depth and pace), or rear-entry with a pillow under the hips are often better than missionary position

**Communicate openly with your partner**: Establish clear signals for when to slow down, change positions, or stop. Emphasize that this is a medical issue, not a reflection of your attraction or relationship

**Focus on non-penetrative intimacy**: Explore other forms of sexual pleasure that don't involve bladder pressure. This can help maintain connection while reducing pain

**Avoid deep penetration**: Shallow thrusting or using shorter sex toys can significantly reduce discomfort

**Take breaks as needed**: Pausing periodically to relax muscles and check in with your body can prevent pain from escalating

Post-Sex Care

**Urinate immediately** to flush out any bacteria that may have entered the urethra and reduce bladder pressure

Drink 8-12 ounces of water to dilute urine (avoid acidic drinks like coffee, soda, or juice)

Repeat pelvic floor relaxation exercises to prevent muscle spasms

Apply a cold pack if you feel any early signs of a flare-up

Take any prescribed post-activity medications as directed by your doctor

Track your symptoms in a journal to identify patterns (e.g., which positions, times of day, or activities trigger more pain)

Medical Treatments for Chronic Pain

If lifestyle adjustments aren't sufficient, your healthcare provider may recommend:

**Pelvic floor physical therapy (PFPT)**: The first-line treatment for IC-related dyspareunia. A specialized therapist can teach you to release tight pelvic floor muscles, reduce trigger points, and improve muscle function

**Oral medications**:

Amitriptyline or other tricyclic antidepressants (reduce nerve pain and inflammation)

Hydroxyzine (antihistamine that reduces bladder inflammation)

Pentosan polysulfate sodium (Elmiron) (may help repair the bladder lining)

**Bladder instillations**: Medications (such as heparin, lidocaine, and sodium bicarbonate) placed directly into the bladder to reduce inflammation and pain

**Nerve stimulation therapies**: Sacral neuromodulation (InterStim) or percutaneous tibial nerve stimulation (PTNS) for refractory cases

**Trigger point injections**: For severe pelvic floor muscle spasms

Emotional and Relationship Considerations

**Be patient with yourself and your partner**: IC is a chronic condition, and managing sexual pain takes time and experimentation

Consider couples counseling to help navigate the emotional impact of IC on your relationship

Join an IC support group (in-person or online) to connect with others who understand your experience

Remember that intimacy is about connection, not just penetrative sex. There are many ways to maintain a fulfilling sexual relationship with IC

When to See a Doctor Immediately

Contact your healthcare provider right away if you experience:

Severe, unrelenting pain that doesn't respond to your usual treatments

Blood in your urine

Fever, chills, or other signs of infection

Inability to urinate

Pain that lasts more than 24-48 hours after sexual activity

**50. Can interstitial cystitis symptoms get worse after sex?**

Can Interstitial Cystitis Symptoms Get Worse After Sex?

**Yes, sexual activity is one of the most common triggers for symptom flares in people with interstitial cystitis/bladder pain syndrome (IC/BPS).** This is a well-documented clinical observation that affects a significant majority of patients.

Prevalence

Approximately **70-90% of women** with IC/BPS report that sexual intercourse worsens their symptoms

About **50% of men** with IC/BPS experience post-coital symptom exacerbation

For many patients, this is one of the most disabling aspects of the condition, significantly impacting quality of life and intimate relationships

Why This Happens

1. Physical Irritation

The bladder and urethra are anatomically very close to the vaginal canal in women

Friction and pressure during intercourse directly irritate the already hypersensitive bladder wall and pelvic floor muscles

The urethral opening (meatus) can become inflamed, triggering bladder spasms

2. Pelvic Floor Dysfunction

Most IC/BPS patients have underlying pelvic floor muscle hypertonicity (tightness)

Sexual activity can further tense these muscles, creating a cycle of pain and spasm

Tight pelvic floor muscles compress the bladder, increasing urgency and frequency

3. Neurogenic Inflammation

IC/BPS involves abnormal sensory nerve signaling in the bladder

Physical stimulation during sex activates these hypersensitive nerves

This triggers the release of inflammatory substances (substance P, histamine) that worsen bladder pain and irritation

4. Other Contributing Factors

Use of spermicides, lubricants, or condoms that contain irritants

Urinary tract infections (which are more common in IC patients)

Dehydration before or after intercourse

Prolonged or vigorous sexual activity

Typical Post-Coital Symptoms

Symptoms usually appear **within minutes to hours** after intercourse and may last from a few hours to several days:

Increased pelvic pain, pressure, or discomfort

Severe urinary urgency and frequency

Burning or stinging during urination

Feeling of incomplete bladder emptying

Lower abdominal cramping

Evidence-Based Management Strategies

Before Intercourse

Empty your bladder completely before and immediately after sex

Drink a glass of water to dilute urine

Avoid known bladder irritants (caffeine, alcohol, spicy foods) for several hours beforehand

Use a pH-neutral, fragrance-free lubricant (avoid products with parabens, glycerin, or spermicides)

Consider gentle pelvic floor relaxation exercises (Kegel relaxation, not contraction)

During Intercourse

Choose positions that minimize pressure on the bladder (e.g., side-lying)

Communicate with your partner about discomfort and pace

Avoid deep penetration if it causes pain

After Intercourse

Urinate immediately to flush out any bacteria

Apply a warm compress to the lower abdomen

Take a warm (not hot) bath

Use prescribed rescue medications as directed by your healthcare provider

Rest and avoid strenuous activity

When to See a Doctor

Contact your healthcare provider if:

Post-coital flares become more frequent or severe

You develop fever, chills, or blood in your urine (signs of a UTI)

Symptoms last longer than 3-4 days

The pain is unbearable despite home management

You experience significant emotional distress or relationship problems

Important Notes

Not all IC/BPS patients experience this, and severity varies widely between individuals

Some patients may find that symptoms improve over time with proper treatment

Pelvic floor physical therapy is highly effective for reducing post-coital pain in many cases

Open communication with your partner and healthcare team is essential for managing this aspect of IC/BPS

**Disclaimer:** This information is for educational purposes only and is not a substitute for professional medical advice, diagnosis, or treatment. Always seek the advice of your physician or other qualified health provider with any questions you may have regarding a medical condition.

**51. What should someone do if interstitial cystitis symptoms worsen during pregnancy?**

Management of Worsening Interstitial Cystitis (IC/BPS) Symptoms During Pregnancy

**Disclaimer**: This information is for educational purposes only and does not constitute medical advice. Interstitial cystitis/bladder pain syndrome (IC/BPS) management during pregnancy requires individualized care from a multidisciplinary team including your obstetrician and urologist. Always consult your healthcare provider before starting, stopping, or adjusting any treatment.

Immediate First Steps When Symptoms Worsen

**Contact your healthcare team promptly**

Notify both your obstetrician and urologist within 24-48 hours of symptom escalation

Document symptom severity, frequency, duration, and any potential triggers (dietary changes, stress, increased activity)

Rule out urinary tract infection (UTI) first, as pregnancy increases UTI risk and symptoms can mimic IC flares

**Initiate basic flare control measures immediately**

Rest in a comfortable position (side-lying is best in later pregnancy to reduce bladder pressure)

Apply a warm (not hot) heating pad to the lower abdomen or perineum for 15-20 minutes at a time

Practice slow, deep breathing to reduce pelvic floor tension and pain perception

Safe Non-Pharmacological Management (First-Line)

These strategies have no known fetal risks and should be the foundation of your management plan:

Bladder and Hydration Management

**Timed voiding**: Urinate every 1-2 hours during the day, even if you don't feel the urge, to prevent bladder overdistension

**Avoid holding urine** for prolonged periods, which can trigger severe flares

**Moderate hydration**: Drink 6-8 cups (1.5-2L) of water daily, sipping small amounts throughout the day rather than large quantities at once

**Eliminate all known dietary triggers**: Strictly avoid caffeine, alcohol, artificial sweeteners, citrus fruits, tomatoes, spicy foods, chocolate, and carbonated beverages

Pelvic Floor and Physical Therapy

**Continue or initiate pregnancy-safe pelvic floor physical therapy** (PFPT) with a therapist trained in both IC and prenatal care

Practice gentle pelvic floor relaxation exercises (not Kegels, which can increase tension)

Use a pregnancy support belt to reduce abdominal pressure on the bladder in the second and third trimesters

Pain and Stress Management

**Warm sitz baths**: Soak in 3-4 inches of warm water for 10-15 minutes, 2-3 times daily (avoid hot water)

**Mind-body techniques**: Guided meditation, progressive muscle relaxation, and prenatal yoga can significantly reduce IC-related pain and stress

**Transcutaneous electrical nerve stimulation (TENS)**: Low-frequency TENS applied to the lower abdomen or sacrum is considered safe during pregnancy for pain relief

Pharmacological Management (Second-Line, Prescriber-Only)

All medications carry potential risks during pregnancy. Your provider will only recommend them if the benefits of symptom control clearly outweigh the risks to the fetus.

| **Medication Class** | **Examples** | **Pregnancy Safety Notes** |
| --- | --- | --- |
| **Analgesics** | Acetaminophen (paracetamol) | First-line pain reliever; use the lowest effective dose for the shortest duration |
|  | NSAIDs (ibuprofen, naproxen) | **AVOID after 20 weeks gestation** due to risk of fetal kidney damage and premature closure of the ductus arteriosus |
|  | Opioids | Only for severe, refractory pain; use the lowest dose possible and monitor closely for maternal and fetal side effects |
| **Antihistamines** | Hydroxyzine | Considered relatively safe; often used for IC-related bladder inflammation and nocturia |
| **Tricyclic Antidepressants** | Amitriptyline | Used cautiously in second and third trimesters for pain and frequency; avoid in first trimester |
| **Bladder Instillations** | Heparin + lidocaine + sodium bicarbonate | **Preferred pharmacological treatment**; minimal systemic absorption, making them very low risk for the fetus |
|  | Pentosan polysulfate sodium (Elmiron) | Limited safety data; generally avoided during pregnancy unless all other treatments have failed |

When to Seek Urgent/Emergent Care

Contact your provider immediately or go to the emergency department if you experience:

Severe, unrelenting pain that does not respond to prescribed treatments

Fever (temperature >100.4°F/38°C) or chills

Blood in the urine (hematuria)

Difficulty urinating or inability to urinate

Signs of preterm labor: regular contractions, pelvic pressure, vaginal bleeding, or fluid leakage

Long-Term Management Considerations

**Multidisciplinary care coordination**: Ensure your obstetrician and urologist communicate regularly about your care plan

**Regular prenatal visits**: Attend all scheduled appointments to monitor both your health and the baby's development

**Labor and delivery planning**: Discuss your IC symptoms with your provider before labor. Vaginal delivery is generally safe, but prolonged pushing may worsen pelvic floor dysfunction. Epidural anesthesia can help reduce pain during labor.

**Postpartum care**: IC symptoms may improve after delivery as hormone levels normalize and bladder pressure decreases. However, some women experience temporary worsening in the immediate postpartum period.

Important Precautions

**Never self-medicate** with over-the-counter or prescription medications without consulting your healthcare provider

Do not stop any prescribed IC medications abruptly without medical supervision

Be aware that pregnancy hormones can significantly alter IC symptoms, and your management plan may need to be adjusted multiple times throughout your pregnancy

Join a support group for women with IC during pregnancy to connect with others who have similar experiences

**52. What can someone do if interstitial cystitis causes anxiety?**

Managing Anxiety Caused by Interstitial Cystitis (IC)

**Disclaimer**: This information is for educational purposes only and does not replace professional medical or mental health advice. Always consult your healthcare provider or a licensed mental health professional for personalized treatment.

Interstitial cystitis (also called bladder pain syndrome, BPS) and anxiety have a well-documented **bidirectional relationship**: IC symptoms (chronic pelvic pain, urinary urgency/frequency, sleep disruption) trigger significant anxiety, and anxiety in turn worsens pelvic floor tension, inflammation, and bladder hypersensitivity, creating a vicious cycle. Below is a comprehensive, evidence-based approach to breaking this cycle.

1. First: Optimize Your IC Medical Management

Anxiety often stems from unmanaged physical symptoms. Work closely with your urologist to:

Adjust medications (e.g., pentosan polysulfate sodium, amitriptyline, hydroxyzine) to reduce baseline pain and urgency

Consider pelvic floor physical therapy (PFPT) – this is a first-line treatment for IC that also relieves the muscle tension caused by anxiety

Explore bladder instillations or neuromodulation (e.g., sacral nerve stimulation) for refractory symptoms

Develop a personalized flare-up action plan to reduce uncertainty and panic when symptoms worsen

2. Evidence-Based Mental Health Interventions

Cognitive Behavioral Therapy (CBT)

**Gold standard** for chronic pain-related anxiety, specifically recommended by the American Urological Association (AUA) for IC management

Helps you identify and challenge catastrophic thoughts about IC (e.g., "This flare will never end" "Flare-ups are temporary, and I have tools to manage them")

Teaches pain coping skills and reduces the fear-avoidance cycle that limits daily activities

Look for a therapist trained in **chronic pain CBT** or **pelvic pain psychology**

Acceptance and Commitment Therapy (ACT)

Focuses on accepting uncomfortable symptoms rather than fighting them, which reduces the anxiety caused by trying to control every bladder sensation

Helps you align your actions with your values, even when living with IC

Particularly effective for long-term chronic conditions where complete symptom elimination may not be possible

Medication for Anxiety (When Needed)

Discuss with your provider whether short-term or long-term anxiety medication is appropriate

Some medications (e.g., certain SSRIs, SNRIs) can help both anxiety and chronic pain

**Important**: Avoid benzodiazepines long-term, as they can worsen pelvic floor dysfunction and have addiction risks

3. Immediate Anxiety Relief Techniques for Flare-Ups

These techniques work quickly to calm the nervous system and reduce pelvic tension:

**Diaphragmatic breathing**: Breathe deeply into your belly for 4 counts, hold for 4, exhale for 6. This activates the parasympathetic nervous system and relaxes the pelvic floor.

**muscle relaxation (PMR)**: Systematically tense and release muscle groups, paying special attention to the pelvic floor, lower back, and abdomen.

**5-4-3-2-1 grounding technique**: Name 5 things you see, 4 things you feel, 3 things you hear, 2 things you smell, 1 thing you taste. This pulls your mind out of anxious rumination.

**Cold exposure**: Splash cold water on your face or hold a cold pack to your forehead. This triggers the mammalian dive reflex and rapidly lowers heart rate.

4. Lifestyle Adjustments That Help Both IC and Anxiety

**Sleep hygiene**: Poor sleep exacerbates both pain and anxiety. Establish a consistent sleep schedule, avoid caffeine after noon, and create a relaxing bedtime routine.

**Gentle exercise**: Low-impact activities like walking, swimming, or yoga reduce stress hormones and improve pelvic blood flow. Avoid high-impact exercises that irritate the bladder.

**IC-friendly diet**: Avoid known bladder irritants (caffeine, alcohol, spicy foods, artificial sweeteners) to reduce symptom flares and the anxiety they cause.

**Limit bladder monitoring**: Constantly checking the time or rushing to the bathroom reinforces anxious patterns. Gradually extend bathroom intervals with your provider's guidance.

5. Build a Support System

Join IC support groups (in-person or online) to connect with others who understand your experience. The Interstitial Cystitis Association (ICA) offers reliable resources and support communities.

Educate your family and friends about IC so they can provide appropriate support and avoid minimizing your symptoms.

Consider working with a health coach who specializes in chronic illness to help you navigate daily challenges.

6. When to Seek Immediate Help

Contact a mental health professional or crisis hotline immediately if you experience:

Persistent thoughts of harming yourself or others

Inability to perform daily activities (work, self-care, relationships)

Panic attacks that occur frequently or are disabling

Severe depression that lasts more than two weeks

Remember that managing IC-related anxiety is a process, and it's okay to take it one day at a time. Combining medical management of IC with targeted anxiety strategies can significantly improve your quality of life.

**53.Can counseling help people cope with long-term interstitial cystitis pain?**

Can Counseling Help People Cope with Long-Term Interstitial Cystitis Pain?

**Yes, counseling is a highly evidence-based, core component of comprehensive long-term interstitial cystitis (IC)/bladder pain syndrome (BPS) management**, recommended by leading guidelines including the American Urological Association (AUA) and the International Continence Society (ICS) . It does not cure IC, but it directly addresses the bidirectional relationship between chronic pelvic pain and psychological distress, breaking the pain-anxiety-depression cycle that often worsens IC symptoms over time.

Why Counseling Is Critical for IC Pain

IC is a chronic, often treatment-resistant condition characterized by persistent pelvic pain, urinary urgency, and frequency. For many patients:

Pain signals become amplified in the brain (central sensitization), where psychological factors directly modulate pain perception

Chronic pain leads to anxiety, depression, sleep disturbances, and social isolation, which in turn lower pain thresholds and increase symptom severity

Patients often develop maladaptive coping behaviors (e.g., excessive fluid restriction, social withdrawal, catastrophizing about pain) that further impair quality of life

Counseling targets these psychological and neurobiological mechanisms, providing skills that complement medical and physical treatments.

Evidence-Based Counseling Approaches for IC

1. Cognitive Behavioral Therapy (CBT) – First-Line Psychological Treatment

CBT has the strongest research support for IC pain management. It helps patients:

Identify and challenge negative thought patterns (e.g., "This pain will never get better" or "I can't handle this") that amplify pain

Develop practical coping strategies (e.g., distraction techniques, pacing activities, relaxation training)

Modify behaviors that worsen symptoms (e.g., avoiding social events, overusing pain medications)

Improve sleep hygiene, which is closely linked to IC pain severity

A 2022 systematic review found that CBT significantly reduces IC pain intensity, urinary symptoms, and depression scores, with benefits lasting 6–12 months after treatment completion .

2. Acceptance and Commitment Therapy (ACT)

ACT is particularly effective for patients with treatment-resistant chronic pain. Instead of focusing on eliminating pain, it teaches:

Accepting pain as a part of life without judgment

Committing to activities that align with personal values, even when experiencing pain

Reducing the struggle against pain, which often increases its intensity

Studies show ACT improves physical function, emotional well-being, and quality of life in IC patients, even when pain intensity remains unchanged .

3. Mindfulness-Based Stress Reduction (MBSR)

MBSR combines mindfulness meditation, body scans, and gentle yoga to:

Reduce stress hormones (cortisol) that exacerbate bladder inflammation and pain

Help patients separate themselves from their pain sensations

Improve awareness of bodily cues without reacting with anxiety

A 2021 pilot study found that 8 weeks of MBSR significantly reduced IC pain, urinary urgency, and perceived stress in women with moderate to severe symptoms .

4. Pain Reprocessing Therapy (PRT)

PRT is a newer, neuroscience-based approach that targets central sensitization. It helps patients:

Recognize that their pain is not always a sign of tissue damage

Retrain the brain to interpret bladder signals as non-threatening

Reduce the emotional charge associated with pain sensations

Early research shows promising results for chronic pelvic pain conditions, including IC .

5. Support Groups

Peer support groups (in-person or online) provide:

Validation of experiences (many IC patients feel misunderstood by family, friends, and even healthcare providers)

Practical tips for managing daily life with IC

Reduced feelings of isolation

Specific Benefits of Counseling for Long-Term IC Patients

**Reduced pain intensity and frequency**: Studies show average pain reductions of 20–40% with evidence-based counseling approaches

**Improved coping skills**: Patients learn to manage pain flares more effectively, reducing reliance on emergency care

**Lower rates of anxiety and depression**: Up to 50% of IC patients experience comorbid depression, which counseling can significantly improve

**Better treatment adherence**: Patients who receive counseling are more likely to follow dietary restrictions, physical therapy regimens, and medication schedules

**Enhanced quality of life**: Counseling helps patients regain control over their lives and participate in activities they previously avoided

How to Maximize the Benefits of Counseling

**Find a counselor with experience in chronic pain, preferably pelvic pain or IC specifically**

**Combine counseling with other treatments**: It works best as part of a multimodal approach that may include medications, physical therapy, dietary changes, and bladder instillations

**Be patient**: Most patients see significant improvements after 8–12 weekly sessions, with maintenance sessions as needed

**Be open and honest**: Share your fears, frustrations, and goals with your counselor to get the most out of treatment

Limitations

Counseling is not a cure for IC and will not eliminate all symptoms for most patients

It requires active participation and practice of skills outside of sessions

Results vary from person to person, and some patients may need to try different approaches before finding what works for them

Disclaimer

This information is for educational purposes only and is not a substitute for professional medical advice, diagnosis, or treatment. Always seek the advice of your physician or other qualified healthcare provider with any questions you may have regarding a medical condition.

**54.How can interstitial cystitis affect work, sleep, and daily life?**

How Interstitial Cystitis (IC/BPS) Impacts Work, Sleep, and Daily Life

Interstitial cystitis (also called bladder pain syndrome, IC/BPS) is a chronic, debilitating condition characterized by **recurring pelvic pain, pressure, or discomfort** in the bladder region, accompanied by **urinary urgency and frequency** (often 8+ times per day and multiple times at night). Its impact extends far beyond urinary symptoms, profoundly affecting nearly every aspect of a person's life, with severity varying widely from mild inconvenience to complete disability.

Impact on Work

IC/BPS is one of the most common chronic urological conditions associated with work impairment, with studies showing that **30-50% of patients report reduced work productivity**, and 10-15% are forced to leave their jobs entirely.

**Constant disruption of work flow**

Frequent bathroom trips (every 15-60 minutes during flare-ups) interrupt concentration, meetings, and task completion. Patients often struggle with jobs requiring sustained focus (e.g., writing, coding, surgery) or uninterrupted presence (e.g., teaching, customer service, public speaking).

Sudden, overwhelming urinary urgency can create embarrassing situations, including accidents, which erode professional confidence.

**Chronic pain and cognitive impairment**

Persistent pelvic pain (ranging from mild discomfort to severe, stabbing pain) reduces attention span, decision-making ability, and overall productivity.

Pain-related fatigue and sleep deprivation (see below) lead to "brain fog," memory lapses, and slower reaction times.

**Absenteeism and career limitations**

Unpredictable flare-ups (triggered by stress, diet, menstruation, or physical activity) result in frequent unplanned absences.

Patients often cannot perform jobs requiring:

Long periods without bathroom access (truck driving, air travel, field work)

Prolonged sitting or standing (desk jobs, retail, nursing)

Heavy lifting or physical exertion (construction, manual labor)

Many patients must request accommodations (e.g., flexible hours, frequent breaks, remote work) or accept demotions to less demanding roles.

Impact on Sleep

Sleep disturbance is **the most commonly reported comorbidity** of IC/BPS, affecting 70-90% of patients, and it creates a vicious cycle with bladder symptoms.

**Nocturia (nighttime urination)**

Patients typically wake 3-10 times per night to urinate, even with minimal fluid intake. Some severe cases require hourly trips to the bathroom.

This prevents entry into deep, restorative sleep stages (REM and slow-wave sleep), leading to fragmented sleep regardless of total time in bed.

**Nocturnal pain exacerbation**

Bladder pain often worsens at night as the bladder fills during sleep. The pressure of accumulated urine stretches the inflamed bladder wall, triggering intense discomfort that jolts patients awake.

Many patients develop conditioned anxiety about falling asleep, fearing they will wake up in severe pain.

**Consequences of chronic sleep deprivation**

Daytime exhaustion, irritability, and mood swings

Worsened pain perception (sleep loss lowers pain thresholds)

Increased risk of depression and anxiety

Impaired immune function and overall physical health

Impact on Daily Life and Activities

IC/BPS restricts personal freedom and forces patients to reorganize their entire lives around their bladder symptoms.

**Social isolation and limited activities**

Patients avoid social gatherings, movies, concerts, or long outings because they cannot guarantee immediate access to a bathroom.

Many pre-plan every outing meticulously, mapping out bathroom locations in advance and limiting their travel radius.

Spontaneity is lost; last-minute plans are often declined due to fear of flare-ups.

**Household and physical limitations**

Routine chores (cleaning, grocery shopping, laundry) become exhausting or impossible during flare-ups.

Physical activities (walking, running, yoga, cycling) can trigger pelvic pain and urinary urgency, leading to a sedentary lifestyle and associated health problems.

Sexual activity is severely impacted: **50-70% of women with IC/BPS experience dyspareunia (pain during intercourse)**, and many men report post-ejaculatory pain. This often leads to decreased libido, relationship strain, and intimacy issues.

**Dietary restrictions and lifestyle changes**

Patients must eliminate common bladder irritants from their diet, including caffeine, alcohol, spicy foods, acidic fruits/vegetables, artificial sweeteners, and carbonated drinks.

This restricts dining out options and makes social meals stressful, as patients must carefully scrutinize menus or bring their own food.

Many patients also need to manage fluid intake carefully, avoiding both dehydration and overhydration, which can worsen symptoms.

Psychological and Emotional Impact

The chronic, invisible nature of IC/BPS makes its psychological toll particularly severe:

**Depression and anxiety**: Affect 40-60% of patients, caused by constant pain, sleep loss, social isolation, and loss of control over one's body.

**Guilt and shame**: Patients often feel guilty for being unable to fulfill work, family, or social obligations. The stigma surrounding urinary symptoms leads many to hide their condition from others.

**Frustration and hopelessness**: IC/BPS has no cure, and diagnosis often takes years (average 3-5 years) as symptoms mimic other conditions. Many patients feel dismissed by healthcare providers who initially attribute their symptoms to stress or psychological issues.

Important Note

While IC/BPS can be life-altering, **early diagnosis and comprehensive treatment** (including medication, physical therapy, dietary changes, and stress management) can significantly reduce symptoms and improve quality of life for most patients.

**Disclaimer**: This information is provided for educational purposes only and is not a substitute for professional medical advice, diagnosis, or treatment. Always seek the advice of your physician or other qualified health provider with any questions you may have regarding a medical condition.

**55.How should interstitial cystitis be managed over the long term?**

Long-Term Management of Interstitial Cystitis (IC/BPS)

Interstitial cystitis, also known as bladder pain syndrome (IC/BPS), is a chronic, relapsing-remitting condition characterized by bladder pain, urinary frequency, urgency, and nocturia. There is no cure, so long-term management focuses on **symptom control, flare prevention, preservation of bladder function, and improvement of quality of life**. Management is highly individualized, as IC/BPS is heterogeneous and responds differently to treatments across patients.

Core Principles of Long-Term Management

Adopt a **multimodal, stepwise approach** (start with least invasive, most effective options)

Prioritize **patient education and self-management**

Address both bladder-specific symptoms and associated conditions (pelvic floor dysfunction, anxiety, depression)

Regularly reassess and adjust the treatment plan based on symptom changes and treatment response

1. Foundational Self-Management Strategies (First-Line, Lifelong)

These strategies form the backbone of long-term IC/BPS management and should be implemented alongside all other treatments.

Diet Modification

**Identify and avoid trigger foods**: Common triggers include acidic foods (citrus, tomatoes), caffeine, alcohol, artificial sweeteners (especially aspartame), spicy foods, chocolate, carbonated beverages, and certain preservatives.

**Keep a detailed food-symptom diary** for 2-4 weeks to identify personal triggers, as triggers vary significantly between individuals.

**Gradual reintroduction**: Once triggers are identified, reintroduce foods one at a time to determine tolerance levels.

Hydration Management

Maintain **balanced hydration**: Avoid both overhydration (which increases bladder pressure) and dehydration (which concentrates urine and irritates the bladder lining).

Sip small amounts of water throughout the day rather than drinking large quantities at once.

Limit fluid intake 2-3 hours before bedtime to reduce nocturia.

Bladder Training

Gradually increase the time between voids by 15-30 minutes every 1-2 weeks, aiming for a voiding interval of 3-4 hours.

Use distraction techniques (deep breathing, counting) to manage urgency between voids.

Avoid "just in case" voiding, as this can worsen frequency over time.

Lifestyle Adjustments

**Quit smoking**: Smoking irritates the bladder lining and increases the risk of bladder cancer.

Avoid tight clothing around the abdomen and pelvis, which can increase pressure on the bladder.

Limit prolonged sitting; take frequent breaks to stand and stretch.

Avoid high-impact exercises that jar the bladder (e.g., running); opt for low-impact activities like swimming, walking, or yoga.

2. Pharmacological Management

Oral Medications

| **Medication Class** | **Examples** | **Indications** | **Long-Term Considerations** |
| --- | --- | --- | --- |
| Tricyclic Antidepressants | Amitriptyline, Nortriptyline | First-line for pain and urgency; reduces bladder spasms and nerve sensitivity | Monitor for anticholinergic side effects (dry mouth, constipation, drowsiness); start low and titrate slowly |
| Antihistamines | Hydroxyzine | Effective for patients with allergic component or mast cell activation | Sedation is common; take at bedtime |
| Glycosaminoglycan (GAG) Replacement | Pentosan Polysulfate Sodium (PPS) | Restores the bladder lining's protective GAG layer | **Black box warning**: Risk of retinal toxicity with long-term use; require annual eye exams |
| Neuropathic Pain Agents | Gabapentin, Pregabalin | Second-line for refractory neuropathic pain | Monitor for dizziness, drowsiness, and weight gain |
| Immunosuppressants | Cyclosporine A | Severe, refractory cases | Significant side effects (nephrotoxicity, hypertension); require close monitoring of blood levels and kidney function |

Intravesical Therapies

These are administered directly into the bladder via catheter, typically in cycles, and are used when oral medications are insufficient.

**First-line**: Dimethyl sulfoxide (DMSO) - anti-inflammatory, analgesic, and muscle relaxant properties; administered weekly for 6-8 weeks, then maintenance every 1-3 months.

**Second-line**:

Heparin + lidocaine combination: Provides rapid pain relief and protects the bladder lining

Botulinum toxin type A (Botox): Injected into the bladder wall to reduce muscle spasms and pain; effects last 6-9 months, requiring repeat treatments

Hyaluronic acid and chondroitin sulfate: GAG layer replacement therapies

3. Physical and Behavioral Therapies

Pelvic Floor Physical Therapy (PFPT)

**Highly recommended** for all IC/BPS patients, as up to 80% have associated pelvic floor dysfunction (tight, overactive pelvic floor muscles).

Techniques include myofascial release, trigger point therapy, and relaxation exercises to reduce muscle tension and pain.

Long-term benefits include reduced pain, improved bladder function, and decreased reliance on medications.

Other Physical Therapies

**Transcutaneous Electrical Nerve Stimulation (TENS)**: Delivers low-voltage electrical currents to the pelvic area to block pain signals; can be used at home for long-term management.

**Posterior Tibial Nerve Stimulation (PTNS)**: Non-invasive neuromodulation that stimulates the tibial nerve to improve bladder function; administered weekly for 12 weeks, then maintenance sessions.

Stress Management

Chronic pain and urinary symptoms can lead to significant stress, which in turn worsens IC/BPS symptoms (bidirectional relationship).

Evidence-based techniques include mindfulness meditation, deep breathing exercises, progressive muscle relaxation, and yoga.

4. Advanced and Surgical Options (Refractory Cases)

These are reserved for patients who have failed all conservative and pharmacological treatments and have severely impaired quality of life.

Neuromodulation

**Sacral Nerve Stimulation (InterStim)**: Implantable device that delivers electrical impulses to the sacral nerves to regulate bladder function; provides long-term symptom relief in approximately 50-70% of refractory patients.

Surgical Procedures

**Hydrodistention**: Bladder is filled with fluid under pressure to stretch the bladder wall; provides temporary relief (3-6 months) and may be repeated periodically.

**Cystectomy with Urinary Diversion**: Last-resort procedure for patients with end-stage IC/BPS who have intractable pain and severely reduced bladder capacity; involves removing the bladder and creating a new way for urine to exit the body.

5. Mental Health and Quality of Life

IC/BPS is associated with high rates of anxiety, depression, sleep disturbances, and sexual dysfunction.

**Cognitive Behavioral Therapy (CBT)**: Effective for managing chronic pain, improving coping skills, and reducing anxiety and depression.

**Support Groups**: Connecting with other IC/BPS patients can provide emotional support and practical advice.

**Sexual Health Counseling**: Addresses sexual dysfunction, which is common in IC/BPS patients due to pain and anxiety.

6. Long-Term Monitoring and Follow-Up

Schedule regular follow-up appointments with a urologist or urogynecologist who specializes in IC/BPS (every 3-6 months initially, then annually once stable).

Monitor for medication side effects (e.g., retinal toxicity with PPS, kidney function with cyclosporine).

Assess bladder function and symptom severity using validated questionnaires (e.g., O'Leary-Sant Symptom and Problem Index).

Update the treatment plan as needed, including tapering or discontinuing medications when symptoms are well-controlled.

Disclaimer

This information is for educational purposes only and is not a substitute for professional medical advice, diagnosis, or treatment. Always seek the advice of your physician or other qualified healthcare provider with any questions you may have regarding a medical condition.

**56.Should people with interstitial cystitis keep following their care plan after symptoms improve?**

**Yes, people with interstitial cystitis/bladder pain syndrome (IC/BPS) should absolutely continue following a modified version of their care plan after symptoms improve.** This is one of the most critical principles in managing this chronic condition, as recommended by the American Urological Association (AUA), Canadian Urological Association (CUA), and all major international guidelines American Urological Association.

Why You Cannot Stop Care Completely

1. IC/BPS is a chronic, incurable condition

There is currently no cure for IC/BPS . Complete, permanent remission occurs in fewer than 10% of patients . Most people experience a pattern of **intermittent flares and periods of symptom improvement** rather than a one-time recovery American Urological Association.

2. High recurrence rate with treatment cessation

Studies show that 90% of patients experience symptom deterioration when treatment is abruptly stopped

For Hunner-type IC (the more severe inflammatory subtype), the 2-year recurrence rate can be as high as 86.2% without maintenance therapy

Even after successful surgical treatment, the average time to relapse is only 20 months without ongoing management

3. Risk of disease progression

Without proper long-term management:

Bladder wall fibrosis can progress, leading to permanently reduced bladder capacity

Symptoms may become more severe and harder to treat over time

Quality of life can significantly decline, with increased risk of anxiety and depression (which affect over 50% of IC/BPS patients)

How to Adjust Your Care Plan After Improvement

Rather than stopping all treatment, the goal is to transition to a **maintenance phase** that keeps symptoms under control with the least possible intervention.

1. Medications

**Gradual tapering, not abrupt stopping**: Work with your doctor to slowly reduce medication doses over weeks or months

**Maintenance doses**: Most patients require a low maintenance dose of at least one medication (often pentosan polysulfate sodium or similar bladder lining protectors)

**Rescue plan**: Keep a supply of fast-acting treatments (like lidocaine bladder instillations) on hand for unexpected flares American Urological Association

2. Bladder instillations

After initial weekly sessions, transition to monthly maintenance instillations for 6-12 months

Maintenance therapy has been shown to reduce recurrence rates by 62% in Hunner-type IC patients

3. Lifestyle modifications (THESE SHOULD BE PERMANENT)

These first-line treatments are the foundation of long-term management and should never be abandoned:

**Dietary restrictions**: Continue avoiding your personal trigger foods (caffeine, alcohol, artificial sweeteners, acidic foods, etc.)

**Fluid management**: Maintain consistent, adequate hydration (1500-2000ml daily)

**Stress management**: Continue practices that reduce stress, as stress is a major trigger for flares

**Pelvic floor care**: Continue relaxation techniques and avoid activities that increase pelvic tension

4. Regular follow-up

Schedule check-ups at 1, 3, and 6 months after achieving symptom control

Long-term annual monitoring is recommended to assess disease progression and adjust treatment as needed

When You Might Be Able to Reduce Treatment More Aggressively

A small percentage of patients (primarily those with non-Hunner type IC) may be able to discontinue medications entirely after several years of stable symptoms . However:

This should only be done under close medical supervision

You must continue all lifestyle modifications

You should have a clear plan for restarting treatment immediately if symptoms return

Disclaimer

This information is for educational purposes only and is not a substitute for professional medical advice, diagnosis, or treatment. Always seek the advice of your physician or other qualified health provider with any questions you may have regarding a medical condition.

**57.Should online claims about a complete cure for interstitial cystitis be trusted?**

Should Online Claims About a Complete Cure for Interstitial Cystitis Be Trusted?

**No. As of May 2026, there is no scientifically proven, universally effective "complete cure" for interstitial cystitis (IC), also known as bladder pain syndrome (BPS). All online claims promising a guaranteed, permanent cure are almost universally untrustworthy and potentially dangerous.**

Disclaimer

This information is for educational purposes only and does not constitute medical advice. Interstitial cystitis is a complex chronic condition that requires personalized diagnosis and management by a qualified urologist or pelvic pain specialist. Always consult a healthcare provider before starting any new treatment.

Why No Complete Cure Exists Today

IC/BPS is not a single disease but a heterogeneous syndrome with multiple potential underlying causes, including:

Defects in the bladder's protective glycosaminoglycan (GAG) layer

Neurogenic inflammation and central sensitization

Immune system dysregulation

Pelvic floor muscle dysfunction

Genetic predispositions

Possible infectious triggers that have not been definitively identified

Because the root cause varies significantly between patients, no single treatment can address all cases. The medical community universally recognizes IC as a chronic, manageable condition rather than a curable one.

Common Misleading Online Claims and Their Risks

1. "Miracle" supplements and natural remedies

**Claims**: Herbal blends, special vitamins, colloidal silver, or "detox" protocols that claim to "heal the bladder" permanently

**Reality**: While some supplements (such as oral pentosan polysulfate sodium, which is FDA-approved) may provide symptom relief for some patients, none have been shown to cure IC. Unregulated supplements can cause liver/kidney damage, interact with prescription medications, and contain undisclosed ingredients.

2. Unproven medical procedures

**Claims**: Stem cell therapy, ozone therapy, hyperbaric oxygen therapy, or experimental surgeries marketed as "cures"

**Reality**: These treatments are not supported by large-scale, peer-reviewed clinical trials. They are often extremely expensive, not covered by insurance, and can cause serious complications including infection, bladder damage, and worsening pain.

3. Diet and lifestyle "cures"

**Claims**: Strict elimination diets, alkaline water regimens, or specific exercise programs that claim to "reverse" IC

**Reality**: Diet modification is a cornerstone of IC management and can significantly reduce symptoms for many patients. However, it does not cure the underlying condition, and triggers vary widely between individuals.

4. "Secret" or "underground" treatments

**Claims**: Treatments that "doctors won't tell you about" or are "suppressed by big pharma"

**Reality**: Legitimate medical breakthroughs are published in peer-reviewed journals and adopted by the medical community worldwide. Claims of suppression are almost always a marketing tactic to sell unproven products.

Evidence-Based Management Strategies

While there is no cure, most patients with IC can achieve significant symptom improvement and maintain a good quality of life with personalized, multimodal treatment:

Oral medications (pentosan polysulfate sodium, amitriptyline, hydroxyzine)

Bladder instillations (dimethyl sulfoxide, heparin, lidocaine)

Pelvic floor physical therapy

Diet and lifestyle modifications

Neuromodulation therapies (sacral nerve stimulation)

Pain management techniques

Red Flags for Untrustworthy Claims

Be extremely skeptical of any online claim that:

Uses words like "guaranteed cure," "permanent relief," or "100% effective"

Relies solely on anecdotal testimonials rather than scientific evidence

Claims to work for "all cases" of IC

Is sold directly to consumers without a prescription

Criticizes mainstream medicine or claims doctors are withholding information

Requires large upfront payments or long-term commitments

How to Find Reliable Information

Consult a urologist or pelvic pain specialist who has experience treating IC

Refer to reputable medical organizations such as the Interstitial Cystitis Association (ICA), American Urological Association (AUA), or National Institute of Diabetes and Digestive and Kidney Diseases (NIDDK)

Look for information published in peer-reviewed medical journals

Join patient support groups led by medical professionals

Final Note

Research into IC is ongoing, and scientists are making progress in understanding its causes and developing new treatments. However, any legitimate cure would first be rigorously tested in clinical trials, reviewed by independent experts, and approved by regulatory agencies such as the FDA. Until then, be wary of any online claim that promises a complete cure.

**58.Is it safe to diagnose or treat interstitial cystitis only with advice from the internet?**

**Absolutely not.** Relying solely on internet information to diagnose or treat interstitial cystitis/bladder pain syndrome (IC/BPS) is dangerous and can lead to serious health consequences, delayed care for life-threatening conditions, and worsening of symptoms. Below is a detailed, evidence-based explanation:

Why Self-Diagnosis from the Internet Is Unreliable

IC/BPS is a complex, chronic condition with **no definitive "gold standard" diagnostic test**—it is a diagnosis of exclusion, meaning doctors must first rule out dozens of other conditions that present with identical symptoms.

**Symptom overlap with life-threatening or treatable conditions**

Internet symptom checklists cannot distinguish IC/BPS from:

Bladder cancer (early-stage symptoms are nearly identical to IC/BPS, especially in smokers or those over 40)

Recurrent urinary tract infections (UTIs) (many IC patients are misdiagnosed with chronic UTIs and overprescribed antibiotics)

Kidney stones, urethral strictures, or bladder outlet obstruction

Prostatitis/chronic pelvic pain syndrome (in men)

Endometriosis, pelvic inflammatory disease, or ovarian cysts (in women)

Neurological conditions (e.g., multiple sclerosis, spinal cord injury)

**Diagnosis requires specialized in-office procedures**

A proper IC/BPS workup cannot be done remotely. It typically includes:

Urinalysis, urine culture, and cytology (to rule out infection and cancer)

Cystoscopy with hydrodistension (the gold standard for identifying Hunner's lesions, a subtype of IC/BPS that requires different treatment)

Urodynamic testing (to evaluate bladder function)

Pelvic floor muscle assessment (many IC patients have coexisting pelvic floor dysfunction)

**IC/BPS has heterogeneous subtypes**

The condition is not a single disease but a spectrum of disorders. Internet resources often present a one-size-fits-all description, but:

Ulcerative (Hunner's lesion) IC/BPS responds very differently to treatment than non-ulcerative IC/BPS

Some patients have primarily bladder pain, while others have urgency/frequency as their main symptom

Subtypes require tailored treatment approaches that only a trained clinician can identify

Why Self-Treatment from the Internet Is Dangerous

IC/BPS treatment is highly individualized, and what works for one person may harm another.

**Prescription medications require medical supervision**

First-line IC/BPS medications (e.g., pentosan polysulfate sodium, amitriptyline, hydroxyzine) have significant risks and contraindications:

Pentosan polysulfate sodium has been linked to retinal damage with long-term use, requiring regular eye exams

Amitriptyline can cause cardiac arrhythmias, drowsiness, and anticholinergic side effects

Self-medicating with these drugs without proper dosing and monitoring can lead to severe adverse reactions

**Common internet "remedies" can worsen symptoms**

Many widely recommended online treatments are either unproven or harmful:

**Overuse of antibiotics**: Most IC/BPS is not caused by infection; unnecessary antibiotics lead to antibiotic resistance, gut dysbiosis, and fungal infections

**Harsh bladder instillations**: Self-administered instillations can cause bladder irritation, infection, or perforation

**Extreme dietary restrictions**: While some foods trigger IC/BPS symptoms, overly restrictive diets can lead to malnutrition and eating disorders

**Unregulated supplements**: Many "IC supplements" sold online are not FDA-approved, contain undisclosed ingredients, or interact with prescription medications

**Delayed treatment for severe cases**

Severe IC/BPS can lead to:

Bladder wall fibrosis and reduced bladder capacity

Chronic kidney damage (in rare cases of long-standing obstruction)

Severe depression, anxiety, and suicidal ideation (IC/BPS has one of the highest rates of mental health comorbidities among chronic conditions)

Inability to work or perform daily activities

The Problem with Internet Information Itself

**Accuracy varies widely**: Many websites contain outdated, incorrect, or commercially biased information (e.g., sites selling unproven products)

**Anecdotal evidence is not medical advice**: Patient forums and social media share personal experiences, which do not constitute clinical evidence. What worked for one person may not work for you, and some anecdotes describe dangerous practices

**Lack of context**: Internet resources cannot account for your individual medical history, allergies, other medications, or comorbid conditions

What You *Can* Safely Use the Internet For

The internet can be a valuable **complement** to professional medical care, not a replacement:

Learn about basic IC/BPS facts from reputable sources (e.g., American Urological Association, Mayo Clinic, Interstitial Cystitis Association)

Prepare questions for your doctor

Find support groups for emotional support

Track your symptoms (using apps or journals) to share with your clinician

Recommended Next Steps

Schedule an appointment with a **urologist who specializes in IC/BPS or chronic pelvic pain**

Bring a detailed symptom journal (including when symptoms occur, what triggers them, and what relieves them)

Ask about all available treatment options and their risks/benefits

Work with your doctor to develop a personalized treatment plan that may include medications, physical therapy, dietary changes, and behavioral modifications

Follow up regularly to adjust your treatment as needed

**Disclaimer**: This information is for educational purposes only and is not a substitute for professional medical advice, diagnosis, or treatment. Always seek the advice of your physician or other qualified health provider with any questions you may have regarding a medical condition.

**Gemini**

**1. What is interstitial cystitis?**

**Interstitial cystitis** (IC), often called **bladder pain syndrome** (BPS), is a chronic condition that causes discomfort, pressure, or intense pain in the bladder and pelvic area.

Normally, your bladder expands until it's full and then sends a signal to your brain that it's time to urinate. With interstitial cystitis, these signals get mixed up. The walls of the bladder become irritated and inflamed, making you feel the need to urinate more often and with much smaller volumes of urine than most people.

Inflammation of the bladder wall in IC. *Glycologix*

**What Does It Feel Like?**

The symptoms of IC vary wildly from person to person. Some people may have periods where they are entirely symptom-free, while others deal with daily discomfort. Common symptoms include:

**Frequent urination:** A need to go to the bathroom much more often than usual, both day and night. In severe cases, people may feel the urge up to 60 times a day.

**Urinary urgency:** An intense, sudden, and difficult-to-delay need to urinate.

**Bladder and pelvic pain:** A throbbing or sharp pain that usually worsens as the bladder fills up and improves temporarily after urinating.

**Pain during sex:** Discomfort or pain during and just after sexual intercourse.

For many women—who are diagnosed with IC far more frequently than men—symptoms often flare up or worsen around their menstrual period.

**What Causes It?**

The exact cause of interstitial cystitis is still unknown, though medical researchers suspect it's likely a combination of several factors.

One leading theory is that people with IC have a defect in the protective lining (**epithelium**) of their bladder. A "leak" in this lining might allow toxic substances naturally found in urine to penetrate the bladder wall and irritate the nerves and muscles.

Other potential, but unproven, contributing factors include:

**Autoimmune reactions:** The body's immune system mistakenly attacking the bladder.

**Genetics:** Heredity might play a role in making some people more susceptible.

**Pelvic floor dysfunction:** Weakness or chronic tension in the muscles that support the bladder and pelvic organs.

**Allergies:** Abnormalities in mast cells, which cause allergic symptoms.

**How is it Managed?**

Because the exact cause isn't fully understood, there is no single test to diagnose it, and there is no known cure. Doctors usually diagnose IC by ruling out other conditions, like urinary tract infections (UTIs) or kidney stones.

However, many treatments can help manage the symptoms and significantly improve quality of life:

**Dietary changes:** Eliminating known "bladder irritants" like caffeine, alcohol, artificial sweeteners, carbonated drinks, and highly acidic foods (like citrus or tomatoes).

**Bladder retraining:** Gradually stretching the time between bathroom visits to help the bladder hold more urine.

**Physical therapy:** Working with a specialist to relieve pelvic floor muscle spasms.

**Medications:** Oral drugs to relax the bladder, reduce pain, or help repair the bladder lining.

**Nerve stimulation:** Using mild electrical pulses (like a TENS unit) to relieve pelvic pain and reduce urinary frequency.

**2.Is interstitial cystitis the same as bladder pain syndrome?**

Yes, for all practical purposes, **interstitial cystitis (IC)** and **bladder pain syndrome (BPS)** are the same condition. Today, most healthcare providers and medical organizations group them together and refer to the condition simply as **IC/BPS**.

The two names exist because medical terminology has evolved as doctors have learned more about the condition.

**Why the Name Changed**

**Interstitial Cystitis (The older term):** "Cystitis" literally means inflammation of the bladder. For a long time, doctors believed this condition was purely an inflammatory disease. However, as diagnostic tools improved, they realized a major flaw in the name: the vast majority of patients suffering from the severe pain and urinary symptoms did *not* actually have visible inflammation or structural damage in their bladders.

**Bladder Pain Syndrome (The newer term):** Medical societies introduced BPS (sometimes called Painful Bladder Syndrome) as a more accurate umbrella term. It focuses on the patient's actual experience—a syndrome characterized by chronic pelvic pain, bladder pressure, and an urgent or frequent need to urinate—rather than strictly requiring proof of inflammation.

**The Two Subtypes of IC/BPS**

While the names are used interchangeably to describe the symptoms, doctors will often try to identify which specific physical subtype of IC/BPS a patient has:

**Non-ulcerative IC/BPS:** This accounts for about 90% of cases. Patients experience all the hallmark symptoms, but a visual inspection of the inside of their bladder (cystoscopy) shows no major ulcers. The bladder wall may look normal, or it might show tiny pinpoint hemorrhages called glomerulations.

**Ulcerative IC/BPS:** Making up about 5% to 10% of cases, these patients have a presentation that aligns with the older, strictest definition of "interstitial cystitis." They have visible, bleeding sores on the bladder wall known as **Hunner's lesions** (or Hunner's ulcers).

Regardless of whether a doctor calls it IC, BPS, or IC/BPS, the diagnostic process and the ultimate goal are the same: finding a management plan that relieves the chronic bladder pressure, pain, and urinary frequency.

**3.Is interstitial cystitis the same as a bladder infection?**

No, they are not the same, though they feel incredibly similar.

While **interstitial cystitis (IC)**—often called bladder pain syndrome—and a **bladder infection (Urinary Tract Infection or UTI)** share almost identical symptoms, they are entirely different conditions with completely different causes and treatments.

Think of it this way: a bladder infection is an uninvited guest (bacteria) causing a temporary ruckus, while interstitial cystitis is an ongoing issue with the building itself (chronic inflammation of the bladder lining).

**Key Differences At a Glance**

| **Feature** | **Bladder Infection (UTI)** | **Interstitial Cystitis (IC)** |
| --- | --- | --- |
| **Cause** | Bacterial overgrowth (usually *E. coli*) | Chronic inflammation; exact cause unknown (possibly nerve or lining damage) |
| **Duration** | Acute (short-term, resolves in days) | Chronic (long-term, symptoms last 6+ weeks or come and go) |
| **Urine Test** | **Positive** for bacteria and white blood cells | **Negative** (sterile urine; no bacteria found) |
| **Primary Treatment** | Antibiotics | Diet changes, stress management, physical therapy, specialized medications |
| **Common Triggers** | Sexual activity, holding urine, dehydration | Stress, caffeine, alcohol, citrus, spicy foods, menstruation |

**What is a Bladder Infection?**

A bladder infection is an **acute bacterial infection**. Bacteria enter the urinary tract through the urethra and multiply in the bladder.

**Symptoms:** Sharp burning during urination, intense urgency, frequency, and sometimes cloudy or bloody urine.

**Resolution:** Because it is bacterial, a standard course of antibiotics typically clears the infection and completely resolves the symptoms within a few days.

**What is Interstitial Cystitis (IC)?**

Interstitial cystitis is a **chronic, non-infectious inflammatory condition**. The bladder wall becomes irritated, inflamed, or sensitive, but there are no bacteria present.

**Symptoms:** It mimics a UTI perfectly—causing pelvic pain, pressure, severe urgency, and forcing some people to use the bathroom 15 to 20 times a day.

**The Cycle:** Symptoms often flare up in response to specific triggers like stress, hormonal shifts (like menstruation), or acidic foods and drinks (caffeine, tomatoes, citrus). Because there is no active infection, antibiotics will not provide relief.

**Why Misdiagnosis is Common**

Because the symptoms look identical on paper, many people with IC are initially misdiagnosed and repeatedly treated for "recurrent UTIs."

The single biggest clue for a doctor is the urine culture. If someone experiences UTI-like symptoms that last for more than six weeks, and multiple urine tests keep coming back **negative** for bacteria, a healthcare provider will typically stop prescribing antibiotics and begin evaluating for interstitial cystitis.

**4.Can interstitial cystitis happen even when there is no infection?**

**Yes, absolutely.**

In fact, happening **without an infection** is one of the defining characteristics of interstitial cystitis (IC), which is also widely known as **Bladder Pain Syndrome (BPS)**.

When someone experiences symptoms that feel exactly like a urinary tract infection, but the doctor's tests keep coming back completely clean, IC is often the primary condition suspected.

**IC vs. Urinary Tract Infection (UTI)**

Because the symptoms look almost identical on the surface, IC is frequently confused with a regular bladder infection. However, they are fundamentally different:

**Urinary Tract Infection (UTI):** This is an acute infection caused by bacteria entering the urinary tract. It is diagnosed via a positive urine culture and successfully treated with antibiotics.

**Interstitial Cystitis (IC):** This is a chronic, non-infectious inflammatory condition of the bladder. Urine tests will show **no bacterial growth**, and antibiotics will not relieve the symptoms.

**What Causes IC If There Is No Infection?**

Medical researchers do not yet know the exact, single cause of IC, but they know it is not an infectious disease. Instead, it is believed to be a complex condition likely involving:

**Bladder Lining Defects:** The protective mucus layer (called the GAG layer) that lines the inside of the bladder may be damaged or leaky. This allows toxins and potassium from the urine to penetrate the bladder wall, irritating the underlying nerves and muscles.

**Nerve Misfiring:** Overactive nerve pathways in the pelvis may send constant pain or urgency signals to the brain, even when the bladder holds very little urine.

**Pelvic Floor Dysfunction:** The muscles supporting the bladder, uterus, and bowels can become chronically tight or spasmed, causing deep pelvic pain that feels like it is coming directly from the bladder.

**Immune System Reactions:** Certain immune cells (mast cells) inside the bladder wall may inappropriately release histamine and other inflammatory chemicals, causing swelling and discomfort.

**Common Flare-Up Triggers**

Because the bladder wall in someone with IC is incredibly sensitive, various non-bacterial triggers can cause a sudden spike in pain, pressure, or the frequent urge to urinate:

**Dietary Irritants:** High-acid foods (citrus fruits, tomatoes), caffeine, alcohol, artificial sweeteners, chocolate, and heavy spices.

**Stress:** High physical or emotional stress triggers the nervous system and can cause pelvic floor muscles to clench, worsening symptoms.

**Hormonal Fluctuations:** Many individuals find that their symptoms flare up right before or during their menstrual cycle.

Because antibiotics do not work for IC, management typically focuses on dietary adjustments, pelvic floor physical therapy, stress management, and specific medications designed to help restore the bladder lining or calm down irritated nerves.

**5.Is interstitial cystitis a type of bladder cancer?**

**The Short Answer**

**No**, interstitial cystitis (IC) is not a type of bladder cancer, nor does it increase your risk of developing bladder cancer. They are two entirely distinct conditions, though they can share some overlapping symptoms.

**What is Interstitial Cystitis?**

Interstitial cystitis (often called painful bladder syndrome) is a **chronic, inflammatory bladder condition**.

**The Cause:** The exact cause isn't fully understood, but it's thought to involve defects in the bladder lining, autoimmune responses, or nerve hypersensitivity.

**The Nature:** It is entirely benign (non-cancerous). While it can cause significant long-term discomfort, it does not involve the growth of malignant tumors and does not damage the bladder in a way that leads to cancer.

**What is Bladder Cancer?**

Bladder cancer is a **malignant disease** that occurs when cells in the bladder lining mutate and grow uncontrollably, forming tumors.

**The Cause:** It is strongly linked to cellular mutations, often triggered by environmental factors like cigarette smoke or chronic exposure to certain industrial chemicals.

**Key Differences At a Glance**

| **Feature** | **Interstitial Cystitis (IC)** | **Bladder Cancer** |
| --- | --- | --- |
| **Condition Type** | Chronic inflammatory / pain syndrome | Malignant (cancerous) growth |
| **Primary Symptoms** | Chronic pelvic pain, intense urgency, and very frequent urination | Blood in the urine (often painless), pelvic pain, changes in urination |
| **Life Threatening?** | No, but it significantly impacts quality of life | Can be, if left untreated or if it spreads |
| **Treatment Approach** | Diet changes, physical therapy, oral medications, bladder instillations | Surgery, chemotherapy, immunotherapy, radiation |

**Why the two are sometimes confused:** Because both conditions can cause frequent urination, urgency, and pelvic discomfort, urologists will typically perform tests—such as a cystoscopy (looking inside the bladder with a small camera) or a urine cytology test—to rule out bladder cancer before officially diagnosing someone with interstitial cystitis.

**6.Is interstitial cystitis caused by stress or anxiety?**

**The Short Answer: Trigger, Not Cause**

The short answer is **no**—stress and anxiety do not directly *cause* interstitial cystitis (IC), which is also widely known as bladder pain syndrome (BPS).

However, there is a massive catch. While emotional or mental stress doesn't create the condition from scratch, it is one of the most notorious and powerful triggers for **worsening symptoms and sparking painful "flares."** If you have IC, high stress or anxiety acts like a volume knob that turns the pain and urgency way up.

**Why Stress and IC Feel So Closely Linked**

Even though stress isn't the root cause, it physically alters how your bladder and nervous system behave. Researchers point to a few key reasons why mental stress turns into physical bladder misery:

**The Pelvic Floor "Clench":** When you are anxious, your body naturally tenses up. Many people subconsciously hold this tension in their pelvic floor muscles. Tight, spasming pelvic muscles put direct pressure on the bladder and surrounding nerves, mimicking or multiplying IC pain.

**Nerve Hypersensitivity:** Chronic stress activates your "fight-or-flight" response, flooding your system with cortisol and adrenaline. Over time, this revs up your central nervous system, making your brain much more sensitive to pain signals. What might normally feel like mild bladder fullness gets registered by the brain as a sharp emergency.

**Inflammation and Mast Cells:** Stress triggers the release of inflammatory chemicals (like histamines) from mast cells in the body. If your bladder is already vulnerable, this influx of chemicals can cause localized irritation and swelling in the bladder wall.

**The Vicious Cycle:** Living with unpredictable bladder pain and the constant urge to find a restroom is naturally anxiety-inducing. This creates a frustrating feedback loop: stress triggers a flare, the flare causes anxiety, and the anxiety keeps the flare going.

**What Actually Causes Interstitial Cystitis?**

Medical researchers still don't know the exact root cause of IC, but they believe it is driven by physical and biological factors rather than psychological ones. The leading theories include:

**A "Leaky" Bladder Lining:** A defect in the protective inner lining (the GAG layer) of the bladder. If this layer is compromised, toxic substances in your urine can seep into the deeper layers of the bladder wall, causing chronic irritation.

**Autoimmune Reactions:** The body’s immune system may mistakenly attack the bladder tissue.

**Pelvic Nerve Damage or Trauma:** Previous pelvic surgeries, infections, or physical trauma may leave the nerves hypersensitive.

**Co-occurring Chronic Conditions:** IC frequently overlaps with other chronic inflammatory or pain conditions, such as irritable bowel syndrome (IBS), fibromyalgia, and chronic fatigue syndrome.

**Calming the Storm: Managing Stress-Related Flares**

Because the mind-bladder connection is so strong, stress management is a formal part of medical treatment guidelines for IC. Some of the most effective ways to lower the intensity of a stress-induced flare include:

**Pelvic Floor Physical Therapy:** Working with a specialized physical therapist to learn how to consciously relax and lengthen tight pelvic muscles is one of the most highly recommended treatments for IC.

**Gentle, Low-Impact Movement:** Activities like walking, swimming, or specialized yoga help release endorphins (the body's natural painkillers) and release muscle tension without jarring the bladder.

**Nervous System Regulation:** Practices like diaphragmatic (belly) breathing, mindfulness meditation, or Cognitive Behavioral Therapy (CBT) can help calm an overactive fight-or-flight response.

**Heat Therapy:** A warm bath or a heating pad placed over the lower abdomen or pelvic area can do wonders for physically relaxing tight muscles and soothing nerve pain.

If you are dealing with chronic bladder pain or frequent urgency, partnering with a urologist or a pelvic pain specialist is the best way to rule out other issues (like standard urinary tract infections) and build a personalized management plan.

**7.Does interstitial cystitis usually last for a long time?**

Yes, **interstitial cystitis (IC)**—also frequently called bladder pain syndrome (BPS)—is a **chronic, long-term condition**. For most people who develop it, it lasts for years and can often be lifelong.

Unlike a standard urinary tract infection (UTI), which is caused by bacteria and clears up quickly with antibiotics, IC is an ongoing inflammation or irritation of the bladder wall with no known definitive cure.

**How the Condition Behaves Over Time**

Because IC is chronic, its pattern can vary quite a bit from person to person, usually following a cyclical path:

**Flares and Remissions:** Symptoms rarely stay at the exact same intensity forever. Most people experience "flares" (periods where pain, pressure, and the urgent need to urinate intensify) alternating with periods of "remission" (where symptoms lessen significantly or disappear entirely for weeks, months, or even years).

**Sudden Changes:** In some cases, symptoms may disappear suddenly without an obvious reason, though they can potentially return down the road.

**Impact on the Bladder:** If left unmanaged over a very long period, chronic inflammation can sometimes lead to scarring of the bladder wall, which can reduce the amount of urine the bladder can comfortably hold.

**Long-Term Management**

While IC is typically a long-term journey, it is highly manageable. Many people are able to achieve prolonged periods of remission and drastic symptom reduction through a combination of therapies:

**Dietary Changes:** Avoiding common bladder irritants like caffeine, alcohol, artificial sweeteners, citrus fruits, and highly acidic or spicy foods.

**Pelvic Floor Physical Therapy:** Working with a specialized physical therapist to relax and lengthen tight pelvic floor muscles, which frequently contribute to IC pain.

**Medications:** Doctors may prescribe oral medications (such as antihistamines or bladder protectants) or recommend bladder instillations, where medication is guided directly into the bladder to soothe the lining.

**Stress Management:** Because the nervous system and bladder are closely linked, stress is a notorious trigger for IC flares. Integrating stress-reduction techniques like yoga, meditation, or gentle exercise often plays a massive role in long-term relief.

If you or someone you know is dealing with persistent bladder pain or urgency, consulting a urologist or a pelvic pain specialist is the most effective way to establish an accurate diagnosis and build a personalized plan to keep symptoms under control.

**8.Can interstitial cystitis symptoms come and go over time?**

Yes, **interstitial cystitis (IC) symptoms absolutely come and go over time.**

For most people, IC is characterized by an unpredictable cycle of **flares** (periods when symptoms suddenly intensify) and **remissions** (periods when symptoms lessen or disappear entirely). It is rarely a steadily progressive disease; rather, symptom severity frequently fluctuates, and someone might experience intense discomfort for a few weeks followed by months or even years of feeling completely fine.

**The Cycle of Flares and Remissions**

**1. Flares (Symptom Spikes)**

During a flare-up, symptoms can closely mimic a severe urinary tract infection (UTI), even though no bacterial infection is actually present. These symptoms typically include:

**Intense pelvic pain,** pressure, or discomfort in the lower abdomen and bladder area.

**An urgent, frequent need to urinate** both day and night.

**Pain or burning** during or after sexual intercourse.

**2. Remissions (Symptom-Free Periods)**

Remission periods vary greatly from person to person. Some individuals experience weeks or months with minimal to no discomfort, while others may go years before experiencing another flare-up.

**Common Triggers for Symptom Fluctuations**

When symptoms suddenly return or worsen, it is usually because a specific trigger has irritated the bladder wall, tightened the pelvic floor muscles, or aggravated local nerves. Common triggers include:

**Dietary Irritants:** High-acid foods (like citrus, tomatoes, and vinegar), caffeine, alcohol, artificial sweeteners, carbonated beverages, and spicy foods.

**Hormonal Shifts:** Many individuals note that symptoms flare up during ovulation or in the days right before their menstrual cycle due to hormonal fluctuations.

**Physical Stress:** Long travel or driving (due to vehicle vibration), wearing tight clothing/pants, or certain high-impact exercises.

**Emotional Stress:** While stress does not cause IC, physical and emotional stress can trigger pelvic floor muscle tension, significantly worsening existing symptoms.

**Sexual Activity:** Intimacy can physically irritate the bladder or trigger pelvic muscle spasms, leading to a flare-up that begins 24 to 48 hours later.

**How Long Do Flares Last?**

The duration of a flare-up is highly individual. A flare can last for **a few hours**, stretch across **several weeks**, or occasionally persist for **months** in more severe cases.

Because symptoms vary so widely, keeping a daily symptom and food diary is a highly effective way to identify personal patterns, avoid known triggers, and work with a healthcare provider to map out a management plan.

**9.What are the most common symptoms of interstitial cystitis?**

Interstitial cystitis (IC), also known as **Bladder Pain Syndrome (BPS)**, is a chronic condition characterized by a cluster of urinary and pelvic symptoms. Because it is a "diagnosis of exclusion," its symptoms often mimic those of a urinary tract infection (UTI), even though no infection is present.

The severity of symptoms can range from mild "pressure" to debilitating pain, and they often fluctuate in cycles known as **flares**.

**Core Symptoms**

The "classic triad" of IC symptoms includes:

**Pelvic Pain:** Persistent pain or pressure in the bladder region (between the belly button and the pubic bone). For many, the pain intensifies as the bladder fills and provides brief relief after urinating.

**Urinary Frequency:** Needing to urinate much more often than usual. In severe cases, people may go up to 40–60 times a day.

**Urinary Urgency:** A sudden, intense, and often painful need to urinate immediately, even if the bladder isn't full.

**Other Common Signs**

**Nocturia:** Waking up multiple times throughout the night to urinate.

**Pain During Intercourse:** Often referred to as dyspareunia; for many, the pain can linger for a day or two after sexual activity.

**Small Voiding Volumes:** Frequently passing only small amounts of urine despite the intense urge to go.

**Gender-Specific Differences**

While IC is significantly more common in women, men can also be affected. The location of the pain often differs by sex:

| **For Women** | **For Men** |
| --- | --- |
| Pain in the vulva, vagina, or pelvic area. | Pain in the scrotum, testes, penis, or the area between the scrotum and anus (perineum). |
| Symptoms often worsen during menstruation. | Pain may occur during or after ejaculation. |

**Common "Flare" Triggers**

Symptoms are rarely constant and can be triggered or worsened by:

**Diet:** Specifically acidic foods (tomatoes, citrus), caffeine, alcohol, artificial sweeteners, and spicy foods.

**Stress:** Physical or emotional stress often correlates with increased pain.

**Tight Clothing:** Pressure from tight jeans or waistbands.

**Prolonged Sitting:** Travel or desk work can aggravate pelvic floor tension.

**Note:** If you experience **blood in your urine** or a **high fever** along with these symptoms, seek medical attention promptly. While blood can occasionally appear with IC, these are also hallmark signs of acute infections or other bladder conditions that require immediate testing.

**10.Can interstitial cystitis cause frequent urination?**

Yes, absolutely. Frequent urination is actually one of the primary hallmark symptoms of **interstitial cystitis (IC)**, which is also commonly referred to as bladder pain syndrome (BPS).

In people with a healthy bladder, the bladder expands until it is full and then sends a signal to the brain that it's time to urinate. With IC, these signals get mixed up. You may feel the need to urinate constantly, even if your bladder is only holding a tiny amount of urine.

**How IC Affects Urination**

**Extreme Frequency:** In severe cases, people with IC may feel the need to urinate up to 60 times a day, including multiple times throughout the night.

**Intense Urgency:** The urge to urinate is often sudden and incredibly strong, making it difficult to delay going to the bathroom.

**Reduced Capacity:** Because the bladder wall can become inflamed, stiff, or scarred over time, the bladder may physically not be able to hold as much urine as it normally would.

**Other Common Symptoms**

Frequent urination rarely happens in isolation with IC. It is usually accompanied by:

**Pain and Pressure:** Discomfort, pressure, or chronic pain in the bladder, lower abdomen, or pelvic region.

**Relief Upon Voiding:** The pain typically worsens as the bladder fills up and temporarily gets better immediately after urinating.

**Pain During Sex:** Discomfort or pain during sexual intercourse is also very common.

**Important Note:** The symptoms of interstitial cystitis can perfectly mimic a urinary tract infection (UTI). If you are experiencing frequent urination and bladder pain, it is highly recommended to see a urologist or healthcare provider. They can rule out an infection and help determine if IC or another condition is the underlying cause.

**11.Can interstitial cystitis make someone feel an urgent need to urinate?**

**Yes, absolutely.** A persistent, sudden, and urgent need to urinate is one of the hallmark symptoms of interstitial cystitis (IC), which is also known as bladder pain syndrome.

In fact, the urgency can be so strong that it feels impossible to ignore, even if you only end up passing a very small amount of urine.

**Why the Urge Happens**

Your bladder is a hollow, muscular organ that stores urine. In a normal urinary system, the bladder expands as it fills and eventually sends a nerve signal to your brain letting you know it's time to go.

With interstitial cystitis, the protective lining of the bladder wall becomes irritated and inflamed. This inflammation causes the pelvic nerves to misfire. Instead of waiting until the bladder is full, the nerves send premature, mixed-up signals to the brain, making you feel a constant or sudden urge to empty your bladder even when it is mostly empty.

**Accompanying Symptoms**

Because of how the nerves and inflammation interact, this urgency rarely happens on its own. People with IC usually also experience:

**Extreme frequency:** Needing to urinate many times throughout the day and night (sometimes up to 60 times a day in severe cases).

**Pressure and pain:** Discomfort or pain in the pelvis or lower abdomen that worsens as the bladder fills and is temporarily relieved right after urinating.

**Flare-ups:** Symptoms that come and go, often triggered by stress, certain foods (like caffeine, citrus, or spicy foods), or the menstrual cycle.

**How It Differs From a UTI**

The intense urgency and pelvic pain of interstitial cystitis feel almost exactly like a urinary tract infection (UTI). However, the key difference is that a UTI is caused by bacteria, while IC is not. If someone frequently feels the urgent need to urinate but their urine tests repeatedly come back completely clear of any infection, interstitial cystitis is often a strong suspect.

**12.Can interstitial cystitis cause bladder pressure without severe pain?**

**Yes, Absolutely.**

Interstitial cystitis (IC), which is also frequently called **bladder pain syndrome (BPS)**, does not always present as severe, agonizing pain. For many people, the primary symptom is a persistent, uncomfortable sensation of **bladder pressure, fullness, or a heavy ache** rather than sharp pain.

Because IC exists on a very broad spectrum, it is entirely common to experience significant, life-disrupting bladder pressure and urgency without ever experiencing what you would classify as "severe pain."

**How Pressure Manifests in Interstitial Cystitis**

Clinical definitions of IC/BPS focus on an "unpleasant sensation" in the bladder region rather than strictly requiring intense pain. This sensation is highly subjective and varies wildly from person to person:

**The Filling Cycle:** The pressure typically builds and worsens as the bladder fills with urine. It often temporarily improves or eases up immediately after you urinate.

**The Phantom "Full" Sensation:** You might feel as though your bladder is completely full or heavy, even if it only contains a tiny volume of urine.

**Varying Intensity:** This pressure can range from a mild, background nuisance to an intense, deeply uncomfortable heaviness in the lower abdomen or pelvic region.

**Accompanying Symptoms to Watch For**

Even without severe pain, IC/BPS usually pairs that pressure with other lower urinary tract symptoms, including:

**Urinary Urgency:** A sudden, compelling need to urinate that is difficult to ignore. In IC, this urgency is usually driven by the desire to relieve that uncomfortable pressure or discomfort, rather than a fear of leaking.

**Urinary Frequency:** Feeling the need to go to the bathroom far more often than normal (sometimes 10 or more times a day, as well as waking up multiple times throughout the night).

**Pelvic Tenderness:** Mild discomfort or a raw feeling when pressure is applied to the lower belly, groin, or pelvic floor muscles.

**A Helpful Note:** Because the pressure and urgency of IC can perfectly mimic a Urinary Tract Infection (UTI) or Overactive Bladder (OAB), it is always best to consult a healthcare professional or urologist. If an active infection is ruled out, they can look into IC/BPS and guide you toward targeted relief, such as pelvic floor physical therapy, dietary adjustments (avoiding bladder irritants like caffeine and acids), or specific medications designed to calm the bladder lining.

**13.Why is interstitial cystitis often hard to diagnose?**

Interstitial Cystitis (IC), now frequently referred to as **Bladder Pain Syndrome (IC/BPS)**, is a chronic bladder condition that causes pelvic pain, urinary urgency, and frequency. It is notoriously difficult and time-consuming to diagnose, often taking patients several years and visits to multiple specialists before receiving a correct answer.

**Key Reasons Why IC/BPS is Hard to Diagnose**

**It is a "Diagnosis of Exclusion":** There is currently no single definitive test—such as a specific blood test, biological marker, or imaging scan—that can instantly confirm IC. Instead, doctors must systematically rule out dozens of other conditions before they can safely settle on an IC diagnosis.

**Massive Symptom Overlap:** The hallmark signs of IC (pelvic pressure, pain, and an urgent, frequent need to urinate) perfectly mimic several other common urological and gynecological conditions.

**Early Symptoms Wax and Wane:** In its early stages, IC symptoms are often intermittent. A person might experience a severe flare-up triggered by stress, sexual activity, or certain foods, followed by weeks or months of complete remission. This unpredictable pattern frequently leads to the assumption that the issue was a temporary infection that resolved on its own.

**The "Hidden" Nature of the Disease:** For the vast majority of patients (roughly 90%), a standard look inside the bladder via a cystoscopy reveals a completely normal-looking bladder wall. Only 5% to 10% of patients present with **Hunner's ulcers** (distinct, inflamed patches on the bladder lining), meaning concrete visual evidence is rare.

**An Unknown Root Cause:** Because medical science does not yet fully understand the exact cause or biological mechanism behind IC—theories span from leaky bladder linings to autoimmune dysfunction and pelvic floor nerve sensitivity—developing a targeted diagnostic test remains an ongoing challenge.

**Distinguishing IC From Similar Conditions**

Because symptoms are so similar, IC is routinely misdiagnosed. A key difference lies in the combination of symptoms and laboratory results:

| **Condition** | **Primary Symptoms** | **Key Distinguishing Factors** |
| --- | --- | --- |
| **Interstitial Cystitis (IC/BPS)** | Pelvic pain/pressure, extreme urinary frequency, and urgency. | **Negative urine cultures** (no bacteria found); pain typically increases as the bladder fills and eases temporarily after emptying. |
| **Urinary Tract Infection (UTI)** | Painful burning during urination, urgency, and frequency. | **Positive urine culture** (bacteria present); symptoms resolve rapidly with a course of antibiotics. |
| **Overactive Bladder (OAB)** | Intense urinary urgency, high frequency, and often sudden leaks. | **Typically lacks the severe pelvic pain/pressure** element characteristic of IC; urge incontinence is much more common. |

**The Common Cycle of Misdiagnosis**

**The UTI Trap:** Up to 74% of IC patients are initially told they have a standard Urinary Tract Infection. Because a flare-up feels identical to a UTI, patients are frequently prescribed multiple rounds of antibiotics. It is only after successive urine tests come back sterile (negative for bacteria) despite ongoing pain that doctors begin to look beyond an infection.

In men, IC is frequently misdiagnosed as chronic prostatitis. In women, it is often mistaken for endometriosis, vulvodynia, or recurrent yeast infections.

To eventually arrive at an accurate diagnosis, healthcare providers will take a detailed medical history, request a "bladder diary," and perform diagnostic procedures like a cystoscopy or biopsy primarily to rule out other underlying issues like bladder stones or bladder cancer.

**14.When should someone with possible interstitial cystitis see a doctor?**

Someone suspects they have interstitial cystitis (IC)—also known as bladder pain syndrome (BPS)—they should see a healthcare provider if they experience any of the following scenarios:

**1. Chronic Symptoms Lasting 6 Weeks or More**

The clinical guideline for considering an IC/BPS diagnosis is having bladder or pelvic pain, pressure, or discomfort alongside lower urinary tract symptoms for **at least 6 weeks** without an active infection or other identifiable cause. If these symptoms are persistent, it is time to seek a medical evaluation.

**2. "UTI" Symptoms with Negative Cultures**

If someone frequently feels like they have a urinary tract infection (UTI)—experiencing a sudden urgency, high frequency, or burning during urination—but their urine cultures consistently come back **negative for bacteria**, a doctor should investigate for IC or other chronic bladder conditions.

**3. Severe Disruption of Daily Life**

A medical appointment is warranted if symptoms begin to take a toll on general well-being and quality of life, including:

**Sleep disturbances:** Waking up multiple times a night to urinate (nocturia), leaving them chronically fatigued.

**Painful intimacy:** Feeling deep pelvic pain or discomfort during or after sexual intercourse.

**Social or professional limitations:** Constantly needing to plan daily activities, travel, or work meetings around immediate restroom availability.

**"Red Flag" Symptoms Requiring Immediate Evaluation**

While IC is a painful, chronic condition, certain symptoms are *not* typical for it and require **urgent medical attention** to rule out more serious issues like kidney infections, bladder stones, or structural abnormalities:

**Visible Blood in the Urine (Gross Hematuria):** Microscopic blood can sometimes show up on laboratory tests during a severe IC flare due to bladder wall irritation, but visible blood (turning urine pink, red, or tea-colored) is not a normal feature of IC and always requires a prompt workup.

**Fever, Chills, or Severe Back/Flank Pain:** These are classic signs of an active, potentially spreading kidney infection rather than chronic bladder inflammation.

**Inability to Urinate:** Sudden urinary retention is a medical emergency that requires immediate intervention.

**Unexplained Weight Loss or Night Sweats:** Broad systemic symptoms like these point to an issue outside of isolated bladder pain.

**What to Expect at the Appointment**

Because there is no single definitive test to diagnose interstitial cystitis, it is a **diagnosis of exclusion**. A primary care provider, urologist, or urogynecologist will typically start by ruling out other possibilities.

The evaluation usually involves:

A detailed medical history and a physical/pelvic exam.

A urinalysis and urine culture to check for infection or abnormal cells.

A recommendation to keep a "bladder diary" to track fluid intake and urination frequency.

Potential advanced testing, such as a cystoscopy (using a tiny camera to look inside the bladder), to check for structural issues, inflammation, or rare bladder ulcers (Hunner's ulcers).

Seeking medical advice early can help establish an accurate diagnosis and kickstart a management plan, which often begins with non-invasive strategies like dietary adjustments, stress management, and pelvic floor physical therapy.

**15.What symptoms with interstitial cystitis should be checked urgently?**

While interstitial cystitis (IC)—also known as bladder pain syndrome—is a chronic, uncomfortable, and often frustrating condition, it is not inherently life-threatening. However, because its standard symptoms (pelvic pain, urgency, and frequent urination) overlap with acute medical emergencies, it is critical to know when a symptom is actually a "red flag" requiring urgent medical attention.

If you or someone else experiences any of the following symptoms alongside or instead of typical IC discomfort, seek immediate medical care at an urgent care or emergency room:

**1. Signs of a Kidney or Systemic Infection**

Because IC is an inflammatory condition rather than a bacterial one, it does not cause systemic symptoms. The following are strong indicators that a bacterial urinary tract infection (UTI) has developed and potentially spread to the kidneys or bloodstream:

**High fever or shaking chills:** A fever means your body is actively fighting an infection.

**Severe back, side, or flank pain:** IC pain is concentrated in the lower abdomen and pelvis. Pain higher up in your back or sides points directly toward your kidneys.

**Nausea and vomiting:** Feeling sick to your stomach, especially when paired with a fever, indicates the infection is impacting your entire system.

**2. Visible Blood in the Urine (Gross Hematuria)**

While microscopic amounts of blood can sometimes show up on a lab test during an IC flare (especially if Hunner's ulcers are present), **noticeable pink, red, or tea-colored urine**, or passing visible blood clots, is a major red flag. This requires an urgent evaluation to rule out severe bacterial infections, bladder stones, or other serious structural conditions.

**3. Acute Urinary Retention**

IC is notorious for making you feel like you have to empty your bladder constantly. However, if you suddenly **cannot pass urine at all** despite a strong, painful urge and a feeling of extreme fullness, this is a medical emergency. Urinary retention can cause severe bladder stretching and kidney damage if not relieved quickly.

**4. A Sudden Change in Pain Integrity**

**Excruciating or localized abdominal pain:** If you experience an abrupt escalation of pain that feels entirely different or significantly worse than your worst IC flares, it needs an immediate evaluation. This helps rule out non-bladder emergencies like a ruptured ovarian cyst, testicular torsion, appendicitis, or a passing kidney stone.

**The Golden Rule for IC Patients:** Having chronic interstitial cystitis does not make you immune to standard UTIs, kidney stones, or other pelvic issues. It can be easy to dismiss severe discomfort as "just another bad flare," but new, worsening, or systemic symptoms should never be ignored. When in doubt, a quick trip to an urgent care facility or ER is the safest approach.

**16.Should blood in the urine be checked if someone has interstitial cystitis symptoms?**

**Yes, absolutely.** If someone is experiencing symptoms of interstitial cystitis (IC)—also known as bladder pain syndrome—and blood is found in their urine, it should always be evaluated by a healthcare professional.

While interstitial cystitis can cause significant bladder pain, pressure, and urgency, blood in the urine (hematuria) is not a standard hallmark of the condition and requires a proper medical workup to rule out other potential causes.

**Why Testing is Essential**

Interstitial cystitis is largely a diagnosis of exclusion, meaning doctors must rule out other conditions before confirming it. When blood appears in the urine, clinicians must investigate because it can be a sign of an entirely different or co-existing medical issue.

**1. Visible vs. Microscopic Blood**

**Gross Hematuria (Visible Blood):** If the urine looks pink, red, or tea-colored, this is considered an urgent concern. Visible blood is **highly atypical** for standard interstitial cystitis and strongly points toward another underlying issue that needs immediate evaluation.

**Microscopic Hematuria:** If the blood is invisible to the naked eye and only detected during a lab urinalysis, it is less immediately alarming but still requires a formal investigation.

**2. Ruling Out Alternative Causes**

Blood in the urine can be a symptom of several conditions that require distinct treatments, including:

**Urinary Tract Infections (UTIs) or Hemorrhagic Cystitis:** A severe bacterial infection can cause the bladder lining to bleed.

**Bladder or Kidney Stones:** Crystals passing through the urinary tract can frictionally irritate the lining, causing noticeable bleeding and sharp pain.

**Urologic Malignancies:** In some cases, blood in the urine (especially painless visible bleeding) can be an early warning sign of bladder, kidney, or prostate cancer. This is particularly critical to rule out for individuals over 50 or those with a history of smoking.

**The Link Between IC and Blood**

While blood should never automatically be blamed on IC, there are specific scenarios where the two intersect:

**Bladder Wall Irritation:** During a severe IC flare, the protective lining of the bladder can become highly inflamed, cracked, or fragile. This intense irritation can cause tiny amounts of **microscopic** blood to leak into the urine.

**Hunner's Lesions:** Around 10% of people with interstitial cystitis have Hunner's lesions, which are inflammatory, ulcer-like sores on the bladder wall. These lesions are more prone to bleeding, especially after the bladder is distended or during a severe flare-up.

**What to Expect at the Doctor**

If blood is detected, a primary care physician or a urologist will typically recommend a series of diagnostic steps based on the patient's age and risk factors:

**Urinalysis and Urine Culture:** To check for bacteria and confirm the concentration of red and white blood cells.

**Imaging:** A renal ultrasound or CT urogram may be ordered to look at the kidneys and ureters.

**Cystoscopy:** A procedure where a small camera is inserted into the bladder to directly inspect the lining for lesions, inflammation, stones, or abnormal tissue growth.

**Bottom Line:** Never assume that blood in the urine is just a standard part of an interstitial cystitis flare. It is a clinical signal that always warrants professional medical confirmation to ensure safety and get the correct treatment.

**17.Should fever with bladder pain be treated as interstitial cystitis?**

**No, fever with bladder pain should not be treated as interstitial cystitis.**

In fact, a fever is a major red flag that points *away* from interstitial cystitis and strongly suggests an active, potentially serious infection that requires immediate medical evaluation.

Here is why treating these symptoms as interstitial cystitis (IC) could be dangerous, and what is likely happening instead.

**Why Fever Rules Out Interstitial Cystitis**

Interstitial cystitis (also known as Bladder Pain Syndrome) is a chronic, **non-infectious** bladder condition. While it causes severe bladder pressure, pain, and a frequent urgency to urinate, it does not cause a fever.

By definition, a diagnosis of IC requires that all other conditions—especially bacterial infections—be completely ruled out. Because IC is not an infection, treating it with standard IC protocols (like dietary changes or bladder instillations) while ignoring a fever will leave an active infection completely untreated.

**What Fever and Bladder Pain Usually Indicate**

When bladder pain is paired with a fever, your immune system is actively fighting off an invader. This combination usually points to one of the following conditions:

**An Acute Urinary Tract Infection (UTI):** While standard UTIs don't always cause a fever in adults, a fever means the infection is escalating.

**A Kidney Infection (Pyelonephritis):** If a bladder infection travels up into the kidneys, it becomes a medical emergency. This is heavily characterized by a high fever, chills, lower back or side (flank) pain, nausea, and vomiting.

**Prostatitis:** In men, an infection or inflammation of the prostate can cause severe bladder-like pain, urinary difficulties, and a fever.

**What You Should Do Next**

If you or someone you know is experiencing bladder pain accompanied by a fever, **see a healthcare provider promptly** (such as at an urgent care or emergency room).

**Get a Urine Culture:** A doctor needs to test the urine to identify the exact bacteria causing the infection.

**Start Antibiotics:** If an infection is confirmed, you will need a course of prescription antibiotics to clear it and prevent it from spreading to the kidneys or bloodstream.

*Disclaimer: I am an AI, not a doctor. Fever combined with pelvic or bladder pain is a clear signal from your body that it needs professional medical attention right away.*

**18.What should someone do if interstitial cystitis pain suddenly becomes much worse?**

A sudden, severe spike in interstitial cystitis (IC) pain—often called a flare—can be incredibly distressing. When a flare hits, the immediate goal is to calm the bladder nerves, relax the pelvic floor, and reduce the acidity of your urine.

Here are the most effective strategies urologists and IC specialists recommend to help break a sudden pain cycle:

**Immediate At-Home Relief**

**Baking soda and water:** Drink a glass of water mixed with 1/2 to 1 teaspoon of baking soda. This acts as a natural antacid to make your urine less acidic, which can quickly take the sting and burn out of urination. *(Note: Skip this if you are on a strict sodium-restricted diet or have high blood pressure).*

**Temperature therapy:** Apply a heating pad, hot water bottle, or cold pack to your lower abdomen or perineum (the area between the genitals and anus). Heat helps relax cramping pelvic muscles, while cold can help numb severe nerve pain. Experiment to see which your body responds to best.

**Warm sitz bath:** Soaking in a warm, plain bath can significantly help relax tight pelvic floor muscles that often spasm during a flare. Avoid using bubble baths, soaps, or bath bombs, as these can irritate the urethra.

**Pelvic floor relaxation:** Rest in positions that stretch and release the pelvic floor. Lie on your back and bring your knees to your chest, or try a gentle, wide-legged yoga squat. **Avoid Kegels** or any pelvic floor strengthening exercises right now, as tightening those muscles will likely make spasms and pain worse.

**Dilute your urine:** Drink plenty of plain water. While it might seem counterintuitive to drink more when you have severe urinary urgency, highly concentrated urine is deeply irritating to an inflamed bladder wall. Diluting it brings relief.

**Remove Hidden Triggers**

**Ditch tight clothing:** Immediately remove belts, tight jeans, pantyhose, or shapewear. Anything that compresses your abdomen or pelvic region restricts blood flow and can severely worsen nerve pain.

**Stop dietary irritants:** Cut out the "Four Cs" immediately: **c**itrus, **c**affeine, **c**arbonation, and **c**hocolate. You should also strictly avoid tomatoes, spicy foods, alcohol, and artificial sweeteners until the flare entirely passes.

**Medications**

**Use your "rescue" meds:** If your doctor has previously prescribed specific medications for flares—such as urinary analgesics (like phenazopyridine), antispasmodics, or muscle relaxers—take them at the very onset of the pain.

**Over-the-counter relief:** Standard NSAIDs like ibuprofen or naproxen can help reduce systemic inflammation and take the edge off the pain.

**When to Contact a Doctor**

Because an IC flare feels almost exactly like a urinary tract infection (UTI), it is very common to mistake one for the other.

You should reach out to your healthcare provider if:

You develop a fever, chills, or see blood in your urine.

The pain is entirely unmanageable at home.

You suspect it might actually be an infection.

Your doctor can run a quick urine culture to rule out a UTI. If it is a severe IC flare that won't break, they can also provide stronger medical interventions, such as a **bladder instillation** (where soothing medications like lidocaine are placed directly into the bladder via a catheter) to stop the pain directly at the source.

**19.What should someone do if they cannot urinate while having interstitial cystitis symptoms?**

If you or someone else is experiencing an absolute inability to urinate for several hours despite having the urge and a full bladder, this is known as **acute urinary retention** and is a **medical emergency**.

People with Interstitial Cystitis (IC) can experience this when severe pain and inflammation cause the pelvic floor muscles to go into a spasm, effectively clamping the urethra shut.

**What to Do Immediately**

**Seek emergency medical care.** Go to the nearest Emergency Room or Urgent Care. Do not wait for a regular doctor's appointment. If left untreated, urine backing up can cause severe, permanent damage to the bladder walls or the kidneys.

**Stop drinking fluids.** Do not try to "flush" the system or trigger urination by drinking more water right now. This will only add volume to an already full bladder and drastically increase the pain.

**Do not force or strain.** Pushing or bearing down hard to force the urine out can backfire by causing your pelvic floor muscles to clench even tighter.

**What to Try While Waiting for Care**

If you are preparing to go to the clinic or waiting for a ride, you can try these steps to encourage the muscles to relax. **Do not use these as a substitute for getting medical help.**

**Take a warm bath.** Immersing your lower body in warm water is one of the most effective ways to relax severe pelvic floor spasms. It is entirely safe (and common) to release urine directly into the bath water if the muscles finally let go.

**Use a warm compress.** If a bath isn't an option, place a heating pad or a warm, damp towel over your lower abdomen or perineum.

**Change your posture on the toilet.** Sit down, lean forward, and rest your elbows on your knees. This specific angle helps naturally relax the pelvic floor structure.

**Practice deep belly breathing.** Focus on taking slow, deep breaths into your stomach. When you exhale, consciously try to "drop" and relax your pelvic area rather than pushing.

**Turn on the faucet.** The sound of running water can sometimes create a psychological trigger that helps the brain signal the bladder to release.

**What to Expect at the Doctor**

Medical professionals can provide immediate, safe relief. They will typically use a quick, non-invasive ultrasound on your lower abdomen to see exactly how much urine is trapped in the bladder. If it is full, they will gently insert a small tube (a catheter) to drain the urine, which relieves the pain and pressure instantly.

*Disclaimer: I am an AI, not a doctor. Because acute urinary retention can lead to serious organ damage, please prioritize seeing a healthcare professional over relying on home remedies.*

**20.What type of doctor usually treats interstitial cystitis?**

Interstitial cystitis (IC), also known as bladder pain syndrome, is most commonly treated by specialists who focus on the urinary tract and pelvic floor.

The primary doctors who diagnose and treat this condition include:

**Urologists:** These are physicians who specialize in the diagnosis and treatment of diseases of the urinary tract in both men and women, as well as the male reproductive system. They are typically the first specialists recommended for IC.

**Urogynecologists:** Also known as Female Pelvic Medicine and Reconstructive Surgery (FPMRS) specialists, these are gynecologists or urologists with advanced training in treating pelvic floor disorders and urinary issues specifically in women.

**Additional Specialists**

Because interstitial cystitis can be complex and often overlaps with other conditions, a multidisciplinary approach is frequently used. A care team might also include:

**Pelvic Floor Physical Therapists:** Specializing in relaxing and strengthening the pelvic muscles, which can significantly reduce IC pain.

**Pain Management Specialists:** Doctors who help develop strategies to manage chronic pelvic pain if standard treatments do not provide enough relief.

**Gastroenterologists or Rheumatologists:** Involved if the patient has overlapping chronic conditions like Irritable Bowel Syndrome (IBS) or autoimmune disorders.

**21.How do doctors usually diagnose interstitial cystitis?**

Diagnosing interstitial cystitis (IC)—also commonly referred to as **Bladder Pain Syndrome (BPS)**—can be a detailed process. Because there is no single, definitive test that can instantly confirm IC, doctors primarily treat it as a **diagnosis of exclusion**. This means their main goal initially is to rule out other conditions that cause similar symptoms, such as urinary tract infections (UTIs), overactive bladder, bladder cancer, or pelvic floor dysfunction.

According to major urological guidelines, the typical diagnostic approach follows a tiered framework.

**1. The Core Assessment**

For most straightforward presentations, a doctor can form a clinical diagnosis using three fundamental steps:

**Detailed Medical History:** The doctor will evaluate your symptom patterns. The hallmark of IC/BPS is pelvic pain, pressure, or discomfort that is directly related to bladder filling (getting progressively worse as the bladder fills and often temporarily improving after urinating). They will also look at urinary frequency (how often you go) and urgency (the sudden, intense need to go).

**Physical Examination:** A doctor will perform an abdominal and pelvic exam. In women, this often includes a pelvic exam to check for tenderness in the pelvic floor muscles or bladder neck. In men, a digital rectal exam may be used to assess the prostate and pelvic floor.

**Urinalysis and Urine Culture:** This is a crucial step to check for white blood cells, red blood cells, and bacteria. A negative urine culture is required to prove that the symptoms are not being caused by an active bacterial UTI.

**2. Symptom Tracking Tools**

To get a clearer picture of how the bladder is behaving day-to-day, a doctor may ask a patient to utilize specific tracking tools:

**Bladder Diary:** Patients track what they drink, how often they urinate, and estimated volumes for a few days. This helps doctors distinguish between true frequency/urgency and other baseline habits.

**Symptom Questionnaires:** Standardized forms, like the O'Leary-Sant Interstitial Cystitis Symptom Index, help quantify the severity of the symptoms and provide a baseline to measure future treatment success.

**3. Advanced Testing (For Unclear or Complicated Cases)**

If a patient's symptoms are unusual, if they have a history of smoking (which increases bladder cancer risks), or if initial treatments fail, a urologist may order more specialized tests:

**Cystoscopy:** A doctor inserts a thin tube with a camera into the urethra to look at the lining of the bladder. While it can't definitively "prove" someone has IC, it is highly useful for ruling out bladder stones or tumors. It is also used to look for **Hunner’s lesions**—distinct, inflammatory ulcers on the bladder wall that are present in about 5% to 10% of IC patients.

**Urodynamic Testing:** This test involves filling the bladder with water via a small catheter to measure pressure and find out how much liquid the bladder can hold before triggering the urge to urinate. It helps rule out nerve-related bladder issues or muscle spasms.

**Bladder Biopsy:** If abnormal tissue is seen during a cystoscopy, a tiny sample may be taken. This is purely to rule out malignancy, not to actively identify IC.

️**An Important Note on Outdated Tests**

You may still read about the **Potassium Sensitivity Test (PST)**, where a doctor instills a potassium solution into the bladder to see if it causes severe pain. Modern urological guidelines **no longer recommend** this test. It is highly painful, can trigger severe symptom flare-ups, and does not provide accurate enough results to alter a patient's treatment path.

**22.Is there one test that can prove someone has interstitial cystitis?**

**No, there is no single test that can definitively prove someone has Interstitial Cystitis (IC)**—which is also widely referred to as Bladder Pain Syndrome (BPS).

Instead, IC is primarily a **diagnosis of exclusion**. This means healthcare providers rule out other conditions that cause identical symptoms—such as urinary tract infections (UTIs), bladder stones, overactive bladder, or bladder cancer—before officially diagnosing IC.

**How Providers Investigate IC**

Because there is no simple blood or urine marker for IC, the diagnostic process relies heavily on checking a person's symptom history and using baseline tests to eliminate other possibilities:

**Symptom Evaluation:** Providers look for the core clinical hallmarks of IC, which include chronic pelvic pain or pressure, a persistent and intense urgency to urinate, and high urinary frequency (sometimes dozens of times a day).

**Urinalysis and Urine Culture:** These standard lab tests check for bacteria, white blood cells, or red blood cells. This is essential to ensure that a simple, hidden bacterial infection isn't the root cause of the discomfort.

**Physical and Pelvic Exams:** A doctor will check for pelvic floor muscle tenderness, structural abnormalities, or localized pain points to see if the issue is muscular or anatomical.

**The Role of Cystoscopy and "Hunner Lesions"**

While a routine evaluation is usually enough for an uncomplicated case, a doctor may sometimes order a **cystoscopy** (inserting a tiny camera through the urethra to view the inside of the bladder). While a cystoscopy cannot rule out IC, it does provide the closest thing to a "proof" in a small subset of patients:

**Hunner Lesions:** In roughly 5% to 10% of individuals with IC, a cystoscopy reveals distinct, inflamed red patches or ulcers on the bladder wall called Hunner lesions. Finding these lesions essentially confirms a diagnosis of IC.

However, **the absence of these lesions does not rule out IC**. The vast majority of people with IC have a completely normal-looking bladder lining during a routine cystoscopy.

**Other Supportive Diagnostic Tools**

If a diagnosis is tricky or complex, a urologist or urogynecologist might use additional tools to paint a clearer picture:

**Bladder Diary:** Keeping a detailed log for a few days tracking fluid intake, urination frequency, and pain levels.

**Urodynamic Testing:** A test that measures how well the bladder holds and releases urine under pressure, helping to rule out nerve or primary bladder muscle malfunction.

**Biopsy:** If unusual patches are seen during a cystoscopy, a tiny tissue sample may be examined under a microscope purely to rule out malignancy.

If you or someone you care about is managing persistent pelvic discomfort or a frequent, urgent need to go, partnering with a urologist or a pelvic pain specialist is the most effective way to systematically rule out look-alike conditions and establish an accurate management plan.

**23.Why do doctors need to rule out other problems before diagnosing interstitial cystitis?**

Doctors must rule out other conditions because interstitial cystitis (IC)—also known as bladder pain syndrome (BPS)—is fundamentally a **diagnosis of exclusion**.

There is no single, definitive test (like a blood test or a distinct imaging scan) that can instantly confirm a person has IC. Instead, a diagnosis is reached by confirming a patient has the characteristic symptoms *and* ensuring that no other underlying disease is responsible for them.

The step-by-step process of elimination is critical for several specific reasons:

**1. Massive Symptom Overlap**

The hallmark symptoms of IC—chronic pelvic pain, urinary urgency (the sudden, intense need to go), and urinary frequency (going many times a day and night)—are not unique to IC. They perfectly mimic several other common medical conditions. If doctors diagnosed IC based on symptoms alone, the risk of misdiagnosis would be incredibly high.

**2. Differentiating Easily Treatable Conditions**

Many conditions that look like IC are highly treatable, and doctors want to exhaust those possibilities first.

**Urinary Tract Infections (UTIs):** A standard bacterial infection is the most common mimic. UTIs are quickly cleared up with standard antibiotics, whereas IC is a chronic condition that does not respond to antibiotics.

**Overactive Bladder (OAB):** OAB causes a sudden, involuntary contraction of the bladder muscle, leading to urgency and frequency. However, unlike IC, OAB typically does not cause the severe, chronic bladder pain or pressure that worsens as the bladder fills.

**3. Catching Serious Underlying Pathologies**

Some conditions that mimic IC symptoms are serious and require immediate, specific intervention. Doctors must rule these out to ensure patient safety:

**Bladder or Urinary Tract Cancer:** Bladder tumors can cause pelvic irritation, frequent urination, or blood in the urine. This must be definitively ruled out early through a urinalysis or a cystoscopy (looking inside the bladder with a camera).

**Bladder Stones:** Hard masses of minerals in the bladder can cause severe pain and irritation that mimics IC but requires surgical removal or breaking up.

**4. Gender-Specific Overlaps**

The pelvic region is crowded with complex anatomy, and problems in neighboring organs can feel exactly like a bladder issue:

**In Women:** Conditions like endometriosis, uterine fibroids, or pelvic floor muscle dysfunction can cause chronic pelvic pain and pressure that overlaps with IC.

**In Men:** Chronic prostatitis or chronic pelvic pain syndrome shares nearly identical symptoms with IC and must be evaluated separately.

**5. Guiding the Right Treatment Plan**

Managing IC often involves long-term lifestyle changes, specialized physical therapy, oral medications, or bladder instillations. If a patient actually has a hidden infection, an anatomical obstruction, or a nerve issue, IC treatments will be ineffective, and the actual underlying condition could get progressively worse.

By carefully eliminating other possibilities first, healthcare providers ensure they aren't treating a chronic syndrome when a straightforward cure exists—or worse, missing a serious condition that requires urgent medical intervention.

**24.Why do doctors check urine when interstitial cystitis is suspected?**

When interstitial cystitis (IC)—also known as Bladder Pain Syndrome (BPS)—is suspected, a doctor’s first step is almost always to order a urine test.

The primary reason for this is that IC is a **diagnosis of exclusion**. Because there is no single, definitive test that can instantly confirm IC, doctors must systematically rule out other conditions that cause identical symptoms (such as pelvic pain, urgency, and frequent urination).

**1. Ruling Out a Urinary Tract Infection (UTI)**

The symptoms of IC mimic a standard bladder infection almost perfectly. A doctor will order a **urinalysis** and a **urine culture** to check for white blood cells and specific bacteria.

**The distinction:** If bacteria are present, it’s a UTI, which is treated with antibiotics. If the symptoms last for six weeks or more and the urine cultures repeatedly come back completely sterile (no infection), IC becomes a strong suspect.

**2. Checking for Hematuria (Blood in the Urine)**

A urinalysis checks for the presence of red blood cells.

**The distinction:** While microscopic amounts of blood can occasionally appear in IC patients due to severe bladder irritation, visible or significant blood in the urine can be a red flag for other serious issues. Doctors need to rule out bladder or kidney stones, structural issues, or bladder tumors before landing on an IC diagnosis.

**3. Screening for Metabolic and Kidney Conditions**

Urine tests look for markers like glucose (sugar) and high levels of protein.

**The distinction:** High glucose levels can indicate undiagnosed diabetes, which frequently causes increased urinary frequency and urgency. High protein levels can point toward kidney dysfunction rather than a localized bladder issue.

**4. Conducting Urine Cytology (If Necessary)**

If a patient has a history of smoking, is over a certain age, or has unexplained blood in their urine, a doctor may order a urine cytology test.

**The distinction:** This lab test examines the cells shed by the bladder lining into the urine to rule out precancerous or cancerous cells.

**Summary:** A urine test doesn't prove you *have* interstitial cystitis; rather, it proves you *don't* have a temporary infection, stones, diabetes, or other conditions that require entirely different treatments.

**25.Is looking inside the bladder always needed to diagnose interstitial cystitis?**

**No, It Is Not Always Required**

According to modern medical guidelines—including those from the American Urological Association (AUA)—a **cystoscopy (looking inside the bladder with a small camera) is not necessary to diagnose interstitial cystitis / bladder pain syndrome (IC/BPS) in straightforward cases.**

For the majority of people, a diagnosis can be safely and accurately established through a clinical evaluation without invasive testing.

**How IC/BPS Is Typically Diagnosed**

Instead of immediately looking inside the bladder, healthcare providers usually focus on a non-invasive clinical assessment:

**Symptom Tracking:** Evaluating whether you have experienced bladder or pelvic pain, pressure, or discomfort—often coupled with a frequent or urgent need to urinate—for at least **6 weeks**.

**Excluding Other Conditions:** Ensuring the symptoms aren't caused by more common issues. This involves a physical/pelvic exam and basic lab tests (like urinalysis and urine cultures) to rule out urinary tract infections (UTIs), bladder stones, or sexually transmitted infections (STIs).

**Baseline Documentation:** Keeping a baseline bladder diary to track fluid intake, daily voiding frequency, and pain patterns.

**When a Cystoscopy *Is* Needed**

While not required for a routine diagnosis, a doctor will recommend a cystoscopy in specific scenarios:

**Diagnostic Uncertainty:** If your symptoms are highly complicated, atypical, or fail to respond to standard initial treatments.

**Ruling Out Structural Mimics:** To directly check for and rule out bladder cancer (especially important if there is unexplained blood in the urine or a history of smoking), bladder stones, or foreign bodies.

**Suspecting Hunner Lesions:** These are distinct, highly inflamed ulcers on the bladder wall that occur in a specific subset of IC patients (most commonly in adults over 50). Finding these lesions is crucial because they require specialized, targeted treatments like cauterization (fulguration) or localized steroid injections.

**Why the Guidelines Changed**

In the past, looking for pinpoint bleeding (glomerulations) via a cystoscopy with hydrodistention (stretching the bladder with fluid) was considered the "gold standard" for diagnosis. Experts moved away from making this mandatory for two main reasons:

**It Is Not Completely Accurate:** Studies revealed that completely healthy individuals without any bladder pain can display these same bleeding points, while some patients with severe, classic IC symptoms have perfectly normal-looking bladder linings.

**Patient Comfort:** The procedure is invasive and can temporarily flare up pelvic pain. Avoiding it when a clinical diagnosis is clear protects patients from unnecessary discomfort.

**26.Can food or drinks make interstitial cystitis symptoms worse?**

**Yes, absolutely.** For many people with interstitial cystitis (IC) — also known as bladder pain syndrome — diet plays a massive role. In fact, modifying what you eat and drink is often the first line of defense for managing flare-ups.

When you have IC, the protective lining of your bladder is often compromised. This means that when highly acidic or irritating compounds from your diet are filtered into your urine, they can directly inflame the bladder wall, leading to increased pain, pressure, and that urgent, frequent need to urinate.

**The Most Common Triggers**

While everyone's body is different, urologists often point to a group of well-known bladder irritants. A helpful way to remember the biggest culprits is the **"Four Cs"**:

**Caffeine:** Coffee, tea, energy drinks, and even chocolate.

**Citrus:** Oranges, grapefruits, lemons, limes, and their juices.

**Carbonation:** Soda, sparkling water, and seltzers.

**Vitamin C:** High-concentration supplements or heavily fortified foods.

**Other frequent triggers include:**

**Tomatoes:** Very acidic and a notoriously common trigger (this includes ketchup, marinara sauce, and tomato paste).

**Spicy Foods:** Chili peppers, hot sauces, and heavy spices.

**Alcohol:** Beer, wine, and liquor are highly irritating and dehydrating.

**Artificial Sweeteners & Additives:** Aspartame, saccharin, and MSG (monosodium glutamate).

**Fermented or Pickled Foods:** Vinegar, pickles, sauerkraut, and soy sauce.

**Bladder-Friendly Alternatives**

You don't have to cut out everything. There are plenty of foods that are generally safe and gentle on the bladder:

**Fruits:** Pears, blueberries, and melons (like honeydew and watermelon).

**Vegetables:** Broccoli, carrots, green beans, asparagus, and squash.

**Proteins:** Lean chicken, turkey, beef, eggs, and fish.

**Grains:** Rice, oats, quinoa, and pasta.

**Beverages:** Plain still water, chamomile or peppermint herbal teas, and milk (or non-dairy alternatives like almond milk).

**Finding Your Personal Triggers**

It's important to know that **not every trigger food will bother every person with IC.** A food that causes a severe flare-up for someone else might be perfectly fine for you.

Because of this, many doctors recommend keeping a daily "food and symptom diary." If you are currently experiencing a lot of pain, you might also benefit from an **elimination diet** — where you remove the most common irritants for a few weeks to let your bladder calm down, and then slowly reintroduce them one by one to see exactly which ones cause your symptoms to return.

**27.Which drinks commonly bother people with interstitial cystitis?**

While triggers can vary significantly from person to person, people with interstitial cystitis (IC) generally find that beverages high in **acid**, **caffeine**, or **carbonation** cause the most severe bladder flares.

Here are the most common offenders and why they cause problems:

| **Beverage Type** | **Why It Irritates the Bladder** | **Bladder-Friendly Alternative** |
| --- | --- | --- |
| **Coffee & Caffeinated Tea** | High in both acid and caffeine. Even decaf coffee often triggers flares because the acid remains. | Chamomile or peppermint tea |
| **Carbonated Drinks** | The carbonation process creates carbonic acid. Sodas also often contain bladder-irritating artificial sweeteners. | Plain, still water (or water infused with cucumber) |
| **Alcohol** | Highly dehydrating and often contains histamines and sulfites (especially in wine and beer). | Mocktails made without citrus or carbonation |
| **Citrus Juices** | Orange, grapefruit, lemon, and lime juices are incredibly acidic and directly inflame the bladder lining. | Pear or blueberry juice (diluted with water) |
| **Energy & Sports Drinks** | Packed with caffeine, artificial dyes, and sweeteners that can overwhelm a sensitive bladder. | Naturally flavored water or milk (if tolerated) |

**The Cranberry Juice Trap:** Because cranberry juice is a famous home remedy for urinary tract infections (UTIs), many people with IC mistakenly drink it hoping for relief. However, cranberry juice is highly acidic. For a bladder already inflamed by IC, drinking it is often compared to pouring battery acid on an open wound.

**Finding Your Triggers**

Because IC is highly individualized, you might tolerate some of these drinks while others cause immediate pain. The most effective way to identify your specific triggers is to do an **elimination diet**—cutting out all common triggers for a few weeks until your symptoms calm down, and then slowly reintroducing them one by one while keeping a bladder diary to track your symptoms.

**28.Can coffee make interstitial cystitis symptoms worse?**

Yes, coffee is one of the most notorious dietary triggers for interstitial cystitis (IC) flare-ups.

For many people with IC, a morning cup of coffee leads to increased pelvic pain, pressure, and frequent trips to the bathroom. This happens for two primary reasons:

**Why Coffee Triggers Symptoms**

**High Acidity:** Coffee is naturally very acidic. In a healthy bladder, a protective mucous layer prevents acidic urine from irritating the tissue. In someone with IC, this lining is often compromised, leaving the bladder wall inflamed or dotted with tiny sores (Hunner's ulcers). When acidic urine hits this inflamed tissue, it causes a burning sensation—much like pouring lemon juice on an open cut.

**Caffeine Content:** Caffeine is both a **diuretic** (which increases the amount of urine your kidneys produce) and a **stimulant**. This combination forces your bladder to fill up faster while simultaneously triggering the bladder muscles to spasm, worsening feelings of sudden urgency and frequency.

Inflamed bladder wall in IC. Cleveland Clinic

**The Decaf Myth**

A common misconception is that switching to decaffeinated coffee will solve the problem. While decaf removes the stimulant effect that causes spasms and frequency, **it does not remove the acid**.

Decaf coffee is almost as acidic as regular coffee, meaning it can still cause significant burning and bladder pain for IC patients.

**How to Manage the Coffee Craving**

If you love coffee but it's aggravating your IC, you might not have to give it up entirely. Many patients find ways to manage the flare-ups with a few adjustments:

**Try low-acid beans:** Some coffee brands specifically process their beans to reduce acidity. Dark roasts also tend to be naturally lower in acid than light roasts.

**Switch to cold brew:** The cold-brewing process extracts far less acid from the coffee grounds compared to traditional hot brewing.

**Use an acid neutralizer:** Many IC patients have success taking an over-the-counter acid reducer (like calcium glycerophosphate, often sold as Prelief) right before taking their first sip. It neutralizes the acid in the drink before it ever reaches your bladder.

**Wean off slowly:** If you decide to cut coffee out completely, do it gradually over a week or two. Quitting caffeine cold turkey can cause severe withdrawal headaches and fatigue, which only adds to the stress of an IC flare.

**29.Does everyone with interstitial cystitis need to avoid the same foods?**

No. People with interstitial cystitis (IC) do not all need to avoid the same foods. The idea of a universal "IC Diet" that bans a massive list of foods forever is a myth—and one that can make eating unnecessarily stressful.

While dietary changes are a highly effective way to manage IC (also known as Bladder Pain Syndrome), triggers are strictly individualized. A food that causes a severe flare-up for one person might be perfectly fine for someone else.

Here is how food actually affects IC and how to figure out what works for you.

**Why Triggers Vary So Much**

You have likely heard that "acidic foods" are the enemy of IC. However, the science is more complex than "acid in equals acid out." Your body has sophisticated buffering systems that regulate the pH of your urine.

When a food triggers bladder pain, it is often because specific compounds in that food—not just its acidity level—irritate the sensitive bladder lining, activate nerve endings, or trigger an inflammatory response. Because everyone's bladder lining and sensitivities are different, your triggers will be unique to you.

**The Most Common Culprits**

While there is no universal forbidden list, certain foods and drinks are statistically more likely to irritate an IC bladder. When trying to identify what is bothering you, these are the usual suspects:

**Caffeinated and carbonated drinks:** Coffee (even decaf, which contains other irritating compounds), tea, energy drinks, and sodas.

**Acidic fruits and vegetables:** Citrus fruits (oranges, lemons, grapefruits), tomatoes, and tomato-based sauces.

**Spicy foods:** Chili peppers, hot sauces, and heavy curries.

**Artificial sweeteners:** Aspartame, saccharin, and sucralose (often found in diet sodas and sugar-free snacks).

**Alcohol:** Beer, wine, and liquor can dehydrate the body and irritate the bladder lining.

**Certain additives:** Monosodium glutamate (MSG) and heavy preservatives found in processed foods.

**How to Find Your Personal Triggers**

Urologists and dietitians generally recommend a personalized approach rather than permanent, sweeping restrictions:

**Start a symptom diary:** Track everything you eat and drink alongside your bladder symptoms for a few weeks. Sometimes the trigger isn't what you expect, or you'll find that *timing* matters (e.g., a small cup of coffee is fine with food, but not on an empty stomach).

**Try a temporary elimination diet:** Remove the most common trigger foods from your diet entirely for 2 to 3 weeks to see if your symptoms improve.

**Reintroduce foods systematically:** Bring the eliminated foods back one at a time, spacing them out by a few days. If your bladder flares up, you've identified a personal trigger. If nothing happens, that food is safe to keep enjoying.

**30.How can someone with interstitial cystitis find out which foods bother their bladder?**

Because Interstitial Cystitis (IC) is so individualized, there is no universal "IC Diet." A food that causes agonizing pain for one person might be completely fine for you.

The gold standard for identifying your specific triggers is a structured **elimination diet**. This process involves removing known irritants to let your bladder heal, and then slowly bringing them back to see how your body reacts.

Before starting, it helps to know what you are looking out for. Dietitians often refer to the most common IC triggers as the **"Four Cs"** — Coffee, Citrus, Chocolate, and Carbonated drinks.

Here is a breakdown of the worst offenders and their bladder-friendly alternatives:

| **Category** | **High-Risk Triggers** | **Bladder-Friendly Alternatives** |
| --- | --- | --- |
| **Beverages** | Coffee, soda, alcohol, energy drinks | Water, chamomile tea, peppermint tea |
| **Fruits** | Citrus, pineapple, strawberries | Pears, blueberries, melon, sweet apples |
| **Vegetables** | Tomatoes, onions, hot peppers | Carrots, cucumbers, broccoli, potatoes |
| **Flavorings** | Vinegar, artificial sweeteners, MSG | Olive oil, garlic, mild herbs (basil, thyme) |

**The Cranberry Trap:** Many people mistake an IC flare for a standard UTI and drink cranberry juice to treat it. Because cranberries are highly acidic, they are actually one of the most severe triggers for IC and will make the pain significantly worse.

To find out exactly which of these foods bother you, follow this step-by-step process:

**1. The Elimination Phase：**2 to 4 weeks。

Stick strictly to a core list of bladder-friendly foods. Completely remove the "High-Risk Triggers" from your diet. The goal is to let your bladder calm down and establish a symptom-free (or low-symptom) baseline so you can accurately judge your reactions later.

**2. The Challenge Phase：**Test one food at a time。

Once your symptoms have improved and are stable, pick **one** food from your restricted list to test. Eat a small amount of it on Day 1. If you don't have a flare, try a normal portion on Day 2. If you still feel fine, that food is likely safe for you. Wait 3 full days before testing a new food, as flares can sometimes be delayed.

**3. The Flare Reaction：**Stop if symptoms return。

If you experience increased frequency, urgency, or pelvic pain during a challenge, stop eating that food immediately and write it down as a personal trigger. Wait until your symptoms completely return to your baseline before you start your next food challenge.

It helps immensely to keep a daily food and symptom journal during this process. Sometimes the trigger isn't just the food itself, but the *amount* — you might find you can tolerate a slice of tomato on a sandwich, but a bowl of tomato soup will cause a flare.

**31.Should people with interstitial cystitis drink less water to urinate less often?**

It is incredibly tempting to drink less water when you have interstitial cystitis (IC) — it seems logical that less fluid going in means fewer painful trips to the bathroom. However, **restricting your water intake is generally a bad idea and can actually make your symptoms much worse.**

Here is why:

**The Concentrated Urine Problem**

When you don't drink enough water, your urine becomes highly concentrated. Concentrated urine is deeply acidic and filled with metabolic waste products.

For a healthy bladder, this isn't a major issue. But for someone with IC, the protective mucosal lining of the bladder is often compromised or inflamed. When highly acidic, concentrated urine hits that raw bladder wall, it acts like salt in a wound. This severe irritation triggers **more** pain, bladder spasms, and an even stronger, more frequent urge to urinate — completely defeating the purpose of drinking less.

**Finding the Sweet Spot**

While you shouldn't dehydrate yourself, you also shouldn't chug massive amounts of water. Gulping down large quantities rapidly overfills and stretches the bladder, which also causes pain and urgency.

The goal is fluid management: finding the balance where your urine is dilute enough that it doesn't burn, but your bladder isn't constantly overwhelmed by high volumes of liquid.

**Best Practices for Hydrating with IC**

**Sip, don't chug:** Spacing out your water intake throughout the day is crucial. Try sipping small amounts continuously rather than drinking whole glasses at once. This allows a steady, gentle flow of dilute urine to enter the bladder without stretching it too quickly.

**Check your urine color:** Your urine is your best real-time indicator. You are aiming for a pale, light yellow. If it is dark yellow or amber, you are dehydrated and your urine is likely irritating your bladder. If it is completely clear like water, you may be over-hydrating and forcing unnecessary bathroom trips.

**Stick to plain water:** Many IC patients have specific dietary triggers. Be sure your hydration comes from plain water. Avoid caffeinated drinks (coffee, tea), carbonated beverages, alcohol, artificial sweeteners, and highly acidic juices (like citrus or cranberry juice, which is notoriously painful for an IC bladder).

**Track your intake:** While a common baseline is roughly 6 to 8 glasses (1.5 to 2 liters) a day, everyone's ideal volume is different. Keeping a voiding diary to track how much you drink and when you feel symptoms can help you find your personal baseline.

*Note: Always discuss your fluid intake and symptom management with your urologist. They can help you determine the specific daily fluid volume that is safest and most comfortable for your body.*

**32.Can emotional stress make interstitial cystitis symptoms worse?**

Yes, emotional stress is widely recognized as a significant factor that can worsen interstitial cystitis (IC), also known as bladder pain syndrome (BPS). While stress does not cause the condition, it is a common trigger for "flares"—periods when symptoms become significantly more intense.

**Why Stress Impacts IC Symptoms**

Research indicates that there is a complex, two-way connection between the brain and the bladder, often involving the following mechanisms:

**Nervous System Sensitivity:** Stress triggers the body’s "fight or flight" response, which can increase overall sensitivity in the nervous system. This may heighten your perception of bladder pain and urgency.

**Hormonal Changes:** Stress causes the release of hormones like cortisol. Chronic elevation of these hormones can affect your immune response, potentially leading to increased inflammation in the bladder.

**Muscle Tension:** Emotional stress often leads to physical tension, particularly in the pelvic floor muscles. Tight pelvic muscles can put extra pressure on the bladder, worsening sensations of urgency and pain.

**Biological Triggers:** Some studies suggest that stress can lead to the activation of mast cells in the bladder, which may release chemicals that cause local inflammation and irritation.

**Managing the Connection**

Because stress can intensify symptoms, many healthcare providers emphasize stress management as a key part of an IC treatment plan. Techniques that patients often find helpful include:

**Mindfulness and Relaxation:** Practices like meditation, deep breathing exercises, or progressive muscle relaxation can help calm the nervous system and reduce the "fight or flight" response.

**Physical Therapy:** Specialized pelvic floor physical therapy can help release the muscle tension that often builds up during periods of high stress.

**Gentle Movement:** Activities such as restorative yoga, Tai Chi, or walking can help reduce stress and improve pelvic floor health without putting excessive pressure on the bladder (avoid high-impact exercises during a flare).

**Cognitive Behavioral Therapy (CBT):** This can be highly effective for managing the psychological impact of chronic pain, helping to break the cycle where pain causes stress, which then causes more pain.

**Routine and Sleep:** Prioritizing good sleep hygiene is essential, as sleep deprivation can significantly lower your body’s ability to cope with both physical and emotional stress.

*Disclaimer: I am an AI, not a doctor. If you are experiencing frequent or severe flares, it is important to discuss these patterns with a urologist or a healthcare provider who specializes in interstitial cystitis. They can help you develop a personalized management plan that addresses both the physical and emotional aspects of the condition.*

**33.What daily habits may help reduce interstitial cystitis flare-ups?**

Managing Interstitial Cystitis (IC), or Bladder Pain Syndrome, is deeply personal because triggers vary significantly from person to person. Because IC involves chronic inflammation of the bladder lining, daily habits are focused on two major goals: protecting the bladder wall from irritating chemicals in urine, and preventing the surrounding pelvic floor muscles from clenching.

Normal vs. Inflamed Bladder Lining. Mayo Clinic

**What to note:** In a healthy bladder, a protective coating keeps waste products in urine from touching the tissue. With IC, that coating is compromised. Acidic or highly concentrated urine directly touches the raw bladder wall, causing intense pain and urgency—much like pouring lemon juice on an open scrape.

Incorporating these targeted daily habits can help stabilize your bladder and reduce the frequency of flare-ups:

**1. Calibrate Your Hydration (The "Lemonade" Rule)**

**Dilute your urine:** Dehydration leads to highly concentrated urine that acts as a direct irritant to the bladder wall. Aim to drink enough water so your urine is a consistent pale yellow—resembling light lemonade.

**Avoid over-hydrating:** Chugging excessive amounts of water to keep your urine completely clear can over-stretch the bladder, triggering frequency and urgency spasms. Sip steadily throughout the day instead.

**2. Outsmart the "Four Cs" and Track Your Meals**

Dietary choices are among the most common flare triggers. While individual food sensitivities vary, minimizing the famous bladder irritants can establish a calmer baseline:

**Caffeine:** Coffee, energy drinks, and green or black teas.

**Citrus:** Oranges, grapefruits, lemons, limes, and their juices.

**Carbonation:** Sparkling water, seltzers, and sodas.

**Chili / Spicy Foods:** This includes hot peppers, heavy spices, and tomatoes or tomato-based sauces.

*The Log Habit:* Keep a daily food journal alongside a brief symptom log. Food-related flares can take anywhere from 30 minutes to 3 days to manifest, making a written history essential for spotting patterns.

**3. Down-Regulate Your Nervous System**

Stress is a major physical trigger for IC. When your body enters a stress response, it instinctively tightens the pelvic floor muscles. Tight muscles compress an already sensitive bladder, causing a painful cycle of spasms.

Dedicate 10 minutes a day to active relaxation techniques like diaphragmatic breathing (belly breathing). Deep belly breathing sends a physical signal to release and drop the pelvic floor muscles.

Practices like journaling, gentle meditation, or reading can help down-regulate your central nervous system.

**4. Swap Out Restrictive Clothing**

Anything that applies sustained pressure to your abdomen or pelvic region can aggravate IC symptoms. Tight jeans, leggings, shapewear, and pantyhose are frequent culprits.

Opt for loose-fitting trousers, joggers, skirts, or dresses to eliminate external mechanical pressure on your pelvis.

**5. Adjust Your Intimacy Habits**

If physical intimacy tends to trigger a flare, small procedural shifts can protect vulnerable nerves and muscles:

Use a generous amount of a clean, hypoallergenic, scent-free lubricant (such as pure coconut oil or aloe-based formulas) to minimize friction.

Always urinate immediately after sex to flush the urethra and reset bladder tone.

Apply a warm heating pad or a cooled gel pack to the perineum for 15 to 20 minutes post-intimacy to prevent the pelvic floor from locking into a spasm.

**34.Can interstitial cystitis be cured completely?**

Currently, there is no complete, permanent cure for interstitial cystitis (IC), also known as bladder pain syndrome (BPS). It is considered a chronic condition.

However, a lack of a "cure" does not mean a lack of relief. In about 50% of cases, symptoms improve significantly or go into complete remission on their own over time. For others, the condition is highly manageable through a trial-and-error process to find the right combination of therapies.

Comparison of a normal vs. inflamed bladder lining. Glycologix

Because IC causes the protective lining of the bladder to become inflamed and irritated, treatments focus on soothing this lining, relaxing the surrounding muscles, and blocking pain signals.

**Standard Treatment Approaches**

Doctors typically start with the most conservative options and move to more advanced therapies if symptoms persist:

**Lifestyle and Dietary Changes:** Identifying and avoiding food triggers is often the first step. Many patients find relief by avoiding the "Four Cs": **c**arbonated beverages, **c**affeine (including chocolate), **c**itrus products, and high concentrations of vitamin **C**. Stress management is also critical, as stress directly triggers flare-ups for many.

**Pelvic Floor Physical Therapy:** IC often causes the muscles around the bladder to spasm or tighten defensively. A specialized physical therapist can help stretch and relax these pelvic floor muscles. *(Note: Standard Kegel exercises are generally avoided, as strengthening already-tight muscles can make IC pain worse).*

**Oral Medications:**

**Amitriptyline:** A tricyclic antidepressant taken at low doses that relaxes the bladder and blocks pain signals.

**Pentosan polysulfate sodium (Elmiron):** The only FDA-approved oral drug specifically for IC. It is believed to work by restoring the damaged inner surface of the bladder, protecting it from irritating substances in urine.

**Antihistamines:** Medications like hydroxyzine can block histamine release in the bladder, reducing inflammation and the frequent urge to urinate.

**Bladder Instillations:** Often called a "bladder bath," this involves using a catheter to fill the bladder with a liquid medication—such as dimethyl sulfoxide (DMSO), heparin, or lidocaine—which is held for about 15 minutes before being released. This delivers anti-inflammatory and numbing medicine directly to the irritated tissue.

**Procedures:** If non-invasive treatments do not work, doctors may recommend **hydrodistention** (stretching the bladder with water under anesthesia, which can "reset" pain nerves and increase capacity), **neuromodulation** (using mild electrical pulses to the sacral nerves to regulate bladder function), or **Botox injections** into the bladder muscle to reduce pain and spasms.

Because IC presents differently in everyone, a treatment that works perfectly for one person might not work for another. It generally requires working closely with a urologist to build a customized management plan.

**35.What is the main goal of interstitial cystitis treatment?**

Because there is currently no definitive cure for interstitial cystitis (IC)—often called bladder pain syndrome—the primary goal of treatment is **symptom management and restoring your quality of life**.

Instead of trying to make the condition vanish completely, therapies are tailored to make it manageable so it doesn't run your life. Treatment plans are highly personalized and generally focus on four core objectives:

**Pain Relief:** Minimizing the chronic pelvic pain, pressure, and intense discomfort that defines the condition.

**Reducing Urgency and Frequency:** Calming the bladder muscles and nerve signals to decrease both the sudden, intense urge to urinate and the total number of bathroom trips throughout the day and night.

**Identifying Flare Triggers:** Pinpointing specific dietary or lifestyle factors—like stress, artificial sweeteners, citrus, or caffeine—that cause symptoms to spike.

**Restoring Daily Function:** Reclaiming control over your sleep, work, and social life, which are frequently disrupted by severe bladder symptoms.

**A Quick Nuance:** Because IC affects everyone differently, what works perfectly for one person might not work for another. Finding the right management plan—which often combines dietary changes, pelvic floor physical therapy, oral medications, or bladder instillations—usually takes some patient trial and error.

**36.How long can interstitial cystitis treatment take to start working?**

Because there is no single "cure" for interstitial cystitis (IC) — and because no one treatment works for everyone — the timeline for finding relief can vary widely. It often requires a process of trial and error, and the time it takes to see results depends entirely on the specific treatment approach.

Here is a breakdown of how long standard IC treatments typically take to start working:

**Oral Medications**

**Pentosan polysulfate sodium (Elmiron):** This is the only oral medication approved by the FDA specifically to treat IC. It is believed to work by restoring the protective mucosal lining of the bladder. It is a slow-acting drug: it typically takes **2 to 4 months** before you begin to feel pain relief, and up to **6 months** to experience a noticeable decrease in urinary frequency.

**Antihistamines and Antidepressants:** Medications like hydroxyzine (an antihistamine) or amitriptyline (a tricyclic antidepressant) are often prescribed off-label to relax the bladder, block nerve pain, and reduce inflammation. These can begin to help control symptoms within a **few weeks**, though finding the correct dosage may take some adjustment.

**Bladder Instillations**

**DMSO (Dimethyl sulfoxide):** This is a liquid medication placed directly into the bladder via a catheter, where it helps relax the bladder and alleviate inflammation. It is typically administered once a week for 6 to 8 weeks. Research suggests that patients who respond to DMSO generally begin to see significant improvement around the **6-week mark**.

**Physical Therapy**

Working with a physical therapist who specializes in the pelvic floor can relieve the muscle tension, tenderness, and connective tissue restrictions that often accompany and exacerbate IC. Depending on the severity of the muscle dysfunction, it usually takes **several weeks to a few months** of consistent therapy to experience sustained relief.

**Diet and Lifestyle Modifications**

Eliminating known bladder irritants (like coffee, alcohol, artificial sweeteners, citrus, and spicy foods) is often the first line of defense. If your symptoms are heavily diet-driven, you might notice a reduction in flare-up intensity within **a few weeks** of strictly avoiding your personal triggers.

**Key insight:** Because these treatments work in different ways — some calm immediate nerve pain, while others take months to physically heal the bladder lining — urologists frequently combine them. For instance, a patient might use diet modifications and physical therapy for shorter-term management while waiting the 4 to 6 months for a drug like Elmiron to reach its full therapeutic effect.

**37.Why do doctors often try interstitial cystitis treatments step by step?**

Doctors use a stepwise (or tiered) approach to treat interstitial cystitis (IC) because there is no universal cure, and the condition varies wildly from person to person. The goal is to achieve maximum symptom relief while exposing you to the lowest possible medical risk.

While the trial-and-error process can be incredibly frustrating when you are in pain, this methodical approach protects you from unnecessary side effects and invasive procedures.

**Why the "Ladder" Approach is Necessary**

**Balancing Risk and Reward:** Treatment options for IC range from completely harmless (like avoiding acidic foods) to irreversible (like surgically removing the bladder). A doctor will not jump to surgery or heavy daily medications if dietary adjustments and pelvic floor exercises can resolve 80% of the pain.

**The Heterogeneity of IC:** Medical experts increasingly believe IC is not just one disease, but a cluster of different conditions that cause similar symptoms. For one person, the pain might be driven by pelvic floor muscle spasms; for another, it might be a defect in the protective mucous lining of the bladder itself. What puts one patient into remission might do absolutely nothing for another.

**Minimizing Medication Side Effects:** The oral medications used for IC (like tricyclic antidepressants, antihistamines, or Elmiron) can cause systemic side effects ranging from drowsiness to, in rare cases, retinal issues. The stepwise approach ensures you only take these if conservative methods aren't enough.

**The Typical Treatment Ladder**

Treatment generally progresses from simple lifestyle changes to complex medical procedures, stopping as soon as an effective combination is found.

| **Step** | **Approach** | **Common Interventions** | **Risk / Invasiveness** |
| --- | --- | --- | --- |
| **1. Behavioral** | Lifestyle and self-care | Diet modification (cutting caffeine/citrus), pelvic floor physical therapy, stress management | Lowest |
| **2. Oral Medications** | Systemic symptom control | Amitriptyline (for nerve pain), antihistamines, Elmiron (to protect the bladder lining) | Low to Moderate |
| **3. Instillations** | Localized bladder therapy | Catheter delivery of soothing "cocktails" (lidocaine, heparin, DMSO) directly into the bladder | Moderate |
| **4. Procedures** | Advanced medical intervention | Hydrodistention (stretching the bladder under anesthesia), Botox injections, sacral nerve stimulators | High |
| **5. Major Surgery** | Irreversible structural changes | Urinary diversion or cystectomy (bladder removal) | Highest (Absolute last resort) |

**A Modern Shift: Multi-Modal Care**

It is worth noting that modern urology guidelines (like those updated by the American Urological Association in 2022) have shifted slightly.

While the philosophy is still "start low and go slow," doctors no longer force patients to rigidly fail one step entirely before allowing them to try the next. Today, doctors prefer a **simultaneous, multi-modal approach**. This means they might start you on Step 1 and Step 2 at the exact same time—for example, prescribing an oral nerve medication while simultaneously having you start pelvic floor physical therapy and an IC-friendly diet—to get your pain under control faster.

**38.Is it safe to take over-the-counter pain medicine for interstitial cystitis?**

Yes, it is generally safe to take over-the-counter (OTC) pain medicine for mild to moderate interstitial cystitis (IC) flare-ups, as long as you follow the recommended dosage and only use them for short-term relief.

However, because IC is a chronic condition, OTC pain relievers are best used as a temporary bridge rather than a long-term solution. They help manage the discomfort but do not treat the underlying bladder wall inflammation or nerve sensitivity.

**Common OTC Options for IC**

**NSAIDs (Ibuprofen, Naproxen, Aspirin):** Non-steroidal anti-inflammatory drugs are often the first choice because they reduce both pain and inflammation. Examples include Advil, Aleve, and Motrin.

**Acetaminophen (Tylenol):** This is a solid alternative for pain relief, especially if you have a history of stomach ulcers or gastrointestinal issues, as it is gentler on the stomach than NSAIDs. Keep in mind that while it relieves pain, it does not actively reduce inflammation.

**Antihistamines (Zyrtec, Claritin, Benadryl):** While not traditional painkillers, OTC allergy medications block histamines. Since IC flare-ups can sometimes be triggered by mast cells releasing histamines in the bladder, these can help reduce irritation, frequency, and nighttime voiding for some people.

**Important Safety Warnings**

OTC medications are highly accessible, but taking them regularly to manage chronic IC pain carries risks:

**Stomach and Kidney Risks:** Taking NSAIDs frequently or for prolonged periods can lead to stomach lining irritation, ulcers, and kidney damage.

**Liver Risks:** Acetaminophen is processed by the liver. Taking more than the recommended daily limit, or combining it with alcohol, can cause severe liver damage.

**Urinary Numbing Agents (like AZO):** OTC medicines containing **phenazopyridine** (often marketed for UTIs) can temporarily numb the urinary tract. While some IC patients use them during severe flares, they are strictly intended for very short-term use (usually no more than 2 days) to avoid kidney damage, and they will turn your urine bright orange.

**When to Step Up Treatment**

If you find yourself reaching for OTC pain medicine regularly, it is time to consult a urologist or a pelvic pain specialist. Long-term reliance on OTC painkillers carries significant health risks, and a doctor can offer specialized treatments designed specifically for the root causes of IC. These often include:

**Prescription oral medications:** Drugs like Elmiron (pentosan polysulfate sodium, which helps repair the bladder lining), tricyclic antidepressants (like amitriptyline) to calm overactive bladder nerves, or prescription-strength antihistamines (like hydroxyzine).

**Bladder instillations:** Soothing medications placed directly into the bladder via a catheter.

**Pelvic floor physical therapy:** To address the pelvic muscle tension and spasms that almost always accompany and worsen IC pain.

**39.Are antibiotics helpful for interstitial cystitis when there is no infection?**

No, antibiotics are not helpful for interstitial cystitis (IC) in the absence of a bacterial infection, and they are not an appropriate treatment for the condition.

Beaufort Memorial Hospital

Because the symptoms of IC—pelvic pain, urgency, and frequent urination—feel exactly like a urinary tract infection (UTI), it is incredibly common for patients to be prescribed rounds of antibiotics before an accurate diagnosis is made. However, using them when your urine cultures show no bacteria will not improve the disease.

Pacific Endometriosis and Pelvic Surgery

Inflammation in Interstitial Cystitis. Glycologix

**Why Antibiotics Don't Work for IC**

Interstitial cystitis (also known as Bladder Pain Syndrome) is a chronic inflammatory disorder, not a bacterial infection.

Beaufort Memorial Hospital

In a healthy bladder, a protective mucosal layer lines the inside of the organ, shielding the tissue from the natural toxins and acidity of urine. In a bladder with IC, this protective layer is often faulty or compromised. When toxins in the urine seep through that barrier, they cause intense irritation and inflammation in the bladder wall.

Premier Medical Group

Antibiotics are designed to do exactly one thing: kill bacteria. Because there is no underlying bacteria causing the irritation in IC, the antibiotics have nothing to fight and cannot repair the compromised bladder lining.

Premier Medical Group

**The Illusion of Antibiotic Relief**

Many IC patients report that they actually *did* feel better while taking antibiotics, which understandably leads to confusion. If you have experienced this, it is likely due to one of three reasons:

Pacific Endometriosis and Pelvic Surgery

**Co-prescribed painkillers:** Antibiotics are frequently prescribed alongside urinary tract analgesics (such as phenazopyridine, commonly known as Azo or Pyridium). The analgesic temporarily numbs the bladder, providing rapid pain relief, while the antibiotic gets the unearned credit.

Pacific Endometriosis and Pelvic Surgery

**Natural symptom cycles:** IC is characterized by "flare-ups" that naturally wax and wane. A painful flare may coincidentally begin to resolve on its own just as you start an antibiotic course.

Pacific Endometriosis and Pelvic Surgery

**Anti-inflammatory side effects:** Certain antibiotics possess mild anti-inflammatory properties. They might temporarily reduce some of the swelling in the bladder wall, but they are an inefficient and temporary way to achieve this.

**What the Medical Guidelines Say**

The American Urological Association (AUA) explicitly states in its treatment guidelines that **long-term or repeated oral antibiotics should not be offered** for interstitial cystitis.

Medscape Reference

Taking antibiotics when you do not have an active infection carries significant risks. It disrupts your gut and vaginal microbiomes—which can actually worsen pelvic discomfort and trigger yeast infections—and contributes to antibiotic resistance. Antibiotics should only be used if an actual, cultured UTI is occurring alongside the IC.

**Standard Treatments for IC**

Instead of antibiotics, urologists use a multi-tiered approach focused on symptom management and reducing inflammation. This usually includes a combination of:

**Oral medications:** Nerve pain medications (like amitriptyline), antihistamines to calm mast cells in the bladder, or pentosan polysulfate (Elmiron), which is designed to help rebuild the bladder's protective coating.

Urology Care Foundation

**Bladder instillations:** A process where soothing medications (like heparin or lidocaine) are placed directly into the bladder via a catheter to coat and protect the lining.

American Urological Association

**Pelvic floor physical therapy:** Chronic bladder pain often causes the surrounding pelvic floor muscles to tightly clench, creating a secondary source of severe pain that physical therapy can help release.

Health Answers by Pfizer

**Dietary modifications:** Identifying and eliminating "trigger foods" that irritate the bladder, most commonly caffeine, alcohol, citrus, tomatoes, and spicy foods.

**40.Should someone keep taking antibiotics for interstitial cystitis if urine tests show no infection?**

Generally speaking, no. If a urine culture comes back negative, continuing to take antibiotics for interstitial cystitis (IC) isn't recommended.

The main reason is that IC is a chronic inflammatory condition, not a bacterial infection. Antibiotics are designed specifically to target and kill bacteria. When no bacteria are present, the medication won't solve the underlying problem. Taking them unnecessarily doesn't help the bladder, and it can introduce unwanted side effects like gut disruption, yeast infections, and antibiotic resistance.

As you can see in the diagram below, the discomfort from IC doesn't stem from a temporary bacterial invasion, but rather from irritation, redness, and structural inflammation within the bladder wall itself.

Normal vs. Interstitial Cystitis Bladder Surface. Glycologix

**The most important rule here:** A person should never stop a prescribed antibiotic course cold turkey without speaking to their healthcare provider first. Even if the latest test is clear, the doctor might have a specific clinical reason for the timeline—such as finishing out a course for an initial suspected infection or waiting on a more specialized, slow-growing culture.

**What actually helps manage IC?**

Since antibiotics won't touch the root cause of an IC flare, standard medical guidelines focus on calming the bladder lining and the surrounding nerves instead:

**Dietary tracking:** Pinpointing and cutting back on common bladder irritants like caffeine, alcohol, citrus fruits, artificial sweeteners, and tomatoes.

**Pelvic floor physical therapy:** A huge portion of people with IC suffer from tight pelvic floor muscles. Specialized physical therapy can help relax these muscles and drastically reduce pain levels.

**Targeted oral medications:** Doctors often utilize non-antibiotic options like nerve-calming medications (such as amitriptyline), antihistamines to reduce bladder mast cell activation (like hydroxyzine), or therapies designed to help protect the bladder lining.

**41.Why might eye checks be needed for some interstitial cystitis medicines?**

If a doctor recommends regular eye checks during interstitial cystitis (IC) treatment, it is almost certainly due to a specific oral medication called **pentosan polysulfate sodium**, widely known by the brand name **Elmiron**.

Elmiron is a common oral medication approved specifically to treat the bladder pain and discomfort caused by IC, but long-term use has been linked to a unique type of retinal toxicity.

**The Risk: Pigmentary Maculopathy**

The primary concern is a condition called **pigmentary maculopathy**. This is a disease that alters the cells in your retina—specifically the **macula**, which is the part of the eye responsible for your clear, central vision (the vision you use for reading, driving, and recognizing faces).

The risk is closely tied to how long someone takes the medication and the total cumulative dose. While it most commonly appears after several years of daily use, it can sometimes develop sooner.

**Early Warning Signs**

Eye specialists monitor patients closely because this toxicity can cause noticeable, disruptive changes to your sight, including:

**Prolonged dark adaptation:** Taking an unusually long time for your eyes to adjust when walking into a dim or dark room.

**Difficulty reading:** Text may look blurry, or pieces of words might seem to disappear.

**Distorted vision:** Straight lines (like doorframes or blinds) might look wavy, curved, or broken.

**Muted colors:** Colors might look faded or "washed out."

**Why Regular Screening is Critical**

The most important reason for routine eye checks is that **this specific eye damage can be irreversible**. Furthermore, medical studies have shown that the retinal changes can sometimes continue to progress even *after* a patient stops taking the medication entirely.

Because early retinal damage doesn't always cause immediate symptoms, specialized imaging is the only way to catch changes before permanent vision impairment occurs.

**What the Exams Look For**

A standard vision test with an eye chart isn't enough to catch this issue early. Instead, an ophthalmologist will use high-tech imaging of the back of the eye, such as:

**Optical Coherence Tomography (OCT):** A scan that takes highly detailed, cross-sectional pictures of the retinal layers.

**Fundus Autofluorescence (FAF):** A specialized scan that uses light to reveal areas of stress or cell death in the retinal pigment.

Current medical guidelines recommend getting a detailed baseline eye exam before starting the medication (or within the first 6 months of treatment), followed by regular periodic or annual screenings. If any early signs of macular changes are discovered, urologists and ophthalmologists will typically work together to safely transition the patient to alternative IC management therapies.

**42.Should people with interstitial cystitis talk to a doctor before using supplements?**

**Yes, absolutely.** Anyone with interstitial cystitis (IC) or bladder pain syndrome should consult a healthcare provider or a registered dietitian who specializes in pelvic pain before starting any new vitamins or supplements.

While certain supplements can be game-changers for managing IC symptoms and soothing the bladder, the wrong ones can trigger excruciating flares. Because the protective mucosal lining of the bladder (the GAG layer) is often compromised in people with IC, substances that a healthy bladder can easily handle may act like acid poured on an open wound.

Here is why medical guidance is so critical for this specific condition.

**The Unique Risks of Supplements for IC**

**The Acid Factor (Vitamins C and B):** Standard multivitamins are one of the most common accidental triggers for IC flares. **Vitamin C (ascorbic acid)** and **B-complex vitamins** (especially B6) are highly acidic and are cleared from the body through the urine. As they sit in a highly sensitive bladder, they can cause severe burning and urgency.

**The Cranberry Trap:** Because IC symptoms mimic urinary tract infections (UTIs), many people instinctively reach for cranberry pills or juice. Cranberries contain high levels of quinic, malic, and citric acids. For an IC bladder, cranberry is a massive irritant and will often make symptoms much worse.

**Hidden Pill Ingredients:** Over-the-counter supplements often use binders, artificial colors, or artificial sweeteners (like aspartame or saccharin) to make the pills palatable or easier to manufacture. Artificial sweeteners are well-documented mast cell degranulators that provoke nerve irritation and bladder flares.

**Prescription Interactions:** IC is often treated with a multi-layered approach, using medications like Elmiron (pentosan polysulfate sodium), amitriptyline, or hydroxyzine. Supplements can interact with these medications or compound their side effects, making a doctor's oversight essential.

**How Common Supplements Affect IC**

When you do speak with your doctor, they will likely evaluate supplements based on whether they irritate the bladder or help rebuild and soothe it. Here is how some of the most common supplements break down:

| **Supplement** | **Typical Purpose** | **IC-Specific Consideration** |
| --- | --- | --- |
| **Vitamin C & B6** | General health / immunity | **High Risk:** Highly acidic; frequent triggers for severe flares. |
| **Cranberry Extract** | UTI prevention | **High Risk:** Extremely acidic; strongly discouraged for IC. |
| **Prelief (Calcium Glycerophosphate)** | Dietary acid reduction | **Helpful:** Often taken right before meals to neutralize acid in trigger foods. |
| **Aloe Vera (Freeze-dried)** | Bladder lining support | **Helpful:** Believed to provide molecules that help restore the bladder's protective coating. |
| **L-Arginine** | Blood flow / inflammation | **Helpful:** May improve pelvic blood flow and reduce spasms, but can interact with blood pressure meds. |
| **Magnesium** | Muscle relaxation | **Helpful:** Can ease tight pelvic floor muscles often associated with IC. |

**Tips for Introducing New Supplements**

When your doctor does approve a supplement, a cautious approach will save you a lot of pain:

**Ask for pH-neutral options:** If you have a medical need for Vitamin C or a multivitamin, ask your doctor about low-acid, buffered, or pH-neutral formulations specifically designed for sensitive bladders.

**Introduce one at a time:** Never start three new supplements in the same week. If you have a flare, you won't know which pill caused it. Start one, wait two weeks, and track your symptoms.

**Use a symptom diary:** Write down exactly what you take, what you eat, and how your bladder feels 2 to 4 hours afterward.

**43.Can pelvic floor physical therapy help interstitial cystitis?**

Pelvic floor physical therapy is widely considered one of the most effective and scientifically proven treatments for interstitial cystitis (IC), also known as bladder pain syndrome. In fact, the American Urological Association (AUA) gives it an "Evidence Grade A" and recommends it as a first-line medical treatment.

To understand why it helps, it helps to look at the relationship between the bladder and the muscles that support it.

The pelvic floor muscles support the bladder. Cleveland Clinic

**The Bladder-Muscle Connection**

Research shows that up to 85% of people diagnosed with IC also have pelvic floor dysfunction. The pelvic floor is a "hammock" of muscles, ligaments, and tissues that stretch across the bottom of the pelvis, supporting your bladder and other pelvic organs.

In people with IC, these muscles are often **hypertonic** (chronically tight or in spasm). This constant tension creates a painful feedback loop:

**Mimics bladder pain:** Trigger points (knots) in the pelvic floor radiate pain that feels exactly like it is coming from the bladder or urethra.

**Causes urgency and frequency:** To urinate comfortably, your pelvic floor must relax. When these muscles are locked in a tight state, you may feel a constant urge to go or struggle to fully empty your bladder.

**Creates inflammation:** Chronic muscle tension restricts blood flow and traps inflammation in the surrounding tissues, amplifying the pain.

**How Physical Therapy Helps**

The goal of physical therapy for IC is the exact opposite of what many people assume. It is strictly focused on **relaxing and lengthening** the muscles, not tightening them. A specialized pelvic floor physical therapist will typically use:

**Manual therapy:** Hands-on techniques (both external and internal) to release trigger points in the pelvic floor, abdomen, low back, and hips.

**Myofascial release:** Gently stretching the connective tissue to improve blood flow and reduce nerve irritation.

**Breathing and down-training:** Teaching you diaphragmatic breathing (deep belly breathing) to actively drop and relax the pelvic floor.

**Home stretching routines:** Providing specific stretches to keep the muscles relaxed between sessions.

Many patients report a significant reduction in pain, urinary frequency, and urgency within the first 4 to 8 weeks of consistent therapy.

**The Golden Rule: No Kegels**

If you have IC, **you should completely avoid Kegel exercises.**

Kegels are designed to strengthen weak pelvic floor muscles by squeezing them. Because IC is driven by muscles that are already overworked and too tight, doing Kegels is like doing bicep curls with a severely cramped arm — it will only irritate the nerves further and trigger a painful symptom flare.

**44.Are pelvic floor strengthening exercises always good for interstitial cystitis?**

No, they are actually often **counterproductive** and can make symptoms significantly worse.

While it is a common misconception that all pelvic health issues require strengthening exercises (like Kegels), Interstitial Cystitis (IC)—also known as Bladder Pain Syndrome (BPS)—is usually the exception to the rule.

**The Problem with Tight Muscles (Hypertonicity)**

In the vast majority of people with Interstitial Cystitis, the pelvic floor muscles are not weak. Instead, they are **hypertonic**, meaning they are already overly tight, tense, and stuck in a state of chronic contraction or spasm.

Think of your pelvic floor as a hammock of muscles supporting your bladder. When the bladder is inflamed from IC, these muscles instinctively clench and tighten to guard the area against pain. Doing strengthening exercises on top of this tension is like trying to lift weights with a muscle that is already experiencing a severe cramp.

Attempting to strengthen a hypertonic pelvic floor typically triggers:

**Increased pain flares:** Clenching already tight muscles irritates the complex network of pelvic nerves, ramping up pelvic and suprapubic pain.

**Worse urgency and frequency:** Overly tight muscles physically press against the bladder, tricking your brain into thinking your bladder is full when it isn't.

**Toileting difficulties:** The pelvic floor muscles must completely relax to allow urine to flow. If they are locked in a contracted state, it can cause hesitancy or a weak stream.

**The Golden Rule for IC:** Avoid voluntary squeezing, holding, or Kegel exercises unless a specialized pelvic floor physical therapist has explicitly evaluated your muscles and told you to do them.

**What Actually Helps?**

Because the underlying issue is usually muscle tightness rather than weakness, the clinical gold standard for IC involves **pelvic floor relaxation and lengthening** (often called "down-training"). The objective is to teach these overworked muscles how to let go.

**Diaphragmatic Breathing:** Deep belly breathing naturally drops, expands, and stretches the pelvic floor muscles with every inhale.

**Pelvic Release Stretches:** Yoga-based stretches like **Child’s Pose** and **Happy Baby** gently open up the hips, groin, and lower back, encouraging the pelvic floor to soften.

**Pelvic Floor Physical Therapy (PFPT):** This is one of the most effective, evidence-based treatments for IC. A specialized physical therapist uses targeted hands-on techniques (both internal and external) to release painful muscular trigger points, reduce inflammation, and restore normal blood flow.

If you or someone you know is navigating IC symptoms, the safest approach is to get an evaluation from a certified pelvic floor physical therapist who can pinpoint your exact muscle tone and guide you through a relaxation routine rather than a strengthening one.

**45.Can bladder training help people with interstitial cystitis urinate less often?**

Yes, bladder training can be highly effective in helping people with interstitial cystitis (IC)—also known as bladder pain syndrome (BPS)—urinate less frequently.

While it might sound counterintuitive to try to control an organ that is actively causing discomfort, bladder training is a cornerstone of behavioral therapy for managing IC symptoms.

**Breaking the "Urgency Cycle"**

When someone has IC, the bladder wall becomes inflamed and highly sensitive. To avoid the pain or pressure that builds up as the bladder fills, people naturally get into the habit of running to the bathroom the moment they feel the slightest urge.

While this brings short-term relief, it creates a problematic feedback loop:

**The Brain gets rewired:** The nervous system becomes hypersensitive, triggering intense "must-go-now" signals at lower and lower fluid volumes.

**The Bladder physically adapts:** Because the bladder is rarely holding a normal volume of liquid, its functional capacity shrinks over time, and the tissue can become stiff.

Bladder training essentially interrupts this cycle, retraining the brain and the bladder to tolerate normal volumes of urine again.

**How It Works in Practice**

Bladder training doesn't mean "holding it" until it hurts. Instead, it relies on a gradual, systematic approach:

**Timed Voiding:** Instead of urinating in response to an urge, patients urinate strictly by the clock (e.g., every 30 minutes to start), whether they feel the need to go or not.

**Urge Suppression:** If an intense urge hits *before* the scheduled time, the goal is to use distraction or deep, diaphragmatic breathing to calm the nervous system and let the wave pass, rather than rushing to the bathroom.

**Gradual Extensions:** Once a specific time interval becomes comfortable and accident-free, the schedule is increased by 15 to 30 minutes. Over 6 to 12 weeks, the goal is to safely stretch the interval to a normal 3 to 4 hours.

**A Note on Pain vs. Urgency**

Bladder training is highly effective for managing *urgency and frequency*, but it is typically recommended for those with mild-to-moderate baseline pain. For IC patients, forcing a highly inflamed bladder to hold urine through severe pain can cause a symptom flare-up.

Because many people with IC also suffer from hypertonic (overly tight) pelvic floor muscles, bladder training is most successful when paired with **pelvic floor physical therapy**. A specialized physical therapist can teach an individual how to physically relax the surrounding muscles so the bladder can fill without triggering painful spasms.

**46.Can bladder medicine placed through a tube help interstitial cystitis symptoms?**

Yes, putting medication directly into the bladder through a tube (a catheter) is a common and often very effective treatment for interstitial cystitis (IC), also known as bladder pain syndrome.

In the medical world, this procedure is called a **bladder instillation** or a **bladder wash**.

**How It Works**

Instead of swallowing a pill and waiting for it to go through your entire digestive system and kidneys, a doctor or nurse uses a thin, flexible tube to place a liquid medication mixture directly into your bladder. You then hold the liquid in your bladder for a set amount of time—usually anywhere from 15 minutes to 2 hours—before urinating it out.

By bypassing the rest of your body, this method delivers a high concentration of the medicine exactly where it hurts, minimizing systemic side effects.

**Common Medications Used**

Doctors rarely use just one medicine; they often mix a "cocktail" tailored to your symptoms. The most common ingredients include:

**Heparin or Pentosan Polysulfate:** These medications help rebuild and repair the bladder's protective lining (the GAG layer), which is often leaky or damaged in people with IC.

**Lidocaine:** A local anesthetic that temporarily numbs the bladder wall to provide immediate relief from severe pain and urgency.

**Sodium Bicarbonate:** An antacid that neutralizes the acidity of the liquid, making the instillation much more comfortable and helping the lidocaine absorb better.

**Dimethyl Sulfoxide (DMSO):** An older FDA-approved treatment that reduces inflammation and helps relax the bladder muscle. Because it can sometimes cause a temporary flare-up or a garlicky taste/odor, it is used less frequently today than heparin/lidocaine mixtures.

**What to Expect**

Treatments are typically given weekly or bi-weekly for a baseline period of 4 to 8 weeks. If the treatments help, many patients are taught how to perform the instillations safely at home, allowing them to manage sudden flare-ups independently.

**A Quick Word on Discomfort:** Inserting the catheter can cause some temporary irritation or a brief sting, especially if your bladder is already highly inflamed. However, once the numbing medication takes effect, most patients find significant relief.

**47.Is major surgery usually needed for interstitial cystitis?**

No, major surgery is rarely needed for interstitial cystitis (IC) — it is strictly considered a treatment of last resort.

For the vast majority of people, IC (also known as bladder pain syndrome) is managed with far less invasive therapies. Doctors generally view surgery as an option only when all other treatments have failed and the symptoms are severely impacting a person's quality of life.

**The Standard Approach to Treatment**

Instead of surgery, a urologist will typically start with a combination of conservative therapies to find what works best for your specific symptoms:

**Lifestyle and Diet Changes:** Identifying and avoiding trigger foods (like caffeine, citrus, and spicy foods) and using stress-reduction techniques.

**Physical Therapy:** Specialized pelvic floor physical therapy to relieve the muscle tension and tenderness that often accompany IC.

**Oral Medications:** Over-the-counter pain relievers, antihistamines to reduce urgency, or prescription medications specifically designed to help restore the bladder's protective inner lining.

**Bladder Instillations:** A procedure where a soothing liquid medication is temporarily washed into the bladder through a catheter to reduce inflammation.

**When is Surgery Considered?**

A specialist will typically only recommend exploring surgical options if a patient has exhausted the treatments above over a long period, or if they have specific physical complications, such as:

A severely shrunken, stiff bladder that can barely hold urine.

The presence of **Hunner's lesions** (distinct inflammatory patches or ulcers on the bladder wall, which occur in only about 5–10% of IC patients).

Even when major surgery is on the table, it is not a guaranteed cure. Because IC can involve the broader pelvic floor and nervous system, some patients continue to experience pelvic pain even after the bladder is completely removed.

**Surgical Options for IC**

When surgical intervention is necessary, doctors usually start with the least invasive options possible before considering major structural changes.

| **Procedure** | **Invasiveness** | **What it involves** |
| --- | --- | --- |
| **Hydrodistention** | Minor | Stretching the bladder with fluid under anesthesia to temporarily relieve symptoms. |
| **Fulguration** | Minor | Using heat or lasers to burn away Hunner's lesions on the bladder wall. |
| **Neuromodulation** | Moderate | Implanting a small device that sends mild electrical pulses to the nerves controlling the bladder. |
| **Bladder Augmentation** | Major | Enlarging the bladder's capacity using a segment of the patient's intestine. |
| **Cystectomy** | Major | Removing the bladder entirely and rerouting the urinary tract to an external bag or internal pouch. |

If you are exploring treatments for IC, it is highly recommended to work with a urologist who specializes in chronic pelvic pain or bladder pain syndrome. They can help you navigate the many non-surgical options available before ever needing to consider an operating room.

**48.Can interstitial cystitis affect sexual life?**

Yes, interstitial cystitis (IC) — also known as bladder pain syndrome — can have a profound impact on a person's sexual life. In fact, patient surveys indicate that up to 90% of people with IC report that the condition has disrupted their intimacy.

Because the topic of sexual pain is often under-discussed in doctor's offices, many people feel isolated by this symptom. However, it is a very common, very real, and highly treatable part of the condition.

Here is a breakdown of why IC affects intimacy and, more importantly, how it can be managed.

**Why IC Causes Sexual Pain**

The disruption to sexual function usually stems from a mix of anatomy, muscle response, and inflammation:

**Anatomical proximity:** In women, the bladder and urethra sit directly against the front wall of the vagina. Deep penetration or thrusting can physically press against the already inflamed bladder, causing sharp or aching pain (dyspareunia).

**Pelvic floor dysfunction:** Chronic bladder pain often causes the muscles of the pelvic floor to subconsciously tense up or spasm in a protective "guarding" reflex. This hyper-tension makes penetration painful and can cause lingering aching after sex.

**Nerve crossover:** The nerves that supply the bladder also supply the genitals. When the bladder is flared, the entire pelvic region can become hypersensitive.

**Impact on men:** While IC is more common in women, men with the condition often experience pain in the perineum or genitals, pain during or after ejaculation, and sometimes erectile dysfunction related to pain anxiety or pelvic muscle tension.

**The Psychological Toll**

The physical pain often triggers a frustrating psychological cycle. When sex hurts, it is natural to develop an anxiety or fear of intimacy. This anxiety causes further involuntary muscle tension, which then leads to even more pain during the next encounter. Over time, this can lead to decreased libido, feelings of guilt or inadequacy, and strain on a relationship if the partners aren't communicating openly about it.

**Strategies for Comfortable Intimacy**

While IC can make sex challenging, many people are able to maintain fulfilling sex lives by adjusting their approach and managing symptoms proactively.

**Empty your bladder:** Make it a habit to urinate immediately before and immediately after sexual activity. This reduces physical pressure on the bladder during sex and helps flush out bacteria to prevent secondary urinary tract infections.

**Pre-treat the pain:** If your doctor has prescribed muscle relaxants, bladder antispasmodics, or specific pain medications, taking them 15 to 20 minutes before intimacy can help prevent a flare-up.

**Use the right lubricant:** Friction exacerbates pelvic tension. Use a generous amount of hypoallergenic, water-based lubricant without any warming, cooling, or flavored additives, as these can severely irritate sensitive tissues.

**Modify positions:** Avoid positions that allow for deep penetration or put direct pressure on the front wall of the vagina. Side-lying ("spooning") or positions where you have control over the depth and angle of penetration are usually much more comfortable.

**Explore "outercourse":** Intimacy does not have to mean penetrative intercourse. When your bladder is flaring, focus on manual stimulation, oral sex, massage, or mutual masturbation to maintain a physical connection without triggering bladder pain.

**Use temperature therapy:** Taking a warm bath before sex can help relax tight pelvic muscles. After sex, applying an ice pack (wrapped in a cloth) to the perineum or suprapubic area can calm inflammation and soothe lingering burning sensations.

**Professional Support**

If you haven't already, strongly consider asking your urologist or gynecologist for a referral to a **pelvic floor physical therapist**. Unlike traditional Kegel exercises (which tighten the muscles and can actually make IC *worse*), a specialized therapist will teach you how to consciously relax and release the tight pelvic muscles that contribute to sexual pain.

Couples counseling or sex therapy can also be incredibly helpful for navigating the communication challenges and emotional weight that chronic pain places on a relationship.

**49.What can someone do if interstitial cystitis causes pain during sex?**

Managing intimacy when dealing with Interstitial Cystitis (IC), or Bladder Pain Syndrome, can be incredibly challenging. It is very common for individuals with IC to experience dyspareunia (painful sex), often because the condition is closely linked to pelvic floor dysfunction, nerve sensitivity, and inflammation.

*Disclaimer: I am an AI, not a doctor. The information below is for educational purposes and should not replace professional medical advice. Always consult with a urologist, urogynecologist, or pelvic floor specialist regarding your specific symptoms.*

**1. Professional Guidance**

If you haven't already, the most effective step is to build a care team that understands the intersection of IC and sexual pain.

**Pelvic Floor Physical Therapy (PFPT):** This is often the gold standard treatment for IC-related pain. Many people with IC have "hypertonic" (too tight) pelvic floor muscles. A specialized PT can help relax these muscles, which can significantly decrease pain during and after intercourse.

**Consult a Specialist:** Seek out a urologist or urogynecologist who specifically lists IC or pelvic pain as an area of expertise. They may be able to offer localized treatments, such as nerve blocks, bladder instillations, or medication adjustments that minimize systemic side effects.

**2. Mechanical and Practical Adjustments**

Simple physical changes can often reduce the amount of direct pressure placed on the bladder during intimacy.

**Choose the Right Lubrication:** Many people with IC are sensitive to chemicals, fragrances, glycerin, and parabens, which are common in drugstore lubricants. Look for **medical-grade silicone lubricants** or **natural, pH-balanced, water-based lubricants** that are free from irritants.

**Experiment with Positioning:** Focus on positions that minimize direct pressure on the suprapubic area (the lower abdomen where the bladder is located). Side-lying positions or positions where you have more control over the depth and angle of penetration are often more comfortable.

**Control the Pace:** Engaging in plenty of foreplay helps the pelvic muscles relax, which may reduce the initial "guarding" or tightening response that often precedes pain.

**3. Routine and After-Care**

Adjusting your routine around intimate activity can help manage the post-coital "flare" that many IC patients experience.

**Pre-Activity:** Some find it helpful to empty the bladder just before intimacy to reduce fullness and pressure.

**Post-Activity:** Gently cleaning the area with warm water (or a bidet) can help remove any external irritants.

**Temperature Therapy:** Applying a heating pad or a warm water bottle to the lower abdomen after intimacy can help relax the pelvic muscles and soothe irritation. Conversely, some people find an ice pack more soothing if they feel a burning sensation.

**4. Communication and Intimacy**

IC is a chronic condition that can take an emotional toll. Open communication is vital.

**Redefine Intimacy:** Focus on physical closeness that does not necessarily involve intercourse. This takes the pressure off the need for a specific sexual act to be the only goal, which can reduce anxiety—and anxiety often causes the pelvic floor to tighten, exacerbating IC pain.

**Set Clear Boundaries:** It is perfectly acceptable to stop at any time if pain begins. Establishing a "stop" or "pause" signal with your partner can help you feel safer and more in control.

**50.Can interstitial cystitis symptoms get worse after sex?**

Yes, absolutely. In fact, it is one of the most common triggers for an interstitial cystitis (IC) flare. Clinical studies show that roughly 75% to 82% of sexually active people with IC experience a temporary worsening of their symptoms after intimacy.

It can be incredibly frustrating to navigate, but knowing exactly *why* it happens can help take away some of the mystery.

**Why Sex Triggers IC Flares**

**Direct Mechanical Pressure:** Because the bladder and urethra sit directly against the vaginal wall, the physical friction and movement of penetration can mechanically irritate an already inflamed bladder lining.

**Pelvic Floor Muscle Tightness:** IC rarely travels alone; it is heavily linked to pelvic floor dysfunction. The muscles that naturally contract during arousal and orgasm can go into spasm or tighten around painful "trigger points," leaving you with a deep ache that can linger for hours or days.

**The Tension Loop:** If you are expecting sex to hurt, your body will unconsciously brace for it by tightening your pelvic muscles. This creates a feedback loop where the fear of pain actually causes more physical tension and a harsher flare afterward.

**How to Calm or Prevent a Post-Sex Flare**

While it is always a good idea to work with a healthcare provider or a pelvic floor physical therapist for long-term relief, there are several immediate, at-home strategies you can use to protect your bladder:

**Hydrate Right After:** Drink a large glass of water immediately after sex. This dilutes your urine so that when you go to empty your bladder, the fluid is less acidic and significantly less irritating to your bladder lining.

**Temperature Therapy:** Apply a cold pack or a heating pad (wrapped in a towel) to your perineum—the area between the vagina/base of the penis and the anus—for 15 to 20 minutes to calm down aggravated nerves.

**Take a Warm Sitz Bath:** Soaking in a warm, shallow bath right afterward is an excellent way to force hyper-tight pelvic floor muscles to drop their tension and reset.

**Reset with Stretching:** Doing a gentle pelvic floor stretch like the "Happy Baby" pose or taking slow, deep diaphragmatic (belly) breaths immediately after sex sends a physical safety signal to your nervous system, letting those muscles relax.

**Use Plenty of Lubricant:** Friction is the enemy of an inflamed bladder. Opt for a generous amount of a high-quality, water- or silicone-based lubricant designed for sensitive skin.

If penetration is consistently causing severe flares, many couples choose to pivot to "outercourse" or non-penetrative intimacy to stay close without triggering the physical mechanics that aggravate the bladder.

**51.What should someone do if interstitial cystitis symptoms worsen during pregnancy?**

It can be incredibly frustrating to deal with an interstitial cystitis (IC) flare while pregnant. While many people actually see their IC symptoms improve during pregnancy (often peaking in the second trimester due to hormonal shifts), it is also common for symptoms to worsen — especially in the first trimester or in the third trimester when the growing baby puts direct physical pressure on the bladder.

If IC symptoms are flaring up during pregnancy, here is the most effective approach to managing the pain safely:

**1. Rule Out a UTI First**

Pregnancy naturally increases the risk for urinary tract infections (UTIs) due to changes in the urinary system. Because a UTI feels exactly like an IC flare, the first step should always be to contact an OB/GYN or midwife for a urine culture. If there is a bacterial infection, it needs prompt treatment with pregnancy-safe antibiotics.

**2. Coordinate Medical Care**

Managing IC during pregnancy requires a urologist and OB/GYN to be on the same page, particularly regarding medications.

**Review oral medications:** Many common IC prescriptions (like Elmiron or amitriptyline) have complex FDA pregnancy safety ratings and may need to be paused or adjusted. Never start or stop a medication without consulting both doctors.

**Explore localized treatments:** If oral medications are restricted, doctors might suggest bladder instillations (such as hyaluronic acid). Because these are administered directly into the bladder via a catheter, they have very low systemic absorption and are often considered safer for the fetus.

**3. Lean Into the "IC Diet"**

Pregnancy cravings can sometimes derail dietary management, but sticking to bladder-friendly foods is one of the safest ways to control symptoms without medication.

**Avoid the triggers:** Steer clear of the "4 Cs" (**C**affeine, **C**arbonation, **C**itrus, and **C**hocolate), as well as tomatoes, spicy foods, and artificial sweeteners.

**Hydrate:** Drink plenty of water to dilute urine. Highly concentrated urine is acidic and will rapidly irritate the bladder wall.

**4. Focus on Pelvic Floor Relaxation**

Pregnancy puts a heavy physical load on the pelvic floor. For many with IC, pain is driven by tight, spasming pelvic floor muscles rather than just the bladder itself.

**Skip the Kegels:** Unless specifically directed by a physical therapist, avoid Kegels. Tightening an already strained pelvic floor will likely make IC pain much worse.

**Practice relaxation:** Focus on deep diaphragmatic breathing (belly breathing). Gentle, pregnancy-safe stretches like Cat-Cow or a modified Happy Baby pose can help release pelvic tension.

**See a specialist:** If possible, ask for a referral to a pelvic floor physical therapist who has experience with both pregnancy and IC. They can safely perform internal or external massage to release trigger points.

**5. Use Safe Comfort Measures**

**Temperature therapy:** A heating pad can help soothe muscle spasms, but **always ask an OB/GYN first**. They typically recommend using it only on the lowest setting for short periods, applied to the perineum or lower back rather than directly over the uterus. Alternatively, a cold compress between the legs can help numb nerve pain.

**Clothing:** Ditch any restrictive clothing or tight waistbands. Switch to loose-fitting maternity wear that puts zero pressure on the lower abdomen.

**Stress reduction:** Physical and emotional stress directly increase pain sensitivity and trigger inflammation. Prioritize rest, try prenatal meditation, and allow the body some grace as it handles both a chronic condition and a pregnancy.

**52.What can someone do if interstitial cystitis causes anxiety?**

It is incredibly common for Interstitial Cystitis (IC) and anxiety to fuel each other. The constant unpredictability of bladder pain and urgency naturally triggers anxiety, while the stress from anxiety causes your body to subconsciously tense up, tightening the pelvic floor muscles and making bladder symptoms worse.

Breaking this cycle requires addressing both the physical tension and the nervous system's stress response. Here are some of the most effective strategies to manage anxiety caused by IC:

**1. Address the Physical Link: Pelvic Floor "Down-Training"**

When someone experiences chronic bladder pain or anxiety, their pelvic floor muscles go into a protective, clenched state called guarding.

**Pelvic Floor Physical Therapy (PFPT):** Working with a specialized pelvic floor physical therapist is one of the most effective treatments for IC. They teach you how to "down-train" or lengthen and relax these muscles, rather than strengthen them (traditional Kegels can actually make IC worse).

**Reverse Kegels and Diaphragmatic Breathing:** Practice belly breathing. When you breathe deeply into your abdomen, your diaphragm drops, which physically pushes down and relaxes the pelvic floor. Think of it as gently releasing the muscles you use to start the flow of urine.

**2. De-escalate the Nervous System**

The brain and the bladder share a massive network of nerve pathways. Calming the brain directly calms the bladder nerves.

**Somatic Tracking:** When a flare starts, the brain sends high-alert danger signals (anxiety). Somatic tracking involves focusing on the physical sensation in the bladder with a sense of neutral curiosity rather than fear, telling yourself, *"This is a temporary nerve irritation, my tissue is safe."*

**Vagus Nerve Stimulation:** Simple physical resets like splashing cold water on your face, humming, or prolonged exhales (breathing out longer than you breathe in) activate the parasympathetic nervous system, which stops the adrenaline rush of an anxiety spike.

**3. Leverage Crossover Medical Treatments**

Some therapies and medications are uniquely suited to target both chronic pain pathways and anxiety simultaneously.

**Chronic Pain Therapy (CBT/PRT):** Cognitive Behavioral Therapy (CBT) and Pain Reprocessing Therapy (PRT) are highly effective at retraining how the brain interprets pain signals, lowering the baseline anxiety that triggers flares.

**Medication Assessment:** Talk to a physician about low-dose tricyclic antidepressants (like amitriptyline). At very low doses—much lower than what is used for depression—these medications help calm overactive bladder nerves, improve sleep, and reduce generalized anxiety.

**A Quick Note on Food Anxiety:** It is easy to develop intense anxiety around eating when trying to manage IC. Try to shift from a mindset of strict elimination to finding your safe "comfort foods" so that eating remains an active tool for comfort rather than a source of stress.

**53.Can counseling help people cope with long-term interstitial cystitis pain?**

Yes, counseling is highly effective for managing long-term interstitial cystitis (IC) or bladder pain syndrome (BPS). In fact, the American Urological Association (AUA) explicitly recommends stress management and coping strategies as part of the standard, multimodal treatment plan for the condition.

Dealing with the constant, unpredictable nature of IC pain is exhausting. It is crucial to know that being referred to a therapist does **not** mean your doctor thinks the pain is "all in your head." IC causes very real, visible physiological changes and inflammation in the bladder lining.

However, because all pain is ultimately processed by the brain, psychological tools are some of the most powerful levers you have to turn down the volume of that physical pain.

Inflamed bladder surface in Interstitial Cystitis. Glycologix

**The Pain Cycle and the Nervous System**

When you experience severe, chronic pain in your pelvis, your central nervous system becomes hyper-vigilant. It gets stuck in a "fight or flight" state. Stress, anxiety, and the anticipation of the next flare-up directly trigger the release of chemicals that cause further bladder inflammation and involuntary tightening of the pelvic floor muscles.

This creates a vicious feedback loop: **Pain Fear & Anxiety Muscle Tension & Inflammation More Pain**.

Counseling helps by actively breaking this cycle. By managing the nervous system's hyperarousal, you can directly reduce the severity and frequency of physical IC flare-ups.

**The Most Effective Therapies for IC**

Research strongly supports specific mind-body interventions for chronic pelvic pain conditions:

**Cognitive Behavioral Therapy (CBT):** This is the most extensively studied psychological treatment for IC. CBT helps you identify and shift the thought patterns, avoidance behaviors, and fears that keep your nervous system on high alert. Recent clinical trials have shown that patients who combine CBT with standard medical bladder treatments experience significantly less anxiety and better overall symptom relief than those using medical treatments alone.

**Acceptance and Commitment Therapy (ACT):** Rather than fighting a losing battle to eliminate the pain entirely, ACT focuses on "psychological flexibility." It teaches you how to notice pain without letting it control your decisions, helping you re-engage with the activities you value and live a full life even when symptoms are present.

**Mindfulness-Based Stress Reduction (MBSR):** Mindfulness teaches body awareness and a non-reactive stance toward physical sensations. By learning to observe the pain rather than panicking about it, you can quiet the nervous system. Studies show it is highly effective in managing chronic pelvic pain over time.

**Pain Reprocessing Therapy (PRT):** This newer approach uses a technique called "somatic tracking" to help retrain the brain to view lingering pain signals as safe rather than dangerous, which can effectively dial down the intensity of chronic nerve pain.

Working with a therapist who specializes in chronic pain can give you a vital toolkit to reclaim your quality of life, working hand-in-hand with your physical therapies and urological treatments.

**54.How can interstitial cystitis affect work, sleep, and daily life?**

Interstitial cystitis (IC), also frequently referred to as bladder pain syndrome (BPS), is a chronic condition that goes far beyond simple physical discomfort. Because it is unpredictable and painful, it exerts a massive cognitive, emotional, and physical toll on a person's entire day.

**The Impact on Sleep**

The disruption to sleep is often one of the most draining aspects of IC, establishing a frustrating cycle of pain and exhaustion.

**Nocturia:** This is the medical term for waking up to urinate at night. People with IC often have to get out of bed multiple times—sometimes anywhere from 4 to more than 10 times a night—driven by intense, painful urgency.

**Fragmented Sleep Cycles:** Because these awakenings are so frequent, the body rarely reaches the deep, restorative phases of sleep. This leads to chronic daytime fatigue and "brain fog" (sluggish memory and concentration).

**The Sleep-Pain Loop:** Sleep and pain have a complex, bidirectional relationship. Sleep deprivation lowers your pain threshold the following day. This means bladder symptoms can feel significantly more intense after a night of poor sleep, which in turn makes it harder to fall asleep the next night.

**The Impact on Work**

Navigating a career while managing an invisible, volatile condition introduces significant friction into professional life.

**Interrupted Workflows:** The sudden, frequent need to use the restroom can disrupt meetings, presentations, or deep-focus tasks. Rigid schedules with limited breaks can cause immense stress.

**Physical Constraints:** Prolonged sitting at a desk puts direct pressure on the pelvic floor muscles, which can easily trigger or worsen a flare.

**Presenteeism and Absenteeism:** "Presenteeism" means showing up to work but underperforming because you feel unwell. Chronic fatigue and baseline pain drain cognitive energy, making decision-making harder. On particularly bad flare days, severe pain forces unexpected absences, adding to professional anxiety regarding job security.

**The Impact on Daily Life**

Beyond work and sleep, IC alters basic routines and requires a heavy mental load of constant planning.

**Constant Background Calculations:** A person living with IC is rarely on autopilot. Every meal or beverage requires a quick mental screening for dietary triggers like caffeine, alcohol, citrus, tomatoes, or artificial sweeteners.

**"Bathroom Mapping":** Simple outings, errands, or travel require meticulous logistics. Individuals often find themselves mapping out exactly where public restrooms are located before even leaving the house.

**Strained Relationships and Intimacy:** Chronic pelvic and bladder pain can make sexual intimacy uncomfortable or deeply painful. This physical barrier frequently strains romantic partnerships and adds emotional stress.

**Social Isolation:** The sheer anxiety of triggering a flare, combined with low physical energy, causes many people to decline invitations or isolate themselves to avoid discomfort and explanations.

**The Invisible Illness Toll:** Because IC is an invisible condition—meaning someone often looks perfectly healthy on the outside while experiencing severe internal pain—individuals frequently report feeling misunderstood or dismissed by friends, family, and coworkers. This disconnect, paired with chronic exhaustion, contributes to significantly higher rates of anxiety and depression among IC patients.

**55.How should interstitial cystitis be managed over the long term?**

Managing interstitial cystitis (IC)—also called Bladder Pain Syndrome (BPS)—over the long term is all about building a highly personalized "toolkit." Because there is no single cure, clinical guidelines from organizations like the American Urological Association (AUA) emphasize a multi-layered approach that minimizes side effects while maximizing quality of life.

The underlying goal is to calm chronic inflammation, repair the bladder’s protective lining, and desensitize hyperactive nerves.

In the diagram above, you can see how IC transforms a smooth, healthy bladder lining into an irritated, inflamed surface. This damage allows acidic components in urine to penetrate the bladder wall, triggering pain and urgency.

**1. The Behavioral Foundation (First-Line)**

Lifestyle habits are the most powerful tools for preventing flares over the long term because they carry zero side effects.

**Dietary Modification:** Certain foods act like "pouring salt on a wound." The most common culprits are the **"Four Cs"**: **C**affeine, **C**itrus, **C**arbonation, and **C**hili (spicy foods), alongside alcohol and artificial sweeteners. An elimination diet helps identify personal triggers.

**Fluid Management:** It is a common mistake to drink less water to avoid running to the bathroom. However, concentrated urine is highly acidic and heavily irritates the bladder wall. Drinking steady, moderate amounts of water throughout the day keeps urine diluted.

**Stress Management:** Stress causes the body to release histamines and activates mast cells in the bladder wall, directly triggering physical flares. Long-term management often relies heavily on mindfulness, cognitive behavioral therapy (CBT), or gentle yoga.

**2. Pelvic Floor Physical Therapy**

A massive piece of the long-term puzzle is addressing the muscles surrounding the bladder.

**The Golden Rule of IC Physical Therapy:** Proactively **avoid Kegels**.

While people often associate bladder issues with weak muscles, IC patients almost always have a **hypertonic pelvic floor**—meaning the muscles are locked in a tight, painful spasm. Kegels make this worse. Specialized pelvic floor physical therapy focuses on myofascial release, trigger point therapy, and lengthening exercises to drop the baseline tension around the bladder.

**3. Oral Medications & Supplements**

When lifestyle shifts are not enough to keep symptoms quiet, daily oral options can help stabilize the bladder's environment.

**Antihistamines (e.g., Hydroxyzine):**These help block the mast cell activation and histamine release that drive bladder allergic-like inflammation.

**Tricyclic Antidepressants (e.g., Amitriptyline):** Used at much lower doses than for depression, these medications block pain signals from the bladder nerves and help relax the bladder muscle.

**GAG Layer Replacements (e.g., Elmiron or OTC Elmiron alternatives like Aloe Vera/Glucosamine):** These aim to chemically rebuild the protective glycosaminoglycan (GAG) layer of the bladder. *Note: Elmiron requires careful long-term monitoring by an ophthalmologist due to potential risks to the retina.*

**4. Advanced & Interventional Tiers**

If symptoms remain severe during long-term management, urologists escalate to targeted procedures:

**Bladder Instillations:** Often called a "bladder cocktail," a doctor uses a thin catheter to place medications like heparin, lidocaine, or sodium bicarbonate directly into the bladder to coat and numb the lining.

**Neuromodulation:** A small implanted device delivers mild electrical pulses to the nerves that control the bladder, disrupting the chronic pain-and-urgency loop.

**Botox Injections:** Injecting Botox directly into the bladder muscle can paralyze overactive nerves and expand bladder capacity, though it carries a small risk of temporary urinary retention.

Ultimately, long-term success is rarely about finding one magic pill; it involves layering these strategies together so that if one defense line wavers, the others keep a flare from taking over.

**56.Should people with interstitial cystitis keep following their care plan after symptoms improve?**

Yes. In general, people with interstitial cystitis (IC) — also known as bladder pain syndrome (BPS) — should continue to follow their care plan, or a modified version of it, even after they feel better.

Because IC is a chronic condition, there is currently no permanent cure. When symptoms improve, it usually means the care plan is actively working to suppress inflammation and nerve sensitivity, not that the underlying condition has disappeared. Abandoning the plan entirely is one of the most common reasons for a sudden, painful flare-up.

However, your daily routine doesn't necessarily have to remain as intense as it was during a flare. Once your symptoms are under control, you transition from an "active treatment phase" to a **maintenance phase**.

**How a Maintenance Plan Works**

Here is a breakdown of how different parts of an IC care plan are typically handled once symptoms improve:

**1. Lifestyle and Diet (Usually Lifelong)**

The behavioral modifications that helped calm your bladder are generally the most important habits to stick with long-term.

**Dietary triggers:** Continuing to limit known bladder irritants (like caffeine, alcohol, artificial sweeteners, citrus, and spicy foods) is crucial. While you might eventually be able to test reintroducing certain foods in small amounts, a baseline IC-friendly diet is usually permanent.

**Stress management:** Physical and emotional stress are major triggers for IC flare-ups. Maintaining habits like gentle exercise, yoga, and prioritizing sleep remains vital.

**Hydration:** Drinking enough water to keep your urine pale yellow helps prevent highly acidic, concentrated urine from irritating the bladder lining.

**2. Pelvic Floor Physical Therapy (Ongoing Practice)**

Many people with IC have underlying pelvic floor dysfunction (tight or spasming muscles). If a physical therapist helped you achieve symptom relief, you should continue the stretching and relaxation techniques (like diaphragmatic breathing or reverse Kegels) at home. Keeping those muscles relaxed prevents the cycle of tension and pain from restarting.

**3. Medications and Procedures (Requires Medical Guidance)**

This is where your plan is most likely to change, but **never stop taking medications abruptly without consulting your urologist.**

**Oral Medications:** Drugs like amitriptyline, antihistamines, or pentosan polysulfate (Elmiron) often take months to reach full effect. If you stop taking them as soon as you feel better, the bladder inflammation or nerve overactivity will often return. Your doctor may eventually try to lower your dose to the minimum effective amount, but this must be done gradually.

**Bladder Instillations:** If you were receiving regular bladder washes, your doctor will likely begin spacing them out—moving from weekly to monthly, and eventually only doing them "as needed" if a flare-up occurs.

**What to Do Next**

If you have been feeling well for a while, schedule a check-in with your urologist or urogynecologist to discuss your progress. You can ask them:

"Now that my symptoms have improved, what does a long-term maintenance plan look like?"

"Are there any medications we can safely start to taper down?"

"What is our exact action plan if I experience a flare-up?"

**57.Should online claims about a complete cure for interstitial cystitis be trusted?**

No, you should treat any online claim of a "complete cure" for interstitial cystitis (IC) with extreme skepticism.

According to major medical institutions like the Mayo Clinic, Johns Hopkins Medicine, and the American Urological Association, **there is currently no known cure for interstitial cystitis**. The exact cause of the condition remains unknown, which makes a universal "cure" impossible at this stage.

Here is what is actually happening in the bladder, and why managing it requires a personalized approach rather than a quick fix:

**Why "Cure" Claims Are Red Flags**

Because IC is a painful, chronic condition that can be incredibly frustrating to live with, patients are often highly vulnerable to aggressive marketing and medical scams. Be highly suspicious of websites or practitioners that:

**Promise a 100% cure rate:** IC is a complex syndrome, often involving the nervous system, pelvic floor muscles, and the protective lining of the bladder. Because it manifests differently in everyone, no single treatment works universally.

**Sell proprietary "hidden infection" tests:** IC is a "diagnosis of exclusion," meaning standard urinary tract infections (UTIs) must be ruled out first. Some alternative clinics claim IC is *always* caused by deep-rooted bacterial infections that only their expensive, proprietary DNA sequencing can find. The American Urological Association does not currently support this as a standard cause or treatment pathway.

**Push exclusive supplements:** There is no "magic pill" or secret herbal blend that can rebuild the bladder lining overnight.

**How IC is Actually Managed**

While there isn't a cure, IC is highly treatable. The goal of legitimate medical care is to manage symptoms, reduce flare-ups, and improve your overall quality of life. Because symptoms vary, it usually involves a trial-and-error approach combining several methods:

**Dietary Modifications:** Eliminating common bladder irritants—often called the "four Cs" (caffeine, citrus, carbonation, and chocolate)—is usually the first step. Spicy foods, tomatoes, and alcohol are also common triggers.

**Pelvic Floor Physical Therapy:** Chronic bladder pain often causes people to unconsciously tense their pelvic muscles, leading to painful spasms. Specialized physical therapy can help release this tension and significantly reduce pain.

**Oral Medications:**

**Elmiron (pentosan polysulfate sodium):** The only FDA-approved oral medication specifically for IC, believed to help restore the inner protective surface of the bladder.

**Tricyclic antidepressants (like amitriptyline):** Used in low doses to help relax the bladder and block nerve pain signals.

**Antihistamines:** Can help reduce urinary urgency and frequency.

**Nerve Stimulation:** Techniques like transcutaneous electrical nerve stimulation (TENS) or sacral nerve implants can help block pain signals and reduce urgency.

**58.Is it safe to diagnose or treat interstitial cystitis only with advice from the internet?**

No, it is absolutely not safe to diagnose or treat interstitial cystitis (IC) based solely on advice from the internet.

Pelvic pain, constant pressure, and the sudden, overwhelming urge to urinate can be incredibly disruptive and frustrating to live with. It is completely understandable why anyone experiencing these symptoms would look for fast answers online. However, bypassing a medical professional for this specific condition carries significant health risks.

**Why Self-Diagnosis is Unsafe**

**The Symptoms are "Great Mimics":** The primary symptoms of IC—pelvic pain, urinary frequency, and a constant feeling of urgency—are identical to those of many other conditions. A website or an online quiz cannot differentiate IC from a persistent Urinary Tract Infection (UTI), bladder stones, pelvic floor dysfunction, endometriosis, or even bladder cancer.

**It's a Diagnosis of Exclusion:** There is no single blood test or scan that instantly confirms IC. Medical guidelines state that a clinical diagnosis of IC/Bladder Pain Syndrome requires a thorough medical history, physical examination, and negative urine cultures to completely rule out other treatable infections or disorders first.

**Internet Treatments Can Worsen Symptoms:** The bladder lining in someone with IC is highly sensitive. Many alternative remedies, unregulated supplements, or "bladder flushes" recommended online contain high levels of vitamin C, potassium, or acidic compounds that can severely flare or irritate the bladder wall further. Conversely, treating a hidden bacterial infection with internet remedies instead of antibiotics can cause it to travel up into the kidneys, leading to severe complications.

Bladder Lining Comparison. Mayo Clinic

Looking at the diagram helps clarify why an in-person medical evaluation is so vital. A **normal bladder surface** has a healthy, stable tissue lining, while a **bladder inflamed by interstitial cystitis** experiences internal irritation, swelling, and localized breakdown of its protective layer. An online checklist simply cannot inspect your anatomy, run lab cultures, or evaluate your physical symptoms to see what is happening underneath.

**What a Safe, Professional Evaluation Looks Like**

If you suspect your bladder symptoms are more than a standard infection, scheduling an appointment with a primary care doctor, urologist, or urogynecologist is the safest step. Here is what they will typically do to find the true cause:

| **Medical Evaluation Step** | **What It Checks For** | **Why It Matters** |
| --- | --- | --- |
| **Urinalysis & Urine Culture** | Bacteria, white blood cells, or blood in the urine. | To rule out active UTIs or lower-level bacterial infections before considering IC. |
| **Pelvic Floor Examination** | Muscle tenderness, spasms, or pelvic wall sensitivity. | To see if the pain is muscular (pelvic floor dysfunction) rather than bladder-specific. |
| **Symptom Index & Bladder Diary** | Patterns of fluid intake, voiding frequency, and pain scales. | Establishes a concrete baseline of your habits to guide safe, non-invasive therapies. |
| **Cystoscopy (If needed)** | Inspected look inside the bladder using a small camera. | Typically reserved for complex cases to rule out bladder stones or look for rare structural lesions. |

**Important Note:** A true clinical diagnosis of IC requires symptoms to be consistently present for at least six weeks alongside documented negative urine cultures.
