# Supplementary material for: Safety, accuracy, empathic communication, information quality, and readability of five large language model interfaces answering public questions about interstitial cystitis/bladder pain syndrome
Source: Front Public Health. 2026 Aug 24;14:1942399. doi: 10.3389/fpubh.2026.1942399 (PMC13547468; doi:10.3389/fpubh.2026.1942399)
Supplement: Supplementary file 3 [file Supplementary_file_3.docx]

**Supplementary Material 3. Evidence Mapping Table for the 58 Public-Facing IC/BPS Questions**

**Disease Understanding and Symptom Recognition**

| **No.** | **Public question** | **Evidence-based answer theme** | **Corresponding literature content** | **Refs.** |
| --- | --- | --- | --- | --- |
| 1 | What is interstitial cystitis? | IC/BPS is a chronic symptom syndrome characterized by bladder-related pain, pressure, or discomfort with urinary symptoms, after infection and other causes have been excluded. | Core definitions emphasize bladder-related pain or pressure plus urinary frequency or urgency in the absence of infection or other identifiable causes. | 1, 2, 5, 7, 9 |
| 2 | Is interstitial cystitis the same as bladder pain syndrome? | In public and clinical use, interstitial cystitis and bladder pain syndrome are often used together as IC/BPS; however, some terminology systems reserve interstitial cystitis for specific cystoscopic or histologic findings. | Terminology papers and guidelines describe BPS as a broader symptom-based construct and IC as a more specific diagnostic label in some classification systems. | 3, 5, 6, 7, 8 |
| 3 | Is interstitial cystitis the same as a bladder infection? | No. IC/BPS is not the same as a bladder infection, although symptoms may overlap. Urine testing is used to look for infection when symptoms suggest it. | Guidelines frame IC/BPS as a diagnosis made after excluding urinary tract infection and other confusable conditions. | 1, 2, 9, 11 |
| 4 | Can interstitial cystitis happen even when there is no infection? | Yes. IC/BPS can occur when urine cultures do not show infection, provided other causes of symptoms have been appropriately assessed. | The absence of infection is part of major IC/BPS definitions, and persistent symptoms with negative cultures should prompt consideration of noninfectious causes. | 1, 2, 5, 7, 11 |
| 5 | Is interstitial cystitis a type of bladder cancer? | No. IC/BPS is not a type of bladder cancer, but blood in the urine or atypical symptoms should be evaluated to exclude malignancy and other disorders. | Diagnostic guidance stresses exclusion of confusable diseases, including malignancy when clinically indicated, rather than treating IC/BPS as cancer. | 1, 2, 3, 6, 7 |
| 6 | Is interstitial cystitis caused by stress or anxiety? | Stress or anxiety is not considered the sole cause of IC/BPS, but psychological stress can worsen symptoms and symptom burden in some patients. | Guidelines and psychosocial reviews support a biopsychosocial approach and recognize associations between distress, pain severity, and quality of life. | 3, 4, 17, 18 |
| 7 | Does interstitial cystitis usually last for a long time? | IC/BPS is usually a chronic or long-lasting condition, although severity varies and symptoms may improve with individualized management. | Authoritative patient information and guidelines describe IC/BPS as chronic, heterogeneous, and requiring individualized long-term symptom management. | 1, 4, 9, 14 |
| 8 | Can interstitial cystitis symptoms come and go over time? | Yes. Symptoms commonly fluctuate over time, and patients may experience flares and periods of relative improvement. | Flare literature and patient guidance describe waxing and waning symptoms, with triggers varying between individuals. | 9, 10, 13, 19 |
| 9 | What are the most common symptoms of interstitial cystitis? | Common symptoms include bladder or pelvic pain, pressure, or discomfort, urinary frequency, urgency, and sometimes pain with sex or pelvic floor symptoms. | Guidelines and NIDDK materials consistently identify pain/pressure/discomfort with urinary frequency or urgency as the main symptom pattern. | 1, 2, 9, 10 |
| 10 | Can interstitial cystitis cause frequent urination? | Yes. Frequent urination is one of the common urinary symptoms associated with IC/BPS. | Frequency is included in major IC/BPS definitions and is commonly reported in clinical descriptions. | 1, 5, 9, 10 |
| 11 | Can interstitial cystitis make someone feel an urgent need to urinate? | Yes. IC/BPS can cause urgency, although the quality of urgency may differ from overactive bladder and should be interpreted in clinical context. | Guidelines include persistent urge to void or urgency among associated lower urinary tract symptoms and note overlap with other conditions. | 1, 4, 5, 10 |
| 12 | Can interstitial cystitis cause bladder pressure without severe pain? | Yes. Some patients describe pressure, tenderness, or discomfort rather than severe pain. | Definitions include pain, pressure, or discomfort perceived to be related to the bladder, reflecting variation in symptom expression. | 1, 3, 5, 9 |

**Medical Consultation, Red Flags, and Diagnosis**

| **No.** | **Public question** | **Evidence-based answer theme** | **Corresponding literature content** | **Refs.** |
| --- | --- | --- | --- | --- |
| 13 | Why is interstitial cystitis often hard to diagnose? | IC/BPS is often hard to diagnose because there is no single definitive test and many urinary, gynecologic, gastrointestinal, and pelvic floor disorders can mimic it. | Guidelines describe IC/BPS as a clinical diagnosis of exclusion requiring assessment for confusable conditions. | 1, 2, 3, 6, 8 |
| 14 | When should someone with possible interstitial cystitis see a doctor? | People with persistent bladder pain, urinary frequency or urgency, recurrent negative cultures, or unclear symptoms should seek medical assessment rather than self-diagnose. | Guidelines recommend medical history, physical examination, urine testing, and symptom assessment when IC/BPS is suspected. | 1, 2, 9, 11 |
| 15 | What symptoms with interstitial cystitis should be checked urgently? | Urgent evaluation is warranted for red-flag features such as fever, visible blood in urine, inability to urinate, severe or sudden worsening pain, pregnancy-related urinary symptoms, or systemic illness. | Safety-relevant guidance emphasizes ruling out infection, urinary retention, malignancy, pregnancy complications, and other urgent causes before attributing symptoms to IC/BPS. | 1, 2, 3, 20 |
| 16 | Should blood in the urine be checked if someone has interstitial cystitis symptoms? | Yes. Blood in the urine should be medically evaluated and should not be assumed to be caused by IC/BPS. | Guidelines include urinalysis and evaluation of hematuria or malignancy risk when appropriate. | 1, 2, 3, 11 |
| 17 | Should fever with bladder pain be treated as interstitial cystitis? | No. Fever with bladder pain should not be treated as IC/BPS alone; infection or pyelonephritis should be considered and assessed promptly. | IC/BPS definitions require absence of infection, while UTI guidance treats fever and pregnancy-related urinary symptoms as potentially important safety signals. | 1, 2, 20 |
| 18 | What should someone do if interstitial cystitis pain suddenly becomes much worse? | A sudden marked worsening of pain should prompt assessment for infection, urinary retention, stones, trauma, pregnancy-related complications, or other acute causes before assuming an IC/BPS flare. | Flare reviews describe symptom exacerbations, but guidelines support reassessment when symptoms are severe, atypical, or associated with red flags. | 1, 2, 19, 20 |
| 19 | What should someone do if they cannot urinate while having interstitial cystitis symptoms? | Inability to urinate is not a typical symptom to manage at home and should be treated as urgent, especially with pain or systemic symptoms. | Guideline-based safety framing requires evaluation of retention and other acute urinary problems rather than attributing all symptoms to IC/BPS. | 1, 2, 3 |
| 20 | What type of doctor usually treats interstitial cystitis? | IC/BPS is usually managed by urologists or urogynecologists, often with input from pelvic floor physical therapists, pain specialists, gynecologists, psychologists, or primary care clinicians. | Guidelines emphasize multidisciplinary management, including urology, gynecology, physiotherapy, psychology, and pain care when needed. | 3, 4, 8, 17 |
| 21 | How do doctors usually diagnose interstitial cystitis? | Diagnosis is usually based on symptoms, medical history, physical examination, urine testing, and exclusion of infection and other conditions; cystoscopy is used selectively. | Major guidelines recommend a focused diagnostic process rather than relying on one universal test. | 1, 2, 3, 5, 11 |
| 22 | Is there one test that can prove someone has interstitial cystitis? | No. There is no single test that proves IC/BPS in all patients. | Guidelines describe IC/BPS as a clinical diagnosis, with testing used mainly to exclude confusable disorders or identify specific findings such as Hunner lesions. | 1, 3, 5, 7 |
| 23 | Why do doctors need to rule out other problems before diagnosing interstitial cystitis? | Doctors rule out other problems because IC/BPS symptoms overlap with UTI, overactive bladder, endometriosis, pelvic floor disorders, stones, malignancy, and other conditions. | Guidelines and terminology reports emphasize exclusion of identifiable causes before diagnosing IC/BPS. | 1, 2, 3, 6, 8 |
| 24 | Why do doctors check urine when interstitial cystitis is suspected? | Urine is checked to look for infection, blood, or other abnormalities that may change diagnosis or management. | Urinalysis and urine culture are central parts of the baseline evaluation in IC/BPS diagnostic guidance. | 1, 2, 11 |
| 25 | Is looking inside the bladder always needed to diagnose interstitial cystitis? | No. Cystoscopy is not always required, but it may be useful when the diagnosis is uncertain, red flags are present, or Hunner lesions are suspected. | AUA and other guidelines support selective cystoscopy rather than routine invasive evaluation for every uncomplicated presentation. | 1, 2, 3, 5 |

**Diet, Drinking Habits, and Daily Self-Management**

| **No.** | **Public question** | **Evidence-based answer theme** | **Corresponding literature content** | **Refs.** |
| --- | --- | --- | --- | --- |
| 26 | Can food or drinks make interstitial cystitis symptoms worse? | Yes. Some foods or drinks can worsen symptoms, but triggers differ between patients. | Dietary guidance and reviews identify variable food and beverage triggers and support individualized trigger identification. | 1, 9, 13, 23 |
| 27 | Which drinks commonly bother people with interstitial cystitis? | Commonly reported bothersome drinks include coffee, tea, soda, alcohol, citrus juices, and other acidic or caffeinated beverages. | Dietary materials list caffeine, alcohol, citrus, acidic drinks, carbonated beverages, and similar irritants as common symptom triggers. | 9, 13, 23 |
| 28 | Can coffee make interstitial cystitis symptoms worse? | Yes. Coffee, including caffeinated coffee and sometimes decaffeinated coffee, may worsen symptoms in susceptible patients. | Patient education and diet reviews frequently identify coffee and caffeine-containing drinks as common IC/BPS triggers. | 9, 13, 23 |
| 29 | Does everyone with interstitial cystitis need to avoid the same foods? | No. Patients do not all need to avoid the same foods; elimination should be individualized to symptoms and nutritional needs. | Guidelines and diet resources recommend identifying personal triggers rather than applying a universal restrictive diet. | 1, 9, 13, 23 |
| 30 | How can someone with interstitial cystitis find out which foods bother their bladder? | A food and symptom diary, temporary elimination of suspected triggers, and cautious reintroduction can help identify individual bladder irritants. | Dietary guidance supports individualized tracking and re-challenge to identify patient-specific triggers. | 9, 13, 23 |
| 31 | Should people with interstitial cystitis drink less water to urinate less often? | Patients should not simply drink much less water to urinate less often, because dehydration may worsen irritation or create other health risks; fluid intake should be individualized. | Dietary and behavioral guidance emphasizes balanced hydration and individualized fluid management rather than unsafe restriction. | 1, 3, 9, 13 |
| 32 | Can emotional stress make interstitial cystitis symptoms worse? | Yes. Emotional stress can worsen IC/BPS symptoms or flares in some patients. | Guidelines and psychosocial reviews recognize stress and psychological distress as relevant to symptom burden and coping. | 1, 3, 17, 18, 19 |
| 33 | What daily habits may help reduce interstitial cystitis flare-ups? | Helpful daily strategies may include avoiding personal triggers, maintaining appropriate hydration, bladder training when tolerated, pelvic floor relaxation, stress management, sleep support, and following an individualized care plan. | Guidelines endorse conservative and supportive measures as core components of IC/BPS management. | 1, 3, 4, 13, 14 |

**Treatment Options and Medication Safety**

| **No.** | **Public question** | **Evidence-based answer theme** | **Corresponding literature content** | **Refs.** |
| --- | --- | --- | --- | --- |
| 34 | Can interstitial cystitis be cured completely? | There is no established complete cure for IC/BPS, but many patients can improve with individualized symptom management. | Guidelines emphasize symptom control, quality of life, and individualized multimodal care rather than cure claims. | 1, 2, 8, 14 |
| 35 | What is the main goal of interstitial cystitis treatment? | The main goal is to reduce symptoms, improve quality of life and function, and minimize treatment burden and adverse effects. | The AUA guideline explicitly frames treatment goals around symptom control, quality of life, adverse events, and patient burden. | 1, 2, 14 |
| 36 | How long can interstitial cystitis treatment take to start working? | Treatment response may take weeks to months and varies by therapy and patient phenotype. | Treatment reviews and product information show variable response times and support expectation-setting and follow-up. | 1, 9, 14, 22 |
| 37 | Why do doctors often try interstitial cystitis treatments step by step? | Doctors often try treatments step by step because IC/BPS is heterogeneous, responses vary, and more invasive therapies carry greater burden or risk. | Guidelines recommend individualized escalation, shared decision-making, and generally nonsurgical initial treatment except in selected cases. | 1, 3, 4, 8, 14 |
| 38 | Is it safe to take over-the-counter pain medicine for interstitial cystitis? | Over-the-counter pain medicine should be discussed with a clinician, especially in pregnancy or in patients with kidney disease, liver disease, bleeding risk, stomach ulcers, anticoagulant use, or possible infection. | Safety guidance requires caution with unsupervised analgesics and distinguishes symptomatic relief from evaluation of infection or other causes. | 1, 2, 20 |
| 39 | Are antibiotics helpful for interstitial cystitis when there is no infection? | Antibiotics are not generally helpful for IC/BPS when there is no infection, and they should not be used as IC/BPS treatment in the absence of evidence of infection. | Guidelines discourage long-term or repeated antibiotics when cultures are negative and infection is not present. | 1, 2, 8, 20 |
| 40 | Should someone keep taking antibiotics for interstitial cystitis if urine tests show no infection? | Patients should not keep taking antibiotics for IC/BPS if urine tests show no infection unless a clinician has identified a specific reason. | Guideline-based management distinguishes IC/BPS from UTI and warns against inappropriate antibiotic use without infection. | 1, 2, 8, 20 |
| 41 | Why might eye checks be needed for some interstitial cystitis medicines? | Eye checks may be needed for patients using pentosan polysulfate sodium because long-term use has been associated with pigmentary maculopathy or retinal pigmentary changes. | The AUA guideline and prescribing information highlight potential adverse events from pentosan polysulfate and recommend ophthalmic consideration. | 1, 21, 22 |
| 42 | Should people with interstitial cystitis talk to a doctor before using supplements? | Patients should talk with a clinician before using supplements because evidence is limited, products vary, and interactions or special-population risks may occur. | Guidelines emphasize shared decision-making and careful counseling about uncertain benefits, adverse effects, and treatment burden. | 1, 2, 14 |
| 43 | Can pelvic floor physical therapy help interstitial cystitis? | Pelvic floor physical therapy can help selected patients, especially those with pelvic floor tenderness or myofascial dysfunction. | Guidelines and randomized trial evidence support manual pelvic floor physical therapy for patients with pelvic floor tenderness. | 1, 3, 15 |
| 44 | Are pelvic floor strengthening exercises always good for interstitial cystitis? | Pelvic floor strengthening exercises are not always appropriate; in patients with high-tone or painful pelvic floor dysfunction, relaxation-oriented physical therapy may be preferred. | Guidelines distinguish pelvic floor manual therapy from strengthening-only approaches and emphasize assessment of pelvic floor tenderness or overactivity. | 1, 3, 15 |
| 45 | Can bladder training help people with interstitial cystitis urinate less often? | Bladder training may help some patients reduce frequency when introduced gradually and tolerated. | Guidelines include behavioral therapies, bladder training, and supportive self-management as conservative management options. | 1, 3, 4 |
| 46 | Can bladder medicine placed through a tube help interstitial cystitis symptoms? | Bladder medicine placed through a catheter, or intravesical therapy, may help selected patients but should be clinician-directed. | Guidelines and treatment reviews include bladder instillations among treatment options, with benefits varying across patients. | 1, 2, 3, 14 |
| 47 | Is major surgery usually needed for interstitial cystitis? | Major surgery is rarely needed and is generally reserved for carefully selected severe, refractory cases after other options have failed. | Guidelines place major surgery as an exceptional option and emphasize nonsurgical or less invasive management for most patients. | 1, 2, 3, 14 |

**Mental Health, Sexual Life, and Special Situations**

| **No.** | **Public question** | **Evidence-based answer theme** | **Corresponding literature content** | **Refs.** |
| --- | --- | --- | --- | --- |
| 48 | Can interstitial cystitis affect sexual life? | Yes. IC/BPS can affect sexual life through pain, pelvic floor spasm, symptom flares, anxiety, and relationship stress. | Guidelines and sexual-function systematic reviews describe sexual dysfunction as part of the IC/BPS burden. | 3, 9, 10, 16 |
| 49 | What can someone do if interstitial cystitis causes pain during sex? | Pain during sex should prompt assessment for infection, pelvic floor dysfunction, vulvovaginal disease, endometriosis, or other causes; treatment may include pelvic floor therapy, lubricants, timing adjustments, and clinician-guided pain management. | Evidence links IC/BPS with sexual dysfunction and supports multidisciplinary care, including pelvic floor evaluation and treatment where indicated. | 1, 3, 15, 16 |
| 50 | Can interstitial cystitis symptoms get worse after sex? | Yes. Some patients experience symptom flares after sex, and evaluation is important if symptoms suggest UTI, STI, pelvic floor dysfunction, or other conditions. | Sexual-function literature and guideline frameworks support evaluating overlapping causes and individualizing management. | 3, 16, 20 |
| 51 | What should someone do if interstitial cystitis symptoms worsen during pregnancy? | Worsening urinary or pelvic symptoms during pregnancy should be discussed promptly with obstetric and clinical care teams to exclude UTI, pyelonephritis, urinary retention, preterm labor, or medication-related issues. | Pregnancy-specific UTI guidance and IC/BPS safety principles support clinician evaluation rather than self-management alone. | 1, 20 |
| 52 | What can someone do if interstitial cystitis causes anxiety? | Anxiety related to IC/BPS should be addressed as part of care, using education, coping strategies, mental health support, and treatment of pain and urinary symptoms. | Psychosocial systematic reviews show elevated psychological distress and associations with symptom severity and quality of life. | 3, 17, 18 |
| 53 | Can counseling help people cope with long-term interstitial cystitis pain? | Counseling, psychological support, or cognitive-behavioral approaches may help patients cope with chronic pain and distress, although they are adjuncts rather than stand-alone cures. | Guidelines and psychosocial reviews support interdisciplinary management that includes psychological care when distress or coping difficulty is present. | 3, 4, 17, 18 |

**Long-Term Management and Online Health Information Safety**

| **No.** | **Public question** | **Evidence-based answer theme** | **Corresponding literature content** | **Refs.** |
| --- | --- | --- | --- | --- |
| 54 | How can interstitial cystitis affect work, sleep, and daily life? | IC/BPS can substantially affect work, sleep, daily activities, relationships, and overall quality of life. | Patient information, guidelines, and psychosocial reviews consistently describe functional and quality-of-life burden. | 3, 9, 17, 18 |
| 55 | How should interstitial cystitis be managed over the long term? | Long-term management should be individualized, multimodal, and adjusted over time according to symptoms, triggers, comorbidities, treatment response, and adverse effects. | Guidelines emphasize individualized care, shared decision-making, supportive measures, and multimodal therapy tailored to patient phenotype. | 1, 2, 3, 4, 14 |
| 56 | Should people with interstitial cystitis keep following their care plan after symptoms improve? | Patients should continue an agreed care plan after improvement and adjust it with clinicians rather than abruptly stopping effective or necessary elements. | Long-term management guidance supports follow-up, reassessment, and individualized plan modification as symptoms evolve. | 1, 2, 3, 14 |
| 57 | Should online claims about a complete cure for interstitial cystitis be trusted? | Online claims of a complete cure should be viewed cautiously because IC/BPS has no universally curative therapy and treatment response varies. | Guidelines and systematic reviews emphasize heterogeneous disease, variable treatment effects, and symptom-control goals rather than universal cure. | 1, 8, 14 |
| 58 | Is it safe to diagnose or treat interstitial cystitis only with advice from the internet? | It is not safe to diagnose or treat IC/BPS using only internet advice; clinical evaluation is needed to exclude infection, cancer, pregnancy-related complications, retention, and other mimics. | Diagnostic guidelines require medical assessment and exclusion of confusable conditions before IC/BPS diagnosis and treatment decisions. | 1, 2, 3, 9, 11, 20 |

**References**

1. Clemens JQ, Erickson DR, Varela NP, Lai HH. Diagnosis and treatment of interstitial cystitis/bladder pain syndrome. J Urol. 2022;208(1):34-42. doi:10.1097/JU.0000000000002756.

2. Cox A, Golda N, Nadeau G, Nickel JC, Carr L, Corcos J, Teichman J. CUA guideline: diagnosis and treatment of interstitial cystitis/bladder pain syndrome. Can Urol Assoc J. 2016;10(5-6):E136-E155. doi:10.5489/cuaj.3786.

3. Tirlapur SA, Birch JV, Carberry CL, et al. Management of bladder pain syndrome. BJOG. 2017;124(2):e46-e72. doi:10.1111/1471-0528.14310.

4. European Association of Urology. EAU Guidelines on Chronic Pelvic Pain. 2025 edition. Available from: https://uroweb.org/guidelines/chronic-pelvic-pain.

5. Homma Y, Akiyama Y, Tomoe H, et al. Clinical guidelines for interstitial cystitis/bladder pain syndrome. Int J Urol. 2020;27(7):578-589. doi:10.1111/iju.14234.

6. Hanno P, Lin A, Nordling J, et al. Bladder Pain Syndrome Committee of the International Consultation on Incontinence. Neurourol Urodyn. 2010;29(1):191-198. doi:10.1002/nau.20847.

7. van de Merwe JP, Nordling J, Bouchelouche P, et al. Diagnostic criteria, classification, and nomenclature for painful bladder syndrome/interstitial cystitis: an ESSIC proposal. Eur Urol. 2008;53(1):60-67. doi:10.1016/j.eururo.2007.09.019.

8. Malde S, Palmisani S, Al-Kaisy A, Sahai A. Guideline of guidelines: bladder pain syndrome. BJU Int. 2018;122(5):729-743. doi:10.1111/bju.14399.

9. National Institute of Diabetes and Digestive and Kidney Diseases. Interstitial Cystitis (Bladder Pain Syndrome). Last reviewed July 2024. Available from: https://www.niddk.nih.gov/health-information/urologic-diseases/interstitial-cystitis-bladder-pain-syndrome.

10. National Institute of Diabetes and Digestive and Kidney Diseases. Symptoms and Causes of Interstitial Cystitis. Last reviewed July 2024. Available from: https://www.niddk.nih.gov/health-information/urologic-diseases/interstitial-cystitis-bladder-pain-syndrome/symptoms-causes.

11. National Institute of Diabetes and Digestive and Kidney Diseases. Diagnosis of Interstitial Cystitis. Last reviewed July 2024. Available from: https://www.niddk.nih.gov/health-information/urologic-diseases/interstitial-cystitis-bladder-pain-syndrome/diagnosis.

12. National Institute of Diabetes and Digestive and Kidney Diseases. Treatment for Interstitial Cystitis. Last reviewed July 2024. Available from: https://www.niddk.nih.gov/health-information/urologic-diseases/interstitial-cystitis-bladder-pain-syndrome/treatment.

13. National Institute of Diabetes and Digestive and Kidney Diseases. Eating, Diet, and Nutrition for Interstitial Cystitis. Last reviewed July 2024. Available from: https://www.niddk.nih.gov/health-information/urologic-diseases/interstitial-cystitis-bladder-pain-syndrome/eating-diet-nutrition.

14. Park JJ, Kim KT, Lee EJ, et al. Current updates relating to treatment for interstitial cystitis/bladder pain syndrome: systematic review and network meta-analysis. BMC Urol. 2024;24:95. doi:10.1186/s12894-024-01485-w.

15. FitzGerald MP, Payne CK, Lukacz ES, et al. Randomized multicenter clinical trial of myofascial physical therapy in women with interstitial cystitis/painful bladder syndrome and pelvic floor tenderness. J Urol. 2012;187(6):2113-2118. doi:10.1016/j.juro.2012.01.123.

16. Sobti A, Shawer S, Ballard P, Khunda A. Bladder pain syndrome and sexual function: a systematic review and meta-analysis. Int Urogynecol J. 2023;34(10):2359-2371. doi:10.1007/s00192-023-05633-y.

17. Fischer-Grote L, Fössing V, Aigner M, Boeckle M, Fehrmann E. Comorbidities of bladder pain syndrome in the context of the HiTOP distress category: a systematic review and meta-analysis. Int Urogynecol J. 2022;33:2335-2356. doi:10.1007/s00192-022-05129-1.

18. McKernan LC, Walsh CG, Reynolds WS, Crofford LJ, Dmochowski RR, Williams DA. Psychosocial comorbidities in interstitial cystitis/bladder pain syndrome: a systematic review. Neurourol Urodyn. 2018;37(3):926-941. doi:10.1002/nau.23421.

19. Barker ES, Clemens JQ, Perera RA, et al. Urologic chronic pelvic pain syndrome flares: a comprehensive, systematic review and meta-analysis of the peer-reviewed flare literature. J Urol. 2024;211(3):344-354. doi:10.1097/JU.0000000000003807.

20. American College of Obstetricians and Gynecologists. Urinary tract infections in pregnant individuals. Clinical Consensus No. 4. Obstet Gynecol. 2023;142(2):435-445. doi:10.1097/AOG.0000000000005269.

21. Janssen Pharmaceuticals. ELMIRON (pentosan polysulfate sodium) prescribing information. Revised June 2020. Available from: https://www.accessdata.fda.gov/drugsatfda_docs/label/2020/020193s014lbl.pdf.

22. European Medicines Agency. Elmiron: EPAR medicine overview. Available from: https://www.ema.europa.eu/en/medicines/human/EPAR/elmiron.

23. Friedlander JI, Shorter B, Moldwin RM. Diet and its role in interstitial cystitis/bladder pain syndrome and comorbid conditions. BJU Int. 2012;109(11):1584-1591. doi:10.1111/j.1464-410X.2011.10860.x.
