# Supplementary material for: Safety, accuracy, empathic communication, information quality, and readability of five large language model interfaces answering public questions about interstitial cystitis/bladder pain syndrome
Source: Front Public Health. 2026 Aug 24;14:1942399. doi: 10.3389/fpubh.2026.1942399 (PMC13547468; doi:10.3389/fpubh.2026.1942399)
Supplement: Supplementary file 6 [file Supplementary_file_6.docx]

**Pairwise comparisons of safety**

| Comparison | N pairs | Safe rate difference, % | Safe rate difference 95% CI | Safe to unsafe, n | Unsafe to safe, n | Matched OR | Matched OR 95% CI | Adjusted *p* | Note |
| --- | --- | --- | --- | --- | --- | --- | --- | --- | --- |
| ChatGPT vs Copilot | 58 | 1.72 | -6.90-10.34 | 4 | 3 | 1.33 | 0.30-5.96 | 1.000 | Pairwise McNemar test |
| ChatGPT vs DeepSeek | 58 | 3.45 | -5.17-12.07 | 4 | 2 | 2.00 | 0.37-10.92 | 1.000 | Pairwise McNemar test |
| ChatGPT vs Doubao | 58 | 3.45 | -5.17-12.07 | 4 | 2 | 2.00 | 0.37-10.92 | 1.000 | Pairwise McNemar test |
| ChatGPT vs Gemini | 58 | 1.72 | -3.45-6.90 | 2 | 1 | 2.00 | 0.18-22.06 | 1.000 | Pairwise McNemar test |
| Copilot vs DeepSeek | 58 | 1.72 | -5.17-8.62 | 3 | 2 | 1.50 | 0.25-8.98 | 1.000 | Pairwise McNemar test |
| Copilot vs Doubao | 58 | 1.72 | -5.17-8.62 | 3 | 2 | 1.50 | 0.25-8.98 | 1.000 | Pairwise McNemar test |
| Copilot vs Gemini | 58 | 0.00 | -8.62-8.62 | 3 | 3 | 1.00 | 0.20-4.95 | 1.000 | Pairwise McNemar test |
| DeepSeek vs Doubao | 58 | 0.00 | -8.62-8.62 | 3 | 3 | 1.00 | 0.20-4.95 | 1.000 | Pairwise McNemar test |
| DeepSeek vs Gemini | 58 | -1.72 | -10.34-5.17 | 2 | 3 | 0.67 | 0.11-3.99 | 1.000 | Pairwise McNemar test |
| Doubao vs Gemini | 58 | -1.72 | -10.34-6.90 | 3 | 4 | 0.75 | 0.17-3.35 | 1.000 | Pairwise McNemar test |

**Note:** Pairwise comparisons were performed using McNemar tests with Benjamini-Hochberg correction. Safe-rate differences and their bootstrap 95% CIs are reported.
