# Supplementary material for: Safety, accuracy, empathic communication, information quality, and readability of five large language model interfaces answering public questions about interstitial cystitis/bladder pain syndrome
Source: Front Public Health. 2026 Aug 24;14:1942399. doi: 10.3389/fpubh.2026.1942399 (PMC13547468; doi:10.3389/fpubh.2026.1942399)
Supplement: Supplementary file 7 [file Supplementary_file_7.docx]

**Accuracy, empathy, reliability and readability: Significant pairwise comparisons of non-binary outcomes.**

| **Metric** | **Comparison** | **N pairs** | **Mean difference** | **Mean difference 95% CI** | **Median difference** | **Median difference 95% CI** | **Rank-biserial correlation** | **Rank-biserial 95% CI** | **Adjusted *p*** | **Note** |
| --- | --- | --- | --- | --- | --- | --- | --- | --- | --- | --- |
| Accuracy | ChatGPT vs Copilot | 58 | 0.33 | 0.14-0.52 | 0.16 | 0.00-0.33 | 0.544 | 0.250-0.798 | 0.004 | Paired Wilcoxon signed-rank test |
| Accuracy | ChatGPT vs DeepSeek | 58 | 0.69 | 0.52-0.86 | 0.67 | 0.66-1.00 | 0.895 | 0.758-0.980 | <0.001 | Paired Wilcoxon signed-rank test |
| Accuracy | ChatGPT vs Doubao | 58 | 0.74 | 0.57-0.90 | 1.00 | 0.50-1.00 | 0.941 | 0.825-1.000 | <0.001 | Paired Wilcoxon signed-rank test |
| Accuracy | ChatGPT vs Gemini | 58 | 0.20 | 0.03-0.36 | 0.00 | 0.00-0.33 | 0.383 | 0.015-0.731 | 0.061 | Paired Wilcoxon signed-rank test |
| Accuracy | Copilot vs DeepSeek | 58 | 0.36 | 0.21-0.51 | 0.33 | 0.00-0.67 | 0.672 | 0.420-0.870 | <0.001 | Paired Wilcoxon signed-rank test |
| Accuracy | Copilot vs Doubao | 58 | 0.41 | 0.23-0.58 | 0.67 | 0.33-1.00 | 0.629 | 0.376-0.840 | <0.001 | Paired Wilcoxon signed-rank test |
| Accuracy | Copilot vs Gemini | 58 | -0.13 | -0.30-0.05 | 0.00 | -0.33-0.00 | -0.230 | -0.544-0.132 | 0.233 | Paired Wilcoxon signed-rank test |
| Accuracy | DeepSeek vs Doubao | 58 | 0.05 | -0.10-0.19 | 0.00 | 0.00-0.00 | 0.107 | -0.283-0.523 | 0.606 | Paired Wilcoxon signed-rank test |
| Accuracy | DeepSeek vs Gemini | 58 | -0.49 | -0.65--0.32 | -0.67 | -1.00-0.00 | -0.853 | -0.959--0.678 | <0.001 | Paired Wilcoxon signed-rank test |
| Accuracy | Doubao vs Gemini | 58 | -0.53 | -0.71--0.36 | -0.67 | -1.00--0.16 | -0.793 | -0.952--0.586 | <0.001 | Paired Wilcoxon signed-rank test |
| Empathy | ChatGPT vs Copilot | 58 | 0.15 | -0.03-0.33 | 0.00 | 0.00-0.33 | 0.277 | -0.058-0.578 | 0.137 | Paired Wilcoxon signed-rank test |
| Empathy | ChatGPT vs DeepSeek | 58 | -0.52 | -0.70--0.33 | -0.50 | -1.00--0.33 | -0.692 | -0.874--0.459 | <0.001 | Paired Wilcoxon signed-rank test |
| Empathy | ChatGPT vs Doubao | 58 | 0.33 | 0.14-0.52 | 0.33 | 0.00-0.66 | 0.493 | 0.186-0.772 | 0.007 | Paired Wilcoxon signed-rank test |
| Empathy | ChatGPT vs Gemini | 58 | 0.04 | -0.15-0.24 | 0.00 | 0.00-0.00 | 0.091 | -0.248-0.418 | 0.616 | Paired Wilcoxon signed-rank test |
| Empathy | Copilot vs DeepSeek | 58 | -0.67 | -0.87--0.46 | -0.49 | -1.00--0.33 | -0.829 | -0.964--0.638 | <0.001 | Paired Wilcoxon signed-rank test |
| Empathy | Copilot vs Doubao | 58 | 0.18 | 0.02-0.33 | 0.00 | 0.00-0.33 | 0.383 | 0.035-0.694 | 0.067 | Paired Wilcoxon signed-rank test |
| Empathy | Copilot vs Gemini | 58 | -0.11 | -0.30-0.09 | 0.00 | -0.33-0.00 | -0.178 | -0.518-0.168 | 0.344 | Paired Wilcoxon signed-rank test |
| Empathy | DeepSeek vs Doubao | 58 | 0.84 | 0.65-1.02 | 1.00 | 0.49-1.16 | 0.945 | 0.852-1.000 | <0.001 | Paired Wilcoxon signed-rank test |
| Empathy | DeepSeek vs Gemini | 58 | 0.56 | 0.38-0.73 | 0.66 | 0.33-1.00 | 0.840 | 0.662-0.969 | <0.001 | Paired Wilcoxon signed-rank test |
| Empathy | Doubao vs Gemini | 58 | -0.29 | -0.48--0.10 | -0.33 | -0.33-0.00 | -0.463 | -0.747--0.143 | 0.017 | Paired Wilcoxon signed-rank test |
| DISCERN | ChatGPT vs Copilot | 58 | 5.11 | 4.25-5.96 | 5.66 | 4.00-7.00 | 0.978 | 0.931-1.000 | <0.001 | Paired Wilcoxon signed-rank test |
| DISCERN | ChatGPT vs DeepSeek | 58 | 8.09 | 7.28-8.83 | 8.34 | 7.00-9.34 | 0.999 | 0.993-1.000 | <0.001 | Paired Wilcoxon signed-rank test |
| DISCERN | ChatGPT vs Doubao | 58 | 6.40 | 5.60-7.19 | 6.67 | 6.00-7.67 | 0.995 | 0.979-1.000 | <0.001 | Paired Wilcoxon signed-rank test |
| DISCERN | ChatGPT vs Gemini | 58 | 2.23 | 1.62-2.86 | 2.16 | 1.00-3.33 | 0.778 | 0.592-0.914 | <0.001 | Paired Wilcoxon signed-rank test |
| DISCERN | Copilot vs DeepSeek | 58 | 2.98 | 2.41-3.51 | 3.00 | 2.33-3.67 | 0.960 | 0.876-1.000 | <0.001 | Paired Wilcoxon signed-rank test |
| DISCERN | Copilot vs Doubao | 58 | 1.29 | 0.70-1.87 | 1.16 | 0.33-2.16 | 0.579 | 0.324-0.783 | <0.001 | Paired Wilcoxon signed-rank test |
| DISCERN | Copilot vs Gemini | 58 | -2.88 | -3.58--2.15 | -3.00 | -3.84--2.16 | -0.857 | -0.968--0.704 | <0.001 | Paired Wilcoxon signed-rank test |
| DISCERN | DeepSeek vs Doubao | 58 | -1.69 | -2.22--1.14 | -1.50 | -2.50--0.67 | -0.763 | -0.920--0.560 | <0.001 | Paired Wilcoxon signed-rank test |
| DISCERN | DeepSeek vs Gemini | 58 | -5.86 | -6.65--5.10 | -5.67 | -6.50--4.83 | -0.995 | -1.000--0.979 | <0.001 | Paired Wilcoxon signed-rank test |
| DISCERN | Doubao vs Gemini | 58 | -4.17 | -4.88--3.48 | -4.33 | -5.34--3.00 | -0.971 | -1.000--0.914 | <0.001 | Paired Wilcoxon signed-rank test |
| EQIP | ChatGPT vs Copilot | 58 | 5.06 | 4.15-5.90 | 5.33 | 4.67-6.34 | 0.933 | 0.834-0.998 | <0.001 | Paired Wilcoxon signed-rank test |
| EQIP | ChatGPT vs DeepSeek | 58 | 13.82 | 12.84-14.84 | 13.33 | 12.00-15.00 | 1.000 | 1.000-1.000 | <0.001 | Paired Wilcoxon signed-rank test |
| EQIP | ChatGPT vs Doubao | 58 | 17.56 | 16.56-18.49 | 17.33 | 16.00-18.67 | 1.000 | 1.000-1.000 | <0.001 | Paired Wilcoxon signed-rank test |
| EQIP | ChatGPT vs Gemini | 58 | 6.89 | 6.04-7.74 | 7.00 | 5.83-8.00 | 1.000 | 1.000-1.000 | <0.001 | Paired Wilcoxon signed-rank test |
| EQIP | Copilot vs DeepSeek | 58 | 8.76 | 7.79-9.70 | 8.00 | 7.33-9.33 | 1.000 | 1.000-1.000 | <0.001 | Paired Wilcoxon signed-rank test |
| EQIP | Copilot vs Doubao | 58 | 12.49 | 11.55-13.40 | 12.66 | 11.34-13.50 | 1.000 | 1.000-1.000 | <0.001 | Paired Wilcoxon signed-rank test |
| EQIP | Copilot vs Gemini | 58 | 1.82 | 0.82-2.84 | 1.67 | 0.34-3.16 | 0.493 | 0.217-0.736 | 0.002 | Paired Wilcoxon signed-rank test |
| EQIP | DeepSeek vs Doubao | 58 | 3.74 | 2.81-4.64 | 4.33 | 3.00-5.33 | 0.842 | 0.690-0.949 | <0.001 | Paired Wilcoxon signed-rank test |
| EQIP | DeepSeek vs Gemini | 58 | -6.94 | -8.00--5.85 | -7.33 | -8.67--6.50 | -0.979 | -1.000--0.936 | <0.001 | Paired Wilcoxon signed-rank test |
| EQIP | Doubao vs Gemini | 58 | -10.67 | -11.62--9.70 | -11.33 | -11.67--9.34 | -1.000 | -1.000--1.000 | <0.001 | Paired Wilcoxon signed-rank test |
| JAMA | ChatGPT vs Copilot | 58 | 0.41 | 0.31-0.52 | 0.33 | 0.00-0.67 | 1.000 | 1.000-1.000 | <0.001 | Paired Wilcoxon signed-rank test |
| JAMA | ChatGPT vs DeepSeek | 58 | 0.89 | 0.82-0.95 | 1.00 | 1.00-1.00 | 1.000 | 1.000-1.000 | <0.001 | Paired Wilcoxon signed-rank test |
| JAMA | ChatGPT vs Doubao | 58 | 1.11 | 1.06-1.18 | 1.00 | 1.00-1.00 | 1.000 | 1.000-1.000 | <0.001 | Paired Wilcoxon signed-rank test |
| JAMA | ChatGPT vs Gemini | 58 | -0.34 | -0.44--0.26 | -0.33 | -0.33-0.00 | -1.000 | -1.000--1.000 | <0.001 | Paired Wilcoxon signed-rank test |
| JAMA | Copilot vs DeepSeek | 58 | 0.48 | 0.37-0.59 | 0.33 | 0.33-0.67 | 1.000 | 1.000-1.000 | <0.001 | Paired Wilcoxon signed-rank test |
| JAMA | Copilot vs Doubao | 58 | 0.70 | 0.62-0.78 | 0.67 | 0.67-1.00 | 1.000 | 1.000-1.000 | <0.001 | Paired Wilcoxon signed-rank test |
| JAMA | Copilot vs Gemini | 58 | -0.76 | -0.84--0.68 | -0.67 | -1.00--0.67 | -1.000 | -1.000--1.000 | <0.001 | Paired Wilcoxon signed-rank test |
| JAMA | DeepSeek vs Doubao | 58 | 0.22 | 0.14-0.32 | 0.00 | 0.00-0.33 | 1.000 | 1.000-1.000 | <0.001 | Paired Wilcoxon signed-rank test |
| JAMA | DeepSeek vs Gemini | 58 | -1.24 | -1.32--1.16 | -1.00 | -1.33--1.00 | -1.000 | -1.000--1.000 | <0.001 | Paired Wilcoxon signed-rank test |
| JAMA | Doubao vs Gemini | 58 | -1.46 | -1.55--1.37 | -1.33 | -1.67--1.33 | -1.000 | -1.000--1.000 | <0.001 | Paired Wilcoxon signed-rank test |
| GQS | ChatGPT vs Copilot | 58 | 0.87 | 0.80-0.94 | 1.00 | 0.67-1.00 | 1.000 | 1.000-1.000 | <0.001 | Paired Wilcoxon signed-rank test |
| GQS | ChatGPT vs DeepSeek | 58 | 1.24 | 1.15-1.33 | 1.00 | 1.00-1.33 | 1.000 | 1.000-1.000 | <0.001 | Paired Wilcoxon signed-rank test |
| GQS | ChatGPT vs Doubao | 58 | 1.57 | 1.47-1.66 | 1.67 | 1.33-1.67 | 1.000 | 1.000-1.000 | <0.001 | Paired Wilcoxon signed-rank test |
| GQS | ChatGPT vs Gemini | 58 | 0.41 | 0.32-0.51 | 0.33 | 0.33-0.50 | 1.000 | 1.000-1.000 | <0.001 | Paired Wilcoxon signed-rank test |
| GQS | Copilot vs DeepSeek | 58 | 0.37 | 0.29-0.47 | 0.33 | 0.33-0.33 | 1.000 | 1.000-1.000 | <0.001 | Paired Wilcoxon signed-rank test |
| GQS | Copilot vs Doubao | 58 | 0.70 | 0.62-0.78 | 0.67 | 0.67-1.00 | 1.000 | 1.000-1.000 | <0.001 | Paired Wilcoxon signed-rank test |
| GQS | Copilot vs Gemini | 58 | -0.46 | -0.55--0.36 | -0.33 | -0.67--0.33 | -1.000 | -1.000--1.000 | <0.001 | Paired Wilcoxon signed-rank test |
| GQS | DeepSeek vs Doubao | 58 | 0.33 | 0.25-0.41 | 0.33 | 0.00-0.34 | 1.000 | 1.000-1.000 | <0.001 | Paired Wilcoxon signed-rank test |
| GQS | DeepSeek vs Gemini | 58 | -0.83 | -0.91--0.75 | -1.00 | -1.00--1.00 | -1.000 | -1.000--1.000 | <0.001 | Paired Wilcoxon signed-rank test |
| GQS | Doubao vs Gemini | 58 | -1.16 | -1.24--1.10 | -1.00 | -1.16--1.00 | -1.000 | -1.000--1.000 | <0.001 | Paired Wilcoxon signed-rank test |
| ARI | ChatGPT vs Copilot | 58 | 0.01 | -0.99-1.03 | -0.60 | -1.50-0.93 | -0.073 | -0.370-0.213 | 0.631 | Paired Wilcoxon signed-rank test |
| ARI | ChatGPT vs DeepSeek | 58 | 3.54 | 2.49-4.61 | 2.89 | 2.22-3.86 | 0.827 | 0.655-0.957 | <0.001 | Paired Wilcoxon signed-rank test |
| ARI | ChatGPT vs Doubao | 58 | -12.09 | -15.09--9.34 | -9.67 | -14.21--6.51 | -0.918 | -0.992--0.807 | <0.001 | Paired Wilcoxon signed-rank test |
| ARI | ChatGPT vs Gemini | 58 | 1.55 | 0.57-2.55 | 0.94 | -0.12-2.15 | 0.377 | 0.098-0.627 | 0.014 | Paired Wilcoxon signed-rank test |
| ARI | Copilot vs DeepSeek | 58 | 3.53 | 2.68-4.40 | 3.78 | 2.65-4.61 | 0.853 | 0.694-0.979 | <0.001 | Paired Wilcoxon signed-rank test |
| ARI | Copilot vs Doubao | 58 | -12.09 | -14.89--9.49 | -9.95 | -13.96--5.43 | -0.953 | -0.992--0.882 | <0.001 | Paired Wilcoxon signed-rank test |
| ARI | Copilot vs Gemini | 58 | 1.54 | 0.75-2.30 | 1.95 | -0.10-2.92 | 0.497 | 0.234-0.720 | 0.001 | Paired Wilcoxon signed-rank test |
| ARI | DeepSeek vs Doubao | 58 | -15.62 | -18.38--12.86 | -13.33 | -17.67--9.48 | -1.000 | -1.000--1.000 | <0.001 | Paired Wilcoxon signed-rank test |
| ARI | DeepSeek vs Gemini | 58 | -1.99 | -2.64--1.29 | -2.35 | -3.10--1.60 | -0.702 | -0.885--0.488 | <0.001 | Paired Wilcoxon signed-rank test |
| ARI | Doubao vs Gemini | 58 | 13.63 | 10.65-16.71 | 10.66 | 7.13-14.87 | 0.976 | 0.925-0.999 | <0.001 | Paired Wilcoxon signed-rank test |
| CL | ChatGPT vs Copilot | 58 | -2.14 | -2.64--1.65 | -2.19 | -2.91--1.77 | -0.882 | -0.972--0.756 | <0.001 | Paired Wilcoxon signed-rank test |
| CL | ChatGPT vs DeepSeek | 58 | 1.29 | 0.83-1.77 | 1.37 | 0.88-1.69 | 0.664 | 0.445-0.853 | <0.001 | Paired Wilcoxon signed-rank test |
| CL | ChatGPT vs Doubao | 58 | -2.75 | -3.16--2.36 | -2.89 | -3.08--2.39 | -0.983 | -1.000--0.942 | <0.001 | Paired Wilcoxon signed-rank test |
| CL | ChatGPT vs Gemini | 58 | 0.65 | 0.22-1.08 | 0.56 | 0.01-1.15 | 0.392 | 0.114-0.653 | 0.012 | Paired Wilcoxon signed-rank test |
| CL | Copilot vs DeepSeek | 58 | 3.43 | 2.87-3.98 | 3.51 | 2.52-4.04 | 0.953 | 0.857-1.000 | <0.001 | Paired Wilcoxon signed-rank test |
| CL | Copilot vs Doubao | 58 | -0.62 | -1.07--0.17 | -0.45 | -0.92--0.02 | -0.363 | -0.612--0.079 | 0.016 | Paired Wilcoxon signed-rank test |
| CL | Copilot vs Gemini | 58 | 2.79 | 2.27-3.32 | 2.82 | 1.94-3.43 | 0.940 | 0.840-0.997 | <0.001 | Paired Wilcoxon signed-rank test |
| CL | DeepSeek vs Doubao | 58 | -4.05 | -4.49--3.61 | -4.13 | -4.41--3.33 | -1.000 | -1.000--1.000 | <0.001 | Paired Wilcoxon signed-rank test |
| CL | DeepSeek vs Gemini | 58 | -0.64 | -1.14--0.16 | -0.88 | -1.18-0.03 | -0.379 | -0.627--0.090 | 0.013 | Paired Wilcoxon signed-rank test |
| CL | Doubao vs Gemini | 58 | 3.40 | 2.98-3.83 | 3.52 | 3.17-3.78 | 0.998 | 0.986-1.000 | <0.001 | Paired Wilcoxon signed-rank test |
| FKGL | ChatGPT vs Copilot | 58 | -0.47 | -1.23-0.34 | -0.90 | -1.46--0.06 | -0.242 | -0.515-0.050 | 0.109 | Paired Wilcoxon signed-rank test |
| FKGL | ChatGPT vs DeepSeek | 58 | 2.52 | 1.70-3.41 | 2.06 | 1.52-2.89 | 0.773 | 0.587-0.918 | <0.001 | Paired Wilcoxon signed-rank test |
| FKGL | ChatGPT vs Doubao | 58 | -10.12 | -12.46--7.94 | -8.12 | -11.63--6.15 | -0.927 | -0.993--0.822 | <0.001 | Paired Wilcoxon signed-rank test |
| FKGL | ChatGPT vs Gemini | 58 | 1.03 | 0.26-1.87 | 0.42 | -0.40-1.11 | 0.273 | -0.026-0.544 | 0.079 | Paired Wilcoxon signed-rank test |
| FKGL | Copilot vs DeepSeek | 58 | 3.00 | 2.35-3.68 | 2.94 | 2.43-3.53 | 0.867 | 0.707-0.981 | <0.001 | Paired Wilcoxon signed-rank test |
| FKGL | Copilot vs Doubao | 58 | -9.65 | -11.77--7.58 | -7.98 | -11.42--4.60 | -0.958 | -0.993--0.889 | <0.001 | Paired Wilcoxon signed-rank test |
| FKGL | Copilot vs Gemini | 58 | 1.51 | 0.91-2.11 | 1.61 | 0.58-2.47 | 0.618 | 0.389-0.812 | <0.001 | Paired Wilcoxon signed-rank test |
| FKGL | DeepSeek vs Doubao | 58 | -12.64 | -14.92--10.46 | -11.24 | -14.68--7.47 | -1.000 | -1.000--1.000 | <0.001 | Paired Wilcoxon signed-rank test |
| FKGL | DeepSeek vs Gemini | 58 | -1.49 | -2.05--0.91 | -1.78 | -2.39--0.98 | -0.667 | -0.847--0.435 | <0.001 | Paired Wilcoxon signed-rank test |
| FKGL | Doubao vs Gemini | 58 | 11.16 | 8.83-13.55 | 8.92 | 6.06-11.93 | 0.983 | 0.943-1.000 | <0.001 | Paired Wilcoxon signed-rank test |
| GFI | ChatGPT vs Copilot | 58 | -0.98 | -1.78--0.14 | -1.33 | -1.96--0.36 | -0.383 | -0.643--0.087 | 0.013 | Paired Wilcoxon signed-rank test |
| GFI | ChatGPT vs DeepSeek | 58 | 2.31 | 1.44-3.23 | 2.12 | 1.27-2.90 | 0.669 | 0.465-0.855 | <0.001 | Paired Wilcoxon signed-rank test |
| GFI | ChatGPT vs Doubao | 58 | -10.93 | -13.30--8.67 | -9.14 | -12.46--6.97 | -0.951 | -0.994--0.873 | <0.001 | Paired Wilcoxon signed-rank test |
| GFI | ChatGPT vs Gemini | 58 | 0.94 | 0.10-1.82 | 0.11 | -0.41-1.26 | 0.224 | -0.084-0.497 | 0.139 | Paired Wilcoxon signed-rank test |
| GFI | Copilot vs DeepSeek | 58 | 3.29 | 2.50-4.07 | 3.27 | 2.47-3.90 | 0.856 | 0.703-0.966 | <0.001 | Paired Wilcoxon signed-rank test |
| GFI | Copilot vs Doubao | 58 | -9.95 | -12.16--7.79 | -8.64 | -11.90--5.21 | -0.965 | -0.994--0.903 | <0.001 | Paired Wilcoxon signed-rank test |
| GFI | Copilot vs Gemini | 58 | 1.92 | 1.25-2.63 | 1.67 | 0.72-2.78 | 0.674 | 0.447-0.847 | <0.001 | Paired Wilcoxon signed-rank test |
| GFI | DeepSeek vs Doubao | 58 | -13.24 | -15.59--11.00 | -11.08 | -14.78--8.37 | -1.000 | -1.000--1.000 | <0.001 | Paired Wilcoxon signed-rank test |
| GFI | DeepSeek vs Gemini | 58 | -1.38 | -2.02--0.78 | -1.47 | -2.16--0.65 | -0.568 | -0.779--0.324 | <0.001 | Paired Wilcoxon signed-rank test |
| GFI | Doubao vs Gemini | 58 | 11.87 | 9.44-14.23 | 9.73 | 7.08-12.22 | 0.987 | 0.957-0.999 | <0.001 | Paired Wilcoxon signed-rank test |
| SMOG | ChatGPT vs Copilot | 58 | -0.56 | -1.13--0.00 | -0.90 | -1.33-0.39 | -0.297 | -0.560--0.020 | 0.055 | Paired Wilcoxon signed-rank test |
| SMOG | ChatGPT vs DeepSeek | 58 | 1.62 | 0.99-2.25 | 1.55 | 1.02-2.22 | 0.683 | 0.459-0.856 | <0.001 | Paired Wilcoxon signed-rank test |
| SMOG | ChatGPT vs Doubao | 58 | -6.76 | -8.07--5.47 | -6.34 | -8.08--4.68 | -0.959 | -0.995--0.891 | <0.001 | Paired Wilcoxon signed-rank test |
| SMOG | ChatGPT vs Gemini | 58 | 0.57 | 0.02-1.16 | 0.09 | -0.36-0.93 | 0.205 | -0.100-0.484 | 0.175 | Paired Wilcoxon signed-rank test |
| SMOG | Copilot vs DeepSeek | 58 | 2.18 | 1.59-2.74 | 2.22 | 1.44-2.73 | 0.819 | 0.646-0.951 | <0.001 | Paired Wilcoxon signed-rank test |
| SMOG | Copilot vs Doubao | 58 | -6.20 | -7.45--5.05 | -5.85 | -7.96--3.94 | -0.963 | -0.997--0.899 | <0.001 | Paired Wilcoxon signed-rank test |
| SMOG | Copilot vs Gemini | 58 | 1.13 | 0.62-1.62 | 1.12 | 0.26-1.78 | 0.549 | 0.287-0.760 | <0.001 | Paired Wilcoxon signed-rank test |
| SMOG | DeepSeek vs Doubao | 58 | -8.38 | -9.64--7.12 | -7.47 | -10.07--6.02 | -1.000 | -1.000--1.000 | <0.001 | Paired Wilcoxon signed-rank test |
| SMOG | DeepSeek vs Gemini | 58 | -1.05 | -1.51--0.59 | -1.19 | -1.73--0.59 | -0.579 | -0.792--0.318 | <0.001 | Paired Wilcoxon signed-rank test |
| SMOG | Doubao vs Gemini | 58 | 7.33 | 6.01-8.66 | 6.74 | 4.74-8.29 | 0.980 | 0.938-0.999 | <0.001 | Paired Wilcoxon signed-rank test |
| FRES | ChatGPT vs Copilot | 58 | 4.93 | 2.47-7.32 | 5.44 | 1.28-8.35 | 0.548 | 0.283-0.767 | <0.001 | Paired Wilcoxon signed-rank test |
| FRES | ChatGPT vs DeepSeek | 58 | -8.73 | -11.29--6.19 | -8.19 | -10.43--6.05 | -0.819 | -0.946--0.641 | <0.001 | Paired Wilcoxon signed-rank test |
| FRES | ChatGPT vs Doubao | 58 | 6.88 | 4.38-9.37 | 7.24 | 4.47-9.45 | 0.693 | 0.480-0.874 | <0.001 | Paired Wilcoxon signed-rank test |
| FRES | ChatGPT vs Gemini | 58 | -4.56 | -6.90--2.21 | -3.36 | -7.62--1.25 | -0.509 | -0.736--0.266 | 0.001 | Paired Wilcoxon signed-rank test |
| FRES | Copilot vs DeepSeek | 58 | -13.66 | -16.34--10.93 | -12.13 | -18.12--10.04 | -0.924 | -0.995--0.801 | <0.001 | Paired Wilcoxon signed-rank test |
| FRES | Copilot vs Doubao | 58 | 1.95 | -0.28-4.26 | 0.93 | -1.37-3.83 | 0.206 | -0.096-0.496 | 0.173 | Paired Wilcoxon signed-rank test |
| FRES | Copilot vs Gemini | 58 | -9.49 | -12.02--7.04 | -10.06 | -13.77--5.32 | -0.816 | -0.940--0.655 | <0.001 | Paired Wilcoxon signed-rank test |
| FRES | DeepSeek vs Doubao | 58 | 15.61 | 13.25-17.84 | 14.90 | 11.57-17.31 | 0.988 | 0.958-1.000 | <0.001 | Paired Wilcoxon signed-rank test |
| FRES | DeepSeek vs Gemini | 58 | 4.17 | 1.60-6.81 | 4.70 | 0.54-6.58 | 0.421 | 0.134-0.669 | 0.006 | Paired Wilcoxon signed-rank test |
| FRES | Doubao vs Gemini | 58 | -11.44 | -13.72--9.15 | -11.04 | -13.26--9.08 | -0.939 | -1.000--0.832 | <0.001 | Paired Wilcoxon signed-rank test |

**Note:** Pairwise comparisons were performed using paired Wilcoxon signed-rank tests with Benjamini-Hochberg correction. Paired mean/median differences and rank-biserial correlations are reported with bootstrap 95% CIs.
